# Loss of Notch signaling in skeletal stem cells enhances bone formation with aging

**DOI:** 10.1038/s41413-023-00283-8

**Published:** 2023-09-27

**Authors:** Lindsey H. Remark, Kevin Leclerc, Malissa Ramsukh, Ziyan Lin, Sooyeon Lee, Backialakshmi Dharmalingam, Lauren Gillinov, Vasudev V. Nayak, Paulo El Parente, Margaux Sambon, Pablo J. Atria, Mohamed A. E. Ali, Lukasz Witek, Alesha B. Castillo, Christopher Y, Park, Ralf H. Adams, Aristotelis Tsirigos, Sophie M. Morgani, Philipp Leucht

**Affiliations:** 1 Department of Orthopaedic Surgery, NYU Robert I. Grossman School of Medicine, New York, NY USA; 2 Applied Bioinformatics Laboratories, NYU Grossman School of Medicine, New York, NY USA; 3 Institute of Comparative Molecular Endocrinology, Ulm University, Ulm, Germany; 4 Max Planck Institute for Molecular Biomedicine, Department of Tissue Morphogenesis, and University of Münster, Faculty of Medicine, D-48149 Münster, Germany; 5 Department of Biochemistry and Molecular Biology, University of Miami Miller School of Medicine, Miami, FL USA; 6 Department of Pathology, NYU Robert I. Grossman School of Medicine, New York, NY USA; 7 Biomaterials Division, New York University College of Dentistry, New York, NY USA; 8 Hansjörg Wyss Department of Plastic Surgery, NYU Grossman School of Medicine, New York University, New York, NY USA; 9 Department of Biomedical Engineering, Tandon School of Engineering, New York University, Brooklyn, NY USA; 10 Department of Cell Biology, NYU Robert I. Grossman School of Medicine, New York, NY USA

**Keywords:** Bone, Pathogenesis

## Abstract

Skeletal stem and progenitor cells (SSPCs) perform bone maintenance and repair. With age, they produce fewer osteoblasts and more adipocytes leading to a loss of skeletal integrity. The molecular mechanisms that underlie this detrimental transformation are largely unknown. Single-cell RNA sequencing revealed that Notch signaling becomes elevated in SSPCs during aging. To examine the role of increased Notch activity, we deleted Nicastrin, an essential Notch pathway component, in SSPCs in vivo. Middle-aged conditional knockout mice displayed elevated SSPC osteo-lineage gene expression, increased trabecular bone mass, reduced bone marrow adiposity, and enhanced bone repair. Thus, Notch regulates SSPC cell fate decisions, and moderating Notch signaling ameliorates the skeletal aging phenotype, increasing bone mass even beyond that of young mice. Finally, we identified the transcription factor *Ebf3* as a downstream mediator of Notch signaling in SSPCs that is dysregulated with aging, highlighting it as a promising therapeutic target to rejuvenate the aged skeleton.

## Introduction

Aging is characterized by a reduction in the osteogenic capacity of skeletal stem and progenitor cells (SSPCs),^1^ the building blocks for bone homeostasis and repair.^2^ Consequently, bone mineral density declines leading to weaker bones that are prone to fracture and repair less efficiently. Deterioration of the skeleton, as in osteoporosis and osteopenia, is one of the most common causes of age-associated impairments.^3^ Current treatments for osteoporosis target the osteoclast, curtailing bone resorption,^4^ and the osteoblast, promoting bone matrix deposition.^4^ To date, there are no therapeutics to rescue or maintain the osteogenic function of aging SSPCs to enhance the anabolic function of the skeleton.

Coincident with geriatric bone loss, there is a reciprocal increase in bone marrow adipose tissue (BMAT). BMAT secretes osteo-inhibitory signals^5,6^ that are prohibitive to bone homeostasis^5,7^ and regeneration,^6,8–11^ exacerbating the aging phenotype. Fatty degeneration of the bone marrow is also associated with a myriad of diseases including diabetes,^12^ cancer,^13^ and compromised hematopoiesis.^14^ As such, it is imperative to elucidate the mechanisms that trigger the detrimental bone-to-fat shift in the skeleton with age to prolong healthspan, preserve skeletal integrity, and enhance the efficiency of fracture healing in the aging population.

SSPCs, marked by the Leptin receptor (*Lepr*), are multi-potent, self-renewing stem cells within the bone marrow that give rise to both osteoblasts and adipocytes.^15^ A plethora of data points towards a progressive switch in the lineage potential of SSPCs, whereby aged SSPCs produce fewer osteoblasts and more adipocytes,^1,6,8,9^ as the underlying cause of skeletal degeneration. However, the molecular mechanisms driving this cell fate switch are still largely unknown.

To address this, we performed single-cell RNA sequencing of young and middle-aged mouse hindlimb bones, finding that Notch-associated genes become dysregulated in SSPCs with age. Recent evidence suggests that Notch signaling is at the core of the aging process.^16^ Notch plays critical roles in development, tissue homeostasis, and disease,^17^ including in skeletal stem cell maintenance during development, fracture healing,^18–20^ and the adipogenic differentiation of mesenchymal stem cells.^21,22^ We found that conditionally deleting an essential component of the Notch pathway, Nicastrin (*Ncstn*), in SSPCs in vivo led to an increase in osteogenic gene expression and enzymatic activity, and osteoprogenitor number. Furthermore, loss of Notch signaling in SSPCs prompted a massive increase in trabecular bone, reduction in bone marrow adiposity, and enhanced bone regeneration in middle-aged mice, effectively curbing skeletal degeneration. Furthermore, we pinpointed the transcription factor *Ebf3* as a downstream target of Notch in SSPCs with a relatively restricted expression pattern, highlighting it as a promising therapeutic target to combat age-related skeletal degeneration.

## Results

### Notch-associated genes become dysregulated in SSPCs during aging

**Fig. 1:**
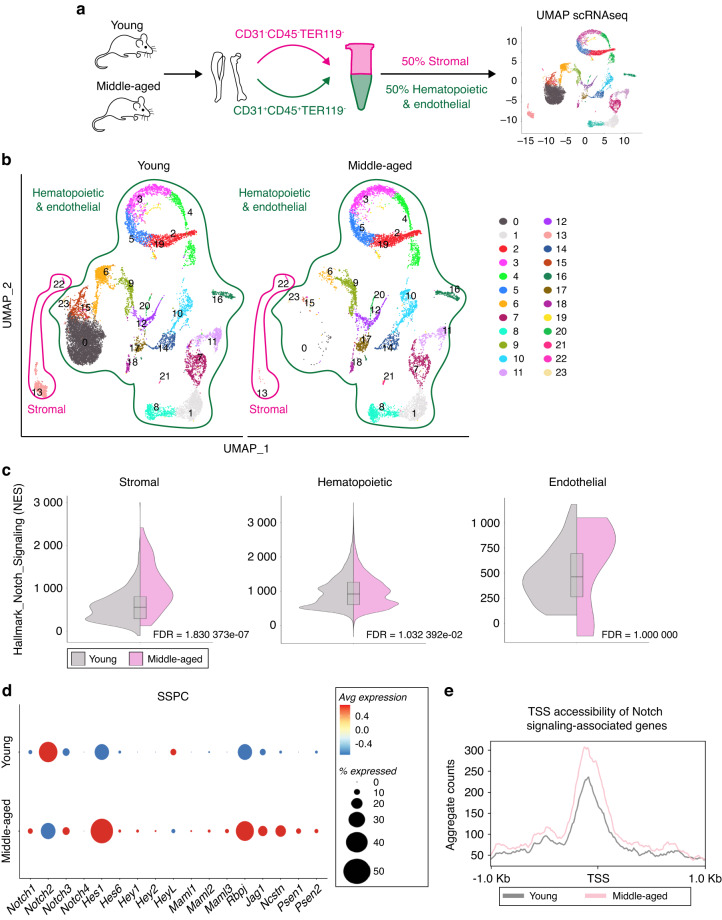
Aging activates Notch signaling genes in skeletal stem cells. **a** Schematic of experimental workflow for scRNAseq of young adult (~3-month-old) and middle-aged (~12-month-old) bone stromal (CD31^−^CD45^−^TER119^−^) and hematopoietic and endothelial compartments (CD31^+^CD45^+^TER119^−^) from hindlimb bone tissue sorted and combined at a 1:1 ratio, then processed for scRNAseq. **b** scRNAseq UMAP-based visualization of stromal, hematopoietic, and endothelial bone and bone marrow populations with 24 clusters. Young *n* = 15 225 cells, Middle-aged *n* = 8 831 cells. The pink outline indicates the stromal compartment and the green outline indicates the hematopoietic and endothelial compartment. **c** Gene set enrichment analysis (GSEA) using the package escape 1.4.1. GSEA analysis was performed on the stromal hematopoietic, and endothelial compartments separately. Split Violin plot shows normalized enrichment score (NES) for hallmark_notch_signaling from the H:hallmark gene sets between young and middle-aged stromal (FDR = 1.830 372e−07, young median NES = 536.031, middle-aged median NES = 927.393), hematopoietic (FDR = 1.032 392e-02, young median NES = 939.566, middle-aged median NES = 899.742), and endothelial populations (FDR = 1.000 000, young median NES = 695.455, middle-aged median NES = 437.276) Wilcoxon test. **d** Dotplot showing expression of Notch signaling pathway genes in cluster 22, which is mainly comprised of *Lepr*^+^ expressing SSPCs between middle-aged and young mice. **e** SSPCs were subjected to ATAC sequencing. Compared to young SSPCs, chromatin in middle-aged SSPCs was more accessible around the transcriptional start site (TSS) in genes associated with Notch signaling

To investigate the molecular etiology of age-related skeletal degeneration, we performed single-cell RNA sequencing (scRNAseq) of the hindlimb skeletal elements of young adult (3-month-old) and middle-aged (12-month-old) mice (Fig. 1a, Fig. S1a, b). We analyzed middle-aged mice (10–14 months) rather than aged (18–24 months) mice to identify factors involved in the progression of skeletal aging rather than in the end product, the irreversibly aged skeleton. Uniform manifold approximation and projection (UMAP) visualization distinguished 24 cell clusters, which we classified as 22 hematopoietic and endothelial and 2 stromal/osteolineage populations based on published scRNAseq data sets^23,24^ (Fig. 1b). Of the 2 stromal clusters, cluster 22 expressed genes characteristic of *Lepr*^+^ SSPCs, including *Lepr*, *Cxcl12*, and *Kitl*^15^ (Table S1). Published scRNAseq studies show that *Lepr*^+^ SSPCs exist in two states: osteo-primed and adipo-primed.^25^ With aging, there is an expansion of the adipo-primed population together with an increase in adipocyte-associated gene expression.^7,26^ We similarly observed an adipogenic shift in the middle-aged *Lepr*^+^ SSPC cluster with increased adipogenic gene expression (*Apoe, Lpl, Cebpa/b, Adipoq*), increased expression of anti-osteogenic BMP inhibitors, and a decrease in pro-osteogenic pathways such as *Bmp4* and *Egfr*^5^ (Fig. S1c).

To explore the pathways responsible for the adipogenic shift with age, we used gene set enrichment analysis (GSEA) to define transcriptional signatures that were enriched in the stromal and osteolineage populations during aging and thus could be involved in age-related skeletal degeneration (Fig. S1d). Evidence from human, young and middle-aged bone samples suggests Notch signaling components to be elevated with aging.^27^ Utilizing the MsigDB “hallmark” gene set,^28^ NOTCH_SIGNALING exhibited a significant increase with aging in stromal/osteo-lineage cells (FDR = 1.830373e−07) (Fig. 1c). We confirmed this observation by qRT-PCR, finding that the expression of key Notch pathway components is elevated within middle-aged bones (Fig. S1e). In contrast, the GSEA NOTCH_SIGNALING did not show a difference in the hematopoietic or endothelial populations (Fig. 1c), although the Notch ligand *Delta4*, *Dll4*, was upregulated in bone marrow endothelial cells with aging, suggesting an increase in Notch signaling activity (Fig. S1f). To interrogate the stromal and osteolineage compartments at higher resolution, we repeated UMAP clustering without hematopoietic and endothelial cells, discerning 5 clusters representing 3 distinct populations (Fig. S2a, b). Cluster 4 was SSPC-like, defined by *Cxcl12, Lepr*, *and Kitl*^15^ (Fig. S2c). Clusters 0–2 resembled more committed osteoprogenitors, expressing *Ly6a* (Sca-1)*, Thy1*,^29^ *Mfap5*,^7^ *Itga5*,^30^ and *Postn*^31^ (Fig. S2d). Cluster 3 was enriched for markers of terminally differentiated osteoblasts, such as *Col1a1, Bglap, Gsn, Clec3b*, and chondrocytes, such as *Comp* and *Acan* (Fig. S2e). The osteoprogenitor (clusters 0–2) and osteo/chondro mature lineage cells (cluster 3) were considerably depleted during aging (Fig. S2b), consistent with the age-related loss of bone mineral density. These analyses also showed that Notch pathway components and downstream targets were elevated primarily in *Lepr*^+^ SSPCs with age (cluster 4) (Fig. 1d and Fig. S3a), consistent with previous human and mouse studies.^27,32^

Aberrant gene expression during aging has been linked to a loss of repressive heterochromatin.^33,34^ To determine whether the transcriptional changes in SSPCs during aging were associated with a transformation of the epigenetic landscape, we performed ATAC-sequencing on young and middle-aged SSPCs. In line with previous observations, middle-aged mice showed a global increase in chromatin accessibility with aging (Fig. S3b). Moreover, chromatin surrounding the transcriptional start site (TSS) of Notch-associated loci became more accessible with age (Fig. 1e), consistent with the observed increase in transcription. Thus, age-related skeletal degeneration is accompanied by epigenetic and transcriptional dysregulation of Notch pathway components in *Lepr*^*+*^ SSPCs.

### Loss of Notch signaling in SSPCs promotes transcriptional osteo-priming

**Fig. 2:**
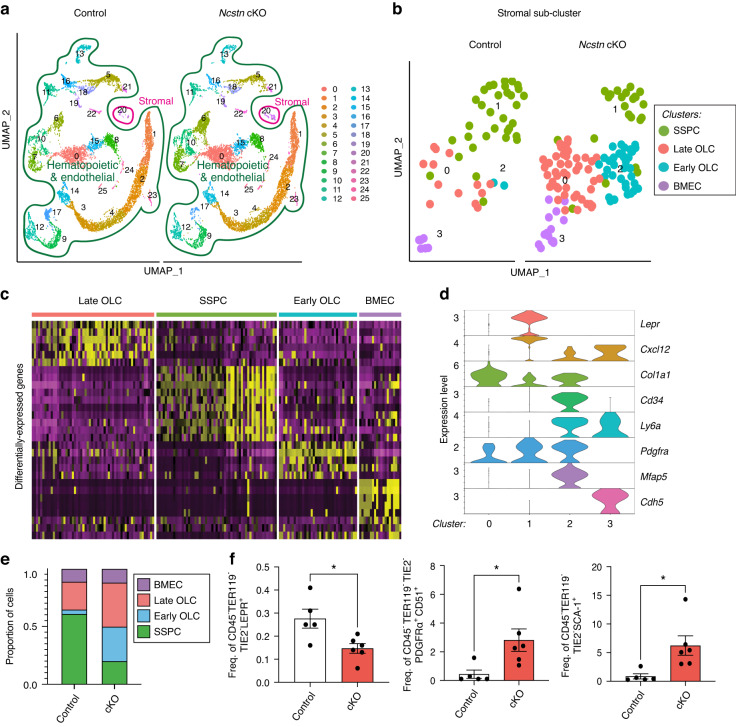
Decreased Notch signaling during aging increases osteogenic and decreases adipogenic gene signatures. **a** UMAP clusters from control *Ncstn*^fl/fl^ (*n* = 8 831 cells) and *Lepr*Cre; *Ncstn*^fl/fl^ cKO (*n* = 9 439 cells) middle-aged digested bone and bone marrow showing a stromal (outlined in pink) and hematopoietic/ endothelial compartment (outlined in green). **b** UMAP after sub-clustering of the stromal compartment of control and *Ncstn* cKO middle-aged mice. SSPC skeletal stem and progenitor cell, OLC osteo-lineage cell, BMEC Bone marrow endothelial cell. **c** Heatmap showing the separation of the 4 clusters. **d** Violin plot showing expression of marker genes for each cluster and of SSPC (*Lepr, Cxcl12*), osteolineage cells (*Cd34, Ly6a, Pdgfra, Mfap5, Col1a1*), and bone marrow endothelial cells (*Cdh5*). **e** Proportion of each cluster found in control and *Ncstn* cKO mice. **f** Flow cytometry analysis to confirm the proportional shift seen in scRNAseq of primitive *Lepr*^+^ SSPCs and more mature osteolineage populations PDGFRα^+^ and SCA-1^+^ cells between middle-aged control (*n* = 5) and *Ncstn* cKO (*n* = 6) mice. *P < 0.05, **P < 0.01, ***P < 0.001. Data were represented as mean ± s.e.m

Inhibition of Notch signaling promotes osteogenesis and reduces adipogenesis in vitro.^32^ However, it is unknown whether Notch regulates the detrimental shift from osteogenesis to adipogenesis in the aging skeleton. To investigate this, we disrupted Notch signaling activity in SSPCs in vivo. We focused on *Lepr*^+^ SSPCs since they are the main source of osteoblasts and adipocytes in adulthood^15,35^ and exhibit dysregulated Notch-associated gene expression during aging (Fig. 1d). Nicastrin (*Ncstn*) is a γ-secretase that activates Notch signaling by cleaving all four Notch receptors. Consequently, loss of *Ncstn* abolishes Notch signaling.^36^ We combined *Ncstn*^fl/fl^ mice^36^ with a *Lepr* Cre driver,^37^ and performed scRNAseq of middle-aged *Ncstn*^fl/fl^ (control) and *Lepr*Cre; *Ncstn*^fl/fl^ (*Ncstn* cKO) femurs and tibiae (Fig. 2a), enriching for skeletal and stromal lineages, as in Fig. 1a. Notch pathway genes were significantly downregulated in *Ncstn* cKO SSPCs compared to control cells (Fig. S4a, b) confirming that *Ncstn* deletion impaired Notch signaling. We also validated this by qRT-PCR for the Notch downstream targets *Hey1* and *Hes1*^18,38,39^ (Fig. S4c), UMAP visualization distinguished 25 hematopoietic and endothelial and 1 stromal cluster (Fig. 2a). Analysis of the stromal population in isolation revealed 4 clusters (Fig. 2b) that we defined as *Lepr*^+^ SSPCs (cluster 1, expressing *Lepr and Cxcl12*), early osteolineage progenitor cells (Early OLCs) (cluster 2, expressing *Pdgfra, Ly6a, Cd34*, and *Mfap5)*, differentiated osteolineage cells (Late OLCs) (cluster 0, expressing *Col1a1*), and bone marrow endothelial cells (BMECs) (cluster 3, expressing *Cdh5*) (Fig. 2c, d). The stromal compartment from *Ncstn* cKO mice had a reduced proportion of SSPCs and an increased proportion of early and late osteoprogenitors compared to controls (Fig. 2e). We confirmed these data by flow cytometry, observing a decrease in the frequency of LEPR^+^ SSPCs (Fig. 2f) and an increase in PDGFRα^+^ and SCA-1^+^ osteoprogenitor frequency in *Ncstn* cKO mice (Fig. 2f, Fig. S5).

**Fig. 3:**
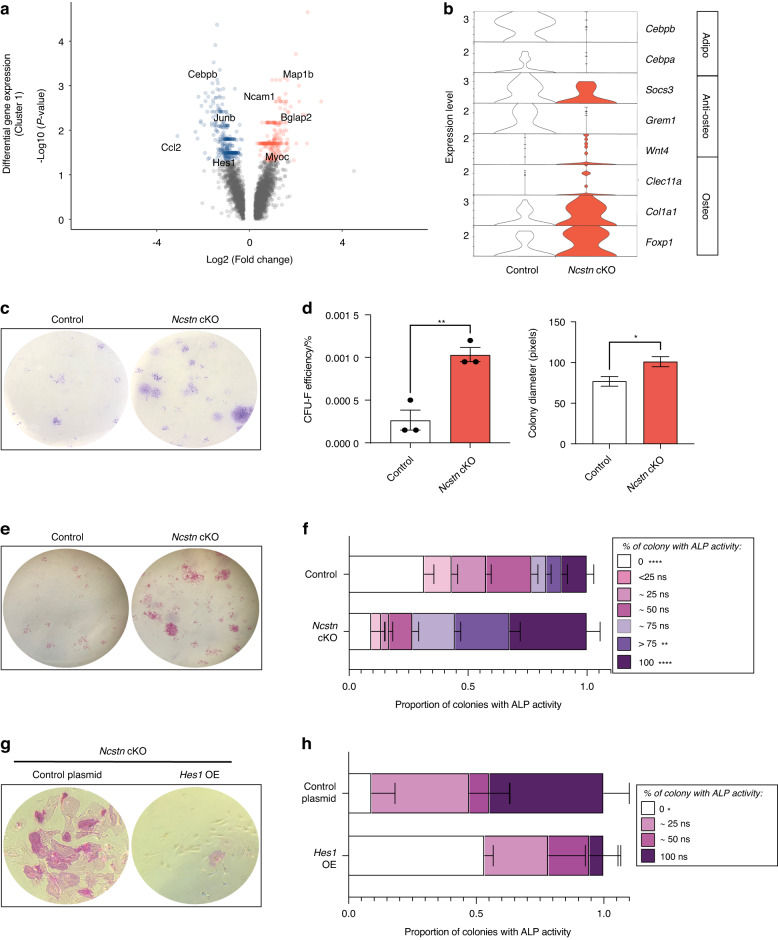
Loss of Notch signaling during aging increases the number of osteoprogenitors. **a** Volcano plot showing differential gene expression between *Ncstn* cKO and control SSPC cluster. Red genes are upregulated in *Ncstn* cKO SSPCs and blue genes are downregulated in *Ncstn* cKO SSPCs compared to control (average log2FC > 0, *P* value < 0.05). **b** Violin plot showing expression of adipogenic, anti-osteogenic, and osteogenic genes between control and *Ncstn* cKO SSPCs. **c** Colony-forming unit fibroblast assay (CFU-F) of control and *Ncstn* cKO middle-aged bone marrow showing representative colony staining with crystal violet. **d** Quantification of CFU-F efficiency (number of cells plated/number of colonies formed) and colony diameter (in pixels)(*n* = 3). **e** Colony-forming unit fibroblast assay (CFU-F) of control and *Ncstn* cKO middle-aged bone marrow showing representative colony staining for alkaline phosphatase (ALP) enzymatic activity, an early osteogenic differentiation marker. **f** Quantification of CFU-Fs showing specified percentages of ALP^+^ cells per colonies (*n* = 3). **g** Colony-forming unit fibroblast assay (CFU-F) of *Ncstn* cKO middle-aged bone marrow showing representative colony staining with ALP with a control plasmid or *Hes1* over-expression plasmid. High-magnification image of a representative colony. **h** Quantification of CFU-Fs showing specified percentages of ALP^+^ cells per colonies (*n* = 3). *P < 0.05, **P < 0.01, ****P < 0.000 1. Data were represented as mean ± s.e.m

To understand the basis of this change, we focused on the Notch signaling deficient SSPC population. We identified 262 genes that were upregulated and 313 that were downregulated in *Ncstn* cKO vs. control *Lepr*^+^ SSPCs (cluster 1, *P* < 0.05) (Fig. 3a, Table S2). Upregulated genes included the osteogenic markers *Bglap2*, *Myoc*,^40^ *Ncam1*,^41^ *Col1a1*, *Wnt4a*,^42^ *Clec11a*,^43,44^ *Foxp1*^45^ (Fig. 3a, b). Conversely, downregulated genes included adipogenic-associated factors, such as *Cebpa, Cebpb*,^46^ *Junb*,^47^ *Ccl2*,^46–49^ and inhibitors of osteogenesis, *Socs3*^50^ and *Grem1*^5^ (Fig. 3a, b). Thus, reducing Notch signaling activity in SSPCs results in elevated osteolineage gene expression, which may transcriptionally “prime” cells for osteogenic differentiation. To examine progenitor number, self-renewal, and osteo-priming at a functional level, we performed colony-forming unit (CFU-F) assays, whereby each stem and progenitor cell gives rise to an individual colony. Bone marrow cells from *Ncstn* cKO mice gave rise to more and larger CFU-Fs than controls (Fig. 3c, d), signifying a greater number of progenitors with increased self-renewal capacity. Notably, CFU-Fs from *Ncstn* cKO mice also displayed elevated alkaline phosphatase (ALP) enzymatic activity, one of the earliest osteogenic markers (Fig. 3e, f), indicating that progenitors are functionally as well as transcriptionally more osteo-primed than those from control mice. The proportion of colonies exhibiting ALP activity was significantly reduced upon overexpression of *Hes1* (Fig. 3g, h), demonstrating that *Ncstn* cKO SSPC osteo-priming was a direct effect of decreased Notch signaling.

Since aging is typically associated with a decrease in osteo-primed and an increase in adipo-primed SSPCs,^7,26^ we then asked whether the stromal compartment of *Ncstn* cKO middle-aged mice more closely resembled that of young rather than middle-aged control mice. To do so, we integrated our young and middle-aged control, and middle-aged *Ncstn* cKO scRNAseq datasets, isolated the stromal population and performed sub-clustering (Fig. S6a). We identified several clusters that expressed periosteal genes, a cluster with an SSPC transcriptional signature, and a cluster expressing bone marrow osteo-chondro lineage markers including chondrocyte, early OLC, and late OLC markers (Fig. S6b–f). Notably, aging in control mice was associated with a decline in bone marrow OLCs that was averted in aging cKO mice (Fig. S6a, g). In contrast, the periosteal clusters were greatly diminished in both middle-aged control and cKO mice (Fig. S6a). The specific rescue of bone marrow OLCs in aging *Lepr*Cre; *Ncstn*^fl/fl^ (cKO) mice is consistent with the fact that *Lepr*^+^ SSPCs predominantly give rise to trabecular bone, not cells within the periosteum.^15^ Together, these data demonstrate that moderating Notch signaling in *Lepr*^+^ SSPCs prevents the loss of osteo-priming and reduction in bone marrow osteoprogenitors with age.

### *Ncstn* cKO mice exhibit increased bone mass and reduced bone marrow adiposity

**Fig. 4:**
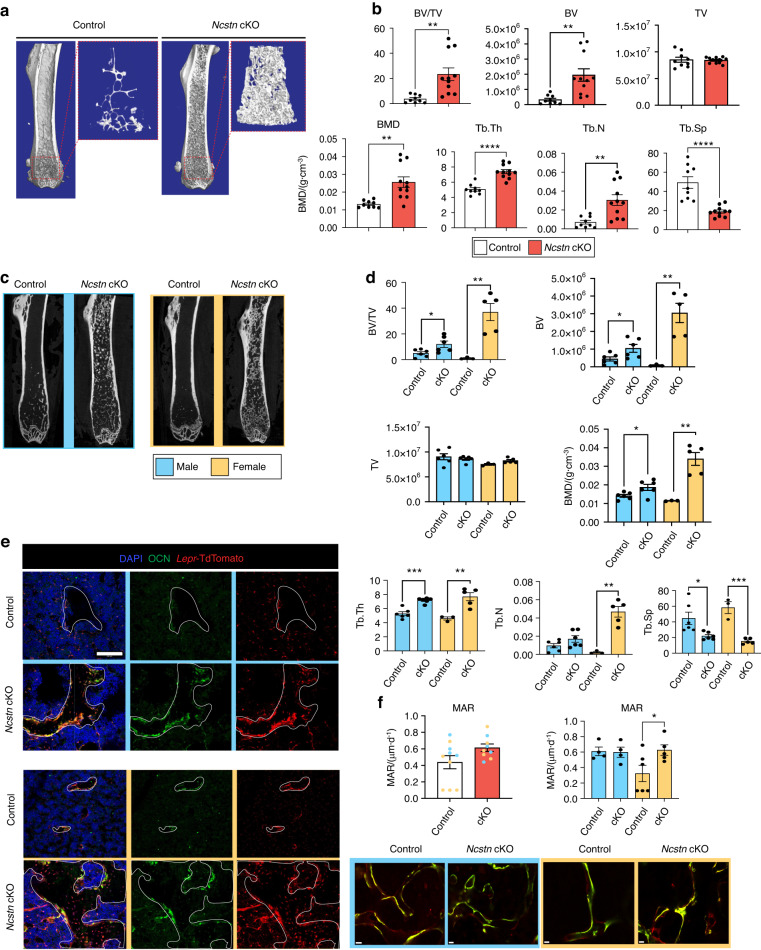
Loss of Notch signaling causes an age and sex-dependent increase in osteogenesis. **a** 3D-rendered coronal microCT cross-section of *Ncstn*^fl/fl^ (control) and *Lepr*Cre; *Ncstn*^fl/fl^ (*Ncstn* cKO) middle-aged mouse femurs. Dotted red box shows isolated metaphyseal trabecular bone. **b** Quantification of trabecular bone parameters throughout the marrow cavity. BV/TV bone volume/tissue volume, BMD Bone Mineral Density, Tb.Th trabecular thickness, Tb.N trabecular number, Tb.Sp trabecular spacing. Control (*n* = 9), *Ncstn* cKO (*n* = 8). **c** Coronal microCT images of the femur separated by sex. Blue = male, yellow = female. **d** Quantification of trabecular bone parameters throughout the marrow cavity separated by sex. Control male (*n* = 6), control female (*n* = 3), *Ncstn* cKO male (*n* = 6), *Ncstn* cKO female (*n* = 5). **e** Representative immunofluorescent images from the femoral metaphysis of *Lepr*Cre; TdTomato and *Ncstn* cKO ; TdTomato male and female mice showing the contribution of the *Lepr* lineage to trabecular bone. Trabecular bone is outlined in white and TdTomato^+^ OCN^+^ osteoblast lining the bone and TdTomato^+^ osteocytes which have a spindle appearance within the trabecular bone. Scale bar = 100 um. **f** Mineral apposition rate (MAR) for control and cKO middle-aged mice with representative imaged, scale bar = 30 um. *P < 0.05, **P < 0.01, ***P < 0.001, ****P < 0.000 1. Data were represented as mean ± s.e.m

Next, we examined how these transcriptional and cell population-based changes in *Ncstn* cKO mice affected the skeletal aging phenotype in vivo. While *Ncstn* cKO mice displayed no gross phenotypic differences compared to littermate controls (Fig. S7a, b), microcomputed tomography (microCT) analysis of middle-aged cKO femurs showed a substantial increase in trabecular bone mass throughout the bone marrow cavity compared to controls (Fig. 4a). Specifically, we detected a significant increase in BV/TV, trabecular thickness (Tb.Th) and trabecular number (Tb.N) and a decrease in trabecular spacing (Tb.Sp) (Fig. 4b). No difference was observed in cortical bone parameters (Fig. S7c). The high bone mass phenotype was also observed in other long bones (data not shown) and, in the vertebral column (Fig. S7d), where it correlated with improved mechanical properties of the cancellous bone (Fig. S7e). The increase in trabecular bone persisted in aged 2-year-old mice (Fig. S7h, i). The phenotype was sex- and age-dependent with the increase in bone mass more pronounced in femurs from female mice (Fig. 4c, d), even at a younger age (~12-week-old) (Fig. S7f, g). Female *Ncstn* cKO middle-aged mice also showed an increase in mineral apposition rate compared to middle-aged female control mice (Fig. 4f) Lineage tracing, using a *Ncstn* cKO; tdTomato mouse, established that the trabecular bone within the marrow cavity was derived directly from *Lepr*^+^ SSPCs (Fig. 4e). To determine whether *Ncstn* cKO mice showed a reduction in catabolic bone resorption, that could additionally contribute to the high bone mass phenotype, we quantified the number of tartrate-resistant acid phosphatase staining^+^ (TRAP^+^) osteoclasts and Osteocalcin (OCN)^+^ osteoblasts (Fig. S7j). TRAP and OCN immunofluorescence staining revealed that *Ncstn* cKO bones contained significantly more osteoclasts and osteoblasts than controls (Fig. S7k), ruling out a reduction of bone resorption as a cause for the observed phenotype.

Moreover, since hematopoietic cells are produced within the bone marrow, we investigated whether the increase in trabecular bone throughout the marrow cavity, affected the systemic hematopoietic lineage output. Despite the decreased bone marrow cellularity in *Ncstn* cKO mice (Fig. S7l), the peripheral blood counts did not exhibit any significant change in lineage output (Fig. S7m, n).

**Fig. 5:**
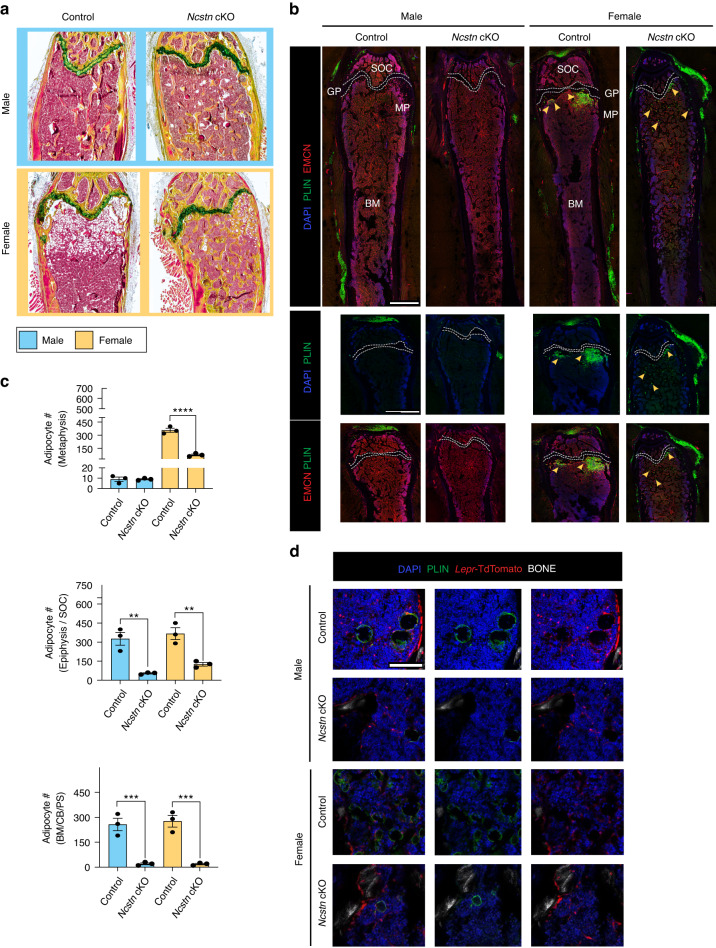
Loss of Notch signaling causes an age and sex-dependent decrease in adipogenesis. **a** Histological sections of control and *Ncstn* cKO middle-aged femurs stained with Movat’s Pentachrome (yellow, bone; red, marrow; blue/green, cartilage). Adipocytes can be seen in the metaphysis as white marrow “ghosts”. Males = blue, females = yellow. **b** Representative immunofluorescent sections of male and female control and *Ncstn* cKO middle-aged femurs stained for perilipin (PLIN)^+^ adipocytes and endomucin (EMNC)^+^ vasculature. Yellow arrowheads point to characteristic adipocyte accumulation in the metaphysis with aging. SOC secondary ossification center, BM bone marrow, MP metaphysis. Top row, scale bar = 1 000 um. Bottom 2 rows, scale bar = 500 um. **c** Quantification of PLIN^+^ adipocytes throughout different compartments of the bone marrow. BM bone marrow, CB cortical bone, PS periosteum. Control male (*n* = 3), control female (*n* = 3), *Ncstn* cKO male (*n* = 3), *Ncstn* cKO female (*n* = 3). **d** Representative immunofluorescent images from the metaphysis of *LeprCre*; TdTomato and *Ncstn* cKO; TdTomato mice. *Lepr* lineage cells in TdTomato can been seen to give rise to PLIN^+^ adipocytes by overlap of TdTomato and PLIN (green). Scale bar = 100 um. *P < 0.05, **P < 0.01, ***P < 0.001. Data were represented as mean ± s.e.m

SSPCs give rise to both osteoblasts and bone marrow adipocytes.^15,35^ Typically, osteogenic and adipogenic differentiation potential is inversely correlated.^21,51^ Consistent with this, in addition to increased bone mineral density, *Ncstn* cKO mice had fewer adipocytes than controls in all anatomic compartments (epiphysis, metaphysis, diaphysis, periosteum) (Fig. 5a–d). In summary, loss of Notch signaling in SSPCs results in an increase in trabecular bone volume and a reduction in bone marrow adiposity in middle-aged mice and thus essentially prevents age-related skeletal degeneration.

### Notch signaling regulates osteo-adipo cell fate decisions in vivo

**Fig. 6:**
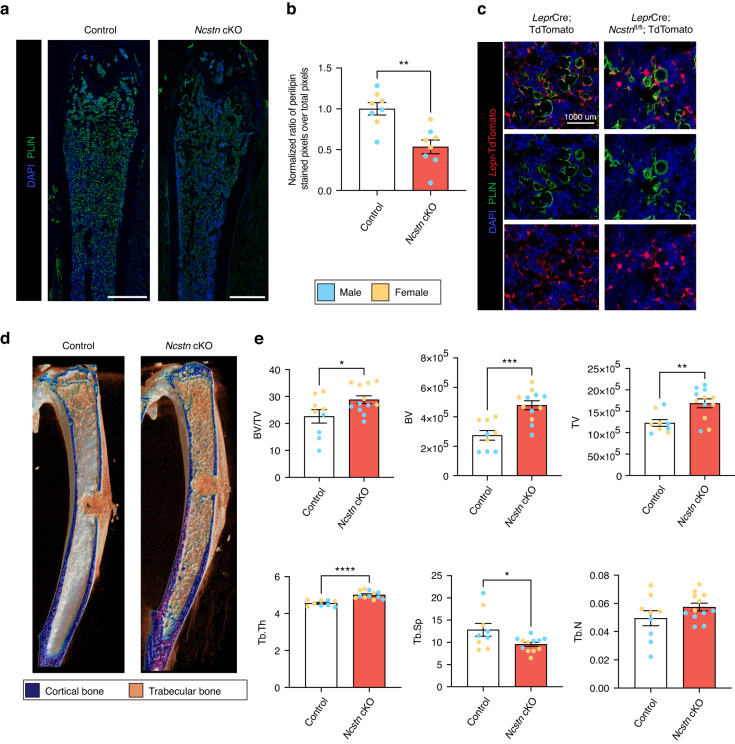
During physical and chemical stress loss of Notch signaling causes decreased adipogenic and increased osteogenic responses. **a** Representative immunofluorescent images from control and *Ncstn* cKO mouse femurs 10 days after 5-FU chemical stress known to induce adipogenesis (PLIN^+^ adipocytes). Scale bar = 1 000 um. **b** Quantification of PLIN^**+**^ staining measured in pixels in the metaphyseal region over the total pixel volume between control (*n* = 8) and *Ncstn* cKO (*n* = 8) mice, (female, yellow; male, blue). **c** Representative immunofluorescent images from *Lepr*Cre; TdTomato and *Ncstn* cKO; TdTomato mouse femurs after 5-FU treatment showing *Lepr* lineage cells marked by TdTomato expression giving rise to PLIN^+^ adipocytes. Scale bar = 1 000 um. **d** Mono-cortical defect injury microCT 3D reconstruction for middle-aged control and *Ncstn* cKO mice, post-operative day (POD) 10. **e** Quantification of trabecular bone parameters for the new woven bone at the injury site formed at POD 10 between control (*n* = 9) and *Ncstn* cKO (*n* = 12) mice. (female, yellow; male, blue). BV/TV bone volume/tissue volume, Tb.Th trabecular thickness, Tb.N trabecular number, Tb.Sp trabecular spacing. *P < 0.05, **P < 0.01, ***P < 0.001. Data were represented as mean ± s.e.m

So far, we showed that Notch signaling in SSPCs controls the homeostatic balance of bone and fat within the skeleton, suggesting that it regulates osteogenic and adipogenic differentiation in vivo. To directly evaluate this, we generated chemical and mechanical skeletal injuries that stimulate adipogenesis and osteogenesis, respectively. To study the adipogenic injury response, we injected *Ncstn* cKO and control mice with 5-Fluorouracil (5-FU), a drug that is commonly used to treat cancer, that triggers fatty degeneration of the bone marrow similar to what is observed during skeletal aging. Control mice treated with 5-FU exhibited adipocytes distributed throughout the bone marrow (Fig. 6a). However, *Ncstn* cKO mice showed a significant reduction in bone marrow adiposity compared to controls (Fig. 6a–c). To assess osteogenic regenerative potential, we created mono-cortical tibial defects^52,53^ in middle-aged mice, which stimulate intramembranous bone formation. At 10 days post-injury, *Ncstn* cKO mice had a higher bone volume/tissue volume (BV/TV) than control mice, indicating enhanced regeneration (Fig. 6d, e). Thus, loss of Notch signaling suppresses adipogenesis and promotes osteogenesis in vivo in both homeostasis (Figs. 4 and 5) and injury (Fig. 6). Moreover, loss of Notch in SSPCs enhances bone repair in elderly individuals.

### Ebf3 is a downstream target of Notch signaling in SSPCs

**Fig. 7:**
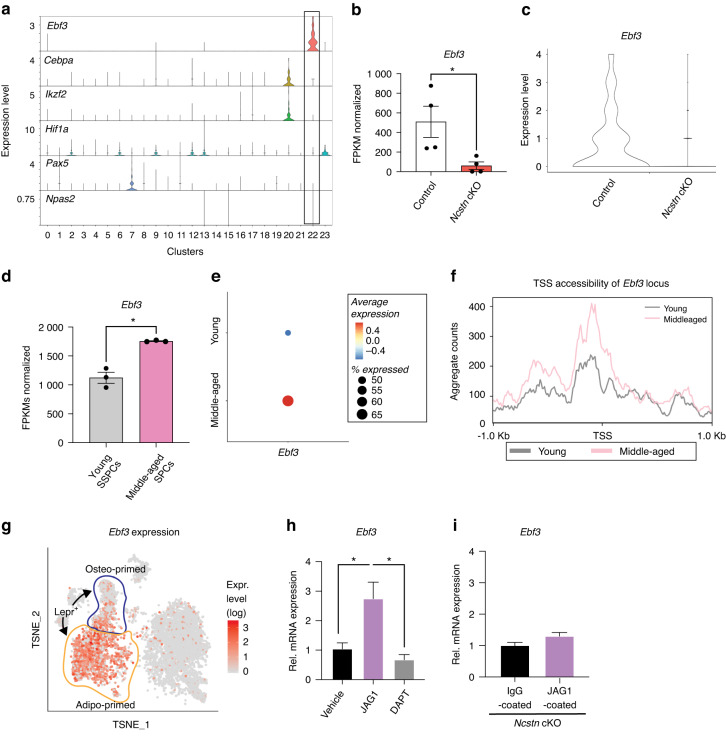
The transcription factor *Ebf3* acts downstream of Notch signaling in SSPCs. **a** Violin plot of compiled scRNAseq data from wildtype young and middle-aged bone/bone marrow showing the expression level of transcription factors identified as differentially expressed in *Ncstn* cKO vs. control SSPC bulk RNAseq. Ebf3 is one of the few transcription factors that is specific for SSPC cluster 22. **b** Normalized FPKMs of *Ebf3* from bulk RNAseq of control and *Ncstn* cKO middle-aged SSPCs. **c** Violin plot showing *Ebf3* expression from scRNAseq of control and *Ncstn* cKO stromal cluster. **d** Bulk RNAseq of young and middle-aged SSPCs showing Normalized FPKMs of *Ebf3*. **e** Dotplot showing expression of *Ebf3* in the SSPC cluster of young and middle-aged mice. **f** SSPCs were subjected to ATAC sequencing. Compared to young SSPCs, chromatin in middle-aged SSPCs was more accessible around the *Ebf3* transcriptional start site (TSS). **g** Expression of *Ebf3* in the *Lepr* lineage cluster from Tikhonova et al. (Nature 2019). Osteo-primed population outlined in blue and adipo-primed population outlined in orange (https://compbio.nyumc.org/niche/). **h** *Ebf3* expression in wildtype SSPCs that were either grown on IgG or JAG1 -coated plates treated with DMSO (1 μL·mL^−1^) (control) or DAPT (10 μmol·L^−1^) (a γ-secretase inhibitor) to inhibit Notch signaling, showing an increase in *Ebf3* with Notch stimulation and decrease in *Ebf3* when Notch signaling is inhibited. **i** qRT-PCR for *Ncstn* cKO SSPCs on IgG or JAG1 -coated plates showing that *Ncstn* cKO SSPCs are not responsive to Notch induced increases in *Ebf3* expression. *P < 0.05, **P < 0.01, ***P < 0.001. Data were represented as mean ± s.e.m

We found that modulating Notch signaling in SSPCs prevented or delayed age-related skeletal degeneration. However, the potential of Notch signaling as a therapeutic target is limited by its known association with cancer^54^ and the widespread expression of pathway components in different cell types within the skeleton (Fig. S8a) increasing the likelihood of off-target effects. Therefore, we aimed to uncover novel effectors downstream of Notch in SSPCs with a restricted expression pattern. To this end, we isolated LEPR^+^CD45^−^CD31^−^TER-119^–^ SSPCs from middle-aged control and *Ncstn* cKO mice and performed bulk RNA sequencing to comprehensively analyze the transcriptional changes in SSPCs. We identified 319 genes that were significantly upregulated and 900 that were downregulated in *Ncstn* cKO SSPCs compared to control SSPCs (*P* < 0.05) (Fig. S8b). Of these, only 28 were transcription factors and hence have the capacity to control the gene regulatory networks that drive cell fate decisions. We analyzed the expression pattern of each transcription factor in our scRNAseq dataset and pinpointed Early B-cell Factor 3 (*Ebf3*) as a promising target due to its relatively SSPC-specific expression (Fig. 7a).^55^ *Ebf3* was downregulated in Notch signaling-deficient SSPCs (Fig. 7b, c) and was also inappropriately upregulated in SSPCs during aging (Fig. 7d, e), coincident with an increase in chromatin accessibility at its transcriptional start site (Fig. 7f). Moreover, previous studies showed that EBF family members promote adipogenic differentiation,^56^ while *Ebf3* inhibits osteogenesis.^55,57,58^ *Ebf3* is more highly expressed in the adipo-primed *Lepr* population (Fig. 7g) and notably, *Lepr*Cre; *Ebf3*^fl/fl^ mice have a near identical increased trabecular bone mass phenotype to *Lepr*Cre; *Ncstn*^fl/fl^ mice,^55^ suggesting that *Ebf3* is downstream of Notch in SSPCs and that it regulates osteogenic and adipogenic cell fate decisions.

To directly investigate a Notch-Ebf3 signaling axis, we isolated and expanded wildtype SSPCs in vitro and seeded them onto tissue culture plates coated with either vehicle control, or the Notch ligand Jagged1 *(*JAG1). SSPCs grown on JAG1*-*coated plates displayed a > 2-fold increase in *Ebf3* expression compared to the control (Fig. 7h). Furthermore, inhibition of Notch signaling using a γ-secretase inhibitor, DAPT (Fig. 7h), or culturing *Ncstn* cKO SSPCs on JAG1*-*coated plates (Fig. 7i) suppressed the increase in *Ebf3*, confirming that *Ebf3* is downstream of Notch.

Together these findings reveal that Notch signaling in skeletal stem cells regulates osteo and adipo cell fate decisions, that pathway components become dysregulated in SSPCs during aging, and that reducing Notch signaling activity is sufficient to prevent age-related skeletal degeneration. Finally, we uncovered a transcriptional effector downstream of Notch in SSPCs that regulates osteogenesis and adipogenesis, which may be investigated in future studies as a potential therapeutic target to prevent or rescue the loss of bone mineral density and fatty degeneration of the bone marrow in elderly individuals.

## Discussion

Here we investigated how cell-intrinsic changes in SSPCs during aging contribute to degeneration of the skeleton. Our scRNAseq dataset of entire hindlimb skeletal elements during aging will also be a valuable resource for future studies addressing non-cell autonomous contributions to this process. In various tissues, such as brain and lung, aging is characterized by the promiscuous re-activation of developmental programs, associated with a loss of repressive heterochromatin.^59^ Notch is a key developmental pathway.^60^ We found that Notch-related genes were aberrantly upregulated in SSPCs during aging, in conjunction with increased chromatin accessibility. Thus, the mechanisms underlying skeletal degeneration may be shared with that observed in other tissues and organs.

*Lepr*^+^ SSPCs are a mix of transcriptionally osteo- and adipo-primed progenitors.^23,24,26,61^ With aging, there is a preferential expansion of the adipo-primed population,^26^ associated with a detrimental bone loss and fatty degeneration of the bone marrow. Here, we showed that Notch signaling is elevated in SSPCs during aging and that blocking Notch signaling enhanced the osteo-priming of *Lepr*^+^ progenitors, consequently increasing osteogenic and decreasing adipogenic differentiation in middle-aged mice. Sex significantly influences skeletal degeneration: female mice show a greater decline in bone mass^62^ and increased metaphyseal BMAT accumulation with age than their male counterparts.^63^ The increased bone mass phenotype in *Ncstn* cKO mice was more prominent in female mice than male mice. Similarly, the conditional knockout of *Ebf1* and *Ebf2* in SSPCs causes a sex-dependent increase in bone mass during aging, with female mice displaying a stronger phenotype.^64^ These findings suggest an interaction between Notch and hormone signaling in aging that should be explored in subsequent studies.

To date, several other genes have been implicated in the age-associated osteo to adipo fate switch. *Lepr* signaling promotes adipogenesis and reduces osteogenesis through JAK2/STAT3.^49^ We observed that loss of Notch signaling in SSPCs decreased *Lepr* expression along with *Socs3*, a downstream target of STAT3 and negative regulator of osteogenesis,^50^ linking Notch to the *Lepr/Jak2/Stat3* axis in aging. In addition, *Foxp1* decreases with age in SSPCs and depletion of *Foxp1* leads to a premature aging phenotype with increased marrow adiposity and decreased bone mass.^65^ *Foxp*1-depleted SSPCs exhibit elevated expression of the Notch-associated transcription factors *Hey1* and *Heyl*, suggesting that *Foxp*1 stimulates osteogenesis and blocks adipogenesis by repressing Notch signaling. We found that *Foxp1* was upregulated in *Ncstn* cKO SSPCs supporting this hypothesis and suggesting that Notch signaling is at the core of the adipo vs. osteo fate decision in aging.

Notch plays a key role in skeletal homeostasis through the regulation of osteogenesis. Conditional deletion of Notch in skeletal progenitors using *Prx1*Cre^66^ increases osteoblast differentiation resulting in a high mass bone phenotype in young mice that culminates in an osteoporotic phenotype and impaired regeneration with age as the progenitor pool is gradually depleted.^18,19,39,67^ In these studies, multiple components of the Notch pathway were conditionally knocked out including: *Presenilin 1* and *2*,^18^ *Notch 1* and *2*,^18^ *Rbpjk*^68^ and *Hey1* and *Heyl*.^39^ While it is unclear why *Ncstn* cKO mice do not develop an osteoporotic phenotype with aging, this may be due to (1) the cell type targeted, as Notch plays context-dependent roles,^20^ (2) canonical vs. non-canonical Notch signaling, and (3) Notch’s role in development vs. adulthood. Specifically, *Prx1*Cre,^66^ is expressed in SSPCs during both development and adulthood, and *Prx1*Cre;*Rbpj*^fl/fl^ mice exhibit developmental abnormalities,^68^ confounding the study of aging phenotypes. Here, our use of the adult SSPC driver, *Lepr*Cre,^15^ enabled us to specifically study the role of Notch signaling in skeletal aging.

The Notch pathway is not an ideal therapeutic target as it has fundamental roles in a wide variety of cell types and is associated with cancer.^69^ However, the downstream mechanisms by which Notch regulates SSPC function remain elusive. Therefore, we aimed to identify SSPC-specific effectors of Notch signaling. The transcription factor *Ebf3* has previously been linked to the inhibition of osteogenesis,^55^ but its upstream regulators were unknown. We defined the transcription factor *Ebf3* as a downstream target of Notch signaling. We believe *Ebf3* to be a good candidate for manipulation because *Lepr*Cre; *Ebf3*^fl/fl^ mice show an increase in trabecular bone mass with aging,^55^ nearly identical to the phenotype in *Lepr*Cre; *Ncstn*^fl/fl^ mice. Crucially, the expression of *Ebf3* in the skeleton is relatively restricted to SSPCs, opening the possibility of developing bone-targeted^70^ EBF3 small molecule inhibitors to modulate SSPC differentiation during aging.

In summary, our study demonstrates that inhibition of Notch signaling in *Lepr*^+^ SSPCs prevents degeneration of the skeleton in middle-aged mice and provides mechanistic insights into the regulation of osteo vs. adipo fate decisions by a Notch-Ebf3 signaling axis. Since Notch signaling is also elevated in the human skeleton during aging,^27^ the molecular mechanisms underlying skeletal degeneration are likely conserved across species. We pinpointed the transcription factor *Ebf3* as a novel downstream effector of Notch signaling that increases with age, highlighting it as a promising therapeutic target to prevent or rescue the age-associated loss of skeletal integrity in elderly individuals.

## Materials and methods

### Animals

Young (~12-week-old) and middle-aged (~52-week-old) C57BL/6 mice (Jax no. 000664), B6.Cg-*Gt(ROSA)26Sor*^*tm14(CAG*−*tdTomato)Hze*^/J (tdTomato, Jax no. 007914), and *B6.129-Leprtm2(cre)Rck/J* (LEPR–Cre, Jax no. 008320) were purchased from Jackson Laboratory (Bar Harbor, ME). Ncstn^fl/fl^^36^ were received from Dr. Aifantis, NYU Robert I. Grossman School of Medicine. Mice were maintained on a 12-h light/dark cycle with food and water provided ad libitum. All animal procedures were performed in accordance to the guidelines of NYU Robert I. Grossman School of Medicine Institutional Animal Care and Use Committee (IACUC).

### Bone and bone marrow cell isolation

Cells were isolated from the tibia and femur and crushed once, using mortar and pestle, then cut into small pieces with scissors. The tissue was subjected to three rounds of enzymatic digestion with 0.2% collagenase at 37 °C under agitation for 30 min each. Cells were filtered through a 70 μm strainer and centrifuged at 1 500 r·min^−1^ for 5 min at 4 °C. Red blood cells were lysed using NH_4_Cl (StemCell Technologies, Vancouver, Canada) for 10 min on ice, washed with staining media (HBSS (Thermo Fisher Scientific, Waltham, MA, USA) containing 2% fetal bovine serum (Life Technologies: 10437-028), 1% HEPES (10 mmol·L^−1^) (Thermo Fisher Scientific) and 1% penicillin-streptomycin (Thermo Fisher Scientific) and resuspended in HBSS for subsequent analyses.

### Flow cytometry

Cells were resuspended in HBSS (Life Technologies: 439 14170161), supplemented with 2% FBS, 1% Penicillin/Streptomycin (Life Technologies: 15140122), and 1% HEPES (Life Technologies: 15630080) (complete HBSS) then stained with appropriate antibodies (Table 1) for 30–45 min in the dark. Cells were washed with 1 mL of complete HBSS solution then centrifuged at 1 500 r·min^−1^ for 5 min. Final cell resuspensions were performed with HBSS for flow cytometry. Cells were sorted on a Sony 450 Biotechnology SY3200TM cell sorter into a 50%/50% solution of complete HBSS and Fetal Bovine Serum or analyzed on a Bio-Rad ZE5 Analyzer. OneComp eBeads (eBioscience 01-1111-41) were used to set initial compensation. Fluorescence minus one (FMO) controls were used for additional compensation and background levels of each stain. Doublets were excluded and gates were determined by internal FMO controls.

**Table 1:** Antibodies for flow cytometry

Antibody	Company	Dilution
CD45-PE	Miltenyi Biotec	1:200
CD31-PE	Miltenyi Biotec	1:200
TER-119-APC	Invitrogen	1:200
TER-119-PE	Miltenyi Biotec	1:200
LY6A (SCA-1)-FITC	Invitrogen	1:200
CD51-BV421	BD Biosciences	1:200
CD140a (PDGFRα)-PE-Cy7	Invitrogen	1:200
CD202b (TIE2)- PE	Invitrogen	1:300
Leptin R Biotinylated Antibody	RND SYSTEMS	1:200
Streptavidin-APC	BioLegend	1:200
DAPI	Thermo Fisher Scientific	1:1 000

### Monocortical tibial defects

All procedures followed protocols approved by the NYU Robert I. Grossman School of Medicine Committee on Animal Research. During surgery, mice were anesthetized with 2% Isoflurane inhalation. A 4 mm incision was made over the proximal anteromedial tibia, then a muscle flap was created over the tibial surface to expose the surface of the bone with care to not disturb the periosteal layer. A 1.0 mm hole was drilled through the anterior cortex with a high-speed dental drill (10 000 r·min^−1^). Incisions were closed with 5–0 Vicryl sutures. Mice were given subcutaneous injections of buprenorphine for analgesia before and after the surgery and were allowed to ambulate freely. Mice were euthanized at indicated days after surgery.

### 5-FU treatment for marrow adipogenic induction

To induce adipogenesis through myeloablation by 5-FU, mice were injected intraperitoneally once with 150 mg·kg^−1^ 5-FU (Sigma-Aldrich, F6627-5G) and euthanized 10 days post-injection.

### MicroCT analyses

Tibias were scanned using a high-resolution SkyScan microCT system (SkyScan 1172, Bruker, Billerica, MA). Images were acquired at 9 μm isotropic resolution using a 10MP digital detector, 10 W energy (100 kV and 100 A), and a 0.5 mm aluminum filter with a 9.7 μm image voxel size. A fixed global threshold method was used based on the manufacturer’s recommendations and preliminary studies showed that mineral variation between groups was not high enough to warrant adaptive thresholding. The samples were oriented, and the volume of interest (VOI) defined with the CTAn software (Bruker). The VOI was contoured manually to capture the entire bone or callus region. The parameters selected to show variations between groups were total bone volume (BV), total tissue volume (TV), respective mineralized volume fraction (BV/TV), trabecular number (Tb.N), trabecular thickness (Tb.Th) and trabecular spacing (Tb.Sp) following the guidelines described by Bouxsein et al.^71^

### Histology

Femurs analyzed using immunofluorescence were dissected and fixed in 4% paraformaldehyde (PFA) for 72 h and washed three times with PBS. After fixation and wash, femurs were embedded in 30% sucrose overnight at 4 °C and at −80 °C until the day before sectioning. The day before sectioning, samples were embedded in OCT and cryosectioned according to Kawamoto’s tape method.^72^

For immunostaining, tape sections were covered in a blocking solution (10% normal goat serum (Sigma), 0.8% Triton X-100) for 45 min at room temperature. Samples were then incubated with primary antibodies overnight 4 °C. Primary antibodies against mouse were used: Endomucin (Santa Cruz, V.7C7, 1:100), Perilipin (Sigma, 1:700), Osteocalcin (Takara Bio, 1:150), Tartrate resistant acid phosphatase (Abcam, 1:100). Slides were then washed and stained in secondary antibodies for 40 min at room temperature in the dark. Secondary antibodies used were Alexa Fluor™ 488 Donkey anti-Rabbit IgG (H + L) (Invitrogen. 1:400), DyLight™650 Donkey anti-Rat IgG (H + L) (Thermo Fisher, 1:400), Alexa Fluor™ 647 Goat anti-Mouse IgG (H + L) (Thermo Fisher, 1:400). Sections were washed and counterstained with DAPI (1:1 000, ThermoFisher). Sections were washed and mounted in Prolong Gold (Life Technologies) anti-fade solution for imaging. Images were photographed using a Zeiss LSM700 laser scanning confocal microscope and Leica Stellaris 8 Falcon laser scanning confocal microscope and analyzed using ImageJ software (National Institutes of Health).

For histomorphometric evaluation, femurs were dissected and fixed in 4% paraformaldehyde (PFA) for 72 h at 4 °C. Bones were decalcified in 19% ethylenediaminetetraaceticacid (EDTA) for 3 weeks at 4 °C. Decalcified samples were embedded into paraffin, and cut into 10-μm-thick sections. Movat’s Pentachrome staining was used to detect osseous and cartilage tissues. Staining sections were photographed using ultra-compact Aperio CS2 system (Leica, Wetzlar, Germany).

### Bone histomorphometry

Middle-aged (12 month) control and cKO mice were injected with 30 mg·kg^−1^ calcein (Sigma C0875-5G) solution. 10d later mice were injected with 50 mg·kg^−1^ alizarin (Thermo Scientific AC155830050) solution. 2 days after alizarin injection mice were euthanized. Femurs were dehydrated in sequential ascending concentrations of ethanol (70%, 80%, 90% and 100%) and embedded undecalcified in methylmethacrylate. One longitudinal section was made using an Isomet Precision Saw (Buehler Ltd., Lake Bluff, IL, USA). One section per femur was analyzed at a magnification of 25× using a Leica Stellaris confocal microscope (Leica Microsystems CMS GmbH, Germany). Static histomorphometric variables on the trabecular surface were obtained using Bioquant software (BIOQUANT Image Analysis System, Corp., Nashville TN), and dynamic bone formation indices were calculated. Static variables were total bone surface (BS, mm), single-label surface (sLS, mm), double-label surface (dLS, mm) and the interlabel width (Ir.L.Wi, μm). Dynamic variables calculated, mineral apposition rate (MAR = Ir.L.Wi/days). When only single labels were present, the mineral apposition rate was estimated as the minimum value observed in that specific experimental group.

### Colony-forming unit assays

2 × 10^6^ cells isolated from the bone marrow of femurs and tibiae as previous described^1^ were seeded in 6- well adherent tissue culture plates using growth media (DMEM containing 10% FBS and 1% penicillin/streptomycin). The next day cells were washed with PBS and growth media was replaced. After 9–10 days CFU-Fs were stained with 1% crystal violet in methanol. CFU-F efficiency was calculated (counted colonies/ cells originally seeded * 100). Colony length was measured by the diameter of the colony in pixels using ImageJ software. ALP staining for osteo-primed progenitors was performed using Alkaline Phosphatase Detection Kit (Sigma Aldrich).

### Hes1 overexpression

CFU-Fs were plated as described above. At the time of seeding cells were also transfected with 1 ug of DNA and 5 uL of Polyethylenimine PEI (1 mg·mL^−1^ PEI) (VWR) in Opti-MEM (Thermo Fisher). Plasmids were both purchases from Vector Builder: a control stuffer plasmid: pRP[Exp]-EGFPCMV > ORF_Stuffer (VB210104-1248han) and hes1 plasmid pRP[Exp]-EGFP-CMV > mHes1[NM_008235.2] (VB210104-1048rgg). Media was changed the next day.

### Isolation and culture of skeletal stem and progenitor cells in vitro

For the in vitro experiments, tibial and femoral bone marrow cells were isolated by dissection. The ends of the bones were cut off to expose the bone marrow and cells were isolated by centrifugation. Cells were re-suspended in growth media (DMEM containing 10% FBS and 1% penicillin-streptomycin) and then plated in 75 ml tissue culture flasks.

To activate Notch signaling in vitro, cells were trypsinized and seeded on pretreated AffiniPure Goat Anti-Human IgG, Fcγ Fragment Specific (Jackson ImmunoResearch 109-005-098) or Jagged-1 (R&D 1277-JG) coated tissue culture plates as described by Kaur et al., and Lee et al.^73,74^

### RNA isolation and quantitative real-time PCR

RNA was isolated from cells using RNeasy Kit (Qiagen, Germantown, MD, USA) according to manufacturer’s instruction. cDNA was synthesized using iScript^TM^ cDNA Synthesis Kit (Bio-Rad, Hercules, CA, USA). Quantitative real-time PCR was carried out using the Applied Biosystems Step One Plus detection system (Thermo Fisher Scientific) and RT2 SYBR Green ROX PCR Master Mix (Qiagen). Results are presented as 2^–ΔΔCt^ values normalized to the expression of 18S. Means and SEMs were calculated in GraphPad Prism 9 software. Primer sequences are listed in Table 2.

**Table 2:** PCR primers

Primer Name	Sequence (5’-3’)
18 S FOR	ACGAGACTCTGGCATGCTAACTAGT
18 S REV	CGCCACTTGTCCCTCTAAGAA
Hes1 FOR	TGCCAGCTGATATAATGGAG
Hes1 REV	CTTTGATGACTTTCTGTGCTC
Hey1 FOR	ACTACAGCTCCTCAGATAGTG
Hey1 REV	AACTCAAGTTTCCATTCTCGTC

All primers were purchased from Integrated DNA Technologies

### Bulk RNA sequencing

Libraries were sequenced on the Illumina NovaSeq 6000 sequencer. Results were demultiplexed and converted to FASTQ format using Illumina bcl2fastq software and sequencing reads were aligned to the mouse genome (build mm10/GRCm38) using the splice-aware STAR aligner [http://www.ncbi.nlm.nih.gov/pubmed/23104886]. The featureCounts program [https://www.ncbi.nlm.nih.gov/pubmed/24227677] was implemented to produce counts for each gene based on how many aligned reads overlap its exons. The counts were normalized and used to test for differential expression using negative binomial generalized linear models implemented by the DESeq2 R package [http://www.ncbi.nlm.nih.gov/pubmed/25516281].

### ATAC sequencing

Transposase-accessible chromatin with sequencing (ATACseq) was performed on 50 Illumnia Hiseq 2 500 run. FASTQ files were generated using bcl2fastq Conversion software (v1.8.4) to convert per-cycle BCL base call files outputted by the sequencing instrument into the FASTQ format. The alignment program, Bowtie2 (v2.3.4.1), was used for mapping reads to the mouse reference genome mm10 and Sambamba (v0.6.7) was used to remove duplicate reads. The algorithm, MACS (in Python v2.7.3), was utilized to call peaks of signal for annotated genomic features. The package NucleoATAC was functioned to call nucleosome positions. The computeMatrix and plotProfile tools in the deepTools suite (v2.3.3) were utilized for generation of signal profile plots. The DiffBind package (Bioconductor v3.3.0) in the R statistical programming environment was used for the differential peak comparisons between young and middle-aged samples.

### Single-cell RNA sequencing

For both young *Ncstn*^fl/fl^ (control), middle-aged *Ncstn*^fl/fl^ (control) and *LeprCre*; *Ncstn*^fl/fl^ (cKO) (*n* = 5 mice per condition pooled), hindlimbs were isolated as described above in bone and bone marrow isolation and then we performed fluorescence-activated cell sorting to isolate hematopoietic and endothelial cells (CD45^+^CD31^+^TER-119^−^) and skeletal and stromal lineages (CD45^−^CD31^−^TER-119^−^). Hematopoietic/endothelial and osteolineage/stromal compartments were then mixed at a 1:1 ratio to allow for enrichment of the rarer osteolineage/stromal cells. Libraries were prepared using the Chromium single cell 3ʹ reagent v2 protocol (10x Genomics) per the manufacturer’s recommendations. Sequencing was performed by the Illumina NovaSeq 6000. Downstream analysis was performed using the Seurat package in R utilizing their integrated pipeline analysis. We filtered out cells with less than 100 genes per cell and with more than 30% mitochondrial content. GSEA analysis was performed utilizing the R program escape 1.4.1.

### Statistical analysis

A priori power analysis to obtain statistical significance (*P* = 0.05, power 80%) resulted in an *n* of 4 for each group after body-size adjustment, expecting a 25% difference between the groups. All cell culture–based assays show biological replicates and were repeated at least three times.

Prism 9 (GraphPad Software, Inc.) was used for statistical computations. A Student’s *t* test was used for all comparisons in which there were two groups; ANOVA analyses followed by the Holms-Sidak correction for post hoc testing was applied for analyses in which there were two or more comparisons being made. Error bars represent SEMs. *P* < 0.05 was statistically significant. An asterisk symbol (*) denotes a *P* value < 0.05, unless denoted otherwise in figure legend.

### Supplementary information

Supplementary Figures

Supplementary Table S1

Supplementary Table S2

## Data Availability

Requests for data and materials should be addressed to P.L. scRNAseq, ATAC-seq, and RNAseq data deposited in GEO (GSE240292).
