# Supplementary material for: Loss of Notch signaling in skeletal stem cells enhances bone formation with aging
Source: Bone Res. 2023 Sep 27;11:50. doi: 10.1038/s41413-023-00283-8 (PMC10522593; doi:10.1038/s41413-023-00283-8)
Supplement: Supplementary file 1 — Supplementary Figures [file 41413_2023_283_MOESM1_ESM.docx]

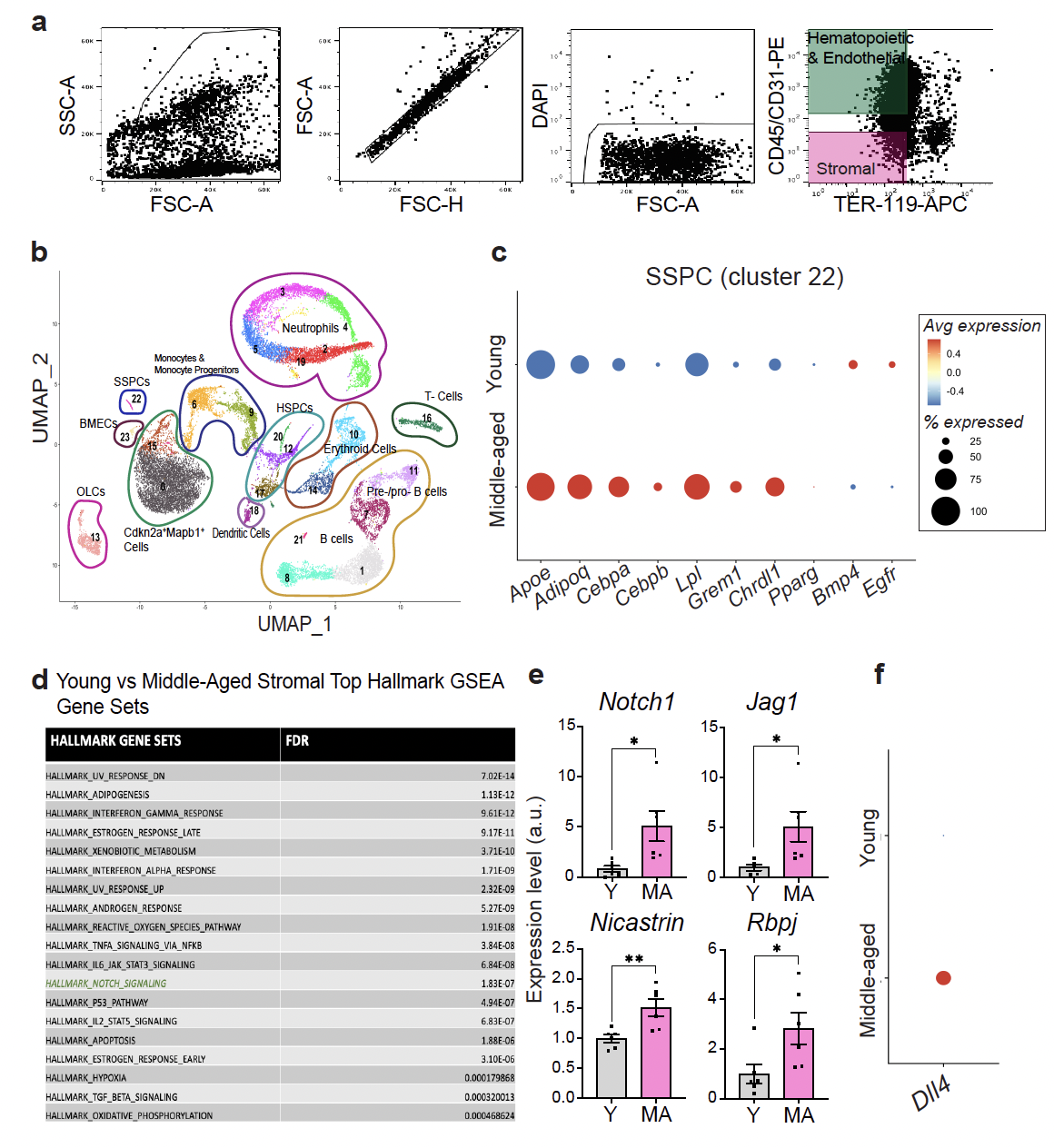


**Supplemental Figure 1. Young and middle-aged bone and bone marrow single-cell RNA sequencing isolation and characterization. (a)** Representative flow cytometry plot for collection of stromal and hematopoietic compartments. **(b)** UMAP for young and middle-aged combined labeled clusters **(c)** Dotplot for the SSPC cluster 22 showing adipogenic (*Apoe, Adipoq, Cebpa, Cebpb, Lpl, Pparg*), anti-osteogenic (*Grem1, Chrdl1*), and osteogenic (*Bmp4, Egfr)* gene expression. **(d)** Stromal young vs middle-aged top 20 significant GSEA Hallmark terms. **(e)** qRT-PCR showing the expression of key Notch signaling pathway components in bone samples from young and middle-aged mice. **(f)** Dotplot for BMEC cluster 23 showing Notch ligand, Dll4 (Delta-4), gene expression.

*p < 0.05, **p < 0.01. Data were represented as mean ± s.e.m.


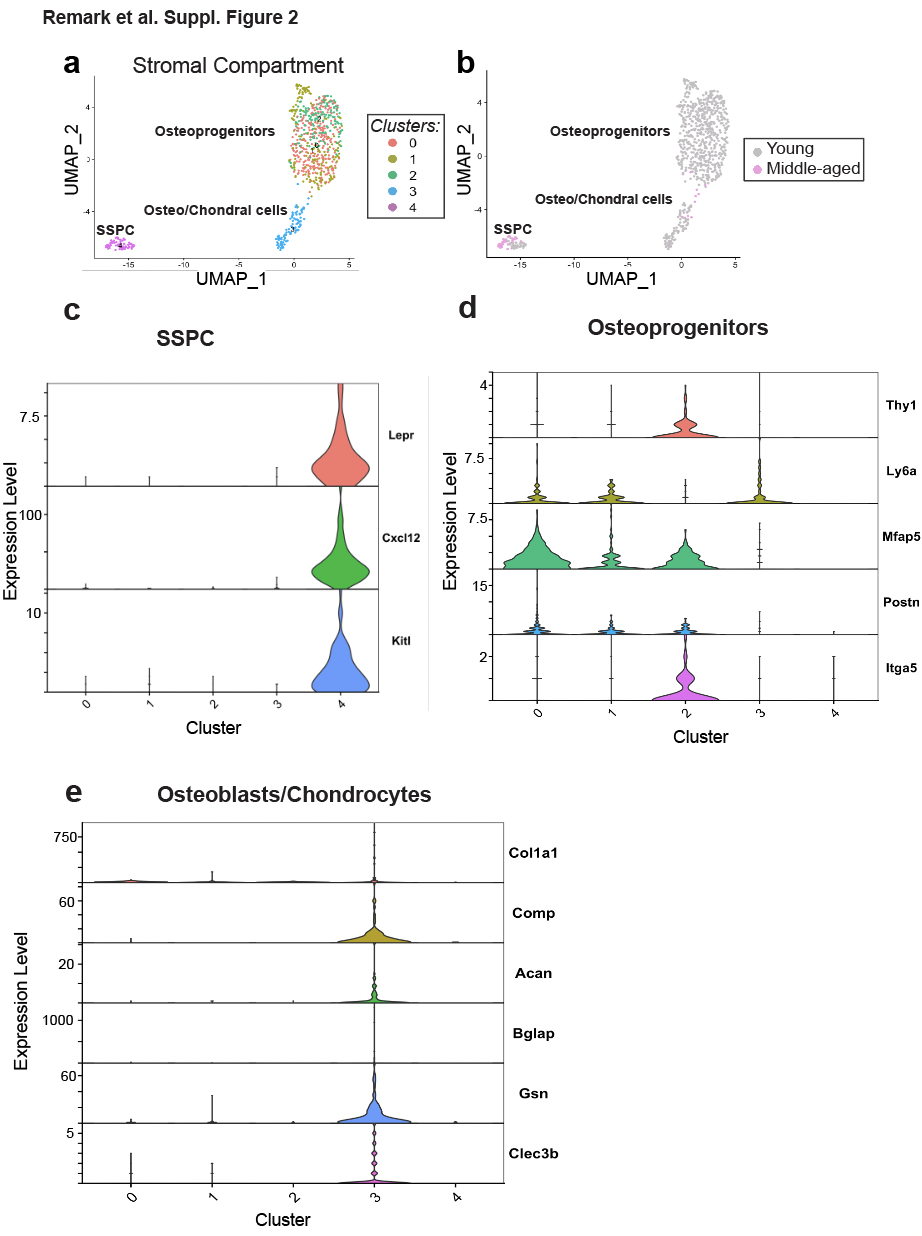


**Supplemental Figure 2. Young and middle-aged bone and bone marrow single-cell RNA**

**sequencing stromal sub clustering. (a)** UMAP showing the sub clustering of the stromal compartment giving 5 sub clusters: SSPCs, osteoprogenitors, and differentiated osteoblasts/chondrocytes. **(b)** UMAP showing the cells from young (gray) and middle-aged mice (pink) and their proportions relative to one another in the integrated analysis. **(c)** Violin plot showing expression of SSPC marker genes found in cluster 4. **(d)** Violin plot showing expression of osteoprogenitor genes found in clusters 0,1,2. **(e)** Violin plot showing expression of differentiated marker genes of osteoblasts and chondrocytes in cluster 3.


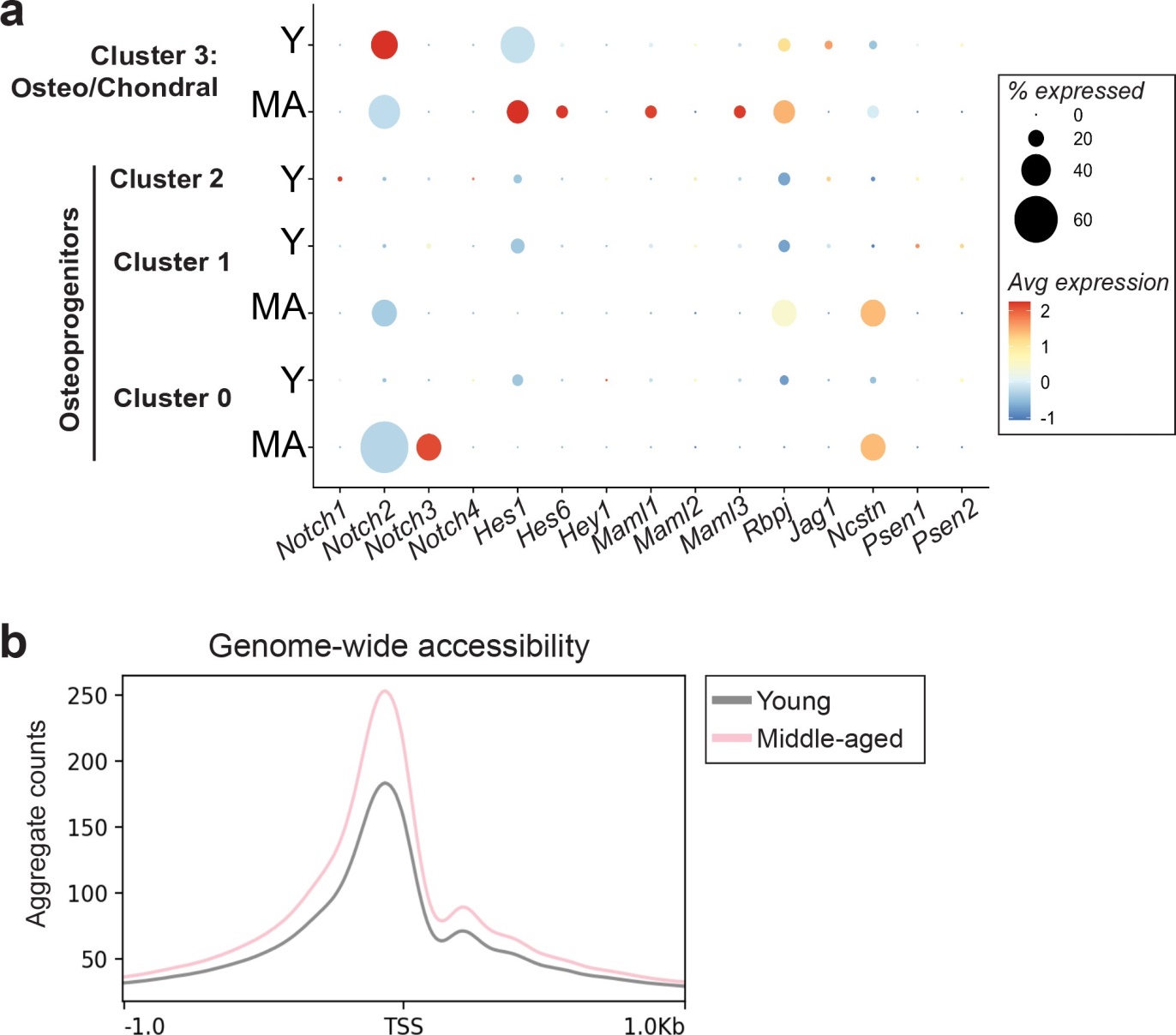


**Supplemental Figure 3. Young and middle-aged bone and bone marrow single-cell RNA sequencing stromal sub clustering Notch gene expression and ATAC sequencing global changes with aging. (a)** UMAP showing the sub clustering of the stromal compartment isolated from young (Y) and middle-aged (MA) mice showing Notch gene expression across clusters. Cluster 2 is only found in young mice. **(b)** ATAC sequencing comparing young to middle-aged SSPCs showing that chromatin in middle-aged SSPCs was more accessible around genomic wide transcriptional start sites (TSS).

#
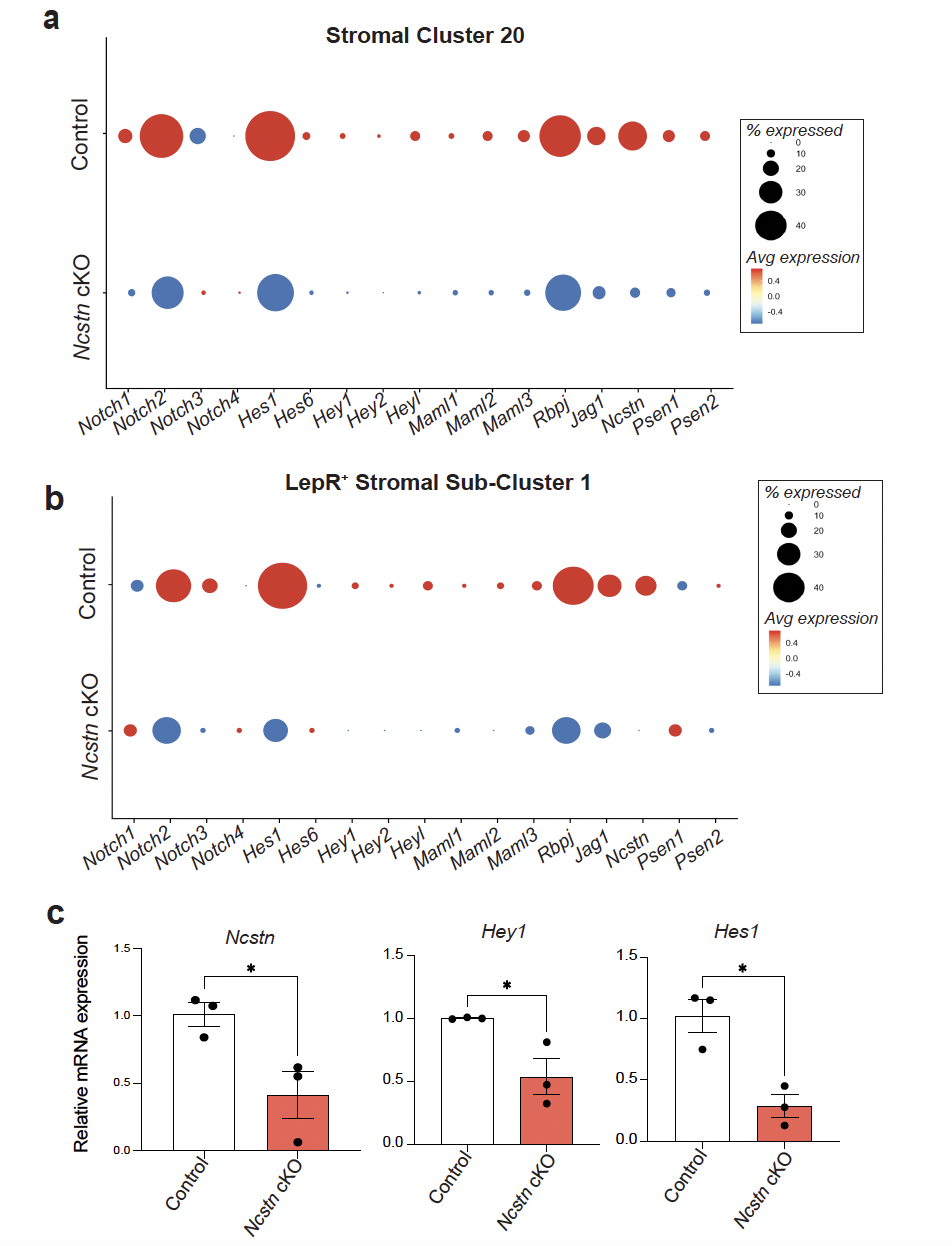
Supplemental Figure 4. *Lepr*Cre; *Ncstn*^fl/fl^ mice exhibit deficits in Notch signaling.

**(a)** Dotplot showing expression of Notch signaling pathway genes in stromal cluster between control and *Ncstn* cKO mice for the entire stromal population (cluster 20, Fig. 2a). **(b)** Dotplot showing expression of Notch signaling pathway genes in stromal subset LepR+ cluster (cluster 1, Fig. 2b) between control and *Ncstn* cKO mice.  **(c)** qPCR for gene expression of *Ncstn* and key Notch target genes: *Hey1* and *Hes1* from control and *Ncstn* cKO middle-aged SSPCs. * p < 0.05. Data
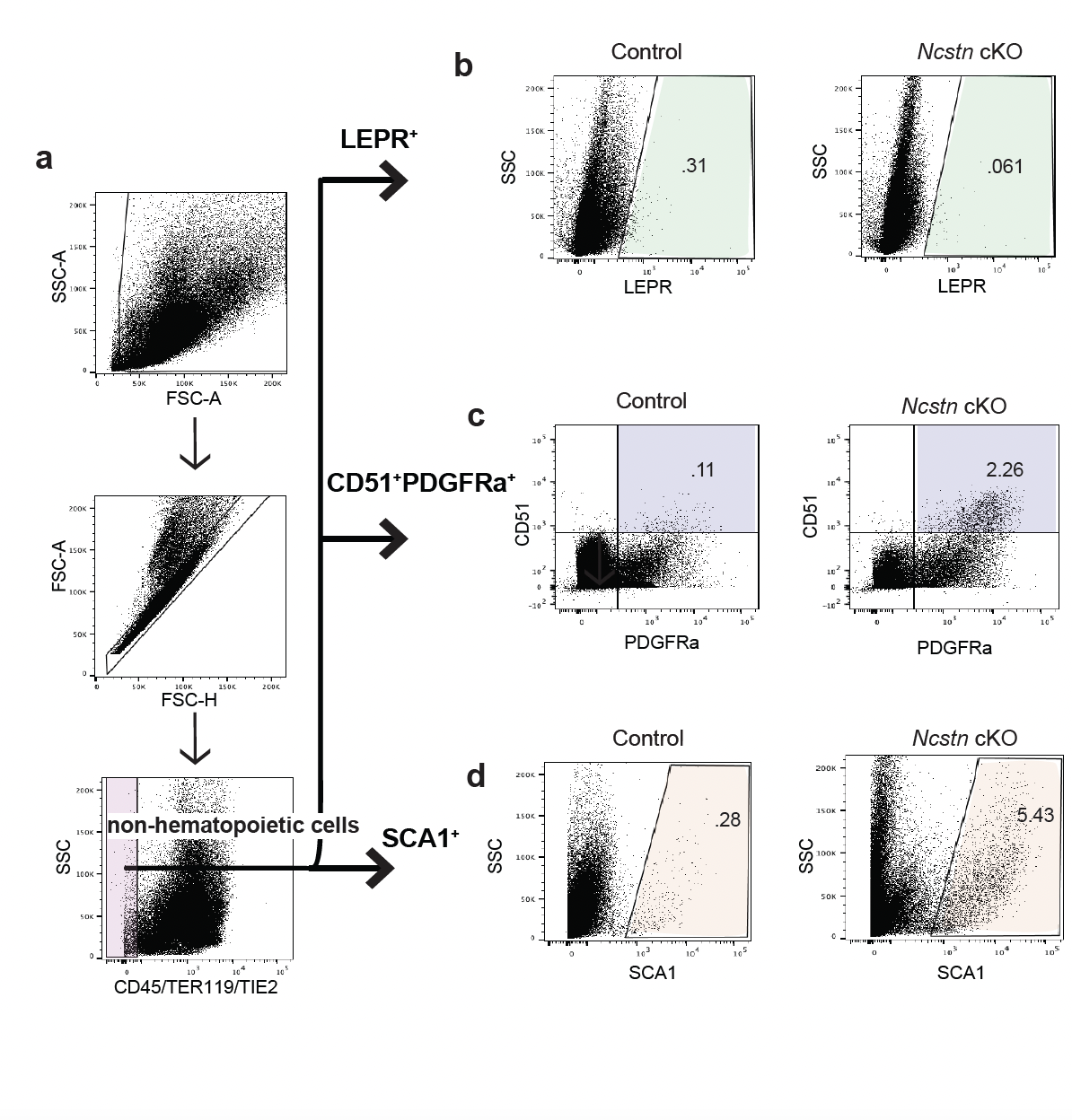
represented as mean ± s.e.m.

**Supplemental Figure 5.** **Flow Cytometry Gating Strategy and Representative Plots for LepR, PdgfraCD51, and Sca-1 Populations.** **(a)** Gating strategy for non-hemopoietic CD45-TER119-TIE2- cells used in subsequent analysis of skeletal stem and progenitor populations outlined. **(b)** Gating strategy for LEPR+ SSPCs and representative plots for control and *Ncstn* cKO frequency of CD45-TER119-TIE2-LEPR+ cells. **(c)** Gating strategy for PDGFRa+CD51+ progenitors and representative plots for control and *Ncstn* cKO frequency of CD45-TER119-TIE2- PDGFRa+CD51+ cells. **(d)** Gating strategy for SCA-1+ progenitors and representative plots for control and *Ncstn* cKO frequency of CD45-TER119-TIE2- SCA-1+ cells.


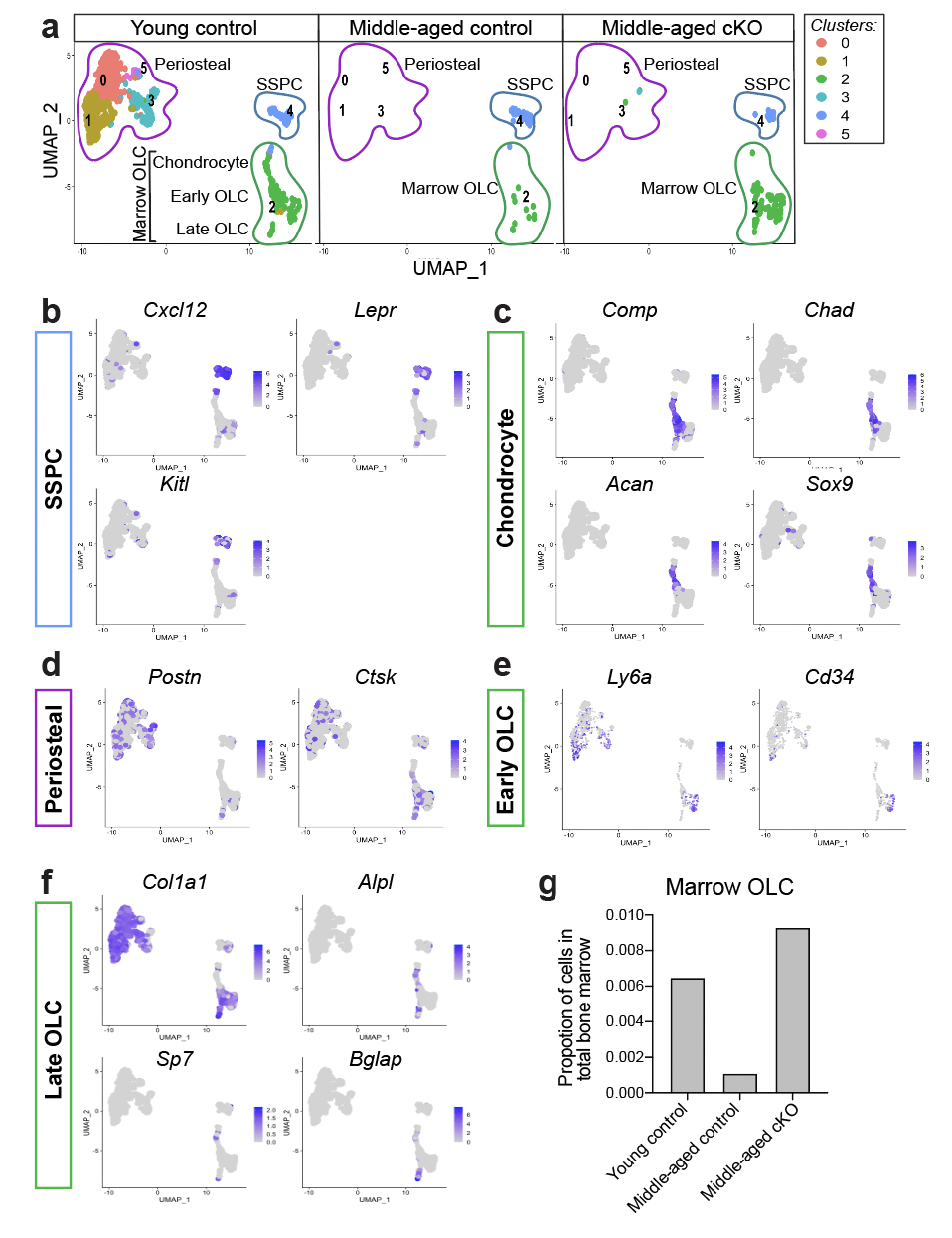


**Supplemental Figure 6. scRNAseq data integration revealing osteo-lineage cell preservation with aging in cKO mice**. **(a)** UMAP shows the sub-clustering of the stromal compartment integrated across young, middle-aged control, and middle-aged cKO mice. Six populations are present- 4 periosteal clusters, 1 SSPC cluster, and 1 bone marrow osteo-lineage cell cluster including chondrocytes, early osteo-lineage progenitor cells, and mature osteolineage cells. **(b)** Featureplot showing expression of SSPC defining genes located to SSPC cluster 4. **(c)** Featureplot showing expression of chondrocyte defining genes expressed in a subset of marrow OLC cluster 2. **(d)** Featureplot showing expression of periosteal defining genes expressed in clusters 0,1,3,5. **(e)** Featureplot showing expression of early osteo-lineage progenitor cell genes expressed in a subset of cluster 2. **(f)** Featureplot showing expression of mature osteo-lineage cell genes expressed in a subset of cluster 2. **(g)** Osteo-lineage cluster size between young, control middle-aged, and middle-aged cKO mice showing size maintenance in the middle-aged cKO.


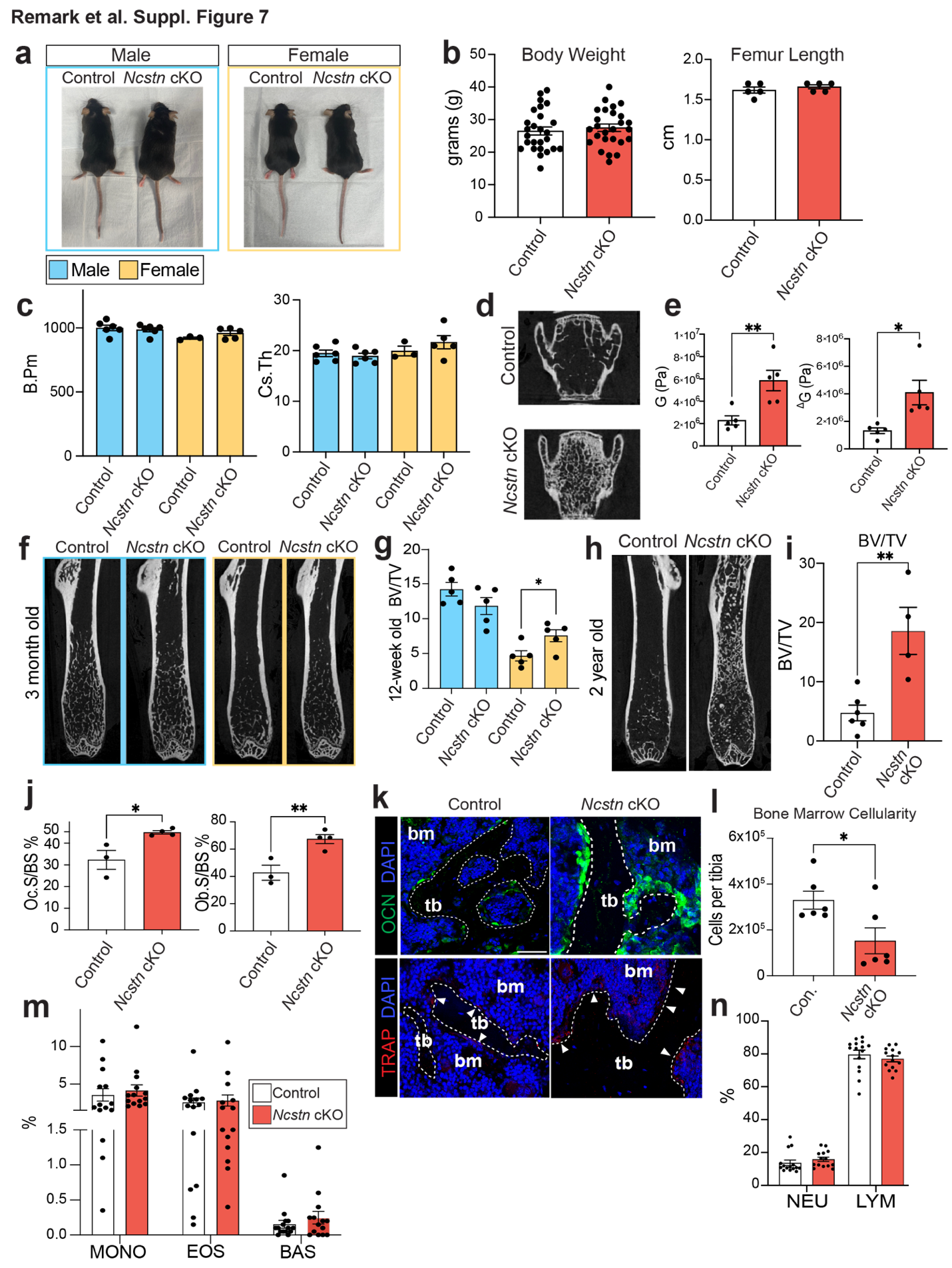


**Supplemental Figure 7. *Lepr*Cre;*Ncstn*^fl/fl^ characterization. (a)** Images of control and *Ncstn*

cKO male and female mice showing no gross phenotypic differences in size and shape. **(b)** control and *Ncstn* cKO body weight (n=26) in grams and femur length (n=5) in centimeters. **(c)** Cortical bone microCT quantification of male and female middle-aged *Ncstn*^fl/fl^ and *Ncstn* cKO mice. B.Pm= bone perimeter, Cs.Th= cross-sectional thickness. **(d)** Representative images of the lumbar spine in middle-aged control and *Ncstn* cKO mice. **(e)** Vertebral mechanical testing for strength of L3 vertebra between middle-aged control and *Ncstn* cKO mice. G and deltaG are given in pascals (Pa). **(f)** Representative coronal images from representative young adult control and *Ncstn* cKO male and female femurs. **(g)** Quantification of trabecular bone volume/tissue volume (BV/TV) for young adult (3 month) control and *Ncstn* cKO male and female femurs (n=5). **(h)** Representative coronal images of 2-year-old femurs showing the phenotype persists with aging. **(i)** Quantification of trabecular bone volume/tissue volume (BV/TV) for 90-week-old control (n=6) and *Nctsn* cKO (n=4) femurs **(j)** Quantification from immunofluorescent sections stained for OCN and TRAP of Ob.S/BS and Oc.S/BS%. Ob.S/BS= osteoblast surface/ total bone surface, Oc.S/BS= osteoclast surface/ total bone surface (n=3). **(k)** Representative immunofluorescent sections of male and female control and *Ncstn* cKO middle-aged femurs from the metaphyseal region where trabecular bone is located. Osteocalcin (OCN), an osteoblast marker, in green and Tartrate- resistant acid phosphatase (TRAP) in red. Trabecular bone is outlined with the dotted white line. White arrowheads point to TRAP+ osteoclasts lining trabecular bone. Tb= trabecular bone, Bm= bone marrow. **(l)** Bone marrow cellularity per tibia in *Ncstn*^fl/fl^ and LepRCre; *Ncstn*^fl/fl^ middle-aged mice (n=6). **(m)** Complete blood count (CBC) analysis for *Ncstn*^fl/fl^ and LepRCre; *Ncstn*^fl/fl^ middle- aged mice. Mono= Monocytes, Eos= Eosinophils, Bas= Basophils (n=14). **(n)** Complete blood count (CBC) analysis for *Ncstn*^fl/fl^ and LepRCre; *Ncstn*^fl/fl^ middle-aged mice. Neu= Neutrophils and Lym= Lymphocytes (n=14). *p < 0.05, **p < 0.01. Data represented as mean ± s.e.m.

#
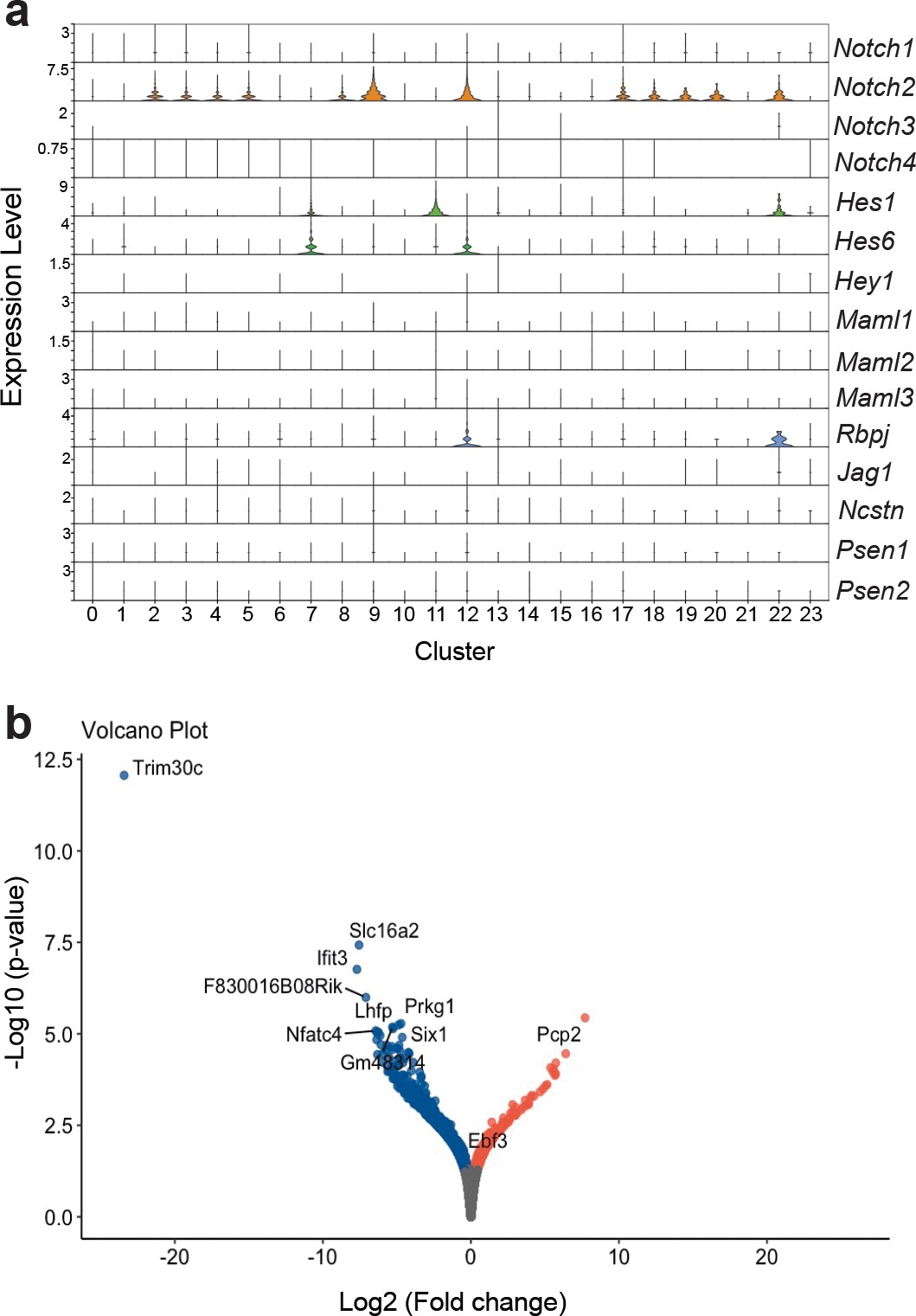


**Supplemental Figure 8. Identifying downstream targets of Notch signaling in SSPCs. (a)** Violin plot from scRNAseq of young and middle-aged bone/bone marrow showing expression of Notch genes across populations. **(b)** Volcano plot from bulk RNAseq of LEPR^+^CD45^-^CD31^-^TER- 119^-^ control and *Ncstn* cKO middle-aged SSPCs (n=4 mice per condition).
