# Supplementary material for: Loss of Notch signaling in skeletal stem cells enhances bone formation with aging
Source: Bone Res. 2023 Sep 27;11:50. doi: 10.1038/s41413-023-00283-8 (PMC10522593; doi:10.1038/s41413-023-00283-8)
Supplement: Supplementary file 2 — Supplementary Table S1 [file 41413_2023_283_MOESM2_ESM.pdf]

Cluster Marker Genes Figure 1B Young and Middle-Aged Mice

	p_val	avg_log2FC	pct.1	pct.2	p_val_adj	cluster
Wfdc2	0	3.24877005	0.669	0.022	0	0
Krt8	0	3.17557636	0.733	0.029	0	0
Tm4sf1	0	3.08258317	0.713	0.037	0	0
Lgals1	0	2.91917486	0.951	0.259	0	0
Krt18	0	2.90328754	0.613	0.025	0	0
Rpl23a	0	2.63276082	0.959	0.396	0	0
Hsp90ab1	0	2.48590695	0.97	0.478	0	0
Vim	0	2.42769708	0.919	0.377	0	0
Cdkn2a	0	2.41453343	0.469	0.016	0	0
Ccnd1	0	2.41249615	0.516	0.032	0	0
Ly6a	0	2.40937186	0.493	0.056	0	0
Lmna	0	2.33216355	0.572	0.062	0	0
Mt2	0	2.31717803	0.463	0.036	0	0
Npm1	0	2.30193871	0.914	0.353	0	0
Tpm1	0	2.28141601	0.596	0.075	0	0
Nedd4	0	2.2455245	0.623	0.117	0	0
Clu	0	2.22782626	0.554	0.036	0	0
Serpinb6a	0	2.2100527	0.473	0.047	0	0
Ahnak	0	2.18091924	0.744	0.153	0	0
Hspb1	0	2.17607942	0.322	0.02	0	0
Plec	0	2.17055762	0.535	0.093	0	0
2200002D01	0	2.15301347	0.414	0.022	0	0
Mt1	0	2.14583928	0.641	0.135	0	0
Spp1	0	2.13666	0.808	0.123	0	0
Fbln2	0	2.12846195	0.563	0.047	0	0
Anxa5	0	2.0818687	0.604	0.158	0	0
Rpl12	0	2.07747793	0.94	0.432	0	0
Rps12	0	2.06425355	0.986	0.611	0	0
S100a10	0	2.05843034	0.833	0.292	0	0
Hmga2	0	2.03630349	0.38	0.015	0	0
Rps21	0	2.029261	0.996	0.74	0	0
Scd2	0	2.01507749	0.434	0.061	0	0
Myof	0	1.95930524	0.406	0.029	0	0
Rps15	0	1.95230849	0.917	0.434	0	0
Rplp1	0	1.93577438	0.995	0.633	0	0
Rpl5	0	1.9231754	0.852	0.356	0	0
S100a4	0	1.87783544	0.629	0.119	0	0
Hsp90aa1	0	1.87567695	0.726	0.276	0	0
S100a6	0	1.86396283	0.992	0.522	0	0
Cd2ap	0	1.85181961	0.435	0.069	0	0
Map1b	0	1.83578972	0.346	0.018	0	0

Rpl13a	0	1.83244251	0.929	0.476	0	0
Cct6a	0	1.82956892	0.459	0.102	0	0
Eef1g	0	1.82033252	0.629	0.197	0	0
Ppp1r14b	0	1.81597767	0.588	0.174	0	0
Rps8	0	1.81480524	0.994	0.682	0	0
Rack1	0	1.80871687	0.905	0.436	0	0
Gnas	0	1.79855068	0.768	0.358	0	0
Hspd1	0	1.79433735	0.524	0.141	0	0
Eef1a1	0	1.7713754	0.992	0.656	0	0
Tnfrsf12a	0	1.77091725	0.323	0.02	0	0
Pmepa1	0	1.76993222	0.435	0.05	0	0
Emp1	0	1.74895566	0.378	0.034	0	0
Slc25a4	0	1.7448735	0.455	0.101	0	0
Mif	0	1.71355775	0.692	0.248	0	0
Calu	0	1.69497972	0.391	0.063	0	0
Rpl17	0	1.6944399	0.95	0.597	0	0
Itgb1	0	1.67166582	0.526	0.147	0	0
Cavin1	0	1.66255491	0.301	0.021	0	0
Onecut2	0	1.6605428	0.263	0.016	0	0
Hdgf	0	1.64345248	0.507	0.175	0	0
Fkbp1a	0	1.63990742	0.468	0.135	0	0
Ybx3	0	1.6365571	0.432	0.123	0	0
Cavin2	0	1.63483542	0.256	0.011	0	0
Cystm1	0	1.62684829	0.282	0.024	0	0
Ncl	0	1.62331731	0.723	0.335	0	0
Rpl8	0	1.62221524	0.982	0.668	0	0
Pcolce	0	1.60570509	0.292	0.021	0	0
Gm42418	0	1.60017421	0.999	0.925	0	0
Hspa8	0	1.58950913	0.845	0.473	0	0
Rpl6	0	1.58385896	0.982	0.699	0	0
Itga6	0	1.56861295	0.306	0.033	0	0
Col18a1	0	1.56327361	0.265	0.013	0	0
Pkm	0	1.55311758	0.676	0.365	0	0
Anxa2	0	1.53764547	0.685	0.351	0	0
Set	0	1.53645887	0.639	0.274	0	0
Tpi1	0	1.53575296	0.412	0.145	0	0
Eif3a	0	1.53213018	0.514	0.189	0	0
Rps14	0	1.5289531	0.966	0.651	0	0
Eef1d	0	1.51957839	0.601	0.256	0	0
Rps17	0	1.51249268	0.869	0.483	0	0
Ybx1	0	1.49781616	0.726	0.377	0	0
Tpt1	0	1.4884507	0.988	0.834	0	0
Igfbp4	0	1.48019123	0.475	0.094	0	0

Cct5	0	1.47370169	0.409	0.137	0	0
Rps25	0	1.46757046	0.9	0.558	0	0
Psma7	0	1.46068257	0.643	0.313	0	0
Nap1l1	0	1.45124139	0.56	0.243	0	0
Nme2	0	1.42353264	0.739	0.324	0	0
Ran	0	1.41628765	0.627	0.285	0	0
Cald1	0	1.41202011	0.359	0.035	0	0
Pfdn1	0	1.39385711	0.373	0.137	0	0
Ldha	0	1.39154387	0.597	0.342	0	0
Tcp1	0	1.38906431	0.363	0.114	0	0
Rrbp1	0	1.38677398	0.527	0.207	0	0
Ctnna1	0	1.38073461	0.284	0.062	0	0
Rtn4	0	1.37965572	0.378	0.12	0	0
Ezr	0	1.37903965	0.389	0.15	0	0
Hmgn1	0	1.37571814	0.513	0.179	0	0
Rpl14	0	1.36344663	0.843	0.444	0	0
Rpl4	0	1.3611916	0.7	0.343	0	0
Nfix	0	1.35545324	0.256	0.045	0	0
Kcnq1ot1	0	1.35435714	0.289	0.063	0	0
Dst	0	1.35202981	0.253	0.023	0	0
1110038B12	0	1.35053805	0.368	0.137	0	0
Pebp1	0	1.3466092	0.408	0.149	0	0
Ktn1	0	1.3361968	0.357	0.103	0	0
Eif2s2	0	1.32902298	0.684	0.373	0	0
St13	0	1.32885498	0.461	0.188	0	0
Pdap1	0	1.32672619	0.492	0.222	0	0
Zfas1	0	1.32278833	0.361	0.147	0	0
Eef1b2	0	1.32040824	0.83	0.459	0	0
Casp3	0	1.30619155	0.279	0.069	0	0
Sfr1	0	1.30574093	0.393	0.163	0	0
Hspa9	0	1.29720398	0.268	0.07	0	0
mt-Nd1	0	1.29281707	0.769	0.397	0	0
Serbp1	0	1.28297948	0.754	0.418	0	0
Rpl31	0	1.27900575	0.816	0.472	0	0
Mybbp1a	0	1.27591127	0.272	0.07	0	0
Sdc4	0	1.27445026	0.37	0.092	0	0
Canx	0	1.27123227	0.432	0.189	0	0
Ywhae	0	1.25639488	0.572	0.295	0	0
Eif3j1	0	1.25608811	0.388	0.168	0	0
Galnt1	0	1.25108383	0.297	0.1	0	0
Uqcc2	0	1.25001648	0.4	0.173	0	0
Mprip	0	1.24614485	0.26	0.057	0	0
Rpl36a	0	1.2337608	0.898	0.502	0	0

Taf1d	0	1.23180383	0.376	0.15	0	0
Gnl3	0	1.22111907	0.251	0.063	0	0
Ctsl	0	1.21756266	0.443	0.149	0	0
Rock2	0	1.21511506	0.315	0.112	0	0
Cct3	0	1.20815765	0.291	0.094	0	0
Eif3c	0	1.20478076	0.392	0.174	0	0
Calr	0	1.20440682	0.447	0.191	0	0
Pdia3	0	1.19870545	0.446	0.209	0	0
Nop56	0	1.1741004	0.269	0.083	0	0
Gas5	0	1.17335768	0.648	0.374	0	0
Prdx1	0	1.16653889	0.657	0.326	0	0
Cox4i1	0	1.16182107	0.723	0.471	0	0
Rps23	0	1.15866122	0.977	0.707	0	0
Eef2	0	1.1579723	0.609	0.327	0	0
Hspe1	0	1.15174709	0.493	0.225	0	0
Rps6	0	1.14611927	0.763	0.433	0	0
Ptms	0	1.13873881	0.348	0.127	0	0
Tpm4	0	1.12588931	0.454	0.204	0	0
Tmem176b	0	1.12056584	0.276	0.078	0	0
Rpl26	0	1.11849024	0.948	0.64	0	0
Rplp2	0	1.09195455	0.949	0.655	0	0
B2m	0	1.08050049	0.866	0.67	0	0
Rpl35	0	1.07068945	0.986	0.719	0	0
Dynll1	0	1.05735235	0.63	0.407	0	0
Rpl13	0	1.03904545	0.988	0.742	0	0
Rplp0	0	1.03789768	0.959	0.682	0	0
Hsp90b1	0	1.03570251	0.583	0.326	0	0
mt-Nd3	0	1.03233614	0.503	0.25	0	0
Rps2	0	1.02522059	0.892	0.563	0	0
Calm1	0	0.98894224	0.803	0.607	0	0
Rpl37	0	0.98856742	0.995	0.895	0	0
Hint1	0	0.97932759	0.624	0.351	0	0
Rpl24	0	0.93574672	0.948	0.719	0	0
Rpl7a	0	0.9236742	0.769	0.506	0	0
mt-Nd2	0	0.91692063	0.599	0.326	0	0
Rpl15	0	0.91030978	0.906	0.598	0	0
Rpl3	0	0.90584479	0.835	0.487	0	0
Rpsa	0	0.89024571	0.961	0.635	0	0
Rps3a1	0	0.87052249	0.974	0.723	0	0
Rps10	0	0.84436263	0.965	0.762	0	0
mt-Co3	0	0.84036509	0.954	0.757	0	0
Rps28	0	0.80375648	0.998	0.806	0	0
Rpl41	0	0.78938054	0.986	0.88	0	0

Fn1	0	0.77483644	0.437	0.136	0	0
Rpl10a	0	0.76136785	0.871	0.537	0	0
Rpl23	0	0.75801191	0.979	0.785	0	0
Rpl28	0	0.75359661	0.955	0.709	0	0
Rpl21	0	0.75285002	0.931	0.665	0	0
Rpl22	0	0.75086418	0.905	0.605	0	0
Rps26	0	0.74830147	0.983	0.737	0	0
Rpl27a	0	0.74724821	0.978	0.754	0	0
mt-Cytb	0	0.73437802	0.867	0.612	0	0
Rpl7	0	0.69317223	0.821	0.586	0	0
mt-Co2	0	0.68896034	0.938	0.745	0	0
Rpl38	0	0.67957621	0.989	0.805	0	0
Rps16	0	0.663537	0.951	0.733	0	0
Rpl36	0	0.65301617	0.965	0.709	0	0
mt-Atp6	0	0.63711118	0.93	0.698	0	0
Rpl32	0	0.56109603	0.95	0.674	0	0
Eif5b	6.56E-307	1.12546183	0.391	0.171	2.12E-302	0
Romo1	2.88E-305	1.10583598	0.521	0.306	9.31E-301	0
Ppia	4.22E-299	0.45623045	0.93	0.653	1.36E-294	0
Impdh2	6.23E-295	1.17499022	0.261	0.084	2.01E-290	0
Eif1ax	1.56E-294	1.24635072	0.356	0.155	5.04E-290	0
Lars2	1.64E-291	1.10184814	0.414	0.193	5.29E-287	0
Nars	1.14E-283	1.22979836	0.327	0.132	3.68E-279	0
Hspa5	7.07E-283	1.09222121	0.527	0.313	2.28E-278	0
Ly6e	1.58E-282	0.91861891	0.603	0.355	5.09E-278	0
Cd63	2.89E-281	0.95877253	0.449	0.227	9.34E-277	0
Vdac1	1.25E-279	1.20118672	0.336	0.143	4.05E-275	0
Prdx2	6.51E-279	0.60744241	0.53	0.278	2.10E-274	0
Cox7c	1.28E-278	0.70314658	0.809	0.637	4.15E-274	0
Rps4x	1.51E-271	0.54480497	0.91	0.639	4.88E-267	0
Tpr	2.42E-270	1.14837555	0.393	0.191	7.80E-266	0
Tomm20	5.43E-265	1.09085211	0.421	0.218	1.75E-260	0
mt-Nd4	2.64E-261	0.71934832	0.659	0.447	8.52E-257	0
Pgk1	6.15E-261	1.34702331	0.398	0.211	1.99E-256	0
Ranbp1	8.79E-261	1.04905573	0.407	0.199	2.84E-256	0
Anxa3	1.27E-260	1.2661755	0.319	0.14	4.11E-256	0
Fxr1	1.82E-258	1.14418333	0.317	0.132	5.86E-254	0
Atp5a1	1.13E-257	1.0291253	0.456	0.251	3.64E-253	0
Sms	1.59E-257	1.1075898	0.263	0.094	5.15E-253	0
Timp2	4.01E-256	1.03028856	0.325	0.134	1.30E-251	0
C1qbp	1.35E-255	1.02167723	0.29	0.109	4.36E-251	0
Denr	4.33E-255	1.1212217	0.286	0.111	1.40E-250	0
Dap	9.27E-255	1.20566453	0.304	0.126	2.99E-250	0

Rpl18a	4.25E-254	0.45235622	0.967	0.765	1.37E-249	0
Ssr3	4.19E-246	1.11459449	0.335	0.15	1.35E-241	0
Tbca	5.62E-243	1.07977044	0.439	0.249	1.81E-238	0
Rps5	7.43E-243	0.50227335	0.902	0.627	2.40E-238	0
Banf1	1.31E-240	1.07703005	0.345	0.161	4.22E-236	0
Syncrip	1.43E-239	1.08512229	0.289	0.116	4.63E-235	0
Smc4	4.69E-239	1.1880622	0.341	0.159	1.51E-234	0
Cct2	3.41E-238	1.06369105	0.331	0.149	1.10E-233	0
S100a11	4.17E-238	0.27000311	0.907	0.626	1.35E-233	0
Tubb5	1.60E-236	0.66506357	0.558	0.31	5.16E-232	0
Rpl30	2.22E-233	0.41090266	0.959	0.781	7.15E-229	0
Rpl34	4.41E-233	0.45638687	0.955	0.793	1.42E-228	0
Aprt	1.63E-229	1.10253261	0.432	0.256	5.27E-225	0
Hnrnpa3	7.25E-229	0.92501223	0.528	0.334	2.34E-224	0
Rps24	2.48E-227	0.36707631	0.992	0.82	8.01E-223	0
Btf3	9.55E-225	0.71786371	0.695	0.512	3.08E-220	0
Hnrnpab	2.41E-223	0.95776046	0.42	0.229	7.78E-219	0
Fkbp3	1.79E-222	1.06417208	0.302	0.133	5.77E-218	0
Eif4a1	2.25E-222	0.93226253	0.503	0.322	7.28E-218	0
Gm10076	3.68E-221	0.47527467	0.931	0.779	1.19E-216	0
Rps18	1.70E-218	0.43404682	0.875	0.592	5.47E-214	0
Csnk2a1	6.82E-209	1.05287037	0.365	0.193	2.20E-204	0
Sod1	2.50E-207	1.02673509	0.279	0.119	8.07E-203	0
Cnbp	9.42E-207	0.97927638	0.356	0.181	3.04E-202	0
Nucks1	3.28E-204	0.98212137	0.378	0.2	1.06E-199	0
Tm9sf3	1.23E-203	1.04259195	0.349	0.179	3.97E-199	0
Sh3glb1	2.92E-199	0.936108	0.452	0.276	9.41E-195	0
Pgam1	1.06E-197	1.1055443	0.396	0.236	3.43E-193	0
Aimp1	1.09E-195	1.0025062	0.339	0.174	3.54E-191	0
Myl12a	1.47E-195	0.9841579	0.496	0.349	4.73E-191	0
Hnrnpa2b1	9.35E-192	0.79557884	0.55	0.379	3.02E-187	0
Csde1	3.46E-191	0.96408825	0.386	0.216	1.12E-186	0
Anp32b	6.24E-190	0.86919352	0.434	0.257	2.02E-185	0
Dstn	1.68E-189	0.73821111	0.559	0.368	5.43E-185	0
Atp5b	5.20E-188	0.91168758	0.451	0.283	1.68E-183	0
Bst2	1.06E-185	0.70703958	0.272	0.117	3.41E-181	0
Hdlbp	3.88E-183	0.96336459	0.263	0.115	1.25E-178	0
Nme1	1.03E-182	0.89164284	0.376	0.204	3.34E-178	0
Elob	9.65E-182	0.71120162	0.66	0.544	3.11E-177	0
Rps20	2.50E-181	0.32680464	0.969	0.685	8.06E-177	0
Rbms1	1.08E-178	0.98793775	0.337	0.178	3.49E-174	0
Rps7	3.93E-176	0.34682599	0.916	0.683	1.27E-171	0
Sptbn1	1.83E-174	0.91717548	0.279	0.129	5.91E-170	0

Ssb	3.50E-170	0.92004977	0.345	0.19	1.13E-165	0
Sec62	1.59E-169	0.90383771	0.423	0.266	5.14E-165	0
Rac1	2.62E-169	0.95100436	0.414	0.264	8.44E-165	0
Rdx	5.15E-169	0.95506537	0.294	0.147	1.66E-164	0
Nop58	2.81E-166	0.8995392	0.265	0.122	9.07E-162	0
Rpl35a	3.61E-165	0.34340708	0.974	0.826	1.16E-160	0
Pcbp2	2.69E-164	0.87427967	0.455	0.303	8.68E-160	0
Cox6c	4.08E-161	0.54902304	0.743	0.613	1.32E-156	0
Dut	5.99E-161	0.73689415	0.329	0.166	1.93E-156	0
Kras	1.52E-159	0.8970749	0.255	0.117	4.91E-155	0
Hmgb1	6.41E-159	0.2567124	0.696	0.498	2.07E-154	0
Manf	9.51E-158	0.90863983	0.328	0.178	3.07E-153	0
Dbi	3.59E-156	0.76371594	0.445	0.276	1.16E-151	0
Cct8	4.94E-156	0.91686309	0.271	0.133	1.59E-151	0
Rpl39	1.57E-152	0.31289079	0.985	0.81	5.08E-148	0
Rbm8a	2.13E-152	0.93217293	0.342	0.198	6.86E-148	0
Sox4	2.09E-150	0.65160671	0.279	0.128	6.75E-146	0
Ptges3	2.19E-148	0.86080488	0.348	0.203	7.05E-144	0
Mrpl52	3.28E-146	0.69105119	0.436	0.269	1.06E-141	0
Rpl18	1.14E-144	0.33909949	0.876	0.673	3.67E-140	0
Atp5md	2.04E-141	0.63778633	0.63	0.518	6.58E-137	0
Tagln2	2.91E-141	0.74359125	0.432	0.276	9.40E-137	0
Rps9	1.15E-140	0.3116259	0.947	0.857	3.71E-136	0
Pdia6	3.62E-139	0.81662312	0.262	0.13	1.17E-134	0
Rpl10	1.03E-138	0.45017314	0.754	0.567	3.31E-134	0
Cks1b	4.22E-136	0.83874776	0.287	0.153	1.36E-131	0
Ppib	2.56E-135	0.76266379	0.445	0.309	8.27E-131	0
Rps27l	2.09E-133	0.66472605	0.474	0.316	6.74E-129	0
Txndc17	7.80E-133	0.96206289	0.34	0.214	2.52E-128	0
Esd	5.32E-131	0.90345836	0.336	0.206	1.72E-126	0
Krtcap2	2.71E-129	0.79179328	0.368	0.227	8.75E-125	0
Rps3	4.81E-129	0.32394324	0.857	0.67	1.55E-124	0
Atp5k	3.47E-128	0.5975305	0.607	0.486	1.12E-123	0
Cct7	1.20E-125	0.87940008	0.265	0.142	3.87E-121	0
P4hb	5.94E-125	0.77485954	0.284	0.156	1.92E-120	0
Vcp	3.02E-119	0.86235253	0.288	0.165	9.77E-115	0
Hnrnpu	6.89E-118	0.75202768	0.41	0.28	2.22E-113	0
Aldoa	1.29E-117	0.7784312	0.505	0.401	4.16E-113	0
Xrn2	3.66E-117	0.79778829	0.27	0.147	1.18E-112	0
Caprin1	1.74E-115	0.85975014	0.261	0.143	5.63E-111	0
Swi5	9.66E-112	0.79622714	0.344	0.223	3.12E-107	0
Ndufa7	9.28E-110	0.63846113	0.517	0.41	2.99E-105	0
Hcfc1r1	1.40E-108	1.00744829	0.25	0.143	4.50E-104	0

Ndufa2	5.10E-108	0.6416253	0.522	0.416	1.65E-103	0
Eif5	9.17E-108	0.7699499	0.396	0.281	2.96E-103	0
Vdac3	1.77E-105	0.83133691	0.252	0.142	5.73E-101	0
Hnrnph1	5.40E-103	0.74686493	0.321	0.201	1.74E-98	0
Rpl27	6.28E-101	0.25646732	0.86	0.691	2.03E-96	0
Atp5j2	2.04E-100	0.52906271	0.62	0.524	6.59E-96	0
Snrpf	4.17E-100	0.42605104	0.518	0.357	1.35E-95	0
Rpl22l1	1.62E-99	0.4898075	0.638	0.494	5.22E-95	0
Psmb4	5.09E-99	0.78718283	0.345	0.235	1.64E-94	0
Snrpd2	3.85E-98	0.6545083	0.4	0.277	1.24E-93	0
Selenof	1.26E-95	0.80839477	0.335	0.229	4.07E-91	0
Eif5a	2.43E-94	0.5760187	0.499	0.387	7.85E-90	0
Smarca5	5.03E-94	0.69969996	0.346	0.232	1.63E-89	0
Tcf25	5.39E-94	0.78356853	0.262	0.154	1.74E-89	0
Msn	1.48E-93	0.7327454	0.4	0.294	4.78E-89	0
Rpn1	1.08E-92	0.80602855	0.256	0.153	3.48E-88	0
Gnb1	8.82E-91	0.78113886	0.329	0.225	2.85E-86	0
Ubap2l	1.22E-88	0.7463023	0.255	0.152	3.95E-84	0
Bsg	2.28E-85	0.66486596	0.343	0.233	7.37E-81	0
Atp5g2	1.62E-81	0.44072276	0.526	0.389	5.22E-77	0
Snrpd1	1.53E-80	0.581792	0.352	0.237	4.95E-76	0
Lgals3	7.93E-79	0.4429528	0.496	0.37	2.56E-74	0
Txn1	1.23E-76	0.31677304	0.713	0.615	3.97E-72	0
Nipbl	4.41E-76	0.64619749	0.25	0.152	1.42E-71	0
Pcbp1	1.02E-74	0.68943409	0.326	0.232	3.30E-70	0
Hnrnpk	2.64E-72	0.54762454	0.459	0.369	8.54E-68	0
Zc3h15	2.62E-70	0.65123667	0.287	0.194	8.45E-66	0
Mrfap1	5.95E-70	0.68793877	0.265	0.175	1.92E-65	0
mt-Nd5	1.23E-69	0.47635453	0.369	0.264	3.98E-65	0
Gdi2	4.38E-67	0.62258713	0.31	0.217	1.42E-62	0
Ndufc1	1.42E-66	0.56755444	0.399	0.309	4.58E-62	0
Gapdh	2.06E-66	0.51057856	0.601	0.534	6.64E-62	0
Hnrnpa1	2.31E-66	0.51694439	0.359	0.256	7.47E-62	0
Eno1	3.23E-66	0.76231341	0.336	0.258	1.04E-61	0
Anp32e	8.75E-66	0.5932504	0.277	0.183	2.82E-61	0
Actn1	1.24E-64	0.66875571	0.273	0.185	4.02E-60	0
Top2a	2.08E-63	0.43168887	0.268	0.169	6.73E-59	0
Bola2	3.71E-63	0.57699369	0.32	0.225	1.20E-58	0
Cd9	2.49E-61	0.46276031	0.415	0.324	8.03E-57	0
Bzw1	3.44E-61	0.62932844	0.289	0.204	1.11E-56	0
Snrnp27	1.83E-60	0.65836644	0.261	0.179	5.92E-56	0
Eif3e	2.02E-59	0.60216472	0.297	0.209	6.51E-55	0
Chchd1	4.67E-59	0.71557349	0.25	0.171	1.51E-54	0

Txnl1	8.15E-59	0.74484612	0.291	0.216	2.63E-54	0
Ywhab	1.53E-58	0.65362245	0.366	0.299	4.93E-54	0
Ywhaq	2.99E-58	0.62312081	0.272	0.189	9.67E-54	0
Cd44	5.99E-58	0.66323522	0.331	0.251	1.93E-53	0
Snrpe	1.83E-55	0.31062743	0.523	0.407	5.89E-51	0
Sf3b2	2.21E-53	0.62115706	0.271	0.195	7.13E-49	0
Cox7b	4.41E-53	0.49304432	0.407	0.331	1.42E-48	0
Snrpd3	1.48E-51	0.58877469	0.329	0.254	4.77E-47	0
Rbm3	3.23E-51	0.40019536	0.496	0.413	1.04E-46	0
Hnrnpc	5.78E-51	0.58158264	0.288	0.21	1.87E-46	0
Snhg8	1.76E-50	0.602889	0.291	0.214	5.68E-46	0
Dek	6.30E-49	0.26380514	0.392	0.29	2.03E-44	0
Hnrnpa0	5.71E-47	0.45681591	0.356	0.277	1.84E-42	0
Dynlrb1	7.37E-46	0.6141092	0.275	0.207	2.38E-41	0
Cfl1	9.04E-46	0.30950916	0.644	0.616	2.92E-41	0
Morf4l1	1.05E-45	0.46955173	0.421	0.357	3.37E-41	0
Wnk1	7.86E-45	0.55323431	0.26	0.188	2.54E-40	0
Eif4g2	1.69E-44	0.48738376	0.435	0.385	5.47E-40	0
Gpx4	4.67E-44	0.5117824	0.439	0.391	1.51E-39	0
Nol7	6.23E-44	0.37961125	0.337	0.256	2.01E-39	0
Rbm39	7.47E-43	0.44102965	0.449	0.399	2.41E-38	0
Skp1a	1.00E-41	0.59206567	0.276	0.212	3.24E-37	0
Ndufa6	1.19E-41	0.57885838	0.368	0.313	3.84E-37	0
7-Sep	7.32E-40	0.62071877	0.272	0.212	2.36E-35	0
Dnaja1	1.13E-39	0.45750181	0.399	0.34	3.63E-35	0
Polr1d	1.61E-38	0.49701839	0.349	0.29	5.20E-34	0
Nop10	5.04E-38	0.40228136	0.314	0.239	1.63E-33	0
Hsbp1	2.90E-37	0.59151755	0.25	0.191	9.35E-33	0
2410006H16I	1.72E-36	0.42539107	0.457	0.388	5.56E-32	0
Mdh2	3.46E-35	0.59699046	0.277	0.225	1.12E-30	0
Ndufa13	1.01E-34	0.45576665	0.455	0.425	3.27E-30	0
Csnk1a1	3.79E-34	0.4959507	0.293	0.233	1.23E-29	0
Lamp1	4.80E-34	0.4656279	0.287	0.225	1.55E-29	0
Pdcd5	6.27E-33	0.53360865	0.269	0.212	2.02E-28	0
Prelid1	4.43E-32	0.5940192	0.3	0.255	1.43E-27	0
Psma3	1.16E-31	0.48205888	0.3	0.246	3.76E-27	0
Ywhaz	7.98E-31	0.35854548	0.505	0.485	2.58E-26	0
Metap2	2.47E-30	0.40165718	0.292	0.233	7.98E-26	0
Pafah1b1	1.02E-29	0.5037148	0.301	0.252	3.28E-25	0
Ndufb9	1.07E-28	0.46870089	0.39	0.358	3.45E-24	0
Polr2k	2.82E-28	0.52143119	0.285	0.238	9.12E-24	0
Tpm3	8.02E-28	0.49195912	0.41	0.391	2.59E-23	0
AY036118	3.39E-27	0.79012464	0.321	0.285	1.09E-22	0

Ubxn1	1.26E-24	0.48737891	0.272	0.229	4.06E-20	0
Eif3h	8.16E-24	0.43187103	0.313	0.27	2.63E-19	0
Ywhah	9.65E-24	0.49668841	0.271	0.23	3.12E-19	0
Srrm2	7.62E-23	0.38743465	0.352	0.314	2.46E-18	0
Slc25a3	1.38E-22	0.36941172	0.415	0.388	4.45E-18	0
Sfpq	2.53E-22	0.41586712	0.269	0.224	8.17E-18	0
Lsm6	2.76E-22	0.47074524	0.259	0.218	8.90E-18	0
Ndufs6	9.75E-22	0.50932828	0.288	0.254	3.15E-17	0
Ube2i	4.14E-21	0.40495134	0.279	0.239	1.34E-16	0
Neat1	2.55E-20	0.54741713	0.251	0.211	8.22E-16	0
Psmb1	1.04E-19	0.38485351	0.333	0.299	3.35E-15	0
Bri3	4.00E-19	0.40772496	0.294	0.258	1.29E-14	0
Pomp	6.49E-19	0.40169888	0.367	0.344	2.10E-14	0
Dync1i2	1.39E-18	0.4365282	0.311	0.281	4.48E-14	0
Prpf4b	1.85E-18	0.31533722	0.282	0.241	5.97E-14	0
Flna	1.51E-17	0.4313094	0.34	0.317	4.88E-13	0
Atp5o	4.09E-17	0.40867505	0.274	0.241	1.32E-12	0
Cebpz	4.29E-17	0.51310777	0.25	0.22	1.38E-12	0
Snrpb	6.47E-16	0.34453156	0.307	0.274	2.09E-11	0
Cox6a1	2.58E-15	0.39006076	0.335	0.316	8.33E-11	0
Jpt1	1.09E-14	0.38705165	0.26	0.229	3.51E-10	0
Uqcr11	3.66E-14	0.30770466	0.381	0.358	1.18E-09	0
S100a13	4.07E-14	0.5476813	0.301	0.29	1.31E-09	0
Trmt112	1.14E-12	0.35617485	0.267	0.239	3.69E-08	0
Tmem258	3.08E-10	0.29322211	0.392	0.388	9.94E-06	0
Psme1	1.19E-08	0.29894371	0.319	0.309	0.00038302	0
Fus	1.32E-08	0.28543396	0.272	0.254	0.00042607	0
Trir	1.85E-08	0.344038	0.281	0.272	0.00059806	0
Ppp1ca	6.24E-08	0.29815405	0.366	0.373	0.00201586	0
Srsf2	6.71E-08	0.29269382	0.274	0.26	0.0021671	0
Gnb2	3.31E-07	0.32246999	0.361	0.372	0.01068527	0
Arpp19	0.00122522	0.25239588	0.332	0.348	1	0
Ndufb8	0.00133784	0.28855643	0.29	0.3	1	0
Psma2	0.00276211	0.25031185	0.31	0.323	1	0
Dnajc7	0	3.90970723	0.86	0.158	0	1
Ebf1	0	3.34565832	0.971	0.159	0	1
Ly6d	0	3.33703267	0.737	0.095	0	1
Ighm	0	3.19788087	0.913	0.176	0	1
Vpreb3	0	3.17772423	0.96	0.13	0	1
Cd79b	0	3.08724837	0.772	0.119	0	1
Fcrla	0	2.89364616	0.518	0.053	0	1
Coq7	0	2.85774768	0.404	0.064	0	1
Arl5c	0	2.81417147	0.481	0.059	0	1

Cd79a	0	2.75525222	0.846	0.149	0	1
Pafah1b3	0	2.72785784	0.638	0.141	0	1
4930597A21	0	2.72145965	0.4	0.039	0	1
Atp1b1	0	2.68295715	0.436	0.055	0	1
Tifa	0	2.64981941	0.475	0.075	0	1
Jund	0	2.6049053	0.925	0.481	0	1
Spib	0	2.59262812	0.409	0.044	0	1
Bach2	0	2.58122478	0.43	0.052	0	1
Gm30211	0	2.52160568	0.358	0.04	0	1
Chchd10	0	2.51419821	0.577	0.117	0	1
Cd72	0	2.45681355	0.427	0.065	0	1
Rhoh	0	2.44002817	0.382	0.063	0	1
Akap12	0	2.398621	0.388	0.049	0	1
Cecr2	0	2.38754943	0.361	0.04	0	1
Ptprcap	0	2.38047699	0.591	0.143	0	1
Cnp	0	2.36493431	0.381	0.064	0	1
Bcl7a	0	2.34716824	0.39	0.062	0	1
Herpud1	0	2.26399277	0.457	0.133	0	1
Irf4	0	2.25339463	0.329	0.044	0	1
Myl4	0	2.25337822	0.384	0.053	0	1
Iglc3	0	2.24853179	0.271	0.04	0	1
Btg1	0	2.19490873	0.955	0.596	0	1
Cd2	0	2.14736514	0.28	0.031	0	1
Foxp1	0	2.10128274	0.524	0.199	0	1
C1galt1	0	2.06162131	0.331	0.072	0	1
Tmsb10	0	1.9438908	0.998	0.728	0	1
Igkc	0	1.91133426	0.89	0.14	0	1
H3f3a	0	1.45591762	0.947	0.747	0	1
Uba52	0	1.17308055	0.953	0.743	0	1
Rps27	0	1.15770652	0.998	0.905	0	1
H3f3b	0	1.07465584	0.937	0.817	0	1
Ptma	0	1.0654348	0.98	0.669	0	1
Fau	0	0.9731874	0.998	0.938	0	1
mt-Co1	0	0.93927952	0.958	0.878	0	1
Cxcr4	9.11E-308	2.06994831	0.505	0.199	2.94E-303	1
Gm49980	2.08E-303	2.1446297	0.323	0.078	6.72E-299	1
Il7r	2.37E-302	1.88288983	0.281	0.057	7.66E-298	1
Sec63	8.59E-292	2.05308348	0.377	0.111	2.77E-287	1
Blnk	5.48E-288	1.81608164	0.283	0.061	1.77E-283	1
Rpl19	6.94E-286	0.94839984	0.947	0.747	2.24E-281	1
Rps15a	3.85E-279	0.85431819	0.974	0.772	1.24E-274	1
Rpl37a	1.16E-267	0.75070042	0.991	0.898	3.74E-263	1
Rpl18a1	2.42E-266	0.85911342	0.965	0.796	7.81E-262	1

Rpl341	6.01E-260	0.83491375	0.962	0.816	1.94E-255	1
Dusp2	3.87E-259	1.95125636	0.304	0.078	1.25E-254	1
Rpl11	6.52E-243	0.9097514	0.918	0.728	2.10E-238	1
Myb	8.42E-241	1.96556416	0.327	0.098	2.72E-236	1
Rps201	8.02E-236	0.79306226	0.975	0.727	2.59E-231	1
Pgls	1.41E-232	1.78198428	0.574	0.328	4.55E-228	1
Rpl35a1	1.72E-229	0.72697296	0.978	0.848	5.56E-225	1
Rps13	1.16E-221	0.82038932	0.933	0.787	3.73E-217	1
2410006H16	1.65E-219	1.48526795	0.624	0.383	5.33E-215	1
Lrmp	7.67E-215	1.83387405	0.292	0.086	2.48E-210	1
Rpl9	8.98E-214	0.81733523	0.9	0.765	2.90E-209	1
Rpl271	3.40E-209	0.90939614	0.876	0.714	1.10E-204	1
Syk	2.54E-207	1.87551806	0.404	0.169	8.21E-203	1
Rpl301	2.52E-202	0.72110419	0.953	0.808	8.12E-198	1
mt-Nd4l	6.38E-199	1.28149139	0.655	0.434	2.06E-194	1
Mzb1	1.50E-198	1.39521474	0.292	0.084	4.85E-194	1
Zfp706	6.85E-198	1.64840895	0.507	0.268	2.21E-193	1
Serinc3	2.27E-197	1.54315759	0.569	0.345	7.34E-193	1
Rps3a11	2.43E-192	0.74829276	0.945	0.763	7.84E-188	1
Smarca4	6.22E-186	1.72313073	0.341	0.127	2.01E-181	1
Nsa2	1.44E-184	1.31901337	0.656	0.47	4.66E-180	1
Pkig	1.97E-184	1.6614797	0.339	0.125	6.35E-180	1
Rpl281	1.02E-176	0.7674234	0.918	0.749	3.28E-172	1
Rps91	3.31E-176	0.67901709	0.929	0.872	1.07E-171	1
Rpl391	5.77E-175	0.62873823	0.992	0.835	1.86E-170	1
Rps261	8.11E-173	0.70167238	0.958	0.775	2.62E-168	1
Tcf3	6.14E-170	1.70195881	0.277	0.091	1.98E-165	1
Rps71	7.68E-166	0.7096226	0.907	0.718	2.48E-161	1
Rps241	5.64E-165	0.53343269	0.991	0.845	1.82E-160	1
Rps19	1.89E-164	0.68897723	0.926	0.718	6.10E-160	1
Rps29	7.91E-158	0.50562806	0.998	0.951	2.55E-153	1
Cd24a	3.56E-153	1.31864678	0.592	0.411	1.15E-148	1
Rpl29	3.88E-153	0.98127562	0.733	0.565	1.25E-148	1
Naca	1.09E-152	0.97683414	0.731	0.563	3.52E-148	1
Rpl181	1.12E-152	0.74463937	0.87	0.704	3.63E-148	1
Ndufb1-ps	6.74E-151	0.95306661	0.732	0.632	2.18E-146	1
Rps281	1.33E-146	0.57106278	0.998	0.834	4.28E-142	1
Sec11c	1.35E-144	1.71314197	0.476	0.3	4.35E-140	1
Sox41	1.21E-142	1.52755914	0.34	0.145	3.90E-138	1
Rpl361	2.35E-142	0.59611369	0.954	0.748	7.58E-138	1
Cytip	1.12E-141	1.60968176	0.362	0.168	3.62E-137	1
Plekha2	8.52E-141	1.58936515	0.25	0.086	2.75E-136	1
Selenow	8.66E-140	1.41343904	0.45	0.252	2.80E-135	1

Cd37	2.41E-139	1.69470446	0.369	0.179	7.78E-135	1
Rpl371	1.52E-137	0.50724626	0.985	0.91	4.92E-133	1
Elof1	2.39E-136	1.66330203	0.324	0.142	7.71E-132	1
Klhl24	5.33E-134	1.57422773	0.259	0.095	1.72E-129	1
Rps31	1.68E-132	0.70012001	0.846	0.699	5.43E-128	1
Dok3	6.01E-130	1.73060428	0.33	0.154	1.94E-125	1
Pdcd4	9.49E-128	1.3633557	0.425	0.23	3.06E-123	1
Rpl321	2.04E-125	0.57183933	0.932	0.717	6.60E-121	1
Ypel3	6.77E-123	1.49079471	0.399	0.219	2.18E-118	1
mt-Cytb1	1.57E-120	0.68983637	0.79	0.656	5.08E-116	1
Oaz1	1.89E-120	0.90499229	0.706	0.637	6.09E-116	1
Rpl351	1.33E-118	0.53975394	0.976	0.76	4.30E-114	1
Gm100761	2.75E-109	0.56792097	0.91	0.803	8.89E-105	1
Rpl27a1	2.20E-107	0.52568175	0.935	0.791	7.10E-103	1
Slc38a2	6.77E-107	1.31619005	0.504	0.363	2.19E-102	1
Rps11	2.03E-103	0.56541184	0.873	0.72	6.55E-99	1
mt-Co21	2.26E-103	0.51538056	0.857	0.78	7.30E-99	1
Malat1	7.74E-103	0.88194798	0.949	0.958	2.50E-98	1
Uqcrh	5.94E-100	1.06190255	0.594	0.51	1.92E-95	1
mt-Co31	8.27E-100	0.43972115	0.884	0.792	2.67E-95	1
Pim1	9.77E-96	1.15031258	0.495	0.329	3.15E-91	1
Dusp5	1.56E-95	1.48290394	0.251	0.109	5.02E-91	1
Ubb	1.81E-95	0.59644396	0.806	0.758	5.83E-91	1
mt-Atp61	2.04E-94	0.51859815	0.834	0.741	6.59E-90	1
Rpl411	4.54E-91	0.41918877	0.977	0.896	1.47E-86	1
Rps51	1.34E-89	0.52881102	0.853	0.672	4.33E-85	1
Marcks	3.50E-89	1.26366464	0.344	0.188	1.13E-84	1
Unc93b1	3.98E-81	1.39207856	0.273	0.137	1.29E-76	1
Hmgb11	6.26E-81	0.531459	0.684	0.528	2.02E-76	1
Nol71	7.27E-80	1.31113928	0.395	0.263	2.35E-75	1
Rps101	5.33E-79	0.45484966	0.913	0.797	1.72E-74	1
Rpl71	9.81E-77	0.66170388	0.721	0.629	3.17E-72	1
Snrpf1	3.06E-73	1.09425627	0.502	0.382	9.87E-69	1
Rps4x1	8.19E-73	0.51065071	0.826	0.686	2.64E-68	1
Chchd2	2.21E-72	0.86000022	0.613	0.582	7.12E-68	1
Rpl211	1.38E-71	0.48182114	0.842	0.712	4.44E-67	1
Lmo4	2.24E-70	1.48093591	0.367	0.253	7.25E-66	1
Cmtm7	7.12E-68	1.24937642	0.357	0.232	2.30E-63	1
Tsc22d3	7.66E-67	1.43818043	0.295	0.17	2.47E-62	1
Nfkbia	3.43E-66	1.12163966	0.362	0.232	1.11E-61	1
Rps181	1.22E-65	0.41347532	0.821	0.639	3.94E-61	1
Snrpe1	1.07E-64	0.84360048	0.529	0.423	3.44E-60	1
Blvrb	1.39E-64	0.96486246	0.28	0.149	4.50E-60	1

Scand1	2.16E-64	1.23394523	0.437	0.352	6.96E-60	1
Rpl381	1.96E-63	0.34798492	0.965	0.834	6.34E-59	1
Serp1	9.71E-62	1.33403478	0.389	0.286	3.13E-57	1
Paip2	9.67E-61	1.27799211	0.363	0.253	3.12E-56	1
Ddx5	3.06E-59	0.88827071	0.572	0.549	9.88E-55	1
Arpc5l	4.61E-59	1.32277864	0.272	0.158	1.49E-54	1
Rps27a	2.72E-58	0.31515814	0.937	0.89	8.78E-54	1
Sub1	5.70E-58	0.92474073	0.532	0.478	1.84E-53	1
Srp14	7.69E-57	1.26918064	0.378	0.281	2.48E-52	1
H2-K1	2.33E-56	0.88525135	0.534	0.455	7.53E-52	1
H2-D1	3.47E-56	0.72905537	0.66	0.663	1.12E-51	1
Mef2c	6.86E-56	1.08344244	0.266	0.149	2.22E-51	1
Gm47283	1.10E-55	1.21450536	0.271	0.159	3.54E-51	1
Ifi27l2a	8.66E-54	0.93927827	0.325	0.192	2.80E-49	1
Eif3f	6.65E-52	1.16763233	0.379	0.285	2.15E-47	1
Cox7c1	4.09E-50	0.5708144	0.693	0.672	1.32E-45	1
Rps161	1.58E-49	0.37122464	0.864	0.773	5.09E-45	1
Rpl241	1.66E-48	0.39933728	0.849	0.761	5.37E-44	1
Btg2	2.82E-47	0.76739847	0.439	0.337	9.11E-43	1
Rpl101	3.65E-47	0.57004178	0.669	0.602	1.18E-42	1
Atp5e	5.14E-46	0.56249601	0.692	0.7	1.66E-41	1
Rpl221	5.63E-45	0.39519699	0.801	0.659	1.82E-40	1
Smim14	8.34E-44	1.27047496	0.286	0.195	2.69E-39	1
Ftl1	5.21E-42	0.30664803	0.795	0.811	1.68E-37	1
Btf31	5.70E-42	0.65154894	0.578	0.549	1.84E-37	1
Erp29	1.96E-41	1.17847732	0.296	0.207	6.32E-37	1
mt-Atp8	2.83E-39	1.02490063	0.355	0.273	9.14E-35	1
Rpl10a1	4.83E-39	0.37621117	0.734	0.598	1.56E-34	1
Clta	8.41E-38	1.01478561	0.382	0.318	2.72E-33	1
Cox8a	1.59E-37	0.51838187	0.68	0.688	5.12E-33	1
Rpl36al	1.93E-36	0.60038622	0.575	0.51	6.23E-32	1
Calm2	4.61E-36	0.8288499	0.511	0.488	1.49E-31	1
Rpl231	4.40E-35	0.25137384	0.92	0.819	1.42E-30	1
Lyn	8.13E-35	1.14897174	0.292	0.213	2.63E-30	1
Klf2	9.60E-35	1.10434626	0.304	0.222	3.10E-30	1
Snrpg	1.56E-33	0.50043037	0.58	0.504	5.04E-29	1
Grk2	7.36E-33	1.08990233	0.334	0.27	2.38E-28	1
Supt4a	2.35E-32	1.15242416	0.338	0.281	7.60E-28	1
Rpl151	5.41E-32	0.35009597	0.753	0.657	1.75E-27	1
Cox7a2	3.49E-30	0.7578966	0.503	0.508	1.13E-25	1
Limd2	4.68E-28	1.23012198	0.286	0.226	1.51E-23	1
Pfdn5	5.34E-27	0.79310803	0.461	0.463	1.72E-22	1
Srsf3	2.10E-23	0.80897271	0.417	0.396	6.77E-19	1

Atp5l	1.87E-22	0.55921691	0.561	0.616	6.05E-18	1
Cox5b	3.39E-20	0.67001041	0.466	0.477	1.09E-15	1
Tra2b	4.32E-20	1.05779167	0.3	0.259	1.39E-15	1
Mbnl1	1.23E-18	0.9460664	0.351	0.329	3.96E-14	1
Calm11	3.63E-18	0.52239449	0.587	0.654	1.17E-13	1
Fam107b	3.08E-17	1.07538406	0.254	0.212	9.94E-13	1
mt-Nd41	3.60E-16	0.41340124	0.499	0.492	1.16E-11	1
Smdt1	4.86E-15	0.8542198	0.35	0.342	1.57E-10	1
Capzb	5.49E-15	0.2655217	0.183	0.31	1.77E-10	1
Hnrnpa01	1.20E-14	0.83359611	0.317	0.292	3.87E-10	1
Ndufa11	2.19E-12	0.85825725	0.283	0.262	7.08E-08	1
Hnrnpl	5.05E-12	0.95778324	0.254	0.226	1.63E-07	1
Erh	5.10E-12	0.79363235	0.26	0.228	1.65E-07	1
Rbm31	5.37E-12	0.57912438	0.423	0.432	1.73E-07	1
Cox7a2l	1.20E-11	0.80614701	0.25	0.217	3.87E-07	1
Son	1.37E-11	0.77016885	0.374	0.39	4.44E-07	1
Pnrc1	1.42E-11	0.82898198	0.329	0.319	4.59E-07	1
Clk1	1.58E-11	0.88053909	0.301	0.286	5.11E-07	1
H2afv	2.43E-11	0.47787514	0.318	0.285	7.86E-07	1
Dync1i21	1.46E-10	0.2888817	0.187	0.296	4.72E-06	1
Cycs	1.51E-10	0.2551568	0.157	0.252	4.89E-06	1
Rpl22l11	1.77E-10	0.29840665	0.534	0.524	5.71E-06	1
Nedd8	2.31E-10	0.78639861	0.347	0.359	7.46E-06	1
Snu13	2.37E-10	0.84145021	0.289	0.273	7.64E-06	1
Timm10b	9.70E-10	0.28845955	0.173	0.272	3.13E-05	1
Actr2	1.09E-09	0.2763061	0.21	0.325	3.52E-05	1
Ucp2	1.96E-09	0.6568234	0.355	0.352	6.34E-05	1
Capza2	2.08E-09	0.29430309	0.187	0.288	6.72E-05	1
Tax1bp1	4.74E-09	0.27439875	0.168	0.261	0.00015305	1
Rac2	1.21E-08	0.57719505	0.392	0.414	0.0003892	1
Bri31	1.34E-08	0.29399861	0.179	0.273	0.00043275	1
Arf1	1.54E-08	0.33350589	0.193	0.296	0.00049832	1
Atp6v0b	3.03E-08	0.31672823	0.192	0.295	0.00097698	1
Rbm391	4.85E-08	0.72834429	0.378	0.412	0.0015644	1
Ptbp3	5.53E-08	0.32875458	0.189	0.285	0.001786	1
Psma31	9.07E-08	0.27988962	0.177	0.265	0.00292937	1
Atp5mpl	9.19E-08	0.61628586	0.36	0.38	0.00296604	1
Cox6b1	1.12E-07	0.48930695	0.447	0.505	0.00360764	1
Ndufa1	1.14E-07	0.31954699	0.244	0.371	0.00368129	1
Akap13	1.15E-07	0.78677693	0.26	0.248	0.00370747	1
Gas51	1.56E-07	0.39824727	0.424	0.434	0.0050492	1
Hnrnpm	2.48E-07	0.77557961	0.297	0.3	0.00799694	1
Map1lc3b	3.45E-07	0.5951164	0.4	0.439	0.0111538	1

Atp6v1f	3.56E-07	0.8330336	0.312	0.328	0.01149934	1
Pomp1	4.61E-07	0.26036435	0.244	0.358	0.01487225	1
Atp6v1g1	1.95E-06	0.38110255	0.242	0.356	0.06282373	1
H2afy	2.17E-06	0.63575972	0.257	0.243	0.06993514	1
Edf1	5.06E-06	0.31693203	0.221	0.323	0.16351455	1
Arpc4	5.12E-06	0.44225244	0.187	0.275	0.16533212	1
Ndufb81	6.32E-06	0.30661021	0.211	0.305	0.20412619	1
Vamp8	8.57E-06	0.43931852	0.199	0.289	0.27662467	1
Eif3h1	8.85E-06	0.71756179	0.276	0.279	0.28569657	1
Cox6c1	9.10E-06	0.30881453	0.566	0.647	0.29364524	1
Ppp1ca1	1.17E-05	0.29353504	0.265	0.381	0.3778161	1
Micos10	2.32E-05	0.28187365	0.197	0.28	0.74920429	1
Atp5md1	2.38E-05	0.40299764	0.471	0.549	0.76817616	1
Eif3k	2.65E-05	0.70214283	0.298	0.315	0.85487998	1
Ly6e1	2.88E-05	0.38306948	0.402	0.409	0.92940556	1
Ube2b	3.16E-05	0.37780859	0.21	0.298	1	1
Psmb11	4.08E-05	0.69318265	0.294	0.307	1	1
Prpf4b1	4.51E-05	0.75372041	0.251	0.249	1	1
Ubl5	6.68E-05	0.51660601	0.442	0.53	1	1
Rpl7a1	7.63E-05	0.29561944	0.523	0.566	1	1
Csnk1a11	8.47E-05	0.31257979	0.182	0.251	1	1
Sumo2	0.00012183	0.55638949	0.331	0.355	1	1
Iqgap1	0.000151	0.28815515	0.288	0.404	1	1
Cd47	0.00017432	0.32611862	0.291	0.414	1	1
Ndufs61	0.00021971	0.37009324	0.191	0.267	1	1
Tmem2581	0.00031319	0.27352349	0.285	0.398	1	1
Mpc2	0.0004508	0.57512877	0.186	0.263	1	1
Atp5k1	0.00054319	0.41863869	0.447	0.518	1	1
Polr1d1	0.0005564	0.67777494	0.285	0.304	1	1
Fis1	0.00060904	0.73996886	0.327	0.38	1	1
Uqcrb	0.00062837	0.33572014	0.24	0.333	1	1
Hnrnpk1	0.00073924	0.57506517	0.348	0.391	1	1
Nop101	0.00095553	0.26650779	0.196	0.26	1	1
Srsf5	0.00097395	0.62997513	0.341	0.39	1	1
Atp5a11	0.00097505	0.27855205	0.225	0.301	1	1
Atp5c1	0.00099735	0.37799467	0.21	0.287	1	1
Hnrnpa11	0.00105355	0.61875361	0.27	0.279	1	1
Sf3b1	0.00108244	0.79315686	0.257	0.271	1	1
Hnrnpf	0.00135849	0.31736658	0.243	0.33	1	1
Ywhaz1	0.00162473	0.55617742	0.411	0.496	1	1
Ndufb3	0.0017279	0.4336009	0.188	0.257	1	1
Fus1	0.00290152	0.39317894	0.198	0.263	1	1
Tomm6	0.00348223	0.56660174	0.362	0.427	1	1

Atp5d	0.00390809	0.37506762	0.21	0.281	1	1
mt-Nd51	0.00394019	0.49702523	0.276	0.287	1	1
Rbx1	0.00399598	0.63520779	0.329	0.381	1	1
Ndufb11	0.00411819	0.45190714	0.242	0.334	1	1
Gabarapl2	0.00491222	0.50020389	0.199	0.269	1	1
Dad1	0.00513559	0.33176185	0.191	0.251	1	1
Psmb3	0.00565569	0.47332886	0.225	0.305	1	1
Pabpc1	0.00684247	0.32810128	0.491	0.596	1	1
Ndufa21	0.00714335	0.26260717	0.332	0.448	1	1
Il1b	0	4.64996621	0.879	0.078	0	2
Cxcl2	0	4.62348217	0.916	0.119	0	2
Wfdc17	0	3.73631263	0.527	0.085	0	2
Ifitm1	0	3.4639631	0.557	0.077	0	2
Dusp1	0	3.35437714	0.869	0.173	0	2
Il1r2	0	3.22093918	0.796	0.133	0	2
Cebpb	0	3.18915072	0.969	0.346	0	2
Acod1	0	3.03282979	0.518	0.038	0	2
Csf3r	0	3.03170038	0.638	0.071	0	2
Clec4d	0	3.01582415	0.698	0.08	0	2
Trem1	0	3.01373776	0.741	0.131	0	2
G0s2	0	2.9324794	0.737	0.197	0	2
Slpi	0	2.92611634	0.862	0.239	0	2
Ccl6	0	2.87838982	0.838	0.131	0	2
Nlrp3	0	2.87716035	0.551	0.056	0	2
Srgn	0	2.85592954	0.99	0.474	0	2
Mxd1	0	2.81868323	0.808	0.212	0	2
Hcar2	0	2.7756905	0.451	0.036	0	2
Junb	0	2.77117626	0.918	0.396	0	2
Slc7a11	0	2.70994176	0.6	0.093	0	2
Retnlg	0	2.66937793	0.919	0.218	0	2
Btg21	0	2.66234353	0.859	0.301	0	2
Msrb1	0	2.64045611	0.861	0.279	0	2
Egr1	0	2.59554153	0.615	0.145	0	2
Lilr4b	0	2.5718653	0.697	0.165	0	2
Hdc	0	2.57001308	0.682	0.161	0	2
Slc16a3	0	2.5590772	0.616	0.122	0	2
Lmnb1	0	2.52441347	0.786	0.257	0	2
Cxcr2	0	2.51388939	0.527	0.068	0	2
Fxyd5	0	2.50854049	0.893	0.329	0	2
Clec4e	0	2.46446801	0.548	0.141	0	2
Tyrobp	0	2.40315504	0.987	0.368	0	2
Tnfaip2	0	2.39876117	0.515	0.159	0	2
Plek	0	2.38860642	0.532	0.125	0	2

Fos	0	2.38258726	0.831	0.324	0	2
Grina	0	2.30789187	0.797	0.268	0	2
Adam8	0	2.3065035	0.594	0.126	0	2
Lst1	0	2.29203313	0.649	0.236	0	2
Mmp9	0	2.21469518	0.792	0.175	0	2
Ifitm2	0	2.20413053	0.865	0.425	0	2
Ccr1	0	2.18871175	0.453	0.077	0	2
Ptgs2	0	2.18628739	0.305	0.044	0	2
Mcl1	0	2.17130903	0.792	0.342	0	2
Slc15a3	0	2.16406697	0.449	0.06	0	2
Lilrb4a	0	2.13467694	0.54	0.147	0	2
Gsr	0	2.13408429	0.734	0.278	0	2
Entpd1	0	2.13116746	0.374	0.046	0	2
Ets2	0	2.09366675	0.523	0.14	0	2
Nfkbiz	0	2.0877202	0.431	0.112	0	2
Sell	0	2.08743011	0.561	0.163	0	2
Tpd52	0	2.0822291	0.54	0.153	0	2
S100a111	0	2.0593708	0.995	0.66	0	2
Lrg1	0	2.05150669	0.453	0.153	0	2
Litaf	0	2.03749698	0.656	0.235	0	2
Stk17b	0	2.03714374	0.739	0.288	0	2
Plaur	0	2.03494094	0.691	0.291	0	2
Ncf2	0	2.03114479	0.604	0.208	0	2
Selenon	0	2.0272868	0.396	0.069	0	2
Lcp1	0	2.01838617	0.743	0.29	0	2
Plk3	0	2.01486174	0.354	0.05	0	2
Fth1	0	2.00524706	0.982	0.913	0	2
Fosl2	0	2.00113484	0.466	0.128	0	2
Samsn1	0	1.99469142	0.699	0.278	0	2
Vps37b	0	1.98973537	0.419	0.114	0	2
Ndel1	0	1.9891683	0.446	0.124	0	2
Sorl1	0	1.95208339	0.442	0.126	0	2
C5ar1	0	1.94933553	0.419	0.103	0	2
Il1f9	0	1.94557015	0.323	0.037	0	2
Cyp4f18	0	1.92764562	0.392	0.069	0	2
Trib1	0	1.91661146	0.442	0.137	0	2
Osm	0	1.91558472	0.376	0.092	0	2
Cd300lf	0	1.91038875	0.377	0.08	0	2
Pim11	0	1.90419586	0.687	0.313	0	2
Hp	0	1.9026568	0.778	0.27	0	2
Kctd12	0	1.90253251	0.485	0.167	0	2
Slfn1	0	1.86457217	0.319	0.053	0	2
Selplg	0	1.85894115	0.467	0.141	0	2

Il1rn	0	1.8307945	0.263	0.044	0	2
Slc2a3	0	1.81331068	0.343	0.081	0	2
Cd300ld	0	1.81086938	0.28	0.027	0	2
Arg2	0	1.81069722	0.292	0.029	0	2
Mcemp1	0	1.80931376	0.54	0.193	0	2
Ptprc	0	1.80830442	0.627	0.245	0	2
Gda	0	1.77026424	0.515	0.185	0	2
S100a9	0	1.76875367	0.996	0.444	0	2
Mrpl33	0	1.75624641	0.793	0.427	0	2
Mmp8	0	1.72801703	0.648	0.162	0	2
Picalm	0	1.72202398	0.534	0.2	0	2
Lsp1	0	1.70985265	0.642	0.276	0	2
Alox5ap	0	1.6884731	0.716	0.295	0	2
Cd33	0	1.67084801	0.321	0.073	0	2
Map1lc3b1	0	1.66691152	0.719	0.412	0	2
Cd52	0	1.65595344	0.967	0.481	0	2
Btg11	0	1.64051355	0.968	0.595	0	2
Actg1	0	1.63642222	0.961	0.696	0	2
Taldo1	0	1.60886825	0.683	0.372	0	2
S100a8	0	1.57746224	0.999	0.462	0	2
Rdh12	0	1.56554796	0.259	0.042	0	2
Fcer1g	0	1.54546748	0.899	0.358	0	2
Pglyrp1	0	1.36228074	0.712	0.243	0	2
Txn11	0	1.34165112	0.873	0.616	0	2
Ftl11	0	1.27734054	0.955	0.797	0	2
H3f3b1	0	1.2748879	0.951	0.816	0	2
Wfdc21	0	1.17446863	0.84	0.243	0	2
S100a61	0	1.16770139	0.97	0.594	0	2
Tmsb4x	0	1.15898504	0.999	0.897	0	2
Actb	0	1.14868773	0.999	0.945	0	2
Eif1	0	1.05277575	0.962	0.829	0	2
Thbs1	6.07E-307	2.02521412	0.527	0.193	1.96E-302	2
Gm20406	2.81E-296	1.63121657	0.262	0.05	9.06E-292	2
Tlr2	6.28E-283	1.63754322	0.277	0.059	2.03E-278	2
Cd14	6.02E-281	2.11040947	0.454	0.163	1.94E-276	2
Pilra	5.68E-278	1.73111681	0.37	0.109	1.83E-273	2
Ccpg1	1.95E-276	1.53355324	0.304	0.072	6.30E-272	2
Gm34084	2.25E-274	1.61523311	0.274	0.06	7.25E-270	2
Zyx	4.49E-263	1.70943415	0.454	0.173	1.45E-258	2
Zfp36	1.07E-258	1.84730498	0.503	0.208	3.44E-254	2
Notch2	2.04E-251	1.65391186	0.474	0.191	6.58E-247	2
Dgat1	1.72E-249	1.70990544	0.444	0.168	5.56E-245	2
Stx11	3.21E-237	1.48162991	0.272	0.067	1.04E-232	2

Sat1	1.17E-235	1.5338739	0.577	0.288	3.78E-231	2
2310001H17I	1.16E-229	1.64592594	0.357	0.117	3.75E-225	2
Dhrs7	3.48E-226	1.56824137	0.45	0.187	1.12E-221	2
Hcst	5.77E-222	1.54084634	0.465	0.2	1.86E-217	2
Prdx5	2.29E-215	1.0716238	0.753	0.427	7.39E-211	2
Malat11	6.77E-208	0.44533887	0.999	0.954	2.19E-203	2
Emilin2	3.65E-207	1.38570989	0.25	0.062	1.18E-202	2
Ier3	1.04E-205	1.56601388	0.369	0.131	3.35E-201	2
Nabp1	3.03E-204	1.46216601	0.26	0.068	9.77E-200	2
Cwc25	5.29E-200	1.7684002	0.292	0.088	1.71E-195	2
Fbxl5	1.93E-198	1.52634999	0.333	0.11	6.22E-194	2
Lyst	4.14E-192	1.44131234	0.323	0.11	1.34E-187	2
Pygl	1.49E-191	1.5437143	0.359	0.136	4.80E-187	2
Nfam1	1.25E-189	1.56441738	0.312	0.105	4.04E-185	2
Rabgef1	3.04E-187	1.45215309	0.268	0.079	9.80E-183	2
Coro1a	2.28E-184	1.08347298	0.683	0.419	7.35E-180	2
Gmfg	8.87E-179	1.0853812	0.641	0.387	2.86E-174	2
Emb	3.50E-177	1.39292561	0.36	0.138	1.13E-172	2
Tmcc1	2.98E-168	1.42617479	0.332	0.125	9.62E-164	2
Ctsd	4.29E-167	1.20800827	0.463	0.218	1.38E-162	2
Cd53	3.83E-165	1.40442314	0.474	0.244	1.24E-160	2
Tnfrsf1a	5.17E-161	1.4179859	0.286	0.098	1.67E-156	2
Slfn4	1.85E-160	1.53894896	0.277	0.093	5.98E-156	2
Pnrc11	2.47E-160	1.29533176	0.535	0.301	7.99E-156	2
Ier2	8.58E-153	1.38563696	0.483	0.264	2.77E-148	2
Nudt4	6.67E-152	1.36750143	0.383	0.171	2.15E-147	2
Tgfbi	2.81E-147	1.07082095	0.262	0.084	9.07E-143	2
Rassf3	2.88E-144	1.33268986	0.254	0.085	9.29E-140	2
Spi1	2.12E-143	1.33079615	0.41	0.205	6.85E-139	2
Gabarap	4.36E-142	1.04866867	0.652	0.469	1.41E-137	2
Arpc3	4.83E-140	0.99650917	0.688	0.506	1.56E-135	2
Prr13	1.08E-139	1.26886586	0.404	0.195	3.50E-135	2
Nr4a1	2.19E-138	1.4686876	0.395	0.19	7.08E-134	2
Cd91	6.55E-137	1.12791373	0.549	0.326	2.11E-132	2
Sbno1	6.61E-135	1.33972339	0.362	0.164	2.13E-130	2
Pirb	7.45E-133	1.31570569	0.337	0.149	2.41E-128	2
Rac21	9.83E-133	0.98696861	0.61	0.395	3.17E-128	2
Rab8b	1.20E-129	1.35848262	0.359	0.169	3.87E-125	2
H2-D11	6.11E-128	0.75766453	0.8	0.651	1.97E-123	2
Resf1	1.67E-123	1.33184298	0.368	0.174	5.38E-119	2
R3hdm4	2.04E-118	1.23821015	0.357	0.175	6.58E-114	2
Kdm7a	3.19E-114	1.22161519	0.285	0.119	1.03E-109	2
Atg3	2.92E-113	1.27183964	0.285	0.121	9.44E-109	2

Ifitm3	4.36E-113	0.54805222	0.693	0.449	1.41E-108	2
Card19	3.60E-112	1.38881781	0.309	0.141	1.16E-107	2
Fmnl1	2.05E-108	1.24123499	0.291	0.127	6.63E-104	2
Samhd1	3.38E-106	1.12714144	0.346	0.166	1.09E-101	2
Kdm6b	2.58E-105	1.41612086	0.333	0.162	8.34E-101	2
Ccnl1	1.40E-101	1.27895765	0.41	0.238	4.53E-97	2
Itgb2	4.56E-100	1.18519899	0.331	0.167	1.47E-95	2
D8Ertd738e	9.48E-100	1.16780533	0.56	0.416	3.06E-95	2
Tut7	4.02E-96	1.19294858	0.361	0.195	1.30E-91	2
Cd441	1.07E-90	1.07000635	0.426	0.255	3.44E-86	2
Ypel31	1.52E-86	0.88322414	0.395	0.22	4.90E-82	2
Lyn1	3.73E-85	0.96559566	0.373	0.207	1.20E-80	2
Atp6v1g11	8.82E-85	0.99029404	0.485	0.335	2.85E-80	2
Gnai2	1.06E-84	0.7900887	0.619	0.484	3.42E-80	2
Cyba	1.51E-84	0.59081714	0.74	0.515	4.87E-80	2
Adipor1	1.81E-84	1.16243392	0.323	0.172	5.84E-80	2
Gpsm3	2.58E-83	1.15683817	0.304	0.159	8.35E-79	2
Cdk11b	3.83E-82	1.20955555	0.335	0.185	1.24E-77	2
Iqgap11	8.13E-82	0.94059321	0.529	0.383	2.62E-77	2
Selenok	2.37E-81	1.0180647	0.52	0.386	7.65E-77	2
Cytip1	3.02E-76	0.89908547	0.327	0.171	9.75E-72	2
Fam32a	4.33E-70	1.09336653	0.295	0.161	1.40E-65	2
Slfn2	5.17E-69	1.06541999	0.288	0.155	1.67E-64	2
Sem1	7.92E-68	0.52333908	0.788	0.661	2.56E-63	2
Ddx51	1.04E-67	0.70325715	0.646	0.543	3.35E-63	2
Anxa21	1.10E-66	0.64978608	0.571	0.411	3.55E-62	2
Hist2h2aa1	2.06E-66	1.01600222	0.25	0.125	6.66E-62	2
Ostf1	4.20E-66	0.88920617	0.454	0.322	1.36E-61	2
Vsir	5.11E-64	1.05129493	0.253	0.132	1.65E-59	2
Rab7	6.09E-64	1.12963328	0.311	0.182	1.97E-59	2
Srsf51	1.67E-63	0.85916459	0.499	0.376	5.40E-59	2
Cdk2ap2	1.53E-62	1.32194926	0.4	0.28	4.95E-58	2
Itgam	1.63E-60	0.9709308	0.302	0.176	5.28E-56	2
Gng5	3.01E-60	0.5591241	0.753	0.647	9.71E-56	2
Lamp2	4.70E-60	1.05222494	0.343	0.213	1.52E-55	2
Myl12b	9.13E-60	0.77949897	0.531	0.417	2.95E-55	2
Cotl1	2.05E-58	0.97701573	0.336	0.213	6.63E-54	2
Klf6	9.60E-57	1.01319234	0.357	0.228	3.10E-52	2
Clk11	8.19E-52	0.86849335	0.396	0.278	2.64E-47	2
Skil	8.19E-51	0.96115384	0.299	0.181	2.64E-46	2
Syk1	9.06E-51	0.82549723	0.3	0.178	2.92E-46	2
Ddx3x	4.81E-50	1.03005594	0.336	0.22	1.55E-45	2
Skap2	4.95E-49	0.99730278	0.252	0.146	1.60E-44	2

Atp6v1e1	9.45E-48	0.81148762	0.451	0.344	3.05E-43	2
Vasp	3.54E-47	0.8923736	0.3	0.19	1.14E-42	2
Ubc	3.24E-45	0.81334621	0.504	0.413	1.05E-40	2
Ppp1r18	8.54E-45	0.9549587	0.266	0.162	2.76E-40	2
Sdcbp	9.84E-45	0.91834839	0.326	0.22	3.18E-40	2
Rhog	9.51E-44	0.98117869	0.268	0.168	3.07E-39	2
Neat11	1.72E-41	0.76486251	0.321	0.211	5.55E-37	2
Pgd	7.91E-39	0.85337509	0.262	0.168	2.55E-34	2
Emd	1.34E-36	1.02159153	0.254	0.165	4.31E-32	2
Ncf1	8.54E-35	0.87120085	0.266	0.177	2.76E-30	2
Myh9	5.72E-34	0.69085483	0.431	0.349	1.85E-29	2
Actn11	2.49E-33	0.66463139	0.293	0.196	8.04E-29	2
Sh3bgrl3	3.28E-32	0.38161413	0.679	0.565	1.06E-27	2
Gadd45a	6.50E-32	0.8966133	0.266	0.18	2.10E-27	2
Cox17	1.05E-29	0.81449011	0.498	0.452	3.39E-25	2
Cap1	1.46E-29	0.73896085	0.289	0.205	4.71E-25	2
Bcl10	2.55E-29	0.81026505	0.264	0.182	8.22E-25	2
Hif1a	8.82E-29	0.66531006	0.258	0.169	2.85E-24	2
Timp21	3.63E-26	0.48406637	0.258	0.168	1.17E-21	2
Dazap2	2.89E-25	0.78247155	0.287	0.213	9.32E-21	2
Akap131	4.18E-25	0.65753112	0.323	0.243	1.35E-20	2
Itm2b	7.66E-25	0.40581906	0.59	0.535	2.47E-20	2
Pet100	9.91E-25	0.65398719	0.41	0.353	3.20E-20	2
Laptm5	1.85E-24	0.64754795	0.345	0.271	5.97E-20	2
Grk21	1.05E-23	0.65854232	0.339	0.27	3.40E-19	2
H2afj	7.76E-23	0.55182041	0.488	0.431	2.51E-18	2
Cdc42	2.66E-22	0.47795807	0.524	0.485	8.60E-18	2
Nfkbia1	2.74E-22	0.8679371	0.308	0.237	8.86E-18	2
Arpc5	5.14E-22	0.55346868	0.409	0.35	1.66E-17	2
Ube2b1	1.68E-21	0.62175685	0.351	0.286	5.41E-17	2
Rap1b	2.46E-21	0.71714172	0.317	0.253	7.93E-17	2
Arpc2	3.53E-21	0.49295398	0.493	0.447	1.14E-16	2
Lbr	5.19E-20	0.64237661	0.313	0.254	1.68E-15	2
Mgst1	7.74E-20	0.69898488	0.295	0.232	2.50E-15	2
Pten	7.48E-19	0.55357165	0.259	0.191	2.41E-14	2
Arpc1b	2.41E-18	0.48326938	0.48	0.439	7.79E-14	2
Ifi27l2a1	3.30E-16	0.71171364	0.266	0.197	1.07E-11	2
Timm10b1	7.89E-15	0.70628851	0.303	0.26	2.55E-10	2
Marcks1	5.47E-14	0.49665885	0.258	0.196	1.77E-09	2
Dnaja11	1.51E-13	0.60904068	0.384	0.35	4.88E-09	2
Capza1	1.66E-13	0.63671627	0.279	0.237	5.37E-09	2
Actr3	4.69E-13	0.49540276	0.379	0.346	1.51E-08	2
Tpm41	8.37E-12	0.34806806	0.313	0.253	2.70E-07	2

Atp6v0b1	4.92E-08	0.54221921	0.305	0.285	0.00159003	2
Supt4a1	6.04E-07	0.48858674	0.3	0.284	0.01948872	2
Tmbim6	1.95E-06	0.4055469	0.315	0.299	0.06292942	2
Aldoa1	1.65E-05	0.26006676	0.427	0.423	0.53227915	2
Rab5if	2.67E-05	0.46884789	0.279	0.271	0.86212791	2
Chmp4b	6.32E-05	0.50589419	0.272	0.262	1	2
Sf3b11	8.22E-05	0.49669225	0.279	0.269	1	2
Prdx6	0.00066684	0.49583692	0.285	0.287	1	2
Ube2d3	0.00081224	0.36084527	0.356	0.365	1	2
Fam49b	0.00379657	0.41406528	0.253	0.252	1	2
Gpi1	0.00698344	0.32217488	0.285	0.289	1	2
Zfp36l2	0.00775937	0.32424696	0.27	0.263	1	2
Ltf	0	3.818803	0.992	0.111	0	3
Ngp	0	3.28907383	0.997	0.226	0	3
Lcn2	0	2.96894102	0.996	0.247	0	3
Camp	0	2.91059137	0.997	0.195	0	3
Ifitm6	0	2.73022475	0.983	0.198	0	3
Wfdc211	0	2.65954424	0.996	0.234	0	3
S100a81	0	2.43838011	0.998	0.465	0	3
S100a91	0	2.39599378	0.998	0.446	0	3
Mmp81	0	2.33923433	0.89	0.146	0	3
Anxa1	0	2.18965323	0.974	0.34	0	3
Pglyrp11	0	2.15997896	0.984	0.224	0	3
Ly6g	0	2.13264568	0.767	0.09	0	3
Adpgk	0	2.10807289	0.87	0.149	0	3
Chil3	0	1.99722936	0.876	0.125	0	3
Mmp91	0	1.99462989	0.953	0.165	0	3
Cd177	0	1.9737085	0.803	0.118	0	3
Cybb	0	1.90524336	0.889	0.188	0	3
Prdx51	0	1.75131805	0.988	0.411	0	3
AA467197	0	1.64713198	0.698	0.085	0	3
Krt83	0	1.60652511	0.485	0.051	0	3
G0s21	0	1.59222193	0.884	0.189	0	3
Plbd1	0	1.56092847	0.73	0.122	0	3
Retnlg1	0	1.53534276	0.954	0.219	0	3
Samsn11	0	1.53115473	0.894	0.265	0	3
Syne1	0	1.46680633	0.564	0.079	0	3
Plaur1	0	1.4634454	0.91	0.276	0	3
Itgam1	0	1.44028057	0.73	0.143	0	3
Hp1	0	1.43452752	0.917	0.262	0	3
Mcemp11	0	1.43343768	0.789	0.175	0	3
Fcer1g1	0	1.4119728	0.986	0.355	0	3
Lyz2	0	1.40059104	0.996	0.358	0	3

Thbs11	0	1.39829171	0.743	0.177	0	3
Lrg11	0	1.38044101	0.717	0.134	0	3
Mrgpra2b	0	1.37848105	0.505	0.073	0	3
Arhgdib	0	1.37130302	0.965	0.398	0	3
Itgb2l	0	1.37033961	0.511	0.061	0	3
Ncf11	0	1.35529368	0.685	0.144	0	3
Dstn1	0	1.35042298	0.929	0.368	0	3
Dgat11	0	1.34688001	0.696	0.15	0	3
Ltb4r1	0	1.30794925	0.522	0.085	0	3
Fpr2	0	1.2693695	0.515	0.074	0	3
Alox5ap1	0	1.26861523	0.899	0.283	0	3
Trem3	0	1.18934085	0.566	0.107	0	3
Pirb1	0	1.18842815	0.603	0.129	0	3
Clec4a2	0	1.16201295	0.628	0.138	0	3
Slfn41	0	1.14739105	0.483	0.077	0	3
Slpi1	0	1.14686145	0.857	0.243	0	3
Plscr1	0	1.14371002	0.426	0.079	0	3
Serpinb1a	0	1.13499791	0.54	0.122	0	3
Cpne3	0	1.13157612	0.567	0.129	0	3
Dhrs71	0	1.12670056	0.662	0.172	0	3
Cyba1	0	1.1185995	0.969	0.498	0	3
Clec4e1	0	1.10479754	0.629	0.137	0	3
Gadd45a1	0	1.09762459	0.583	0.155	0	3
Ckap4	0	1.09622143	0.633	0.164	0	3
Mgst11	0	1.08954109	0.715	0.2	0	3
Ly6c2	0	1.08062049	0.781	0.187	0	3
Cd521	0	1.0765097	0.99	0.482	0	3
Hdc1	0	1.07273286	0.721	0.161	0	3
Chil1	0	1.06971977	0.434	0.07	0	3
Atp6v1e11	0	1.01708176	0.802	0.317	0	3
Pygl1	0	1.00582524	0.56	0.122	0	3
B230208H11	0	0.99002291	0.352	0.052	0	3
Ldhc	0	0.9788828	0.264	0.023	0	3
Hcst1	0	0.97847983	0.664	0.186	0	3
Adam81	0	0.97446464	0.628	0.126	0	3
Actb1	0	0.96635186	0.999	0.946	0	3
Lilr4b1	0	0.9642069	0.713	0.167	0	3
Degs1	0	0.94868237	0.551	0.141	0	3
Lta4h	0	0.94758285	0.453	0.12	0	3
Rflnb	0	0.94158499	0.404	0.082	0	3
Vasp1	0	0.93858028	0.602	0.166	0	3
Padi4	0	0.93687029	0.375	0.06	0	3
Mxd11	0	0.92742491	0.797	0.216	0	3

Ostf11	0	0.92555173	0.772	0.297	0	3
St3gal5	0	0.92235212	0.432	0.097	0	3
Aldh2	0	0.91917405	0.675	0.24	0	3
Rac22	0	0.90985116	0.892	0.374	0	3
Acpp	0	0.89809364	0.283	0.032	0	3
Lcp11	0	0.89140987	0.817	0.286	0	3
Rgs18	0	0.88688635	0.429	0.102	0	3
Fosl1	0	0.88674296	0.365	0.064	0	3
Glrx	0	0.88205562	0.468	0.121	0	3
R3hdm41	0	0.87928983	0.578	0.158	0	3
Arrb2	0	0.87905524	0.409	0.081	0	3
Ncf21	0	0.87902499	0.697	0.203	0	3
Itgb21	0	0.87898273	0.564	0.149	0	3
Cdk11b1	0	0.87528429	0.576	0.167	0	3
1700047M11	0	0.87083945	0.264	0.025	0	3
Gca	0	0.86937821	0.303	0.042	0	3
C5ar11	0	0.85495291	0.48	0.099	0	3
Pnkp	0	0.8367721	0.399	0.094	0	3
Bmx	0	0.83497057	0.367	0.061	0	3
Tyrobp1	0	0.82599884	0.982	0.371	0	3
Mapk13	0	0.81698679	0.307	0.044	0	3
Tmsb4x1	0	0.81380176	0.998	0.898	0	3
Trib11	0	0.8037388	0.514	0.133	0	3
Hk3	0	0.8019678	0.344	0.074	0	3
Samd9l	0	0.79594051	0.464	0.11	0	3
S100a112	0	0.78453728	0.993	0.662	0	3
Gdpd3	0	0.78447552	0.321	0.061	0	3
Ceacam10	0	0.77613645	0.262	0.032	0	3
Mmp25	0	0.75891378	0.27	0.035	0	3
Nhsl2	0	0.7442544	0.272	0.045	0	3
Fmo5	0	0.73125898	0.261	0.039	0	3
Lst11	0	0.71914799	0.748	0.231	0	3
Pilra1	0	0.71855782	0.449	0.104	0	3
C3	0	0.7118218	0.474	0.123	0	3
Ceacam1	0	0.71099684	0.282	0.05	0	3
Slc2a31	0	0.67800534	0.371	0.08	0	3
Il1r21	0	0.61412969	0.611	0.152	0	3
Slc16a31	0	0.59440867	0.515	0.132	0	3
Msrb11	0	0.54954061	0.804	0.287	0	3
Trem11	0	0.53012572	0.567	0.148	0	3
Lilrb4a1	1.92E-307	0.69680002	0.528	0.15	6.21E-303	3
Mgst2	2.63E-303	0.86334606	0.433	0.112	8.51E-299	3
Cdadc1	5.36E-300	0.76391473	0.282	0.051	1.73E-295	3

Vsir1	4.77E-297	0.76979121	0.445	0.118	1.54E-292	3
Glipr2	5.34E-296	0.77425218	0.386	0.093	1.72E-291	3
Gpsm31	7.17E-296	0.7475984	0.499	0.145	2.31E-291	3
G6pdx	2.67E-294	0.80877292	0.346	0.078	8.62E-290	3
Ncf4	4.88E-294	0.788375	0.5	0.147	1.58E-289	3
Pilrb2	2.70E-293	0.68832778	0.294	0.056	8.72E-289	3
Hsd11b1	6.05E-292	0.71904565	0.299	0.059	1.95E-287	3
Tmcc11	2.31E-291	0.762527	0.445	0.117	7.44E-287	3
Cd331	2.05E-290	0.62820158	0.343	0.072	6.62E-286	3
Prdx61	5.12E-289	0.90370597	0.682	0.255	1.65E-284	3
F630028O10	1.19E-288	0.88002794	0.364	0.085	3.83E-284	3
Gsr1	2.61E-287	0.6658133	0.751	0.279	8.42E-283	3
Mettl9	8.49E-287	0.78667514	0.442	0.122	2.74E-282	3
Tkt	5.45E-284	0.87842369	0.7	0.269	1.76E-279	3
Ifitm31	1.10E-281	0.56510647	0.93	0.432	3.54E-277	3
Grina1	1.97E-281	0.52675473	0.763	0.274	6.35E-277	3
Slc7a111	2.47E-280	0.51151028	0.433	0.109	7.96E-276	3
Fos1	9.39E-280	0.4983318	0.836	0.326	3.03E-275	3
Rasa2	4.07E-277	0.76796066	0.357	0.084	1.31E-272	3
Xdh	8.98E-267	0.71447	0.364	0.089	2.90E-262	3
Lgals31	2.33E-266	0.55272	0.885	0.359	7.54E-262	3
Sp140	1.19E-264	0.67571554	0.315	0.069	3.85E-260	3
Sorl11	1.20E-264	0.57047696	0.452	0.127	3.88E-260	3
Rasgrp4	1.58E-260	0.63627633	0.277	0.054	5.10E-256	3
Pram1	1.01E-259	0.64734889	0.252	0.046	3.28E-255	3
Nfam11	5.51E-259	0.63174458	0.388	0.101	1.78E-254	3
Cebpe	4.62E-256	0.71234775	0.282	0.059	1.49E-251	3
Flna1	5.57E-246	0.7169297	0.722	0.29	1.80E-241	3
Cd141	1.69E-238	0.56983348	0.502	0.161	5.47E-234	3
Coro1a1	3.53E-238	0.70602265	0.889	0.405	1.14E-233	3
Osm1	8.06E-237	0.59720543	0.364	0.095	2.60E-232	3
Tcp11l2	3.70E-236	0.57517837	0.303	0.069	1.19E-231	3
Pira2	5.51E-233	0.58712807	0.287	0.063	1.78E-228	3
Pdcd6	7.17E-232	0.82414231	0.485	0.165	2.31E-227	3
Clec12a	1.10E-231	0.6597172	0.409	0.12	3.54E-227	3
Lamtor4	6.88E-228	0.71581695	0.649	0.254	2.22E-223	3
Actn12	1.28E-226	0.53961931	0.538	0.177	4.14E-222	3
Cotl11	2.88E-226	0.57732274	0.552	0.196	9.29E-222	3
Anxa11	4.02E-226	0.63377538	0.343	0.089	1.30E-221	3
Ets21	1.10E-222	0.49249471	0.47	0.147	3.56E-218	3
Kctd121	1.54E-222	0.50908828	0.505	0.167	4.97E-218	3
Prr131	1.23E-220	0.58039235	0.538	0.186	3.97E-216	3
Cd300lf1	1.50E-219	0.49753278	0.335	0.085	4.84E-215	3

Mpc21	5.72E-219	0.65943279	0.589	0.231	1.85E-214	3
Cd92	1.18E-217	0.53867542	0.761	0.311	3.80E-213	3
Suco	5.14E-212	0.62503449	0.353	0.098	1.66E-207	3
Lmnb11	1.92E-207	0.46350402	0.665	0.269	6.19E-203	3
Hck	2.81E-206	0.53450828	0.304	0.078	9.08E-202	3
Litaf1	2.00E-204	0.44074812	0.625	0.24	6.45E-200	3
Cd531	7.22E-204	0.45950327	0.609	0.234	2.33E-199	3
Abhd5	1.07E-203	0.68581318	0.277	0.069	3.44E-199	3
Gmfg1	1.90E-198	0.53092907	0.828	0.374	6.12E-194	3
Sun2	1.99E-192	0.59678179	0.327	0.093	6.42E-188	3
Pgd1	4.42E-188	0.57940059	0.443	0.154	1.43E-183	3
Capg	1.08E-187	0.4983856	0.482	0.169	3.50E-183	3
Msra	4.04E-180	0.5556485	0.303	0.086	1.30E-175	3
Stap1	9.19E-180	0.5287063	0.262	0.067	2.97E-175	3
Cnn2	7.90E-179	0.51699428	0.408	0.137	2.55E-174	3
Lims1	2.15E-178	0.52689848	0.359	0.113	6.93E-174	3
Nr4a11	3.90E-178	0.4834132	0.494	0.183	1.26E-173	3
Actr31	7.73E-178	0.56573692	0.705	0.32	2.50E-173	3
Spi11	5.07E-176	0.50159809	0.514	0.198	1.64E-171	3
Cebpd	5.30E-176	0.59618703	0.361	0.117	1.71E-171	3
Nabp11	5.12E-175	0.46426549	0.27	0.069	1.65E-170	3
Sem11	1.82E-173	0.59517688	0.981	0.646	5.87E-169	3
Lasp1	8.05E-172	0.50931397	0.386	0.128	2.60E-167	3
Cpne2	2.77E-167	0.52699849	0.265	0.072	8.95E-163	3
Gpi11	8.50E-167	0.54016767	0.611	0.263	2.75E-162	3
Cyb5r4	3.45E-165	0.4797881	0.282	0.079	1.11E-160	3
Cyfip2	2.46E-164	0.43213236	0.388	0.133	7.95E-160	3
Selenok1	2.42E-163	0.54387656	0.756	0.368	7.81E-159	3
Sh3bgrl31	8.55E-160	0.5552635	0.946	0.544	2.76E-155	3
Sri	9.79E-158	0.52705053	0.628	0.286	3.16E-153	3
AB124611	3.47E-155	0.44213217	0.313	0.097	1.12E-150	3
Unc119	1.28E-154	0.46852954	0.289	0.087	4.15E-150	3
Stx111	7.54E-153	0.39842834	0.256	0.069	2.43E-148	3
Zyx1	6.33E-152	0.41921834	0.455	0.174	2.04E-147	3
Myo1f	3.41E-151	0.48659707	0.264	0.076	1.10E-146	3
Lmo41	1.25E-150	0.37397459	0.565	0.239	4.04E-146	3
Ptprc1	8.89E-150	0.31213459	0.59	0.25	2.87E-145	3
Myl6	1.12E-149	0.52829611	0.97	0.687	3.62E-145	3
Wdr1	1.50E-149	0.45690964	0.425	0.162	4.83E-145	3
Gadd45g	3.16E-149	0.46993287	0.264	0.075	1.02E-144	3
Scp2	1.94E-148	0.49646015	0.496	0.206	6.26E-144	3
Cap11	3.40E-141	0.40585585	0.476	0.191	1.10E-136	3
Tecr	5.80E-140	0.4668128	0.503	0.214	1.87E-135	3

Arpc51	1.06E-139	0.4508285	0.683	0.329	3.41E-135	3
Rab8b1	1.27E-138	0.41945727	0.425	0.165	4.09E-134	3
Ndel11	8.88E-138	0.31164903	0.372	0.132	2.87E-133	3
Rhog1	2.36E-137	0.47569811	0.404	0.158	7.63E-133	3
Arpc31	1.60E-136	0.48859115	0.88	0.492	5.17E-132	3
Arpc41	1.54E-135	0.40951233	0.551	0.246	4.96E-131	3
Pfn1	2.28E-135	0.48958506	0.987	0.733	7.37E-131	3
Plp2	9.47E-134	0.44839349	0.383	0.145	3.06E-129	3
4932438A13	2.03E-133	0.3986265	0.361	0.13	6.56E-129	3
Flot1	3.35E-132	0.41382663	0.299	0.099	1.08E-127	3
Ppp1r181	3.30E-131	0.39077436	0.397	0.152	1.07E-126	3
Phlda1	2.56E-127	0.39303243	0.306	0.103	8.26E-123	3
Rabgef11	2.85E-127	0.35708113	0.26	0.08	9.22E-123	3
Tgoln1	2.24E-125	0.36191833	0.33	0.117	7.24E-121	3
Mindy1	1.37E-124	0.39167548	0.309	0.107	4.43E-120	3
Fosl21	1.93E-124	0.37386929	0.367	0.138	6.22E-120	3
Svil	4.16E-124	0.34069033	0.291	0.097	1.34E-119	3
Slfn21	1.52E-123	0.36088406	0.383	0.148	4.91E-119	3
Myh91	2.00E-120	0.37562799	0.669	0.33	6.44E-116	3
Magoh	7.27E-119	0.47472747	0.528	0.246	2.35E-114	3
Csf2ra	2.14E-118	0.37006152	0.283	0.098	6.90E-114	3
Efhd2	8.61E-118	0.35442099	0.376	0.151	2.78E-113	3
Taldo11	4.62E-116	0.31994014	0.732	0.37	1.49E-111	3
Rab71	1.34E-114	0.33117441	0.416	0.174	4.34E-110	3
Ier21	1.59E-112	0.27224936	0.554	0.26	5.12E-108	3
Nfkbiz1	1.86E-112	0.38558936	0.326	0.122	6.00E-108	3
Mfsd14b	7.12E-112	0.41000103	0.263	0.091	2.30E-107	3
Myl12b1	3.49E-111	0.36005477	0.766	0.399	1.13E-106	3
Stxbp2	2.07E-109	0.38795135	0.252	0.085	6.68E-105	3
2310001H17	7.58E-109	0.25119432	0.323	0.121	2.45E-104	3
Lyst1	2.28E-108	0.34626196	0.303	0.112	7.36E-104	3
Golim4	1.31E-106	0.27016646	0.291	0.104	4.22E-102	3
Hcls1	7.44E-106	0.31581136	0.338	0.134	2.40E-101	3
Actg11	3.82E-105	0.34273282	0.977	0.696	1.23E-100	3
Selenon1	1.46E-104	0.25189245	0.25	0.082	4.72E-100	3
Cklf	1.62E-101	0.34728348	0.26	0.094	5.23E-97	3
Capza11	1.30E-99	0.30201581	0.474	0.222	4.19E-95	3
Rab6a	2.63E-99	0.29011951	0.308	0.118	8.48E-95	3
Ppp2r5a	8.89E-99	0.29828574	0.421	0.188	2.87E-94	3
Tut71	2.13E-98	0.2633896	0.429	0.191	6.88E-94	3
Nadk	9.92E-97	0.34008012	0.274	0.102	3.20E-92	3
Cwc251	8.17E-94	0.32386152	0.254	0.092	2.64E-89	3
Rab5if1	3.17E-92	0.31385303	0.516	0.252	1.02E-87	3

Rnf130	2.33E-84	0.29748978	0.293	0.123	7.53E-80	3
Atxn10	1.87E-83	0.29058349	0.303	0.127	6.04E-79	3
1110008P14I	4.29E-76	0.25087993	0.304	0.133	1.38E-71	3
Txn12	5.28E-71	0.28297566	0.944	0.612	1.71E-66	3
Ptpre	1.62E-69	0.2712869	0.251	0.106	5.24E-65	3
Gapdh1	9.43E-69	0.26339707	0.869	0.523	3.04E-64	3
Camp1	0	4.10034646	1	0.21	0	4
Chil31	0	3.56312719	0.994	0.132	0	4
Ngp1	0	3.40414533	0.999	0.24	0	4
Lcn21	0	2.93443984	1	0.261	0	4
Ltf1	0	2.62283962	0.99	0.127	0	4
S100a82	0	2.40504729	1	0.475	0	4
Wfdc212	0	2.33258949	0.999	0.248	0	4
S100a92	0	2.18991638	1	0.456	0	4
Hmgn2	0	2.18115656	0.963	0.34	0	4
Anxa12	0	2.08406466	0.987	0.35	0	4
Pglyrp12	0	1.9353174	0.987	0.238	0	4
Fcnb	0	1.8800604	0.606	0.02	0	4
Orm1	0	1.79427502	0.643	0.019	0	4
Ifitm61	0	1.76016364	0.936	0.215	0	4
Arhgdib1	0	1.69753827	0.992	0.406	0	4
Serpinb1a1	0	1.64708718	0.784	0.115	0	4
Cybb1	0	1.64529129	0.927	0.198	0	4
Lyz21	0	1.61711304	0.994	0.369	0	4
Cebpe1	0	1.60209133	0.647	0.041	0	4
Cd1771	0	1.53302488	0.846	0.128	0	4
Prdx52	0	1.43912961	0.986	0.421	0	4
Lta4h1	0	1.39320068	0.724	0.11	0	4
Adpgk1	0	1.38896533	0.845	0.164	0	4
Clec4a21	0	1.30927214	0.765	0.139	0	4
Cyba2	0	1.27917982	0.99	0.506	0	4
Hp2	0	1.27837299	0.957	0.271	0	4
F630028O10	0	1.2783495	0.557	0.079	0	4
Ly6g1	0	1.24077313	0.618	0.111	0	4
Ltb4r11	0	1.21469685	0.643	0.086	0	4
Trem31	0	1.20217759	0.687	0.109	0	4
Mgst21	0	1.14211186	0.643	0.105	0	4
Dstn2	0	1.1112749	0.951	0.377	0	4
Cpne31	0	1.03824516	0.677	0.13	0	4
C31	0	1.02011365	0.687	0.116	0	4
Syne11	0	1.01328476	0.583	0.086	0	4
Gca1	0	0.98672874	0.454	0.038	0	4
Lamtor41	0	0.97343024	0.824	0.25	0	4

Ncf41	0	0.95820177	0.67	0.143	0	4
AA4671971	0	0.94306673	0.61	0.102	0	4
Prdx62	0	0.94145364	0.815	0.255	0	4
Itgb2l1	0	0.93748693	0.526	0.068	0	4
Scp21	0	0.93652692	0.736	0.197	0	4
Abhd51	0	0.92403245	0.507	0.059	0	4
B230208H11	0	0.91219824	0.459	0.051	0	4
Cebpd1	0	0.88224237	0.58	0.109	0	4
Clec12a1	0	0.86459066	0.586	0.114	0	4
Rflnb1	0	0.8589107	0.523	0.081	0	4
Ckap41	0	0.85429295	0.693	0.169	0	4
Ly6c21	0	0.85338431	0.839	0.194	0	4
Zmpste24	0	0.84073788	0.405	0.064	0	4
Plscr11	0	0.82568394	0.464	0.083	0	4
Chil11	0	0.82374315	0.491	0.073	0	4
Agpat2	0	0.8033069	0.431	0.047	0	4
Krt831	0	0.79544209	0.416	0.063	0	4
Ncf12	0	0.78624692	0.675	0.154	0	4
Plbd11	0	0.77452593	0.615	0.14	0	4
St3gal51	0	0.76841667	0.506	0.099	0	4
Plaur2	0	0.71404698	0.892	0.289	0	4
1700020L24I	0	0.7034908	0.316	0.025	0	4
Lrg12	0	0.68902131	0.698	0.145	0	4
Mcemp12	0	0.68792075	0.737	0.189	0	4
Hsd11b11	0	0.66376345	0.378	0.058	0	4
Ceacam11	0	0.64238591	0.354	0.05	0	4
Ldhc1	0	0.63690659	0.255	0.028	0	4
Mapk131	0	0.6256299	0.329	0.047	0	4
Glrx1	0	0.6249154	0.536	0.123	0	4
Pnkp1	0	0.61366837	0.468	0.096	0	4
Megf9	0	0.58868557	0.285	0.036	0	4
Itgam2	0	0.58333679	0.624	0.16	0	4
Stk39	0	0.56919545	0.281	0.03	0	4
Ms4a3	0	0.5664439	0.297	0.027	0	4
Alox5ap2	8.29E-308	0.69216397	0.909	0.293	2.68E-303	4
Ncam1	2.38E-306	0.39652109	0.321	0.051	7.69E-302	4
G0s22	1.82E-305	0.28685562	0.745	0.209	5.86E-301	4
Arrb21	1.69E-302	0.56039836	0.424	0.086	5.46E-298	4
Pirb2	2.93E-301	0.67152118	0.563	0.14	9.47E-297	4
Pilrb21	5.64E-297	0.50805162	0.337	0.058	1.82E-292	4
Tkt1	1.22E-295	0.80151474	0.809	0.271	3.93E-291	4
Dhrs72	2.46E-295	0.56450492	0.659	0.181	7.95E-291	4
Gpsm32	2.35E-292	0.56471707	0.57	0.147	7.58E-288	4

Ero1l	1.16E-290	0.45673443	0.346	0.062	3.76E-286	4
Acsl1	1.72E-290	0.57541736	0.343	0.062	5.55E-286	4
Igsf6	3.27E-288	0.53806893	0.348	0.064	1.06E-283	4
Rgs181	1.52E-285	0.58412773	0.463	0.106	4.92E-281	4
Gadd45g1	6.33E-285	0.50850052	0.375	0.072	2.04E-280	4
Tecr1	5.41E-275	0.65905039	0.683	0.209	1.75E-270	4
Nhsl21	7.23E-274	0.53995705	0.291	0.048	2.33E-269	4
1810037I17R	1.65E-270	0.93740863	0.84	0.324	5.33E-266	4
Mrgpra2b1	2.25E-268	0.53579707	0.406	0.087	7.26E-264	4
Dgat12	6.90E-268	0.41116216	0.61	0.165	2.23E-263	4
Hcst2	1.81E-266	0.45443878	0.665	0.194	5.84E-262	4
Fcer1g2	1.62E-263	0.76511398	0.976	0.367	5.23E-259	4
Tuba4a	3.02E-261	0.54955764	0.461	0.112	9.75E-257	4
Acpp1	2.06E-259	0.50725024	0.255	0.038	6.65E-255	4
Gadd45a2	3.25E-256	0.60529001	0.579	0.163	1.05E-251	4
Mettl91	1.50E-252	0.52804127	0.488	0.125	4.84E-248	4
Dgat2	1.21E-244	0.42684364	0.274	0.046	3.91E-240	4
Aldh21	4.30E-241	0.6107459	0.731	0.244	1.39E-236	4
Mgst12	3.04E-238	0.5262201	0.676	0.211	9.81E-234	4
Degs11	1.27E-235	0.52081461	0.535	0.15	4.11E-231	4
Ap3s1	1.68E-234	0.49678693	0.511	0.139	5.42E-230	4
Rac23	1.49E-232	0.7182294	0.92	0.382	4.81E-228	4
G6pdx1	5.73E-231	0.45418507	0.365	0.081	1.85E-226	4
H2afz	2.02E-226	0.85108195	0.971	0.554	6.53E-222	4
Emp3	7.19E-226	0.62145224	0.804	0.297	2.32E-221	4
Cdkn3	1.56E-225	0.43691302	0.287	0.055	5.02E-221	4
Tmed3	2.09E-225	0.40922308	0.376	0.087	6.74E-221	4
Samsn12	3.44E-225	0.41339871	0.804	0.281	1.11E-220	4
Cited2	8.86E-225	0.44717699	0.411	0.102	2.86E-220	4
Ndufv3	1.92E-221	0.54083263	0.685	0.23	6.20E-217	4
Gpi12	2.64E-221	0.53910071	0.751	0.261	8.52E-217	4
1110008P14I	6.82E-216	0.39509527	0.467	0.127	2.20E-211	4
Hk31	2.85E-214	0.45983203	0.345	0.078	9.19E-210	4
Itgb22	6.54E-205	0.35003406	0.537	0.158	2.11E-200	4
Rinl	1.19E-203	0.37551433	0.25	0.046	3.84E-199	4
Pygl2	1.49E-203	0.38540707	0.479	0.134	4.82E-199	4
Pram11	1.56E-203	0.40726395	0.261	0.049	5.02E-199	4
Rgcc	2.77E-203	0.33259299	0.428	0.113	8.93E-199	4
Vamp5	1.69E-201	0.31902419	0.365	0.088	5.46E-197	4
Sun21	1.07E-198	0.37130164	0.377	0.095	3.46E-194	4
Mpc22	3.95E-197	0.4471405	0.667	0.232	1.28E-192	4
Rnaseh2c	4.00E-191	0.48910395	0.615	0.211	1.29E-186	4
Ppm1m	3.39E-189	0.3504229	0.258	0.052	1.09E-184	4

Tmx4	5.04E-189	0.34134582	0.308	0.07	1.63E-184	4
Spi12	9.44E-188	0.30949687	0.604	0.199	3.05E-183	4
Hmgb2	3.26E-187	0.77643738	0.971	0.624	1.05E-182	4
Ethe1	4.39E-187	0.39816343	0.434	0.124	1.42E-182	4
Aprt1	3.76E-183	0.44875515	0.724	0.268	1.21E-178	4
Msra1	3.88E-180	0.36054443	0.345	0.087	1.25E-175	4
Glipr21	5.70E-177	0.31965382	0.373	0.099	1.84E-172	4
Sp1401	9.28E-177	0.33116317	0.31	0.074	3.00E-172	4
Hck1	7.88E-176	0.26645338	0.329	0.081	2.54E-171	4
Mcee	2.49E-173	0.39671045	0.301	0.073	8.03E-169	4
Atp6v1e12	2.52E-173	0.52861431	0.795	0.326	8.15E-169	4
Lmo42	9.10E-173	0.27962957	0.666	0.238	2.94E-168	4
Vsir2	1.41E-172	0.3235253	0.428	0.125	4.54E-168	4
Rbfa	3.35E-170	0.3813416	0.297	0.072	1.08E-165	4
1810058I24R	5.43E-170	0.26025599	0.639	0.23	1.75E-165	4
Ostf12	2.54E-169	0.42425113	0.784	0.305	8.21E-165	4
Cd93	4.10E-169	0.32740739	0.817	0.315	1.32E-164	4
Hacd2	2.09E-167	0.26795671	0.261	0.058	6.75E-163	4
Kcnab2	3.08E-166	0.37072343	0.255	0.057	9.93E-162	4
Tmsb4x2	3.11E-164	0.63666896	0.999	0.9	1.01E-159	4
Slfn42	3.41E-163	0.33606589	0.347	0.093	1.10E-158	4
Sh3bgrl32	1.37E-161	0.6302793	0.975	0.55	4.41E-157	4
Nfu1	1.97E-160	0.31335269	0.359	0.101	6.35E-156	4
Vasp2	4.59E-158	0.27121626	0.534	0.178	1.48E-153	4
Cklf1	9.31E-158	0.32777187	0.339	0.092	3.00E-153	4
Xdh1	1.74E-157	0.32541786	0.347	0.095	5.63E-153	4
Cpne21	1.45E-156	0.29980025	0.294	0.073	4.68E-152	4
Plp21	1.14E-154	0.30854896	0.456	0.145	3.69E-150	4
Sqor	2.49E-154	0.26525607	0.272	0.065	8.04E-150	4
Rps6ka1	4.46E-152	0.29453619	0.252	0.059	1.44E-147	4
Magoh1	7.60E-150	0.36460133	0.644	0.244	2.45E-145	4
Ak2	4.76E-148	0.29462209	0.393	0.121	1.54E-143	4
Nin	7.02E-147	0.33851694	0.289	0.075	2.27E-142	4
Arpc42	1.06E-145	0.31059532	0.643	0.246	3.42E-141	4
Flna2	5.23E-140	0.31225171	0.738	0.297	1.69E-135	4
Npepps	2.77E-139	0.26108557	0.293	0.079	8.94E-135	4
Padi41	4.06E-138	0.28265193	0.277	0.071	1.31E-133	4
Fpr21	1.79E-135	0.33015571	0.324	0.093	5.79E-131	4
Pgp	1.11E-133	0.29084977	0.298	0.084	3.57E-129	4
Twf2	8.69E-133	0.27697255	0.322	0.095	2.80E-128	4
Atp6v0c	3.51E-127	0.30399194	0.834	0.374	1.13E-122	4
Gmfg2	1.19E-123	0.29074253	0.857	0.38	3.84E-119	4
Cd522	5.67E-119	0.3894316	0.977	0.492	1.83E-114	4

Actb2	1.85E-115	0.5228482	1	0.946	5.97E-111	4
Ndufb7	3.06E-115	0.30842897	0.803	0.359	9.89E-111	4
Cks2	3.16E-114	0.3682341	0.578	0.242	1.02E-109	4
Coro1a2	5.03E-113	0.29833153	0.903	0.413	1.62E-108	4
Sem12	4.87E-105	0.48506204	0.981	0.652	1.57E-100	4
Pfn11	1.72E-97	0.44655415	0.992	0.737	5.54E-93	4
Retnlg2	0	3.54065772	0.997	0.231	0	5
Mmp82	0	2.94140112	0.97	0.156	0	5
Mmp92	0	2.51602539	0.945	0.181	0	5
Thbs12	0	2.29154984	0.774	0.187	0	5
S100a93	0	2.27405471	0.998	0.457	0	5
G0s23	0	2.26718742	0.876	0.203	0	5
S100a83	0	2.16843186	0.999	0.476	0	5
Slpi2	0	2.04577704	0.881	0.253	0	5
Wfdc213	0	1.98545075	0.992	0.249	0	5
Pglyrp13	0	1.96255653	0.949	0.241	0	5
Ifitm62	0	1.93316973	0.857	0.221	0	5
Slc16a32	0	1.81065268	0.665	0.131	0	5
Adam82	0	1.80192411	0.693	0.132	0	5
Samsn13	0	1.7897216	0.815	0.281	0	5
Mxd12	0	1.76486703	0.84	0.225	0	5
Dgat13	0	1.75126391	0.669	0.162	0	5
Plaur3	0	1.73638334	0.875	0.29	0	5
Adpgk2	0	1.73545072	0.666	0.175	0	5
Hdc2	0	1.72021848	0.739	0.17	0	5
Il1r22	0	1.67399068	0.727	0.154	0	5
S100a113	0	1.65041491	0.997	0.668	0	5
Trem12	0	1.5975638	0.659	0.15	0	5
Mcemp13	0	1.58519094	0.673	0.194	0	5
Grina2	0	1.54744682	0.835	0.279	0	5
Lcn22	0	1.53558014	0.988	0.262	0	5
Hp3	0	1.51847492	0.849	0.278	0	5
Clec4e2	0	1.51408211	0.601	0.148	0	5
Cd1772	0	1.5066349	0.57	0.145	0	5
Lmnb12	0	1.50223731	0.792	0.27	0	5
Cebpb1	0	1.46494645	0.937	0.363	0	5
Alox5ap3	0	1.4637512	0.839	0.298	0	5
Ncf22	0	1.45663178	0.677	0.214	0	5
Clec4d1	0	1.45126824	0.578	0.103	0	5
Lilr4b2	0	1.44829538	0.67	0.18	0	5
Cxcr21	0	1.43973583	0.502	0.081	0	5
Dhrs73	0	1.43901449	0.592	0.186	0	5
Lcp12	0	1.43606093	0.802	0.297	0	5

Lrg13	0	1.432165	0.564	0.154	0	5
Gsr2	0	1.40908945	0.752	0.288	0	5
Msrb12	0	1.40796687	0.831	0.295	0	5
Prr132	0	1.38532852	0.606	0.189	0	5
Tyrobp2	0	1.37364233	0.985	0.383	0	5
Il1f91	0	1.33501425	0.337	0.044	0	5
Slc7a112	0	1.32741158	0.521	0.11	0	5
Srgn1	0	1.32454951	0.967	0.488	0	5
Prdx53	0	1.2963147	0.922	0.426	0	5
Cxcl21	0	1.29237227	0.698	0.151	0	5
Fcer1g3	0	1.26110549	0.956	0.369	0	5
Bmx1	0	1.25882746	0.356	0.068	0	5
Cd523	0	1.25491974	0.986	0.492	0	5
Lilrb4a2	0	1.22271173	0.559	0.156	0	5
Dhrs9	0	1.22207192	0.317	0.047	0	5
Actg12	0	1.12772887	0.987	0.701	0	5
Tmsb4x3	0	1.01011569	1	0.9	0	5
Ngp2	0	0.7946854	0.947	0.244	0	5
Fos2	6.18E-303	1.102231	0.829	0.337	2.00E-298	5
R3hdm42	1.49E-302	1.45448955	0.552	0.168	4.80E-298	5
Rdh121	1.71E-302	1.15107805	0.293	0.045	5.52E-298	5
S100a62	2.29E-297	1.17075439	0.985	0.602	7.39E-293	5
Stx112	2.41E-297	1.24202014	0.35	0.067	7.76E-293	5
Mrgpra2b2	4.87E-296	1.48106462	0.393	0.088	1.57E-291	5
C5ar12	1.15E-293	1.22957966	0.449	0.109	3.73E-289	5
Actb3	5.97E-291	1.08290383	0.999	0.947	1.93E-286	5
Fth11	9.48E-291	1.10711043	0.986	0.914	3.06E-286	5
Fpr1	2.72E-290	1.12598338	0.254	0.036	8.79E-286	5
Ly6g2	3.35E-287	1.28573188	0.47	0.12	1.08E-282	5
Litaf2	2.22E-286	1.19969978	0.679	0.244	7.18E-282	5
Fpr22	2.56E-286	1.29821177	0.396	0.089	8.27E-282	5
Pygl3	2.93E-285	1.34062753	0.483	0.135	9.45E-281	5
Dusp11	3.50E-274	0.92163674	0.638	0.204	1.13E-269	5
Junb1	2.14E-271	1.05476824	0.864	0.412	6.91E-267	5
Mgst13	3.24E-271	1.32942101	0.611	0.216	1.04E-266	5
Cdk11b2	5.91E-267	1.35276598	0.545	0.177	1.91E-262	5
Slfn43	4.84E-265	1.27711139	0.387	0.091	1.56E-260	5
Gm204061	1.80E-264	1.13069909	0.298	0.054	5.80E-260	5
Btg22	1.86E-255	1.03555611	0.765	0.321	6.00E-251	5
Pilra2	4.79E-255	1.16862304	0.427	0.112	1.55E-250	5
Slc2a32	5.24E-254	1.18129158	0.37	0.085	1.69E-249	5
Mrpl331	1.96E-253	1.12065338	0.853	0.433	6.34E-249	5
Sell1	7.50E-252	1.14562477	0.54	0.174	2.42E-247	5

Ccr11	1.17E-251	1.03798878	0.388	0.09	3.79E-247	5
Itgam3	6.04E-248	1.22726596	0.52	0.166	1.95E-243	5
Pira21	6.78E-248	1.12677411	0.318	0.066	2.19E-243	5
Lyz22	1.64E-246	0.35126917	0.963	0.372	5.29E-242	5
Cd332	4.87E-242	1.0926994	0.345	0.077	1.57E-237	5
Tmcc12	1.69E-241	1.26579242	0.44	0.123	5.47E-237	5
Cd300lf2	1.76E-240	1.11498395	0.369	0.088	5.67E-236	5
Fxyd51	2.55E-240	0.93566655	0.793	0.349	8.22E-236	5
Taldo12	2.18E-239	1.1100928	0.784	0.374	7.02E-235	5
Gda1	1.99E-236	1.15654228	0.559	0.191	6.41E-232	5
Stk17b1	1.68E-233	1.04929576	0.715	0.3	5.43E-229	5
Ncf13	6.15E-232	1.24418367	0.499	0.165	1.98E-227	5
Ets22	9.73E-225	1.08390947	0.487	0.152	3.14E-220	5
Anxa13	1.21E-223	0.9717009	0.811	0.361	3.89E-219	5
Itgb23	6.53E-222	1.13987591	0.489	0.162	2.11E-217	5
Sorl12	3.19E-216	1.11124997	0.442	0.134	1.03E-211	5
Hcst3	5.43E-214	1.13163497	0.547	0.202	1.75E-209	5
Ifitm32	2.73E-213	0.79110117	0.88	0.445	8.83E-209	5
Ly6c22	1.61E-208	0.93259988	0.583	0.21	5.18E-204	5
Selenon2	2.08E-205	0.94501753	0.336	0.081	6.72E-201	5
Acod11	7.22E-200	0.89220928	0.294	0.063	2.33E-195	5
Ndel12	2.05E-199	1.06125882	0.43	0.133	6.61E-195	5
Osm2	1.77E-198	1.09499054	0.365	0.1	5.70E-194	5
Trib12	1.83E-193	1.03605527	0.445	0.144	5.91E-189	5
Ccl61	4.78E-193	0.75552197	0.518	0.167	1.54E-188	5
Coro1a3	1.22E-189	0.92257692	0.824	0.418	3.93E-185	5
Hacd4	2.88E-189	0.93981959	0.261	0.056	9.29E-185	5
Kctd122	1.75E-188	1.09124312	0.491	0.175	5.65E-184	5
Txn13	6.08E-188	0.86802609	0.925	0.62	1.96E-183	5
Ostf13	1.27E-187	1.04511987	0.669	0.312	4.10E-183	5
Lst12	9.52E-187	0.82278638	0.613	0.249	3.07E-182	5
Nabp12	2.32E-182	0.98284931	0.295	0.071	7.50E-178	5
Cd94	5.43E-182	0.91501117	0.704	0.323	1.75E-177	5
Padi42	1.31E-181	1.01123153	0.289	0.071	4.22E-177	5
Fosl22	2.09E-181	0.99740843	0.427	0.139	6.76E-177	5
Rac24	1.10E-180	0.91117402	0.776	0.391	3.56E-176	5
Chil12	1.87E-179	1.05461128	0.314	0.084	6.05E-175	5
Fosl11	1.90E-178	1.09329108	0.294	0.074	6.12E-174	5
Slc15a31	1.42E-176	0.79672209	0.313	0.078	4.57E-172	5
Mcl11	3.01E-176	0.94043635	0.718	0.358	9.71E-172	5
Themis2	3.26E-175	0.85783936	0.252	0.055	1.05E-170	5
Csf3r1	9.31E-175	0.7957493	0.358	0.101	3.01E-170	5
Vasp3	9.14E-174	1.00755805	0.486	0.181	2.95E-169	5

Plk31	1.04E-169	0.89592479	0.268	0.063	3.36E-165	5
Slfn11	1.50E-165	0.79296016	0.267	0.063	4.84E-161	5
Cyp4f181	8.36E-163	0.84959	0.311	0.082	2.70E-158	5
Cd142	4.71E-161	1.04382927	0.457	0.17	1.52E-156	5
Ifitm21	3.12E-160	0.92571805	0.786	0.44	1.01E-155	5
Ptprc2	1.05E-157	0.80623885	0.592	0.256	3.38E-153	5
Il1b1	1.46E-156	0.33875082	0.405	0.126	4.71E-152	5
Anxa111	5.80E-154	0.94934331	0.321	0.096	1.87E-149	5
Gmfg3	2.17E-153	0.80402178	0.748	0.387	7.01E-149	5
Vsir3	2.22E-153	1.01038826	0.377	0.128	7.16E-149	5
Arhgdib2	4.74E-152	0.78512761	0.824	0.417	1.53E-147	5
Rab8b2	1.90E-148	0.9662226	0.441	0.169	6.12E-144	5
Xdh2	2.08E-147	0.98261615	0.315	0.097	6.71E-143	5
Phlda11	4.27E-145	0.98033308	0.335	0.105	1.38E-140	5
Gdpd31	1.13E-142	0.9614628	0.258	0.069	3.64E-138	5
Plbd12	1.64E-142	0.94209533	0.412	0.152	5.31E-138	5
Nlrp31	4.60E-141	0.72864207	0.297	0.083	1.48E-136	5
Pgd2	8.51E-141	0.96385433	0.417	0.161	2.75E-136	5
Zyx2	4.51E-140	0.85892583	0.452	0.18	1.46E-135	5
Actn13	6.84E-140	0.87318923	0.475	0.188	2.21E-135	5
Lsp11	6.28E-139	0.73688215	0.623	0.286	2.03E-134	5
Pirb3	1.86E-138	0.88739089	0.403	0.15	6.00E-134	5
Itgb2l2	7.23E-137	0.94790768	0.278	0.083	2.33E-132	5
Fmnl11	4.92E-135	0.91780918	0.363	0.127	1.59E-130	5
Sat11	1.15E-134	0.84907869	0.608	0.293	3.70E-130	5
Clec4a22	2.27E-134	0.9762451	0.409	0.16	7.33E-130	5
Nfam12	1.17E-132	0.88198173	0.328	0.11	3.77E-128	5
Nudt41	5.38E-132	0.87384041	0.435	0.173	1.74E-127	5
Gadd45a3	1.83E-131	0.94248464	0.434	0.172	5.91E-127	5
Sem13	3.81E-131	0.67266054	0.934	0.655	1.23E-126	5
Gpsm33	4.02E-128	0.96012754	0.397	0.157	1.30E-123	5
Lgals32	4.91E-127	0.54223859	0.755	0.376	1.59E-122	5
Plek1	8.04E-126	0.73992275	0.389	0.143	2.59E-121	5
Cyba3	1.51E-123	0.63496458	0.887	0.512	4.88E-119	5
Fbxl51	8.49E-123	0.91426803	0.335	0.115	2.74E-118	5
Syne12	1.08E-122	0.93290072	0.305	0.103	3.50E-118	5
Suco1	4.28E-122	0.8696909	0.313	0.106	1.38E-117	5
Samd9l1	7.02E-122	0.90115756	0.343	0.123	2.27E-117	5
Glipr22	7.51E-122	0.91035021	0.305	0.103	2.43E-117	5
Lyst2	1.34E-121	0.85115338	0.329	0.115	4.33E-117	5
Spi13	2.33E-121	0.85904434	0.465	0.207	7.53E-117	5
Ccpg11	9.64E-120	0.76000252	0.268	0.08	3.11E-115	5
Arpc32	5.04E-119	0.72679579	0.817	0.503	1.63E-114	5

Trem32	1.63E-118	0.90752525	0.343	0.129	5.27E-114	5
Cotl12	3.03E-117	0.82620388	0.463	0.208	9.77E-113	5
Rflnb2	6.48E-114	0.94488917	0.283	0.095	2.09E-109	5
Ckap42	1.57E-113	0.90234019	0.426	0.186	5.07E-109	5
Mindy11	3.01E-113	0.83548104	0.315	0.11	9.72E-109	5
Degs12	2.52E-112	0.95782976	0.383	0.159	8.14E-108	5
4932438A13I	4.23E-109	0.84335878	0.352	0.135	1.37E-104	5
Tut72	1.23E-107	0.79455581	0.44	0.195	3.97E-103	5
Cd532	9.11E-107	0.72905531	0.513	0.247	2.94E-102	5
Atp6v1e13	1.31E-106	0.80655966	0.618	0.337	4.22E-102	5
2310001H17I	1.78E-105	0.74285325	0.331	0.124	5.76E-101	5
Emb1	2.06E-104	0.7211484	0.367	0.144	6.64E-100	5
Glrx2	1.08E-103	0.88971764	0.339	0.135	3.50E-99	5
Sbno11	1.28E-102	0.81164386	0.397	0.167	4.12E-98	5
Pdcd61	7.35E-100	0.95670761	0.393	0.176	2.37E-95	5
Selplg1	3.03E-99	0.69565486	0.377	0.155	9.78E-95	5
Egr11	4.37E-98	0.47328658	0.407	0.169	1.41E-93	5
Nr4a12	6.94E-98	0.781838	0.427	0.193	2.24E-93	5
Samhd11	1.84E-94	0.65216327	0.397	0.168	5.95E-90	5
Pnkp2	2.06E-93	0.8654118	0.282	0.107	6.65E-89	5
Selenok2	5.07E-93	0.7299513	0.638	0.382	1.64E-88	5
Msra2	8.12E-93	0.79927209	0.259	0.093	2.62E-88	5
Map1lc3b2	8.22E-91	0.67077113	0.683	0.422	2.65E-86	5
Eif11	2.34E-89	0.43319142	0.976	0.832	7.55E-85	5
Nfkbiz2	4.24E-89	0.71443109	0.319	0.126	1.37E-84	5
Fcgr3	4.59E-88	0.58987622	0.296	0.114	1.48E-83	5
Ier22	8.99E-88	0.69560841	0.508	0.268	2.90E-83	5
Tnfrsf1a1	2.25E-87	0.78121387	0.275	0.104	7.28E-83	5
Rassf31	9.82E-87	0.65472343	0.253	0.089	3.17E-82	5
Iqgap12	1.27E-86	0.62923883	0.644	0.38	4.11E-82	5
AB1246111	2.70E-86	0.67186631	0.275	0.104	8.72E-82	5
Myl12b2	1.76E-85	0.62413091	0.68	0.411	5.69E-81	5
Svil1	3.70E-85	0.73761941	0.271	0.102	1.19E-80	5
Sh3bgrl33	1.59E-84	0.5276617	0.868	0.556	5.13E-80	5
Kdm7a1	4.85E-84	0.68855248	0.305	0.122	1.56E-79	5
Tpd521	6.65E-84	0.60525976	0.388	0.172	2.15E-79	5
Myh92	1.05E-83	0.65297729	0.595	0.341	3.38E-79	5
Timp22	1.26E-83	0.45756504	0.389	0.162	4.06E-79	5
Ncf42	1.62E-83	0.75911987	0.356	0.162	5.23E-79	5
Ppp1r182	1.16E-82	0.73152476	0.356	0.16	3.76E-78	5
Gnai21	7.76E-82	0.59450246	0.736	0.481	2.50E-77	5
Atp6v1g12	1.77E-81	0.64679123	0.575	0.333	5.70E-77	5
Lasp11	1.94E-81	0.67129723	0.323	0.137	6.28E-77	5

Cwc252	3.75E-81	0.74454485	0.257	0.095	1.21E-76	5
Rab6a1	4.86E-80	0.73341663	0.299	0.123	1.57E-75	5
Rasa21	8.44E-79	0.78486004	0.252	0.095	2.73E-74	5
Anxa22	9.57E-77	0.39118839	0.709	0.407	3.09E-72	5
Arpc52	1.93E-76	0.63680914	0.581	0.342	6.25E-72	5
Hcls11	8.91E-76	0.67117674	0.317	0.139	2.87E-71	5
Vps37b1	2.27E-75	0.57812551	0.309	0.128	7.33E-71	5
Actr32	9.23E-75	0.58383464	0.575	0.335	2.98E-70	5
Csf2ra1	3.39E-72	0.69172035	0.256	0.103	1.10E-67	5
Adipor11	4.56E-65	0.61888251	0.356	0.174	1.47E-60	5
Notch21	1.62E-64	0.61805739	0.396	0.202	5.23E-60	5
Rhog2	5.33E-63	0.67830808	0.335	0.167	1.72E-58	5
Picalm1	8.85E-63	0.47963344	0.419	0.215	2.86E-58	5
Mettl92	3.77E-62	0.70429984	0.292	0.136	1.22E-57	5
Cap12	1.33E-61	0.57989449	0.389	0.202	4.29E-57	5
Gabarap1	2.61E-61	0.45496285	0.706	0.471	8.43E-57	5
Flna3	2.63E-60	0.55129059	0.527	0.31	8.48E-56	5
Cybb2	3.80E-60	0.42694847	0.448	0.227	1.23E-55	5
Rab72	1.33E-57	0.55880628	0.355	0.182	4.31E-53	5
Gpi13	1.36E-57	0.58296624	0.471	0.278	4.40E-53	5
Rgs182	6.87E-57	0.67092985	0.257	0.119	2.22E-52	5
Tkt2	8.57E-57	0.57868157	0.485	0.29	2.77E-52	5
Tgoln11	2.72E-55	0.59245009	0.271	0.124	8.77E-51	5
Plp22	3.47E-52	0.57823725	0.305	0.154	1.12E-47	5
Zfp361	2.06E-49	0.38298508	0.403	0.222	6.64E-45	5
Capza12	4.68E-48	0.55942822	0.394	0.231	1.51E-43	5
Cnn21	7.32E-48	0.5545076	0.292	0.149	2.36E-43	5
Ier31	1.44E-47	0.28985325	0.291	0.142	4.64E-43	5
Dstn3	1.82E-47	0.41385791	0.638	0.396	5.87E-43	5
Hist2h2aa11	2.32E-47	0.45847564	0.267	0.127	7.50E-43	5
Bcl101	4.43E-47	0.60913469	0.331	0.181	1.43E-42	5
Ccnl11	5.95E-47	0.47288365	0.413	0.242	1.92E-42	5
Cd442	7.49E-45	0.3897533	0.44	0.258	2.42E-40	5
Pfn12	2.46E-44	0.34689382	0.933	0.741	7.95E-40	5
Lbr1	1.23E-43	0.4513802	0.411	0.249	3.99E-39	5
Arpc21	1.77E-43	0.36896074	0.648	0.439	5.71E-39	5
Skap21	2.49E-43	0.51082879	0.282	0.147	8.03E-39	5
Pet1001	3.90E-42	0.47460085	0.519	0.348	1.26E-37	5
Atxn101	7.58E-42	0.47120503	0.261	0.133	2.45E-37	5
Slfn22	1.19E-41	0.47586197	0.296	0.158	3.83E-37	5
Nktr	6.82E-41	0.55930874	0.296	0.16	2.20E-36	5
Diaph1	1.89E-39	0.45655854	0.277	0.145	6.09E-35	5
Ypel32	2.09E-39	0.28107647	0.394	0.224	6.75E-35	5

Clk12	1.66E-38	0.37701988	0.442	0.278	5.36E-34	5
Efhd21	2.19E-38	0.48110182	0.289	0.16	7.06E-34	5
Lyn2	4.12E-38	0.32824828	0.365	0.211	1.33E-33	5
Lamp21	2.13E-37	0.37953905	0.366	0.215	6.89E-33	5
Rab5if2	4.19E-36	0.49257267	0.407	0.264	1.35E-31	5
Prdx63	1.82E-35	0.54023432	0.425	0.279	5.86E-31	5
Rap1b1	1.03E-34	0.3891823	0.397	0.25	3.34E-30	5
Cdc42se1	1.42E-34	0.44508542	0.296	0.168	4.59E-30	5
Gapdh2	1.99E-34	0.27273202	0.765	0.536	6.44E-30	5
D8Ertd738e1	5.45E-34	0.44043888	0.579	0.418	1.76E-29	5
Cdk2ap21	1.29E-33	0.4461259	0.425	0.281	4.17E-29	5
Srsf52	1.31E-32	0.3602732	0.537	0.377	4.24E-28	5
Sri1	3.95E-32	0.41208432	0.447	0.303	1.28E-27	5
Hipk1	3.41E-31	0.45645633	0.307	0.182	1.10E-26	5
Ccnd3	1.14E-30	0.43073705	0.313	0.192	3.68E-26	5
Cdc421	2.09E-30	0.31496884	0.657	0.479	6.75E-26	5
Lamtor42	9.52E-30	0.41064136	0.415	0.275	3.07E-25	5
Itm2b1	2.17E-29	0.26045679	0.726	0.529	6.99E-25	5
Lmo43	4.19E-29	0.30949137	0.393	0.255	1.35E-24	5
Arhgap45	2.26E-28	0.41073951	0.265	0.155	7.29E-24	5
Card191	6.66E-27	0.36078228	0.255	0.148	2.15E-22	5
Fam32a1	7.00E-27	0.36346132	0.276	0.165	2.26E-22	5
Wdr11	2.45E-26	0.4369046	0.283	0.175	7.90E-22	5
Ppp2r5a1	1.71E-25	0.39731512	0.31	0.199	5.53E-21	5
Actr21	6.40E-23	0.26689183	0.436	0.309	2.07E-18	5
Ube2d31	1.86E-22	0.27989568	0.489	0.358	6.02E-18	5
Ifrd1	6.39E-22	0.33169795	0.26	0.16	2.06E-17	5
Dazap21	1.12E-21	0.3495752	0.32	0.212	3.63E-17	5
Arpc43	2.84E-20	0.30332248	0.369	0.262	9.17E-16	5
Aldh22	2.08E-16	0.26671814	0.366	0.266	6.72E-12	5
Magoh2	1.40E-15	0.37260975	0.349	0.262	4.51E-11	5
Tmbim61	2.80E-15	0.26661952	0.394	0.295	9.05E-11	5
Apoe	0	5.33091254	0.835	0.079	0	6
C1qa	0	4.46405014	0.664	0.006	0	6
C1qb	0	4.29919047	0.655	0.005	0	6
Arg1	0	4.00088492	0.475	0.004	0	6
C1qc	0	3.98635192	0.567	0.004	0	6
Ctss	0	3.81065631	0.799	0.097	0	6
Mmp12	0	3.28822187	0.255	0.002	0	6
Ctsc	0	3.18887786	0.569	0.073	0	6
Pf4	0	3.15805893	0.357	0.009	0	6
H2-Aa	0	2.94598223	0.516	0.075	0	6
H2-Ab1	0	2.87759867	0.482	0.097	0	6

Cd74	0	2.70101102	0.741	0.148	0	6
Mrc1	0	2.69048025	0.293	0.006	0	6
H2-Eb1	0	2.6270301	0.438	0.069	0	6
Ccl62	0	2.59823031	0.621	0.163	0	6
Clec4n	0	2.53251202	0.281	0.007	0	6
Tgfbi1	0	2.52750944	0.41	0.081	0	6
Fcgr2b	0	2.51280312	0.353	0.057	0	6
Psap	0	2.49483615	0.729	0.288	0	6
Lgmn	0	2.47467666	0.349	0.038	0	6
Dab2	0	2.34787315	0.267	0.017	0	6
Ccl9	0	2.29664099	0.286	0.042	0	6
Aif1	0	2.27292667	0.268	0.017	0	6
Ifi30	0	2.20882044	0.5	0.116	0	6
Lyz23	0	1.81326932	0.898	0.379	0	6
Gm424181	0	1.60439136	0.994	0.939	0	6
Bcl2a1b	7.54E-301	2.23810429	0.305	0.049	2.43E-296	6
Ctsb	9.82E-261	2.57486463	0.577	0.225	3.17E-256	6
Fth12	5.36E-251	1.2642884	0.984	0.915	1.73E-246	6
Trf	4.49E-249	2.43420726	0.353	0.081	1.45E-244	6
Fn11	3.52E-233	1.7514006	0.547	0.183	1.14E-228	6
Mpeg1	2.49E-226	2.02629408	0.327	0.074	8.04E-222	6
Cst3	9.24E-185	1.86025606	0.718	0.465	2.98E-180	6
Ctsd1	1.88E-173	2.22093579	0.503	0.224	6.06E-169	6
Grn	4.58E-170	2.03637021	0.398	0.14	1.48E-165	6
Ctsz	4.89E-167	1.89873266	0.429	0.161	1.58E-162	6
B2m1	2.32E-165	1.16094784	0.854	0.705	7.51E-161	6
Ms4a6c	3.11E-161	1.6713942	0.268	0.064	1.00E-156	6
Tpt11	1.80E-158	0.80445031	0.949	0.863	5.80E-154	6
Gm2a	2.57E-157	1.96867776	0.325	0.098	8.30E-153	6
Ftl12	1.37E-149	1.11927331	0.882	0.806	4.42E-145	6
Fcgr31	4.21E-145	2.14755089	0.335	0.113	1.36E-140	6
Wfdc171	1.17E-136	1.10574132	0.344	0.108	3.77E-132	6
Lamp11	1.78E-131	1.69953713	0.47	0.226	5.76E-127	6
Spp11	2.90E-130	1.21997262	0.56	0.255	9.37E-126	6
Ly86	2.26E-123	1.60822172	0.258	0.075	7.29E-119	6
Rps121	3.12E-121	0.74952258	0.899	0.681	1.01E-116	6
Eef1a11	4.08E-120	0.75169441	0.92	0.718	1.32E-115	6
Mt11	2.16E-118	1.08620207	0.503	0.231	6.98E-114	6
Rps291	2.19E-116	0.57266923	0.998	0.953	7.06E-112	6
Rps151	2.44E-115	0.98802601	0.756	0.526	7.87E-111	6
Rplp11	7.68E-113	0.68413158	0.939	0.699	2.48E-108	6
Rps81	1.40E-112	0.66891347	0.951	0.739	4.52E-108	6
mt-Co32	3.97E-112	0.78894892	0.909	0.794	1.28E-107	6

Npc2	2.10E-110	1.59604467	0.466	0.242	6.78E-106	6
Tmsb4x4	2.59E-100	0.59733345	0.997	0.9	8.38E-96	6
mt-Atp62	1.28E-89	0.76933392	0.857	0.743	4.14E-85	6
Fcer1g4	5.72E-89	0.93172554	0.661	0.387	1.85E-84	6
Hspa81	3.12E-80	0.95842327	0.69	0.546	1.01E-75	6
Cstb	5.26E-79	1.56648998	0.393	0.212	1.70E-74	6
Laptm51	4.48E-78	1.58023676	0.44	0.268	1.45E-73	6
Rps251	4.63E-77	0.80852601	0.756	0.625	1.49E-72	6
Rps211	2.78E-74	0.43160933	0.952	0.787	8.96E-70	6
Rpl13a1	5.87E-74	0.74550254	0.739	0.565	1.90E-69	6
mt-Co22	3.05E-73	0.67744837	0.86	0.783	9.84E-69	6
Fabp5	3.73E-72	2.01483744	0.253	0.105	1.20E-67	6
Rpl51	6.89E-67	0.88014526	0.63	0.454	2.22E-62	6
Xist	1.44E-65	1.22989159	0.285	0.126	4.66E-61	6
Rack11	2.81E-65	0.7431328	0.696	0.529	9.06E-61	6
mt-Co11	2.09E-63	0.4953224	0.927	0.882	6.74E-59	6
Ybx11	1.27E-62	0.99961343	0.588	0.445	4.12E-58	6
Rpl23a1	1.60E-61	0.65572692	0.69	0.508	5.16E-57	6
Rps141	3.58E-61	0.55833313	0.841	0.713	1.16E-56	6
Ctsh	1.55E-60	1.45565819	0.252	0.113	5.01E-56	6
Crip1	1.82E-60	0.75861326	0.589	0.394	5.88E-56	6
Rplp21	8.30E-60	0.57148282	0.846	0.712	2.68E-55	6
Ctsl1	1.08E-59	1.30804517	0.367	0.204	3.50E-55	6
Rpl311	7.91E-58	0.81083836	0.658	0.541	2.55E-53	6
Rrbp11	9.48E-56	1.14263858	0.428	0.268	3.06E-51	6
Rpl382	1.67E-55	0.43972688	0.963	0.838	5.39E-51	6
Lgals33	2.02E-53	1.15683235	0.519	0.391	6.52E-49	6
mt-Nd11	9.35E-51	0.85150067	0.603	0.471	3.02E-46	6
Actg13	5.80E-50	0.57547438	0.805	0.712	1.87E-45	6
Rpl61	8.75E-49	0.43845891	0.884	0.754	2.83E-44	6
Rpl232	6.50E-48	0.41552606	0.927	0.821	2.10E-43	6
Mif1	1.21E-47	0.89724823	0.49	0.336	3.92E-43	6
Tyrobp3	3.71E-46	0.31850328	0.659	0.403	1.20E-41	6
Rps171	1.26E-44	0.66509588	0.68	0.56	4.06E-40	6
Rpl121	1.30E-44	0.50597954	0.713	0.533	4.21E-40	6
mt-Nd21	2.43E-44	0.91041834	0.512	0.378	7.85E-40	6
Atox1	2.75E-41	1.2074441	0.474	0.392	8.87E-37	6
mt-Nd42	6.35E-41	0.80920898	0.586	0.488	2.05E-36	6
Atp6v0c1	1.93E-40	1.19836505	0.473	0.396	6.23E-36	6
Rpl261	3.22E-39	0.44990803	0.824	0.7	1.04E-34	6
Hsp90b11	2.58E-38	0.87805015	0.491	0.376	8.34E-34	6
App	2.32E-36	1.24503288	0.272	0.16	7.50E-32	6
Rpl171	2.87E-35	0.42416608	0.77	0.668	9.28E-31	6

Hsp90ab11	5.40E-35	0.37098056	0.718	0.577	1.74E-30	6
Calm12	9.03E-35	0.64695994	0.663	0.648	2.92E-30	6
Marcks2	2.22E-34	0.96529095	0.318	0.194	7.17E-30	6
Rpl37a1	4.94E-33	0.26858762	0.97	0.902	1.59E-28	6
Lcp13	1.33E-32	1.12723223	0.414	0.32	4.28E-28	6
Rps231	6.36E-32	0.34107962	0.884	0.759	2.05E-27	6
Nme21	1.75E-28	0.57166654	0.529	0.408	5.64E-24	6
Itm2b2	8.09E-28	0.85993771	0.56	0.538	2.61E-23	6
Rpl412	2.17E-27	0.27003655	0.957	0.9	7.02E-23	6
H2-K11	1.95E-26	0.70182507	0.525	0.458	6.31E-22	6
Akr1a1	1.43E-25	1.04565716	0.268	0.175	4.62E-21	6
Hif1a1	1.84E-25	1.11466681	0.263	0.171	5.92E-21	6
Cd631	2.46E-25	1.04923185	0.355	0.271	7.94E-21	6
Rpl141	5.89E-25	0.45658596	0.631	0.524	1.90E-20	6
Cd524	1.44E-24	0.33336921	0.688	0.51	4.65E-20	6
Rps61	3.89E-24	0.58822114	0.572	0.501	1.25E-19	6
Calr1	1.42E-21	0.92820673	0.329	0.242	4.58E-17	6
Cotl13	1.49E-21	1.27583833	0.29	0.219	4.82E-17	6
S100a41	2.66E-21	0.41128418	0.345	0.223	8.58E-17	6
Sh3bgrl	3.41E-21	1.05080269	0.263	0.181	1.10E-16	6
Rps162	6.01E-20	0.28997675	0.848	0.777	1.94E-15	6
mt-Cytb2	1.08E-19	0.37672431	0.71	0.665	3.50E-15	6
P4hb1	1.62E-19	0.95053942	0.259	0.18	5.24E-15	6
Cfl11	1.92E-19	0.57216748	0.598	0.623	6.19E-15	6
Gapdh3	3.75E-19	0.71736003	0.547	0.548	1.21E-14	6
Pdia31	4.33E-19	0.87059476	0.336	0.256	1.40E-14	6
Gdi21	5.18E-19	1.03554877	0.306	0.234	1.67E-14	6
Rps92	5.33E-19	0.27485212	0.856	0.878	1.72E-14	6
Sdcbp1	7.48E-19	1.13047466	0.293	0.225	2.42E-14	6
Prdx11	1.69E-18	0.74353227	0.464	0.394	5.46E-14	6
Fxyd52	2.98E-18	0.62767108	0.43	0.37	9.62E-14	6
Arpc22	8.22E-18	0.873102	0.461	0.45	2.65E-13	6
Rpl222	2.21E-17	0.29624018	0.762	0.665	7.14E-13	6
Ifitm33	3.25E-17	0.58784319	0.518	0.466	1.05E-12	6
Ly6e2	4.58E-17	0.55138142	0.475	0.405	1.48E-12	6
Hspa51	1.18E-16	0.81825257	0.408	0.357	3.80E-12	6
Rps111	4.57E-16	0.25750879	0.803	0.729	1.48E-11	6
Itgb11	5.27E-16	0.65807486	0.304	0.225	1.70E-11	6
Rpl36al1	6.91E-16	0.4732866	0.559	0.513	2.23E-11	6
Gpx1	1.57E-15	0.47848831	0.538	0.52	5.07E-11	6
Rpl7a2	6.32E-15	0.46009118	0.581	0.562	2.04E-10	6
Eef21	2.15E-14	0.64221946	0.433	0.385	6.94E-10	6
Rpl36a1	4.71E-14	0.31044496	0.678	0.582	1.52E-09	6

Tpi11	5.86E-14	0.88490591	0.263	0.2	1.89E-09	6
Slc25a5	2.29E-12	0.90925427	0.315	0.27	7.41E-08	6
Cox7b1	3.21E-12	0.87407563	0.375	0.345	1.04E-07	6
Cox7c2	4.54E-12	0.35591548	0.65	0.675	1.47E-07	6
Cox4i11	8.24E-12	0.57041633	0.512	0.527	2.66E-07	6
Vim1	1.34E-11	0.3472411	0.536	0.491	4.34E-07	6
Cdc422	1.25E-10	0.71269373	0.465	0.49	4.03E-06	6
Canx1	1.52E-10	0.67741751	0.29	0.239	4.91E-06	6
Rpl42	6.13E-10	0.49883796	0.448	0.418	1.98E-05	6
Lamp22	7.49E-10	0.88363275	0.263	0.221	2.42E-05	6
Pkm1	1.47E-09	0.59536655	0.435	0.432	4.75E-05	6
Cyba4	3.26E-09	0.39135664	0.561	0.531	0.00010514	6
Psme11	5.36E-09	0.80173565	0.337	0.31	0.00017303	6
Dbi1	1.31E-08	0.61284231	0.344	0.311	0.0004231	6
Sh3bgrl34	1.60E-08	0.42153472	0.555	0.574	0.00051782	6
Aldoa2	2.39E-08	0.74563903	0.417	0.424	0.00077297	6
Rpl33	2.77E-08	0.26614171	0.597	0.561	0.00089584	6
Capza21	2.86E-08	0.85838466	0.303	0.279	0.00092218	6
Eef1b21	3.79E-08	0.32667136	0.558	0.538	0.00122333	6
Pgk11	5.53E-08	0.87272267	0.278	0.25	0.00178686	6
Ucp21	6.38E-08	0.64721031	0.371	0.352	0.00205895	6
mt-Nd31	1.17E-07	0.63063462	0.334	0.303	0.00376873	6
Actr33	2.29E-07	0.84415262	0.35	0.348	0.0073841	6
Cox5a	2.59E-07	0.65788612	0.392	0.393	0.00837017	6
Arhgdia	5.15E-07	0.78933249	0.251	0.218	0.01663467	6
Ndufb1-ps1	6.00E-07	0.35745718	0.597	0.642	0.01937504	6
Atp5b1	6.41E-07	0.6445612	0.336	0.318	0.02070553	6
Eef1g1	1.66E-06	0.5063018	0.322	0.288	0.0535211	6
Clta1	1.73E-06	0.75086544	0.333	0.323	0.05582569	6
Ifitm22	1.95E-06	0.29553083	0.461	0.459	0.06286407	6
Hsp90aa11	2.65E-06	0.55603623	0.385	0.372	0.08544573	6
Eif3a1	2.83E-06	0.55666237	0.289	0.257	0.09142497	6
Atp6v0e	3.70E-06	0.27868378	0.21	0.311	0.11939575	6
Eif4a11	4.10E-06	0.63029706	0.364	0.361	0.13238015	6
Bola21	6.23E-06	0.63023873	0.273	0.244	0.2010202	6
Prelid11	6.55E-06	0.25396397	0.183	0.269	0.21131343	6
Anxa51	1.66E-05	0.41227661	0.289	0.252	0.53739916	6
Atp5e1	1.83E-05	0.26871545	0.641	0.703	0.59077419	6
Cybb3	2.92E-05	0.73808431	0.26	0.238	0.94387365	6
Lars21	4.95E-05	0.58798538	0.262	0.24	1	6
Tubb51	0.00011319	0.27191418	0.392	0.362	1	6
Tkt3	0.00012619	0.3864668	0.214	0.305	1	6
H2-D12	0.00013289	0.27518046	0.588	0.667	1	6

Arpc1b1	0.00015155	0.53986106	0.407	0.444	1	6
Tuba1b	0.00015772	0.53981032	0.26	0.237	1	6
Ptprc3	0.00035856	0.61501026	0.282	0.274	1	6
Hnrnpa2b11	0.0005986	0.46784335	0.401	0.417	1	6
Mrpl521	0.00076658	0.46948803	0.315	0.305	1	6
Ssr4	0.00090257	0.59046371	0.263	0.253	1	6
Smdt11	0.00093427	0.25615511	0.257	0.347	1	6
Arf11	0.00095257	0.31509809	0.211	0.292	1	6
Dync1i22	0.00118889	0.28773616	0.214	0.291	1	6
Ndufb82	0.00146942	0.26438188	0.224	0.302	1	6
Nap1l11	0.00207931	0.49773469	0.314	0.311	1	6
Ldha1	0.00279821	0.56122398	0.37	0.398	1	6
Psmb41	0.00309485	0.27277337	0.197	0.262	1	6
Rap1b2	0.00370072	0.71389157	0.255	0.258	1	6
Ywhab1	0.00383699	0.34909486	0.238	0.318	1	6
Atp5f1	0.00542952	0.33637488	0.246	0.328	1	6
Pomp2	0.00633505	0.27119876	0.268	0.353	1	6
Pgam11	0.00647745	0.67910457	0.267	0.271	1	6
Pcbp21	0.00673152	0.57148796	0.321	0.337	1	6
Rnasek	0.00871561	0.35997816	0.222	0.296	1	6
Arpc53	0.0089074	0.67563598	0.327	0.356	1	6
Hist1h2ae	0	4.26345796	0.778	0.165	0	7
Hist1h2ap	0	3.83436824	0.855	0.223	0	7
Hist1h1b	0	3.30984762	0.713	0.1	0	7
H2afv1	0	3.11252553	0.976	0.253	0	7
Stmn1	0	3.10363525	0.971	0.229	0	7
Pclaf	0	3.07903682	0.845	0.136	0	7
Hmgb21	0	2.78745117	0.999	0.626	0	7
Myl41	0	2.75903912	0.806	0.043	0	7
Hmgb12	0	2.75296541	0.999	0.518	0	7
H2afx	0	2.55791168	0.69	0.114	0	7
Tuba1b1	0	2.44288892	0.869	0.206	0	7
Ptma1	0	2.42684055	0.999	0.678	0	7
Hmgn21	0	2.31101349	0.976	0.345	0	7
Hist1h1e	0	2.30239984	0.65	0.133	0	7
Tubb52	0	2.28418239	0.945	0.334	0	7
Dek1	0	2.1868519	0.918	0.281	0	7
Top2a1	0	2.17430853	0.777	0.161	0	7
H2afz1	0	2.17051462	0.994	0.556	0	7
Dut1	0	2.16893485	0.76	0.173	0	7
Elof11	0	2.14377851	0.795	0.124	0	7
Cbx3	0	2.12109335	0.945	0.313	0	7
Nusap1	0	2.02966879	0.609	0.114	0	7

Birc5	0	1.98655255	0.65	0.123	0	7
Vpreb31	0	1.9578705	0.99	0.157	0	7
Ube2s	0	1.94989806	0.834	0.282	0	7
Hist1h1a	0	1.94656471	0.456	0.03	0	7
Hist1h4d	0	1.91526318	0.551	0.085	0	7
Pafah1b31	0	1.87367497	0.827	0.148	0	7
Chchd101	0	1.86873797	0.826	0.12	0	7
Ucp22	0	1.8623407	0.909	0.325	0	7
Rrm2	0	1.84605727	0.537	0.073	0	7
Zfp7061	0	1.83979707	0.881	0.258	0	7
Hist1h3c	0	1.83779868	0.458	0.055	0	7
Pcna	0	1.82081036	0.691	0.166	0	7
Hist1h2br	0	1.82004933	0.45	0.056	0	7
Hist1h2bb	0	1.81022013	0.429	0.041	0	7
Hist1h1d	0	1.78147475	0.442	0.057	0	7
Ezh2	0	1.75458959	0.61	0.093	0	7
Ptprcap1	0	1.75158673	0.827	0.146	0	7
Hist1h4h	0	1.73082818	0.499	0.08	0	7
Ppia1	0	1.72755393	0.999	0.699	0	7
Pgls1	0	1.71937181	0.873	0.321	0	7
Hist1h3e	0	1.7189612	0.409	0.042	0	7
Alyref	0	1.7148745	0.74	0.202	0	7
Lockd	0	1.69447903	0.583	0.086	0	7
Snrpd11	0	1.69311678	0.797	0.235	0	7
Erh1	0	1.68815849	0.767	0.204	0	7
Uhrf1	0	1.68660476	0.536	0.067	0	7
Hist1h2bj	0	1.68461921	0.377	0.034	0	7
Gmnn	0	1.67663919	0.563	0.088	0	7
Cks1b1	0	1.67132687	0.672	0.157	0	7
Snrpg1	0	1.66701782	0.98	0.487	0	7
Anp32e1	0	1.66146249	0.719	0.178	0	7
Gm302111	0	1.65804446	0.515	0.042	0	7
Cnp1	0	1.64115125	0.584	0.065	0	7
Snrpe2	0	1.62884138	0.931	0.407	0	7
Selenoh	0	1.62697185	0.684	0.161	0	7
Dnajc9	0	1.61060676	0.527	0.078	0	7
Id3	0	1.60628648	0.591	0.067	0	7
Snu131	0	1.60102159	0.774	0.249	0	7
1810059H22	0	1.58997448	0.371	0.023	0	7
Pkig1	0	1.58956012	0.667	0.116	0	7
Hist1h2ab	0	1.57613432	0.382	0.036	0	7
Hist1h3b	0	1.56695652	0.368	0.034	0	7
Cbx1	0	1.55800106	0.664	0.157	0	7

Ebf11	0	1.55545923	0.974	0.186	0	7
Gm43305	0	1.55012046	0.339	0.039	0	7
Cd24a1	0	1.54273557	0.921	0.401	0	7
Lsm5	0	1.53574834	0.725	0.205	0	7
Sumo21	0	1.53147344	0.841	0.329	0	7
Gm499801	0	1.52526056	0.493	0.078	0	7
Lig1	0	1.52449558	0.483	0.083	0	7
Mcm7	0	1.52209543	0.565	0.118	0	7
Rbbp4	0	1.47356633	0.609	0.153	0	7
Slbp	0	1.46340731	0.556	0.126	0	7
Rpa3	0	1.46032419	0.546	0.11	0	7
Tmpo	0	1.45754069	0.618	0.151	0	7
Tipin	0	1.45712713	0.479	0.074	0	7
Tubb4b	0	1.44962394	0.593	0.166	0	7
Srsf31	0	1.43396063	0.87	0.374	0	7
H3f3a1	0	1.43126143	0.995	0.751	0	7
Lrmp1	0	1.42064113	0.532	0.081	0	7
Cdca3	0	1.41867952	0.422	0.069	0	7
Il7r1	0	1.4057678	0.506	0.054	0	7
Rpgrip1	0	1.40200668	0.506	0.105	0	7
Irf41	0	1.39900258	0.478	0.046	0	7
Gm472831	0	1.39632146	0.598	0.147	0	7
Bach21	0	1.39587699	0.52	0.06	0	7
Lgals9	0	1.39464621	0.566	0.12	0	7
Csrp2	0	1.390152	0.5	0.065	0	7
Ighm1	0	1.38932956	0.936	0.199	0	7
Spc24	0	1.38707043	0.434	0.066	0	7
Hist1h3f	0	1.35659148	0.328	0.035	0	7
Cenpw	0	1.35561927	0.486	0.097	0	7
Smc2	0	1.35462619	0.483	0.107	0	7
Maz	0	1.35405236	0.505	0.102	0	7
Bcl7a1	0	1.35362869	0.54	0.066	0	7
Hist1h4i	0	1.34377676	0.428	0.081	0	7
Cdca8	0	1.33738161	0.403	0.078	0	7
Cenps	0	1.3368608	0.382	0.038	0	7
Fcrla1	0	1.32890982	0.571	0.066	0	7
Snrpf2	0	1.32323338	0.895	0.367	0	7
H2afy1	0	1.31658155	0.72	0.221	0	7
Fbxo5	0	1.30451586	0.35	0.035	0	7
Herpud11	0	1.30382025	0.618	0.136	0	7
Dnmt1	0	1.29884225	0.449	0.094	0	7
Hist2h2ac	0	1.29239588	0.337	0.035	0	7
Mzb11	0	1.28959741	0.58	0.077	0	7

Cecr21	0	1.28937416	0.448	0.046	0	7
Myb1	0	1.27460348	0.527	0.095	0	7
Cenpm	0	1.25916848	0.343	0.033	0	7
Mki67	0	1.25856331	0.615	0.151	0	7
Rnf7	0	1.2566268	0.546	0.138	0	7
Hist1h2bm	0	1.25514194	0.287	0.025	0	7
Pbk	0	1.25394373	0.348	0.049	0	7
Cd79b1	0	1.25076309	0.788	0.14	0	7
Arpc5l1	0	1.24341196	0.573	0.147	0	7
Smarca41	0	1.24322075	0.566	0.122	0	7
Pou2af1	0	1.23403575	0.443	0.041	0	7
Ccna2	0	1.23329533	0.385	0.069	0	7
Dnajc71	0	1.21364338	0.751	0.187	0	7
Rhoh1	0	1.19496282	0.476	0.069	0	7
Akap121	0	1.17089305	0.45	0.057	0	7
Blnk1	0	1.16097245	0.449	0.06	0	7
Cd79a1	0	1.13960171	0.902	0.169	0	7
4930597A21	0	1.13200613	0.372	0.052	0	7
Ncapd2	0	1.13022511	0.343	0.05	0	7
Strbp	0	1.12768248	0.392	0.057	0	7
Wdr76	0	1.12628648	0.296	0.042	0	7
Rsph1	0	1.08239902	0.294	0.018	0	7
Hist1h2bn	0	1.07783877	0.251	0.021	0	7
Xrcc6	0	1.07628313	0.389	0.06	0	7
Tcf31	0	1.06970476	0.477	0.087	0	7
Cd721	0	1.05891104	0.528	0.072	0	7
Arntl	0	1.04035279	0.285	0.022	0	7
Clspn	0	1.02252693	0.274	0.034	0	7
Hes6	0	1.02194453	0.359	0.057	0	7
Tifa1	0	0.99717155	0.469	0.089	0	7
Slamf7	0	0.86843129	0.274	0.028	0	7
Spib1	0	0.83175045	0.389	0.058	0	7
Pax5	0	0.7477351	0.311	0.041	0	7
Nucks11	6.34E-307	1.28996428	0.699	0.215	2.05E-302	7
Ccne2	2.13E-304	1.2209586	0.311	0.047	6.88E-300	7
Uba521	3.49E-304	1.1130955	0.995	0.748	1.13E-299	7
Ccnb2	8.52E-303	1.40540477	0.403	0.08	2.75E-298	7
Nasp	1.46E-302	1.25645212	0.467	0.104	4.70E-298	7
Cox7a21	2.07E-302	1.32798005	0.918	0.487	6.67E-298	7
Lsm3	8.28E-301	1.25763907	0.538	0.139	2.67E-296	7
Esco2	1.11E-300	1.05738969	0.271	0.036	3.57E-296	7
Kif11	2.03E-299	1.17847574	0.356	0.062	6.55E-295	7
Ralgps2	7.54E-299	0.89600514	0.398	0.072	2.43E-294	7

Cks21	6.27E-298	1.49526907	0.7	0.238	2.03E-293	7
Cplx2	2.95E-297	0.85069224	0.276	0.035	9.51E-293	7
Fam53b	9.25E-295	0.88869817	0.352	0.058	2.99E-290	7
Cenpa	1.68E-289	1.65876532	0.497	0.124	5.44E-285	7
Glcci1	6.85E-289	0.88498936	0.275	0.037	2.21E-284	7
Arl5c1	9.78E-287	1.01049737	0.406	0.077	3.16E-282	7
Cox8a1	4.40E-286	1.1308843	0.97	0.673	1.42E-281	7
Nsmce4a	7.43E-284	1.150052	0.454	0.104	2.40E-279	7
Tmsb101	1.69E-282	1.16287165	1	0.737	5.45E-278	7
Cxcr41	2.34E-276	1.03495986	0.665	0.202	7.56E-272	7
Gtf2a2	2.89E-273	1.17526008	0.497	0.128	9.32E-269	7
Rfc3	5.67E-271	1.13843134	0.398	0.082	1.83E-266	7
Rpa2	4.01E-270	0.97249904	0.28	0.043	1.30E-265	7
Rfc4	4.08E-270	0.99427992	0.291	0.046	1.32E-265	7
Cdk1	1.71E-269	1.25780063	0.4	0.086	5.52E-265	7
Asf1b	2.89E-267	0.93474711	0.267	0.039	9.34E-263	7
Supt16	1.26E-266	1.20903869	0.544	0.152	4.07E-262	7
Ndufa4	2.29E-265	1.2314595	0.895	0.447	7.39E-261	7
Cmtm71	5.60E-265	1.08976439	0.677	0.22	1.81E-260	7
Ranbp11	4.60E-262	1.22372524	0.672	0.222	1.49E-257	7
Stambpl1	7.47E-261	0.81471852	0.307	0.05	2.41E-256	7
Hmga1	4.24E-260	0.96966815	0.396	0.082	1.37E-255	7
Nol72	1.24E-256	1.16864085	0.713	0.252	3.99E-252	7
Mrpl18	1.25E-255	1.14281816	0.501	0.135	4.03E-251	7
Oaz11	4.65E-254	1.06126013	0.955	0.627	1.50E-249	7
Cox5b1	4.09E-253	1.16823881	0.894	0.455	1.32E-248	7
Cmc2	4.33E-252	1.00684911	0.369	0.077	1.40E-247	7
Crip11	1.81E-248	0.75019831	0.905	0.378	5.85E-244	7
Lsm61	4.08E-247	1.15517897	0.623	0.207	1.32E-242	7
Slc1a5	3.23E-245	0.90013821	0.334	0.062	1.04E-240	7
Hmgn11	3.33E-243	1.07672227	0.696	0.229	1.08E-238	7
Larp7	6.19E-241	1.06447621	0.414	0.098	2.00E-236	7
Ndufb1-ps2	2.97E-240	1.02204412	0.956	0.624	9.59E-236	7
Nsd2	3.77E-238	0.92555528	0.363	0.076	1.22E-233	7
Calm21	1.75E-236	1.26730359	0.887	0.47	5.64E-232	7
Pttg1	2.56E-236	1.27271459	0.462	0.123	8.28E-232	7
Ran1	7.17E-236	1.10024597	0.81	0.336	2.32E-231	7
Cisd1	5.57E-235	0.96459156	0.37	0.08	1.80E-230	7
Srsf10	8.34E-235	1.04140006	0.505	0.142	2.69E-230	7
Fxn	1.54E-233	0.80063068	0.255	0.04	4.96E-229	7
Srsf7	2.56E-233	1.17289766	0.609	0.206	8.25E-229	7
B3gnt2	6.50E-233	0.98542918	0.436	0.11	2.10E-228	7
Snrpb1	1.05E-232	1.09936529	0.701	0.26	3.40E-228	7

Mcm6	2.12E-232	1.0641211	0.367	0.08	6.85E-228	7
Bub3	6.86E-232	1.21252775	0.429	0.112	2.22E-227	7
Cenpx	2.32E-231	1.07799715	0.451	0.121	7.51E-227	7
Cdkn31	1.42E-230	1.24854739	0.297	0.056	4.59E-226	7
Bloc1s2	1.82E-230	1.08499229	0.429	0.108	5.88E-226	7
Dtymk	4.91E-230	1.06145727	0.486	0.135	1.58E-225	7
Calm3	4.57E-229	1.1464002	0.65	0.239	1.48E-224	7
Nrm	5.34E-229	0.9157211	0.274	0.047	1.72E-224	7
Hmgb3	2.26E-228	1.00289446	0.349	0.074	7.29E-224	7
Grk22	7.48E-228	1.14279569	0.678	0.255	2.42E-223	7
Rbm32	5.81E-227	1.15820772	0.842	0.41	1.88E-222	7
Mcm5	1.41E-226	0.95674124	0.311	0.061	4.55E-222	7
Rrm1	2.36E-225	0.92001537	0.357	0.078	7.63E-221	7
Coq71	2.74E-223	0.85808963	0.361	0.077	8.86E-219	7
Dck	3.88E-223	0.86767725	0.361	0.078	1.25E-218	7
Gclc	7.14E-222	0.80844661	0.259	0.042	2.31E-217	7
Mcm3	1.37E-220	0.97963944	0.31	0.061	4.43E-216	7
Suz12	1.01E-219	0.95169062	0.38	0.089	3.27E-215	7
Rps202	1.24E-219	0.9347221	0.995	0.734	4.01E-215	7
Dusp21	2.00E-219	0.81238495	0.375	0.082	6.45E-215	7
Ndufs8	6.69E-219	0.97834931	0.525	0.159	2.16E-214	7
Phf5a	1.83E-218	1.01658193	0.462	0.129	5.91E-214	7
Prdx4	1.34E-217	0.83152324	0.281	0.051	4.33E-213	7
Hes1	4.06E-216	1.17506686	0.329	0.067	1.31E-211	7
Anp32b1	1.43E-213	1.08803669	0.701	0.274	4.63E-209	7
Ssna1	8.04E-213	1.02484114	0.438	0.122	2.60E-208	7
Zfpm1	9.15E-213	0.75278735	0.255	0.042	2.96E-208	7
Ptp4a3	1.14E-212	0.81994161	0.395	0.093	3.67E-208	7
Cyfip21	2.66E-211	1.08413454	0.466	0.136	8.60E-207	7
Banf11	1.52E-209	0.98886358	0.564	0.182	4.90E-205	7
Dctpp1	1.82E-209	1.00737542	0.4	0.101	5.87E-205	7
Ppp1cc	1.37E-208	1.11199201	0.511	0.162	4.43E-204	7
Desi1	6.00E-208	0.83723797	0.264	0.047	1.94E-203	7
Atp5j	9.24E-208	1.08600855	0.809	0.398	2.98E-203	7
Ube2a	2.34E-206	1.04844404	0.519	0.169	7.54E-202	7
Atp5g21	2.86E-205	1.04762092	0.831	0.398	9.23E-201	7
Sec11c1	3.17E-205	1.13896696	0.705	0.295	1.02E-200	7
Nup210	6.97E-205	0.81299383	0.282	0.055	2.25E-200	7
Kif15	2.39E-204	0.85365745	0.253	0.045	7.71E-200	7
Ctcf	3.71E-204	1.01539578	0.433	0.119	1.20E-199	7
E2f2	1.04E-201	0.86435588	0.306	0.064	3.37E-197	7
Usp1	2.69E-201	0.89187912	0.341	0.078	8.68E-197	7
Rpl392	4.00E-201	0.86000757	0.998	0.84	1.29E-196	7

Ndufs5	1.45E-200	1.05138988	0.665	0.27	4.67E-196	7
Lsm4	1.62E-200	1.01402832	0.59	0.21	5.23E-196	7
Hnrnpa31	2.26E-200	1.06653602	0.776	0.355	7.28E-196	7
Gngt2	9.82E-200	0.63596606	0.301	0.059	3.17E-195	7
Atp5h	3.36E-199	1.0382498	0.855	0.46	1.09E-194	7
Cd19	3.70E-199	0.56636631	0.253	0.043	1.19E-194	7
Sgo1	3.75E-199	0.92788171	0.297	0.061	1.21E-194	7
Uqcr10	2.29E-198	1.02837577	0.787	0.374	7.38E-194	7
Klhl6	5.05E-196	0.7862483	0.281	0.055	1.63E-191	7
Ddx39	7.50E-195	0.92581058	0.388	0.101	2.42E-190	7
Hnrnpd	1.80E-194	1.00969109	0.537	0.182	5.82E-190	7
Tra2b1	6.41E-194	1.0142951	0.635	0.244	2.07E-189	7
Jund1	1.38E-193	0.91350258	0.913	0.497	4.46E-189	7
Wtap	6.58E-192	0.94984069	0.445	0.131	2.13E-187	7
Nxt1	3.89E-191	0.87741581	0.275	0.056	1.26E-186	7
Exosc8	1.22E-189	0.90644517	0.349	0.086	3.95E-185	7
H3f3b2	1.25E-189	0.85144128	0.992	0.819	4.05E-185	7
Myef2	7.36E-189	0.82620112	0.365	0.088	2.38E-184	7
Sf3b5	3.26E-188	0.96927229	0.557	0.197	1.05E-183	7
Hnrnpab1	5.54E-188	0.96450758	0.657	0.251	1.79E-183	7
Camk2d	5.64E-188	0.73464301	0.357	0.083	1.82E-183	7
Siva1	1.46E-187	1.13625694	0.463	0.147	4.72E-183	7
Plekha21	1.51E-187	0.7878889	0.358	0.086	4.88E-183	7
Clic1	1.73E-187	1.01492648	0.858	0.456	5.59E-183	7
Snx2	1.18E-186	0.73419971	0.331	0.076	3.82E-182	7
Psmb9	3.47E-186	0.95724601	0.471	0.149	1.12E-181	7
Ndufa111	1.38E-185	0.94088911	0.632	0.245	4.47E-181	7
Hist1h2bc	5.26E-185	1.10877983	0.457	0.138	1.70E-180	7
U2af1	1.33E-184	0.95902654	0.574	0.208	4.31E-180	7
Atp5g3	2.08E-184	0.97754202	0.642	0.255	6.72E-180	7
Ndufb111	1.80E-182	0.99242321	0.697	0.308	5.83E-178	7
Hnrnpa12	1.37E-180	0.91909831	0.667	0.259	4.44E-176	7
Snrpd21	1.73E-179	0.93023047	0.69	0.284	5.59E-175	7
Rbm15	2.22E-176	0.91072369	0.294	0.067	7.18E-172	7
Smc41	4.29E-176	0.89344552	0.533	0.181	1.38E-171	7
Micos101	1.83E-175	0.92930564	0.635	0.255	5.89E-171	7
Ubl51	1.00E-174	0.98116216	0.855	0.506	3.24E-170	7
Ccdc34	3.15E-174	0.88335601	0.385	0.106	1.02E-169	7
Rpl291	6.62E-174	0.89212258	0.922	0.561	2.14E-169	7
Slc16a1	9.76E-174	0.70778017	0.27	0.055	3.15E-169	7
Polr2j	2.07E-173	0.8950817	0.492	0.167	6.68E-169	7
Spc25	5.35E-173	0.93530443	0.258	0.054	1.73E-168	7
Sec631	8.85E-172	0.73082986	0.419	0.118	2.86E-167	7

Atp5mpl1	1.79E-171	0.9383501	0.755	0.359	5.78E-167	7
Sae1	9.15E-171	0.72387411	0.294	0.067	2.95E-166	7
Cycs1	1.07E-170	0.95605483	0.583	0.227	3.44E-166	7
Atp5j21	1.94E-170	0.90940479	0.886	0.528	6.25E-166	7
Ubald2	2.98E-169	1.00317878	0.511	0.182	9.63E-165	7
Tacc3	1.95E-168	0.85186988	0.251	0.052	6.29E-164	7
Hmgn5	3.44E-168	0.80669446	0.275	0.061	1.11E-163	7
Rpl282	2.27E-167	0.7758108	0.992	0.751	7.32E-163	7
Smdt12	3.47E-167	0.95137827	0.714	0.324	1.12E-162	7
Tmem243	1.69E-166	0.689036	0.334	0.084	5.45E-162	7
Nudc	4.71E-166	0.83207309	0.411	0.122	1.52E-161	7
Timm13	9.13E-165	0.88928368	0.575	0.221	2.95E-160	7
Hnrnpu1	3.11E-164	0.87313427	0.684	0.289	1.00E-159	7
Csrp1	1.50E-163	0.79064679	0.306	0.075	4.84E-159	7
Cox5a1	2.71E-163	0.93547435	0.762	0.374	8.75E-159	7
Srsf21	1.91E-162	0.96461819	0.603	0.246	6.16E-158	7
Racgap1	1.25E-161	0.883631	0.29	0.069	4.04E-157	7
Cenpf	1.53E-161	1.06696093	0.327	0.085	4.95E-157	7
Cdca7	1.52E-160	0.82790271	0.27	0.061	4.90E-156	7
Exosc3	2.80E-160	0.75259617	0.292	0.07	9.05E-156	7
Hnrnpul1	3.67E-160	0.86773595	0.464	0.156	1.19E-155	7
Rbx11	5.20E-160	0.92358688	0.738	0.359	1.68E-155	7
Foxp11	1.38E-159	0.69689951	0.575	0.207	4.45E-155	7
Serinc31	7.40E-159	0.8039903	0.742	0.344	2.39E-154	7
Mef2c1	1.40E-158	0.57184245	0.458	0.143	4.51E-154	7
Mrps14	2.83E-158	0.86648356	0.497	0.179	9.13E-154	7
Churc1	9.22E-158	0.83191567	0.396	0.12	2.98E-153	7
Med30	2.62E-157	0.94902862	0.351	0.101	8.45E-153	7
Ndufab1	5.57E-157	0.87831622	0.523	0.192	1.80E-152	7
Pim12	9.68E-157	0.82105107	0.73	0.323	3.13E-152	7
Nono	1.68E-156	0.83709031	0.459	0.153	5.44E-152	7
Ikzf1	5.29E-156	0.88104823	0.415	0.132	1.71E-151	7
Cdk4	2.00E-155	0.85490288	0.419	0.131	6.47E-151	7
Lsm2	3.59E-155	0.81927843	0.372	0.109	1.16E-150	7
Hnrnpa02	1.91E-154	0.80826478	0.656	0.276	6.16E-150	7
Stag2	7.01E-154	0.94353639	0.443	0.151	2.26E-149	7
Tex30	1.05E-153	0.75704381	0.254	0.056	3.39E-149	7
Sf3b6	3.77E-153	0.89092992	0.602	0.25	1.22E-148	7
Naca1	5.68E-153	0.7881584	0.933	0.558	1.83E-148	7
Lsm8	3.16E-152	0.81034676	0.409	0.131	1.02E-147	7
Cox6c2	5.57E-152	0.79432685	0.922	0.627	1.80E-147	7
Orc6	6.44E-152	0.75257511	0.304	0.077	2.08E-147	7
Anapc5	1.16E-151	0.80079836	0.355	0.102	3.76E-147	7

C1galt11	1.22E-151	0.68197255	0.32	0.081	3.95E-147	7
Cdc42ep3	1.78E-151	0.77204719	0.298	0.075	5.74E-147	7
Sarnp	3.58E-151	0.90889281	0.577	0.234	1.16E-146	7
Ubb1	3.68E-151	0.87150597	0.963	0.752	1.19E-146	7
Hnrnpf1	7.07E-151	0.88169879	0.679	0.305	2.28E-146	7
Mrps36	9.84E-151	0.76901878	0.389	0.117	3.18E-146	7
Snrpa1	1.68E-150	0.77777318	0.317	0.085	5.41E-146	7
Paip21	2.92E-150	0.78602876	0.605	0.245	9.44E-146	7
Dbf4	3.05E-149	0.8319671	0.28	0.069	9.85E-145	7
Ewsr1	3.90E-149	0.77997557	0.446	0.151	1.26E-144	7
Lsm7	4.53E-149	0.8402371	0.429	0.141	1.46E-144	7
Chchd21	1.54E-148	0.79510817	0.889	0.569	4.97E-144	7
Atp5e2	1.55E-148	0.74434105	0.953	0.687	4.99E-144	7
Scaf11	2.19E-148	0.72933296	0.516	0.186	7.06E-144	7
Cox6b11	1.93E-147	0.85665484	0.837	0.483	6.23E-143	7
Psmb12	2.36E-147	0.82518228	0.656	0.289	7.61E-143	7
Atp5k2	3.56E-147	0.84494408	0.858	0.495	1.15E-142	7
Mrpl34	1.21E-146	0.74603426	0.456	0.157	3.89E-142	7
Nop102	1.45E-146	0.83660764	0.598	0.238	4.68E-142	7
Fau1	2.67E-146	0.51108169	0.998	0.94	8.60E-142	7
Nedd81	4.68E-146	0.85036687	0.705	0.34	1.51E-141	7
Thoc7	8.04E-146	0.77662417	0.507	0.185	2.60E-141	7
Ndufc11	2.03E-145	0.87285006	0.677	0.311	6.55E-141	7
Rbmxl1	2.60E-145	0.71098656	0.266	0.064	8.38E-141	7
Rps292	2.33E-144	0.61278196	0.999	0.953	7.54E-140	7
Rad21	2.55E-144	0.83898859	0.381	0.118	8.24E-140	7
Pin1	5.02E-144	0.74923876	0.32	0.089	1.62E-139	7
Erp291	6.44E-144	0.73662243	0.532	0.198	2.08E-139	7
Dok31	7.09E-144	0.81460981	0.444	0.154	2.29E-139	7
Nsmce1	1.52E-143	0.79606873	0.318	0.088	4.92E-139	7
Sumo1	1.90E-143	0.79827543	0.532	0.209	6.15E-139	7
Hnrnpl1	3.14E-143	0.85489682	0.537	0.213	1.02E-138	7
Hint11	3.15E-143	0.80636032	0.807	0.39	1.02E-138	7
Atad2	3.61E-143	0.77618212	0.343	0.099	1.17E-138	7
Eny2	7.40E-143	0.80685326	0.484	0.174	2.39E-138	7
Rps15a1	6.54E-142	0.65361302	0.997	0.778	2.11E-137	7
Atp1b11	1.07E-141	0.53281186	0.3	0.074	3.46E-137	7
Hnrnpdl	1.14E-141	0.80050228	0.483	0.178	3.67E-137	7
Nudt21	1.39E-141	0.76205531	0.413	0.135	4.48E-137	7
Ddx39b	1.82E-141	0.79141421	0.468	0.168	5.88E-137	7
Raly	9.19E-141	0.82564765	0.52	0.203	2.97E-136	7
Hnrnpa2b12	1.27E-140	0.80699966	0.775	0.398	4.09E-136	7
Hpf1	1.33E-140	0.81680299	0.353	0.107	4.31E-136	7

Frg1	2.39E-140	0.83009928	0.431	0.149	7.72E-136	7
Hnrnpm1	3.93E-140	0.8113796	0.642	0.282	1.27E-135	7
Rpl191	4.57E-140	0.68569236	0.99	0.752	1.47E-135	7
Mdh1	1.60E-139	0.74415363	0.393	0.127	5.16E-135	7
Sap18	6.12E-138	0.85962526	0.532	0.219	1.98E-133	7
Tspan13	1.32E-137	0.5381235	0.33	0.09	4.26E-133	7
Mrpl57	1.34E-137	0.83414637	0.467	0.174	4.34E-133	7
Elob1	7.58E-137	0.7858593	0.882	0.553	2.45E-132	7
Tram1	1.02E-136	0.73132774	0.443	0.156	3.28E-132	7
Tomm7	1.93E-136	0.8242484	0.822	0.469	6.23E-132	7
Jpt11	6.75E-136	0.96194681	0.537	0.221	2.18E-131	7
Psma6	2.46E-135	0.75960597	0.429	0.151	7.93E-131	7
Hist2h2aa12	4.78E-135	0.99602977	0.375	0.122	1.54E-130	7
Smc6	4.45E-134	0.79195458	0.396	0.131	1.44E-129	7
Ndufa22	4.90E-133	0.78571501	0.791	0.421	1.58E-128	7
Hells	8.28E-133	0.64189163	0.297	0.08	2.67E-128	7
Trmt1121	1.67E-132	0.74214729	0.563	0.229	5.39E-128	7
Psip1	1.85E-132	0.63058136	0.353	0.106	5.96E-128	7
Topbp1	2.56E-132	0.68354657	0.276	0.072	8.27E-128	7
Ube2i1	1.69E-131	0.78762859	0.557	0.232	5.44E-127	7
Apobec3	3.37E-130	0.65082264	0.279	0.073	1.09E-125	7
Ndufc2	4.15E-130	0.71148759	0.533	0.215	1.34E-125	7
Anp32a	7.83E-130	0.83120599	0.595	0.269	2.53E-125	7
Ddt	1.09E-129	0.6009843	0.267	0.069	3.53E-125	7
Tmem131l	8.85E-129	0.67094993	0.298	0.083	2.86E-124	7
Tpx2	4.10E-128	0.83078471	0.294	0.083	1.32E-123	7
Rbm17	5.75E-127	0.74047053	0.33	0.1	1.86E-122	7
Clta2	1.18E-126	0.65236336	0.668	0.306	3.80E-122	7
Nsa21	1.66E-125	0.67041574	0.861	0.466	5.38E-121	7
Snrnp40	1.73E-125	0.6474937	0.257	0.067	5.57E-121	7
Ube2c	1.94E-125	1.3831878	0.404	0.147	6.26E-121	7
Emc6	3.58E-125	0.68559681	0.369	0.121	1.16E-120	7
Ssrp1	3.80E-124	0.64952901	0.341	0.105	1.23E-119	7
Hprt	4.69E-124	0.73133037	0.349	0.113	1.51E-119	7
Rtraf	7.95E-124	0.73231496	0.512	0.204	2.57E-119	7
Ndufb83	1.40E-123	0.72198175	0.616	0.282	4.51E-119	7
Rps52	1.50E-123	0.66989543	0.97	0.672	4.86E-119	7
Polr2i	2.18E-123	0.7307978	0.385	0.132	7.04E-119	7
4930523C07I	4.41E-123	0.64732543	0.304	0.088	1.42E-118	7
Pds5a	1.58E-122	0.70947232	0.305	0.09	5.09E-118	7
Ube2n	2.23E-122	0.72559185	0.374	0.126	7.19E-118	7
Ndufb10	3.68E-122	0.72947944	0.544	0.231	1.19E-117	7
Rpl111	5.34E-122	0.63962808	0.982	0.731	1.72E-117	7

Taf10	5.39E-122	0.73747035	0.524	0.219	1.74E-117	7
Atp5o1	9.53E-122	0.74216575	0.545	0.234	3.08E-117	7
Eif4a3	2.11E-121	0.72411531	0.321	0.1	6.81E-117	7
Pgp1	3.59E-121	0.75311999	0.292	0.086	1.16E-116	7
Magoh3	2.28E-120	0.83684264	0.553	0.253	7.36E-116	7
Arl6ip1	2.46E-120	0.95760729	0.482	0.199	7.95E-116	7
Rps27l1	3.68E-120	0.74309211	0.693	0.333	1.19E-115	7
Mrps21	9.02E-120	0.78325315	0.629	0.309	2.91E-115	7
Blvrb1	1.15E-119	0.5111312	0.422	0.146	3.70E-115	7
Nap1l12	5.91E-119	0.65119456	0.654	0.294	1.91E-114	7
Mrpl20	7.44E-119	0.724807	0.47	0.188	2.40E-114	7
Atp5g1	2.25E-118	0.75286553	0.608	0.272	7.25E-114	7
Emg1	4.63E-118	0.68083717	0.4	0.142	1.49E-113	7
Psmc3	4.72E-118	0.72175954	0.452	0.174	1.52E-113	7
Ctbp1	6.17E-118	0.69640346	0.382	0.134	1.99E-113	7
Psmb6	8.53E-118	0.74788927	0.519	0.22	2.75E-113	7
Yeats4	2.40E-117	0.71892602	0.368	0.127	7.76E-113	7
Dazap1	8.52E-117	0.62695419	0.288	0.084	2.75E-112	7
Rcc2	9.48E-117	0.58635513	0.315	0.096	3.06E-112	7
Srsf1	9.83E-117	0.66305041	0.348	0.114	3.17E-112	7
Trim28	1.28E-116	0.66661197	0.328	0.103	4.12E-112	7
Rps271	2.16E-116	0.37656887	0.999	0.908	6.97E-112	7
Psmc1	3.26E-116	0.73130796	0.364	0.124	1.05E-111	7
Cdkn2d	5.18E-115	0.99308766	0.387	0.146	1.67E-110	7
Csk	6.56E-115	0.55929292	0.354	0.116	2.12E-110	7
0610010K14l	1.23E-114	0.71009094	0.31	0.097	3.97E-110	7
Pdhb	1.90E-114	0.63715956	0.286	0.084	6.12E-110	7
mt-Nd4l1	2.56E-114	0.58745278	0.804	0.434	8.25E-110	7
Mrpl28	4.49E-114	0.6157354	0.303	0.093	1.45E-109	7
Pfdn6	5.36E-114	0.72448389	0.383	0.136	1.73E-109	7
Zfp91	6.96E-114	0.63904499	0.437	0.164	2.25E-109	7
Uchl3	1.69E-113	0.62361884	0.315	0.099	5.45E-109	7
Timm17b	1.76E-113	0.73637029	0.32	0.104	5.68E-109	7
Dleu2	2.17E-113	0.73901486	0.368	0.129	7.02E-109	7
Cirbp	4.39E-113	0.60059407	0.301	0.092	1.42E-108	7
Pa2g4	5.37E-113	0.66680501	0.368	0.126	1.73E-108	7
Polr2l	1.09E-112	0.72800421	0.355	0.122	3.52E-108	7
Sp3	2.76E-112	0.79151161	0.358	0.126	8.91E-108	7
Selenow1	5.30E-112	0.53646508	0.597	0.251	1.71E-107	7
Foxn3	1.27E-111	0.57468571	0.295	0.088	4.11E-107	7
Sec61g	1.41E-111	0.58844583	0.936	0.598	4.54E-107	7
Smarcc1	4.22E-111	0.61474377	0.314	0.097	1.36E-106	7
Smc3	4.58E-111	0.63372228	0.374	0.131	1.48E-106	7

Syk2	3.04E-110	0.62804085	0.448	0.174	9.83E-106	7
Pole4	4.39E-110	0.67980066	0.301	0.095	1.42E-105	7
Gtf2h5	3.71E-109	0.67035094	0.468	0.192	1.20E-104	7
Rpl36al2	3.88E-109	0.62434721	0.882	0.497	1.25E-104	7
Mrps33	5.84E-109	0.69221121	0.458	0.185	1.88E-104	7
Ndufb5	8.78E-109	0.67380259	0.511	0.218	2.83E-104	7
Tma7	1.87E-108	0.76332374	0.701	0.376	6.03E-104	7
Sdhb	6.66E-108	0.71149474	0.422	0.166	2.15E-103	7
Lbr2	1.29E-107	0.70227331	0.537	0.244	4.17E-103	7
Rpsa1	2.86E-107	0.62727811	0.972	0.692	9.23E-103	7
Gm11808	3.29E-107	0.63794483	0.421	0.162	1.06E-102	7
Uqcrq	4.92E-107	0.7307796	0.715	0.398	1.59E-102	7
Cpsf6	1.25E-106	0.56159907	0.269	0.078	4.03E-102	7
Psmb5	1.78E-106	0.66937789	0.502	0.217	5.75E-102	7
Mrpl42	4.12E-106	0.61038682	0.303	0.097	1.33E-101	7
Hist1h1c	6.80E-106	0.76743288	0.391	0.148	2.20E-101	7
Ndufs62	1.29E-104	0.67058743	0.542	0.247	4.17E-100	7
Pmf1	1.71E-104	0.48887813	0.28	0.085	5.52E-100	7
Ets1	2.11E-104	0.52745025	0.395	0.144	6.80E-100	7
Ndufb31	2.20E-104	0.72375975	0.524	0.237	7.12E-100	7
Ywhah1	2.44E-104	0.65677728	0.513	0.225	7.87E-100	7
Uqcrfs1	8.99E-104	0.67618036	0.413	0.162	2.90E-99	7
Tgfb1	9.34E-104	0.56038387	0.401	0.15	3.02E-99	7
Tyms	2.10E-103	0.73697162	0.253	0.075	6.77E-99	7
Smarca51	2.26E-103	0.66088733	0.543	0.243	7.30E-99	7
Mrps28	2.85E-103	0.57718714	0.262	0.078	9.20E-99	7
Top1	3.29E-103	0.74383505	0.666	0.352	1.06E-98	7
Fmr1	4.83E-103	0.58401061	0.256	0.074	1.56E-98	7
Psma21	1.17E-102	0.64248598	0.622	0.305	3.77E-98	7
Lmnb13	1.91E-102	0.47525471	0.604	0.283	6.17E-98	7
Snrpd31	2.32E-102	0.69934047	0.552	0.256	7.49E-98	7
Zcrb1	3.62E-102	0.71801756	0.433	0.176	1.17E-97	7
Smchd1	4.21E-102	0.58579893	0.331	0.112	1.36E-97	7
Rtf1	5.63E-102	0.64012134	0.399	0.152	1.82E-97	7
Polr2k1	7.95E-102	0.60592432	0.526	0.234	2.57E-97	7
Hnrnpk2	2.63E-101	0.61272661	0.713	0.372	8.49E-97	7
Smc1a	3.12E-101	0.6836767	0.374	0.139	1.01E-96	7
Pold4	4.93E-101	0.61782034	0.371	0.136	1.59E-96	7
Ube2e3	8.79E-101	0.66744245	0.254	0.075	2.84E-96	7
Grpel1	1.15E-100	0.58896884	0.339	0.12	3.70E-96	7
Rpl18a2	1.47E-100	0.53043902	0.995	0.8	4.76E-96	7
Rps163	1.01E-99	0.55014907	0.984	0.77	3.25E-95	7
Gm100762	2.77E-99	0.62692933	0.976	0.804	8.95E-95	7

Serbp11	5.82E-99	0.59241139	0.842	0.473	1.88E-94	7
Dpm3	6.18E-99	0.63831661	0.538	0.243	1.99E-94	7
Dyrk1a	6.79E-99	0.61973279	0.254	0.076	2.19E-94	7
Rfc2	3.72E-98	0.56873238	0.266	0.082	1.20E-93	7
Atpif1	7.08E-98	0.48552005	0.766	0.41	2.29E-93	7
Chchd11	1.17E-97	0.58400615	0.434	0.176	3.77E-93	7
Prc1	1.53E-97	0.67698315	0.279	0.089	4.95E-93	7
Lmo44	2.31E-97	0.72485541	0.527	0.249	7.47E-93	7
Ywhaq1	2.95E-97	0.56853099	0.465	0.194	9.52E-93	7
Rpl22l12	3.73E-97	0.68898821	0.828	0.51	1.20E-92	7
Arpp191	3.89E-97	0.65932028	0.639	0.329	1.26E-92	7
Nmt1	4.45E-97	0.64972681	0.457	0.198	1.44E-92	7
Eif3h2	5.71E-97	0.59509166	0.568	0.265	1.85E-92	7
Psmb2	6.81E-97	0.65800545	0.489	0.217	2.20E-92	7
Ndufa5	7.36E-97	0.58619587	0.463	0.196	2.38E-92	7
Ppp1ca2	1.52E-96	0.70095038	0.66	0.357	4.92E-92	7
Ndufa12	1.56E-96	0.62302484	0.382	0.146	5.04E-92	7
Mrps24	1.77E-96	0.63608662	0.406	0.162	5.70E-92	7
Znrd1	4.58E-96	0.61647657	0.315	0.109	1.48E-91	7
Rpl91	1.70E-95	0.47781654	0.978	0.766	5.49E-91	7
U2af2	2.58E-95	0.64223784	0.354	0.132	8.31E-91	7
Hnrnpr	2.83E-95	0.54276888	0.292	0.095	9.13E-91	7
Cux1	2.94E-95	0.56535204	0.361	0.134	9.50E-91	7
Mrpl51	3.92E-95	0.65647873	0.335	0.123	1.27E-90	7
Anapc13	7.37E-95	0.71727319	0.443	0.192	2.38E-90	7
Rps262	1.16E-94	0.55668886	0.993	0.78	3.75E-90	7
Ccnd31	4.82E-94	0.84661401	0.426	0.187	1.56E-89	7
Rps32	5.46E-94	0.55108178	0.961	0.698	1.76E-89	7
Ap2s1	7.26E-94	0.64388605	0.429	0.184	2.34E-89	7
Rnps1	8.23E-94	0.53123303	0.325	0.113	2.66E-89	7
Cdv3	9.73E-94	0.6258635	0.404	0.163	3.14E-89	7
Prpf4b2	1.21E-93	0.60761748	0.52	0.236	3.91E-89	7
Sox42	4.88E-93	0.47933817	0.4	0.149	1.58E-88	7
Tmco1	5.80E-93	0.6194263	0.38	0.15	1.87E-88	7
Srsf6	6.51E-93	0.57904531	0.389	0.153	2.10E-88	7
Arf6	7.24E-93	0.59805013	0.352	0.132	2.34E-88	7
Pcbd2	9.84E-93	0.69785639	0.296	0.104	3.18E-88	7
Mrpl43	1.20E-92	0.54665714	0.316	0.112	3.88E-88	7
2410006H16I	1.27E-92	0.44873952	0.745	0.385	4.10E-88	7
Fkbp2	3.99E-92	0.69409093	0.377	0.15	1.29E-87	7
Prdx12	1.07E-91	0.4866739	0.76	0.379	3.44E-87	7
Rps112	2.32E-91	0.5210463	0.971	0.721	7.48E-87	7
Ifi27l2a2	3.15E-91	0.614015	0.459	0.19	1.02E-86	7

AI662270	4.88E-91	0.56318391	0.348	0.131	1.57E-86	7
Dcp2	8.74E-91	0.58945644	0.253	0.08	2.82E-86	7
Ndufaf8	1.24E-90	0.56155338	0.31	0.11	4.01E-86	7
Cdc26	1.25E-90	0.55538874	0.285	0.096	4.04E-86	7
Supt4a2	1.62E-90	0.59503473	0.556	0.272	5.22E-86	7
Polr2g	2.78E-90	0.56881394	0.313	0.112	8.96E-86	7
Mrpl54	3.09E-90	0.5075125	0.354	0.133	9.98E-86	7
Psmb31	5.46E-90	0.64579987	0.564	0.286	1.76E-85	7
Cbx5	1.39E-89	0.4962207	0.305	0.104	4.50E-85	7
Snhg9	1.75E-89	0.62018731	0.347	0.131	5.65E-85	7
Slc38a21	1.75E-89	0.53074901	0.684	0.359	5.65E-85	7
Abhd17a	4.52E-89	0.51720987	0.281	0.094	1.46E-84	7
Map3k1	5.95E-89	0.45179626	0.264	0.084	1.92E-84	7
Top2b	8.72E-89	0.5270323	0.331	0.12	2.82E-84	7
Dhx9	1.60E-88	0.53202359	0.341	0.126	5.15E-84	7
Marcks3	1.60E-88	0.43107885	0.461	0.188	5.17E-84	7
Rps191	2.10E-88	0.49594069	0.982	0.722	6.78E-84	7
Cnot6	3.56E-88	0.50854634	0.267	0.086	1.15E-83	7
Ptbp1	7.57E-88	0.54974267	0.314	0.113	2.44E-83	7
Cox6a11	1.74E-87	0.60118559	0.598	0.306	5.63E-83	7
Rps72	2.25E-87	0.47531074	0.98	0.721	7.27E-83	7
Psmc4	2.84E-87	0.51870823	0.265	0.087	9.18E-83	7
Dcun1d5	4.01E-87	0.61483242	0.422	0.181	1.30E-82	7
Rbbp7	5.60E-87	0.54371148	0.346	0.13	1.81E-82	7
U2surp	6.04E-87	0.53649737	0.363	0.141	1.95E-82	7
Rps242	9.65E-86	0.41575386	0.998	0.85	3.12E-81	7
Trir1	1.49E-85	0.59689958	0.534	0.261	4.81E-81	7
Snhg1	2.18E-85	0.57019092	0.457	0.2	7.05E-81	7
Ilkap	4.58E-85	0.50372381	0.25	0.08	1.48E-80	7
Uqcrh1	1.06E-84	0.53794337	0.815	0.502	3.41E-80	7
Bola3	2.07E-84	0.4874667	0.314	0.114	6.70E-80	7
Arglu1	4.92E-84	0.60833385	0.437	0.192	1.59E-79	7
Rpl272	7.12E-84	0.4892509	0.966	0.715	2.30E-79	7
Eif5a1	8.29E-84	0.56838349	0.713	0.396	2.68E-79	7
Ndufb71	8.46E-84	0.62600617	0.662	0.37	2.73E-79	7
Rpl35a2	8.54E-84	0.45503134	0.997	0.851	2.76E-79	7
Abracl	9.92E-84	0.6055533	0.544	0.269	3.20E-79	7
Mrpl522	1.49E-83	0.4969412	0.605	0.29	4.81E-79	7
Rpl212	2.32E-83	0.48553989	0.976	0.71	7.50E-79	7
Eif3f1	4.40E-83	0.50009813	0.571	0.279	1.42E-78	7
Eif3k1	4.53E-83	0.5588851	0.591	0.299	1.46E-78	7
Rbm38	5.67E-83	0.51555624	0.361	0.143	1.83E-78	7
Rbm8a1	7.76E-83	0.53254407	0.477	0.217	2.51E-78	7

Fam133b	7.85E-83	0.58095282	0.311	0.116	2.53E-78	7
Rpl322	8.66E-83	0.50894755	0.988	0.721	2.80E-78	7
Srrm1	1.16E-82	0.50605022	0.409	0.173	3.73E-78	7
1110004F10I	2.48E-82	0.5257724	0.414	0.175	8.01E-78	7
Atp5md2	2.67E-82	0.53929191	0.833	0.528	8.61E-78	7
Sf1	3.12E-82	0.56672401	0.35	0.139	1.01E-77	7
Mcmbp	4.07E-82	0.62099378	0.257	0.088	1.31E-77	7
Cd371	8.11E-82	0.42443308	0.424	0.183	2.62E-77	7
Med21	1.17E-81	0.53072197	0.321	0.122	3.77E-77	7
Atp6v1f1	2.72E-81	0.53178501	0.595	0.313	8.79E-77	7
Snrpc	5.69E-81	0.54030521	0.39	0.165	1.84E-76	7
Pbrm1	5.79E-81	0.52962495	0.383	0.157	1.87E-76	7
Thrap3	6.85E-81	0.50665282	0.334	0.129	2.21E-76	7
Tut4	1.48E-80	0.40518877	0.296	0.105	4.76E-76	7
Ciao2a	2.17E-80	0.5955841	0.341	0.138	7.00E-76	7
Nfkbia2	6.77E-80	0.57612909	0.488	0.23	2.19E-75	7
Bcl7c	1.69E-79	0.48761218	0.253	0.086	5.45E-75	7
Mrpl12	1.93E-79	0.55578668	0.272	0.096	6.22E-75	7
Abcf1	2.41E-79	0.5352414	0.371	0.151	7.79E-75	7
Srp9	2.42E-79	0.58908883	0.511	0.253	7.83E-75	7
Brd8	3.48E-79	0.47903187	0.252	0.084	1.12E-74	7
Srp141	3.59E-79	0.48954454	0.551	0.276	1.16E-74	7
Clec2d	4.91E-79	0.44418569	0.263	0.089	1.58E-74	7
Eif1b	1.03E-78	0.66170875	0.31	0.121	3.34E-74	7
Cetn3	1.04E-78	0.5542262	0.373	0.156	3.37E-74	7
Khsrp	1.05E-78	0.62147978	0.289	0.106	3.38E-74	7
Dynll11	1.30E-78	0.6064686	0.749	0.441	4.20E-74	7
Pdap11	2.58E-78	0.43542643	0.554	0.266	8.33E-74	7
Rps131	2.64E-78	0.43980883	0.978	0.789	8.54E-74	7
Akr1b3	3.14E-78	0.47424667	0.253	0.085	1.01E-73	7
Sf3b4	9.08E-78	0.47558645	0.26	0.089	2.93E-73	7
Tardbp	2.91E-77	0.45426169	0.351	0.139	9.41E-73	7
Pdcd51	3.61E-77	0.49929699	0.46	0.213	1.16E-72	7
Psmb8	3.86E-77	0.45530915	0.457	0.206	1.25E-72	7
Llph	3.88E-77	0.52605387	0.426	0.189	1.25E-72	7
Park7	1.05E-76	0.50486576	0.475	0.222	3.40E-72	7
Srsf11	1.10E-76	0.49875617	0.379	0.16	3.56E-72	7
Mob4	1.57E-76	0.46347877	0.322	0.126	5.08E-72	7
Set1	2.10E-76	0.43409143	0.669	0.337	6.78E-72	7
Stk4	3.10E-76	0.47729751	0.309	0.118	1.00E-71	7
Nme11	3.30E-76	0.56476357	0.487	0.229	1.06E-71	7
Polr2m	3.33E-76	0.48112793	0.253	0.086	1.07E-71	7
Pfdn2	5.37E-76	0.54500808	0.366	0.155	1.73E-71	7

Rps3a12	6.03E-76	0.47219489	0.986	0.767	1.95E-71	7
Dnajc8	1.73E-75	0.47909169	0.386	0.165	5.59E-71	7
Elf2	2.06E-75	0.48981084	0.253	0.087	6.66E-71	7
Atp5c11	2.10E-75	0.5210996	0.534	0.268	6.77E-71	7
Uqcrb1	2.37E-75	0.5602202	0.59	0.312	7.65E-71	7
Srsf9	3.12E-75	0.54426189	0.406	0.182	1.01E-70	7
Ccdc124	7.18E-75	0.51889051	0.27	0.098	2.32E-70	7
Tomm61	8.84E-75	0.53850215	0.697	0.408	2.86E-70	7
Med28	9.72E-75	0.53657372	0.274	0.101	3.14E-70	7
Atp5l1	1.39E-74	0.52139308	0.861	0.599	4.48E-70	7
Ppm1g	4.46E-74	0.50819795	0.253	0.089	1.44E-69	7
Edf11	9.97E-74	0.57887007	0.564	0.302	3.22E-69	7
Tomm22	1.12E-73	0.53178704	0.445	0.212	3.61E-69	7
Tceal9	1.41E-73	0.46213148	0.349	0.142	4.54E-69	7
Rpl413	1.50E-73	0.52200401	0.995	0.898	4.84E-69	7
Rpl342	1.57E-73	0.42863039	0.99	0.82	5.08E-69	7
Commd1	3.29E-73	0.59602858	0.33	0.138	1.06E-68	7
Ywhae1	1.28E-72	0.46017336	0.638	0.34	4.12E-68	7
Smarce1	1.50E-72	0.47339649	0.265	0.095	4.84E-68	7
Rfc1	2.36E-72	0.50499948	0.252	0.089	7.61E-68	7
Fkbp31	4.66E-72	0.44030853	0.376	0.159	1.51E-67	7
Fus2	5.12E-72	0.5109935	0.5	0.246	1.65E-67	7
Dynlt1f	5.21E-72	0.53837417	0.266	0.099	1.68E-67	7
Uqcr111	7.86E-72	0.57111543	0.618	0.35	2.54E-67	7
Rps182	8.40E-72	0.50148361	0.938	0.639	2.71E-67	7
Pnn	8.95E-72	0.44678997	0.27	0.098	2.89E-67	7
Tagln21	1.49E-71	0.40994275	0.594	0.296	4.83E-67	7
Slc25a51	1.52E-71	0.51039015	0.518	0.26	4.90E-67	7
Nop581	2.98E-71	0.52365688	0.347	0.143	9.62E-67	7
Ndufb2	6.18E-71	0.49645575	0.454	0.216	1.99E-66	7
Pcif1	6.35E-71	0.53973031	0.275	0.105	2.05E-66	7
Ndufa14	1.78E-70	0.55079667	0.613	0.348	5.74E-66	7
Inpp5d	2.32E-70	0.43103132	0.255	0.091	7.48E-66	7
Tle4	4.81E-70	0.51299254	0.31	0.125	1.55E-65	7
Rplp01	8.01E-70	0.48313088	0.959	0.731	2.59E-65	7
Mrps18c	8.06E-70	0.56874088	0.325	0.138	2.60E-65	7
Rpl182	1.18E-69	0.45724597	0.95	0.705	3.80E-65	7
Swi51	1.46E-69	0.4859169	0.484	0.238	4.72E-65	7
Coa3	1.57E-69	0.54509706	0.295	0.119	5.08E-65	7
Rpl223	2.21E-69	0.46730644	0.947	0.656	7.13E-65	7
Tmem256	3.41E-69	0.48830188	0.524	0.265	1.10E-64	7
Ndufa3	4.03E-69	0.57734515	0.672	0.399	1.30E-64	7
Srp19	4.37E-69	0.47049318	0.362	0.157	1.41E-64	7

Ndufv2	1.04E-68	0.48188932	0.363	0.16	3.37E-64	7
Ssbp1	1.69E-68	0.46848794	0.34	0.144	5.45E-64	7
Rpp21	2.32E-68	0.52901947	0.328	0.139	7.50E-64	7
Rpl37a2	1.09E-67	0.38312865	0.998	0.901	3.52E-63	7
Ufm1	1.34E-67	0.47801146	0.307	0.125	4.34E-63	7
H2-Q7	2.23E-67	0.46697735	0.252	0.09	7.21E-63	7
Hnrnph11	7.06E-67	0.40129116	0.456	0.216	2.28E-62	7
Sec11a	8.29E-67	0.46810766	0.31	0.127	2.68E-62	7
Atp5f11	1.25E-66	0.48737576	0.572	0.311	4.02E-62	7
Fundc2	1.77E-66	0.50291841	0.314	0.13	5.73E-62	7
Cwc15	2.37E-66	0.48499856	0.385	0.176	7.64E-62	7
Mrps12	5.21E-66	0.48174862	0.25	0.093	1.68E-61	7
Mrpl30	6.60E-66	0.43979659	0.378	0.171	2.13E-61	7
Naa38	7.18E-66	0.49039974	0.352	0.154	2.32E-61	7
Prmt1	1.17E-65	0.39159377	0.306	0.122	3.78E-61	7
Prrc2c	1.01E-64	0.4575356	0.472	0.234	3.27E-60	7
Dpy30	1.20E-64	0.52117236	0.308	0.129	3.89E-60	7
mt-Atp81	2.00E-64	0.38150188	0.523	0.268	6.47E-60	7
Ddx24	2.81E-64	0.41053836	0.317	0.131	9.08E-60	7
Rps4x2	2.84E-64	0.43043351	0.942	0.685	9.16E-60	7
Sptssa	3.57E-64	0.48802744	0.272	0.107	1.15E-59	7
Matr3	4.60E-64	0.40704825	0.287	0.114	1.48E-59	7
Ndufs3	6.97E-64	0.3935559	0.27	0.105	2.25E-59	7
Rp9	8.19E-64	0.49949854	0.355	0.16	2.64E-59	7
Ybx12	1.35E-63	0.3108621	0.764	0.436	4.36E-59	7
Xist1	1.37E-63	0.84342438	0.299	0.126	4.41E-59	7
1810037I17R	5.25E-63	0.53742229	0.61	0.34	1.70E-58	7
Hdgf1	1.00E-62	0.31163751	0.483	0.234	3.23E-58	7
Mien1	1.27E-62	0.45042759	0.294	0.121	4.11E-58	7
Ost4	1.49E-62	0.46515579	0.598	0.34	4.80E-58	7
mt-Co12	2.67E-62	0.30319454	0.975	0.88	8.61E-58	7
Sec61b	3.58E-62	0.41958213	0.733	0.437	1.16E-57	7
Tsn	3.78E-62	0.43001556	0.297	0.122	1.22E-57	7
Polr2f	5.02E-62	0.44253698	0.353	0.157	1.62E-57	7
Ifi203	5.88E-62	0.33966407	0.301	0.121	1.90E-57	7
Dnajc19	6.65E-62	0.45297038	0.308	0.132	2.15E-57	7
Csnk2b	9.35E-62	0.52117699	0.396	0.193	3.02E-57	7
Snrnp70	1.07E-61	0.42903166	0.38	0.175	3.45E-57	7
Mndal	2.04E-61	0.37919553	0.284	0.112	6.60E-57	7
Sf3b12	2.24E-61	0.41860411	0.494	0.259	7.24E-57	7
Elf1	2.40E-61	0.36856851	0.315	0.132	7.75E-57	7
Wbp11	2.90E-61	0.4277836	0.296	0.123	9.36E-57	7
Psma1	3.55E-61	0.46127465	0.344	0.155	1.15E-56	7

Psmd13	4.51E-61	0.48576395	0.253	0.099	1.46E-56	7
Ube2m	5.15E-61	0.4718995	0.355	0.163	1.66E-56	7
Tmem14c	5.80E-61	0.30164203	0.458	0.226	1.87E-56	7
Snhg3	6.66E-61	0.58276951	0.301	0.128	2.15E-56	7
Celf2	7.42E-61	0.41562806	0.378	0.176	2.40E-56	7
Bcas2	9.74E-61	0.43031963	0.318	0.137	3.14E-56	7
Fli1	1.91E-60	0.49880468	0.274	0.113	6.17E-56	7
Rpl10a2	4.14E-60	0.43027343	0.915	0.594	1.34E-55	7
Cisd2	4.79E-60	0.52372418	0.307	0.134	1.55E-55	7
Tcea1	7.56E-60	0.4084722	0.368	0.17	2.44E-55	7
Smim11	9.95E-60	0.47419916	0.308	0.133	3.21E-55	7
Zmat2	2.13E-59	0.39132033	0.303	0.128	6.86E-55	7
Tacc1	2.27E-59	0.48490622	0.331	0.149	7.33E-55	7
Zc3h151	3.07E-59	0.45616243	0.418	0.204	9.91E-55	7
Rpl72	3.09E-59	0.39868593	0.902	0.623	9.98E-55	7
Ppp2r5a2	4.00E-59	0.49818094	0.397	0.196	1.29E-54	7
Atp5a12	4.49E-59	0.37228686	0.538	0.283	1.45E-54	7
Ndufs7	4.87E-59	0.44286225	0.374	0.177	1.57E-54	7
Eloc	6.00E-59	0.47830917	0.459	0.24	1.94E-54	7
Psmd8	7.27E-59	0.4535094	0.389	0.187	2.35E-54	7
Psma5	7.74E-59	0.3261455	0.376	0.173	2.50E-54	7
Ube2l3	1.54E-58	0.45700822	0.405	0.199	4.98E-54	7
Adgre5	1.91E-58	0.41780671	0.319	0.139	6.18E-54	7
Cggbp1	4.71E-58	0.48755398	0.384	0.187	1.52E-53	7
Btf32	8.91E-58	0.40419327	0.828	0.538	2.88E-53	7
Ndufb6	1.50E-57	0.41801893	0.313	0.138	4.85E-53	7
Ube2d2a	4.65E-57	0.39838433	0.341	0.155	1.50E-52	7
Cox7c3	4.70E-57	0.38312137	0.909	0.662	1.52E-52	7
Sub11	6.58E-57	0.35833316	0.759	0.469	2.12E-52	7
Tmem167	2.22E-56	0.4178935	0.438	0.223	7.16E-52	7
Lamtor2	2.83E-56	0.47666838	0.483	0.261	9.14E-52	7
Rbm22	4.12E-56	0.43130295	0.266	0.109	1.33E-51	7
Pcbp11	4.15E-56	0.35849433	0.471	0.241	1.34E-51	7
Elavl1	5.76E-56	0.42702324	0.27	0.112	1.86E-51	7
Tmem2582	9.55E-56	0.40303087	0.644	0.376	3.08E-51	7
Tmem179b	1.49E-55	0.47895826	0.268	0.113	4.82E-51	7
Brd1	1.58E-55	0.43686394	0.253	0.102	5.10E-51	7
Vdac31	2.86E-55	0.32764522	0.345	0.157	9.22E-51	7
Dbi2	2.94E-55	0.33482076	0.567	0.3	9.48E-51	7
Serp11	3.45E-55	0.33641703	0.522	0.283	1.11E-50	7
Rps282	3.78E-55	0.41622999	0.999	0.84	1.22E-50	7
Ndufb4	4.71E-55	0.45655403	0.444	0.232	1.52E-50	7
Dnajc2	5.93E-55	0.36929408	0.262	0.106	1.91E-50	7

Atp5d1	1.44E-54	0.41606095	0.49	0.265	4.66E-50	7
Eif3i	1.82E-54	0.3618385	0.356	0.165	5.88E-50	7
Ppp4c	2.09E-54	0.43428923	0.277	0.119	6.76E-50	7
Pfdn51	4.01E-54	0.3603449	0.724	0.449	1.29E-49	7
Klhl241	5.77E-54	0.30580095	0.253	0.101	1.86E-49	7
Zeb2	6.58E-54	0.25102471	0.313	0.135	2.12E-49	7
Rpl152	9.84E-54	0.38303239	0.936	0.651	3.18E-49	7
Pin4	1.20E-53	0.37967628	0.307	0.138	3.86E-49	7
Fam111a	1.69E-53	0.46458474	0.327	0.152	5.47E-49	7
Gmfg4	3.99E-53	0.37085518	0.675	0.394	1.29E-48	7
Snrnp271	4.23E-53	0.40337849	0.381	0.187	1.37E-48	7
Dhx15	3.50E-52	0.35196514	0.325	0.149	1.13E-47	7
Tomm201	3.57E-52	0.32749221	0.483	0.25	1.15E-47	7
Ssb1	4.10E-52	0.33247439	0.424	0.213	1.32E-47	7
Lamtor5	5.54E-52	0.4348981	0.286	0.127	1.79E-47	7
Rnaseh2c1	8.38E-52	0.45689671	0.422	0.224	2.70E-47	7
Cnot3	9.67E-52	0.4320721	0.272	0.117	3.12E-47	7
Paics	2.50E-51	0.33020372	0.275	0.117	8.08E-47	7
Polr1d2	2.96E-51	0.36583266	0.524	0.292	9.55E-47	7
Cmpk1	3.26E-51	0.45396712	0.322	0.154	1.05E-46	7
Gadd45gip1	3.81E-51	0.29672196	0.254	0.104	1.23E-46	7
Rpl352	4.14E-51	0.38225476	0.992	0.766	1.34E-46	7
Emp31	4.71E-51	0.49624827	0.545	0.315	1.52E-46	7
Bzw11	7.65E-51	0.36902334	0.416	0.213	2.47E-46	7
Pcbp22	9.68E-51	0.33901241	0.568	0.324	3.13E-46	7
Marcksl1	1.08E-50	0.38311136	0.252	0.105	3.48E-46	7
Limd21	1.16E-50	0.37892559	0.42	0.221	3.74E-46	7
Luc7l3	1.77E-50	0.32325633	0.264	0.111	5.73E-46	7
Psenen	2.75E-50	0.40765638	0.456	0.248	8.89E-46	7
Papola	5.01E-50	0.30074294	0.254	0.106	1.62E-45	7
Mrpl23	6.19E-50	0.32089248	0.311	0.141	2.00E-45	7
Mier1	7.37E-50	0.35857814	0.346	0.166	2.38E-45	7
Prpf40a	1.09E-49	0.3372064	0.321	0.148	3.51E-45	7
Cnot1	1.54E-49	0.29585709	0.251	0.104	4.98E-45	7
Ubxn11	1.57E-49	0.35516326	0.435	0.228	5.06E-45	7
Sfpq1	5.66E-49	0.38520212	0.427	0.224	1.83E-44	7
Acin1	9.25E-49	0.36822425	0.331	0.157	2.99E-44	7
Gstp1	1.60E-48	0.45741236	0.301	0.141	5.15E-44	7
Higd1a	1.90E-48	0.28462543	0.365	0.179	6.15E-44	7
Rps102	2.42E-48	0.33606116	0.982	0.797	7.82E-44	7
Rheb	4.95E-48	0.39103206	0.28	0.127	1.60E-43	7
Eif3e1	5.35E-48	0.25030538	0.431	0.218	1.73E-43	7
Rac25	6.64E-48	0.31823159	0.679	0.399	2.14E-43	7

Psmb7	6.68E-48	0.35645963	0.277	0.124	2.16E-43	7
Rpl102	7.82E-48	0.34950942	0.861	0.594	2.52E-43	7
D8Ertd738e2	9.51E-48	0.31618533	0.666	0.415	3.07E-43	7
Rplp22	1.43E-47	0.34865094	0.954	0.707	4.63E-43	7
Eif1ax1	1.65E-47	0.3158069	0.379	0.19	5.33E-43	7
Brd7	2.98E-47	0.41649046	0.28	0.128	9.62E-43	7
Luc7l2	4.13E-47	0.30868022	0.404	0.21	1.33E-42	7
Pabpn1	4.64E-47	0.2599144	0.293	0.132	1.50E-42	7
Rpl302	6.51E-47	0.29019297	0.985	0.811	2.10E-42	7
2310009A05I	9.88E-47	0.4429709	0.257	0.116	3.19E-42	7
Rbis	1.25E-46	0.3054366	0.34	0.164	4.04E-42	7
Rps22	1.31E-46	0.41175719	0.899	0.621	4.23E-42	7
Cuta	2.16E-46	0.42306275	0.336	0.167	6.97E-42	7
Son1	3.17E-46	0.34378759	0.617	0.377	1.02E-41	7
Ndufa8	3.65E-46	0.43911312	0.339	0.171	1.18E-41	7
Dynlrb11	1.55E-45	0.3446517	0.402	0.213	5.01E-41	7
Tomm5	1.57E-45	0.38740776	0.29	0.134	5.07E-41	7
Phb2	1.74E-45	0.33387618	0.269	0.121	5.63E-41	7
Ptges31	3.08E-45	0.37334058	0.422	0.225	9.95E-41	7
Mrps16	3.67E-45	0.29834562	0.321	0.155	1.19E-40	7
Cox16	4.73E-45	0.40985977	0.253	0.113	1.53E-40	7
Pycard	4.90E-45	0.36824036	0.289	0.137	1.58E-40	7
Cox171	6.42E-45	0.3467874	0.689	0.444	2.07E-40	7
1810026B05I	1.48E-44	0.41040184	0.25	0.112	4.79E-40	7
Nudcd3	1.59E-44	0.25763514	0.258	0.113	5.15E-40	7
Morf4l11	2.95E-44	0.29174378	0.602	0.36	9.52E-40	7
Aimp11	1.31E-43	0.27723365	0.389	0.201	4.22E-39	7
Ptbp31	2.14E-43	0.35652021	0.47	0.268	6.91E-39	7
Iscu	3.49E-43	0.36156753	0.274	0.128	1.13E-38	7
Unc93b11	4.36E-43	0.26202338	0.296	0.14	1.41E-38	7
Hnrnpc1	4.37E-43	0.28336281	0.411	0.218	1.41E-38	7
Phip	9.90E-43	0.27738714	0.278	0.127	3.19E-38	7
Timm23	9.96E-43	0.31417006	0.277	0.129	3.21E-38	7
Psma32	2.40E-42	0.29316756	0.449	0.248	7.75E-38	7
Khdrbs1	2.78E-42	0.26709062	0.296	0.141	8.98E-38	7
Uqcrc1	3.70E-42	0.32324444	0.276	0.13	1.20E-37	7
Snrpb2	5.80E-42	0.27454064	0.285	0.133	1.87E-37	7
Fam107b1	6.66E-42	0.36429344	0.382	0.207	2.15E-37	7
Ndufb91	7.39E-42	0.36010565	0.571	0.354	2.39E-37	7
Bloc1s1	9.66E-42	0.48148526	0.359	0.194	3.12E-37	7
Rbm392	1.13E-41	0.3408954	0.628	0.399	3.65E-37	7
Wdr83os	2.86E-41	0.3338187	0.327	0.165	9.22E-37	7
Chic2	4.13E-41	0.36837041	0.274	0.132	1.33E-36	7

Sep-71	5.16E-41	0.28309208	0.399	0.216	1.67E-36	7
Dusp111	8.03E-41	0.3052102	0.354	0.185	2.59E-36	7
Rpl310	9.31E-41	0.2709099	0.85	0.548	3.01E-36	7
Mapre1	1.25E-40	0.38034863	0.358	0.191	4.02E-36	7
Scand11	1.51E-40	0.30090243	0.569	0.348	4.87E-36	7
Pomp3	1.61E-40	0.33030849	0.558	0.338	5.19E-36	7
Calm13	1.80E-40	0.27342273	0.893	0.637	5.82E-36	7
Bri32	2.13E-40	0.33146082	0.452	0.257	6.88E-36	7
Mrfap11	2.35E-40	0.28754765	0.359	0.186	7.60E-36	7
Sys1	3.70E-40	0.28545646	0.271	0.129	1.20E-35	7
Rps27a1	1.12E-39	0.27497371	0.983	0.89	3.61E-35	7
Slirp	3.78E-39	0.26666415	0.268	0.127	1.22E-34	7
Eif4h	4.06E-39	0.27728143	0.266	0.125	1.31E-34	7
Rex1bd	4.07E-39	0.29364296	0.274	0.133	1.31E-34	7
Pcnp	5.94E-39	0.28962471	0.302	0.152	1.92E-34	7
Syf2	6.66E-39	0.30363623	0.364	0.196	2.15E-34	7
Dctn3	3.28E-38	0.38016102	0.321	0.171	1.06E-33	7
Cops9	3.42E-38	0.32721439	0.456	0.268	1.11E-33	7
Rpl362	5.06E-38	0.28502875	0.991	0.753	1.63E-33	7
Sf3b21	9.55E-38	0.30033265	0.374	0.203	3.08E-33	7
Rpl36a2	2.75E-37	0.276332	0.864	0.573	8.88E-33	7
Klf21	9.77E-37	0.33346128	0.392	0.22	3.15E-32	7
Gng51	1.15E-36	0.35586297	0.874	0.645	3.71E-32	7
Nhp2	1.21E-36	0.28843994	0.274	0.134	3.89E-32	7
Cox7b2	1.35E-36	0.29068369	0.549	0.337	4.36E-32	7
Dctn4	2.13E-36	0.28908194	0.307	0.159	6.86E-32	7
Skp1a1	3.81E-36	0.25974176	0.392	0.217	1.23E-31	7
Fubp1	3.86E-36	0.25893484	0.252	0.119	1.24E-31	7
Eif4e2	6.37E-36	0.30430053	0.257	0.126	2.06E-31	7
Rhoa	1.22E-35	0.32283136	0.67	0.444	3.95E-31	7
Cd471	3.18E-35	0.25735181	0.608	0.394	1.03E-30	7
Cfl12	3.94E-35	0.29475228	0.83	0.611	1.27E-30	7
Aurkaip1	4.78E-35	0.3443881	0.312	0.169	1.54E-30	7
Birc6	9.78E-35	0.31124597	0.3	0.156	3.16E-30	7
Rer1	1.04E-34	0.34538558	0.274	0.141	3.35E-30	7
Reep5	1.37E-34	0.30038756	0.448	0.264	4.44E-30	7
Rpl233	7.66E-34	0.26744655	0.987	0.819	2.47E-29	7
Cyc1	9.95E-33	0.25360231	0.288	0.15	3.21E-28	7
Rsrc2	1.98E-32	0.26403928	0.35	0.196	6.41E-28	7
Cdkn1b	4.52E-32	0.29552486	0.293	0.155	1.46E-27	7
Snx3	2.57E-31	0.25574648	0.324	0.18	8.29E-27	7
Wasf2	5.96E-31	0.27086826	0.281	0.149	1.92E-26	7
Trappc2l	8.20E-31	0.30039278	0.26	0.137	2.65E-26	7

Capzb1	2.05E-30	0.26464645	0.468	0.292	6.62E-26	7
Cript	4.69E-30	0.32530282	0.276	0.149	1.51E-25	7
Ppp1cb	6.43E-30	0.28599465	0.321	0.182	2.08E-25	7
Rasgrp2	1.20E-29	0.27517951	0.271	0.145	3.88E-25	7
Fis11	5.50E-29	0.27671078	0.555	0.367	1.78E-24	7
Ube2k	5.89E-29	0.27173182	0.254	0.134	1.90E-24	7
Ndufv31	1.34E-28	0.25993115	0.407	0.248	4.33E-24	7
Eif4g21	2.83E-28	0.25950096	0.579	0.386	9.14E-24	7
Ndufs4	5.17E-28	0.29405826	0.31	0.179	1.67E-23	7
Cd741	0	4.16081215	0.976	0.139	0	8
H2-Eb11	0	3.74647782	0.707	0.058	0	8
Iglc2	0	3.39089837	0.55	0.025	0	8
H2-Aa1	0	3.37027754	0.7	0.068	0	8
Ms4a1	0	3.160662	0.487	0.009	0	8
H2-Ab11	0	3.15812037	0.674	0.089	0	8
Ly6d1	0	2.93680177	0.823	0.113	0	8
Cd79a2	0	2.8704428	0.92	0.17	0	8
Iglc31	0	2.72362355	0.527	0.036	0	8
Mef2c2	0	2.66965616	0.599	0.137	0	8
Iglc1	0	2.66309793	0.366	0.024	0	8
Ccr7	0	2.62382993	0.4	0.021	0	8
Igkc1	0	2.40178581	0.949	0.164	0	8
Fcmr	0	2.34834401	0.322	0.007	0	8
Gm31243	0	2.30578647	0.285	0.008	0	8
Ighm2	0	2.11726042	0.78	0.208	0	8
Cd79b2	0	2.11240402	0.642	0.149	0	8
Ighd	0	2.05977893	0.253	0.013	0	8
Gm8369	0	1.99394285	0.302	0.03	0	8
Cd83	0	1.99025876	0.257	0.016	0	8
H2-Ob	0	1.94991274	0.262	0.022	0	8
S1pr1	0	1.94405157	0.286	0.034	0	8
Rps272	0	1.8460787	1	0.909	0	8
Btg12	0	1.51516273	0.95	0.609	0	8
Rps243	0	1.40330096	0.995	0.85	0	8
Uba522	0	1.37459904	0.975	0.75	0	8
Ebf12	0	1.36715672	0.807	0.195	0	8
Rps192	0	1.35390248	0.977	0.723	0	8
Rps15a2	0	1.34065376	0.993	0.778	0	8
Rps203	0	1.31411092	0.992	0.735	0	8
Fau2	0	1.30673032	0.999	0.94	0	8
Rpl37a3	0	1.24914577	0.996	0.901	0	8
Rps293	0	1.17156658	1	0.953	0	8
Rpl303	1.07E-293	1.23975496	0.986	0.812	3.46E-289	8

Rpl35a3	2.69E-282	1.08595061	0.994	0.851	8.70E-278	8
Rpl92	3.65E-271	1.22971668	0.96	0.767	1.18E-266	8
Rps132	1.87E-270	1.20677088	0.961	0.79	6.05E-266	8
Rpl18a3	4.82E-261	1.17410173	0.981	0.801	1.56E-256	8
Jund2	1.47E-257	1.45798306	0.858	0.5	4.76E-253	8
Rpl393	1.19E-256	1.05685196	0.998	0.841	3.83E-252	8
Rps73	6.22E-253	1.24398082	0.946	0.723	2.01E-248	8
Rpl343	5.10E-236	1.0652129	0.977	0.821	1.65E-231	8
Rpl192	3.89E-234	1.11394879	0.965	0.754	1.26E-229	8
Tsc22d31	3.48E-227	2.05286803	0.494	0.165	1.12E-222	8
Rpl363	1.56E-225	1.0574058	0.979	0.754	5.05E-221	8
Rpl383	2.97E-218	0.99169808	0.986	0.838	9.60E-214	8
Cd525	3.58E-207	1.31410637	0.887	0.501	1.16E-202	8
Rps283	4.78E-205	0.90158402	0.998	0.84	1.54E-200	8
Rpl27a2	5.88E-205	0.96885526	0.973	0.794	1.90E-200	8
Rpl112	4.72E-186	0.98368009	0.945	0.733	1.53E-181	8
Rps4x3	1.05E-185	1.05130624	0.926	0.686	3.38E-181	8
Rps183	2.50E-184	1.07264574	0.914	0.641	8.07E-180	8
Rps113	7.01E-181	1.04708678	0.933	0.723	2.26E-176	8
Rpl213	1.33E-179	1.03771703	0.924	0.713	4.30E-175	8
Rps27a2	1.62E-176	0.80076306	0.981	0.89	5.23E-172	8
Cd372	5.64E-174	1.86330569	0.471	0.181	1.82E-169	8
Rpl131	1.22E-173	0.91380951	0.976	0.786	3.93E-169	8
Rps53	1.28E-173	1.04512269	0.921	0.675	4.13E-169	8
Rpl234	1.31E-168	0.88525522	0.971	0.82	4.23E-164	8
Rps103	1.34E-167	0.87593571	0.962	0.799	4.33E-163	8
Ltb	7.56E-165	1.71812819	0.25	0.053	2.44E-160	8
Rpl273	1.75E-164	0.9784647	0.921	0.718	5.64E-160	8
mt-Nd4l2	2.49E-163	1.32338405	0.721	0.439	8.04E-159	8
Rpl183	3.09E-157	0.96234406	0.911	0.708	9.98E-153	8
Rps33	3.08E-154	0.98453136	0.9	0.701	9.95E-150	8
Rps164	1.55E-153	0.90983215	0.942	0.773	5.02E-149	8
Rpl353	2.20E-149	0.79901189	0.988	0.767	7.12E-145	8
Rpl323	1.36E-148	0.86520188	0.949	0.724	4.39E-144	8
Stk17b2	4.23E-145	1.65783751	0.581	0.31	1.37E-140	8
Rps3a13	5.61E-138	0.79208384	0.965	0.768	1.81E-133	8
Tmsb102	9.77E-131	0.53519851	0.989	0.738	3.15E-126	8
Rpl372	3.57E-129	0.65368561	0.994	0.913	1.15E-124	8
Ptprcap2	2.10E-128	1.32983425	0.434	0.166	6.77E-124	8
Rps93	1.06E-127	0.71932476	0.949	0.873	3.43E-123	8
Rps263	1.68E-126	0.75181268	0.973	0.781	5.42E-122	8
Rpl292	4.62E-124	1.02584266	0.788	0.568	1.49E-119	8
Klf22	1.04E-122	1.7071867	0.464	0.217	3.35E-118	8

Rpl224	1.12E-119	0.85926081	0.896	0.659	3.62E-115	8
Gm100763	1.48E-119	0.73712354	0.955	0.805	4.77E-115	8
mt-Co13	8.86E-118	0.65782229	0.944	0.881	2.86E-113	8
Cxcr42	1.05E-117	1.48918878	0.463	0.212	3.41E-113	8
Napsa	3.65E-113	1.68995306	0.351	0.138	1.18E-108	8
Pdcd41	5.48E-113	1.45209132	0.48	0.234	1.77E-108	8
Shisa5	1.20E-110	1.59836495	0.307	0.106	3.87E-106	8
Ets11	1.04E-107	1.57216397	0.361	0.146	3.35E-103	8
Ifi301	6.07E-106	1.61111832	0.34	0.126	1.96E-101	8
Rel	1.07E-105	1.60291395	0.287	0.097	3.47E-101	8
Ralgps21	4.15E-105	1.50317642	0.258	0.079	1.34E-100	8
Cd69	8.02E-103	1.3914131	0.259	0.08	2.59E-98	8
Cd722	8.43E-102	1.4444132	0.272	0.086	2.72E-97	8
Rpsa2	2.79E-101	0.74037445	0.915	0.695	9.01E-97	8
H3f3a2	2.61E-99	0.73152991	0.887	0.756	8.41E-95	8
Rps210	8.57E-96	0.7724297	0.851	0.623	2.77E-91	8
Sub12	2.07E-93	1.22340906	0.637	0.475	6.68E-89	8
Malat12	3.59E-90	0.75786861	0.954	0.958	1.16E-85	8
Rpl10a3	1.59E-89	0.77686117	0.822	0.599	5.14E-85	8
Fcrla2	5.85E-89	1.13305118	0.255	0.082	1.89E-84	8
Mzb12	2.66E-88	1.0821	0.274	0.092	8.59E-84	8
Zfp36l1	1.86E-87	1.32332688	0.314	0.122	6.00E-83	8
Foxp12	1.38E-84	1.28422517	0.426	0.215	4.44E-80	8
mt-Cytb3	1.07E-82	0.70543094	0.818	0.66	3.47E-78	8
mt-Atp63	1.58E-82	0.57194452	0.896	0.741	5.10E-78	8
Vpreb32	4.31E-78	0.4336303	0.45	0.184	1.39E-73	8
mt-Co23	1.52E-76	0.55692036	0.897	0.781	4.92E-72	8
Rpl81	8.14E-76	0.58321802	0.921	0.727	2.63E-71	8
Ly6e3	1.71E-74	1.00987566	0.597	0.4	5.51E-70	8
Rpl414	7.80E-74	0.48764506	0.983	0.899	2.52E-69	8
Rpl36a3	2.87E-64	0.65873386	0.787	0.578	9.26E-60	8
Ptma2	9.21E-63	0.33360757	0.946	0.681	2.97E-58	8
Rpl103	2.58E-62	0.72273549	0.748	0.6	8.33E-58	8
Rps232	2.23E-59	0.48694644	0.931	0.757	7.21E-55	8
Rpl22l13	7.04E-59	0.85435095	0.664	0.518	2.27E-54	8
Rpl73	9.92E-56	0.66091388	0.748	0.631	3.20E-51	8
Rpl153	1.77E-55	0.53235257	0.846	0.656	5.72E-51	8
Rpl312	5.02E-55	0.71685959	0.708	0.555	1.62E-50	8
Rps212	6.08E-51	0.28417854	0.955	0.788	1.96E-46	8
Junb2	1.44E-50	0.6402961	0.595	0.429	4.65E-46	8
mt-Co33	1.18E-49	0.34244079	0.887	0.795	3.81E-45	8
H3f3b3	2.04E-49	0.38093133	0.896	0.824	6.57E-45	8
Ifi27l2a3	2.49E-48	0.8764744	0.367	0.195	8.05E-44	8

Dad11	3.92E-48	1.23880083	0.378	0.24	1.27E-43	8
Rpl262	3.33E-46	0.46752212	0.856	0.699	1.08E-41	8
Pkig2	1.99E-44	1.06500651	0.272	0.136	6.41E-40	8
2410006H16	4.25E-44	0.85930142	0.531	0.396	1.37E-39	8
Cytip2	1.92E-43	1.06059789	0.316	0.177	6.21E-39	8
Selenow2	6.85E-43	0.96635125	0.405	0.261	2.21E-38	8
Pfdn52	8.37E-43	0.89818369	0.544	0.459	2.70E-38	8
Snrpg2	2.20E-42	0.62211725	0.658	0.503	7.11E-38	8
Rpl242	2.21E-42	0.44245247	0.876	0.763	7.15E-38	8
Rpl62	8.02E-42	0.34265921	0.923	0.752	2.59E-37	8
Sh3bgrl35	2.91E-40	0.66426575	0.67	0.569	9.39E-36	8
Nsa22	1.00E-38	0.79859679	0.578	0.481	3.23E-34	8
Ctss1	2.65E-38	0.44309367	0.26	0.125	8.56E-34	8
mt-Atp82	3.54E-38	1.03710612	0.397	0.274	1.14E-33	8
Ptpn18	4.68E-37	0.97394293	0.41	0.291	1.51E-32	8
Naca2	1.67E-36	0.57339629	0.679	0.571	5.40E-32	8
Ubb2	4.20E-35	0.43418563	0.805	0.76	1.36E-30	8
Pold41	3.30E-33	1.13952483	0.252	0.142	1.07E-28	8
Coro1a4	3.88E-33	0.73405797	0.539	0.435	1.25E-28	8
Rpl122	4.28E-33	0.33491645	0.726	0.533	1.38E-28	8
H2-K12	3.21E-32	0.70140026	0.552	0.457	1.04E-27	8
Tmsb4x5	6.98E-32	0.25503267	0.997	0.901	2.25E-27	8
Eif12	5.57E-30	0.39680367	0.852	0.839	1.80E-25	8
Unc93b12	6.49E-30	1.0067113	0.25	0.143	2.10E-25	8
Rplp23	1.90E-29	0.38099095	0.846	0.712	6.15E-25	8
Rpl36al3	3.73E-28	0.53441255	0.625	0.51	1.20E-23	8
Syk3	4.77E-26	0.89946331	0.285	0.183	1.54E-21	8
Ddx52	1.88E-22	0.69169091	0.565	0.55	6.06E-18	8
H2-D13	3.36E-22	0.42852565	0.677	0.662	1.09E-17	8
mt-Nd43	5.41E-21	0.4858986	0.555	0.49	1.75E-16	8
Rpl283	4.39E-20	0.2500936	0.884	0.757	1.42E-15	8
Cyba5	7.00E-19	0.47015312	0.618	0.529	2.26E-14	8
Rpl142	2.17E-18	0.37721476	0.634	0.525	7.01E-14	8
mt-Nd22	5.26E-18	0.49881724	0.464	0.381	1.70E-13	8
Eif3f2	1.16E-17	0.76032126	0.361	0.289	3.75E-13	8
Zfp7062	2.40E-17	0.66622557	0.361	0.284	7.75E-13	8
Lyn3	9.06E-17	0.98543644	0.288	0.216	2.93E-12	8
Atp5e3	8.08E-16	0.37885471	0.698	0.7	2.61E-11	8
Serp12	5.60E-15	0.84042316	0.347	0.292	1.81E-10	8
Lsp12	8.68E-15	0.70254968	0.363	0.301	2.80E-10	8
mt-Nd52	3.47E-14	0.6813644	0.347	0.284	1.12E-09	8
Serinc32	1.49E-13	0.62263357	0.409	0.361	4.80E-09	8
Notch22	9.70E-12	0.88827298	0.263	0.21	3.13E-07	8

Grcc10	9.03E-11	0.83698734	0.31	0.271	2.91E-06	8
Pnrc12	1.02E-10	0.73443887	0.353	0.318	3.29E-06	8
Limd22	4.99E-10	0.92298287	0.269	0.229	1.61E-05	8
Gas52	4.31E-09	0.40221769	0.463	0.432	0.00013904	8
Nol73	1.65E-08	0.68440736	0.31	0.272	0.0005321	8
Erp292	4.87E-08	0.67489674	0.254	0.212	0.00157092	8
Scand12	6.73E-08	0.66834961	0.365	0.358	0.00217394	8
Arpc44	1.02E-07	0.25135689	0.168	0.273	0.00329356	8
Psmb81	1.63E-07	0.72312627	0.252	0.217	0.00527514	8
Akap132	7.47E-07	0.79793292	0.275	0.248	0.02411361	8
Atp6v1f2	1.59E-06	0.69490729	0.332	0.326	0.05120394	8
Atp5g22	7.86E-06	0.39887937	0.43	0.418	0.25361415	8
Rac26	0.00018183	0.47854458	0.394	0.413	1	8
Hmgn12	0.00018443	0.43828684	0.278	0.25	1	8
Oaz12	0.00023313	0.31327532	0.574	0.646	1	8
Gdi22	0.00031509	0.5968025	0.252	0.236	1	8
Snrpe3	0.00037097	0.27356088	0.439	0.431	1	8
Slc38a22	0.00069129	0.52948953	0.362	0.375	1	8
Ubl52	0.00088043	0.39845582	0.468	0.526	1	8
Rnasek1	0.00102784	0.32901051	0.212	0.296	1	8
Psme12	0.00137071	0.57463839	0.305	0.311	1	8
mt-Nd32	0.00184111	0.3357435	0.318	0.304	1	8
Atp6v0b2	0.0021686	0.28230235	0.209	0.29	1	8
Nop103	0.0029683	0.50712075	0.263	0.255	1	8
Btf33	0.00600314	0.25669173	0.517	0.553	1	8
Cd533	0.00699415	0.5590954	0.262	0.262	1	8
Uqcrh2	0.00707048	0.31520691	0.464	0.519	1	8
Clta3	0.00712682	0.47286234	0.312	0.324	1	8
Plac8	0.00779356	0.26263645	0.292	0.28	1	8
Clk13	0.0087239	0.63755238	0.279	0.288	1	8
Tra2b2	0.00948034	0.61006532	0.259	0.262	1	8
Lyz24	0	3.16100612	0.99	0.377	0	9
Ms4a6c1	0	3.05175886	0.864	0.037	0	9
Ifitm34	0	2.98327037	0.99	0.444	0	9
Ccr2	0	2.77283852	0.787	0.028	0	9
Ifi27l2a4	0	2.75117417	0.792	0.175	0	9
F13a1	0	2.69422808	0.736	0.024	0	9
S100a42	0	2.62307277	0.941	0.196	0	9
Atf3	0	2.60099908	0.745	0.048	0	9
Crip12	0	2.59223258	0.947	0.378	0	9
Ly6c23	0	2.45729204	0.806	0.203	0	9
Ccl2	0	2.40868949	0.472	0.02	0	9
Psap1	0	2.28743629	0.971	0.279	0	9

Ifi302	0	2.26108737	0.82	0.103	0	9
Apoc2	0	2.2250277	0.427	0.013	0	9
Lgals34	0	2.22304396	0.947	0.371	0	9
Plac81	0	2.19091179	0.881	0.252	0	9
Cst31	0	2.1008961	0.987	0.454	0	9
Clec4a3	0	2.0123066	0.487	0.01	0	9
Fcer1g5	0	2.00760161	0.99	0.373	0	9
Wfdc172	0	1.98035238	0.802	0.087	0	9
Ctss2	0	1.97720188	0.869	0.097	0	9
Mpeg11	0	1.94191856	0.704	0.058	0	9
Ms4a4c	0	1.94125126	0.524	0.013	0	9
Lst13	0	1.92576455	0.888	0.239	0	9
Tyrobp4	0	1.8423507	0.988	0.389	0	9
Smpdl3a	0	1.82262682	0.551	0.048	0	9
Cybb4	0	1.78030153	0.847	0.21	0	9
Tmsb103	0	1.76794713	0.987	0.738	0	9
Gm9733	0	1.76340326	0.428	0.038	0	9
Npc21	0	1.74013814	0.852	0.224	0	9
Alox5ap4	0	1.71270188	0.906	0.3	0	9
Klf4	0	1.70041369	0.576	0.075	0	9
Cd143	0	1.67761882	0.709	0.161	0	9
Gngt21	0	1.67290274	0.376	0.056	0	9
Ctsh1	0	1.62971017	0.632	0.096	0	9
Ccl91	0	1.62864048	0.516	0.032	0	9
Hopx	0	1.62661209	0.484	0.033	0	9
Cdkn1a	0	1.61003652	0.549	0.074	0	9
Napsa1	0	1.59018851	0.657	0.124	0	9
Ly861	0	1.57822145	0.582	0.06	0	9
Pld4	0	1.55423473	0.517	0.029	0	9
Ifitm23	0	1.54089961	0.953	0.436	0	9
Ms4a6b	0	1.53284472	0.429	0.022	0	9
Gm2a1	0	1.51357162	0.619	0.085	0	9
Emp32	0	1.48408232	0.807	0.303	0	9
Cyba6	0	1.47324821	0.965	0.512	0	9
Fxyd53	0	1.4695206	0.936	0.347	0	9
Pycard1	0	1.44321563	0.61	0.122	0	9
Atox11	0	1.42377495	0.889	0.373	0	9
Emb2	0	1.41058457	0.653	0.132	0	9
Ptpn181	0	1.40210014	0.823	0.272	0	9
Dbi3	0	1.39248586	0.821	0.289	0	9
Sat12	0	1.38705365	0.812	0.287	0	9
Clec4a1	0	1.38015934	0.306	0.006	0	9
Gpx11	0	1.38008871	0.97	0.499	0	9

Cebpb2	0	1.37330806	0.958	0.368	0	9
Coro1a5	0	1.35157916	0.923	0.417	0	9
Lsp13	0	1.33629443	0.84	0.279	0	9
Nfil3	0	1.32165182	0.392	0.043	0	9
Gpr141	0	1.30994272	0.367	0.039	0	9
Rassf4	0	1.30907845	0.39	0.024	0	9
Lamp12	0	1.30687358	0.756	0.214	0	9
Zfp362	0	1.30215567	0.736	0.208	0	9
Cd68	0	1.29789958	0.428	0.035	0	9
Metrnl	0	1.29339094	0.312	0.021	0	9
Zeb21	0	1.28668334	0.601	0.122	0	9
Mndal1	0	1.28649426	0.558	0.1	0	9
Csf1r	0	1.27247511	0.369	0.028	0	9
Fn12	0	1.26722043	0.787	0.173	0	9
Gm46224	0	1.26586681	0.342	0.021	0	9
Tgfbi2	0	1.23467535	0.559	0.076	0	9
Tmsb4x6	0	1.23286679	0.999	0.901	0	9
Ctsc1	0	1.229799	0.591	0.074	0	9
Msrb13	0	1.22804047	0.856	0.299	0	9
Cd302	0	1.22399414	0.366	0.018	0	9
Gng10	0	1.21122686	0.519	0.114	0	9
Ciao2a1	0	1.20752684	0.543	0.129	0	9
Itgb7	0	1.18679777	0.356	0.021	0	9
Ftl13	0	1.17740972	0.992	0.801	0	9
Ifngr1	0	1.1691802	0.492	0.102	0	9
AI839979	0	1.1577765	0.309	0.019	0	9
Ap1s2	0	1.14901219	0.538	0.123	0	9
Gm36161	0	1.14566969	0.293	0.025	0	9
Gm21188	0	1.13477827	0.313	0.032	0	9
Cx3cr1	0	1.13333454	0.261	0.022	0	9
Emilin21	0	1.11444464	0.451	0.059	0	9
Ccl63	0	1.11149061	0.768	0.158	0	9
Cd300a	0	1.09228989	0.329	0.043	0	9
Idh1	0	1.08076853	0.378	0.05	0	9
Cd48	0	1.07804105	0.394	0.061	0	9
Naaa	0	1.06574923	0.325	0.017	0	9
Il6ra	0	1.04902049	0.329	0.031	0	9
Grn1	0	1.04584708	0.566	0.133	0	9
Ctsz1	0	1.03141048	0.644	0.152	0	9
Tnfsf13	0	1.02346762	0.279	0.024	0	9
Ccl3	0	0.9727776	0.337	0.03	0	9
Ctsa	0	0.97206688	0.402	0.068	0	9
Mcub	0	0.96808666	0.322	0.024	0	9

Lrp1	0	0.96740014	0.364	0.049	0	9
Trps1	0	0.94662311	0.302	0.036	0	9
Sowahc	0	0.93840437	0.25	0.018	0	9
Atp1a3	0	0.91023314	0.29	0.034	0	9
Nr4a2	0	0.90446793	0.257	0.027	0	9
C1galt1c1	0	0.90113604	0.285	0.029	0	9
Irf5	0	0.86632814	0.268	0.016	0	9
Rnase6	0	0.83136597	0.266	0.026	0	9
Ahnak1	9.83E-306	0.9986144	0.85	0.254	3.18E-301	9
Sh3bgrl36	2.84E-304	1.2583192	0.965	0.555	9.18E-300	9
Ggh	3.46E-299	0.90855142	0.318	0.047	1.12E-294	9
Fau3	4.15E-298	0.90325012	0.997	0.94	1.34E-293	9
Ctsb1	1.81E-291	0.95803714	0.732	0.219	5.83E-287	9
Sem14	2.11E-289	1.12082404	0.968	0.657	6.81E-285	9
Klf13	1.19E-282	1.17635781	0.582	0.159	3.85E-278	9
Pim13	4.33E-281	1.19724874	0.852	0.318	1.40E-276	9
Tlr21	2.41E-279	0.97107973	0.36	0.062	7.79E-275	9
Mrpl332	6.22E-278	1.30680468	0.89	0.435	2.01E-273	9
Cd526	6.62E-277	1.16226679	0.977	0.497	2.14E-272	9
H2-DMa	7.54E-277	0.82624217	0.363	0.063	2.43E-272	9
Acer3	4.45E-275	0.77841437	0.256	0.033	1.44E-270	9
Laptm52	2.63E-270	1.11627904	0.747	0.255	8.50E-266	9
Mgst14	3.22E-270	1.20123092	0.678	0.216	1.04E-265	9
Samhd12	5.98E-269	1.15632117	0.591	0.161	1.93E-264	9
Notch23	7.19E-268	1.11144475	0.642	0.192	2.32E-263	9
Nr4a13	4.01E-267	1.19170466	0.629	0.186	1.29E-262	9
Rilpl2	1.23E-266	0.96661798	0.364	0.067	3.96E-262	9
Fos3	1.52E-265	1.18021763	0.85	0.341	4.91E-261	9
Gpr65	1.45E-263	0.85251836	0.31	0.05	4.70E-259	9
Serf2	5.76E-261	0.9990682	0.971	0.718	1.86E-256	9
Slfn23	3.04E-260	1.08385133	0.547	0.147	9.80E-256	9
Erp293	4.05E-257	1.00950816	0.646	0.194	1.31E-252	9
C32	1.25E-256	0.95915212	0.508	0.131	4.02E-252	9
Hp4	6.99E-255	1.08823781	0.812	0.286	2.26E-250	9
Lyn4	1.39E-254	1.07193553	0.646	0.199	4.48E-250	9
Apoe1	2.71E-252	1.07563687	0.445	0.101	8.76E-248	9
Ikbkb	5.71E-250	0.9053763	0.335	0.061	1.84E-245	9
Rnf1301	1.19E-244	1.07413169	0.468	0.12	3.84E-240	9
BC028528	6.19E-244	0.87857129	0.257	0.038	2.00E-239	9
Sec61b1	6.25E-244	1.20652592	0.873	0.431	2.02E-239	9
Lamtor43	2.09E-242	1.20467213	0.708	0.262	6.74E-238	9
Itm2b3	3.31E-241	1.15660603	0.911	0.522	1.07E-236	9
Klf23	7.00E-241	1.13542904	0.651	0.209	2.26E-236	9

H2afj1	7.78E-239	1.19348045	0.857	0.415	2.51E-234	9
Ccdc12	8.75E-237	1.01426095	0.622	0.2	2.83E-232	9
Cytip3	8.86E-237	0.94165363	0.582	0.165	2.86E-232	9
Unc93b13	1.87E-236	0.85815444	0.507	0.131	6.04E-232	9
Tspo	2.50E-236	1.24556089	0.851	0.443	8.07E-232	9
Sirpa	1.74E-235	0.87386276	0.373	0.077	5.63E-231	9
Tpd522	6.06E-235	0.90998232	0.58	0.165	1.96E-230	9
Nrros	7.19E-235	0.7919723	0.304	0.054	2.32E-230	9
Dnajc15	6.21E-234	0.82201429	0.372	0.079	2.00E-229	9
Rgs10	1.52E-233	0.71370154	0.294	0.049	4.90E-229	9
Rpl36al4	1.64E-232	1.10401617	0.927	0.496	5.31E-228	9
Pitpna	4.32E-229	0.9538321	0.542	0.157	1.40E-224	9
Hacd41	2.86E-228	0.76191472	0.311	0.056	9.24E-224	9
Rps294	1.11E-227	0.80013708	0.997	0.953	3.57E-223	9
Fam174a	4.58E-227	0.89135311	0.289	0.051	1.48E-222	9
Rps27a3	5.46E-227	0.83860743	0.995	0.889	1.76E-222	9
Actb4	4.29E-225	1.05586244	1	0.947	1.38E-220	9
Ramp1	4.89E-223	0.89010008	0.426	0.102	1.58E-218	9
Hexa	1.69E-222	0.80367824	0.341	0.069	5.47E-218	9
Myo1g	1.82E-220	0.82229667	0.266	0.044	5.89E-216	9
Fcgr32	2.15E-220	0.84914015	0.439	0.109	6.95E-216	9
Mia2	9.41E-220	0.82101093	0.403	0.093	3.04E-215	9
Ndufb1-ps3	4.92E-218	0.98600155	0.936	0.626	1.59E-213	9
Dusp12	4.62E-215	0.79273393	0.644	0.208	1.49E-210	9
Reep51	4.93E-214	1.04242768	0.677	0.254	1.59E-209	9
Ifi2031	6.31E-212	0.90640044	0.453	0.114	2.04E-207	9
Glud1	9.27E-212	0.81798712	0.488	0.132	2.99E-207	9
Pomp4	5.65E-209	1.03750637	0.769	0.329	1.82E-204	9
Atp6v0b3	8.11E-209	1.01427879	0.694	0.267	2.62E-204	9
Sla	1.88E-207	0.78676587	0.285	0.053	6.07E-203	9
Shisa51	4.38E-206	0.68172159	0.422	0.101	1.41E-201	9
Creg1	1.41E-205	0.78328369	0.497	0.141	4.54E-201	9
Scand13	1.40E-203	1.00210405	0.78	0.338	4.53E-199	9
Arpc1b2	3.06E-202	1.02966876	0.851	0.423	9.88E-198	9
Fam49b1	1.46E-198	0.99000844	0.637	0.234	4.73E-194	9
Taldo13	4.07E-198	0.97424884	0.816	0.376	1.31E-193	9
Arl6ip5	4.17E-198	0.81663641	0.38	0.091	1.35E-193	9
Prdx54	6.11E-198	0.99564006	0.905	0.432	1.97E-193	9
Psmb82	2.38E-197	0.88400054	0.602	0.2	7.68E-193	9
Capg1	3.65E-195	0.94499354	0.547	0.175	1.18E-190	9
Tmem14c1	1.10E-194	0.75693196	0.628	0.219	3.54E-190	9
Tm6sf1	1.31E-194	0.88904876	0.3	0.063	4.24E-190	9
Itga4	5.85E-193	0.80632299	0.372	0.089	1.89E-188	9

Clec4a23	7.87E-193	1.02450343	0.507	0.158	2.54E-188	9
Prkcd	4.98E-192	0.83650667	0.447	0.123	1.61E-187	9
Gltp	6.08E-191	0.74838983	0.324	0.071	1.96E-186	9
Chil32	1.50E-189	1.04386714	0.537	0.163	4.86E-185	9
Smdt13	1.04E-188	1.00405074	0.741	0.323	3.36E-184	9
Sub13	4.98E-188	0.95422488	0.875	0.464	1.61E-183	9
Fosb	1.96E-187	0.82453394	0.469	0.133	6.33E-183	9
Ucp23	8.21E-187	0.81364106	0.786	0.332	2.65E-182	9
Arpc23	6.98E-186	0.9600988	0.849	0.432	2.25E-181	9
Card192	4.05E-185	0.83070811	0.468	0.139	1.31E-180	9
Cotl14	4.33E-185	0.84822384	0.584	0.205	1.40E-180	9
Ssr41	2.01E-184	0.80710158	0.655	0.234	6.47E-180	9
Junb3	1.22E-183	0.64316721	0.882	0.416	3.93E-179	9
Capzb2	8.83E-183	0.90626643	0.692	0.282	2.85E-178	9
Ethe11	2.78E-182	0.91205181	0.432	0.128	8.98E-178	9
Ptpn1	3.05E-182	0.85075267	0.4	0.106	9.85E-178	9
Bin2	3.42E-182	0.77517215	0.278	0.056	1.10E-177	9
Clta4	1.09E-179	0.89247444	0.724	0.304	3.53E-175	9
Atp5h1	1.72E-179	0.94843738	0.865	0.461	5.54E-175	9
Ndufb32	1.72E-179	0.88300867	0.625	0.233	5.56E-175	9
Uba523	3.27E-179	0.77852398	0.986	0.749	1.06E-174	9
Sap30	5.39E-178	0.77422399	0.319	0.073	1.74E-173	9
Rps114	1.30E-177	0.83534372	0.989	0.72	4.20E-173	9
Psma71	3.65E-177	0.87538528	0.809	0.364	1.18E-172	9
Fabp51	8.71E-177	0.83749622	0.382	0.1	2.81E-172	9
Add3	3.06E-176	0.77542276	0.301	0.066	9.88E-172	9
Ninj1	4.12E-176	0.79332086	0.359	0.088	1.33E-171	9
Prdx13	1.47E-175	0.89096416	0.833	0.377	4.74E-171	9
Atp5l2	7.42E-175	0.89316052	0.924	0.597	2.40E-170	9
Tmem160	1.51E-174	0.75386975	0.444	0.131	4.86E-170	9
H2afz2	5.75E-173	1.01097161	0.922	0.561	1.86E-168	9
Rnh1	5.52E-172	0.78833374	0.366	0.095	1.78E-167	9
Plbd13	1.01E-171	0.97212897	0.476	0.152	3.26E-167	9
Atp6v0c2	5.08E-171	0.91095813	0.795	0.381	1.64E-166	9
Soat1	2.79E-170	0.70709591	0.371	0.096	9.00E-166	9
Gstm1	2.31E-169	0.63976423	0.26	0.051	7.47E-165	9
Man2b1	4.94E-169	0.81322951	0.357	0.092	1.60E-164	9
Gstp11	9.74E-168	0.751234	0.449	0.134	3.14E-163	9
Spi14	9.71E-167	0.82175601	0.569	0.205	3.13E-162	9
Nfe2l2	3.55E-166	0.85329125	0.476	0.151	1.15E-161	9
Cox5b2	9.83E-165	0.92468206	0.843	0.459	3.18E-160	9
Cstb1	4.16E-164	0.71465136	0.581	0.204	1.34E-159	9
Ifitm63	3.17E-163	0.32995671	0.7	0.234	1.02E-158	9

Ptpn6	3.19E-162	0.77446861	0.413	0.123	1.03E-157	9
Cyth4	1.24E-160	0.6684726	0.282	0.062	3.99E-156	9
Stx7	1.24E-160	0.65737267	0.32	0.078	3.99E-156	9
Gng2	5.11E-160	0.64461408	0.368	0.097	1.65E-155	9
Sec61g1	8.64E-159	0.81526061	0.942	0.599	2.79E-154	9
H3f3a3	2.04E-158	0.63321299	0.971	0.753	6.57E-154	9
Atp6v1b2	2.13E-158	0.72912573	0.313	0.077	6.86E-154	9
Esd1	4.02E-157	0.75678938	0.586	0.217	1.30E-152	9
St8sia4	5.86E-157	0.53224117	0.293	0.066	1.89E-152	9
Xbp1	9.38E-157	0.7285058	0.38	0.107	3.03E-152	9
Adgre51	1.22E-156	0.79074621	0.439	0.134	3.94E-152	9
Glipr1	2.27E-156	0.70332927	0.349	0.092	7.32E-152	9
Rpl104	3.22E-156	0.81501765	0.946	0.591	1.04E-151	9
Brk1	1.65E-155	0.79554232	0.516	0.183	5.34E-151	9
Dpm31	4.65E-155	0.74034681	0.624	0.24	1.50E-150	9
Dock10	2.00E-153	0.54113338	0.253	0.053	6.46E-149	9
Timm10b2	2.13E-153	0.80094721	0.615	0.247	6.88E-149	9
Rap1b3	4.11E-153	0.90775603	0.607	0.241	1.33E-148	9
Ly6e4	5.46E-153	0.70488265	0.86	0.388	1.76E-148	9
Prdx41	6.40E-153	0.55244955	0.253	0.053	2.07E-148	9
Bri33	1.45E-152	0.80763699	0.624	0.249	4.69E-148	9
Arhgdib3	3.82E-152	0.80859875	0.872	0.419	1.23E-147	9
Nt5c	4.10E-152	0.69847662	0.365	0.102	1.32E-147	9
Gdpd32	9.42E-152	0.79397386	0.29	0.07	3.04E-147	9
Selplg2	9.81E-152	0.65230513	0.474	0.152	3.17E-147	9
Selenop	1.31E-151	0.48163626	0.263	0.055	4.22E-147	9
Ccl4	4.36E-151	0.29896837	0.256	0.054	1.41E-146	9
Capza22	1.56E-150	0.83114892	0.629	0.263	5.04E-146	9
Atp5e4	2.23E-150	0.72825538	0.957	0.688	7.20E-146	9
S100a101	5.76E-150	0.81418715	0.82	0.389	1.86E-145	9
Ptprc4	6.92E-150	0.82700792	0.637	0.258	2.23E-145	9
Vsir4	1.34E-149	0.80165421	0.414	0.129	4.33E-145	9
Atp6v0e1	2.03E-149	0.78317608	0.668	0.288	6.55E-145	9
Vamp81	4.24E-149	0.78075097	0.642	0.265	1.37E-144	9
Rps94	2.07E-148	0.66922275	0.992	0.871	6.68E-144	9
Il17ra	1.68E-146	0.72097031	0.312	0.079	5.43E-142	9
Atp2b1	4.88E-146	0.69059617	0.405	0.12	1.58E-141	9
Bax	6.03E-146	0.66607547	0.439	0.14	1.95E-141	9
Jarid2	1.14E-145	0.63095216	0.276	0.065	3.67E-141	9
Eif3k2	1.66E-144	0.74892732	0.689	0.295	5.37E-140	9
Hint12	2.38E-144	0.79771842	0.821	0.391	7.67E-140	9
Mcl12	7.85E-144	0.74524659	0.764	0.359	2.54E-139	9
Mcts1	9.11E-144	0.71388282	0.348	0.099	2.94E-139	9

Bloc1s11	4.78E-143	0.7710576	0.507	0.188	1.54E-138	9
Efhd22	1.59E-141	0.8074753	0.448	0.154	5.13E-137	9
Dleu21	5.67E-141	0.73914989	0.407	0.128	1.83E-136	9
Uqcrb2	3.02E-140	0.73973669	0.704	0.308	9.76E-136	9
Cdc423	4.66E-140	0.79759946	0.841	0.472	1.50E-135	9
Ndufb51	4.11E-139	0.69048841	0.568	0.216	1.33E-134	9
Csf2ra2	9.61E-139	0.71805105	0.347	0.1	3.10E-134	9
Fam89b	5.47E-138	0.62154596	0.28	0.069	1.77E-133	9
Cmpk11	5.68E-138	0.7404073	0.437	0.149	1.83E-133	9
Rap1a	1.68E-137	0.75867891	0.487	0.177	5.41E-133	9
Ndufb101	2.61E-137	0.68207489	0.583	0.231	8.43E-133	9
Nme22	3.94E-137	0.68459103	0.858	0.393	1.27E-132	9
Igsf61	5.30E-137	0.72106046	0.276	0.07	1.71E-132	9
Arf5	7.20E-137	0.70320698	0.724	0.346	2.32E-132	9
Rpl35a4	8.34E-137	0.64376157	0.994	0.851	2.69E-132	9
Mapkapk2	3.66E-136	0.69935167	0.36	0.109	1.18E-131	9
Eif3f3	5.12E-136	0.67882944	0.661	0.275	1.65E-131	9
Myo1f1	9.28E-136	0.73849883	0.3	0.08	3.00E-131	9
Gnai22	2.02E-135	0.79089045	0.852	0.478	6.53E-131	9
Uqcrq1	1.23E-134	0.74891023	0.794	0.395	3.97E-130	9
Ncf43	1.35E-134	0.7453515	0.453	0.159	4.35E-130	9
Aprt2	2.67E-134	0.81830121	0.634	0.277	8.61E-130	9
Myadm	4.75E-134	0.46701781	0.367	0.103	1.53E-129	9
Ndufa15	4.83E-134	0.76914057	0.723	0.344	1.56E-129	9
Ppp2r5a3	3.54E-133	0.70396348	0.508	0.191	1.14E-128	9
Arpc45	7.40E-133	0.74965277	0.595	0.253	2.39E-128	9
Cycs2	2.00E-132	0.77876717	0.568	0.229	6.46E-128	9
Cisd11	3.24E-132	0.63257462	0.311	0.084	1.05E-127	9
Pirb4	3.40E-132	0.74857057	0.441	0.15	1.10E-127	9
Arpc33	2.61E-131	0.74223734	0.88	0.503	8.44E-127	9
Tmem1671	2.83E-131	0.66900941	0.552	0.218	9.15E-127	9
Tor1a	1.14E-130	0.60042214	0.254	0.062	3.67E-126	9
Cox6b12	4.63E-130	0.76540184	0.85	0.484	1.49E-125	9
Sell2	9.37E-130	0.60349304	0.492	0.18	3.03E-125	9
Lamtor21	1.13E-129	0.75376537	0.6	0.256	3.66E-125	9
Psme2	6.63E-129	0.63762241	0.476	0.173	2.14E-124	9
Esyt1	1.83E-128	0.56019417	0.26	0.064	5.91E-124	9
Nedd82	3.52E-128	0.68786442	0.726	0.34	1.14E-123	9
Sdf2l1	3.69E-128	0.58656003	0.253	0.061	1.19E-123	9
Actg14	5.80E-128	0.60798945	0.959	0.705	1.87E-123	9
Rpl37a4	6.88E-128	0.58457016	0.998	0.901	2.22E-123	9
Slc25a52	1.31E-127	0.64656156	0.621	0.256	4.22E-123	9
Sh3bgrl1	2.33E-127	0.60361305	0.478	0.171	7.52E-123	9

Cox8a2	5.61E-127	0.69215421	0.936	0.676	1.81E-122	9
Atp5c12	5.69E-127	0.69799154	0.621	0.264	1.84E-122	9
Clec12a2	6.24E-127	0.76585272	0.39	0.129	2.02E-122	9
Thbs13	7.91E-126	0.64739553	0.537	0.204	2.55E-121	9
Flna4	8.38E-126	0.7238676	0.676	0.305	2.71E-121	9
Cdk2ap22	1.17E-125	0.75899237	0.62	0.274	3.78E-121	9
Srsf91	1.26E-125	0.63305539	0.483	0.179	4.07E-121	9
Id2	1.36E-125	0.62201107	0.253	0.061	4.40E-121	9
Tmem2561	2.04E-125	0.71079082	0.612	0.262	6.59E-121	9
Akr1a11	6.20E-125	0.55544189	0.47	0.165	2.00E-120	9
Tln1	1.13E-124	0.72884909	0.578	0.243	3.65E-120	9
Tomm71	6.32E-124	0.68449089	0.865	0.468	2.04E-119	9
Mrps241	1.95E-123	0.61942328	0.452	0.161	6.28E-119	9
Atp6v1f3	2.97E-123	0.63555241	0.677	0.31	9.58E-119	9
Sdhb1	3.56E-123	0.64046531	0.453	0.165	1.15E-118	9
Abracl1	2.18E-122	0.7372556	0.605	0.267	7.03E-118	9
Cfp	2.56E-122	0.47638253	0.275	0.072	8.27E-118	9
Ost41	5.51E-122	0.69630483	0.706	0.335	1.78E-117	9
Trappc2l1	6.60E-122	0.65762524	0.387	0.131	2.13E-117	9
Fosl23	1.43E-121	0.63289662	0.42	0.142	4.62E-117	9
Myl12b3	1.68E-121	0.7294886	0.785	0.409	5.44E-117	9
Tomm62	7.36E-121	0.69170091	0.791	0.405	2.38E-116	9
Chmp4b1	7.83E-121	0.65693355	0.585	0.248	2.53E-116	9
Vim2	1.41E-120	0.49331681	0.879	0.475	4.56E-116	9
Plin2	1.64E-120	0.63094006	0.32	0.093	5.28E-116	9
Pfdn53	3.65E-120	0.67412401	0.828	0.445	1.18E-115	9
Cebpd2	4.42E-120	0.72402243	0.371	0.124	1.43E-115	9
Mrpl301	5.02E-120	0.59572175	0.459	0.168	1.62E-115	9
Rpl93	5.94E-120	0.56847116	0.983	0.766	1.92E-115	9
S100a131	7.25E-120	0.64103166	0.62	0.277	2.34E-115	9
Klf61	1.05E-119	0.7139748	0.548	0.223	3.40E-115	9
Fyb	1.44E-119	0.73286866	0.263	0.068	4.66E-115	9
Anxa52	5.50E-119	0.39292804	0.608	0.237	1.78E-114	9
Polr2l1	6.37E-119	0.67789657	0.372	0.122	2.06E-114	9
Mrpl58	3.05E-118	0.5912099	0.282	0.079	9.85E-114	9
Atp5d2	5.31E-118	0.67714176	0.606	0.26	1.72E-113	9
Atp5mpl2	7.78E-118	0.71111806	0.744	0.361	2.51E-113	9
Ap2a2	1.06E-117	0.68011923	0.365	0.119	3.41E-113	9
Ier32	1.82E-117	0.8110672	0.395	0.138	5.88E-113	9
Irf8	2.94E-117	0.33904224	0.255	0.063	9.48E-113	9
Ndufa31	3.03E-116	0.71377742	0.756	0.396	9.78E-112	9
Pnp	4.22E-115	0.71330729	0.366	0.123	1.36E-110	9
Srp91	1.83E-114	0.6494905	0.58	0.251	5.92E-110	9

Ndufc21	1.95E-114	0.57018493	0.537	0.216	6.29E-110	9
Laptm4a	5.33E-114	0.51955839	0.412	0.142	1.72E-109	9
Rac11	5.66E-114	0.58952854	0.63	0.28	1.83E-109	9
Tnfaip21	5.85E-114	0.37108462	0.47	0.174	1.89E-109	9
Plekho2	1.32E-113	0.52303371	0.28	0.077	4.26E-109	9
Ifnar2	2.30E-113	0.55201297	0.324	0.1	7.43E-109	9
Mrpl523	4.14E-113	0.54094387	0.678	0.287	1.34E-108	9
Nmt11	3.71E-112	0.61863221	0.493	0.197	1.20E-107	9
H2afy2	2.46E-111	0.53529235	0.566	0.229	7.96E-107	9
Zfand5	2.77E-111	0.76101483	0.467	0.183	8.94E-107	9
Tmem50a	3.70E-111	0.57501404	0.464	0.177	1.20E-106	9
Asah1	5.23E-111	0.45132021	0.333	0.1	1.69E-106	9
Sec11c2	1.42E-110	0.60608671	0.637	0.299	4.57E-106	9
Sf3b51	1.62E-110	0.56580108	0.505	0.201	5.22E-106	9
Psmb10	4.31E-110	0.55554055	0.354	0.115	1.39E-105	9
Naca3	5.58E-110	0.63054606	0.925	0.56	1.80E-105	9
Actr22	5.89E-110	0.64697154	0.638	0.301	1.90E-105	9
Rps27l2	1.02E-109	0.60580599	0.721	0.333	3.28E-105	9
Cuta1	1.12E-109	0.56654433	0.437	0.163	3.63E-105	9
Psmb21	1.55E-109	0.61903364	0.524	0.216	5.00E-105	9
Bola22	3.41E-109	0.53252414	0.568	0.23	1.10E-104	9
Serp13	7.25E-109	0.54486592	0.622	0.279	2.34E-104	9
Rpl193	1.01E-108	0.57672982	0.991	0.753	3.25E-104	9
Cib1	1.05E-108	0.48689462	0.37	0.126	3.39E-104	9
Rpl293	1.62E-108	0.6150274	0.919	0.562	5.23E-104	9
Ppib1	4.23E-107	0.58065327	0.685	0.322	1.37E-102	9
Cenpx1	9.37E-107	0.63010233	0.362	0.127	3.02E-102	9
Tmem2583	1.58E-106	0.67488467	0.731	0.373	5.09E-102	9
Cope	1.59E-106	0.54061918	0.434	0.164	5.14E-102	9
Mpc1	1.95E-106	0.60975624	0.441	0.173	6.30E-102	9
9-Sep	6.03E-106	0.567955	0.3	0.091	1.95E-101	9
Gmfg5	1.10E-105	0.57610825	0.77	0.39	3.56E-101	9
Ndufs81	1.33E-105	0.52123185	0.435	0.164	4.29E-101	9
Ndufa41	3.58E-105	0.65056051	0.822	0.451	1.16E-100	9
Ndufb84	5.04E-105	0.60905445	0.617	0.283	1.63E-100	9
Rhoa1	5.81E-104	0.67429461	0.786	0.439	1.87E-99	9
Uqcrfs11	1.29E-103	0.51511366	0.428	0.162	4.16E-99	9
Stk17b3	3.00E-103	0.42300013	0.666	0.307	9.68E-99	9
Kdm6b1	6.05E-103	0.61582874	0.429	0.163	1.95E-98	9
Atp1b3	1.47E-102	0.49547604	0.375	0.129	4.73E-98	9
Fis12	2.88E-102	0.70138172	0.691	0.361	9.30E-98	9
Akap133	7.57E-102	0.56029492	0.547	0.235	2.44E-97	9
Kdm7a2	1.53E-101	0.54030185	0.356	0.121	4.93E-97	9

Tmpo1	2.37E-101	0.61417173	0.424	0.162	7.65E-97	9
BC005537	1.05E-100	0.53310708	0.5	0.206	3.40E-96	9
Itgb24	2.16E-100	0.66950967	0.429	0.168	6.98E-96	9
Tor1aip1	2.72E-100	0.54446701	0.362	0.127	8.78E-96	9
Psmb32	2.81E-100	0.58152318	0.611	0.284	9.08E-96	9
Edf12	4.91E-100	0.57256786	0.631	0.3	1.59E-95	9
Uqcr101	5.36E-100	0.5888502	0.74	0.378	1.73E-95	9
Dnajc191	6.32E-100	0.60122272	0.362	0.129	2.04E-95	9
Ndufb21	7.20E-100	0.52643198	0.516	0.214	2.32E-95	9
Fgr	2.63E-99	0.70291681	0.259	0.075	8.49E-95	9
Tnfrsf1a2	4.68E-99	0.52348435	0.317	0.103	1.51E-94	9
Zfp36l21	5.80E-99	0.61260116	0.567	0.249	1.87E-94	9
Fkbp21	1.36E-98	0.54729035	0.399	0.15	4.39E-94	9
Sf3b61	6.65E-98	0.56583057	0.564	0.253	2.15E-93	9
Cyp4f182	7.12E-98	0.41517371	0.288	0.085	2.30E-93	9
Cox6c3	8.79E-98	0.59820801	0.917	0.628	2.84E-93	9
Taf101	1.28E-97	0.56200893	0.513	0.22	4.13E-93	9
Ncf23	1.33E-97	0.49752293	0.524	0.226	4.29E-93	9
Ndufa81	1.34E-97	0.55406607	0.424	0.168	4.33E-93	9
Nsa23	1.79E-97	0.57960256	0.854	0.468	5.78E-93	9
Tmed10	3.27E-97	0.54199098	0.5	0.213	1.06E-92	9
Cd534	3.29E-97	0.51760666	0.56	0.247	1.06E-92	9
Dctn31	4.69E-97	0.59481835	0.418	0.167	1.52E-92	9
Psmd81	1.06E-96	0.49528913	0.457	0.184	3.43E-92	9
Eif3h3	2.24E-96	0.45806909	0.594	0.264	7.24E-92	9
Atp5g11	7.64E-96	0.49289375	0.616	0.272	2.47E-91	9
Sft2d1	8.07E-96	0.60601267	0.291	0.095	2.60E-91	9
H2-T23	8.40E-96	0.55176506	0.423	0.164	2.71E-91	9
Cmc1	1.54E-95	0.46571518	0.254	0.075	4.96E-91	9
Dad12	2.22E-95	0.44962981	0.547	0.232	7.17E-91	9
Zyx3	1.18E-94	0.57591501	0.449	0.183	3.80E-90	9
Ndufb112	1.37E-94	0.59620854	0.632	0.312	4.42E-90	9
Cdkn2d1	1.52E-94	0.56845773	0.385	0.147	4.91E-90	9
Mrps211	3.52E-94	0.54692806	0.636	0.309	1.14E-89	9
Hk2	4.61E-94	0.53395183	0.291	0.095	1.49E-89	9
Ostf14	6.32E-94	0.56867327	0.647	0.317	2.04E-89	9
Higd2a	1.20E-93	0.55393198	0.451	0.186	3.88E-89	9
Rnasek2	2.13E-93	0.555831	0.583	0.278	6.87E-89	9
Psmg4	6.28E-93	0.46138226	0.321	0.108	2.03E-88	9
Degs13	7.56E-93	0.64537522	0.407	0.16	2.44E-88	9
Tmco11	8.00E-93	0.50840661	0.394	0.15	2.58E-88	9
Cox7b3	1.62E-92	0.52621309	0.679	0.331	5.24E-88	9
Anxa23	2.17E-92	0.43035501	0.781	0.406	7.01E-88	9

Fam111a1	2.73E-92	0.54620383	0.39	0.149	8.82E-88	9
Gpx41	3.52E-92	0.61466065	0.72	0.386	1.14E-87	9
Gsr3	6.17E-92	0.52223991	0.628	0.298	1.99E-87	9
Eif4ebp1	8.57E-92	0.53154282	0.409	0.16	2.77E-87	9
Plaur4	1.59E-91	0.37560054	0.666	0.306	5.12E-87	9
Rpl184	1.86E-91	0.53580107	0.969	0.705	6.01E-87	9
Actr34	7.04E-91	0.58113208	0.656	0.334	2.27E-86	9
Lamp23	1.00E-90	0.43879556	0.495	0.211	3.23E-86	9
Ndufa61	1.23E-90	0.46874417	0.641	0.31	3.96E-86	9
Eno11	1.41E-90	0.50961122	0.563	0.261	4.56E-86	9
Plek2	1.53E-90	0.44392911	0.387	0.146	4.93E-86	9
Mrps141	1.73E-90	0.48286734	0.445	0.182	5.58E-86	9
D8Ertd738e3	3.55E-90	0.55463424	0.738	0.412	1.15E-85	9
Uqcr112	3.83E-90	0.49650903	0.703	0.347	1.24E-85	9
Ier23	5.34E-90	0.56348185	0.569	0.268	1.72E-85	9
Xdh3	8.15E-90	0.61838096	0.296	0.1	2.63E-85	9
Znhit1	9.39E-90	0.59235682	0.443	0.187	3.03E-85	9
Scp22	3.36E-89	0.59014635	0.486	0.215	1.09E-84	9
Arhgap30	6.65E-89	0.56486968	0.333	0.119	2.15E-84	9
Rhog3	3.19E-88	0.56447158	0.406	0.165	1.03E-83	9
Rab5if3	9.50E-88	0.59259865	0.546	0.258	3.07E-83	9
Lamtor51	4.70E-87	0.46789129	0.34	0.125	1.52E-82	9
Otulin	2.19E-86	0.51522297	0.313	0.109	7.06E-82	9
Magohb	3.34E-86	0.51226471	0.26	0.083	1.08E-81	9
M6pr	4.00E-86	0.44494625	0.262	0.083	1.29E-81	9
Psmb51	4.12E-86	0.47216944	0.495	0.218	1.33E-81	9
Gng52	4.74E-86	0.57674814	0.924	0.643	1.53E-81	9
Skap22	5.78E-86	0.53661438	0.371	0.144	1.87E-81	9
Rpl394	7.53E-86	0.49486478	0.994	0.841	2.43E-81	9
Psme13	1.15E-85	0.4496942	0.62	0.296	3.72E-81	9
Rplp02	4.15E-85	0.51453974	0.978	0.73	1.34E-80	9
Fermt3	5.50E-85	0.57483149	0.34	0.13	1.78E-80	9
Ap2s11	7.53E-85	0.51628591	0.432	0.184	2.43E-80	9
Rps184	8.49E-85	0.46602198	0.966	0.639	2.74E-80	9
Ufc1	9.23E-85	0.46328823	0.313	0.111	2.98E-80	9
Cited21	1.21E-84	0.57351522	0.309	0.11	3.90E-80	9
Nfam13	2.84E-84	0.58795186	0.312	0.113	9.17E-80	9
Snrpb3	1.03E-83	0.4855867	0.575	0.267	3.34E-79	9
Tgfb11	1.34E-83	0.47627463	0.386	0.152	4.34E-79	9
Cox5a2	1.56E-83	0.46840693	0.729	0.377	5.05E-79	9
Cmtm72	2.02E-83	0.27817094	0.528	0.229	6.52E-79	9
Lsm51	3.52E-83	0.53199678	0.489	0.217	1.14E-78	9
Rps54	7.80E-83	0.49012966	0.978	0.672	2.52E-78	9

Ube2l31	8.87E-83	0.52113969	0.453	0.198	2.87E-78	9
Timm131	1.87E-82	0.42182898	0.509	0.225	6.04E-78	9
Tma71	3.91E-82	0.55983775	0.698	0.377	1.26E-77	9
Ubl53	4.34E-82	0.54279617	0.834	0.508	1.40E-77	9
Chmp2a	6.00E-82	0.47556843	0.499	0.228	1.94E-77	9
Rgs2	6.08E-82	0.51939946	0.406	0.166	1.96E-77	9
Dock2	6.09E-82	0.46268216	0.279	0.095	1.97E-77	9
Dazap22	8.21E-82	0.45377353	0.47	0.206	2.65E-77	9
Fmnl12	1.90E-81	0.49310187	0.347	0.13	6.12E-77	9
Irf2bp2	2.16E-81	0.363825	0.423	0.174	6.98E-77	9
Rpl113	2.37E-81	0.48177373	0.978	0.732	7.66E-77	9
Hck2	7.57E-81	0.56766773	0.26	0.087	2.44E-76	9
Pin41	8.90E-81	0.39917958	0.356	0.136	2.87E-76	9
Cox161	1.15E-80	0.46185643	0.308	0.111	3.71E-76	9
Mrpl571	1.84E-80	0.4133988	0.421	0.177	5.94E-76	9
H2-D14	2.38E-80	0.54570645	0.934	0.65	7.67E-76	9
Arhgdia1	3.13E-80	0.41619054	0.476	0.208	1.01E-75	9
Sod2	3.92E-80	0.57867454	0.309	0.113	1.26E-75	9
Siva11	6.36E-80	0.50268994	0.381	0.151	2.05E-75	9
Higd1a1	1.35E-79	0.40563347	0.424	0.177	4.34E-75	9
Ptpre1	2.02E-79	0.59862107	0.297	0.108	6.53E-75	9
Mef2a	3.05E-79	0.43188381	0.276	0.093	9.83E-75	9
Picalm2	3.93E-78	0.36109286	0.488	0.214	1.27E-73	9
Rps193	5.64E-78	0.42224224	0.994	0.722	1.82E-73	9
Rps133	7.35E-78	0.42388247	0.977	0.79	2.37E-73	9
Clic11	1.39E-77	0.53921925	0.778	0.461	4.49E-73	9
Ndufb41	1.73E-77	0.45798601	0.501	0.23	5.58E-73	9
Cnih4	4.52E-77	0.46448482	0.4	0.168	1.46E-72	9
Ndufa131	6.70E-77	0.4770978	0.745	0.417	2.16E-72	9
Emc7	1.04E-76	0.38688712	0.283	0.099	3.36E-72	9
Rps15a3	3.13E-76	0.41609488	0.989	0.779	1.01E-71	9
Atp6v0d1	3.79E-76	0.49160306	0.354	0.142	1.22E-71	9
Cox6a12	4.57E-76	0.44840254	0.61	0.306	1.47E-71	9
Atp5f12	5.67E-76	0.52127508	0.598	0.311	1.83E-71	9
Rps244	7.40E-76	0.39311521	0.996	0.85	2.39E-71	9
Rpl344	7.59E-76	0.45291834	0.988	0.82	2.45E-71	9
Cox14	1.03E-75	0.40295297	0.399	0.167	3.34E-71	9
AI6622701	1.28E-75	0.53567315	0.335	0.132	4.13E-71	9
Atp6v1g13	2.68E-75	0.45414773	0.637	0.333	8.67E-71	9
Rer11	2.90E-75	0.44939168	0.346	0.138	9.37E-71	9
Tmem234	3.46E-75	0.37251991	0.359	0.142	1.12E-70	9
Rassf32	4.60E-75	0.4440446	0.265	0.09	1.48E-70	9
Rgcc1	5.44E-75	0.80204184	0.312	0.122	1.76E-70	9

Pcbd21	6.68E-75	0.48700014	0.286	0.105	2.16E-70	9
Ostc	6.75E-75	0.39486413	0.408	0.172	2.18E-70	9
Polr1d3	8.40E-75	0.36875096	0.596	0.289	2.71E-70	9
Cbl	1.06E-74	0.54187952	0.312	0.119	3.42E-70	9
Gda2	1.47E-74	0.45025943	0.451	0.2	4.76E-70	9
Tacc11	3.73E-74	0.41581941	0.362	0.148	1.20E-69	9
Ier3ip1	4.57E-74	0.41795335	0.428	0.187	1.47E-69	9
Celf21	6.48E-74	0.45132756	0.407	0.175	2.09E-69	9
Arl6ip11	7.48E-74	0.50892584	0.442	0.201	2.41E-69	9
Iscu1	1.30E-73	0.45869111	0.324	0.126	4.20E-69	9
Capns1	1.52E-73	0.41667442	0.433	0.191	4.92E-69	9
Irf2	1.74E-73	0.39157848	0.362	0.145	5.63E-69	9
Rps284	1.80E-73	0.4983028	0.997	0.84	5.82E-69	9
Gm118081	2.72E-73	0.37675302	0.394	0.164	8.79E-69	9
Gnb21	2.90E-73	0.50610764	0.652	0.357	9.36E-69	9
Snrpg3	2.93E-73	0.41868772	0.876	0.493	9.46E-69	9
Vapa	5.18E-73	0.46339305	0.479	0.225	1.67E-68	9
Oaz13	5.86E-73	0.46020393	0.9	0.63	1.89E-68	9
Rexo2	1.06E-72	0.30793076	0.291	0.104	3.43E-68	9
Tmem59	1.41E-72	0.38485547	0.36	0.146	4.55E-68	9
Naa381	1.55E-72	0.42795833	0.371	0.154	5.02E-68	9
Rpl324	2.31E-72	0.44865214	0.981	0.722	7.47E-68	9
Snf8	3.37E-72	0.3909742	0.276	0.099	1.09E-67	9
Map3k11	3.70E-72	0.30435929	0.253	0.084	1.19E-67	9
Rex1bd1	4.02E-72	0.4525626	0.329	0.13	1.30E-67	9
Uqcrh3	4.11E-72	0.45048812	0.833	0.502	1.33E-67	9
Rpsa3	4.77E-72	0.45126483	0.984	0.692	1.54E-67	9
Rpp211	5.29E-72	0.45930333	0.342	0.139	1.71E-67	9
Cops91	1.73E-71	0.45235903	0.535	0.265	5.59E-67	9
AB1246112	3.21E-71	0.49826592	0.283	0.105	1.04E-66	9
Psma33	3.68E-71	0.35222764	0.524	0.245	1.19E-66	9
Lamtor1	3.96E-71	0.43093633	0.291	0.11	1.28E-66	9
Atp1a1	4.33E-71	0.39275093	0.306	0.113	1.40E-66	9
H13	6.14E-71	0.36401738	0.333	0.13	1.98E-66	9
Psmb91	1.26E-70	0.4000632	0.37	0.155	4.05E-66	9
Sarnp1	1.89E-70	0.41214662	0.501	0.239	6.11E-66	9
Krtcap21	3.09E-70	0.34728291	0.532	0.244	9.96E-66	9
Rbx12	3.67E-70	0.44545647	0.669	0.363	1.18E-65	9
Ndufs51	8.35E-70	0.3810682	0.558	0.276	2.70E-65	9
Myl61	1.50E-69	0.47958148	0.927	0.697	4.85E-65	9
Spcs2	1.74E-69	0.42464393	0.425	0.189	5.60E-65	9
Ndufb72	1.54E-68	0.48421802	0.672	0.37	4.98E-64	9
Rps74	2.13E-68	0.38019893	0.985	0.721	6.87E-64	9

Sptssa1	3.32E-68	0.37077003	0.285	0.107	1.07E-63	9
0610012G03	3.92E-68	0.38601849	0.266	0.097	1.27E-63	9
Sp100	7.35E-68	0.47556248	0.288	0.11	2.37E-63	9
Unc1191	1.07E-67	0.54585516	0.256	0.094	3.45E-63	9
Sik1	1.10E-67	0.3949799	0.313	0.122	3.55E-63	9
Rnaseh2c2	1.25E-67	0.49511692	0.462	0.222	4.05E-63	9
Gm100764	1.67E-67	0.41823302	0.978	0.804	5.40E-63	9
Polr2i1	4.31E-67	0.29152047	0.335	0.135	1.39E-62	9
Llph1	5.02E-67	0.31753337	0.43	0.189	1.62E-62	9
Ubb3	5.45E-67	0.39640135	0.949	0.753	1.76E-62	9
Ifrd11	6.83E-67	0.52216844	0.364	0.156	2.20E-62	9
Mien11	1.01E-65	0.40978089	0.305	0.121	3.27E-61	9
Atp5j1	7.29E-65	0.46154768	0.706	0.404	2.35E-60	9
Hcls12	1.70E-64	0.39955243	0.334	0.14	5.49E-60	9
Rpl304	1.97E-64	0.39189656	0.988	0.812	6.37E-60	9
Ndufa112	2.02E-64	0.35576078	0.512	0.252	6.51E-60	9
Mrps331	2.06E-64	0.33180923	0.418	0.187	6.65E-60	9
Sap181	2.45E-64	0.41968729	0.461	0.224	7.91E-60	9
Prdx64	3.26E-64	0.48790182	0.535	0.275	1.05E-59	9
Btf34	3.56E-64	0.41483678	0.853	0.537	1.15E-59	9
Apbb1ip	4.54E-64	0.47951904	0.265	0.1	1.47E-59	9
Anapc131	4.96E-64	0.40203535	0.419	0.193	1.60E-59	9
Tsc22d32	5.14E-64	0.26618966	0.391	0.17	1.66E-59	9
Mrpl201	8.15E-64	0.38692102	0.415	0.191	2.63E-59	9
Rel1	1.06E-63	0.30280828	0.267	0.098	3.41E-59	9
Pfn13	1.21E-63	0.44196461	0.972	0.741	3.90E-59	9
Arpc54	1.33E-63	0.4529099	0.621	0.342	4.31E-59	9
Rpl274	1.52E-63	0.38376225	0.972	0.716	4.91E-59	9
Rtraf1	1.82E-63	0.27246378	0.459	0.207	5.88E-59	9
Rpl18a4	2.61E-63	0.39121575	0.992	0.8	8.42E-59	9
Tle5	2.99E-63	0.45901643	0.476	0.239	9.64E-59	9
Atp5j22	3.08E-63	0.44218895	0.838	0.531	9.94E-59	9
Grcc101	3.89E-63	0.35739995	0.523	0.261	1.25E-58	9
Supt4a3	4.32E-63	0.36953107	0.535	0.273	1.40E-58	9
Nfkbia3	2.20E-62	0.45380588	0.475	0.231	7.12E-58	9
Psenen1	3.61E-62	0.3694693	0.496	0.247	1.17E-57	9
Chchd22	4.63E-62	0.41104125	0.88	0.57	1.50E-57	9
Trmt1122	8.72E-62	0.33462208	0.491	0.234	2.82E-57	9
Atp5g23	2.01E-61	0.38598754	0.741	0.404	6.48E-57	9
Ndufs41	2.32E-61	0.39339619	0.385	0.176	7.47E-57	9
Cfl13	3.50E-61	0.4768652	0.862	0.611	1.13E-56	9
Rbm33	3.57E-61	0.41546698	0.725	0.417	1.15E-56	9
Uqcrc11	9.63E-61	0.39050862	0.311	0.129	3.11E-56	9

Ndufc12	1.42E-60	0.31229911	0.61	0.315	4.57E-56	9
Cyb5a	3.08E-60	0.33960144	0.359	0.157	9.95E-56	9
Arf12	7.90E-60	0.38150809	0.53	0.276	2.55E-55	9
Sdcbp2	1.21E-59	0.3018826	0.453	0.218	3.90E-55	9
Cox7a22	1.22E-59	0.38011687	0.811	0.493	3.92E-55	9
Etfb	1.24E-59	0.34276073	0.359	0.157	4.00E-55	9
Rpl243	1.26E-59	0.39312831	0.981	0.758	4.05E-55	9
Selenok3	1.31E-59	0.38783482	0.676	0.383	4.21E-55	9
Ssh2	2.46E-59	0.41222944	0.25	0.093	7.94E-55	9
Mbnl11	2.80E-59	0.30870958	0.597	0.318	9.05E-55	9
Tmem179b1	4.45E-59	0.42371551	0.28	0.113	1.44E-54	9
Dpm1	4.75E-59	0.36786977	0.287	0.116	1.53E-54	9
Mfsd14b1	5.74E-59	0.45326041	0.251	0.096	1.85E-54	9
1810037I17R	7.13E-59	0.46099129	0.618	0.34	2.30E-54	9
Osm3	1.05E-58	0.3390515	0.274	0.107	3.38E-54	9
Micos102	1.08E-58	0.31180954	0.524	0.261	3.47E-54	9
Erh2	1.54E-58	0.25872962	0.474	0.219	4.96E-54	9
Twf21	1.84E-58	0.41339141	0.256	0.1	5.94E-54	9
Tiparp	2.25E-58	0.44039687	0.283	0.113	7.27E-54	9
Ube2a1	3.97E-58	0.37281878	0.379	0.176	1.28E-53	9
Psma51	4.15E-58	0.27607994	0.386	0.173	1.34E-53	9
Ndufs71	4.50E-58	0.36615336	0.384	0.177	1.45E-53	9
Rps4x4	4.83E-58	0.37410748	0.969	0.685	1.56E-53	9
Ndufv21	5.04E-58	0.33036788	0.36	0.161	1.63E-53	9
Rpl225	1.04E-57	0.37686104	0.963	0.656	3.34E-53	9
Cisd21	1.09E-57	0.32257003	0.315	0.134	3.53E-53	9
Atp5o2	1.23E-57	0.32073037	0.481	0.238	3.96E-53	9
Psma22	2.64E-57	0.35375303	0.571	0.308	8.52E-53	9
Ndufb92	2.95E-57	0.35760382	0.633	0.352	9.52E-53	9
Psmb13	3.89E-57	0.26170183	0.569	0.294	1.26E-52	9
Bcl102	4.55E-57	0.40752847	0.383	0.18	1.47E-52	9
Lilrb4a3	7.00E-57	0.26494393	0.376	0.169	2.26E-52	9
Tapbp	7.72E-57	0.35011196	0.29	0.118	2.49E-52	9
Arhgap451	8.01E-57	0.33344911	0.343	0.152	2.58E-52	9
Ywhab2	9.27E-57	0.38862105	0.558	0.302	2.99E-52	9
Cox4i12	1.46E-56	0.32605483	0.826	0.512	4.71E-52	9
Msn1	3.15E-56	0.37558684	0.571	0.305	1.02E-51	9
Atp5g31	3.75E-56	0.32903507	0.519	0.262	1.21E-51	9
Ppia2	1.55E-55	0.42524451	0.97	0.701	5.00E-51	9
Nadk1	1.74E-55	0.41651943	0.267	0.108	5.61E-51	9
Cox7c4	2.22E-55	0.35571448	0.935	0.662	7.16E-51	9
Mpc23	1.92E-54	0.32176278	0.48	0.246	6.20E-50	9
Snx31	2.28E-54	0.2864231	0.38	0.177	7.36E-50	9

Smim111	2.35E-54	0.3590232	0.31	0.134	7.58E-50	9
Sec11a1	3.77E-54	0.31335864	0.3	0.128	1.22E-49	9
Pgd3	4.81E-54	0.41023719	0.357	0.167	1.55E-49	9
Commd11	5.73E-54	0.35804173	0.314	0.14	1.85E-49	9
Stk38	6.49E-54	0.41792749	0.271	0.111	2.10E-49	9
Mcemp14	7.37E-54	0.28251444	0.438	0.21	2.38E-49	9
Psma4	1.90E-53	0.25505304	0.371	0.169	6.12E-49	9
Psma11	1.93E-53	0.29499371	0.342	0.155	6.23E-49	9
Grb2	2.49E-53	0.36171971	0.352	0.164	8.02E-49	9
Gabarap2	2.50E-53	0.38743192	0.752	0.471	8.08E-49	9
Rab8b3	2.90E-53	0.3898176	0.371	0.175	9.37E-49	9
Itgam4	4.96E-53	0.36955268	0.375	0.177	1.60E-48	9
Atp6v1e14	7.00E-53	0.34234639	0.616	0.34	2.26E-48	9
Polr2j1	7.34E-53	0.26738214	0.372	0.173	2.37E-48	9
Ndufb61	7.76E-53	0.34620294	0.313	0.139	2.51E-48	9
Ndufa71	9.59E-53	0.33034638	0.72	0.42	3.10E-48	9
Ddost	2.21E-52	0.27563544	0.275	0.113	7.15E-48	9
Rps34	3.20E-52	0.32776001	0.952	0.699	1.03E-47	9
Mrpl541	2.33E-51	0.26558952	0.311	0.136	7.52E-47	9
Eif3i1	2.49E-51	0.27399345	0.361	0.165	8.05E-47	9
Rpl132	2.95E-51	0.36614945	0.995	0.786	9.53E-47	9
Slk	5.93E-51	0.28569968	0.276	0.115	1.92E-46	9
Lman2	7.11E-51	0.29593646	0.28	0.119	2.30E-46	9
Grpel11	7.14E-51	0.31185599	0.286	0.123	2.30E-46	9
Selenos	1.38E-50	0.27900806	0.284	0.121	4.46E-46	9
Rpl10a4	1.88E-50	0.32261347	0.94	0.593	6.07E-46	9
Mrps161	4.09E-50	0.31132501	0.336	0.155	1.32E-45	9
Syf21	5.91E-50	0.26492373	0.399	0.195	1.91E-45	9
Ndufs63	1.20E-49	0.2762991	0.482	0.251	3.86E-45	9
Aurkaip11	1.56E-49	0.33482525	0.35	0.167	5.05E-45	9
Rpl384	1.78E-49	0.35345093	0.999	0.837	5.74E-45	9
Hprt1	2.17E-49	0.32835238	0.274	0.117	6.99E-45	9
2310009A05	4.43E-49	0.36596802	0.267	0.116	1.43E-44	9
Rasgrp21	7.19E-49	0.28315031	0.313	0.143	2.32E-44	9
Prelid12	2.88E-48	0.34233543	0.476	0.254	9.31E-44	9
Rab5c	3.22E-48	0.35150499	0.272	0.118	1.04E-43	9
Nop53	3.38E-48	0.26880775	0.288	0.124	1.09E-43	9
Atp5k3	3.46E-48	0.35741789	0.781	0.499	1.12E-43	9
Srp191	6.33E-48	0.29631205	0.339	0.159	2.04E-43	9
Snrpd32	8.02E-48	0.27350095	0.499	0.259	2.59E-43	9
Ubl3	1.41E-47	0.25744127	0.268	0.112	4.55E-43	9
Ppp1ca3	1.64E-47	0.28202845	0.626	0.36	5.30E-43	9
Ssu72	1.92E-47	0.34146381	0.354	0.174	6.20E-43	9

Tmbim4	2.02E-47	0.31686563	0.291	0.132	6.52E-43	9
Glipr23	2.45E-47	0.3369604	0.254	0.108	7.92E-43	9
Diaph11	5.63E-47	0.33666267	0.314	0.145	1.82E-42	9
Ndufa23	9.60E-47	0.30494394	0.71	0.426	3.10E-42	9
Cwc151	2.36E-46	0.29031946	0.363	0.177	7.64E-42	9
Rpl7a3	2.77E-46	0.27836303	0.877	0.548	8.94E-42	9
Tuba1c	3.02E-46	0.32792697	0.265	0.115	9.74E-42	9
Ubc1	3.64E-46	0.31020347	0.677	0.408	1.18E-41	9
Alyref1	3.92E-46	0.30937829	0.431	0.218	1.27E-41	9
Arhgef1	6.25E-46	0.3436293	0.303	0.14	2.02E-41	9
Ccnl12	6.88E-46	0.29610116	0.454	0.242	2.22E-41	9
Coa31	9.20E-46	0.26508318	0.272	0.12	2.97E-41	9
Csnk2b1	1.11E-45	0.28484501	0.384	0.194	3.58E-41	9
Tomm221	1.45E-45	0.2844519	0.415	0.214	4.69E-41	9
Spop	1.64E-45	0.25949304	0.263	0.113	5.30E-41	9
Ndufv32	4.52E-45	0.32467886	0.458	0.246	1.46E-40	9
Lmo45	4.69E-45	0.28847231	0.466	0.253	1.51E-40	9
Smim4	2.74E-44	0.39079751	0.333	0.164	8.83E-40	9
Cdc42se11	2.73E-43	0.29439725	0.343	0.167	8.82E-39	9
Vps28	3.54E-43	0.27213079	0.332	0.162	1.14E-38	9
Sp31	4.24E-43	0.30685653	0.283	0.13	1.37E-38	9
Ppp2r5c	2.33E-42	0.28713976	0.27	0.121	7.53E-38	9
Ccnd32	4.69E-42	0.275874	0.371	0.19	1.52E-37	9
Rpl284	1.13E-41	0.29387852	0.978	0.752	3.66E-37	9
Rps165	2.62E-41	0.29980501	0.982	0.771	8.46E-37	9
Rps104	5.12E-40	0.28495223	0.985	0.798	1.65E-35	9
Rabac1	5.23E-40	0.29980134	0.382	0.202	1.69E-35	9
Sh3kbp1	5.55E-40	0.27635247	0.254	0.115	1.79E-35	9
Rpl364	4.76E-39	0.27850668	0.994	0.753	1.54E-34	9
Rpl27a3	5.99E-39	0.29588864	0.989	0.793	1.93E-34	9
Mob1a	1.15E-38	0.27808775	0.319	0.159	3.71E-34	9
Tmed5	1.81E-38	0.33535283	0.301	0.149	5.86E-34	9
Gtf2b	3.68E-37	0.27419519	0.251	0.117	1.19E-32	9
Gapdh4	1.20E-36	0.29012424	0.798	0.536	3.88E-32	9
Rps264	3.11E-35	0.2620608	0.989	0.781	1.00E-30	9
Tmbim62	3.27E-35	0.26320642	0.492	0.291	1.06E-30	9
Elob2	2.41E-34	0.26521814	0.806	0.558	7.80E-30	9
Rpl354	8.65E-34	0.29156639	0.989	0.767	2.79E-29	9
Rpl373	1.78E-33	0.25059783	0.995	0.913	5.76E-29	9
Hmgb22	7.55E-30	0.45149629	0.862	0.633	2.44E-25	9
Cenpa1	6.98E-25	0.322392	0.253	0.137	2.25E-20	9
Hbb-bs	0	6.4222931	1	0.209	0	10
Hba-a2	0	6.39460085	1	0.079	0	10

Hbb-bt	0	6.19414531	1	0.081	0	10
Hba-a1	0	5.90171994	1	0.078	0	10
Tmcc2	0	4.61708988	0.726	0.014	0	10
Bpgm	0	3.62715799	0.764	0.025	0	10
Alas2	0	3.54923375	0.845	0.019	0	10
Xpo7	0	3.49191631	0.635	0.041	0	10
Slc4a1	0	3.33174605	0.816	0.028	0	10
Snca	0	3.32724836	0.765	0.015	0	10
Gypa	0	3.21474329	0.848	0.026	0	10
Car2	0	3.16190688	0.82	0.057	0	10
Mkrn1	0	3.15943055	0.814	0.13	0	10
Tfdp2	0	3.09510861	0.769	0.062	0	10
Tent5c	0	3.04402597	0.699	0.026	0	10
Rsad2	0	3.02382654	0.538	0.017	0	10
Hmbs	0	3.00272695	0.649	0.058	0	10
Epb41	0	2.82324846	0.712	0.066	0	10
Isg15	0	2.80496659	0.371	0.048	0	10
Slc25a37	0	2.7242828	0.696	0.072	0	10
Prxl2a	0	2.4806874	0.527	0.021	0	10
Gpx12	0	2.45814433	0.987	0.499	0	10
Cd36	0	2.41271642	0.399	0.009	0	10
Ppp1r15a	0	2.3839721	0.578	0.097	0	10
Trim10	0	2.37937539	0.456	0.009	0	10
Ube2l6	0	2.35673656	0.552	0.033	0	10
Rec114	0	2.35294254	0.437	0.009	0	10
Clcn3	0	2.24033241	0.427	0.066	0	10
Isg20	0	2.23901664	0.578	0.051	0	10
Fech	0	2.23189195	0.537	0.039	0	10
Creg11	0	2.21752519	0.631	0.135	0	10
Apol11b	0	2.19651257	0.334	0.004	0	10
Blvrb2	0	2.17367611	0.767	0.131	0	10
Slfn14	0	2.0717425	0.319	0.002	0	10
Kel	0	2.06485361	0.286	0.016	0	10
Glrx5	0	2.04453445	0.631	0.127	0	10
Nt5c3	0	2.04130622	0.43	0.058	0	10
Tspo2	0	2.03764938	0.399	0.015	0	10
Rhd	0	1.99021682	0.324	0.024	0	10
Prdx21	0	1.98822773	0.859	0.308	0	10
Arl4a	0	1.98771807	0.352	0.044	0	10
Ranbp10	0	1.89718629	0.364	0.033	0	10
Ube2o	0	1.89163798	0.393	0.019	0	10
2-Mar	0	1.84953863	0.457	0.053	0	10
Ypel4	0	1.83362283	0.256	0.004	0	10

Rad23a	0	1.78789791	0.472	0.082	0	10
Specc1	0	1.76352127	0.37	0.04	0	10
Sec61g2	0	1.74593083	0.909	0.601	0	10
1600020E01I	0	1.69719534	0.355	0.027	0	10
Urod	0	1.59997167	0.348	0.045	0	10
Slc25a39	0	1.57820292	0.445	0.077	0	10
Fam126a	0	1.55845754	0.304	0.035	0	10
Isca1	0	1.53666865	0.414	0.069	0	10
Hagh	0	1.52377999	0.391	0.063	0	10
Pnpo	0	1.47193188	0.29	0.017	0	10
Cdr2	0	1.44161	0.255	0.01	0	10
Cmas	0	1.4293957	0.354	0.055	0	10
Dhrs11	0	1.42769885	0.269	0.028	0	10
Spta1	0	1.42228222	0.259	0.016	0	10
Bcl2l1	0	1.37982999	0.325	0.045	0	10
Dnajb2	0	1.33368823	0.25	0.024	0	10
Fam210b	0	1.32067359	0.268	0.022	0	10
Fam220a	0	1.30074501	0.27	0.005	0	10
Plaat3	0	1.21983913	0.285	0.035	0	10
Cat	6.79E-308	1.26870222	0.311	0.043	2.19E-303	10
Ube2h	8.97E-307	1.58282245	0.416	0.078	2.90E-302	10
1810058I24R	4.62E-301	1.9001753	0.67	0.234	1.49E-296	10
Bnip3l	1.06E-297	1.77185733	0.642	0.211	3.42E-293	10
Bsdc1	4.47E-295	1.45636338	0.283	0.037	1.44E-290	10
Pabpc11	5.63E-294	1.7936249	0.922	0.572	1.82E-289	10
Rnf10	9.21E-279	1.65953755	0.514	0.134	2.97E-274	10
Trak2	8.58E-269	1.7706495	0.303	0.048	2.77E-264	10
Ctse	4.61E-268	1.62827543	0.262	0.035	1.49E-263	10
Tfrc	2.13E-244	1.90679162	0.416	0.098	6.88E-240	10
Gclm	3.88E-240	1.47872324	0.412	0.095	1.25E-235	10
St3gal52	5.29E-240	1.73594183	0.43	0.107	1.71E-235	10
Slc25a511	3.02E-238	1.38399772	0.307	0.054	9.75E-234	10
Cpeb4	5.65E-230	1.67415565	0.284	0.049	1.82E-225	10
Psme3	3.92E-193	1.36052851	0.302	0.063	1.27E-188	10
Gabarapl21	2.60E-185	1.32755445	0.604	0.248	8.39E-181	10
Dcaf12	1.31E-183	1.23286455	0.296	0.063	4.23E-179	10
Ubb4	1.16E-170	0.95742806	0.924	0.755	3.73E-166	10
Rbm381	7.82E-170	1.56699624	0.435	0.14	2.53E-165	10
Yipf4	7.06E-144	1.22183874	0.315	0.085	2.28E-139	10
Ccndbp1	2.24E-132	0.92653598	0.265	0.065	7.23E-128	10
Cd24a2	6.40E-128	1.03687784	0.761	0.41	2.07E-123	10
Gpcpd1	1.42E-123	1.36973954	0.308	0.091	4.60E-119	10
Metap21	4.39E-120	1.4129246	0.512	0.234	1.42E-115	10

Lmo2	1.14E-111	1.15989076	0.25	0.068	3.67E-107	10
Arf51	2.39E-105	1.15599521	0.635	0.35	7.71E-101	10
Eif51	1.66E-103	1.17484942	0.57	0.294	5.36E-99	10
Ppp1cb1	9.32E-96	1.02628823	0.418	0.178	3.01E-91	10
Cir1	2.20E-84	0.90994913	0.288	0.099	7.12E-80	10
Dennd4a	1.28E-79	1.4487105	0.351	0.155	4.14E-75	10
Zfand6	1.44E-74	0.85493316	0.292	0.11	4.66E-70	10
Ctsb2	1.79E-72	0.79351337	0.472	0.232	5.77E-68	10
Azin1	3.35E-72	1.14403882	0.31	0.128	1.08E-67	10
Ncoa4	1.10E-71	1.06116843	0.251	0.092	3.57E-67	10
Gadd45a4	9.03E-69	1.0337583	0.377	0.178	2.92E-64	10
AI6622702	1.70E-63	0.90427294	0.309	0.133	5.49E-59	10
Ube2b2	2.07E-61	0.78215991	0.502	0.281	6.67E-57	10
Ucp24	3.26E-61	0.59192203	0.593	0.342	1.05E-56	10
Nusap11	8.23E-57	0.63583833	0.307	0.13	2.66E-52	10
Atpif11	1.28E-56	0.87821642	0.643	0.417	4.13E-52	10
Bola31	3.64E-56	0.84974769	0.275	0.116	1.18E-51	10
Ube2c1	1.79E-53	0.59520423	0.337	0.152	5.76E-49	10
Map1lc3b3	1.70E-51	0.55396345	0.641	0.427	5.49E-47	10
Ube2r2	7.85E-50	0.74202729	0.268	0.117	2.53E-45	10
Wdr26	1.34E-48	0.98093023	0.324	0.164	4.34E-44	10
Bsg1	1.61E-48	0.85792451	0.437	0.249	5.20E-44	10
Adipor12	5.00E-48	0.73302409	0.348	0.176	1.61E-43	10
Pnp1	1.96E-43	0.96267934	0.264	0.128	6.33E-39	10
Ypel5	1.40E-39	0.64996828	0.25	0.117	4.51E-35	10
Ghitm	1.66E-35	0.53675474	0.27	0.135	5.34E-31	10
Iscu2	8.45E-35	0.57848404	0.258	0.129	2.73E-30	10
Pkn2	9.56E-35	0.78306783	0.266	0.137	3.09E-30	10
Riok3	3.16E-27	0.61098524	0.261	0.145	1.02E-22	10
Oaz14	5.18E-27	0.3359839	0.785	0.636	1.67E-22	10
Pim14	1.96E-21	0.28750396	0.48	0.336	6.34E-17	10
Hipk11	6.61E-19	0.77066034	0.279	0.185	2.13E-14	10
Ypel33	1.76E-18	0.434203	0.346	0.228	5.69E-14	10
Cyb5a1	9.87E-17	0.67445353	0.25	0.163	3.19E-12	10
Cd472	6.05E-16	0.73488159	0.478	0.401	1.95E-11	10
Gda3	3.45E-13	0.31734649	0.302	0.207	1.11E-08	10
Serinc33	5.96E-12	0.44221168	0.455	0.359	1.92E-07	10
Wnk11	4.93E-11	0.51343623	0.273	0.2	1.59E-06	10
Mpc24	1.41E-08	0.37800909	0.32	0.254	0.00045419	10
Akap134	4.65E-05	0.41847076	0.286	0.248	1	10
Vpreb1	0	5.04370651	0.952	0.016	0	11
Igll1	0	4.69877695	0.925	0.014	0	11
Dntt	0	3.12280233	0.613	0.005	0	11

Vpreb33	0	3.1098087	0.991	0.167	0	11
Mzb13	0	2.9225918	0.754	0.076	0	11
Lef1	0	2.79265879	0.555	0.016	0	11
Vpreb2	0	2.69533815	0.464	0.003	0	11
Ebf13	0	2.64726927	0.994	0.195	0	11
Tspan131	0	2.50569873	0.647	0.081	0	11
Cplx21	0	2.45809246	0.51	0.03	0	11
Crip13	0	2.41118143	0.933	0.384	0	11
Pmf11	0	2.38976765	0.502	0.079	0	11
Bfsp2	0	2.28059208	0.421	0.015	0	11
Myl10	0	2.22101387	0.277	0.004	0	11
Id31	0	2.18606671	0.606	0.072	0	11
Hes11	0	2.14180841	0.497	0.064	0	11
Cmtm73	0	2.13809676	0.751	0.223	0	11
Chchd102	0	2.04299643	0.727	0.132	0	11
Cd79a3	0	2.00390439	0.921	0.177	0	11
Gm30948	0	1.95909354	0.315	0.001	0	11
Blnk2	0	1.9171587	0.509	0.063	0	11
Impdh1	0	1.73021551	0.323	0.027	0	11
Rag1	0	1.71602619	0.304	0.024	0	11
Gimap6	0	1.71428973	0.339	0.031	0	11
Ptprcap3	0	1.68558115	0.697	0.159	0	11
Tifa2	0	1.68045294	0.495	0.092	0	11
Adgrg1	0	1.48925728	0.288	0.022	0	11
1700027J07F	0	1.47220488	0.255	0.011	0	11
Smarca42	1.41E-305	1.89739498	0.567	0.128	4.56E-301	11
Pde2a	6.78E-305	1.94024834	0.443	0.082	2.19E-300	11
Slamf71	8.97E-305	1.50333573	0.284	0.031	2.89E-300	11
Myb2	1.71E-289	1.74592821	0.494	0.102	5.53E-285	11
AU020206	1.95E-282	1.45192184	0.298	0.037	6.29E-278	11
Gmfg6	9.89E-278	1.88651728	0.836	0.391	3.19E-273	11
Ucp25	2.05E-277	1.89623272	0.798	0.336	6.60E-273	11
Akap122	4.44E-276	1.48093487	0.394	0.064	1.43E-271	11
Cd191	7.75E-249	1.25598687	0.307	0.043	2.50E-244	11
Rps194	2.24E-244	1.21363266	0.985	0.725	7.24E-240	11
Rasgrp22	1.75E-242	1.80586023	0.514	0.137	5.65E-238	11
Ptpn182	1.87E-241	1.74076933	0.726	0.281	6.02E-237	11
Rps245	5.39E-241	1.07515149	0.999	0.852	1.74E-236	11
Cd79b3	2.93E-240	1.13700602	0.611	0.155	9.47E-236	11
Xrcc61	2.85E-223	1.55110827	0.355	0.065	9.19E-219	11
Uba524	3.38E-222	1.17631578	0.976	0.752	1.09E-217	11
Rpl395	9.90E-222	1.0565104	0.995	0.842	3.20E-217	11
B3gnt21	5.27E-218	1.6483202	0.458	0.113	1.70E-213	11

Ighm3	3.03E-207	0.81733681	0.707	0.217	9.78E-203	11
Mef2c3	1.19E-206	1.4533755	0.524	0.144	3.85E-202	11
Rps115	4.34E-201	1.13042741	0.975	0.724	1.40E-196	11
Bcl7a2	8.55E-195	1.34943053	0.372	0.078	2.76E-190	11
Rabgap1l	2.03E-193	1.46600121	0.371	0.081	6.56E-189	11
Rpl94	6.93E-193	1.05594287	0.969	0.769	2.24E-188	11
Ptma3	1.55E-192	1.18329911	0.977	0.683	5.02E-188	11
Stambpl11	3.87E-181	1.26142377	0.292	0.053	1.25E-176	11
Rps15a4	7.78E-181	0.95430187	0.992	0.781	2.51E-176	11
Rps134	2.80E-178	0.98686186	0.973	0.792	9.03E-174	11
Rpl18a5	4.03E-174	0.94673495	0.992	0.802	1.30E-169	11
Pgls2	7.27E-169	1.37706479	0.719	0.334	2.35E-164	11
Tbc1d10c	7.51E-167	1.24043835	0.256	0.046	2.43E-162	11
Selenom	1.09E-166	1.70405544	0.255	0.046	3.53E-162	11
Rpl114	1.89E-165	0.96764161	0.968	0.735	6.09E-161	11
Sec632	9.93E-163	1.45567764	0.433	0.121	3.21E-158	11
Il7r2	2.23E-162	1.09026052	0.319	0.066	7.19E-158	11
Rps273	2.90E-159	0.76029075	0.999	0.91	9.35E-155	11
Rpl365	3.04E-156	0.89512442	0.992	0.756	9.81E-152	11
Ifi2032	2.28E-151	1.31339521	0.417	0.118	7.37E-147	11
Foxn31	2.51E-150	1.20680841	0.355	0.088	8.11E-146	11
Ndufb1-ps4	7.05E-149	1.11777496	0.886	0.631	2.28E-144	11
Rpl275	1.35E-148	0.93315028	0.947	0.719	4.37E-144	11
Rpl294	1.41E-145	1.06891415	0.88	0.567	4.56E-141	11
Fam53b1	9.37E-143	1.22366093	0.291	0.064	3.03E-138	11
Zfp7063	8.11E-140	1.19167864	0.644	0.274	2.62E-135	11
Naca4	2.74E-139	1.05964683	0.863	0.566	8.86E-135	11
Arl5c2	2.61E-136	1.06624294	0.337	0.084	8.42E-132	11
Tcf4	6.33E-136	1.20097114	0.363	0.098	2.04E-131	11
Rps295	2.36E-133	0.6717007	1	0.953	7.63E-129	11
Lgals91	4.14E-133	1.26658447	0.418	0.131	1.34E-128	11
Rpl35a5	8.80E-133	0.75075184	0.993	0.853	2.84E-128	11
H2afv2	3.29E-132	1.21635837	0.619	0.276	1.06E-127	11
Rps285	1.28E-130	0.7422347	0.998	0.842	4.12E-126	11
H3f3a4	1.67E-130	0.8366945	0.946	0.756	5.40E-126	11
Rpl185	2.38E-130	0.86587264	0.954	0.708	7.69E-126	11
Tcf32	1.12E-126	1.10356058	0.352	0.097	3.62E-122	11
Lrmp2	1.65E-126	1.06976571	0.342	0.093	5.31E-122	11
Tmsb104	2.03E-126	0.62798496	0.991	0.741	6.54E-122	11
Hnrnpul11	1.49E-125	1.40674725	0.447	0.161	4.82E-121	11
Ptp4a31	1.04E-123	1.24330592	0.349	0.098	3.37E-119	11
Rpl37a5	2.75E-123	0.70093978	0.998	0.902	8.88E-119	11
4930597A21	1.67E-122	1.23465374	0.266	0.06	5.38E-118	11

Rpl154	2.31E-120	0.87803381	0.923	0.655	7.45E-116	11
Pdcd42	9.87E-120	1.17622938	0.568	0.233	3.19E-115	11
Itga41	2.57E-119	1.28173002	0.329	0.093	8.30E-115	11
Srp142	4.04E-119	1.27465461	0.598	0.277	1.30E-114	11
Ezh21	4.54E-119	1.12109217	0.364	0.109	1.47E-114	11
Rpl194	6.57E-119	0.75909216	0.968	0.756	2.12E-114	11
Ndufa42	2.09E-117	1.19836488	0.738	0.458	6.75E-113	11
Rpl27a4	2.99E-117	0.74824148	0.977	0.796	9.64E-113	11
Rpl226	1.56E-116	0.8349393	0.931	0.66	5.03E-112	11
Rps265	6.33E-116	0.77040589	0.968	0.783	2.05E-111	11
Rps204	1.55E-114	0.75520289	0.977	0.738	4.99E-110	11
Elk4	1.77E-114	1.2194404	0.267	0.066	5.71E-110	11
Pafah1b32	8.56E-114	0.99749758	0.48	0.17	2.76E-109	11
Dck1	1.70E-113	1.11740955	0.307	0.084	5.50E-109	11
Jund3	1.41E-112	1.00241487	0.836	0.505	4.56E-108	11
Rps27a4	1.52E-112	0.6442935	0.978	0.891	4.90E-108	11
Pkig3	2.53E-111	1.09665857	0.403	0.132	8.16E-107	11
Oaz15	2.44E-110	0.91862614	0.841	0.635	7.86E-106	11
Serinc34	1.52E-109	1.15219947	0.677	0.351	4.91E-105	11
Rps75	2.03E-109	0.76480351	0.948	0.725	6.55E-105	11
Rpl214	3.00E-108	0.77655299	0.947	0.714	9.70E-104	11
Snrpe4	5.24E-108	1.04985664	0.729	0.421	1.69E-103	11
Xist2	5.89E-108	1.2460008	0.382	0.125	1.90E-103	11
Rcsd1	1.05E-107	1.30546203	0.314	0.093	3.40E-103	11
H2afy3	2.21E-107	1.16407971	0.539	0.233	7.14E-103	11
Rps185	4.79E-107	0.79744797	0.923	0.643	1.55E-102	11
Rpl305	2.94E-106	0.68801172	0.975	0.814	9.48E-102	11
Cd373	4.25E-106	1.14612539	0.47	0.184	1.37E-101	11
Rpl345	5.69E-104	0.68597475	0.986	0.822	1.84E-99	11
Inpp5d1	3.19E-103	1.12273697	0.308	0.091	1.03E-98	11
Rpl325	1.65E-99	0.71146162	0.973	0.725	5.31E-95	11
Snu132	1.02E-97	1.10402318	0.554	0.264	3.30E-93	11
Dusp22	1.66E-97	0.96595028	0.304	0.088	5.35E-93	11
Cox7a2l1	1.88E-97	1.22418753	0.493	0.209	6.07E-93	11
Rps166	2.09E-97	0.70195384	0.956	0.774	6.76E-93	11
Arpc5l2	2.10E-97	1.02648215	0.423	0.158	6.77E-93	11
Rpl285	6.91E-97	0.70518955	0.947	0.755	2.23E-92	11
Elof12	7.53E-97	1.03727443	0.403	0.147	2.43E-92	11
Tsc22d4	1.34E-95	1.30652119	0.585	0.309	4.32E-91	11
Rps35	1.05E-94	0.73806284	0.915	0.703	3.40E-90	11
Mndal2	1.02E-93	1.04866856	0.341	0.112	3.29E-89	11
Hmgb13	1.88E-92	1.15205568	0.784	0.532	6.06E-88	11
Uqcrh4	7.38E-92	0.97862359	0.769	0.507	2.38E-87	11

Slc38a23	1.25E-91	1.09569971	0.661	0.364	4.04E-87	11
Ets12	4.12E-90	1.10013887	0.393	0.147	1.33E-85	11
Rtraf2	2.10E-88	1.03189266	0.485	0.209	6.79E-84	11
Frg11	8.07E-88	1.1787967	0.394	0.154	2.61E-83	11
Cd691	1.29E-87	1.09274427	0.274	0.081	4.17E-83	11
Snrpg4	1.83E-87	0.93195169	0.754	0.501	5.90E-83	11
Rps4x5	8.56E-87	0.67645319	0.942	0.688	2.76E-82	11
Tomm72	4.81E-86	0.92957007	0.726	0.477	1.55E-81	11
Erh3	1.74E-85	1.10560895	0.478	0.222	5.62E-81	11
Rpl415	5.65E-85	0.59945879	0.99	0.9	1.82E-80	11
Gm100765	7.64E-85	0.64665724	0.965	0.806	2.47E-80	11
Ndufs52	1.56E-83	1.0609214	0.539	0.279	5.04E-79	11
Chchd23	2.22E-83	0.86864151	0.777	0.577	7.17E-79	11
Nsa24	3.99E-83	0.93151759	0.763	0.475	1.29E-78	11
AW112010	2.91E-81	0.63935133	0.261	0.077	9.39E-77	11
Cbx11	7.24E-81	0.98394912	0.419	0.172	2.34E-76	11
Jun	6.70E-80	1.09832261	0.413	0.172	2.16E-75	11
Clic12	1.11E-78	0.93293645	0.704	0.467	3.59E-74	11
Cox8a3	1.57E-78	0.70068487	0.844	0.682	5.08E-74	11
Atp5g24	2.08E-78	0.90401316	0.674	0.409	6.71E-74	11
Rpl244	3.76E-78	0.62332985	0.953	0.761	1.21E-73	11
Clta5	9.39E-78	1.01237911	0.581	0.313	3.03E-73	11
Rpl235	1.59E-77	0.56370761	0.988	0.821	5.12E-73	11
Atp5mpl3	1.03E-75	0.89967781	0.629	0.369	3.31E-71	11
Fau4	1.44E-75	0.4241828	0.995	0.941	4.66E-71	11
Cbx31	3.85E-75	0.96697191	0.591	0.334	1.24E-70	11
Uqcrb3	5.79E-75	0.96576556	0.567	0.317	1.87E-70	11
Mob41	7.78E-75	1.13559691	0.333	0.128	2.51E-70	11
Rpl36al5	3.94E-74	0.76213904	0.781	0.505	1.27E-69	11
Psmb83	1.19E-72	0.91929853	0.455	0.21	3.83E-68	11
Sox43	1.24E-72	0.89436731	0.387	0.152	3.99E-68	11
Rps105	1.50E-72	0.56950598	0.963	0.8	4.85E-68	11
Rpl355	2.08E-72	0.57412979	0.984	0.769	6.73E-68	11
Rpl10a5	1.18E-71	0.66677478	0.874	0.599	3.82E-67	11
Pan3	1.77E-70	1.11033769	0.323	0.125	5.71E-66	11
Btf35	4.03E-69	0.75999335	0.779	0.543	1.30E-64	11
Pfdn54	5.40E-69	0.91349871	0.7	0.454	1.74E-64	11
Pde4b	6.29E-69	1.09902673	0.274	0.096	2.03E-64	11
Rps55	1.33E-68	0.58618207	0.913	0.678	4.28E-64	11
Rps95	3.10E-68	0.50948572	0.956	0.874	9.99E-64	11
Atp1b12	4.45E-68	0.61911455	0.253	0.079	1.44E-63	11
Tut41	6.83E-68	0.97550922	0.296	0.108	2.20E-63	11
Snx21	8.73E-68	0.77386213	0.253	0.082	2.82E-63	11

Rpl385	2.50E-67	0.52449037	0.99	0.839	8.08E-63	11
Gstp12	1.82E-65	0.94831198	0.339	0.141	5.87E-61	11
Mgst22	1.56E-64	1.18062578	0.315	0.129	5.04E-60	11
Rps3a14	1.68E-64	0.53185753	0.964	0.77	5.43E-60	11
Arl6ip12	1.04E-63	0.9459709	0.425	0.204	3.37E-59	11
Uqcr102	1.37E-61	0.89885171	0.609	0.386	4.42E-57	11
Ypel34	1.49E-61	1.08437459	0.446	0.225	4.82E-57	11
Sumo11	1.06E-60	0.96318332	0.433	0.217	3.43E-56	11
Ly6e5	1.36E-60	0.67909266	0.691	0.398	4.39E-56	11
Snrpf3	1.41E-60	0.76519905	0.628	0.383	4.56E-56	11
Sub14	9.02E-60	0.77395686	0.695	0.475	2.91E-55	11
Atp1b31	1.84E-58	0.87332112	0.322	0.133	5.93E-54	11
Atp5e5	3.20E-58	0.61644157	0.837	0.695	1.03E-53	11
Paip22	1.18E-57	0.87376866	0.482	0.254	3.81E-53	11
Top2b1	2.96E-57	0.88165712	0.306	0.124	9.55E-53	11
Rpgrip11	4.41E-57	0.85939122	0.292	0.118	1.42E-52	11
Rpl74	1.79E-56	0.59388622	0.843	0.629	5.77E-52	11
Akap135	3.18E-56	0.9293813	0.46	0.241	1.03E-51	11
Zfp36l11	3.88E-56	0.72622564	0.313	0.124	1.25E-51	11
Ppp1r14b1	6.13E-56	0.66873432	0.508	0.254	1.98E-51	11
Elf11	1.38E-55	0.89714858	0.318	0.134	4.47E-51	11
Timm132	2.94E-53	0.8189107	0.438	0.23	9.50E-49	11
Atp5j23	7.17E-53	0.72327371	0.71	0.538	2.31E-48	11
Sumo22	9.93E-53	0.83108128	0.558	0.345	3.21E-48	11
Mrpl542	1.32E-52	0.82039181	0.318	0.137	4.26E-48	11
Ndufb52	1.34E-52	0.84261587	0.433	0.225	4.32E-48	11
mt-Nd4l3	1.92E-52	0.53659435	0.703	0.443	6.20E-48	11
H2-K13	2.02E-52	0.66266795	0.7	0.453	6.52E-48	11
Ndufb22	3.24E-52	0.86756227	0.425	0.22	1.05E-47	11
AI6622703	1.03E-51	0.84133595	0.309	0.135	3.31E-47	11
Cox7a23	1.65E-51	0.68981743	0.692	0.501	5.33E-47	11
Ppia3	6.60E-51	0.70413076	0.868	0.707	2.13E-46	11
Calm22	2.23E-50	0.68724532	0.694	0.482	7.19E-46	11
Cox6c4	5.85E-50	0.60767744	0.779	0.636	1.89E-45	11
Gng101	1.34E-48	0.93084562	0.29	0.127	4.32E-44	11
Polr2i2	4.06E-48	0.82595969	0.308	0.138	1.31E-43	11
Tra2b3	7.84E-48	0.85067288	0.46	0.255	2.53E-43	11
Rpl7a4	1.05E-47	0.58147367	0.763	0.555	3.39E-43	11
Sf3b62	1.58E-47	0.75776607	0.462	0.26	5.10E-43	11
Rpl105	3.17E-47	0.58343665	0.788	0.6	1.02E-42	11
mt-Co14	4.89E-47	0.36565957	0.976	0.881	1.58E-42	11
Cox7c5	8.52E-47	0.51382177	0.835	0.668	2.75E-42	11
Ubb5	7.87E-46	0.45968133	0.889	0.757	2.54E-41	11

Cox5b3	8.39E-46	0.69374164	0.648	0.47	2.71E-41	11
Lsm71	1.11E-45	0.71966322	0.322	0.148	3.57E-41	11
Atp5g32	1.40E-45	0.75422572	0.468	0.266	4.53E-41	11
Cdkn1b1	1.76E-45	1.05715907	0.321	0.156	5.69E-41	11
Krtcap22	2.56E-45	0.70438328	0.462	0.25	8.28E-41	11
Rps233	3.09E-44	0.41379043	0.949	0.758	9.97E-40	11
Eif3f4	3.38E-44	0.74481013	0.495	0.285	1.09E-39	11
Rpl263	3.83E-44	0.43421108	0.913	0.698	1.24E-39	11
Polr1d4	8.00E-44	0.73188321	0.499	0.296	2.58E-39	11
Nedd83	8.89E-44	0.72984276	0.547	0.351	2.87E-39	11
Sec61b2	1.35E-43	0.70519466	0.624	0.444	4.37E-39	11
Rpl22l14	4.34E-43	0.55535881	0.726	0.518	1.40E-38	11
Pold42	8.50E-43	0.77565033	0.305	0.142	2.74E-38	11
Hnrnpa03	1.15E-42	0.69923593	0.491	0.286	3.73E-38	11
Hnrnpm2	1.28E-42	0.78104127	0.49	0.293	4.12E-38	11
Hnrnpa13	1.83E-42	0.68997968	0.475	0.271	5.91E-38	11
Tgfb12	3.51E-42	0.8465931	0.323	0.156	1.13E-37	11
Atp5h2	9.84E-42	0.63963553	0.641	0.473	3.18E-37	11
Selenow3	2.24E-41	0.62780237	0.472	0.26	7.23E-37	11
Sh3bgrl2	7.61E-41	0.73290846	0.351	0.179	2.46E-36	11
Rplp24	8.29E-41	0.45669372	0.891	0.712	2.68E-36	11
Cxcr43	9.38E-41	0.49966058	0.417	0.217	3.03E-36	11
Atp5k4	2.55E-40	0.6164274	0.669	0.506	8.22E-36	11
Mtdh	3.70E-40	0.78177773	0.336	0.169	1.19E-35	11
Psma23	3.98E-40	0.69141869	0.5	0.314	1.28E-35	11
Prrc2c1	4.82E-40	0.7783175	0.426	0.239	1.56E-35	11
Foxp13	5.81E-40	0.5719633	0.413	0.218	1.88E-35	11
Park71	1.17E-39	0.63085705	0.417	0.227	3.76E-35	11
Rpl36a4	2.65E-39	0.43688205	0.821	0.578	8.57E-35	11
Rnf71	4.27E-39	0.66441988	0.311	0.152	1.38E-34	11
Herpud12	6.01E-39	0.52276359	0.323	0.153	1.94E-34	11
Emg11	6.55E-39	0.68688521	0.308	0.149	2.11E-34	11
Mrps142	9.35E-39	0.69867711	0.357	0.188	3.02E-34	11
Ndufs82	1.15E-38	0.73347047	0.334	0.17	3.73E-34	11
Scaf111	2.41E-38	0.74929736	0.368	0.196	7.79E-34	11
Psmb14	2.74E-38	0.66314126	0.493	0.299	8.84E-34	11
Ubl54	2.87E-38	0.64822479	0.669	0.518	9.27E-34	11
Grcc102	3.16E-38	0.71538449	0.447	0.266	1.02E-33	11
Rplp03	4.26E-38	0.39972743	0.911	0.735	1.38E-33	11
Eif3h4	5.31E-38	0.67340584	0.464	0.272	1.71E-33	11
Ndufa121	2.96E-37	0.56062778	0.311	0.151	9.56E-33	11
Srp92	3.07E-37	0.72057921	0.436	0.259	9.90E-33	11
Dcun1d51	4.11E-37	0.70705088	0.352	0.187	1.33E-32	11

Eif3e2	4.22E-37	0.62681857	0.404	0.222	1.36E-32	11
Nop104	7.56E-37	0.65399356	0.436	0.248	2.44E-32	11
Gm118082	8.95E-37	0.71161451	0.327	0.169	2.89E-32	11
Rpl133	1.31E-36	0.36829068	0.971	0.788	4.22E-32	11
Znrd11	7.62E-36	0.64829787	0.252	0.114	2.46E-31	11
Sdc41	1.53E-35	0.54588352	0.311	0.146	4.94E-31	11
Tcea11	3.09E-35	0.78150557	0.33	0.174	9.99E-31	11
Sarnp2	3.28E-35	0.67871019	0.415	0.244	1.06E-30	11
Cyfip22	3.61E-35	0.86898191	0.289	0.146	1.17E-30	11
Mdh11	5.66E-35	0.64783918	0.278	0.135	1.83E-30	11
Micos103	6.22E-35	0.65009727	0.442	0.267	2.01E-30	11
mt-Atp83	1.60E-34	0.60735862	0.458	0.273	5.15E-30	11
Ssna11	2.17E-34	0.69592288	0.27	0.132	7.02E-30	11
Raly1	8.42E-34	0.7376875	0.372	0.212	2.72E-29	11
Thoc71	1.05E-33	0.65850292	0.359	0.195	3.39E-29	11
Phf5a1	2.77E-33	0.6546885	0.282	0.14	8.95E-29	11
Mbnl12	3.35E-33	0.65746492	0.501	0.325	1.08E-28	11
Cox7b4	3.51E-33	0.60400383	0.515	0.341	1.13E-28	11
Snhg11	3.99E-33	0.55430087	0.376	0.206	1.29E-28	11
Ifi27	1.24E-32	0.78510629	0.277	0.139	3.99E-28	11
Tmem2584	1.34E-32	0.60391513	0.559	0.382	4.32E-28	11
2410006H16I	1.51E-32	0.49898459	0.598	0.395	4.87E-28	11
Calm31	1.70E-32	0.71951165	0.411	0.253	5.48E-28	11
Mrpl341	3.80E-32	0.61871925	0.314	0.166	1.23E-27	11
Atp5j3	5.99E-32	0.65410643	0.559	0.412	1.93E-27	11
Smdt14	1.10E-31	0.60189962	0.508	0.336	3.54E-27	11
Mrpl302	1.69E-31	0.70413802	0.322	0.175	5.45E-27	11
Supt4a4	2.49E-31	0.75255889	0.436	0.28	8.05E-27	11
Psmb61	4.14E-31	0.5943039	0.389	0.229	1.34E-26	11
Ndufv22	5.52E-31	0.61129406	0.311	0.165	1.78E-26	11
Rps213	5.61E-31	0.50293426	0.788	0.628	1.81E-26	11
Ndufb113	5.74E-31	0.64689436	0.484	0.321	1.85E-26	11
Eif3k3	7.28E-31	0.63217354	0.479	0.307	2.35E-26	11
Tmem2562	8.40E-31	0.64078442	0.433	0.272	2.71E-26	11
Birc61	9.36E-31	0.63798709	0.3	0.158	3.02E-26	11
Tma72	2.01E-30	0.59074022	0.552	0.385	6.49E-26	11
Brd2	3.55E-30	0.61818734	0.273	0.137	1.15E-25	11
Tle41	4.01E-30	0.72124989	0.256	0.129	1.29E-25	11
Rpl313	4.49E-30	0.39223502	0.76	0.555	1.45E-25	11
Psmb92	4.88E-30	0.63668446	0.299	0.159	1.57E-25	11
Ndufc22	4.93E-30	0.6261912	0.379	0.225	1.59E-25	11
Llph2	6.14E-30	0.66310378	0.346	0.195	1.98E-25	11
Ralbp1	7.03E-30	0.82214491	0.301	0.166	2.27E-25	11

Snrpb4	9.83E-30	0.59890077	0.441	0.275	3.17E-25	11
Marcks4	1.02E-29	0.3974508	0.365	0.194	3.29E-25	11
Snrpd22	1.44E-29	0.57920172	0.464	0.298	4.65E-25	11
Mrpl231	1.81E-29	0.57465024	0.283	0.145	5.85E-25	11
Rpl374	1.83E-29	0.29367262	0.986	0.914	5.92E-25	11
Etfb1	2.02E-29	0.53569751	0.304	0.161	6.52E-25	11
Ndufa113	2.71E-29	0.64380287	0.416	0.258	8.76E-25	11
Psmb22	4.59E-29	0.62561639	0.375	0.224	1.48E-24	11
Rbbp41	4.89E-29	0.5514245	0.314	0.17	1.58E-24	11
Nol74	6.41E-29	0.58874826	0.433	0.268	2.07E-24	11
Cox5a3	6.75E-29	0.57869786	0.542	0.387	2.18E-24	11
Rbx13	7.00E-29	0.63078755	0.518	0.372	2.26E-24	11
Mrps332	1.67E-28	0.5472879	0.341	0.192	5.40E-24	11
Cox6b13	3.01E-28	0.52374203	0.64	0.495	9.73E-24	11
Selenoh1	4.72E-28	0.63374185	0.322	0.181	1.52E-23	11
Atpif12	5.55E-28	0.34845024	0.607	0.42	1.79E-23	11
Rp91	9.95E-28	0.67198306	0.298	0.164	3.21E-23	11
Ywhaq2	1.17E-27	0.50380372	0.353	0.202	3.79E-23	11
Anp32a1	1.18E-27	0.60648114	0.43	0.279	3.82E-23	11
Atp5l3	1.32E-27	0.45752755	0.716	0.608	4.26E-23	11
Psmb33	1.36E-27	0.60338323	0.442	0.294	4.39E-23	11
Ssr42	1.48E-27	0.5202469	0.411	0.248	4.78E-23	11
Serbp12	1.76E-27	0.4845332	0.673	0.483	5.67E-23	11
Anapc132	2.21E-27	0.67297969	0.338	0.198	7.13E-23	11
Ndufb33	1.00E-26	0.61734796	0.393	0.246	3.23E-22	11
Ndufc13	1.26E-26	0.60416831	0.477	0.323	4.05E-22	11
Ndufab11	1.77E-26	0.55826456	0.348	0.203	5.73E-22	11
Smim112	2.05E-26	0.68190573	0.26	0.137	6.63E-22	11
Skp1a2	3.79E-26	0.60228233	0.368	0.22	1.22E-21	11
B2m2	4.53E-26	0.32185514	0.861	0.707	1.46E-21	11
Cdk41	6.22E-26	0.44007684	0.27	0.14	2.01E-21	11
Mrps212	1.13E-25	0.59101772	0.465	0.319	3.63E-21	11
Ethe12	1.22E-25	0.69000378	0.254	0.137	3.95E-21	11
Ddx39b1	2.05E-25	0.54098801	0.315	0.178	6.63E-21	11
Hnrnpk3	3.11E-25	0.51262288	0.539	0.382	1.00E-20	11
Sdhb2	5.11E-25	0.5704381	0.304	0.173	1.65E-20	11
Wtap1	6.04E-25	0.59439112	0.263	0.142	1.95E-20	11
Atp6v1f4	6.74E-25	0.59530849	0.467	0.321	2.18E-20	11
Nmt12	1.11E-24	0.58768074	0.341	0.205	3.60E-20	11
Srsf32	1.45E-24	0.54167638	0.533	0.393	4.68E-20	11
Dut2	1.69E-24	0.70045437	0.327	0.196	5.47E-20	11
Dbi4	1.87E-24	0.4185366	0.472	0.307	6.03E-20	11
Cd24a3	1.87E-24	0.38099572	0.575	0.42	6.04E-20	11

Ndufb102	1.91E-24	0.59412607	0.381	0.241	6.15E-20	11
Gm472832	2.00E-24	0.4041713	0.299	0.163	6.45E-20	11
Snhg81	2.34E-24	0.44369537	0.378	0.225	7.55E-20	11
Celf22	5.32E-24	0.66704197	0.306	0.181	1.72E-19	11
1110038B12	5.36E-24	0.42610317	0.323	0.182	1.73E-19	11
Rtf11	7.03E-24	0.64252981	0.283	0.16	2.27E-19	11
Smarca52	7.34E-24	0.55726093	0.395	0.252	2.37E-19	11
Lsm81	1.87E-23	0.56297492	0.256	0.14	6.04E-19	11
Rbis1	1.91E-23	0.48519246	0.299	0.168	6.18E-19	11
Fkbp1a1	2.12E-23	0.31705888	0.353	0.201	6.84E-19	11
Abracl2	2.57E-23	0.60745736	0.412	0.277	8.29E-19	11
Ywhae2	6.65E-23	0.4369759	0.506	0.349	2.15E-18	11
Trir2	7.29E-23	0.60450463	0.406	0.269	2.35E-18	11
Wasf21	1.26E-22	0.58519731	0.269	0.151	4.08E-18	11
U2surp1	1.77E-22	0.44068153	0.269	0.148	5.71E-18	11
Ndufa24	1.78E-22	0.47158612	0.576	0.434	5.73E-18	11
Clint1	2.04E-22	0.69972385	0.292	0.175	6.57E-18	11
Cox141	2.80E-22	0.62103682	0.293	0.173	9.05E-18	11
Smim141	2.99E-22	0.64669794	0.322	0.198	9.65E-18	11
Elob3	9.39E-22	0.46706637	0.657	0.566	3.03E-17	11
Hint13	1.20E-21	0.45145134	0.55	0.405	3.87E-17	11
Ube2i2	1.22E-21	0.54345922	0.374	0.243	3.94E-17	11
Tceal91	1.43E-21	0.39018437	0.268	0.147	4.63E-17	11
Prpf4b3	2.02E-21	0.51832846	0.385	0.245	6.54E-17	11
Dpm32	2.17E-21	0.48037297	0.391	0.252	7.02E-17	11
Ythdc1	2.64E-21	0.55070261	0.288	0.168	8.52E-17	11
Atp5md3	2.75E-21	0.3860835	0.649	0.538	8.87E-17	11
Ndufs42	2.89E-21	0.56951751	0.299	0.181	9.35E-17	11
Uqcrfs12	5.89E-21	0.54375977	0.288	0.17	1.90E-16	11
Cd81	6.41E-21	0.41654619	0.292	0.169	2.07E-16	11
Pfdn21	8.56E-21	0.54640043	0.277	0.161	2.76E-16	11
Ndufa82	1.03E-20	0.58279909	0.29	0.175	3.31E-16	11
Ndufa16	1.15E-20	0.53554375	0.486	0.356	3.72E-16	11
Psma52	1.49E-20	0.41730835	0.303	0.178	4.80E-16	11
Anp32e2	1.95E-20	0.47325561	0.326	0.199	6.29E-16	11
Sin3b	1.96E-20	0.5672815	0.304	0.186	6.33E-16	11
Sec61g3	2.01E-20	0.29069064	0.732	0.61	6.48E-16	11
Ndufs64	2.67E-20	0.51707719	0.383	0.256	8.61E-16	11
Snrpd12	2.86E-20	0.48564682	0.39	0.257	9.25E-16	11
Naa382	3.01E-20	0.42685302	0.277	0.159	9.73E-16	11
Arpc34	3.49E-20	0.43231586	0.648	0.516	1.13E-15	11
Siva12	3.67E-20	0.50468798	0.27	0.158	1.18E-15	11
Ppp1ca4	4.27E-20	0.56308956	0.488	0.367	1.38E-15	11

Uqcrq2	5.35E-20	0.44895885	0.536	0.408	1.73E-15	11
Supt161	5.88E-20	0.43960912	0.285	0.166	1.90E-15	11
Gng53	6.49E-20	0.41161796	0.769	0.651	2.09E-15	11
Chchd12	7.27E-20	0.45036405	0.305	0.184	2.35E-15	11
Drap1	7.53E-20	0.52000279	0.315	0.193	2.43E-15	11
Rps27l3	9.75E-20	0.49019859	0.482	0.345	3.15E-15	11
Stag21	1.08E-19	0.54810728	0.271	0.161	3.50E-15	11
Psme14	1.78E-19	0.44598645	0.442	0.306	5.74E-15	11
Srsf92	2.04E-19	0.53588355	0.305	0.189	6.58E-15	11
Grk23	2.16E-19	0.63933733	0.398	0.271	6.98E-15	11
Rbm34	2.68E-19	0.46786664	0.542	0.427	8.66E-15	11
Ndufa51	2.96E-19	0.44611195	0.328	0.204	9.55E-15	11
Arglu11	3.67E-19	0.51941951	0.319	0.199	1.18E-14	11
Bax1	3.82E-19	0.52119022	0.258	0.149	1.23E-14	11
Sap182	4.00E-19	0.53979742	0.35	0.23	1.29E-14	11
Tbca1	4.76E-19	0.45505929	0.425	0.285	1.54E-14	11
Tomm63	4.89E-19	0.55513201	0.525	0.418	1.58E-14	11
Arpp192	6.76E-19	0.5404844	0.457	0.34	2.18E-14	11
Lsm52	7.27E-19	0.53991181	0.345	0.225	2.35E-14	11
Uqcr113	7.88E-19	0.48281637	0.491	0.358	2.55E-14	11
Cwc152	8.05E-19	0.51756579	0.296	0.182	2.60E-14	11
Dtymk1	1.33E-18	0.37854668	0.258	0.148	4.29E-14	11
Psma61	1.53E-18	0.42536879	0.27	0.16	4.93E-14	11
Cuta2	1.55E-18	0.46364986	0.283	0.171	5.01E-14	11
Tomm222	1.63E-18	0.51483831	0.337	0.219	5.25E-14	11
Pten1	1.68E-18	0.5626474	0.308	0.192	5.42E-14	11
Mrpl524	2.41E-18	0.33910896	0.455	0.3	7.77E-14	11
Son2	2.72E-18	0.46623622	0.512	0.384	8.77E-14	11
Hnrnpc2	3.35E-18	0.48225024	0.345	0.223	1.08E-13	11
Polr2j2	4.16E-18	0.52265586	0.286	0.178	1.34E-13	11
Bloc1s12	6.03E-18	0.60242749	0.306	0.198	1.95E-13	11
Atp5f13	9.33E-18	0.45936479	0.44	0.319	3.01E-13	11
Lsm31	1.36E-17	0.41184442	0.26	0.154	4.39E-13	11
Lsm41	1.47E-17	0.42232853	0.345	0.224	4.76E-13	11
1110004F10I	1.54E-17	0.4250023	0.297	0.182	4.98E-13	11
Tagln22	1.82E-17	0.38681174	0.45	0.305	5.89E-13	11
Ewsr11	1.85E-17	0.49128176	0.268	0.161	5.98E-13	11
Prdx14	2.42E-17	0.30155595	0.545	0.392	7.80E-13	11
Csnk1a12	3.02E-17	0.46765643	0.363	0.241	9.77E-13	11
Sf3b52	3.34E-17	0.46120654	0.324	0.21	1.08E-12	11
Snrpd33	3.84E-17	0.46560061	0.385	0.266	1.24E-12	11
Pdcd52	5.17E-17	0.44755536	0.337	0.22	1.67E-12	11
Gtf2h51	5.53E-17	0.45901335	0.312	0.201	1.79E-12	11

Atp5o3	1.01E-16	0.45321644	0.364	0.244	3.26E-12	11
U2af11	1.09E-16	0.43921946	0.335	0.221	3.51E-12	11
Eif3i2	1.41E-16	0.36529393	0.281	0.17	4.55E-12	11
Nono1	1.42E-16	0.3801638	0.271	0.163	4.59E-12	11
Ndufb42	1.58E-16	0.38700463	0.355	0.238	5.11E-12	11
Psmc31	2.04E-16	0.51464554	0.29	0.184	6.60E-12	11
Ubald21	2.14E-16	0.44786314	0.304	0.194	6.92E-12	11
Atp5g12	3.18E-16	0.5136473	0.402	0.283	1.03E-11	11
Polr2k2	4.20E-16	0.42933453	0.36	0.244	1.36E-11	11
Ppp1cb2	4.21E-16	0.52844297	0.288	0.185	1.36E-11	11
Hnrnpa32	5.30E-16	0.39643524	0.492	0.371	1.71E-11	11
Top11	5.91E-16	0.49450215	0.475	0.363	1.91E-11	11
Mrps242	6.64E-16	0.44607155	0.274	0.17	2.14E-11	11
Ndufb73	7.82E-16	0.43895427	0.493	0.379	2.52E-11	11
Ubxn12	9.56E-16	0.45881485	0.345	0.234	3.09E-11	11
Ost42	1.03E-15	0.44824375	0.464	0.348	3.34E-11	11
Swi52	1.16E-15	0.38723901	0.363	0.245	3.76E-11	11
Hsbp11	1.36E-15	0.45375053	0.307	0.2	4.39E-11	11
Stmn11	1.42E-15	0.7104154	0.357	0.261	4.57E-11	11
Hnrnpf2	2.07E-15	0.41930221	0.434	0.319	6.69E-11	11
Atp5c13	2.88E-15	0.4448899	0.391	0.276	9.30E-11	11
Sfpq2	2.89E-15	0.3733483	0.344	0.229	9.33E-11	11
Tubb53	6.68E-15	0.41713629	0.483	0.359	2.16E-10	11
Tsc22d33	6.79E-15	0.37515204	0.279	0.176	2.19E-10	11
Mrps162	7.56E-15	0.33931301	0.259	0.16	2.44E-10	11
Ncl1	7.77E-15	0.28776721	0.565	0.413	2.51E-10	11
Psenen2	8.32E-15	0.4518237	0.36	0.255	2.69E-10	11
Psma34	9.50E-15	0.37363413	0.367	0.253	3.07E-10	11
Aimp12	1.00E-14	0.36191895	0.316	0.206	3.23E-10	11
Slc25a53	1.22E-14	0.38914609	0.383	0.268	3.93E-10	11
Psmd82	1.23E-14	0.48947953	0.293	0.193	3.98E-10	11
Ube2a2	1.96E-14	0.49685138	0.278	0.182	6.32E-10	11
Dnajc81	2.14E-14	0.44725906	0.27	0.172	6.92E-10	11
Tmed9	3.75E-14	0.35828305	0.255	0.159	1.21E-09	11
Nipbl1	3.97E-14	0.44040166	0.268	0.169	1.28E-09	11
Cdv31	4.03E-14	0.43771639	0.268	0.171	1.30E-09	11
Eny21	4.04E-14	0.29800686	0.291	0.185	1.30E-09	11
Alyref2	5.96E-14	0.43896395	0.327	0.224	1.92E-09	11
Edf13	6.16E-14	0.46178606	0.419	0.311	1.99E-09	11
Hnrnph12	7.00E-14	0.38769426	0.331	0.223	2.26E-09	11
St131	8.18E-14	0.30919655	0.361	0.243	2.64E-09	11
Srsf111	8.80E-14	0.37510394	0.263	0.167	2.84E-09	11
Lsm62	9.38E-14	0.35575459	0.328	0.223	3.03E-09	11

Srsf101	1.23E-13	0.3638275	0.251	0.156	3.96E-09	11
Ndufa32	1.65E-13	0.40423406	0.506	0.409	5.33E-09	11
Fkbp32	1.81E-13	0.29358677	0.264	0.166	5.85E-09	11
Abcf11	2.81E-13	0.40918257	0.252	0.158	9.07E-09	11
Ap2s12	3.80E-13	0.4899902	0.284	0.192	1.23E-08	11
Romo11	4.46E-13	0.33480723	0.465	0.348	1.44E-08	11
Ndufa72	4.71E-13	0.39932229	0.539	0.429	1.52E-08	11
Cggbp11	4.99E-13	0.43480059	0.286	0.193	1.61E-08	11
Pcna1	6.16E-13	0.42381243	0.283	0.188	1.99E-08	11
Ndufa62	6.23E-13	0.40828345	0.426	0.321	2.01E-08	11
Zfas11	6.77E-13	0.35641058	0.29	0.19	2.19E-08	11
Tomm202	6.93E-13	0.34204567	0.368	0.258	2.24E-08	11
Acin11	7.06E-13	0.38330421	0.254	0.162	2.28E-08	11
Atp5d3	7.84E-13	0.41953818	0.376	0.271	2.53E-08	11
Ssu721	1.09E-12	0.54149666	0.264	0.179	3.53E-08	11
Hist1h2ae1	3.36E-12	0.88027861	0.279	0.191	1.09E-07	11
Ndufa132	3.73E-12	0.36080461	0.524	0.428	1.21E-07	11
Cytip4	4.48E-12	0.36414175	0.273	0.18	1.45E-07	11
Micos13	4.85E-12	0.34294426	0.299	0.203	1.57E-07	11
Syf22	5.22E-12	0.53355963	0.288	0.201	1.68E-07	11
Psma41	5.47E-12	0.26034713	0.269	0.175	1.77E-07	11
Hnrnpdl1	6.20E-12	0.26543904	0.284	0.189	2.00E-07	11
Ndufb85	6.58E-12	0.35835612	0.395	0.294	2.12E-07	11
Psmb52	9.23E-12	0.34125701	0.32	0.227	2.98E-07	11
Hnrnpd1	1.09E-11	0.31726735	0.288	0.195	3.51E-07	11
Ddx53	1.21E-11	0.34295879	0.647	0.547	3.89E-07	11
Lamtor22	1.34E-11	0.37645943	0.365	0.268	4.31E-07	11
Fus3	1.54E-11	0.34619395	0.355	0.254	4.96E-07	11
Hnrnpl2	1.98E-11	0.37201261	0.319	0.225	6.41E-07	11
S100a132	2.97E-11	0.42316062	0.385	0.289	9.57E-07	11
Ssb2	5.21E-11	0.26397176	0.319	0.219	1.68E-06	11
Srsf22	6.14E-11	0.30887512	0.357	0.259	1.98E-06	11
Rbm393	6.36E-11	0.31002066	0.509	0.406	2.05E-06	11
Mrpl202	1.67E-10	0.27488351	0.285	0.198	5.39E-06	11
Srsf71	1.83E-10	0.27682825	0.313	0.222	5.90E-06	11
Hmgb23	1.98E-10	0.45542166	0.703	0.641	6.40E-06	11
Ppp1cc1	2.43E-10	0.26224254	0.26	0.176	7.84E-06	11
Mrfap12	2.68E-10	0.32254609	0.277	0.191	8.64E-06	11
Ptbp32	2.68E-10	0.37116872	0.365	0.274	8.66E-06	11
Srsf53	2.88E-10	0.40848718	0.471	0.383	9.30E-06	11
Wdr83os1	3.24E-10	0.31789045	0.251	0.17	1.05E-05	11
Atp5a13	3.84E-10	0.26562985	0.395	0.291	1.24E-05	11
Dek2	3.98E-10	0.3077424	0.404	0.308	1.28E-05	11

Mier11	6.00E-10	0.38210346	0.253	0.172	1.94E-05	11
Scand14	6.00E-10	0.41665072	0.442	0.355	1.94E-05	11
Dynll12	6.21E-10	0.30954032	0.543	0.452	2.01E-05	11
Snrnp272	7.70E-10	0.38194341	0.276	0.193	2.49E-05	11
Hist1h2ap1	1.63E-09	0.6366335	0.337	0.25	5.25E-05	11
Ndufs72	2.35E-09	0.32505829	0.261	0.184	7.58E-05	11
Sf3b13	3.01E-09	0.33653023	0.355	0.267	9.70E-05	11
Zcrb11	4.47E-09	0.43337	0.259	0.186	0.00014439	11
Rsrc21	8.30E-09	0.37149867	0.276	0.201	0.00026805	11
Limd23	9.21E-09	0.48507397	0.301	0.228	0.00029721	11
Taf102	1.01E-08	0.29271097	0.312	0.23	0.00032701	11
Bola23	1.26E-08	0.2530262	0.331	0.242	0.00040553	11
Trmt1123	1.28E-08	0.26528101	0.33	0.242	0.00041286	11
Cycs3	2.48E-08	0.32701731	0.32	0.242	0.00080117	11
Morf4l12	2.87E-08	0.29315072	0.454	0.368	0.00092612	11
Pet1002	3.32E-08	0.32609188	0.433	0.355	0.0010734	11
Luc7l21	5.19E-08	0.30156236	0.293	0.216	0.00167594	11
Npc22	5.55E-08	0.30500093	0.333	0.25	0.00179324	11
Arpc46	8.44E-08	0.38506202	0.334	0.266	0.00272458	11
Chd4	1.06E-07	0.25149101	0.275	0.2	0.00342461	11
Pcbp12	1.80E-07	0.2704706	0.327	0.249	0.00582535	11
Atf4	1.88E-07	0.42061836	0.3	0.234	0.00607065	11
Pnrc13	3.08E-07	0.30319384	0.393	0.317	0.00993989	11
Dynlrb12	4.63E-07	0.29363036	0.289	0.22	0.01493972	11
Dync1i23	6.50E-07	0.36838127	0.351	0.285	0.02097252	11
Dusp112	6.72E-07	0.36389165	0.254	0.191	0.02169709	11
Csnk2b2	7.12E-07	0.28512738	0.267	0.2	0.02297676	11
Zc3h152	1.21E-06	0.26781096	0.279	0.211	0.03902811	11
Ddx6	1.56E-06	0.31138391	0.293	0.226	0.0504125	11
Tuba1b2	2.08E-06	0.4031704	0.292	0.236	0.06711176	11
Prkar1a	2.65E-06	0.30056495	0.291	0.228	0.08569724	11
Higd2a1	4.65E-06	0.26399563	0.255	0.196	0.15021545	11
Fis13	5.36E-06	0.30603357	0.436	0.374	0.17289107	11
Hmgn22	5.80E-06	0.37257822	0.412	0.374	0.18717657	11
Clk14	6.48E-06	0.42789255	0.341	0.285	0.20927208	11
Eloc1	8.38E-06	0.27800708	0.308	0.248	0.27054354	11
Eif5a2	1.01E-05	0.29155518	0.463	0.409	0.3258307	11
Timm10b3	1.37E-05	0.2583511	0.326	0.261	0.4436143	11
Magoh4	2.30E-05	0.28000041	0.318	0.265	0.74271282	11
Pafah1b11	2.99E-05	0.2742927	0.318	0.261	0.96450999	11
Tra2a	3.90E-05	0.36712181	0.255	0.204	1	11
Dnajb6	0.00013505	0.34466411	0.288	0.238	1	11
Ube2l32	0.00071778	0.2685657	0.251	0.208	1	11

Ccdc121	0.00262936	0.26045306	0.254	0.218	1	11
Elane	0	5.80965285	0.879	0.014	0	12
Prtn3	0	5.76990542	0.955	0.036	0	12
Mpo	0	4.90740348	0.84	0.012	0	12
Ctsg	0	4.84262645	0.873	0.012	0	12
Ms4a31	0	3.12905651	0.696	0.023	0	12
Plac82	0	2.79399168	0.945	0.261	0	12
Nkg7	0	2.31879575	0.606	0.034	0	12
Ly6c24	0	2.26021558	0.798	0.213	0	12
Ramp11	0	2.08627703	0.798	0.097	0	12
Etfb2	0	1.92180869	0.827	0.147	0	12
Gstm11	0	1.69074896	0.652	0.043	0	12
Ssr43	0	1.67457845	0.844	0.236	0	12
Clec12a3	0	1.63493077	0.709	0.124	0	12
Cmtm74	0	1.47644783	0.844	0.224	0	12
Alyref3	0	1.42172463	0.852	0.21	0	12
Rgcc2	0	1.40896916	0.659	0.115	0	12
Prss57	0	1.39006239	0.441	0.008	0	12
Tmed31	0	1.35284623	0.665	0.087	0	12
Ap3s11	0	1.30736045	0.723	0.143	0	12
C1qbp1	0	1.30673962	0.689	0.132	0	12
Sdf2l11	0	1.29715841	0.617	0.053	0	12
Tuba4a1	0	1.23197019	0.662	0.116	0	12
Siva13	0	1.22523731	0.742	0.145	0	12
Cenpx2	0	1.2174131	0.725	0.12	0	12
Ms4a6c2	0	1.1537089	0.486	0.062	0	12
Gatm	0	1.12008312	0.472	0.027	0	12
F13a11	0	1.09665733	0.447	0.044	0	12
Ssr2	0	1.08026641	0.677	0.125	0	12
Med211	0	1.06148072	0.635	0.116	0	12
Ddost1	0	1.03920455	0.659	0.104	0	12
Alas1	0	1.01943069	0.47	0.043	0	12
Dtymk2	0	0.99351292	0.716	0.135	0	12
AI6622704	0	0.98614418	0.683	0.125	0	12
Srm	0	0.97001148	0.46	0.051	0	12
Dctpp11	0	0.96755219	0.604	0.101	0	12
Ass1	0	0.96547576	0.393	0.023	0	12
Cenpw1	0	0.95404584	0.651	0.1	0	12
Mrpl121	0	0.95066478	0.592	0.09	0	12
Nhp21	0	0.93275947	0.674	0.125	0	12
Tyms1	0	0.8990514	0.504	0.071	0	12
Grpel12	0	0.89751856	0.672	0.115	0	12
Tipin1	0	0.89157505	0.555	0.08	0	12

Dmkn	0	0.87307504	0.342	0.003	0	12
Fmc1	0	0.86530005	0.639	0.099	0	12
Tnfsf131	0	0.85580721	0.421	0.024	0	12
Cst7	0	0.84972064	0.357	0.005	0	12
Mydgf	0	0.77517488	0.571	0.085	0	12
Gmnn1	0	0.77257531	0.617	0.096	0	12
Hat1	0	0.76964953	0.496	0.06	0	12
1700020L24F	0	0.76656809	0.393	0.031	0	12
Exosc7	0	0.75302721	0.475	0.055	0	12
Timm8a1	0	0.74960359	0.438	0.048	0	12
Polr2l2	0	0.74542017	0.656	0.118	0	12
Syce2	0	0.74486029	0.473	0.048	0	12
Mcee1	0	0.73381738	0.523	0.073	0	12
Gm1604a	0	0.72251368	0.377	0.031	0	12
BC035044	0	0.70630537	0.378	0.032	0	12
Phb	0	0.70602467	0.483	0.061	0	12
Fcor	0	0.69515295	0.352	0.015	0	12
Svip	0	0.69245508	0.502	0.067	0	12
Mrpl36	0	0.68813743	0.597	0.096	0	12
Mrps281	0	0.67453788	0.556	0.073	0	12
Psmg1	0	0.6486053	0.387	0.035	0	12
Tmem147	0	0.64471783	0.533	0.066	0	12
Dnajc151	0	0.62443287	0.539	0.079	0	12
Agpat5	0	0.62136102	0.518	0.065	0	12
Exosc5	0	0.61433757	0.443	0.049	0	12
Naa10	0	0.61155362	0.456	0.06	0	12
Pam16	0	0.60438235	0.502	0.072	0	12
Magohb1	0	0.60160672	0.53	0.078	0	12
B4galnt1	0	0.59669143	0.435	0.041	0	12
Gria3	0	0.59341567	0.261	0.005	0	12
Abhd11	0	0.5811857	0.33	0.026	0	12
Echs1	0	0.57336586	0.476	0.058	0	12
Dmac1	0	0.57311561	0.413	0.05	0	12
Parl	0	0.56741088	0.424	0.048	0	12
Ppih	0	0.5667107	0.445	0.051	0	12
Kit	0	0.55850645	0.3	0.023	0	12
Mrpl44	0	0.55803094	0.304	0.023	0	12
1190007I07R	0	0.55762144	0.412	0.044	0	12
Mfsd10	0	0.52578448	0.406	0.04	0	12
Rnaseh2b	0	0.52201066	0.378	0.042	0	12
Lage3	0	0.51653439	0.473	0.062	0	12
Yif1b	0	0.50271096	0.45	0.048	0	12
Spn	0	0.4841314	0.374	0.039	0	12

Ptpn7	0	0.42418686	0.313	0.029	0	12
Bphl	0	0.42139372	0.253	0.017	0	12
Hacd1	0	0.41609988	0.352	0.033	0	12
Mogs	0	0.38737651	0.269	0.021	0	12
B230118H07	0	0.34469174	0.281	0.021	0	12
Tm7sf3	0	0.30931964	0.25	0.018	0	12
Cd481	7.17E-308	0.75969838	0.466	0.064	2.32E-303	12
Atp8b4	9.87E-308	0.65576569	0.418	0.052	3.19E-303	12
Suclg1	1.17E-307	0.77376326	0.531	0.082	3.77E-303	12
Cdca71	2.03E-305	0.60115908	0.45	0.06	6.56E-301	12
Napsa2	4.30E-305	0.93029777	0.681	0.132	1.39E-300	12
Vkorc1	7.77E-305	0.45432266	0.394	0.047	2.51E-300	12
Fh1	1.63E-303	0.54357843	0.399	0.048	5.26E-299	12
Sptssa2	1.46E-302	0.77225819	0.594	0.101	4.72E-298	12
Mcm51	3.22E-300	0.57894243	0.451	0.062	1.04E-295	12
Uchl5	3.28E-300	0.57786313	0.477	0.068	1.06E-295	12
Dnajc192	4.89E-300	0.80130191	0.665	0.125	1.58E-295	12
Cdk42	6.99E-299	0.97335991	0.678	0.129	2.26E-294	12
Idh2	1.88E-297	0.62827467	0.486	0.07	6.06E-293	12
Snx14	3.86E-297	0.51533436	0.316	0.031	1.25E-292	12
Ciao2a2	5.45E-297	1.00184452	0.674	0.132	1.76E-292	12
Lmo21	8.92E-296	0.61933264	0.46	0.065	2.88E-291	12
Nxt11	2.85E-294	0.60604703	0.425	0.056	9.20E-290	12
Snrpa11	2.86E-294	0.72287866	0.527	0.083	9.22E-290	12
Xpc	4.70E-293	0.40020302	0.339	0.037	1.52E-288	12
Mrpl11	2.30E-292	0.55166885	0.466	0.066	7.42E-288	12
Aarsd1	2.36E-292	0.42583161	0.355	0.04	7.62E-288	12
Thyn1	4.60E-292	0.44924881	0.294	0.028	1.49E-287	12
Rpa21	5.08E-291	0.55587579	0.374	0.045	1.64E-286	12
Mrpl511	1.13E-290	0.80290239	0.638	0.118	3.66E-286	12
Serpinb1a2	1.27E-290	1.41917026	0.659	0.138	4.10E-286	12
Mrto4	2.93E-290	0.59162393	0.454	0.064	9.47E-286	12
Gng12	7.06E-290	0.66559499	0.576	0.099	2.28E-285	12
Spc241	1.20E-289	0.70449411	0.479	0.072	3.88E-285	12
Stk16	2.08E-289	0.67713683	0.459	0.066	6.72E-285	12
Mrps34	1.01E-288	0.43511301	0.431	0.058	3.27E-284	12
Kdelr2	1.28E-288	0.84467895	0.643	0.123	4.12E-284	12
F630028O10	2.66E-288	1.09077406	0.54	0.093	8.60E-284	12
H2afy4	3.06E-288	1.50204393	0.837	0.227	9.88E-284	12
Lsm72	5.05E-288	0.87948324	0.696	0.139	1.63E-283	12
Mrpl27	1.72E-287	0.48764881	0.425	0.057	5.56E-283	12
Khk	4.78E-287	0.34671293	0.293	0.028	1.54E-282	12
Apex1	1.33E-284	0.55094101	0.432	0.059	4.31E-280	12

Nbdy	1.64E-284	0.34118015	0.281	0.026	5.31E-280	12
Atp5g13	3.40E-284	1.55266907	0.868	0.271	1.10E-279	12
Impa2	1.51E-283	0.55703931	0.38	0.047	4.87E-279	12
Lrpap1	5.65E-283	0.42881683	0.335	0.037	1.82E-278	12
Mcm31	6.60E-283	0.55833002	0.443	0.062	2.13E-278	12
Tmem205	8.59E-283	0.46007794	0.367	0.044	2.77E-278	12
Wdr12	5.03E-282	0.4786689	0.329	0.036	1.62E-277	12
Lsm32	1.07E-280	0.87509109	0.693	0.142	3.47E-276	12
Mrps15	3.54E-280	0.60169334	0.56	0.096	1.14E-275	12
Ebna1bp2	5.48E-280	0.63696551	0.54	0.09	1.77E-275	12
Tmem97	2.50E-279	0.39472106	0.293	0.029	8.07E-275	12
Hexa1	1.02E-278	0.5752136	0.47	0.07	3.29E-274	12
0610012G03	1.64E-278	0.65671674	0.544	0.092	5.29E-274	12
Ppia4	3.53E-276	1.48983723	0.999	0.705	1.14E-271	12
Mrpl181	1.59E-275	0.74280811	0.684	0.137	5.14E-271	12
Nudt5	2.34E-274	0.34446217	0.389	0.05	7.55E-270	12
Trappc6a	7.55E-274	0.46696479	0.431	0.061	2.44E-269	12
Bola24	3.15E-273	1.34503996	0.837	0.228	1.02E-268	12
Lman21	3.19E-273	0.8359328	0.601	0.112	1.03E-268	12
Pycard2	1.05E-272	0.79269686	0.646	0.129	3.38E-268	12
S100a1	2.47E-272	0.4971886	0.587	0.104	7.99E-268	12
Mtln	7.37E-272	0.39013235	0.457	0.067	2.38E-267	12
Rpa31	9.56E-272	0.67113626	0.619	0.116	3.09E-267	12
Exosc81	1.60E-271	0.62336483	0.521	0.086	5.15E-267	12
Erh4	3.55E-271	1.3338326	0.805	0.214	1.15E-266	12
Adssl1	5.92E-271	0.51283119	0.44	0.064	1.91E-266	12
Tsfm	5.24E-270	0.40222225	0.322	0.036	1.69E-265	12
Nup2101	1.16E-268	0.3864494	0.408	0.056	3.75E-264	12
Uck2	1.47E-268	0.33598456	0.306	0.032	4.75E-264	12
Dut3	5.70E-267	1.09554687	0.783	0.184	1.84E-262	12
Pnp2	2.57E-266	0.76895538	0.617	0.12	8.29E-262	12
Cks1b2	1.20E-265	0.9852448	0.741	0.166	3.87E-261	12
Mapkapk3	2.99E-265	0.46970598	0.317	0.035	9.64E-261	12
Mrpl35	3.47E-265	0.43228627	0.464	0.071	1.12E-260	12
Selenoh2	2.29E-264	1.02877287	0.744	0.17	7.40E-260	12
Fkbp22	6.20E-264	0.78298383	0.689	0.146	2.00E-259	12
Gng102	8.36E-264	0.84382088	0.607	0.119	2.70E-259	12
Dpy301	8.68E-264	0.73438792	0.626	0.123	2.80E-259	12
Rfc21	1.10E-262	0.63122845	0.489	0.079	3.56E-258	12
Lbp	2.93E-262	0.4054162	0.306	0.033	9.45E-258	12
Mrpl421	1.23E-261	0.54326458	0.539	0.094	3.97E-257	12
Hint2	1.44E-261	0.46435536	0.384	0.051	4.64E-257	12
Mcts11	3.09E-261	0.58477373	0.55	0.097	9.99E-257	12

Emc61	2.51E-260	0.69568608	0.613	0.119	8.11E-256	12
Mrpl581	7.75E-260	0.51893287	0.477	0.077	2.50E-255	12
Ahsa1	5.97E-259	0.55370118	0.504	0.084	1.93E-254	12
Bcap29	1.27E-258	0.32972568	0.285	0.029	4.10E-254	12
Nop16	6.46E-258	0.44092568	0.333	0.04	2.09E-253	12
Lsm21	1.19E-257	0.66090495	0.579	0.108	3.85E-253	12
Stmn12	4.62E-257	1.20715411	0.876	0.246	1.49E-252	12
Mrpl17	3.76E-256	0.60953529	0.505	0.085	1.21E-251	12
Snhg31	1.37E-255	0.79956126	0.614	0.122	4.43E-251	12
Dnmt11	2.49E-255	0.6431954	0.544	0.098	8.04E-251	12
Tma16	4.91E-254	0.4451653	0.323	0.038	1.58E-249	12
Naa383	6.99E-254	0.79402952	0.686	0.148	2.26E-249	12
Phpt1	1.13E-253	0.29378461	0.322	0.038	3.66E-249	12
Rnaset2a	2.99E-253	0.36432562	0.352	0.045	9.64E-249	12
Tmem1601	3.91E-253	0.66802071	0.639	0.13	1.26E-248	12
Rbmxl11	4.79E-253	0.47436544	0.425	0.063	1.55E-248	12
Timm9	7.80E-253	0.4233674	0.306	0.034	2.52E-248	12
Clns1a	9.04E-253	0.37909112	0.393	0.055	2.92E-248	12
Snrnp401	1.05E-252	0.66032317	0.431	0.065	3.40E-248	12
Sumo3	1.62E-252	0.51592099	0.504	0.085	5.22E-248	12
Ccdc115	2.38E-252	0.33061222	0.282	0.029	7.69E-248	12
Park72	2.63E-252	1.18210903	0.809	0.217	8.50E-248	12
Cycs4	6.31E-251	1.33637661	0.795	0.228	2.04E-246	12
Kcnab21	1.14E-250	0.43709759	0.403	0.058	3.68E-246	12
Mrps121	1.36E-250	0.50950679	0.511	0.088	4.39E-246	12
Cmc11	3.52E-250	0.38610485	0.456	0.072	1.14E-245	12
Pa2g41	3.71E-250	0.75968534	0.617	0.124	1.20E-245	12
Mrps25	3.89E-250	0.32172579	0.409	0.059	1.26E-245	12
Mrpl19	1.06E-248	0.34609068	0.403	0.058	3.44E-244	12
Znhit3	1.19E-248	0.38593192	0.266	0.027	3.83E-244	12
Psmg41	2.43E-248	0.50852302	0.565	0.105	7.85E-244	12
Cuedc2	1.04E-247	0.6042548	0.576	0.11	3.36E-243	12
Coa32	6.35E-247	0.61057824	0.587	0.114	2.05E-242	12
Nudc1	1.69E-245	0.74202348	0.607	0.122	5.45E-241	12
Gsto1	2.31E-245	0.42325551	0.467	0.076	7.45E-241	12
Nsmce4a1	3.11E-245	0.61123243	0.568	0.108	1.00E-240	12
Hprt2	3.20E-245	0.67074807	0.575	0.111	1.03E-240	12
Timm231	5.13E-245	0.70799803	0.607	0.122	1.66E-240	12
Tmx41	1.24E-244	0.64724756	0.448	0.073	3.99E-240	12
Lyl1	2.54E-244	0.31352196	0.282	0.031	8.19E-240	12
Ndufaf81	6.18E-244	0.48236498	0.565	0.107	2.00E-239	12
Ruvbl2	7.61E-244	0.46800989	0.335	0.042	2.46E-239	12
Ptrhd1	9.96E-244	0.34368609	0.39	0.056	3.21E-239	12

Cacybp	3.94E-243	0.77726991	0.61	0.123	1.27E-238	12
Bet1	3.99E-243	0.45860285	0.365	0.05	1.29E-238	12
Acer31	8.62E-242	0.40721925	0.304	0.035	2.78E-237	12
Mrpl22	1.47E-241	0.37771747	0.419	0.064	4.74E-237	12
Rpgrip12	1.75E-241	0.61549383	0.571	0.111	5.64E-237	12
Eif3i3	1.83E-241	0.94403891	0.699	0.159	5.90E-237	12
Tomm40	2.14E-241	0.43214492	0.367	0.05	6.92E-237	12
Asrgl1	9.23E-241	0.45864071	0.253	0.025	2.98E-236	12
AI506816	1.80E-240	0.36125827	0.451	0.072	5.80E-236	12
Rasgrp23	7.17E-240	0.64825888	0.635	0.136	2.32E-235	12
Smim113	8.20E-240	0.52047243	0.623	0.128	2.65E-235	12
Hmgn23	2.54E-239	1.58404499	0.911	0.359	8.20E-235	12
Ubl4a	5.65E-239	0.40688652	0.444	0.072	1.82E-234	12
Mrpl16	1.48E-238	0.38090306	0.329	0.042	4.79E-234	12
Acaa2	1.98E-238	0.28588015	0.313	0.038	6.38E-234	12
Glipr11	5.08E-238	0.74239135	0.504	0.092	1.64E-233	12
Nt5c1	6.39E-238	0.44304851	0.543	0.101	2.06E-233	12
Mrpl232	1.17E-237	0.55265137	0.643	0.135	3.77E-233	12
Selenos1	1.96E-237	0.62398039	0.581	0.115	6.34E-233	12
Snrnp25	1.79E-236	0.33899157	0.335	0.043	5.79E-232	12
Ufc11	2.00E-236	0.52814942	0.557	0.107	6.47E-232	12
Snhg91	3.24E-236	0.58556614	0.616	0.127	1.05E-231	12
Lig11	3.68E-236	0.53260466	0.507	0.091	1.19E-231	12
Gtf2f2	4.36E-236	0.31895523	0.25	0.025	1.41E-231	12
Phf5a2	2.48E-235	0.56802938	0.627	0.13	8.00E-231	12
Sec61b3	2.65E-235	1.59883323	0.897	0.438	8.57E-231	12
A430005L14I	4.66E-235	0.32912357	0.282	0.032	1.50E-230	12
Stk26	6.13E-235	0.38979297	0.332	0.043	1.98E-230	12
Ostc1	4.05E-234	0.92402153	0.707	0.167	1.31E-229	12
Tor1a1	5.02E-234	0.43924835	0.4	0.061	1.62E-229	12
Bcat2	3.94E-233	0.27420005	0.253	0.026	1.27E-228	12
Rnaset2b	8.03E-233	0.27026517	0.32	0.04	2.59E-228	12
Atp6ap2	1.51E-232	0.55151432	0.485	0.086	4.86E-228	12
Mcm61	3.59E-232	0.52029364	0.476	0.083	1.16E-227	12
Ncbp2	5.36E-232	0.45885555	0.406	0.062	1.73E-227	12
Nans	5.76E-232	0.46616357	0.376	0.054	1.86E-227	12
M6pr1	7.89E-232	0.42284545	0.467	0.08	2.55E-227	12
Ssna12	1.96E-231	0.62850395	0.6	0.123	6.33E-227	12
Haus1	4.48E-231	0.39910276	0.275	0.031	1.45E-226	12
Cuta3	9.55E-231	0.73031138	0.69	0.16	3.08E-226	12
Fdps	1.22E-230	0.38214896	0.488	0.085	3.95E-226	12
Lta4h2	1.43E-230	0.92143489	0.601	0.131	4.62E-226	12
Osgep	1.90E-230	0.51032808	0.377	0.055	6.13E-226	12

Tmem223	2.89E-230	0.42129836	0.402	0.062	9.33E-226	12
Rpia	8.15E-230	0.32397259	0.371	0.054	2.63E-225	12
Tspan32	3.79E-229	0.46207928	0.287	0.034	1.22E-224	12
Mrps26	4.79E-229	0.41203567	0.421	0.067	1.55E-224	12
Tent5a	6.09E-229	0.33293044	0.317	0.04	1.97E-224	12
Ormdl2	1.30E-228	0.44492644	0.341	0.046	4.19E-224	12
Eif5a3	3.04E-228	1.46502429	0.924	0.396	9.81E-224	12
Wdr74	5.37E-228	0.30726073	0.297	0.036	1.73E-223	12
Phb21	6.22E-228	0.67323964	0.572	0.115	2.01E-223	12
Mcm71	7.39E-228	0.45819764	0.61	0.125	2.39E-223	12
Mrpl2	9.38E-228	0.43164494	0.371	0.054	3.03E-223	12
Srsf72	1.09E-227	1.03120588	0.787	0.209	3.51E-223	12
Sar1b	1.86E-227	0.51126672	0.386	0.058	5.99E-223	12
Spcs21	2.64E-227	0.8995509	0.731	0.184	8.54E-223	12
Bola1	1.55E-226	0.40314905	0.397	0.061	5.01E-222	12
Ndufa9	2.01E-225	0.33114568	0.361	0.051	6.49E-221	12
Eef1akmt1	2.07E-225	0.26573155	0.294	0.035	6.68E-221	12
Exosc31	7.97E-225	0.51816688	0.428	0.07	2.57E-220	12
Ranbp12	9.01E-225	1.16609215	0.812	0.227	2.91E-220	12
Pdia61	1.31E-224	0.76363532	0.643	0.144	4.23E-220	12
Sf3b53	1.33E-224	0.90901315	0.763	0.198	4.28E-220	12
Nifk	1.46E-224	0.44040128	0.387	0.059	4.72E-220	12
Erp294	2.14E-224	0.93370306	0.763	0.198	6.91E-220	12
Edem2	2.34E-224	0.34847423	0.278	0.032	7.55E-220	12
Spcs3	2.88E-224	0.38288985	0.508	0.094	9.31E-220	12
Glod4	4.96E-224	0.35752093	0.346	0.048	1.60E-219	12
Wdr18	5.07E-224	0.32503289	0.266	0.029	1.64E-219	12
Rab44	1.78E-223	0.48022533	0.354	0.05	5.75E-219	12
Gpc1	2.18E-223	0.33173426	0.319	0.041	7.04E-219	12
Uxt	2.33E-223	0.38205317	0.329	0.044	7.54E-219	12
Nasp1	2.35E-223	0.50849481	0.553	0.109	7.58E-219	12
Ncln	2.51E-223	0.25462783	0.298	0.037	8.12E-219	12
Npm3	6.36E-223	0.57999049	0.499	0.092	2.05E-218	12
Mrpl281	1.19E-222	0.51278212	0.496	0.091	3.85E-218	12
Txnl4a	1.25E-221	0.48882409	0.483	0.088	4.02E-217	12
Nucb1	1.29E-221	0.30826293	0.454	0.078	4.16E-217	12
Nubp2	3.22E-221	0.28796481	0.277	0.032	1.04E-216	12
Rsl24d1	4.39E-221	0.35614627	0.434	0.073	1.42E-216	12
Ssr1	4.64E-221	0.56669654	0.648	0.145	1.50E-216	12
Cdk11	1.31E-220	0.78241603	0.485	0.09	4.22E-216	12
Rfc5	1.85E-220	0.35064615	0.275	0.032	5.96E-216	12
Pole41	2.16E-220	0.32818174	0.501	0.093	6.96E-216	12
C1d	1.73E-219	0.60095543	0.483	0.089	5.58E-215	12

Gstp13	2.01E-219	0.49422209	0.626	0.134	6.50E-215	12
Rps214	2.23E-219	1.37092501	0.98	0.624	7.20E-215	12
Ndufv23	3.56E-219	0.73390597	0.67	0.155	1.15E-214	12
Cmss1	4.27E-219	0.31936207	0.268	0.03	1.38E-214	12
Ppie	5.66E-219	0.25218466	0.303	0.038	1.83E-214	12
Ddx391	1.29E-218	0.53124495	0.528	0.103	4.15E-214	12
Bloc1s21	2.07E-218	0.52310824	0.552	0.111	6.69E-214	12
Bri3bp	4.69E-218	0.43263954	0.298	0.037	1.51E-213	12
Vma21	6.10E-218	0.38691404	0.432	0.073	1.97E-213	12
Cdca81	8.49E-218	0.55318657	0.464	0.083	2.74E-213	12
Psmb84	1.02E-217	0.95399465	0.758	0.203	3.28E-213	12
Stoml2	1.53E-217	0.34814555	0.393	0.062	4.93E-213	12
Ptrh2	3.33E-217	0.28767952	0.274	0.032	1.08E-212	12
Nr2c2ap	5.36E-217	0.29131464	0.275	0.032	1.73E-212	12
Fam173a	5.75E-217	0.44246281	0.483	0.089	1.86E-212	12
Mrpl4	7.57E-217	0.37971658	0.361	0.053	2.45E-212	12
Erp44	1.05E-216	0.38865949	0.351	0.051	3.40E-212	12
Dbi5	1.16E-216	1.2556357	0.863	0.297	3.76E-212	12
Mdh12	6.14E-216	0.54903713	0.594	0.127	1.98E-211	12
Mrps7	6.79E-216	0.42529644	0.367	0.055	2.19E-211	12
Sec11a2	1.05E-215	0.63232952	0.581	0.123	3.38E-211	12
Fbl	1.29E-215	0.59450928	0.521	0.101	4.16E-211	12
Cope1	2.12E-215	0.71011499	0.677	0.161	6.83E-211	12
Prmt11	5.85E-215	0.61520539	0.569	0.118	1.89E-210	12
Baz1a	1.05E-214	0.46053596	0.518	0.102	3.39E-210	12
Tfdp1	1.37E-214	0.4888671	0.457	0.082	4.41E-210	12
Snrpd13	3.30E-214	1.11004016	0.822	0.245	1.06E-209	12
Ptgr1	3.42E-214	0.44131703	0.279	0.034	1.10E-209	12
Ctsz2	3.45E-214	0.64634632	0.678	0.159	1.11E-209	12
Ndufa52	4.63E-214	0.82567247	0.738	0.193	1.50E-209	12
Ppa1	6.13E-214	0.46449442	0.383	0.06	1.98E-209	12
Ndufab12	8.35E-214	0.97279229	0.731	0.192	2.70E-209	12
Fabp52	9.96E-214	0.69771775	0.507	0.101	3.21E-209	12
Pop5	1.21E-213	0.40323582	0.387	0.061	3.90E-209	12
Zfp593	1.94E-213	0.39366406	0.328	0.045	6.27E-209	12
Ndufb23	2.90E-213	0.95000204	0.761	0.212	9.38E-209	12
Ethe13	3.15E-213	0.65888139	0.588	0.128	1.02E-208	12
Uqcrc12	6.72E-213	0.62683979	0.581	0.124	2.17E-208	12
Uqcrb4	1.34E-212	1.27491611	0.844	0.31	4.33E-208	12
Sdhd	1.48E-212	0.57853992	0.502	0.096	4.78E-208	12
Timm22	1.72E-212	0.32025906	0.32	0.044	5.57E-208	12
Mrpl1	8.00E-212	0.26847979	0.316	0.043	2.58E-207	12
Psmc5	1.10E-211	0.42279468	0.477	0.088	3.56E-207	12

Txn2	1.21E-211	0.59134223	0.527	0.105	3.90E-207	12
Tmed91	1.40E-211	0.61729991	0.648	0.148	4.53E-207	12
Mrpl50	1.42E-211	0.33909822	0.309	0.041	4.57E-207	12
Eif4a31	1.64E-211	0.62886248	0.507	0.099	5.29E-207	12
Mrpl15	1.71E-211	0.35596927	0.472	0.087	5.52E-207	12
Lyar	2.07E-211	0.4712286	0.444	0.078	6.69E-207	12
Dhrs4	2.38E-211	0.25186044	0.253	0.028	7.68E-207	12
Ptpmt1	2.51E-211	0.42826496	0.301	0.039	8.09E-207	12
Eif1ad	3.79E-211	0.32384989	0.348	0.051	1.22E-206	12
Gfer	1.12E-210	0.32535744	0.282	0.035	3.60E-206	12
Bax2	1.30E-210	0.54306285	0.627	0.139	4.20E-206	12
Mrps243	1.44E-210	0.632147	0.674	0.159	4.64E-206	12
Ndufv1	2.60E-210	0.32831922	0.419	0.071	8.39E-206	12
Slc25a54	2.68E-210	1.13487175	0.833	0.256	8.66E-206	12
Galk1	4.29E-210	0.34584976	0.311	0.042	1.38E-205	12
Pgp2	9.06E-210	0.47272625	0.461	0.085	2.92E-205	12
Dcps	1.09E-209	0.36788813	0.328	0.046	3.53E-205	12
H2afz3	1.98E-209	1.30348204	0.971	0.566	6.40E-205	12
Ppil1	2.98E-209	0.32722847	0.332	0.047	9.64E-205	12
Umps	5.81E-209	0.36065553	0.274	0.033	1.88E-204	12
Psma12	7.62E-209	0.65225003	0.645	0.149	2.46E-204	12
Cenph	1.26E-208	0.32665908	0.304	0.04	4.06E-204	12
Syngr2	3.46E-208	0.36933966	0.438	0.077	1.12E-203	12
Tmem208	8.51E-208	0.4663964	0.521	0.104	2.75E-203	12
Psma53	1.99E-207	0.79162146	0.686	0.168	6.43E-203	12
Commd7	2.57E-207	0.32339334	0.396	0.065	8.30E-203	12
Tctex1d2	2.90E-207	0.39851293	0.344	0.051	9.38E-203	12
Nme12	3.70E-207	1.09503044	0.786	0.225	1.19E-202	12
Pde6d	4.61E-207	0.33666266	0.282	0.035	1.49E-202	12
Rexo21	4.65E-207	0.46674751	0.512	0.101	1.50E-202	12
Ndufa10	1.43E-206	0.54127845	0.479	0.091	4.62E-202	12
0610010K14I	2.26E-206	0.43483582	0.496	0.096	7.31E-202	12
Dnajb11	3.68E-206	0.37412824	0.403	0.067	1.19E-201	12
Ndufs73	8.11E-206	0.70476757	0.693	0.172	2.62E-201	12
Mrpl543	1.38E-205	0.52484856	0.594	0.131	4.44E-201	12
Higd1a2	1.75E-205	0.53936302	0.706	0.172	5.65E-201	12
Birc51	2.08E-205	0.79903272	0.598	0.135	6.72E-201	12
Lmo1	2.61E-205	0.39573153	0.297	0.039	8.43E-201	12
Rps27a5	3.00E-205	1.01365226	0.996	0.891	9.69E-201	12
Ift27	3.09E-205	0.34277791	0.271	0.033	9.96E-201	12
Anapc15	4.03E-205	0.3365097	0.328	0.047	1.30E-200	12
Eif1b1	1.12E-204	0.49338558	0.555	0.118	3.63E-200	12
Emc71	1.24E-204	0.46260041	0.493	0.096	4.00E-200	12

Erg28	1.38E-204	0.28050626	0.313	0.043	4.46E-200	12
Nip7	1.44E-204	0.39661127	0.339	0.05	4.65E-200	12
Sephs2	1.64E-204	0.40217154	0.46	0.086	5.28E-200	12
H2-DMa1	1.95E-204	0.35800549	0.402	0.067	6.31E-200	12
Hmgn51	4.76E-204	0.38115151	0.383	0.062	1.54E-199	12
Slirp1	5.82E-204	0.51845804	0.566	0.121	1.88E-199	12
Dnajc91	6.26E-204	0.41843825	0.472	0.089	2.02E-199	12
Arl6ip4	1.36E-203	0.46870171	0.421	0.074	4.38E-199	12
Mrps18c1	1.40E-203	0.46794874	0.601	0.134	4.51E-199	12
Mcmbp1	3.51E-203	0.38709931	0.459	0.085	1.13E-198	12
Mrpl55	3.93E-203	0.41180279	0.326	0.047	1.27E-198	12
Rps19bp1	5.34E-203	0.35959309	0.396	0.066	1.72E-198	12
Psme21	7.68E-203	0.70230236	0.687	0.172	2.48E-198	12
Rrm21	7.92E-203	0.46693811	0.456	0.085	2.56E-198	12
Ddt1	1.27E-202	0.27120528	0.406	0.069	4.09E-198	12
Glrx3	1.56E-202	0.36147335	0.492	0.095	5.04E-198	12
Ppil3	2.35E-202	0.36662054	0.285	0.037	7.58E-198	12
Nop105	2.60E-202	1.1913089	0.773	0.24	8.40E-198	12
Psmb101	2.81E-202	0.42309512	0.547	0.113	9.06E-198	12
Tm6sf11	5.42E-202	0.38252355	0.389	0.064	1.75E-197	12
Mcm4	1.73E-201	0.41967158	0.348	0.053	5.58E-197	12
Lsm63	1.83E-201	0.84836362	0.763	0.211	5.92E-197	12
Phgdh	1.92E-201	0.61977338	0.479	0.093	6.21E-197	12
Camta1	6.47E-201	0.33668288	0.361	0.057	2.09E-196	12
Psmb23	1.02E-200	0.90285903	0.758	0.214	3.29E-196	12
Mrpl40	1.15E-200	0.37140747	0.344	0.052	3.70E-196	12
Pdia4	1.24E-200	0.40304265	0.518	0.105	3.99E-196	12
Pclaf1	1.40E-200	0.85075954	0.636	0.156	4.53E-196	12
Mrps143	2.23E-200	0.69781452	0.696	0.179	7.19E-196	12
Rrp15	2.63E-200	0.31192699	0.272	0.034	8.48E-196	12
Emg12	3.76E-200	0.60236197	0.616	0.141	1.21E-195	12
Rfc41	8.10E-200	0.43306575	0.332	0.049	2.62E-195	12
Ndufb34	8.93E-200	0.94646394	0.785	0.235	2.88E-195	12
Polr2d	2.68E-199	0.36179717	0.332	0.049	8.64E-195	12
Ndufb62	3.36E-199	0.44677241	0.595	0.134	1.09E-194	12
Fkbp4	8.09E-199	0.45689793	0.525	0.108	2.61E-194	12
Ndufa83	8.77E-199	0.69994214	0.668	0.165	2.83E-194	12
Cdk2ap1	1.12E-198	0.25830896	0.325	0.047	3.62E-194	12
Rer12	1.71E-198	0.55249674	0.592	0.134	5.52E-194	12
Timm17a	2.10E-198	0.38853953	0.403	0.069	6.79E-194	12
Comtd1	3.94E-198	0.36041517	0.25	0.029	1.27E-193	12
Srsf93	5.59E-198	0.70982488	0.699	0.178	1.81E-193	12
Ssbp11	1.29E-197	0.57136229	0.607	0.14	4.17E-193	12

Uqcrfs13	1.64E-197	0.72468768	0.655	0.16	5.31E-193	12
Mrpl303	1.84E-197	0.59671247	0.677	0.166	5.93E-193	12
Tomm51	3.08E-197	0.55806175	0.579	0.129	9.95E-193	12
Emc2	3.35E-197	0.28554292	0.376	0.062	1.08E-192	12
Polr2j3	3.72E-197	0.55209891	0.684	0.167	1.20E-192	12
Timm10	4.04E-197	0.28354892	0.307	0.043	1.31E-192	12
Polr2h	4.61E-197	0.38919932	0.352	0.055	1.49E-192	12
Rps186	7.20E-197	1.25732321	0.964	0.644	2.32E-192	12
Nudcd2	7.62E-197	0.41771188	0.352	0.055	2.46E-192	12
Manf1	9.35E-197	0.82438082	0.726	0.196	3.02E-192	12
Utp11	9.96E-197	0.40319926	0.479	0.093	3.22E-192	12
Fermt31	1.08E-196	0.49317616	0.574	0.127	3.48E-192	12
Ndufb53	1.83E-196	0.85844474	0.761	0.217	5.90E-192	12
Pole3	2.09E-196	0.3121047	0.339	0.052	6.74E-192	12
Bin1	2.61E-196	0.25348096	0.338	0.051	8.43E-192	12
Samm50	3.23E-196	0.3424438	0.342	0.053	1.04E-191	12
Rpn2	6.13E-196	0.34742726	0.553	0.118	1.98E-191	12
Hypk	6.47E-196	0.38935795	0.453	0.086	2.09E-191	12
Zcchc17	6.64E-196	0.25811776	0.357	0.056	2.14E-191	12
Ndufa122	1.24E-195	0.49941789	0.62	0.143	4.01E-191	12
1500011B03I	1.29E-195	0.25665839	0.252	0.03	4.17E-191	12
Mrps18a	3.23E-195	0.42191777	0.415	0.074	1.04E-190	12
Dohh	5.43E-195	0.30524147	0.282	0.037	1.75E-190	12
C1galt1c11	6.49E-195	0.46641989	0.265	0.034	2.10E-190	12
Mrpl203	8.25E-195	0.74112048	0.706	0.187	2.66E-190	12
Nrros1	9.62E-195	0.33615197	0.355	0.056	3.11E-190	12
Lsm53	1.54E-194	0.83475682	0.76	0.214	4.97E-190	12
Bex3	1.94E-194	0.45749054	0.453	0.086	6.26E-190	12
Irf81	2.47E-194	0.57694414	0.374	0.063	7.97E-190	12
Polr2f1	4.52E-194	0.50970082	0.64	0.152	1.46E-189	12
Mpc11	7.69E-194	0.67157706	0.672	0.171	2.48E-189	12
2-Mar	1.02E-193	0.37985505	0.403	0.071	3.30E-189	12
Cdc261	1.07E-193	0.4273299	0.476	0.094	3.45E-189	12
Gorasp2	1.81E-193	0.30707334	0.323	0.048	5.83E-189	12
Htra2	2.92E-193	0.39189755	0.284	0.038	9.43E-189	12
Lamtor52	5.63E-193	0.3781195	0.559	0.122	1.82E-188	12
Pno1	1.31E-192	0.28250615	0.285	0.038	4.22E-188	12
Cnpy2	3.41E-192	0.31584894	0.44	0.082	1.10E-187	12
Apopt1	7.33E-192	0.35193379	0.384	0.066	2.37E-187	12
Bcl2l12	8.79E-192	0.3583151	0.271	0.035	2.84E-187	12
Glo1	1.31E-191	0.33497907	0.412	0.074	4.23E-187	12
Stub1	1.35E-191	0.51082852	0.492	0.101	4.34E-187	12
Bcl7c1	1.97E-191	0.41715369	0.443	0.083	6.36E-187	12

Parvg	2.31E-191	0.4076778	0.362	0.06	7.45E-187	12
Ran2	3.19E-191	1.25962492	0.923	0.342	1.03E-186	12
Polr3k	4.93E-191	0.33430281	0.383	0.065	1.59E-186	12
Sdhc	6.14E-191	0.3619739	0.339	0.053	1.98E-186	12
Polr2g1	1.09E-190	0.29877595	0.524	0.11	3.52E-186	12
Vrk1	1.62E-190	0.3828256	0.418	0.076	5.24E-186	12
Ifngr11	5.90E-190	0.57610195	0.509	0.108	1.91E-185	12
Ufm11	6.29E-190	0.3865139	0.553	0.121	2.03E-185	12
Tk1	8.28E-190	0.32436239	0.294	0.041	2.67E-185	12
Ttc32	8.31E-190	0.32951589	0.31	0.045	2.68E-185	12
Tmem2563	1.11E-189	0.98023767	0.808	0.262	3.58E-185	12
Snhg15	2.47E-189	0.28961067	0.355	0.058	7.96E-185	12
Ndufc23	4.21E-189	0.81523716	0.76	0.215	1.36E-184	12
Trappc5	4.83E-189	0.36525749	0.406	0.073	1.56E-184	12
Cdk6	4.89E-189	0.53681044	0.482	0.098	1.58E-184	12
Tex261	5.55E-189	0.3428689	0.374	0.063	1.79E-184	12
Krtcap23	5.82E-189	0.97401827	0.786	0.242	1.88E-184	12
Ndufs31	6.54E-189	0.39999205	0.493	0.101	2.11E-184	12
Gm16286	6.87E-189	0.30774638	0.432	0.081	2.22E-184	12
Creg12	6.98E-189	0.45030454	0.613	0.144	2.25E-184	12
Uqcc3	1.05E-188	0.30319384	0.441	0.084	3.38E-184	12
Cmc21	2.43E-188	0.37794345	0.431	0.081	7.86E-184	12
Ndufs83	3.01E-188	0.61278913	0.649	0.162	9.71E-184	12
Mlec	3.42E-188	0.40582662	0.508	0.106	1.10E-183	12
Psma62	4.70E-188	0.5022907	0.629	0.151	1.52E-183	12
Rnaseh2c3	5.25E-188	0.81566727	0.753	0.218	1.70E-183	12
Gm118083	6.04E-188	0.44643668	0.654	0.16	1.95E-183	12
Fcnb1	1.02E-187	1.35574054	0.297	0.046	3.28E-183	12
Psmd6	1.06E-187	0.37660328	0.512	0.108	3.43E-183	12
Eny22	1.78E-187	0.58299311	0.689	0.175	5.75E-183	12
Kpna2	2.64E-187	0.33064625	0.271	0.036	8.53E-183	12
Ciapin1	3.06E-187	0.29837715	0.268	0.035	9.88E-183	12
Mrps17	3.22E-187	0.3952555	0.466	0.092	1.04E-182	12
Ak21	3.46E-187	0.60176264	0.55	0.124	1.12E-182	12
Ndufaf2	3.76E-187	0.30753656	0.377	0.064	1.22E-182	12
Rrm11	6.92E-187	0.35206875	0.432	0.081	2.24E-182	12
Aurkaip12	1.14E-186	0.58562779	0.646	0.162	3.68E-182	12
Dnajc3	1.17E-186	0.27610812	0.447	0.086	3.76E-182	12
Nubp1	1.34E-186	0.36209569	0.322	0.049	4.32E-182	12
Tmem38b	1.36E-186	0.44385854	0.288	0.041	4.40E-182	12
Smn1	1.62E-186	0.2613862	0.426	0.08	5.24E-182	12
Bzw2	2.19E-186	0.40349291	0.338	0.053	7.08E-182	12
Ccdc167	3.12E-186	0.32868595	0.333	0.053	1.01E-181	12

Cdc34	3.38E-186	0.34377224	0.399	0.071	1.09E-181	12
Cinp	4.06E-186	0.33740427	0.253	0.032	1.31E-181	12
Rpsa4	6.06E-186	1.12263984	0.994	0.697	1.96E-181	12
Rab32	6.60E-186	0.28048711	0.29	0.041	2.13E-181	12
Mrps11	7.99E-186	0.25347071	0.313	0.047	2.58E-181	12
Rnh11	8.03E-186	0.40740696	0.475	0.096	2.59E-181	12
Alg5	1.48E-185	0.33286291	0.319	0.048	4.78E-181	12
Mad2l1	3.54E-185	0.33915881	0.314	0.047	1.14E-180	12
Zfp771	4.41E-185	0.33286586	0.25	0.031	1.42E-180	12
Rassf41	4.94E-185	0.32040987	0.263	0.034	1.59E-180	12
Llph3	5.10E-185	0.63247692	0.706	0.186	1.65E-180	12
Hspa14	8.72E-185	0.28110843	0.349	0.057	2.81E-180	12
Dad13	1.17E-184	0.77892075	0.783	0.23	3.78E-180	12
Smc21	1.23E-184	0.40109421	0.524	0.113	3.97E-180	12
Rnf126	1.32E-184	0.25125808	0.266	0.035	4.25E-180	12
Hells1	1.46E-184	0.3302236	0.428	0.081	4.72E-180	12
Igsf62	3.61E-184	0.46211515	0.392	0.07	1.17E-179	12
Chchd13	4.39E-184	0.57574154	0.678	0.174	1.42E-179	12
Mapkapk21	5.63E-184	0.34238055	0.511	0.109	1.82E-179	12
Dtnbp1	7.74E-184	0.32817436	0.354	0.059	2.50E-179	12
Psma42	1.05E-183	0.6238471	0.652	0.164	3.39E-179	12
Smim41	1.76E-183	0.6131624	0.626	0.159	5.67E-179	12
Dcp21	1.87E-183	0.2824772	0.419	0.078	6.04E-179	12
Timm133	2.92E-183	0.77532359	0.76	0.222	9.42E-179	12
Mrps163	3.75E-183	0.49023297	0.617	0.15	1.21E-178	12
Pgam12	4.83E-183	0.89574262	0.802	0.255	1.56E-178	12
Ift20	5.14E-183	0.31172818	0.473	0.096	1.66E-178	12
Pin11	3.70E-182	0.39304008	0.451	0.089	1.20E-177	12
Gadd45gip11	3.81E-182	0.40679018	0.486	0.1	1.23E-177	12
Cnpy3	1.28E-181	0.28145665	0.29	0.041	4.15E-177	12
Mrpl41	1.67E-181	0.28846555	0.387	0.069	5.39E-177	12
Rex1bd2	1.74E-181	0.37654443	0.559	0.127	5.62E-177	12
Smim27	4.71E-181	0.39008797	0.426	0.082	1.52E-176	12
Psmb53	5.24E-181	0.78652907	0.747	0.215	1.69E-176	12
Rnf72	6.44E-181	0.47601497	0.604	0.145	2.08E-176	12
Hpf11	7.04E-181	0.38512187	0.502	0.107	2.27E-176	12
Eif3g	7.74E-181	0.35915858	0.467	0.095	2.50E-176	12
Rps27l4	9.31E-181	1.18014724	0.865	0.335	3.00E-176	12
Tuba1b3	1.58E-180	0.78859152	0.764	0.222	5.11E-176	12
Micos131	2.00E-180	0.64238153	0.707	0.192	6.45E-176	12
Cox5a4	2.21E-180	1.20692803	0.885	0.378	7.13E-176	12
Rpn11	2.89E-180	0.57972794	0.636	0.162	9.33E-176	12
Rnaseh2a	1.06E-179	0.29773668	0.317	0.049	3.43E-175	12

B930036N10	1.58E-179	0.33950947	0.377	0.067	5.10E-175	12
Ppan	2.05E-179	0.33696822	0.253	0.033	6.62E-175	12
Tpgs1	2.60E-179	0.29129037	0.261	0.035	8.40E-175	12
Nrm1	3.10E-179	0.28237763	0.32	0.05	1.00E-174	12
Commd12	3.28E-179	0.54524084	0.571	0.135	1.06E-174	12
Tmco12	1.72E-178	0.46184282	0.608	0.147	5.54E-174	12
Mrpl572	2.13E-178	0.54328932	0.668	0.174	6.87E-174	12
Nin1	2.69E-178	0.38436545	0.41	0.078	8.67E-174	12
Timm50	3.27E-178	0.35827082	0.323	0.051	1.06E-173	12
Taf103	7.87E-178	0.68915658	0.755	0.218	2.54E-173	12
Pebp11	8.34E-178	0.74925415	0.707	0.19	2.69E-173	12
Atic	1.71E-177	0.31900313	0.311	0.048	5.51E-173	12
Akr1b31	2.01E-177	0.35101238	0.429	0.083	6.49E-173	12
Spint2	8.27E-177	0.35855289	0.365	0.064	2.67E-172	12
1700123O20	1.34E-176	0.32959305	0.346	0.059	4.33E-172	12
Rsl1d1	2.15E-176	0.43070023	0.425	0.083	6.93E-172	12
Pts	2.37E-176	0.2675359	0.373	0.066	7.64E-172	12
Bola32	2.59E-176	0.35074884	0.514	0.112	8.36E-172	12
Gspt1	5.17E-176	0.32045587	0.464	0.095	1.67E-171	12
Acat1	7.43E-176	0.31713741	0.387	0.071	2.40E-171	12
Tm2d3	9.41E-176	0.30600605	0.265	0.036	3.04E-171	12
Mphosph10	1.35E-175	0.27906881	0.291	0.043	4.36E-171	12
Ptges32	1.58E-175	0.79209867	0.757	0.219	5.11E-171	12
Zfp422	1.96E-175	0.26360762	0.258	0.035	6.34E-171	12
Rpl326	1.97E-175	1.07568436	0.984	0.727	6.36E-171	12
Atp5g33	2.07E-175	0.92592371	0.806	0.258	6.67E-171	12
Creld2	4.10E-175	0.31894289	0.274	0.039	1.32E-170	12
Lamtor23	4.18E-175	0.92815119	0.782	0.256	1.35E-170	12
Idh3a	4.89E-175	0.29810848	0.297	0.045	1.58E-170	12
Ndufa43	7.75E-175	1.23610795	0.891	0.456	2.50E-170	12
Pop7	8.18E-175	0.32027957	0.355	0.061	2.64E-170	12
Tmbim41	9.00E-175	0.35960287	0.552	0.127	2.91E-170	12
Cetn31	2.51E-174	0.45247266	0.62	0.153	8.09E-170	12
Cebpd3	3.38E-174	0.48102904	0.536	0.123	1.09E-169	12
Tmem14c2	3.40E-174	0.66108745	0.753	0.222	1.10E-169	12
Mrps23	5.07E-174	0.32375323	0.342	0.058	1.64E-169	12
Uqcrc2	1.20E-173	0.38975895	0.426	0.084	3.87E-169	12
Cct81	1.76E-173	0.53683949	0.608	0.149	5.68E-169	12
Sypl	1.81E-173	0.25381166	0.438	0.087	5.84E-169	12
Twf22	2.03E-173	0.38337823	0.463	0.097	6.54E-169	12
Nipsnap3b	2.18E-173	0.30310984	0.316	0.05	7.03E-169	12
Uchl31	2.74E-173	0.29615326	0.472	0.098	8.85E-169	12
Ddrgk1	3.16E-173	0.26935548	0.378	0.069	1.02E-168	12

Hk32	3.78E-173	0.51276925	0.422	0.084	1.22E-168	12
Eif2s1	7.21E-173	0.34718271	0.469	0.098	2.33E-168	12
Sod21	9.50E-173	0.46071741	0.502	0.111	3.07E-168	12
Glrx21	2.84E-172	0.28387533	0.432	0.086	9.17E-168	12
Mis18a	7.71E-172	0.34534342	0.262	0.036	2.49E-167	12
Kxd1	1.40E-171	0.25379842	0.33	0.055	4.53E-167	12
U2af12	3.68E-171	0.76448948	0.728	0.211	1.19E-166	12
Fam174a1	1.15E-170	0.25342287	0.326	0.054	3.72E-166	12
Sae11	1.34E-170	0.43587109	0.377	0.069	4.32E-166	12
Nfu11	1.96E-170	0.49747257	0.48	0.104	6.34E-166	12
Ebp	4.92E-170	0.27336778	0.364	0.065	1.59E-165	12
Ly862	7.13E-170	0.52070363	0.392	0.075	2.30E-165	12
Mrpl342	7.79E-170	0.47288829	0.624	0.158	2.52E-165	12
Etv6	4.14E-169	0.27245469	0.309	0.049	1.34E-164	12
Arhgef6	9.39E-169	0.30039454	0.29	0.044	3.03E-164	12
Ndufs2	1.23E-168	0.29807161	0.473	0.101	3.97E-164	12
Cks22	1.33E-168	0.94749155	0.764	0.246	4.29E-164	12
Dock101	2.26E-168	0.34683711	0.325	0.054	7.29E-164	12
Emc8	2.42E-168	0.37884455	0.368	0.067	7.80E-164	12
Pigyl	2.63E-168	0.28197028	0.33	0.056	8.50E-164	12
Ccr21	5.35E-168	0.35859263	0.326	0.054	1.73E-163	12
Psmd14	8.38E-168	0.31020608	0.475	0.102	2.70E-163	12
Emc10	1.18E-167	0.28642906	0.384	0.072	3.81E-163	12
Blmh	1.23E-167	0.29365753	0.281	0.042	3.98E-163	12
Fundc21	1.75E-167	0.3633888	0.544	0.127	5.65E-163	12
Mrpl48	2.12E-167	0.3539314	0.383	0.072	6.85E-163	12
Naa20	4.19E-167	0.32500518	0.376	0.07	1.35E-162	12
Nop582	5.86E-167	0.52784358	0.575	0.14	1.89E-162	12
Gpx13	7.59E-167	1.04712021	0.913	0.509	2.45E-162	12
Tmed2	8.94E-167	0.56383576	0.713	0.199	2.89E-162	12
Uqcr114	9.86E-167	1.17485398	0.836	0.349	3.18E-162	12
Rnf1302	1.01E-166	0.43236121	0.531	0.124	3.26E-162	12
Ppp2cb	1.20E-166	0.3272117	0.46	0.098	3.86E-162	12
Pdhb1	2.06E-166	0.26132599	0.421	0.084	6.65E-162	12
Pnn1	3.04E-166	0.31891169	0.456	0.096	9.80E-162	12
Hist1h2ap2	6.27E-166	0.63824061	0.734	0.239	2.03E-161	12
Mrps333	6.98E-166	0.52860236	0.675	0.184	2.25E-161	12
Pcbd22	8.96E-166	0.32611233	0.47	0.102	2.89E-161	12
Rpl36al6	1.12E-165	1.15851085	0.913	0.504	3.61E-161	12
Tmem179b2	1.29E-165	0.3236429	0.493	0.11	4.15E-161	12
Abracl3	1.40E-165	0.87136588	0.796	0.267	4.52E-161	12
Psmc2	2.08E-165	0.27152147	0.415	0.083	6.72E-161	12
Tex301	2.30E-165	0.30800902	0.335	0.058	7.41E-161	12

Slbp1	4.03E-165	0.45763447	0.557	0.135	1.30E-160	12
Idh3g	5.40E-165	0.35499341	0.419	0.084	1.74E-160	12
Nudt211	6.58E-165	0.44472847	0.563	0.136	2.13E-160	12
2300009A05I	1.01E-164	0.25129274	0.253	0.035	3.28E-160	12
Lsm82	2.72E-164	0.35508883	0.552	0.132	8.79E-160	12
Ybx31	9.87E-164	0.45983681	0.667	0.175	3.19E-159	12
Cenpm1	1.16E-163	0.37173883	0.275	0.041	3.76E-159	12
Ilf2	1.18E-163	0.27362429	0.381	0.072	3.82E-159	12
Lsm12	1.33E-163	0.29201965	0.492	0.11	4.30E-159	12
Pfdn22	1.38E-163	0.38386973	0.603	0.152	4.44E-159	12
Mrps361	3.00E-163	0.29685518	0.518	0.119	9.67E-159	12
Rheb1	5.17E-163	0.41704495	0.524	0.122	1.67E-158	12
Prim1	7.81E-163	0.31112613	0.268	0.04	2.52E-158	12
Snrpa	1.30E-162	0.26322904	0.389	0.075	4.20E-158	12
Xbp11	3.31E-162	0.26150895	0.485	0.108	1.07E-157	12
Golt1b	5.40E-162	0.25862351	0.271	0.04	1.74E-157	12
Aamp	8.27E-162	0.36439036	0.515	0.12	2.67E-157	12
Vdac2	1.06E-161	0.72550415	0.719	0.212	3.43E-157	12
Jtb	1.60E-161	0.28781795	0.383	0.074	5.16E-157	12
Psmc41	2.49E-161	0.39893241	0.419	0.086	8.04E-157	12
Apip	4.53E-161	0.2630695	0.285	0.045	1.46E-156	12
Myb3	8.26E-161	0.37085497	0.472	0.105	2.67E-156	12
Atraid	1.44E-160	0.2950013	0.313	0.053	4.66E-156	12
Atpif13	2.67E-160	1.04707338	0.869	0.414	8.62E-156	12
Ppp1r35	4.69E-160	0.33186748	0.381	0.074	1.52E-155	12
Mrpl24	4.83E-160	0.28962535	0.47	0.104	1.56E-155	12
Cdca31	5.28E-160	0.37726488	0.389	0.077	1.71E-155	12
H2afx1	6.00E-160	0.40215822	0.536	0.13	1.94E-155	12
Rwdd1	7.70E-160	0.32055125	0.557	0.135	2.49E-155	12
Rps296	1.02E-159	0.83439846	0.996	0.954	3.30E-155	12
Gm472833	1.81E-159	0.46311923	0.603	0.156	5.86E-155	12
Ube2f	3.17E-159	0.25803388	0.348	0.063	1.02E-154	12
Lsm42	3.79E-159	0.58567048	0.732	0.214	1.22E-154	12
Csgalnact2	4.01E-159	0.26843832	0.272	0.041	1.30E-154	12
Pcna2	6.95E-159	0.52024435	0.655	0.178	2.24E-154	12
Fxn1	1.99E-158	0.27415462	0.279	0.044	6.44E-154	12
Nudt3	3.60E-158	0.25573936	0.351	0.065	1.16E-153	12
Cisd22	6.69E-158	0.30578569	0.54	0.13	2.16E-153	12
Med8	9.26E-158	0.28144599	0.349	0.064	2.99E-153	12
Mrpl32	1.20E-157	0.30081452	0.456	0.1	3.86E-153	12
Ddx39b2	1.30E-157	0.4186467	0.638	0.169	4.20E-153	12
G3bp1	1.56E-157	0.30869159	0.56	0.137	5.05E-153	12
Adgrg3	3.24E-157	0.41679922	0.278	0.044	1.05E-152	12

Cdc37	5.35E-157	0.27697718	0.555	0.135	1.73E-152	12
Srp93	1.19E-156	0.76753276	0.773	0.25	3.84E-152	12
Snapc5	1.36E-156	0.30228859	0.392	0.079	4.39E-152	12
Atp5o4	1.66E-156	0.71116535	0.75	0.234	5.37E-152	12
Timm17b1	1.81E-156	0.41235154	0.463	0.104	5.83E-152	12
Pttg11	2.29E-156	0.46164256	0.527	0.128	7.40E-152	12
Cyc11	3.30E-156	0.45695891	0.572	0.144	1.07E-151	12
Anp32b2	4.17E-156	0.85554989	0.825	0.279	1.35E-151	12
Rab9	4.39E-156	0.29246663	0.271	0.042	1.42E-151	12
Mcrip1	4.87E-156	0.25833684	0.392	0.078	1.57E-151	12
Yrdc	4.92E-156	0.25198487	0.282	0.045	1.59E-151	12
Rps195	1.63E-155	0.97284143	0.985	0.727	5.27E-151	12
Supt162	1.79E-155	0.47813421	0.601	0.158	5.78E-151	12
Ndufb103	2.93E-155	0.69750898	0.745	0.232	9.47E-151	12
Sf3a3	3.31E-155	0.26910965	0.314	0.054	1.07E-150	12
Fkbp33	3.49E-155	0.44567584	0.606	0.157	1.13E-150	12
Ube2m1	3.63E-155	0.40289068	0.606	0.159	1.17E-150	12
Mrpl431	4.35E-155	0.28278322	0.485	0.111	1.40E-150	12
Rbis2	1.25E-154	0.29873624	0.614	0.159	4.02E-150	12
Cited22	1.72E-154	0.38055039	0.473	0.109	5.57E-150	12
Emb3	1.75E-154	0.54761298	0.557	0.144	5.66E-150	12
Ndufb43	4.61E-154	0.64482957	0.738	0.228	1.49E-149	12
Bcap31	4.79E-154	0.45607216	0.571	0.145	1.54E-149	12
Thoc72	5.14E-154	0.51395914	0.668	0.187	1.66E-149	12
Ndufv33	7.21E-154	0.74302421	0.744	0.241	2.33E-149	12
Nme23	7.31E-154	1.07986644	0.902	0.399	2.36E-149	12
Ube2n1	9.64E-154	0.41690444	0.523	0.127	3.11E-149	12
Dpm33	1.33E-153	0.64125995	0.763	0.242	4.31E-149	12
Bcas21	4.09E-153	0.33016144	0.544	0.133	1.32E-148	12
Pih1d1	7.21E-153	0.29449815	0.297	0.05	2.33E-148	12
Rpl314	9.64E-153	1.18159759	0.967	0.551	3.11E-148	12
Brk11	1.64E-152	0.40607282	0.661	0.184	5.29E-148	12
Bub31	2.25E-152	0.35674527	0.493	0.116	7.27E-148	12
Rnpep	2.68E-152	0.30861338	0.33	0.06	8.66E-148	12
Rplp04	3.44E-152	1.04255535	0.993	0.734	1.11E-147	12
Tbcb	5.38E-152	0.34049486	0.523	0.127	1.74E-147	12
Psmb93	1.47E-151	0.46300924	0.581	0.152	4.74E-147	12
Sf3b63	2.27E-151	0.72318769	0.774	0.252	7.33E-147	12
Chrac1	2.37E-151	0.30207109	0.464	0.105	7.66E-147	12
Cops3	2.98E-151	0.36559868	0.32	0.058	9.62E-147	12
2310009A05	4.61E-151	0.32437446	0.48	0.112	1.49E-146	12
Ten1	1.14E-150	0.26646659	0.378	0.076	3.69E-146	12
Ech1	1.32E-150	0.34761845	0.45	0.101	4.26E-146	12

Cct71	2.20E-150	0.36511098	0.598	0.156	7.11E-146	12
Rbbp71	2.26E-150	0.35749181	0.528	0.129	7.31E-146	12
Rpl295	2.39E-150	1.05267824	0.933	0.568	7.71E-146	12
P4hb2	9.60E-150	0.35913663	0.627	0.171	3.10E-145	12
Tubb4b1	1.24E-149	0.72663463	0.616	0.173	4.01E-145	12
Psma35	1.96E-149	0.68515499	0.758	0.243	6.32E-145	12
Anapc133	2.75E-149	0.40287306	0.67	0.19	8.88E-145	12
Snrpd34	1.56E-148	0.77318903	0.769	0.255	5.03E-144	12
Aurkb	2.33E-148	0.34553384	0.277	0.045	7.51E-144	12
Pigx	2.96E-148	0.30598951	0.349	0.067	9.54E-144	12
Ppm1g1	3.89E-148	0.2851986	0.409	0.087	1.26E-143	12
Hnrnpd2	4.97E-148	0.44794726	0.661	0.185	1.60E-143	12
Srp192	6.22E-148	0.43203373	0.584	0.155	2.01E-143	12
Snhg4	8.50E-148	0.25476861	0.262	0.041	2.74E-143	12
Rpl10a6	1.04E-147	1.06973077	0.969	0.598	3.35E-143	12
Cfap20	1.06E-147	0.33189148	0.293	0.05	3.42E-143	12
Snrpb5	1.22E-147	0.72291401	0.806	0.265	3.93E-143	12
Atp5c14	1.51E-147	0.74984507	0.79	0.266	4.88E-143	12
Ndufs43	1.57E-147	0.43019668	0.624	0.172	5.08E-143	12
Swi53	2.08E-147	0.62063105	0.747	0.235	6.72E-143	12
Ptma4	2.11E-147	0.88418172	0.994	0.685	6.80E-143	12
Eif6	2.55E-147	0.32467662	0.553	0.14	8.23E-143	12
Eif1ax2	2.58E-147	0.46864921	0.659	0.185	8.34E-143	12
Rpl115	3.42E-147	0.95839026	0.985	0.736	1.10E-142	12
Rpl95	3.42E-147	0.93021145	0.974	0.77	1.11E-142	12
Pfdn61	1.01E-146	0.25724185	0.543	0.137	3.27E-142	12
Cisd12	1.33E-146	0.32544706	0.402	0.085	4.30E-142	12
Polr2i3	2.25E-146	0.28891623	0.531	0.133	7.25E-142	12
Rae1	2.49E-146	0.28481369	0.291	0.05	8.04E-142	12
Cebpe2	7.44E-146	0.71275813	0.338	0.067	2.40E-141	12
Psmd131	1.71E-145	0.29931352	0.434	0.097	5.51E-141	12
Srsf102	1.79E-145	0.25486108	0.572	0.147	5.77E-141	12
Ube2a3	5.72E-145	0.36389581	0.624	0.172	1.85E-140	12
Rps36	7.49E-145	0.98335898	0.975	0.703	2.42E-140	12
Tmed101	7.98E-145	0.56463231	0.705	0.212	2.58E-140	12
Strap	8.87E-145	0.27280632	0.421	0.092	2.87E-140	12
Wdr761	1.06E-144	0.25894413	0.281	0.047	3.41E-140	12
Mphosph6	3.80E-144	0.28665693	0.279	0.047	1.23E-139	12
Vdac32	4.28E-144	0.27704282	0.585	0.154	1.38E-139	12
Rbbp42	8.11E-144	0.37992007	0.598	0.162	2.62E-139	12
Spi15	8.75E-144	0.51035857	0.694	0.208	2.82E-139	12
Cox5b4	2.28E-143	1.05164821	0.875	0.465	7.37E-139	12
Ube2j2	2.43E-143	0.36793791	0.348	0.069	7.85E-139	12

Lamtor11	2.68E-143	0.44448541	0.459	0.108	8.66E-139	12
Iqgap2	5.33E-143	0.36061371	0.277	0.047	1.72E-138	12
Cox142	5.84E-143	0.31065324	0.607	0.165	1.89E-138	12
Fam162a	1.27E-142	0.26014091	0.445	0.102	4.10E-138	12
Timm8b	1.41E-142	0.31189396	0.562	0.147	4.54E-138	12
Ap2s13	1.71E-142	0.5213851	0.636	0.183	5.53E-138	12
Rpl22l15	3.53E-142	1.05409054	0.911	0.514	1.14E-137	12
Lamtor44	4.78E-142	0.86335936	0.763	0.268	1.54E-137	12
Ggh1	8.74E-142	0.27901654	0.295	0.052	2.82E-137	12
Dpm2	1.60E-141	0.28862349	0.319	0.06	5.16E-137	12
Med281	4.73E-141	0.29484215	0.435	0.1	1.53E-136	12
Gtf3a	6.92E-141	0.31495101	0.338	0.066	2.23E-136	12
Mplkip	1.26E-140	0.25824878	0.357	0.072	4.05E-136	12
Gtf2h52	1.34E-140	0.31872009	0.667	0.191	4.34E-136	12
Cct4	2.40E-140	0.27867659	0.499	0.122	7.74E-136	12
Reep52	3.62E-140	0.72434902	0.761	0.258	1.17E-135	12
Wdr83os2	5.84E-140	0.35700881	0.588	0.16	1.89E-135	12
Ube2l33	6.10E-140	0.36588972	0.671	0.196	1.97E-135	12
Trmt1124	6.96E-140	0.57582387	0.735	0.231	2.25E-135	12
Prdx42	7.38E-140	0.42574502	0.3	0.055	2.38E-135	12
Snrpf4	1.00E-139	1.00246001	0.859	0.378	3.23E-135	12
Mdh21	1.00E-139	0.57380179	0.721	0.222	3.24E-135	12
Rilpl21	1.13E-139	0.28007995	0.355	0.072	3.64E-135	12
Hint14	1.27E-139	1.04524561	0.866	0.397	4.09E-135	12
Ndufb86	1.34E-139	0.79453226	0.789	0.283	4.33E-135	12
Spcs1	7.36E-139	0.40793597	0.664	0.194	2.38E-134	12
Rps4x6	1.54E-138	1.00306743	0.985	0.689	4.98E-134	12
Sumo12	1.99E-138	0.4425034	0.702	0.21	6.43E-134	12
Gtf2a21	3.58E-138	0.29936018	0.525	0.135	1.16E-133	12
Srsf23	4.90E-138	0.63300373	0.76	0.248	1.58E-133	12
Hacd21	2.40E-137	0.25115704	0.322	0.062	7.76E-133	12
Micos104	5.54E-137	0.66277783	0.764	0.259	1.79E-132	12
Dnajc21	6.75E-137	0.27954924	0.444	0.104	2.18E-132	12
Rpl143	9.83E-137	1.01040765	0.942	0.518	3.17E-132	12
Psmd83	1.05E-136	0.36426568	0.642	0.183	3.39E-132	12
Tmem1672	1.34E-136	0.57219315	0.703	0.219	4.33E-132	12
Rps6ka11	4.08E-136	0.28343289	0.32	0.062	1.32E-131	12
Sap183	5.29E-136	0.50776621	0.696	0.221	1.71E-131	12
Cox7b5	7.26E-136	0.85909369	0.831	0.333	2.34E-131	12
Selenof1	1.14E-135	0.65050988	0.732	0.238	3.69E-131	12
Ptpn183	4.32E-135	0.64307897	0.79	0.282	1.40E-130	12
Vps29	6.79E-135	0.28850346	0.512	0.132	2.19E-130	12
Snrpg5	7.36E-135	1.01822671	0.894	0.499	2.38E-130	12

Aprt3	7.53E-135	0.73428277	0.782	0.279	2.43E-130	12
Tacc31	1.08E-134	0.29341695	0.295	0.055	3.50E-130	12
Znrd12	1.29E-134	0.252537	0.456	0.109	4.17E-130	12
Rps205	1.39E-134	0.88432948	0.993	0.739	4.47E-130	12
Cnih41	3.07E-134	0.2559642	0.597	0.166	9.92E-130	12
Snrpd23	4.14E-133	0.76451513	0.802	0.289	1.34E-128	12
Ddx21	1.18E-132	0.26327893	0.537	0.142	3.80E-128	12
Rps56	2.27E-132	0.95029353	0.981	0.677	7.34E-128	12
Guk1	6.36E-132	0.26400855	0.328	0.066	2.05E-127	12
Ccnh	6.38E-132	0.25140256	0.262	0.045	2.06E-127	12
Sap301	1.61E-131	0.27072719	0.358	0.076	5.21E-127	12
Hspe11	1.83E-131	0.87790335	0.748	0.269	5.92E-127	12
Psmb62	3.23E-131	0.5101332	0.7	0.221	1.04E-126	12
Calr2	7.94E-131	0.75886964	0.699	0.233	2.56E-126	12
Uqcr103	8.27E-131	0.94110383	0.852	0.38	2.67E-126	12
Ndufs65	1.09E-130	0.51792567	0.748	0.247	3.50E-126	12
Tspo1	2.51E-130	1.06707414	0.868	0.449	8.09E-126	12
Nucks12	3.32E-130	0.45191282	0.722	0.224	1.07E-125	12
Rpl155	4.10E-130	0.9532442	0.971	0.656	1.32E-125	12
Set2	5.89E-130	0.89069804	0.844	0.339	1.90E-125	12
Psmb42	1.14E-129	0.59511739	0.742	0.244	3.67E-125	12
Rps116	1.18E-129	0.87644995	0.977	0.725	3.80E-125	12
Pdcd43	2.01E-129	0.69326987	0.689	0.232	6.48E-125	12
Sec61g4	2.23E-129	0.92667657	0.918	0.605	7.21E-125	12
Rrp1	2.45E-129	0.30617817	0.381	0.085	7.90E-125	12
Mrfap13	3.04E-129	0.38366311	0.623	0.182	9.81E-125	12
Atp5d4	4.21E-129	0.6548818	0.766	0.261	1.36E-124	12
Sarnp3	4.39E-129	0.48764119	0.732	0.236	1.42E-124	12
Mgst23	6.96E-129	0.54800784	0.476	0.126	2.25E-124	12
Atp5g25	1.58E-128	0.98404797	0.876	0.405	5.12E-124	12
Magoh5	7.39E-128	0.63001394	0.745	0.253	2.39E-123	12
Tagln23	8.65E-128	0.71006851	0.808	0.295	2.79E-123	12
Dek3	9.18E-128	0.69818491	0.808	0.297	2.97E-123	12
Iscu3	1.16E-127	0.25609468	0.486	0.125	3.75E-123	12
Bag1	1.34E-127	0.27958749	0.553	0.153	4.34E-123	12
Drap11	2.68E-127	0.31656573	0.63	0.185	8.64E-123	12
Cmpk12	1.06E-126	0.36156652	0.543	0.15	3.41E-122	12
Atp6v0b4	1.09E-126	0.59595918	0.777	0.272	3.51E-122	12
Nmt13	1.15E-126	0.31024161	0.654	0.197	3.70E-122	12
Anxa31	1.16E-126	0.29588645	0.588	0.166	3.75E-122	12
Cdkn32	1.86E-126	0.34640994	0.304	0.061	6.00E-122	12
Pcbp13	5.86E-126	0.45164983	0.734	0.238	1.89E-121	12
Vapa1	8.19E-126	0.48724888	0.697	0.223	2.64E-121	12

Akr1a12	2.46E-125	0.29010085	0.587	0.167	7.96E-121	12
Gapdh5	5.28E-125	0.93822226	0.901	0.538	1.70E-120	12
Rab5if4	5.42E-125	0.67288388	0.738	0.258	1.75E-120	12
Rps62	7.25E-125	0.99660525	0.921	0.492	2.34E-120	12
Hist1h1b1	1.78E-124	0.6325109	0.45	0.12	5.73E-120	12
Pdcd53	3.01E-124	0.34992149	0.68	0.211	9.70E-120	12
Dcun1d52	5.27E-124	0.28516068	0.619	0.18	1.70E-119	12
Npc23	5.55E-124	0.38466774	0.732	0.239	1.79E-119	12
Ndufa114	8.25E-124	0.45472139	0.748	0.249	2.66E-119	12
Sdhb3	1.27E-123	0.48010046	0.569	0.166	4.10E-119	12
H2afj2	1.35E-123	0.93245178	0.854	0.423	4.35E-119	12
Sumo23	1.83E-123	0.81474499	0.863	0.338	5.92E-119	12
Banf12	2.73E-123	0.2593423	0.635	0.188	8.83E-119	12
Tomm73	2.73E-122	0.94703217	0.878	0.475	8.82E-118	12
Pak1ip1	8.54E-122	0.28662542	0.355	0.079	2.76E-117	12
Atp5f14	1.05E-121	0.77842868	0.809	0.309	3.40E-117	12
Ccnb21	1.94E-121	0.30869458	0.376	0.087	6.28E-117	12
H2afv3	7.20E-121	0.29991997	0.792	0.273	2.32E-116	12
Snhg12	5.69E-120	0.30100599	0.648	0.199	1.84E-115	12
Cox8a4	6.71E-120	0.8761389	0.9	0.681	2.17E-115	12
Rps234	3.16E-119	0.83986779	0.974	0.759	1.02E-114	12
Ccdc1241	5.00E-119	0.25289258	0.402	0.097	1.61E-114	12
Ndufs53	6.45E-119	0.52104411	0.766	0.275	2.08E-114	12
Psma24	6.57E-119	0.69106741	0.814	0.306	2.12E-114	12
Rpl286	8.03E-118	0.8400502	0.965	0.756	2.59E-113	12
Rtraf3	8.12E-118	0.35844631	0.661	0.205	2.62E-113	12
Psmb34	8.50E-118	0.64497455	0.77	0.285	2.75E-113	12
Sec11c3	2.03E-117	0.54017565	0.796	0.3	6.55E-113	12
Mif2	5.39E-117	0.85853847	0.808	0.33	1.74E-112	12
Rps15a5	6.23E-117	0.78401189	0.985	0.782	2.01E-112	12
Pdia32	1.41E-116	0.3258563	0.734	0.246	4.56E-112	12
Tecr2	3.01E-116	0.51753392	0.671	0.223	9.73E-112	12
Uqcc21	5.03E-116	0.33909269	0.664	0.209	1.62E-111	12
Tomm223	1.57E-115	0.39274364	0.661	0.21	5.06E-111	12
Ost43	2.83E-115	0.73712327	0.814	0.338	9.14E-111	12
Naca5	3.70E-115	0.88409046	0.934	0.566	1.20E-110	12
Eif4a12	7.37E-115	0.7940357	0.833	0.347	2.38E-110	12
Rgs183	1.21E-114	0.29132064	0.448	0.117	3.92E-110	12
Tomm203	1.81E-114	0.51615193	0.732	0.248	5.85E-110	12
Atp5mpl4	1.86E-114	0.8190695	0.82	0.365	6.00E-110	12
Calm32	2.86E-114	0.44664336	0.723	0.245	9.25E-110	12
Rpl396	6.53E-114	0.78004267	0.994	0.844	2.11E-109	12
Smdt15	1.80E-113	0.71969855	0.796	0.329	5.81E-109	12

Cbx32	3.03E-113	0.69428532	0.83	0.329	9.78E-109	12
Ncf44	4.66E-113	0.43533215	0.544	0.162	1.50E-108	12
Rpl186	1.23E-112	0.80832426	0.971	0.709	3.97E-108	12
Trem33	2.14E-112	0.52872709	0.469	0.131	6.91E-108	12
Adh5	5.56E-112	0.30753526	0.316	0.069	1.79E-107	12
Uqcrq3	9.10E-112	0.83989056	0.838	0.401	2.94E-107	12
Atp5h3	1.65E-111	0.86425206	0.866	0.467	5.31E-107	12
Emp33	2.46E-111	0.73079617	0.792	0.312	7.95E-107	12
Rpl134	3.07E-111	0.7810927	0.999	0.789	9.91E-107	12
Ndufb114	3.42E-111	0.63592272	0.796	0.313	1.11E-106	12
Uhrf11	6.50E-111	0.25055766	0.349	0.082	2.10E-106	12
Eif3f5	3.59E-110	0.49992945	0.773	0.278	1.16E-105	12
Eif3k4	4.98E-110	0.61461323	0.792	0.299	1.61E-105	12
Clta6	7.32E-110	0.62839294	0.795	0.309	2.36E-105	12
Hmgb24	1.62E-109	0.66981313	0.961	0.634	5.22E-105	12
Cops92	6.12E-109	0.43184417	0.738	0.264	1.98E-104	12
Cenpa2	4.03E-108	0.56479636	0.461	0.133	1.30E-103	12
Rpl416	7.50E-108	0.71332333	0.994	0.9	2.42E-103	12
Hnrnpa14	1.65E-107	0.51032015	0.748	0.265	5.34E-103	12
Prdx65	2.14E-107	0.51303603	0.758	0.273	6.90E-103	12
1810037I17R	3.10E-107	0.81319616	0.799	0.339	1.00E-102	12
Zfp7064	3.58E-107	0.44615036	0.761	0.273	1.16E-102	12
Rpl106	6.10E-107	0.84661342	0.904	0.598	1.97E-102	12
Atp6v0c3	7.77E-107	0.76964751	0.843	0.387	2.51E-102	12
Asnsd1	1.21E-106	0.31240141	0.492	0.142	3.92E-102	12
Tmem2585	3.74E-106	0.75330567	0.827	0.376	1.21E-101	12
Arpc47	4.91E-106	0.42020413	0.716	0.255	1.58E-101	12
Copz1	1.28E-105	0.25651174	0.346	0.083	4.12E-101	12
Rps76	2.95E-105	0.76307325	0.971	0.726	9.53E-101	12
Rpl36a5	6.62E-105	0.8342219	0.937	0.577	2.14E-100	12
Prelid13	1.19E-103	0.40886962	0.716	0.251	3.85E-99	12
Arl6ip13	1.21E-103	0.39656287	0.608	0.201	3.91E-99	12
Ndufc14	4.49E-103	0.577976	0.793	0.314	1.45E-98	12
Atp6v0e2	4.75E-103	0.55437247	0.761	0.292	1.53E-98	12
Adk	5.21E-103	0.29154959	0.278	0.06	1.68E-98	12
Pgls3	7.61E-103	0.45899127	0.834	0.333	2.46E-98	12
Gm100766	8.21E-103	0.75358302	0.967	0.807	2.65E-98	12
Tma73	1.01E-102	0.69303527	0.833	0.378	3.27E-98	12
Gpi14	3.32E-102	0.6094849	0.729	0.276	1.07E-97	12
Ppp1cc2	3.99E-102	0.28761532	0.549	0.168	1.29E-97	12
Tubb54	5.62E-102	0.69144035	0.834	0.35	1.82E-97	12
Metap22	4.66E-101	0.28738017	0.684	0.233	1.51E-96	12
Polr1d5	5.88E-101	0.50566141	0.774	0.289	1.90E-96	12

Prpf38a	6.80E-101	0.26574658	0.253	0.052	2.19E-96	12
Rplp25	1.98E-100	0.76970205	0.962	0.711	6.40E-96	12
Tle51	2.10E-100	0.30654978	0.69	0.236	6.77E-96	12
Rpl7a5	2.47E-100	0.84905355	0.93	0.552	7.99E-96	12
Timm10b4	3.06E-100	0.3595497	0.707	0.251	9.89E-96	12
Hnrnpf3	5.16E-99	0.49968178	0.806	0.309	1.66E-94	12
Snu133	1.17E-98	0.36630523	0.729	0.261	3.77E-94	12
Cst32	1.34E-98	0.66797841	0.872	0.466	4.33E-94	12
Hspd11	3.28E-98	0.29575327	0.636	0.211	1.06E-93	12
Scp23	1.21E-97	0.38404739	0.632	0.215	3.91E-93	12
Rpl276	3.44E-97	0.74136969	0.952	0.721	1.11E-92	12
Agpat21	1.62E-96	0.28623091	0.277	0.062	5.24E-92	12
Rpl195	8.28E-96	0.71687964	0.984	0.757	2.67E-91	12
Rpl37a6	2.09E-95	0.63122822	0.994	0.903	6.75E-91	12
Ndufa17	5.79E-95	0.6389921	0.805	0.348	1.87E-90	12
Arhgdib4	4.43E-94	0.84490596	0.881	0.426	1.43E-89	12
Rpl227	1.03E-93	0.75374793	0.961	0.661	3.33E-89	12
Edf14	1.38E-93	0.47039207	0.771	0.301	4.46E-89	12
Tomm64	1.60E-93	0.70361219	0.834	0.41	5.17E-89	12
Atp5b2	3.57E-93	0.46623184	0.786	0.306	1.15E-88	12
Ppp4c1	4.36E-93	0.25068038	0.415	0.118	1.41E-88	12
Hnrnpab2	4.55E-93	0.36097108	0.712	0.257	1.47E-88	12
Ppib2	6.45E-93	0.5498214	0.79	0.325	2.08E-88	12
Vamp82	8.95E-93	0.32865744	0.723	0.269	2.89E-88	12
Atp5j4	1.01E-92	0.71890531	0.828	0.406	3.26E-88	12
Lamp13	1.33E-92	0.26645149	0.646	0.226	4.28E-88	12
Nedd84	1.71E-92	0.5575433	0.811	0.344	5.51E-88	12
Rpl366	2.77E-91	0.73490465	0.975	0.758	8.95E-87	12
Tkt4	3.95E-91	0.50015418	0.748	0.288	1.27E-86	12
Cnbp1	5.59E-91	0.25146652	0.619	0.207	1.80E-86	12
Ube2s1	1.67E-90	0.43719238	0.766	0.295	5.38E-86	12
Arf13	2.03E-90	0.32824618	0.739	0.274	6.57E-86	12
Eif3h5	2.52E-90	0.29864414	0.728	0.266	8.13E-86	12
Rab2a	7.15E-90	0.25001132	0.616	0.21	2.31E-85	12
Psma72	1.52E-89	0.64483814	0.83	0.371	4.92E-85	12
Ybx13	1.56E-89	0.64547502	0.895	0.439	5.05E-85	12
Atp5e6	2.67E-89	0.64685141	0.91	0.693	8.61E-85	12
Psme15	5.99E-89	0.38448957	0.764	0.298	1.93E-84	12
Ltb4r12	1.62E-88	0.39730571	0.383	0.109	5.23E-84	12
Eno12	3.12E-88	0.29556374	0.706	0.262	1.01E-83	12
Ube2i3	1.22E-87	0.30981528	0.664	0.235	3.94E-83	12
Anp32a2	1.23E-87	0.33157415	0.721	0.272	3.97E-83	12
Rpl236	1.63E-87	0.65852872	0.985	0.822	5.25E-83	12

Rpl386	3.05E-87	0.64870885	0.993	0.84	9.84E-83	12
Atp5a14	3.88E-87	0.33438405	0.747	0.282	1.25E-82	12
Cox6b14	4.21E-87	0.76314546	0.856	0.49	1.36E-82	12
Rpl35a6	7.77E-87	0.62664236	0.978	0.854	2.51E-82	12
Rps167	1.48E-86	0.66292644	0.969	0.775	4.79E-82	12
Btf36	4.02E-86	0.77308139	0.924	0.541	1.30E-81	12
Sem15	1.13E-85	0.69574551	0.886	0.664	3.66E-81	12
Psmb15	1.85E-85	0.39423367	0.758	0.293	5.97E-81	12
Rpl264	4.41E-85	0.70605815	0.952	0.699	1.42E-80	12
Romo12	8.96E-85	0.45707156	0.803	0.339	2.89E-80	12
Hist1h4d1	1.12E-84	0.28199716	0.355	0.1	3.63E-80	12
Rps286	9.68E-84	0.64334381	0.997	0.843	3.13E-79	12
Hp5	1.77E-83	0.37627165	0.76	0.296	5.70E-79	12
Slc25a31	4.98E-83	0.6498678	0.837	0.381	1.61E-78	12
Cox6a13	2.21E-82	0.33498322	0.769	0.307	7.13E-78	12
Mrpl333	2.90E-81	0.62340289	0.833	0.445	9.36E-77	12
Atp5k5	4.00E-81	0.6874342	0.886	0.501	1.29E-76	12
Snrpe5	6.07E-81	0.63201557	0.854	0.419	1.96E-76	12
Rbx14	1.53E-80	0.58769974	0.805	0.365	4.92E-76	12
Arpp193	2.60E-80	0.43527847	0.779	0.331	8.38E-76	12
Atp5l4	4.18E-80	0.69303141	0.889	0.603	1.35E-75	12
Pomp5	9.23E-79	0.4998751	0.776	0.336	2.98E-74	12
Srsf33	9.29E-79	0.55926095	0.853	0.385	3.00E-74	12
Sh3bgrl37	1.59E-78	0.74746997	0.892	0.564	5.13E-74	12
Ndufb74	1.89E-78	0.54581225	0.812	0.371	6.09E-74	12
Cox7c6	2.35E-78	0.62316843	0.923	0.667	7.59E-74	12
Rps172	3.27E-77	0.6216681	0.916	0.556	1.05E-72	12
Itm2b4	2.78E-76	0.73819218	0.892	0.529	8.96E-72	12
Rps266	4.11E-76	0.6340443	0.977	0.785	1.33E-71	12
Rps3a15	1.18E-75	0.66017027	0.991	0.771	3.80E-71	12
Rpl43	2.97E-75	0.5284607	0.868	0.407	9.59E-71	12
Eef1b22	8.40E-74	0.65555705	0.916	0.528	2.71E-69	12
Chchd24	1.07E-73	0.6497524	0.885	0.575	3.45E-69	12
Hmgb14	1.55E-73	0.41142311	0.914	0.53	5.00E-69	12
Ndufb93	2.69E-73	0.48180216	0.786	0.352	8.69E-69	12
Ndufa33	3.07E-73	0.53713416	0.811	0.4	9.91E-69	12
Rpl18a6	6.70E-73	0.58318457	0.983	0.804	2.16E-68	12
Hsp90b12	1.92E-72	0.3719806	0.818	0.369	6.19E-68	12
Cox7a24	9.34E-72	0.6275557	0.841	0.498	3.02E-67	12
Rpl75	1.99E-71	0.64891554	0.951	0.627	6.43E-67	12
Rpl27a5	6.50E-71	0.59238155	0.987	0.797	2.10E-66	12
Sub15	1.40E-70	0.56724238	0.854	0.472	4.50E-66	12
Rbm35	3.08E-70	0.60247088	0.852	0.418	9.95E-66	12

Ndufb1-ps5	1.61E-69	0.55389258	0.905	0.632	5.18E-65	12
Serf21	3.10E-69	0.53378368	0.902	0.725	1.00E-64	12
Cox6c5	2.41E-68	0.5919695	0.894	0.633	7.79E-64	12
Atp6v1f5	3.95E-68	0.28467901	0.75	0.314	1.28E-63	12
Oaz16	1.72E-67	0.58829888	0.929	0.634	5.55E-63	12
Prdx15	2.81E-67	0.53860846	0.817	0.385	9.06E-63	12
Mrps213	5.82E-67	0.25504363	0.744	0.312	1.88E-62	12
Rps135	2.12E-66	0.534094	0.948	0.794	6.84E-62	12
Atp5j24	2.37E-66	0.59604045	0.869	0.535	7.65E-62	12
Clec4a24	4.47E-66	0.48557208	0.447	0.166	1.44E-61	12
Hsp90aa12	5.92E-66	0.28411184	0.817	0.36	1.91E-61	12
Taldo14	8.83E-66	0.42241269	0.821	0.384	2.85E-61	12
Rps246	3.13E-65	0.4932128	0.996	0.853	1.01E-60	12
Srgn2	5.66E-65	0.43561112	0.908	0.503	1.83E-60	12
Actr23	1.11E-64	0.25150418	0.732	0.304	3.57E-60	12
Ncl2	1.45E-64	0.53755349	0.815	0.407	4.67E-60	12
Rpl356	1.93E-63	0.59079712	0.969	0.771	6.23E-59	12
Npm11	7.12E-63	0.4706948	0.891	0.462	2.30E-58	12
Serbp13	1.16E-62	0.56057046	0.878	0.479	3.75E-58	12
Rpl306	2.69E-62	0.51470436	0.977	0.815	8.67E-58	12
Aldoa3	6.05E-62	0.42101707	0.831	0.411	1.95E-57	12
Rpl346	1.16E-61	0.51726061	0.972	0.824	3.73E-57	12
Uba525	1.19E-61	0.61018138	0.866	0.757	3.85E-57	12
Rps106	1.64E-60	0.52644975	0.965	0.801	5.30E-56	12
Atox12	2.14E-59	0.29153618	0.802	0.384	6.91E-55	12
Arpc1b3	1.65E-58	0.50326905	0.847	0.43	5.31E-54	12
Rps82	8.19E-58	0.47602974	0.978	0.743	2.64E-53	12
Myl12b4	4.35E-57	0.46346011	0.833	0.414	1.40E-52	12
Gmfg7	2.77E-55	0.28736929	0.824	0.395	8.94E-51	12
Rpl63	1.36E-52	0.45787046	0.985	0.754	4.40E-48	12
Gpx42	7.59E-52	0.29715412	0.792	0.39	2.45E-47	12
mt-Nd4l4	4.48E-50	0.26447041	0.812	0.441	1.45E-45	12
Rpl315	4.12E-47	0.41069535	0.876	0.537	1.33E-42	12
Rpl375	8.54E-46	0.41216862	0.988	0.914	2.76E-41	12
Rpl82	1.60E-44	0.39535664	0.991	0.728	5.17E-40	12
Rpl245	2.00E-44	0.44014207	0.952	0.763	6.44E-40	12
Rps252	5.29E-43	0.38335839	0.889	0.624	1.71E-38	12
Ndufa25	8.92E-43	0.26721104	0.811	0.428	2.88E-38	12
Atp5md4	3.64E-41	0.36246041	0.849	0.533	1.17E-36	12
Clic13	6.55E-41	0.38457356	0.844	0.464	2.12E-36	12
Rpl215	1.52E-40	0.39910063	0.946	0.716	4.90E-36	12
Rpl172	2.02E-40	0.29802929	0.932	0.666	6.51E-36	12
Rack12	4.27E-40	0.30136829	0.923	0.526	1.38E-35	12

Rps96	1.49E-38	0.35613994	0.956	0.874	4.81E-34	12
Ubl55	1.74E-37	0.35191141	0.825	0.514	5.61E-33	12
Dstn4	1.78E-35	0.33515239	0.744	0.399	5.76E-31	12
Ubb6	4.27E-34	0.39481794	0.951	0.757	1.38E-29	12
Arpc35	2.29E-33	0.32719118	0.86	0.51	7.40E-29	12
Cd632	1.59E-26	0.35457188	0.477	0.269	5.14E-22	12
Pfn14	4.18E-26	0.33736682	0.939	0.746	1.35E-21	12
Cyba7	8.03E-25	0.27686917	0.847	0.524	2.59E-20	12
Crip14	3.72E-24	0.38663107	0.668	0.396	1.20E-19	12
Cfl14	1.25E-22	0.29657295	0.881	0.614	4.04E-18	12
Col3a1	0	6.82208463	0.907	0.015	0	13
Col1a1	0	6.79241161	0.923	0.012	0	13
Col1a2	0	6.60529587	0.946	0.012	0	13
Sparc	0	5.7472117	0.981	0.075	0	13
Bgn	0	5.139556	0.892	0.008	0	13
Dcn	0	5.02904345	0.676	0.003	0	13
Timp1	0	4.72844169	0.657	0.036	0	13
Mgp	0	4.69062025	0.558	0.005	0	13
Col5a2	0	4.22602959	0.728	0.002	0	13
Fstl1	0	4.10259445	0.7	0.007	0	13
Tnc	0	3.96762519	0.418	0.008	0	13
Sfrp1	0	3.8737969	0.411	0.001	0	13
Serpinh1	0	3.80288865	0.739	0.038	0	13
Serping1	0	3.74909631	0.568	0.004	0	13
Mfap5	0	3.74852905	0.514	0	0	13
Aebp1	0	3.6965343	0.579	0.011	0	13
Lum	0	3.55398128	0.485	0.001	0	13
Col5a1	0	3.5525117	0.588	0.016	0	13
Fbn1	0	3.52172609	0.518	0.007	0	13
Fn13	0	3.4986403	0.887	0.181	0	13
Igfbp7	0	3.49523982	0.701	0.057	0	13
Tagln	0	3.48841718	0.343	0.002	0	13
Ccdc80	0	3.41388879	0.511	0.005	0	13
Mmp2	0	3.300647	0.459	0.001	0	13
Timp3	0	3.27441791	0.399	0.013	0	13
Col15a1	0	3.26140727	0.44	0.002	0	13
Postn	0	3.21846122	0.361	0.001	0	13
Col6a3	0	3.183047	0.429	0.004	0	13
Lox	0	3.13793037	0.424	0.002	0	13
Cald11	0	3.10870317	0.63	0.09	0	13
Gpx3	0	3.10671909	0.426	0.009	0	13
Rbp1	0	3.08161311	0.462	0.025	0	13
Col4a1	0	3.05007802	0.492	0.058	0	13

Serpinf1	0	2.92240039	0.42	0.008	0	13
Col12a1	0	2.91254052	0.324	0.002	0	13
Serpine2	0	2.90500281	0.305	0.016	0	13
Acta2	0	2.87990798	0.274	0.018	0	13
Col6a1	0	2.83955445	0.526	0.036	0	13
Meg3	0	2.75906754	0.352	0.001	0	13
Col6a2	0	2.72684866	0.43	0.02	0	13
Igf1	0	2.71995082	0.303	0.004	0	13
Col4a2	0	2.64232904	0.368	0.027	0	13
Rcn3	0	2.6418417	0.381	0.008	0	13
Fbln21	0	2.59631617	0.676	0.144	0	13
Lrp11	0	2.53747038	0.473	0.051	0	13
Cpxm1	0	2.49709785	0.277	0.002	0	13
Plod2	0	2.49357578	0.42	0.03	0	13
Vcan	0	2.4535709	0.339	0.019	0	13
Loxl1	0	2.40611738	0.305	0.003	0	13
Snhg18	0	2.38460858	0.33	0.003	0	13
Thbs2	0	2.38270632	0.277	0.001	0	13
Mmp14	0	2.30950528	0.334	0.035	0	13
Bmp1	0	2.2781668	0.295	0.008	0	13
Palld	0	2.24090524	0.267	0.006	0	13
Cdh11	0	2.14679311	0.25	0.003	0	13
Ppic	0	1.97505944	0.281	0.023	0	13
Prnp	0	1.96571191	0.253	0.02	0	13
Loxl2	9.35E-308	2.00873596	0.28	0.025	3.02E-303	13
Igfbp41	2.22E-286	3.01738232	0.632	0.163	7.16E-282	13
Selenom1	1.17E-274	2.00797294	0.346	0.045	3.78E-270	13
Cd633	6.98E-203	2.16108882	0.685	0.263	2.25E-198	13
Gm424182	1.99E-169	1.34346183	0.991	0.94	6.42E-165	13
Lgals11	2.11E-165	1.35150136	0.875	0.395	6.81E-161	13
Pmepa11	8.69E-162	1.86465681	0.464	0.123	2.81E-157	13
Nid1	7.57E-145	1.76053914	0.297	0.058	2.44E-140	13
Rrbp12	1.29E-134	1.76573232	0.614	0.267	4.17E-130	13
Emp11	1.89E-127	1.70854587	0.377	0.1	6.09E-123	13
Calu1	1.02E-125	1.61888642	0.421	0.126	3.29E-121	13
Vkorc11	1.28E-117	1.64099891	0.25	0.051	4.13E-113	13
Pcolce1	6.51E-117	1.52550864	0.309	0.073	2.10E-112	13
P4hb3	2.47E-106	1.88013582	0.459	0.176	7.96E-102	13
Spp12	2.16E-90	2.72400511	0.545	0.263	6.97E-86	13
Laptm4a1	2.15E-87	1.6818409	0.393	0.147	6.94E-83	13
Crip15	1.30E-85	1.31527792	0.719	0.394	4.19E-81	13
Itgb12	2.99E-78	1.45048182	0.486	0.221	9.65E-74	13
C33	4.01E-76	2.63535657	0.355	0.142	1.29E-71	13

Zbtb20	1.37E-74	1.38402275	0.25	0.07	4.43E-70	13
Ctsl2	9.04E-74	1.41814977	0.459	0.205	2.92E-69	13
App1	2.82E-73	1.62073282	0.386	0.16	9.11E-69	13
Csrp21	1.31E-72	1.71476867	0.264	0.081	4.23E-68	13
Nme24	1.45E-70	1.05055742	0.68	0.406	4.69E-66	13
Tpm11	7.44E-70	1.49048842	0.423	0.18	2.40E-65	13
Lman1	4.19E-66	1.35686903	0.268	0.086	1.35E-61	13
Hspa52	1.30E-63	1.30564247	0.579	0.353	4.21E-59	13
Hsp90b13	2.61E-63	1.27308989	0.596	0.375	8.44E-59	13
Ptms1	1.46E-58	1.29016659	0.381	0.168	4.72E-54	13
Rplp12	2.28E-56	0.57218055	0.959	0.704	7.36E-52	13
Rps122	8.56E-56	0.65894273	0.898	0.686	2.76E-51	13
Timp23	2.36E-55	1.48372966	0.37	0.169	7.63E-51	13
Calr3	9.82E-54	1.24209683	0.457	0.24	3.17E-49	13
Kdelr21	3.86E-53	1.45290944	0.312	0.132	1.25E-48	13
Xist3	2.57E-52	1.12907054	0.323	0.128	8.28E-48	13
Pdia33	4.19E-51	1.35402542	0.454	0.254	1.35E-46	13
Nedd41	2.36E-50	1.06301152	0.439	0.22	7.63E-46	13
Rps83	1.97E-49	0.54495028	0.953	0.744	6.37E-45	13
Ctsb3	3.28E-48	1.16637453	0.439	0.236	1.06E-43	13
Vim3	4.48E-47	0.79897276	0.703	0.487	1.45E-42	13
Wls	4.51E-46	1.17623952	0.268	0.106	1.46E-41	13
Hdlbp1	6.74E-46	1.35591107	0.314	0.142	2.18E-41	13
Tnfaip22	3.21E-44	1.43426787	0.358	0.182	1.04E-39	13
Mt12	2.78E-43	1.50598157	0.44	0.238	8.99E-39	13
Tceal92	3.56E-43	1.3484276	0.314	0.147	1.15E-38	13
Tpt12	1.31E-41	0.4770978	0.926	0.866	4.24E-37	13
Selenos2	7.81E-41	1.31263849	0.278	0.124	2.52E-36	13
Ppib3	4.28E-39	1.20368031	0.49	0.334	1.38E-34	13
Tpm42	1.93E-38	1.20479085	0.426	0.253	6.23E-34	13
mt-Cytb4	3.92E-37	0.61345951	0.811	0.663	1.27E-32	13
Rps152	1.51E-35	0.64649775	0.719	0.533	4.86E-31	13
Ckap43	6.64E-35	1.4661729	0.34	0.195	2.14E-30	13
S100a12	1.83E-34	1.28156537	0.252	0.114	5.91E-30	13
Thbs14	2.20E-34	0.92843958	0.399	0.213	7.09E-30	13
Malat13	1.67E-33	0.72437203	0.965	0.957	5.38E-29	13
Runx1	4.07E-32	1.26374733	0.253	0.12	1.31E-27	13
Slc25a41	1.56E-31	0.96808378	0.328	0.173	5.02E-27	13
Sptbn11	2.67E-31	1.17845196	0.3	0.157	8.61E-27	13
Cd811	2.70E-31	1.17230832	0.314	0.169	8.73E-27	13
Myl62	2.83E-30	0.722661	0.764	0.706	9.12E-26	13
Rps215	6.49E-29	0.28442305	0.948	0.791	2.10E-24	13
Gnas1	8.56E-29	0.85408297	0.568	0.443	2.76E-24	13

Mt21	1.84E-27	1.33939935	0.259	0.125	5.96E-23	13
Ly6a1	2.73E-27	1.01382075	0.289	0.146	8.81E-23	13
Ssr31	4.56E-27	1.11864916	0.321	0.186	1.47E-22	13
Rps173	1.44E-26	0.54824358	0.711	0.562	4.64E-22	13
Rpl417	5.86E-26	0.36520825	0.972	0.901	1.89E-21	13
Sec61g5	7.60E-26	0.62290033	0.701	0.611	2.45E-21	13
Rpl23a2	2.04E-25	0.34900511	0.695	0.512	6.58E-21	13
Manf2	9.42E-25	1.02927226	0.337	0.207	3.04E-20	13
Eef1a12	1.64E-24	0.33483867	0.882	0.724	5.30E-20	13
Ostc2	4.53E-24	1.13631033	0.299	0.179	1.46E-19	13
Ifitm35	3.24E-23	0.51297027	0.598	0.465	1.05E-18	13
Pdia62	2.61E-22	1.07015247	0.271	0.155	8.43E-18	13
Rpl52	6.44E-22	0.59969367	0.604	0.459	2.08E-17	13
Sec621	6.63E-22	0.93593211	0.415	0.296	2.14E-17	13
Marcks5	8.26E-22	0.96949915	0.323	0.197	2.67E-17	13
Npc24	5.43E-21	1.05586036	0.371	0.249	1.75E-16	13
Rpl13a2	3.34E-20	0.43180793	0.705	0.57	1.08E-15	13
Bsg2	3.77E-20	0.94520472	0.371	0.254	1.22E-15	13
Arf4	4.09E-19	1.03143996	0.305	0.196	1.32E-14	13
Selenof2	1.93E-18	0.95004661	0.355	0.249	6.22E-14	13
Lmna1	3.65E-18	0.59570509	0.295	0.169	1.18E-13	13
Hif1a2	5.27E-18	0.98939313	0.278	0.173	1.70E-13	13
Ssr44	5.28E-18	0.87597664	0.355	0.251	1.71E-13	13
Dad14	1.09E-17	0.90685565	0.348	0.243	3.51E-13	13
Rpl123	1.65E-17	0.31667828	0.713	0.537	5.34E-13	13
mt-Atp64	2.23E-17	0.36333468	0.819	0.746	7.21E-13	13
Anxa53	3.77E-17	0.78869609	0.365	0.251	1.22E-12	13
Lars22	4.04E-17	0.87438331	0.346	0.238	1.30E-12	13
Canx2	5.16E-17	0.8688642	0.345	0.239	1.67E-12	13
Rpl316	5.87E-16	0.47817005	0.648	0.544	1.89E-11	13
Ahnak2	6.55E-16	0.45806347	0.417	0.277	2.11E-11	13
Serf22	1.85E-15	0.48510892	0.741	0.729	5.96E-11	13
Sdc42	2.78E-15	0.75541378	0.252	0.149	8.97E-11	13
Egr12	6.44E-15	0.93851996	0.275	0.179	2.08E-10	13
Krtcap24	1.22E-14	0.7862459	0.349	0.255	3.93E-10	13
Gm100767	2.68E-14	0.26863326	0.891	0.81	8.64E-10	13
Rack13	3.10E-14	0.36112619	0.652	0.534	1.00E-09	13
Selenow4	1.22E-12	0.67896909	0.358	0.265	3.95E-08	13
mt-Nd44	1.24E-12	0.47732421	0.558	0.491	4.01E-08	13
Rpn12	9.22E-12	0.84277267	0.252	0.173	2.98E-07	13
Eef1b23	1.98E-11	0.39919569	0.617	0.537	6.40E-07	13
mt-Nd12	1.05E-10	0.39005313	0.552	0.475	3.38E-06	13
Lamp24	2.88E-09	0.8633913	0.284	0.222	9.31E-05	13

Mrpl525	3.80E-09	0.67299255	0.371	0.303	0.00012255	13
AY0361181	1.09E-08	1.02935514	0.345	0.291	0.00035308	13
Mif3	1.25E-08	0.45666574	0.426	0.341	0.00040228	13
Ost44	4.27E-08	0.76027198	0.378	0.351	0.00137948	13
Dynll13	5.03E-08	0.49881503	0.486	0.455	0.00162443	13
Spcs11	7.87E-08	0.864636	0.261	0.206	0.0025403	13
mt-Nd23	8.15E-08	0.45978239	0.448	0.383	0.00263059	13
Hspa82	2.08E-07	0.31579751	0.586	0.552	0.00672907	13
Hmgn13	4.68E-07	0.45582223	0.318	0.249	0.0150969	13
Eef1g2	4.88E-07	0.52364428	0.351	0.288	0.01577015	13
Fkbp1a2	4.98E-07	0.59948908	0.268	0.205	0.01606601	13
Prdx16	6.63E-07	0.41776356	0.457	0.396	0.02140521	13
Tmed102	1.14E-06	0.83199821	0.27	0.225	0.0367349	13
Rpl76	1.22E-06	0.25961875	0.689	0.635	0.0394204	13
Morf4l13	3.49E-06	0.58477076	0.395	0.371	0.11265994	13
Micos105	3.67E-06	0.65699863	0.312	0.272	0.1184726	13
Rps27l5	4.10E-06	0.50157227	0.393	0.349	0.13251378	13
Rps63	4.86E-06	0.33406029	0.543	0.503	0.15696527	13
Myl12a1	1.45E-05	0.6105756	0.395	0.38	0.46729517	13
Rab2a1	1.72E-05	0.77084954	0.259	0.22	0.55490803	13
Cst33	2.63E-05	1.55133532	0.462	0.478	0.84789244	13
S100a102	2.89E-05	0.3891173	0.465	0.407	0.93309794	13
Eef22	3.82E-05	0.48598896	0.414	0.387	1	13
Sec61b4	4.68E-05	0.43500401	0.459	0.45	1	13
Tm9sf31	5.12E-05	0.5993729	0.256	0.215	1	13
Csnk1a13	7.46E-05	0.66281182	0.278	0.245	1	13
Dynlrb13	0.00010499	0.65144091	0.255	0.221	1	13
mt-Nd33	0.00016845	0.357556	0.345	0.304	1	13
Romo13	0.00028462	0.48384087	0.37	0.352	1	13
Swi54	0.00036539	0.69557295	0.274	0.249	1	13
Tagln24	0.00088698	0.50806059	0.327	0.31	1	13
Anxa24	0.00115528	0.395917	0.432	0.423	1	13
S100a133	0.00275457	0.69002227	0.297	0.292	1	13
Eif2s21	0.00387194	0.27839239	0.448	0.44	1	13
Slc4a11	0	4.38165879	0.879	0.041	0	14
Car21	0	3.94301133	0.927	0.068	0	14
Gypa1	0	3.83043331	0.879	0.041	0	14
Rhd1	0	3.76406763	0.816	0.017	0	14
Hemgn	0	3.64366547	0.777	0.01	0	14
Aqp1	0	3.63096528	0.732	0.015	0	14
Hba-a11	0	3.56500608	0.967	0.096	0	14
Hist1h2ap3	0	3.53626513	0.9	0.237	0	14
Ctse1	0	3.24946835	0.728	0.027	0	14

Hbb-bt1	0	3.24699866	0.964	0.099	0	14
Blvrb3	0	3.22244097	0.847	0.141	0	14
Slc25a371	0	3.20977215	0.726	0.083	0	14
Mki671	0	3.19185931	0.838	0.156	0	14
Hmbs1	0	3.15904947	0.821	0.065	0	14
Tfrc1	0	3.13720636	0.75	0.095	0	14
Mgst3	0	3.11115951	0.719	0.076	0	14
Hbb-bs1	0	3.07765397	0.971	0.225	0	14
Prdx22	0	3.03865164	0.948	0.316	0	14
Sptb	0	2.96832546	0.597	0.008	0	14
Hist1h2ae2	0	2.94464921	0.812	0.178	0	14
Hba-a21	0	2.9227339	0.962	0.097	0	14
Cpox	0	2.8774298	0.633	0.024	0	14
Cldn13	0	2.84287598	0.605	0.012	0	14
Atpif14	0	2.83522977	0.949	0.413	0	14
Ermap	0	2.74033441	0.574	0.012	0	14
Kel1	0	2.68191573	0.535	0.015	0	14
Spta11	0	2.6777528	0.517	0.013	0	14
Tspan33	0	2.63406045	0.542	0.012	0	14
Ank1	0	2.61822132	0.535	0.01	0	14
Glrx51	0	2.58983396	0.728	0.134	0	14
Cd24a4	0	2.56684582	0.943	0.412	0	14
Alas21	0	2.54463946	0.605	0.041	0	14
Rhag	0	2.51891527	0.522	0.012	0	14
Abcb10	0	2.40780484	0.493	0.02	0	14
Fech1	0	2.40077275	0.568	0.048	0	14
Bsg3	0	2.38763377	0.829	0.242	0	14
Pdzk1ip1	0	2.38028255	0.396	0.012	0	14
Sox6	0	2.34837427	0.406	0.013	0	14
Nxpe2	0	2.32131748	0.485	0.01	0	14
Car1	0	2.29820642	0.312	0.006	0	14
Tspo21	0	2.28673397	0.455	0.021	0	14
Dmtn	0	2.25237144	0.385	0.005	0	14
Birc52	0	2.23780054	0.659	0.135	0	14
Rrm22	0	2.21686449	0.537	0.084	0	14
Alad	0	2.18708409	0.478	0.026	0	14
Pigq	0	2.17122554	0.44	0.027	0	14
Ubac1	0	2.15853422	0.458	0.032	0	14
Icam4	0	2.15634476	0.419	0.007	0	14
Clcn31	0	2.09358644	0.519	0.071	0	14
Abcg2	0	1.9884882	0.369	0.015	0	14
Ranbp101	0	1.94055977	0.393	0.038	0	14
Hebp1	0	1.92414387	0.37	0.013	0	14

Tfdp21	0	1.91967421	0.533	0.081	0	14
Klf1	0	1.91252142	0.374	0.003	0	14
Snca1	0	1.90612513	0.442	0.037	0	14
Zfpm11	0	1.89716021	0.424	0.042	0	14
Isg201	0	1.89565864	0.489	0.063	0	14
Btnl10	0	1.81232751	0.316	0.005	0	14
Slc43a1	0	1.79361744	0.312	0.011	0	14
Slc16a10	0	1.78707321	0.357	0.024	0	14
Urod1	0	1.76287829	0.405	0.049	0	14
Tal1	0	1.72252999	0.285	0.007	0	14
Rnf123	0	1.7187136	0.303	0.019	0	14
Epor	0	1.70140377	0.282	0.003	0	14
Fam210b1	0	1.68765045	0.341	0.024	0	14
Slc43a3	0	1.68465525	0.334	0.028	0	14
Smim1	0	1.67974068	0.307	0.007	0	14
Slc38a5	0	1.67069161	0.272	0.002	0	14
Epb42	0	1.64864846	0.284	0.012	0	14
Atp2b4	0	1.63575058	0.287	0.02	0	14
Ppox	0	1.57873202	0.281	0.022	0	14
Ptdss2	0	1.48478646	0.269	0.013	0	14
Abcb4	0	1.46180103	0.263	0.011	0	14
Trim101	0	1.40339955	0.284	0.022	0	14
Hist1h4d2	6.11E-307	2.37188846	0.545	0.096	1.97E-302	14
Hist1h1b2	7.68E-297	2.2493717	0.605	0.117	2.48E-292	14
Hist1h4h1	8.23E-289	2.24466768	0.515	0.089	2.66E-284	14
Ppp1r15a1	3.44E-288	1.97549589	0.576	0.106	1.11E-283	14
Gpx14	2.49E-285	2.1605746	0.961	0.509	8.03E-281	14
Hist1h3c1	4.41E-284	2.13054003	0.437	0.065	1.42E-279	14
Hagh1	1.42E-273	1.81236049	0.444	0.068	4.57E-269	14
8-Mar	2.28E-265	1.57251749	0.277	0.026	7.36E-261	14
Hist1h2bb1	5.35E-265	1.96147215	0.378	0.051	1.73E-260	14
Kcnn4	4.50E-262	1.65654368	0.359	0.045	1.45E-257	14
Lbr3	1.59E-259	2.45072012	0.765	0.245	5.14E-255	14
Top2a2	3.28E-257	2.13515725	0.693	0.177	1.06E-252	14
Gch1	9.75E-256	1.71133024	0.349	0.044	3.15E-251	14
Stom	1.92E-246	1.7671515	0.346	0.045	6.19E-242	14
Ube2c2	1.46E-243	2.34155571	0.615	0.148	4.71E-239	14
Gclm1	5.26E-243	1.91534402	0.506	0.098	1.70E-238	14
Arf52	4.52E-241	2.22196805	0.827	0.351	1.46E-236	14
Rad23a1	1.21E-233	1.77513076	0.478	0.089	3.90E-229	14
Odc1	3.48E-232	1.85911836	0.548	0.118	1.12E-227	14
Hist1h2bn1	9.75E-227	1.53594548	0.254	0.026	3.15E-222	14
E2f21	2.11E-226	1.91511718	0.403	0.067	6.82E-222	14

Hist1h2br1	2.28E-220	1.99467542	0.395	0.066	7.36E-216	14
Nusap12	3.95E-218	2.02984793	0.551	0.127	1.28E-213	14
Fam117a	9.37E-216	1.6789251	0.357	0.055	3.02E-211	14
Dek4	2.74E-215	1.94168329	0.796	0.299	8.83E-211	14
Creg13	4.54E-207	1.88438274	0.577	0.146	1.47E-202	14
Cdca32	7.41E-207	1.7344881	0.416	0.077	2.39E-202	14
Ccne21	1.70E-205	1.93482757	0.341	0.052	5.49E-201	14
Tubb4b2	1.42E-204	2.02347285	0.61	0.175	4.59E-200	14
Tmem14c3	3.85E-202	2.66380074	0.644	0.227	1.24E-197	14
H2afx2	8.53E-202	1.88973903	0.54	0.131	2.75E-197	14
Dhrs111	2.10E-201	1.39836508	0.269	0.033	6.77E-197	14
Tmcc21	6.13E-191	1.20805263	0.294	0.039	1.98E-186	14
Josd2	8.20E-189	1.40040417	0.315	0.048	2.65E-184	14
Hist1h3b1	3.20E-187	1.59161978	0.299	0.044	1.03E-182	14
Ncapg2	7.15E-182	1.50445852	0.299	0.044	2.31E-177	14
Hist2h2ac1	7.44E-181	1.64239336	0.29	0.043	2.40E-176	14
Herc1	6.63E-178	1.68656678	0.414	0.085	2.14E-173	14
Slc16a11	6.46E-176	1.53418488	0.341	0.058	2.09E-171	14
Ccna21	7.63E-174	1.65739462	0.385	0.076	2.46E-169	14
Fbxo51	1.05E-167	1.38758825	0.287	0.044	3.39E-163	14
Hmgb25	1.43E-165	1.16039368	0.962	0.635	4.62E-161	14
Ehbp1l1	2.18E-162	1.58129879	0.343	0.064	7.03E-158	14
Hist1h2ab1	3.29E-159	1.498395	0.287	0.047	1.06E-154	14
Lockd1	1.48E-155	1.60605936	0.427	0.101	4.77E-151	14
Esco21	7.86E-155	1.40074714	0.266	0.041	2.54E-150	14
Bola33	1.35E-153	1.64604039	0.45	0.115	4.36E-149	14
Hist1h1d1	8.49E-153	1.81305993	0.344	0.069	2.74E-148	14
Pabpc4	1.53E-152	1.26060144	0.356	0.07	4.93E-148	14
Hist1h1a1	1.59E-149	1.34966082	0.276	0.045	5.14E-145	14
Knl1	2.05E-148	1.44586158	0.303	0.055	6.62E-144	14
Trim59	1.05E-147	1.50658351	0.328	0.062	3.38E-143	14
Cd473	8.15E-147	1.63454016	0.793	0.394	2.63E-142	14
Epb411	2.78E-146	1.48093922	0.388	0.086	8.97E-142	14
Prc11	3.49E-146	1.60407641	0.393	0.09	1.13E-141	14
Cenpf1	3.17E-142	1.67282219	0.388	0.089	1.02E-137	14
Hist1h3f1	3.36E-142	1.41723173	0.264	0.044	1.09E-137	14
Rbm382	6.41E-140	1.66548837	0.496	0.144	2.07E-135	14
Tuba1b4	1.92E-139	1.43219085	0.641	0.227	6.18E-135	14
Tmx1	5.02E-139	1.41438846	0.302	0.057	1.62E-134	14
Trak21	5.35E-138	1.3852344	0.292	0.053	1.73E-133	14
Smc22	3.48E-132	1.51996092	0.432	0.117	1.12E-127	14
Ube2l61	3.51E-129	1.18982061	0.274	0.05	1.13E-124	14
Cdk12	7.60E-128	1.4682905	0.38	0.094	2.46E-123	14

Cd82	1.37E-127	1.39918129	0.303	0.061	4.43E-123	14
Hist1h2bj1	3.71E-127	1.30159096	0.256	0.045	1.20E-122	14
Gclc1	1.30E-126	1.14138672	0.264	0.047	4.20E-122	14
Ube2s2	1.36E-126	1.50834216	0.697	0.298	4.40E-122	14
Lmo22	4.28E-126	1.2426878	0.323	0.07	1.38E-121	14
Hist1h4i1	8.15E-121	1.60663883	0.362	0.09	2.63E-116	14
Metap23	2.23E-120	1.51680733	0.597	0.237	7.20E-116	14
Fam241a	5.28E-119	1.33442438	0.326	0.075	1.70E-114	14
Dleu22	5.87E-118	1.59190753	0.442	0.133	1.89E-113	14
Rrm12	3.02E-116	1.31902032	0.347	0.085	9.75E-112	14
Hmmr	1.53E-115	1.37970505	0.292	0.062	4.94E-111	14
Ncapd21	8.76E-114	1.34620004	0.281	0.058	2.83E-109	14
Kif111	1.21E-111	1.33908461	0.308	0.07	3.92E-107	14
Rb1	1.18E-109	1.28856401	0.277	0.058	3.82E-105	14
Hist1h3e1	3.35E-109	1.21876369	0.264	0.054	1.08E-104	14
Ccnb22	5.00E-109	1.26907446	0.347	0.089	1.61E-104	14
Serinc35	7.31E-108	1.48600768	0.719	0.354	2.36E-103	14
E2f4	1.42E-104	1.05247398	0.261	0.053	4.60E-100	14
Ncoa7	2.34E-103	1.27089017	0.261	0.055	7.57E-99	14
Cpeb41	2.59E-102	1.25061583	0.258	0.054	8.37E-98	14
Hist1h1e1	5.21E-101	1.41823571	0.454	0.15	1.68E-96	14
Anp32b3	4.72E-100	1.38167143	0.628	0.286	1.53E-95	14
Isca11	8.97E-100	1.18840445	0.315	0.078	2.89E-95	14
Cks23	1.56E-96	1.25642491	0.589	0.252	5.03E-92	14
Sgo11	1.44E-95	1.24777991	0.282	0.067	4.66E-91	14
Tmpo2	2.35E-95	1.51288652	0.468	0.166	7.60E-91	14
Arl4a1	9.15E-94	0.91096788	0.25	0.053	2.95E-89	14
Slbp2	1.84E-93	1.45174395	0.418	0.14	5.93E-89	14
Tyms2	1.68E-88	1.17762355	0.297	0.078	5.43E-84	14
Uhrf12	2.05E-88	1.13473286	0.313	0.083	6.62E-84	14
Cdkn33	6.44E-87	1.28981457	0.259	0.063	2.08E-82	14
Acp1	1.52E-86	1.14883026	0.326	0.091	4.90E-82	14
Grina3	3.19E-85	1.21282197	0.644	0.301	1.03E-80	14
Usp7	4.87E-85	1.24892418	0.321	0.09	1.57E-80	14
H3f3a5	5.71E-84	0.81859972	0.943	0.758	1.84E-79	14
Pcna3	1.58E-83	1.478079	0.467	0.184	5.12E-79	14
Klf3	2.51E-83	1.31422487	0.334	0.098	8.12E-79	14
Cyb5a2	5.12E-83	1.27685864	0.437	0.159	1.65E-78	14
Cenpa3	5.63E-83	1.17042079	0.405	0.135	1.82E-78	14
Mcm72	6.20E-82	1.28021201	0.392	0.133	2.00E-77	14
5-Mar	2.64E-81	1.00806567	0.287	0.076	8.51E-77	14
Tubb55	5.23E-80	1.00028173	0.715	0.354	1.69E-75	14
21-Mar	1.99E-76	1.09063105	0.256	0.065	6.41E-72	14

Azin11	4.84E-75	1.30867086	0.374	0.13	1.56E-70	14
Tpx21	2.64E-74	1.13411578	0.299	0.087	8.53E-70	14
Nt5c31	4.48E-72	0.94919669	0.261	0.07	1.45E-67	14
Carhsp1	3.80E-71	0.99794859	0.323	0.101	1.23E-66	14
Lig12	1.58E-70	1.01045622	0.313	0.097	5.12E-66	14
Dck2	1.64E-70	1.03083951	0.295	0.086	5.29E-66	14
Cbx33	3.92E-70	1.02755453	0.643	0.336	1.27E-65	14
Cdca82	1.56E-67	1.04287773	0.29	0.089	5.04E-63	14
Arl6ip14	5.36E-67	1.60537017	0.463	0.206	1.73E-62	14
Itga42	1.14E-66	1.08023761	0.305	0.096	3.69E-62	14
Gmnn2	2.77E-66	1.05915123	0.318	0.105	8.94E-62	14
Wapl	7.63E-66	1.00878784	0.334	0.113	2.46E-61	14
Usp15	1.63E-65	1.16886326	0.318	0.106	5.25E-61	14
Adam10	5.25E-65	1.06589024	0.382	0.138	1.70E-60	14
Svbp	1.26E-64	0.96096064	0.29	0.09	4.07E-60	14
Oaz17	1.42E-62	0.81726127	0.822	0.638	4.58E-58	14
Hmgb31	2.61E-62	0.88607012	0.271	0.083	8.44E-58	14
Smc42	7.39E-62	1.0687436	0.455	0.192	2.39E-57	14
Reep53	1.78E-61	1.13949586	0.529	0.266	5.76E-57	14
Tipin2	9.83E-59	0.90227581	0.277	0.089	3.17E-54	14
Hist1h2bc1	1.37E-58	1.31675089	0.374	0.147	4.41E-54	14
Tuba4a2	1.61E-58	1.17887509	0.338	0.126	5.19E-54	14
Sec61g6	1.86E-53	0.83500332	0.793	0.609	6.01E-49	14
Orc61	3.87E-53	0.88428286	0.259	0.084	1.25E-48	14
Rgcc3	7.48E-53	0.94027242	0.331	0.126	2.42E-48	14
Atad21	1.29E-52	1.10939956	0.297	0.106	4.16E-48	14
Mcm62	8.37E-51	0.82033328	0.266	0.089	2.70E-46	14
Mkrn11	5.21E-49	0.75907005	0.372	0.154	1.68E-44	14
Ctsb4	7.69E-49	0.80598794	0.486	0.236	2.48E-44	14
AI6622705	2.18E-48	0.94479147	0.336	0.136	7.04E-44	14
Picalm3	5.77E-46	0.9414185	0.452	0.22	1.86E-41	14
Mrps362	1.29E-45	0.83608786	0.316	0.125	4.16E-41	14
Tmem2564	1.22E-44	0.85481967	0.509	0.272	3.93E-40	14
Smap1	5.96E-43	0.85614275	0.299	0.119	1.92E-38	14
Ubald22	6.38E-43	1.02036786	0.401	0.192	2.06E-38	14
Ccdc341	1.76E-42	0.81460474	0.294	0.115	5.69E-38	14
St3gal53	2.48E-41	0.91964143	0.292	0.117	8.01E-37	14
Eif13	3.61E-41	0.51543572	0.918	0.838	1.17E-36	14
Alyref4	4.61E-41	0.92769937	0.429	0.223	1.49E-36	14
Gpx43	5.90E-41	0.90374479	0.605	0.396	1.90E-36	14
Cbx51	1.54E-40	0.92133662	0.279	0.109	4.98E-36	14
Hist2h2aa13	1.58E-40	1.15655003	0.307	0.13	5.11E-36	14
Pkn21	9.51E-40	0.83423086	0.325	0.138	3.07E-35	14

Ago2	5.90E-39	0.95674717	0.318	0.136	1.90E-34	14
H2afz4	2.21E-38	0.57515118	0.781	0.572	7.14E-34	14
Rpgrip13	3.05E-37	0.78694407	0.285	0.12	9.83E-33	14
Myef21	9.11E-37	0.83529163	0.253	0.098	2.94E-32	14
Rbbp43	1.60E-34	0.73444239	0.359	0.17	5.17E-30	14
Tnfaip23	2.17E-34	0.54601472	0.377	0.182	7.00E-30	14
Rad211	1.95E-32	0.83226923	0.285	0.127	6.28E-28	14
Cenpw2	3.32E-32	0.74858387	0.263	0.112	1.07E-27	14
Nudt42	3.63E-32	0.89198558	0.361	0.183	1.17E-27	14
Xist4	4.96E-32	1.08900859	0.289	0.13	1.60E-27	14
Srsf103	9.77E-32	0.72567045	0.326	0.155	3.16E-27	14
Pclaf2	1.33E-31	0.42421246	0.356	0.165	4.29E-27	14
Pttg12	1.93E-31	0.82591264	0.295	0.135	6.24E-27	14
Ucp26	2.31E-31	0.55513679	0.573	0.347	7.46E-27	14
Eny23	3.17E-31	0.70782162	0.364	0.185	1.02E-26	14
Pcmt1	1.31E-30	0.8242375	0.274	0.124	4.24E-26	14
Hist1h1c1	2.52E-29	0.85395303	0.315	0.155	8.15E-25	14
Srsf24	7.15E-29	0.83383837	0.442	0.258	2.31E-24	14
Supt163	9.55E-29	0.74846563	0.331	0.166	3.08E-24	14
Pnp3	2.42E-28	0.65078664	0.281	0.131	7.82E-24	14
Ndufb94	6.60E-28	0.80281266	0.514	0.361	2.13E-23	14
Dennd4a1	1.99E-27	0.75836679	0.316	0.16	6.41E-23	14
mt-Co15	7.65E-27	0.49923999	0.93	0.883	2.47E-22	14
Mier12	6.39E-26	0.7349653	0.33	0.171	2.06E-21	14
H2afj3	1.13E-25	0.68596393	0.6	0.431	3.64E-21	14
Dtymk3	4.36E-25	0.64961757	0.294	0.148	1.41E-20	14
Cox6a14	7.37E-25	0.76203553	0.476	0.316	2.38E-20	14
Sp32	1.17E-24	0.81706068	0.272	0.134	3.76E-20	14
Dut4	1.66E-24	0.76123109	0.356	0.197	5.37E-20	14
AY0361182	3.09E-24	0.6003422	0.467	0.288	9.97E-20	14
Ndufa44	3.20E-24	0.6259951	0.617	0.464	1.03E-19	14
Cdkn2d2	3.62E-24	1.11023799	0.285	0.154	1.17E-19	14
Mrpl512	7.52E-23	0.61919982	0.263	0.13	2.43E-18	14
Stmn13	2.89E-22	0.34223707	0.45	0.259	9.33E-18	14
Ctbp11	1.03E-21	0.6507951	0.277	0.142	3.32E-17	14
Nfkbia4	2.05E-21	0.51860669	0.401	0.238	6.62E-17	14
Tspo3	5.21E-21	0.74143167	0.612	0.457	1.68E-16	14
Rpa32	5.46E-21	0.56283726	0.253	0.128	1.76E-16	14
Luc7l22	1.12E-20	0.68058078	0.364	0.215	3.63E-16	14
Srrm11	5.94E-20	0.62681631	0.32	0.181	1.92E-15	14
Rnaseh2c4	6.82E-20	0.72189902	0.369	0.23	2.20E-15	14
Celf1	2.01E-19	0.5825258	0.253	0.128	6.48E-15	14
Hipk12	2.49E-19	0.7510297	0.32	0.186	8.03E-15	14

Siva14	3.58E-19	0.59640837	0.289	0.159	1.16E-14	14
Cks1b3	7.79E-19	0.54072095	0.321	0.178	2.52E-14	14
Cox172	1.14E-18	0.64211034	0.561	0.453	3.67E-14	14
Snhg92	3.73E-18	0.63255509	0.259	0.138	1.21E-13	14
Anp32e3	6.78E-18	0.52666411	0.344	0.2	2.19E-13	14
Fam104a	8.96E-17	0.54578058	0.287	0.163	2.89E-12	14
Eef1d1	2.51E-16	0.39035231	0.485	0.327	8.09E-12	14
Bcas22	2.60E-16	0.46770058	0.259	0.142	8.39E-12	14
Tra2b4	2.94E-16	0.5793166	0.396	0.259	9.49E-12	14
Ybx32	3.37E-16	0.40985544	0.321	0.186	1.09E-11	14
Wnk12	6.23E-16	0.71455866	0.326	0.2	2.01E-11	14
Rsrp1	1.16E-15	0.63371806	0.321	0.197	3.76E-11	14
Smarca53	3.33E-15	0.47557727	0.388	0.253	1.07E-10	14
Ccnd33	4.33E-15	0.63495807	0.312	0.195	1.40E-10	14
Mrpl573	4.74E-15	0.42300869	0.308	0.185	1.53E-10	14
Snhg13	8.64E-15	0.46732784	0.336	0.209	2.79E-10	14
Sec622	3.51E-14	0.51629182	0.432	0.296	1.13E-09	14
Lars23	3.79E-14	0.45664347	0.375	0.237	1.22E-09	14
Pbrm11	4.39E-14	0.51078139	0.281	0.165	1.42E-09	14
Gm472834	4.39E-14	0.56540038	0.284	0.165	1.42E-09	14
Sumo24	2.02E-13	0.39380575	0.488	0.349	6.53E-09	14
Ran3	4.26E-13	0.33818425	0.511	0.355	1.37E-08	14
Calm33	7.70E-13	0.55350114	0.37	0.256	2.49E-08	14
Pabpc12	1.50E-12	0.32922714	0.701	0.585	4.83E-08	14
Tacc12	7.49E-12	0.57285903	0.253	0.155	2.42E-07	14
Snx32	1.63E-11	0.4176744	0.287	0.184	5.27E-07	14
Actn4	4.60E-11	0.47868179	0.253	0.156	1.49E-06	14
Tmem134	8.53E-11	0.39887087	0.266	0.167	2.75E-06	14
Srsf61	1.89E-10	0.46213937	0.258	0.162	6.09E-06	14
Ptbp33	3.25E-10	0.41859606	0.385	0.275	1.05E-05	14
Ywhah2	1.98E-09	0.38661329	0.339	0.236	6.40E-05	14
Atp5h4	4.67E-09	0.41655141	0.535	0.477	0.00015076	14
Ppp1cb3	5.42E-09	0.53133699	0.274	0.186	0.00017513	14
Ewsr12	7.91E-09	0.34087016	0.251	0.162	0.00025524	14
Smdt16	8.07E-09	0.34818311	0.439	0.34	0.0002605	14
Mrpl204	1.45E-08	0.36014335	0.287	0.199	0.00046956	14
Ndufa53	4.75E-08	0.40124611	0.29	0.206	0.00153212	14
Cltc	6.04E-08	0.37738984	0.328	0.236	0.00195082	14
Jpt12	6.53E-08	0.381012	0.326	0.233	0.00210886	14
1810058I24R	1.31E-07	0.68062897	0.326	0.251	0.00423031	14
Ndufb104	2.55E-07	0.32977543	0.326	0.244	0.00824711	14
Resf11	4.67E-07	0.44740663	0.268	0.187	0.0150815	14
Micos106	5.58E-07	0.34631442	0.347	0.271	0.01801502	14

Uqcr115	5.78E-07	0.45426628	0.426	0.361	0.01867498	14
Thoc73	6.98E-07	0.3065273	0.279	0.198	0.02252722	14
1810037I17R	1.51E-06	0.38220216	0.427	0.351	0.0487624	14
Erh5	1.59E-06	0.328958	0.308	0.229	0.05120043	14
Raly2	4.21E-06	0.29193674	0.294	0.216	0.13606256	14
Gm100768	5.30E-06	0.31430323	0.808	0.812	0.17095848	14
mt-Atp84	5.35E-06	0.31852162	0.359	0.278	0.17279385	14
Atf41	7.90E-06	0.38443151	0.307	0.234	0.25509477	14
Snrpe6	9.78E-06	0.26050566	0.498	0.43	0.31590281	14
Tut73	1.41E-05	0.27941946	0.279	0.206	0.45468984	14
Atp5b3	2.01E-05	0.26926167	0.392	0.317	0.64943186	14
Gabarapl22	3.03E-05	0.27987697	0.331	0.261	0.97909596	14
BC0055371	5.05E-05	0.36644536	0.282	0.217	1	14
Ndufab13	0.00011625	0.25900603	0.269	0.206	1	14
Vamp83	0.00020767	0.26293551	0.343	0.28	1	14
mt-Co34	3.12E-272	2.28589966	0.988	0.795	1.01E-267	15
mt-Co24	2.86E-263	2.19439261	0.988	0.782	9.24E-259	15
Gm424183	3.02E-258	2.42519838	0.996	0.94	9.73E-254	15
mt-Atp65	1.84E-247	2.2278199	0.974	0.743	5.94E-243	15
mt-Co16	3.06E-220	1.81562207	0.991	0.882	9.86E-216	15
Cp	4.63E-217	2.32154876	0.267	0.029	1.49E-212	15
mt-Cytb5	9.30E-217	2.09636614	0.956	0.66	3.00E-212	15
Spp13	2.43E-193	2.28110937	0.753	0.259	7.85E-189	15
mt-Nd13	1.39E-183	2.2952875	0.832	0.469	4.48E-179	15
mt-Nd24	2.74E-182	2.31500747	0.777	0.375	8.84E-178	15
mt-Nd45	8.60E-175	2.19672347	0.823	0.485	2.78E-170	15
Onecut21	4.45E-162	2.27058679	0.342	0.063	1.44E-157	15
mt-Nd34	6.82E-139	2.15209543	0.654	0.296	2.20E-134	15
Ahnak3	1.41E-122	2.05309364	0.633	0.272	4.54E-118	15
Clu1	4.12E-119	2.85256562	0.461	0.14	1.33E-114	15
Nedd42	1.96E-106	1.82839973	0.544	0.218	6.33E-102	15
Fbln22	1.41E-101	1.80212281	0.456	0.151	4.56E-97	15
Krt81	4.38E-98	1.6589212	0.493	0.173	1.41E-93	15
Hsp90ab12	4.26E-91	1.15338616	0.853	0.578	1.38E-86	15
mt-Nd53	4.35E-83	1.96191545	0.542	0.28	1.40E-78	15
Tm4sf11	1.27E-72	1.40544634	0.451	0.176	4.10E-68	15
Fn14	2.85E-71	1.36586005	0.474	0.194	9.21E-67	15
Cd2ap1	1.87E-66	1.63327289	0.372	0.142	6.04E-62	15
Plec1	1.49E-65	1.75547558	0.419	0.182	4.80E-61	15
Malat14	1.40E-63	1.11445585	0.989	0.957	4.53E-59	15
Map1b1	1.15E-59	1.62256108	0.267	0.084	3.72E-55	15
Eef1a13	4.67E-57	0.81057659	0.893	0.724	1.51E-52	15
Kcnq1ot11	2.36E-56	1.88431534	0.296	0.107	7.63E-52	15

mt-Nd4l5	2.42E-51	1.66236835	0.632	0.448	7.81E-47	15
Itga61	2.58E-51	1.61679485	0.258	0.088	8.33E-47	15
Scd21	4.23E-50	1.55285592	0.333	0.137	1.37E-45	15
Nfix1	4.63E-48	1.54971992	0.251	0.087	1.50E-43	15
Sox44	6.58E-45	1.51770509	0.346	0.156	2.12E-40	15
Pmepa12	1.37E-43	1.47913055	0.307	0.129	4.41E-39	15
Lmna2	1.47E-43	1.34350362	0.361	0.168	4.75E-39	15
Hspa83	5.27E-41	0.96787408	0.682	0.55	1.70E-36	15
Vim4	2.25E-38	0.9094709	0.663	0.489	7.25E-34	15
Krt181	5.61E-35	1.18081879	0.321	0.148	1.81E-30	15
Lars24	2.07E-34	1.5532355	0.396	0.237	6.67E-30	15
Myof1	1.48E-33	1.30998471	0.253	0.107	4.77E-29	15
Itgb13	1.60E-33	1.1944636	0.395	0.225	5.17E-29	15
Eif3a2	1.63E-33	1.32132414	0.416	0.255	5.25E-29	15
Npm12	1.50E-32	0.7279814	0.663	0.47	4.84E-28	15
Gnas2	7.58E-31	1.08451819	0.565	0.443	2.45E-26	15
Ccnd11	1.57E-29	1.28881804	0.281	0.133	5.07E-25	15
Neat12	8.55E-27	1.66704976	0.351	0.217	2.76E-22	15
Igfbp42	6.82E-26	1.17765009	0.316	0.173	2.20E-21	15
Rpl53	1.85E-25	0.7785023	0.588	0.46	5.97E-21	15
Rplp13	2.91E-25	0.49976527	0.856	0.708	9.38E-21	15
Rpl23a3	9.77E-25	0.73376491	0.646	0.514	3.16E-20	15
S100a43	1.36E-23	0.78620509	0.395	0.225	4.38E-19	15
Hsp90aa13	2.24E-23	1.04828313	0.491	0.37	7.25E-19	15
Mt22	2.85E-23	1.11784859	0.256	0.125	9.21E-19	15
Rps153	3.45E-23	0.68104542	0.642	0.535	1.11E-18	15
Rpl13a3	5.24E-23	0.66691348	0.674	0.571	1.69E-18	15
Rack14	3.26E-22	0.63364131	0.656	0.534	1.05E-17	15
Hspd12	2.00E-20	1.16344169	0.344	0.221	6.47E-16	15
Wfdc22	6.29E-20	0.81622157	0.295	0.158	2.03E-15	15
Hsp90b14	2.27E-19	1.03979009	0.472	0.38	7.33E-15	15
Ly6a2	3.43E-19	0.99230374	0.272	0.147	1.11E-14	15
Rps216	5.80E-19	0.40261742	0.875	0.793	1.87E-14	15
Taf1d1	3.17E-18	1.26205172	0.302	0.196	1.02E-13	15
Lgals12	1.39E-16	0.32265665	0.598	0.404	4.50E-12	15
B2m3	2.05E-15	0.66652938	0.688	0.713	6.61E-11	15
Sptbn12	2.28E-15	1.18510636	0.254	0.159	7.36E-11	15
mt-Atp85	2.86E-15	1.53223044	0.36	0.278	9.23E-11	15
Pdia34	4.88E-15	1.07179886	0.349	0.258	1.58E-10	15
Hspa53	1.07E-14	0.99537003	0.433	0.358	3.46E-10	15
Eef23	1.66E-14	0.90715877	0.447	0.386	5.37E-10	15
Pkm2	2.02E-14	1.02911	0.47	0.431	6.51E-10	15
Eef1g3	5.43E-14	0.85347896	0.381	0.288	1.75E-09	15

Mt13	8.85E-14	0.85823767	0.358	0.241	2.86E-09	15
Ptms2	3.15E-13	1.0193863	0.263	0.172	1.02E-08	15
Anxa54	2.07E-12	0.94533359	0.339	0.252	6.68E-08	15
Tpm12	2.67E-12	0.77717227	0.282	0.185	8.62E-08	15
Canx3	3.65E-12	1.11248557	0.318	0.24	1.18E-07	15
Rpl83	1.85E-11	0.27782568	0.83	0.733	5.98E-07	15
Rtn41	4.77E-11	1.03818087	0.253	0.174	1.54E-06	15
Hdgf2	9.82E-11	1.05125406	0.314	0.244	3.17E-06	15
Rbms11	1.16E-10	1.18315176	0.282	0.21	3.73E-06	15
Cd634	1.95E-10	0.90821617	0.342	0.274	6.30E-06	15
Hnrnpa2b13	3.43E-10	0.90743964	0.442	0.415	1.11E-05	15
Ly6e6	4.04E-10	0.79486359	0.454	0.408	1.30E-05	15
Rrbp13	4.53E-10	0.92863765	0.342	0.275	1.46E-05	15
AY0361183	1.15E-09	1.08589104	0.347	0.292	3.71E-05	15
Cd443	4.68E-09	1.05673005	0.316	0.267	0.00015122	15
Hnrnph13	6.99E-09	1.06009335	0.284	0.226	0.00022572	15
Rpl124	1.23E-08	0.37004228	0.609	0.54	0.00039581	15
Rps123	3.28E-08	0.30366446	0.714	0.692	0.0010577	15
Srrm21	4.60E-08	0.96672254	0.356	0.321	0.00148545	15
Hnrnpu2	7.66E-08	0.96960194	0.346	0.307	0.00247375	15
Tm9sf32	1.10E-07	1.0360293	0.268	0.215	0.00355334	15
Rpl44	1.40E-07	0.66646671	0.444	0.419	0.00451099	15
Wnk13	1.51E-07	0.98059283	0.256	0.202	0.00486512	15
Tpt13	3.02E-07	0.37015118	0.784	0.869	0.0097347	15
Calr4	1.31E-06	0.83106468	0.295	0.245	0.04225478	15
Ctsl3	2.62E-06	0.76142797	0.265	0.211	0.08468207	15
Ncl3	3.93E-06	0.52820454	0.449	0.417	0.12702766	15
Tpr1	7.13E-06	0.91328462	0.274	0.234	0.23010001	15
H2-K14	1.40E-05	0.6006175	0.46	0.462	0.45073065	15
Eif5b1	1.55E-05	0.81336821	0.26	0.218	0.50041964	15
Csnk1a14	0.00012923	0.89927445	0.27	0.245	1	15
Prdx17	0.0002103	0.53208555	0.398	0.397	1	15
Rpl317	0.0003097	0.26597948	0.582	0.562	1	15
Ccnl13	0.00033377	0.36412539	0.158	0.254	1	15
Hmgn14	0.00043805	0.55603279	0.284	0.251	1	15
Anxa25	0.000451	0.71124575	0.404	0.424	1	15
Gnb22	0.00045244	0.28419065	0.239	0.373	1	15
Psmb43	0.00066628	0.30054835	0.167	0.261	1	15
Set3	0.00077381	0.52727435	0.367	0.353	1	15
Csde11	0.00079819	0.91962406	0.268	0.252	1	15
Aldh23	0.00177907	0.33676692	0.179	0.274	1	15
Pafah1b12	0.00198868	0.38361825	0.174	0.265	1	15
Tle52	0.00221129	0.39675415	0.165	0.251	1	15

Tpm43	0.00260349	0.67789607	0.275	0.257	1	15
Nap1l13	0.00275509	0.66545995	0.316	0.311	1	15
Rps142	0.00278495	0.2567913	0.688	0.72	1	15
Son3	0.00343709	0.29658398	0.263	0.392	1	15
Ppp1r14b2	0.0043101	0.58895298	0.282	0.263	1	15
Ywhab3	0.00443103	0.43810125	0.211	0.316	1	15
Iqgap13	0.00504817	0.28310892	0.27	0.398	1	15
Rac12	0.00562069	0.34296431	0.204	0.298	1	15
Fus4	0.00585201	0.84453105	0.263	0.258	1	15
Cd474	0.00605472	0.25009354	0.277	0.408	1	15
Mbnl13	0.00686539	0.26936725	0.232	0.333	1	15
Serbp14	0.00709108	0.34300667	0.472	0.491	1	15
Tmbim63	0.00743023	0.38311793	0.205	0.303	1	15
Ywhae3	0.00818985	0.64529216	0.34	0.355	1	15
Ccl5	0	6.30172464	0.608	0.004	0	16
AW1120101	0	3.82385647	0.716	0.069	0	16
Ms4a4b	0	3.74764106	0.704	0.008	0	16
Nkg71	0	3.34715679	0.591	0.038	0	16
Trbc2	0	3.23661218	0.552	0.001	0	16
Cd3d	0	3.08701932	0.556	0.01	0	16
Cd3g	0	2.93385352	0.503	0.001	0	16
Xcl1	0	2.83125188	0.318	0.001	0	16
Klrd1	0	2.72617338	0.416	0.008	0	16
Ctla2a	0	2.61758768	0.461	0.028	0	16
Cd3e	0	2.57654593	0.411	0.001	0	16
Txk	0	2.55381912	0.433	0.001	0	16
Trbc1	0	2.52298237	0.375	0.001	0	16
Vps37b2	0	2.46905476	0.659	0.126	0	16
Il2rb	0	2.29092685	0.371	0.001	0	16
H2-Q71	0	2.12251958	0.589	0.086	0	16
Trac	0	1.97155753	0.281	0.002	0	16
Gimap3	0	1.95414233	0.32	0.005	0	16
Klre1	0	1.95384938	0.256	0	0	16
Ctsw	0	1.94598244	0.311	0.001	0	16
Cd7	0	1.90891058	0.254	0.009	0	16
Ptpn22	0	1.83981023	0.379	0.037	0	16
Gimap5	0	1.76441071	0.309	0.013	0	16
Tcf7	0	1.71895925	0.279	0.004	0	16
Gimap61	7.19E-287	1.72933182	0.352	0.035	2.32E-282	16
Pdcd44	2.04E-250	2.30290062	0.782	0.233	6.58E-246	16
Rps274	2.74E-247	1.68144494	0.998	0.911	8.85E-243	16
Rps247	1.81E-241	1.63679214	0.992	0.854	5.83E-237	16
Shisa52	1.10E-239	1.98435701	0.552	0.105	3.54E-235	16

Hcst4	7.31E-232	2.39562343	0.712	0.209	2.36E-227	16
Rps15a6	1.18E-218	1.59869654	0.994	0.784	3.81E-214	16
Rps297	4.06E-218	1.33326457	0.998	0.954	1.31E-213	16
Rpl37a7	1.07E-215	1.372349	1	0.903	3.44E-211	16
Rgs1	1.33E-215	1.95885052	0.316	0.038	4.28E-211	16
Rpl307	4.71E-210	1.4361966	0.994	0.816	1.52E-205	16
Uba526	3.40E-208	1.58184855	0.994	0.755	1.10E-203	16
Cd21	3.56E-208	1.41086565	0.347	0.044	1.15E-203	16
Tnfaip3	3.21E-206	1.92812953	0.427	0.072	1.04E-201	16
Bcl2	2.16E-200	1.37763731	0.256	0.026	6.98E-196	16
Rpl387	3.08E-200	1.29987721	0.996	0.841	9.94E-196	16
Tmsb105	6.50E-194	1.54396822	0.996	0.744	2.10E-189	16
Ptpn184	3.03E-193	1.9811843	0.766	0.286	9.78E-189	16
Rpl96	1.12E-189	1.43298817	0.987	0.771	3.60E-185	16
Ltb1	4.25E-187	1.74522545	0.36	0.056	1.37E-182	16
Rps136	2.27E-182	1.37187585	0.989	0.794	7.33E-178	16
Rpl397	9.01E-179	1.26206324	1	0.844	2.91E-174	16
H2-K15	5.96E-178	1.80603454	0.893	0.452	1.93E-173	16
Rps77	4.19E-176	1.43866684	0.983	0.728	1.35E-171	16
Rpl196	4.24E-173	1.27169029	0.991	0.758	1.37E-168	16
Rpl367	1.21E-164	1.28146048	0.989	0.759	3.91E-160	16
Rps187	2.41E-156	1.37103375	0.972	0.646	7.77E-152	16
Rps117	2.31E-152	1.28665843	0.979	0.727	7.46E-148	16
1-Sep	1.54E-150	1.62407384	0.384	0.079	4.97E-146	16
Rpl187	9.85E-150	1.26930021	0.976	0.711	3.18E-145	16
Rps206	1.60E-149	1.29424923	0.992	0.741	5.17E-145	16
Rpl237	6.97E-149	1.13855101	1	0.823	2.25E-144	16
Fau5	1.28E-147	0.9278859	0.996	0.942	4.13E-143	16
Rps27a6	5.12E-143	1.04547136	0.994	0.892	1.65E-138	16
Zfp36l22	5.39E-143	2.02258442	0.672	0.254	1.74E-138	16
Rps107	3.25E-142	1.14166804	0.991	0.802	1.05E-137	16
Rpl347	9.91E-142	1.10819447	0.989	0.824	3.20E-137	16
Ms4a6b1	1.84E-141	1.38000765	0.25	0.035	5.93E-137	16
Il7r3	4.05E-140	1.67780253	0.356	0.069	1.31E-135	16
Rpl18a7	4.23E-138	1.13033361	0.991	0.805	1.37E-133	16
Spry2	8.85E-138	2.17374582	0.313	0.057	2.86E-133	16
Rps37	3.52E-136	1.20978116	0.966	0.705	1.14E-131	16
Rps57	8.64E-136	1.24103992	0.966	0.68	2.79E-131	16
Rpl135	1.73E-135	1.10167008	0.991	0.791	5.60E-131	16
Rpl116	4.82E-131	1.14229094	0.976	0.738	1.56E-126	16
Rpl216	8.40E-131	1.15650664	0.977	0.717	2.71E-126	16
Rpl27a6	3.05E-130	1.07173245	0.991	0.798	9.85E-126	16
Rpl35a7	2.84E-128	1.03683097	0.994	0.855	9.17E-124	16

Rpl277	1.33E-125	1.13383582	0.962	0.722	4.28E-121	16
Rps287	1.82E-124	1.05145091	0.994	0.844	5.87E-120	16
Ifngr12	5.13E-123	1.63787337	0.427	0.112	1.66E-118	16
Rpsa5	1.06E-120	1.13203356	0.976	0.699	3.43E-116	16
Ets13	4.33E-119	1.5263212	0.499	0.148	1.40E-114	16
Rps168	7.64E-119	1.06244158	0.974	0.776	2.47E-114	16
Rps4x7	4.27E-117	1.13201548	0.959	0.692	1.38E-112	16
Rpl327	1.28E-115	1.0606458	0.979	0.728	4.12E-111	16
Junb4	4.05E-110	1.32256146	0.831	0.428	1.31E-105	16
Smad7	1.47E-106	1.30921778	0.341	0.078	4.74E-102	16
Rps196	5.75E-106	1.11555743	0.974	0.729	1.86E-101	16
Id21	1.52E-104	1.50947205	0.299	0.065	4.90E-100	16
Rps235	5.34E-103	0.94543152	0.985	0.76	1.72E-98	16
Saraf	9.83E-99	1.46051497	0.305	0.07	3.17E-94	16
Dnajc152	1.25E-98	1.43878793	0.339	0.086	4.05E-94	16
Rpl22l16	3.82E-98	1.19566783	0.855	0.518	1.23E-93	16
Rpl376	2.56E-97	0.81743101	0.996	0.914	8.27E-93	16
Ptprc5	3.97E-96	1.46674631	0.618	0.267	1.28E-91	16
Btg13	2.80E-95	0.96534352	0.934	0.617	9.03E-91	16
Rps217	1.85E-92	1.0155103	0.938	0.627	5.96E-88	16
H2-Q6	4.48E-92	1.10666793	0.25	0.049	1.45E-87	16
Dusp23	3.92E-86	1.27980862	0.343	0.09	1.26E-81	16
Emb4	1.02E-84	1.57969576	0.433	0.15	3.28E-80	16
Rps3a16	9.81E-84	0.83931274	0.979	0.773	3.17E-79	16
Rpl228	1.54E-83	0.91280484	0.944	0.664	4.96E-79	16
Ccnd2	4.10E-83	1.21333093	0.305	0.076	1.32E-78	16
Rpl318	5.86E-83	1.02371718	0.885	0.555	1.89E-78	16
Rpl36a6	2.49E-81	0.94471292	0.915	0.58	8.03E-77	16
Gm100769	1.70E-77	0.81034382	0.974	0.808	5.49E-73	16
Rpl296	7.50E-76	0.99846469	0.863	0.572	2.42E-71	16
Rplp14	2.21E-75	0.75582761	0.987	0.705	7.12E-71	16
Rplp26	3.41E-73	0.82403251	0.953	0.713	1.10E-68	16
Pnrc14	2.55E-72	1.29787934	0.618	0.313	8.23E-68	16
Rpl10a7	5.59E-72	0.89211935	0.908	0.602	1.81E-67	16
Cd527	7.30E-68	0.82899186	0.895	0.511	2.36E-63	16
Rplp05	4.06E-67	0.84866375	0.947	0.737	1.31E-62	16
Rps218	1.00E-64	0.59254594	0.987	0.791	3.24E-60	16
Ptprcap4	3.21E-61	0.86574212	0.461	0.172	1.04E-56	16
H2-D15	5.43E-61	0.90299219	0.876	0.658	1.75E-56	16
Jak1	9.23E-60	1.31527555	0.386	0.15	2.98E-55	16
Rpl357	1.70E-58	0.72453058	0.989	0.772	5.49E-54	16
Rpl156	2.84E-57	0.77589763	0.911	0.659	9.18E-53	16
Mbnl14	5.55E-57	1.11586635	0.591	0.325	1.79E-52	16

mt-Nd4l6	4.46E-56	0.84900178	0.733	0.446	1.44E-51	16
Crip16	7.52E-56	0.63495572	0.759	0.395	2.43E-51	16
Rps97	8.45E-56	0.59975827	0.979	0.874	2.73E-51	16
Rpl246	1.50E-55	0.68248072	0.959	0.764	4.85E-51	16
Rpl84	3.49E-53	0.64471661	0.959	0.731	1.13E-48	16
Dusp51	9.19E-53	1.07584078	0.328	0.116	2.97E-48	16
Snrpg6	3.41E-52	0.7551081	0.832	0.503	1.10E-47	16
Dad15	1.62E-51	1.21193668	0.495	0.24	5.23E-47	16
Rps267	2.80E-49	0.62906062	0.974	0.786	9.06E-45	16
Selenow5	4.34E-48	0.90089292	0.531	0.262	1.40E-43	16
Ubb7	8.33E-47	0.6206424	0.934	0.758	2.69E-42	16
Nsa25	8.16E-46	0.8034139	0.734	0.479	2.63E-41	16
Psme16	1.58E-44	0.98054929	0.546	0.306	5.10E-40	16
Rpl418	3.34E-44	0.52661062	0.994	0.901	1.08E-39	16
Selplg3	6.56E-44	1.13444802	0.369	0.162	2.12E-39	16
Zfp36l12	8.08E-44	0.99663234	0.331	0.126	2.61E-39	16
Arl6ip51	1.22E-43	1.0651583	0.275	0.1	3.94E-39	16
B2m4	1.47E-42	0.5568678	0.881	0.709	4.75E-38	16
Pfdn55	3.60E-42	0.89340531	0.674	0.458	1.16E-37	16
Ndfip1	2.89E-41	1.10960225	0.384	0.178	9.33E-37	16
Rpl125	3.29E-41	0.53983097	0.853	0.535	1.06E-36	16
Tmsb4x7	1.02E-40	0.50356598	1	0.903	3.29E-36	16
Rpl107	1.23E-40	0.66969978	0.84	0.602	3.97E-36	16
Fxyd54	8.43E-40	0.62977838	0.627	0.367	2.72E-35	16
Rpl77	6.26E-39	0.63500168	0.866	0.631	2.02E-34	16
Cxcr44	1.58E-38	0.82077908	0.446	0.219	5.11E-34	16
Rpl144	1.83E-38	0.58664708	0.817	0.523	5.90E-34	16
Rpl64	2.49E-38	0.48078247	0.966	0.756	8.04E-34	16
Elf12	5.92E-37	0.96115363	0.32	0.137	1.91E-32	16
Gm118084	1.52E-34	1.01147444	0.352	0.171	4.90E-30	16
Mndal3	2.22E-34	0.87665705	0.284	0.117	7.16E-30	16
Tpt14	2.29E-34	0.35708476	0.979	0.865	7.40E-30	16
Psmb85	1.27E-33	0.98334921	0.407	0.214	4.11E-29	16
Itga43	1.88E-33	0.90101222	0.25	0.098	6.08E-29	16
Naca6	4.56E-33	0.58172402	0.821	0.571	1.47E-28	16
Eef1a14	1.47E-32	0.36468361	0.96	0.723	4.74E-28	16
Stk17b4	1.02E-30	0.71904428	0.529	0.318	3.28E-26	16
Ppia5	1.85E-30	0.39519984	0.942	0.708	5.97E-26	16
Eif14	3.46E-30	0.48365933	0.949	0.837	1.12E-25	16
Rpl36al7	7.21E-30	0.53826938	0.77	0.509	2.33E-25	16
Rps143	1.08E-29	0.40797019	0.928	0.714	3.50E-25	16
Tsc22d34	1.15E-29	0.81625798	0.356	0.176	3.71E-25	16
Leprotl1	1.98E-29	1.02526814	0.266	0.118	6.38E-25	16

Eef1b24	2.28E-29	0.5728348	0.765	0.534	7.38E-25	16
Fosb1	5.32E-29	0.88326578	0.313	0.144	1.72E-24	16
Ier24	2.11E-28	0.8813488	0.461	0.277	6.80E-24	16
Pabpc13	2.42E-28	0.54849694	0.759	0.584	7.80E-24	16
Uqcrh5	2.96E-27	0.59591143	0.691	0.513	9.56E-23	16
Rpl265	1.86E-26	0.40139417	0.925	0.701	6.02E-22	16
Ddx54	2.11E-25	0.71234739	0.682	0.548	6.80E-21	16
Jund4	5.03E-25	0.28898486	0.731	0.512	1.62E-20	16
Sub16	5.51E-25	0.71018503	0.653	0.479	1.78E-20	16
Tle42	1.23E-24	0.82568861	0.273	0.131	3.97E-20	16
Atp5e7	2.48E-24	0.47808717	0.836	0.697	8.00E-20	16
Rpl173	7.83E-24	0.31758708	0.876	0.669	2.53E-19	16
Ifi2033	1.15E-23	0.71078986	0.269	0.126	3.73E-19	16
Klf24	7.16E-23	0.83575037	0.384	0.225	2.31E-18	16
Tle53	8.45E-23	0.87819662	0.397	0.246	2.73E-18	16
Rac27	6.29E-22	0.63544878	0.588	0.408	2.03E-17	16
S100a103	2.52E-21	0.42962104	0.655	0.403	8.12E-17	16
Nfkbia5	3.26E-21	0.85897071	0.399	0.239	1.05E-16	16
Zc3hav1	4.28E-20	0.79441911	0.266	0.137	1.38E-15	16
Grcc103	4.82E-20	0.75886506	0.416	0.27	1.56E-15	16
Arhgap452	1.01E-19	0.79113155	0.29	0.158	3.27E-15	16
Bola25	3.63E-19	0.66387418	0.395	0.242	1.17E-14	16
Snhg14	4.00E-19	0.68849991	0.358	0.209	1.29E-14	16
Atp5h5	3.87E-18	0.56703802	0.629	0.475	1.25E-13	16
Mrpl526	6.26E-17	0.52161155	0.465	0.302	2.02E-12	16
H2-T231	7.56E-17	0.718153	0.298	0.173	2.44E-12	16
Cox8a5	1.11E-16	0.41283709	0.804	0.685	3.58E-12	16
Eif3f6	1.21E-16	0.59321288	0.433	0.289	3.90E-12	16
Slc38a24	1.56E-16	0.60666237	0.516	0.371	5.03E-12	16
Arhgef11	2.24E-15	0.79693047	0.254	0.145	7.22E-11	16
Atp5g26	3.23E-15	0.41378514	0.597	0.415	1.04E-10	16
Nop106	5.53E-15	0.52333396	0.395	0.252	1.78E-10	16
Rabac11	7.88E-15	0.7602537	0.322	0.207	2.54E-10	16
Tomm74	2.70E-14	0.45489405	0.633	0.483	8.72E-10	16
Rps64	2.84E-14	0.34964723	0.678	0.501	9.15E-10	16
mt-Atp86	5.26E-14	0.51805876	0.409	0.277	1.70E-09	16
Gstp14	6.49E-14	0.70535356	0.25	0.146	2.10E-09	16
Hspe12	7.23E-14	0.37167594	0.439	0.279	2.34E-09	16
Pfn15	1.19E-13	0.42155707	0.847	0.75	3.83E-09	16
Eif3e3	1.76E-13	0.47428119	0.354	0.226	5.67E-09	16
Tra2b5	2.31E-13	0.58273473	0.382	0.26	7.47E-09	16
Laptm53	3.43E-13	0.60135857	0.392	0.274	1.11E-08	16
Snhg82	3.68E-13	0.61398174	0.347	0.228	1.19E-08	16

Cytip5	4.17E-13	0.47463174	0.303	0.181	1.35E-08	16
Sh3bgrl38	1.13E-12	0.41125445	0.721	0.57	3.64E-08	16
Limd24	4.46E-12	0.58818057	0.335	0.228	1.44E-07	16
Vps281	4.46E-12	0.61590643	0.267	0.167	1.44E-07	16
Ndufa45	4.74E-12	0.34767197	0.623	0.465	1.53E-07	16
Akap136	6.49E-12	0.51846755	0.36	0.247	2.10E-07	16
Kdm6b2	7.04E-12	0.68907683	0.275	0.173	2.27E-07	16
Serf23	1.06E-11	0.35436857	0.836	0.727	3.43E-07	16
Atp5g14	1.78E-11	0.44163264	0.412	0.285	5.75E-07	16
mt-Nd25	1.04E-10	0.34862896	0.518	0.382	3.37E-06	16
Psmb94	1.13E-10	0.60011293	0.256	0.162	3.63E-06	16
Psme22	1.88E-09	0.6598072	0.269	0.185	6.07E-05	16
Trmt1125	2.39E-09	0.47739821	0.339	0.243	7.73E-05	16
Eif3h6	2.46E-09	0.37922861	0.382	0.277	7.96E-05	16
Gas53	3.33E-09	0.26147594	0.565	0.43	0.00010746	16
Dpm34	3.43E-09	0.50129722	0.35	0.255	0.00011058	16
Fam107b2	3.72E-09	0.66827545	0.298	0.214	0.00012012	16
Celf23	4.18E-09	0.53886884	0.271	0.184	0.0001351	16
Rpl7a6	6.18E-09	0.25622047	0.701	0.559	0.00019965	16
Tma74	1.09E-08	0.4117209	0.48	0.389	0.00035141	16
Coro1a6	1.46E-08	0.33638547	0.546	0.438	0.00047223	16
Park73	3.40E-08	0.4657392	0.32	0.232	0.00109808	16
Abracl4	4.14E-08	0.57427822	0.356	0.281	0.00133789	16
H2afj4	5.25E-08	0.4097398	0.524	0.433	0.00169514	16
Prrc2c2	7.56E-08	0.4277851	0.333	0.244	0.00243927	16
Atp5d5	8.74E-08	0.37451996	0.362	0.273	0.00282258	16
Clk15	9.91E-08	0.40645998	0.373	0.285	0.00320061	16
Klf131	1.27E-07	0.6350156	0.25	0.176	0.0041147	16
Cox143	2.69E-07	0.49702327	0.25	0.176	0.00867445	16
Xrn21	3.43E-07	0.37417844	0.252	0.172	0.01106164	16
Cd535	5.47E-07	0.41262664	0.337	0.26	0.01764406	16
Atp5mpl5	6.18E-07	0.31463071	0.465	0.376	0.01996413	16
Arpp194	6.37E-07	0.41258558	0.418	0.343	0.02055192	16
Srsf54	8.83E-07	0.37929093	0.456	0.384	0.02850841	16
Sap184	1.28E-06	0.57598946	0.296	0.233	0.04143232	16
Micos132	1.51E-06	0.47033712	0.277	0.205	0.04880568	16
Krtcap25	1.51E-06	0.2810684	0.347	0.255	0.0488735	16
Dnaja12	1.71E-06	0.46392068	0.427	0.351	0.05516417	16
Dusp113	1.79E-06	0.4432153	0.262	0.192	0.0578242	16
Taf104	2.29E-06	0.48069745	0.299	0.232	0.07389917	16
Ptp4a2	3.35E-06	0.42595654	0.277	0.204	0.10810921	16
Myl12b5	3.96E-06	0.29794756	0.497	0.424	0.12799163	16
Tagln25	4.96E-06	0.34904696	0.39	0.308	0.15999999	16

Klf62	1.09E-05	0.40651089	0.307	0.237	0.35065019	16
Edf15	1.46E-05	0.39136646	0.379	0.313	0.4700724	16
Sumo13	1.82E-05	0.44043834	0.282	0.223	0.58598375	16
Sell3	1.84E-05	0.48050382	0.254	0.192	0.59255593	16
Atp6v1f6	3.06E-05	0.39192885	0.38	0.325	0.98760356	16
Tomm65	3.10E-05	0.34005753	0.478	0.421	1	16
Anp32a3	4.19E-05	0.44141942	0.339	0.283	1	16
Emp34	4.31E-05	0.51184615	0.377	0.324	1	16
Ubald23	6.35E-05	0.46143391	0.254	0.196	1	16
Arl6ip15	6.73E-05	0.31773427	0.271	0.211	1	16
Ndufa18	6.80E-05	0.36573625	0.411	0.36	1	16
Ost45	0.00010234	0.38484949	0.403	0.351	1	16
Ndufb54	0.00013343	0.33802532	0.29	0.231	1	16
Ndufv34	0.00019726	0.44483057	0.299	0.255	1	16
Srrm22	0.000209	0.31142319	0.375	0.321	1	16
Tbca2	0.00025461	0.29990932	0.348	0.289	1	16
Ndufa54	0.00027923	0.30890315	0.262	0.207	1	16
S100a134	0.00028765	0.3788626	0.337	0.292	1	16
Ndufa133	0.00031763	0.32426564	0.467	0.431	1	16
Zc3h153	0.00032458	0.38384545	0.267	0.213	1	16
Ndufa34	0.00033598	0.34013607	0.446	0.411	1	16
Lsm43	0.00099384	0.34314579	0.273	0.227	1	16
Polr1d6	0.00207801	0.25955796	0.348	0.302	1	16
Srp94	0.00254266	0.34963201	0.298	0.265	1	16
Pak2	0.00938531	0.35361151	0.264	0.234	1	16
Srsf73	0.00956498	0.37297896	0.26	0.225	1	16
Ndufb105	0.00986803	0.28174685	0.281	0.246	1	16
Ctla2a1	0	4.03148926	0.874	0.022	0	17
Kit1	0	1.92268304	0.604	0.02	0	17
Nrgn	0	1.83283749	0.524	0.018	0	17
Vamp51	0	1.75751887	0.648	0.093	0	17
Lmo23	0	1.74907532	0.618	0.066	0	17
Myct1	0	1.6505522	0.407	0.002	0	17
Cdk61	0	1.534042	0.698	0.098	0	17
Angpt1	0	1.47698112	0.478	0.005	0	17
Adgrg11	0	1.47282723	0.538	0.022	0	17
Ctla2b	0	1.35192172	0.4	0.008	0	17
Nkg72	0	1.27100571	0.533	0.041	0	17
Snx141	0	1.20547844	0.439	0.032	0	17
Gata2	0	1.18363612	0.352	0.005	0	17
Dapp1	0	1.12958334	0.465	0.041	0	17
Runx3	0	1.04357045	0.396	0.031	0	17
Sdsl	0	1.02779007	0.334	0.004	0	17

Cd34	0	0.97416768	0.318	0.014	0	17
Muc13	0	0.95460941	0.304	0.003	0	17
Adgrl4	0	0.94332079	0.279	0.007	0	17
Gm15657	0	0.8889638	0.268	0.004	0	17
Slc22a3	0	0.83357441	0.268	0.002	0	17
Meis1	0	0.73257427	0.3	0.018	0	17
Nr4a21	8.41E-305	1.32957484	0.375	0.031	2.71E-300	17
Rgs11	6.18E-299	0.75886308	0.412	0.037	2.00E-294	17
Car22	8.65E-266	0.6971876	0.593	0.081	2.79E-261	17
Ifitm11	2.14E-264	1.84155535	0.648	0.104	6.92E-260	17
Myb4	2.21E-255	1.2407123	0.65	0.106	7.13E-251	17
1-Jun	7.06E-244	1.7889124	0.783	0.17	2.28E-239	17
Bcl11a	4.91E-237	0.88454011	0.366	0.038	1.59E-232	17
Bex31	2.67E-234	1.09344721	0.565	0.087	8.62E-230	17
Rpl328	6.78E-228	1.64867237	1	0.729	2.19E-223	17
Rps188	2.82E-221	1.71118321	1	0.647	9.09E-217	17
Klf10	2.16E-207	0.94348169	0.419	0.055	6.97E-203	17
Nr4a14	9.60E-207	1.95889354	0.748	0.196	3.10E-202	17
Nop583	1.15E-203	1.22005612	0.693	0.143	3.71E-199	17
Rps197	9.84E-203	1.52407044	0.998	0.73	3.18E-198	17
Ppif	1.95E-202	0.66197712	0.254	0.021	6.31E-198	17
1190007I07R	1.34E-201	0.82255703	0.384	0.049	4.34E-197	17
Ccnd21	7.29E-200	0.87074424	0.49	0.074	2.35E-195	17
Vkorc12	4.17E-197	0.72583535	0.391	0.051	1.35E-192	17
Rps248	3.76E-195	1.32878726	1	0.854	1.21E-190	17
Cmtm75	1.49E-194	1.35895658	0.851	0.231	4.82E-190	17
Rps27a7	2.10E-194	1.26722007	1	0.892	6.77E-190	17
Rps78	3.92E-193	1.45834469	1	0.728	1.26E-188	17
Rpl398	1.50E-192	1.32814324	1	0.845	4.83E-188	17
Mef2c4	1.77E-192	1.17485043	0.693	0.148	5.73E-188	17
Rpl97	2.15E-191	1.44356477	0.993	0.772	6.93E-187	17
Pitpnc1	3.84E-188	0.73351471	0.346	0.042	1.24E-183	17
Etfb3	2.14E-187	1.19119001	0.691	0.156	6.92E-183	17
Tspan321	3.18E-187	0.84354392	0.314	0.036	1.03E-182	17
Rpl368	5.51E-186	1.34551859	1	0.76	1.78E-181	17
Fosb2	1.13E-185	1.31661361	0.652	0.139	3.65E-181	17
Dctpp12	2.09E-185	1.05958857	0.57	0.107	6.76E-181	17
Pdcd45	6.04E-184	1.5615185	0.844	0.234	1.95E-179	17
Rgs184	1.50E-183	1.39457762	0.572	0.118	4.84E-179	17
Snhg41	3.30E-183	0.67912824	0.343	0.042	1.06E-178	17
Hint21	7.78E-181	0.78988897	0.389	0.055	2.51E-176	17
Rps15a7	1.07E-179	1.32512015	0.998	0.784	3.47E-175	17
Cox6b2	9.05E-177	0.79994711	0.256	0.025	2.92E-172	17

Etv61	4.55E-176	0.74388019	0.371	0.051	1.47E-171	17
Pam161	5.94E-175	0.87811603	0.462	0.077	1.92E-170	17
Rpl10a8	2.65E-173	1.43337446	0.998	0.602	8.56E-169	17
Snhg32	7.78E-171	1.14035078	0.604	0.128	2.51E-166	17
Rps207	1.59E-170	1.28640173	0.998	0.742	5.12E-166	17
Rps58	7.17E-170	1.34848308	1	0.68	2.31E-165	17
Rpl238	1.37E-169	1.1924124	1	0.823	4.44E-165	17
Rps4x8	1.76E-169	1.31710264	1	0.692	5.69E-165	17
Ssbp2	1.89E-169	0.66046919	0.284	0.032	6.10E-165	17
Hmgb32	4.47E-169	0.89739231	0.467	0.08	1.44E-164	17
Rps298	9.06E-169	1.08369819	1	0.954	2.92E-164	17
Rps219	1.24E-168	1.4377323	0.995	0.627	3.99E-164	17
Iqgap21	5.10E-166	0.7359475	0.348	0.048	1.65E-161	17
Erh6	3.67E-165	1.20091446	0.801	0.22	1.19E-160	17
Rps288	1.00E-164	1.11990239	1	0.845	3.23E-160	17
Ramp12	1.03E-164	1.28209201	0.533	0.109	3.34E-160	17
Rpl217	4.01E-164	1.28223148	1	0.717	1.29E-159	17
Snhg93	6.32E-164	1.02216434	0.613	0.132	2.04E-159	17
Snhg151	3.17E-161	0.84342542	0.391	0.06	1.02E-156	17
C1qbp2	3.24E-160	1.01075101	0.634	0.139	1.05E-155	17
Rpsa6	8.34E-160	1.27907099	1	0.7	2.69E-155	17
Zfp36l23	1.31E-158	1.29141037	0.835	0.253	4.22E-154	17
Pebp12	4.08E-158	0.95148018	0.767	0.194	1.32E-153	17
Gas54	9.84E-158	1.39903083	0.97	0.423	3.18E-153	17
Rpl319	3.52E-157	1.3656512	0.993	0.554	1.14E-152	17
H2-Q72	7.75E-156	0.60221489	0.499	0.09	2.50E-151	17
Rpl117	1.67E-155	1.23686498	1	0.738	5.38E-151	17
Khk1	4.08E-155	0.65281076	0.268	0.031	1.32E-150	17
Rps118	2.13E-154	1.23823964	0.998	0.728	6.88E-150	17
Rpl36a7	1.31E-153	1.30518413	0.998	0.58	4.21E-149	17
Nop107	2.00E-153	1.21222178	0.828	0.245	6.46E-149	17
Cmc12	2.73E-153	0.75685703	0.435	0.076	8.82E-149	17
Fam110a	1.34E-152	0.80776454	0.275	0.033	4.33E-148	17
Rpl188	2.23E-152	1.21845701	1	0.712	7.21E-148	17
Atpif15	6.08E-152	1.44872122	0.941	0.417	1.96E-147	17
Rpl287	8.54E-152	1.20558773	1	0.758	2.76E-147	17
Rpl388	1.18E-151	1.09124365	1	0.842	3.82E-147	17
Anks1	1.22E-151	0.67889485	0.359	0.054	3.94E-147	17
Gm472835	2.38E-151	0.89462128	0.668	0.159	7.68E-147	17
Serf1	1.14E-150	0.69135173	0.421	0.07	3.70E-146	17
Phpt11	2.18E-150	0.64666408	0.307	0.041	7.03E-146	17
Fermt32	2.57E-150	1.06665894	0.572	0.132	8.30E-146	17
Ddx211	2.97E-150	0.90531431	0.627	0.145	9.60E-146	17

Bzw21	6.01E-150	0.65851524	0.366	0.056	1.94E-145	17
Rpl22l17	8.94E-150	1.38034906	0.982	0.517	2.89E-145	17
Rpl157	1.60E-148	1.24353364	0.998	0.658	5.18E-144	17
Rpl136	1.01E-147	1.11936711	1	0.791	3.25E-143	17
Rpl358	1.78E-147	1.12773369	0.998	0.773	5.76E-143	17
Ier33	1.73E-146	0.98216599	0.604	0.141	5.58E-142	17
Rpl18a8	1.04E-145	1.10796378	1	0.805	3.35E-141	17
Rpl35a8	1.31E-144	1.02366123	0.998	0.855	4.24E-140	17
Skil1	2.27E-144	1.21583101	0.68	0.181	7.34E-140	17
Ppan1	2.78E-144	0.59401767	0.277	0.035	8.97E-140	17
Egr13	2.79E-144	1.18755312	0.664	0.173	9.02E-140	17
Rpl37a8	1.32E-143	1.02650355	1	0.904	4.27E-139	17
Cbfa2t3	3.41E-143	0.54238269	0.295	0.039	1.10E-138	17
Smad71	1.29E-142	0.75394587	0.435	0.078	4.15E-138	17
Elf13	3.21E-142	0.92388546	0.581	0.133	1.03E-137	17
Rpl308	4.18E-142	1.08583262	1	0.816	1.35E-137	17
Msi2	9.00E-142	0.70701593	0.43	0.076	2.91E-137	17
Rpl297	1.12E-141	1.24835017	0.989	0.57	3.61E-137	17
Rps137	1.38E-141	1.10637814	1	0.794	4.46E-137	17
Eif3i4	2.37E-141	0.78801738	0.67	0.165	7.64E-137	17
Gabarapl1	7.50E-141	0.50726665	0.27	0.033	2.42E-136	17
AI6622706	8.60E-141	0.83647375	0.577	0.133	2.78E-136	17
Rps38	4.53E-140	1.17770924	0.993	0.705	1.46E-135	17
Park74	1.52E-138	1.04728842	0.762	0.224	4.89E-134	17
Rpl145	7.81E-138	1.25584677	0.986	0.521	2.52E-133	17
Ninj11	2.08E-137	0.76168775	0.471	0.094	6.73E-133	17
Marcksl11	5.15E-137	0.91748388	0.497	0.105	1.66E-132	17
Gse1	1.94E-136	0.56828168	0.336	0.051	6.26E-132	17
Snhg16	4.78E-136	0.95987188	0.735	0.202	1.54E-131	17
Uba527	9.03E-136	1.18855552	0.995	0.756	2.91E-131	17
Rpl266	1.55E-135	1.14352796	1	0.701	5.01E-131	17
Gm26532	1.81E-133	0.73092361	0.348	0.056	5.84E-129	17
Stmn14	1.85E-132	1.12247809	0.808	0.254	5.97E-128	17
5430416N02	5.50E-132	0.61492554	0.295	0.042	1.77E-127	17
Smim271	6.35E-132	0.76506954	0.435	0.086	2.05E-127	17
Nfkbia6	1.32E-131	0.96723184	0.773	0.232	4.25E-127	17
Tagln26	1.60E-130	1.08158899	0.879	0.3	5.17E-126	17
Fbl1	1.97E-130	0.68901318	0.501	0.106	6.36E-126	17
Spint21	2.39E-130	0.66674287	0.38	0.067	7.71E-126	17
Rps3a17	2.57E-130	1.04808676	1	0.773	8.31E-126	17
Ier25	2.59E-130	1.18969416	0.801	0.272	8.35E-126	17
Mrpl233	2.33E-129	0.72928112	0.588	0.141	7.51E-125	17
Rpl348	4.24E-129	1.00438447	1	0.825	1.37E-124	17

Snhg83	6.42E-129	0.81558399	0.767	0.221	2.07E-124	17
Cenpx3	7.42E-129	0.84291814	0.545	0.13	2.39E-124	17
Rps268	3.25E-128	1.07835364	0.998	0.786	1.05E-123	17
Kdm6b3	9.19E-128	1.03232416	0.622	0.167	2.97E-123	17
H2afy5	5.96E-127	1.42639996	0.737	0.235	1.92E-122	17
Rpl197	1.07E-126	1.04061822	1	0.759	3.46E-122	17
Lsm73	3.10E-126	0.82641038	0.593	0.146	1.00E-121	17
Atp5g15	1.63E-125	1.10856341	0.826	0.278	5.25E-121	17
Naca7	3.42E-125	1.1469645	0.989	0.569	1.10E-120	17
Rpl377	7.49E-125	0.9276568	1	0.915	2.42E-120	17
Hnrnpa15	3.01E-124	1.03128063	0.819	0.269	9.72E-120	17
Rpl229	1.40E-123	1.07288445	0.998	0.664	4.51E-119	17
Gm118085	3.76E-123	0.78779634	0.629	0.166	1.21E-118	17
Plac83	1.29E-122	1.41487409	0.767	0.271	4.17E-118	17
Rps236	4.10E-122	1.03226884	0.998	0.761	1.32E-117	17
Tnfaip31	6.22E-122	0.80045674	0.394	0.074	2.01E-117	17
Rpl27a7	8.86E-122	0.99313986	0.998	0.799	2.86E-117	17
Fmc11	3.02E-121	0.71597652	0.481	0.108	9.76E-117	17
Acyp1	3.96E-121	0.6815205	0.364	0.067	1.28E-116	17
Syce21	4.55E-120	0.61061825	0.327	0.055	1.47E-115	17
Rsl1d11	6.19E-120	0.63995056	0.428	0.086	2.00E-115	17
Apex11	2.88E-118	0.54851287	0.357	0.064	9.29E-114	17
Fut8	3.09E-117	0.51148733	0.261	0.037	9.97E-113	17
Naa101	4.54E-117	0.61187941	0.357	0.066	1.46E-112	17
Micos133	1.67E-116	0.7852429	0.682	0.198	5.40E-112	17
Suclg2	1.98E-116	0.46383573	0.265	0.038	6.39E-112	17
Tgfb13	8.88E-116	0.72096294	0.588	0.154	2.87E-111	17
Trp53	1.94E-115	0.6995491	0.458	0.1	6.26E-111	17
Eif5a4	5.25E-115	1.07918965	0.938	0.401	1.69E-110	17
Fabp53	1.32E-114	0.77025319	0.469	0.106	4.26E-110	17
Hscb	3.10E-114	0.48414184	0.256	0.037	1.00E-109	17
Cdkn1a1	2.03E-112	1.01685946	0.414	0.09	6.54E-108	17
Trmt1126	3.23E-112	0.86770061	0.746	0.236	1.04E-107	17
Srm1	3.54E-112	0.54089068	0.33	0.058	1.14E-107	17
Wdr121	3.87E-112	0.57908148	0.265	0.04	1.25E-107	17
Rps65	3.05E-111	1.10072643	0.963	0.496	9.85E-107	17
Ssr45	3.44E-111	0.85699255	0.762	0.244	1.11E-106	17
Rplp06	7.37E-111	0.99841402	1	0.737	2.38E-106	17
Rps169	2.52E-110	0.94284418	0.998	0.776	8.15E-106	17
Pts1	2.59E-110	0.63368939	0.357	0.069	8.36E-106	17
Ppie1	2.67E-110	0.4941197	0.27	0.042	8.61E-106	17
Tsc22d2	8.00E-110	0.50398687	0.316	0.054	2.58E-105	17
Rpl108	1.63E-109	1.06659565	0.986	0.6	5.25E-105	17

Llph4	2.02E-109	0.77234788	0.661	0.192	6.53E-105	17
Rps108	2.26E-109	0.93646111	1	0.802	7.31E-105	17
Tsc22d1	2.53E-109	0.75775582	0.4	0.081	8.16E-105	17
Cdk43	1.10E-108	0.69797733	0.542	0.137	3.56E-104	17
Ppp1r15a2	1.46E-107	0.48241868	0.483	0.111	4.70E-103	17
Mrps282	2.49E-107	0.60832793	0.389	0.081	8.03E-103	17
Pigp	2.59E-107	0.47883277	0.265	0.041	8.36E-103	17
Zfpm12	1.41E-106	0.59117078	0.291	0.048	4.57E-102	17
Tmem2231	5.22E-106	0.60339239	0.343	0.067	1.69E-101	17
Exosc51	8.08E-106	0.54064113	0.311	0.056	2.61E-101	17
Bola11	1.52E-105	0.55689283	0.343	0.066	4.89E-101	17
Epb41l4aos	7.27E-105	0.52846253	0.334	0.062	2.35E-100	17
Mrpl527	7.61E-105	0.90480288	0.84	0.295	2.46E-100	17
Rpl247	3.80E-104	0.92505116	0.998	0.764	1.23E-99	17
Rfc22	1.07E-103	0.57473961	0.396	0.085	3.44E-99	17
Hmgn52	2.37E-103	0.54631008	0.341	0.066	7.65E-99	17
Nudc2	3.02E-103	0.65463702	0.51	0.129	9.74E-99	17
Ptpn185	3.94E-103	0.90053863	0.794	0.288	1.27E-98	17
Bola26	5.10E-103	0.79113219	0.741	0.236	1.65E-98	17
Rcc21	1.08E-102	0.50746475	0.439	0.1	3.48E-98	17
Psmb86	1.20E-102	0.70669633	0.684	0.21	3.87E-98	17
2310039H08	1.36E-102	0.54325174	0.311	0.057	4.39E-98	17
Gstp15	2.64E-102	0.67090455	0.535	0.141	8.52E-98	17
Atp5g27	4.20E-102	1.05053386	0.918	0.41	1.36E-97	17
Mtln1	6.79E-102	0.55098915	0.362	0.073	2.19E-97	17
Csrp11	7.63E-102	0.6924667	0.378	0.08	2.46E-97	17
Pa2g42	1.40E-101	0.65884417	0.513	0.131	4.51E-97	17
Nrip1	4.97E-101	0.49996587	0.339	0.064	1.60E-96	17
Cdca72	6.25E-101	0.62669548	0.336	0.066	2.02E-96	17
Snrpg7	3.98E-100	0.98797346	0.977	0.502	1.29E-95	17
Smim114	6.44E-100	0.64254539	0.513	0.135	2.08E-95	17
Psmg42	9.64E-100	0.58926727	0.46	0.111	3.11E-95	17
Btg3	1.79E-99	0.56949813	0.346	0.069	5.79E-95	17
Trappc6a1	2.55E-99	0.54447433	0.334	0.067	8.24E-95	17
Snrpd14	4.72E-99	0.82880722	0.757	0.253	1.52E-94	17
H2-Q61	6.06E-99	0.38320726	0.288	0.049	1.96E-94	17
Dusp24	1.91E-98	0.6060784	0.407	0.09	6.17E-94	17
Ndufab14	3.42E-98	0.73738817	0.654	0.2	1.10E-93	17
Pbx1	3.77E-98	0.76653093	0.339	0.067	1.22E-93	17
Zc3h12a	5.38E-98	0.52692315	0.314	0.059	1.74E-93	17
Taok3	5.44E-98	0.59854396	0.33	0.065	1.76E-93	17
Nifk1	1.86E-97	0.5511296	0.325	0.064	6.01E-93	17
Ncl4	2.13E-97	1.00921138	0.927	0.409	6.88E-93	17

Rpl174	2.48E-97	0.91179883	0.993	0.667	8.00E-93	17
Ndufa46	2.54E-97	1.09448217	0.938	0.459	8.19E-93	17
Dpm35	9.74E-97	0.78736583	0.732	0.249	3.15E-92	17
Eef1b25	1.93E-96	0.9577142	0.979	0.531	6.23E-92	17
Hspe13	2.44E-96	0.88771049	0.785	0.273	7.86E-92	17
Hk1	4.48E-95	0.52699227	0.311	0.06	1.45E-90	17
Tmem176b1	8.01E-95	0.54228195	0.469	0.114	2.59E-90	17
H2-DMa2	2.37E-94	0.44072444	0.348	0.072	7.65E-90	17
Spn1	2.45E-94	0.54743832	0.259	0.045	7.91E-90	17
Rpl278	3.69E-94	0.88327984	0.995	0.722	1.19E-89	17
Ankrd13a	4.90E-94	0.57640948	0.378	0.085	1.58E-89	17
Tut42	2.16E-93	0.44543398	0.446	0.108	6.97E-89	17
Arid1b	6.41E-93	0.40578046	0.339	0.069	2.07E-88	17
Snrpf5	1.50E-92	0.87152153	0.918	0.382	4.84E-88	17
2410006H16I	1.72E-92	0.92942205	0.906	0.393	5.55E-88	17
Nucb11	3.19E-92	0.51584973	0.373	0.084	1.03E-87	17
Creg14	9.09E-92	0.64483524	0.531	0.15	2.94E-87	17
Ppia6	1.01E-91	0.91688349	1	0.708	3.26E-87	17
Ruvbl1	1.32E-91	0.52531635	0.355	0.076	4.26E-87	17
Cd692	1.55E-91	0.88941529	0.368	0.083	5.01E-87	17
Polr2l3	1.74E-91	0.64453172	0.481	0.127	5.61E-87	17
Exosc71	2.12E-91	0.4237173	0.314	0.062	6.84E-87	17
Gm1007610	2.69E-91	0.85051065	1	0.808	8.69E-87	17
Pan31	3.01E-91	0.58417006	0.476	0.126	9.71E-87	17
Prmt12	5.90E-91	0.56530725	0.478	0.124	1.91E-86	17
Cirbp1	5.98E-91	0.55399885	0.403	0.096	1.93E-86	17
Rps98	1.02E-90	0.76236348	0.995	0.874	3.31E-86	17
Aamp1	2.56E-90	0.58336095	0.471	0.125	8.26E-86	17
Uqcrb5	3.44E-90	0.81023611	0.822	0.316	1.11E-85	17
Eif3f7	9.00E-90	0.64176886	0.801	0.283	2.90E-85	17
Eif3e4	3.57E-89	0.67343457	0.675	0.22	1.15E-84	17
Dad16	4.34E-89	0.6928335	0.707	0.237	1.40E-84	17
1110038B12I	8.52E-89	0.55246752	0.604	0.179	2.75E-84	17
Mrpl544	8.82E-89	0.49789141	0.503	0.137	2.85E-84	17
Thoc74	1.40E-88	0.68793112	0.616	0.193	4.51E-84	17
Cd482	2.63E-88	0.58976247	0.332	0.071	8.48E-84	17
Mrpl122	3.36E-88	0.57645204	0.407	0.099	1.09E-83	17
Fundc22	3.60E-88	0.52626833	0.49	0.132	1.16E-83	17
Rpl78	3.80E-88	0.88307493	0.991	0.63	1.23E-83	17
Ptprcap5	3.89E-88	0.28576816	0.606	0.171	1.26E-83	17
Nme13	4.25E-88	0.71145814	0.707	0.232	1.37E-83	17
Rps27l6	4.54E-88	0.83665493	0.87	0.341	1.47E-83	17
Ndufb24	1.52E-87	0.66781199	0.668	0.219	4.92E-83	17

Glipr12	7.95E-87	0.63413509	0.403	0.098	2.57E-82	17
Cbx34	8.92E-87	0.82667223	0.84	0.334	2.88E-82	17
Phb1	3.24E-86	0.46577787	0.323	0.068	1.05E-81	17
Rnf138	4.91E-86	0.45698371	0.252	0.044	1.58E-81	17
Tmco13	6.68E-86	0.72293193	0.519	0.154	2.16E-81	17
Rsl24d11	6.92E-86	0.49977426	0.348	0.078	2.23E-81	17
Mat2a	1.14E-85	0.63607309	0.398	0.099	3.68E-81	17
Gnl31	1.20E-85	0.49701132	0.407	0.098	3.87E-81	17
Set4	1.87E-85	0.78318019	0.872	0.343	6.04E-81	17
Snrpd24	1.93E-85	0.77716589	0.787	0.295	6.24E-81	17
Glrx31	2.08E-85	0.51114154	0.41	0.101	6.73E-81	17
Skp1a3	3.14E-85	0.69408196	0.65	0.218	1.01E-80	17
Sptssa3	4.50E-85	0.56101219	0.423	0.11	1.45E-80	17
Lage31	8.46E-85	0.45430667	0.32	0.069	2.73E-80	17
Srsf74	1.20E-84	0.62885171	0.659	0.218	3.86E-80	17
Uqcr116	2.18E-84	0.84852045	0.842	0.354	7.03E-80	17
Psmb24	2.50E-84	0.58482685	0.668	0.222	8.06E-80	17
Cbx12	4.25E-84	0.53395327	0.577	0.174	1.37E-79	17
Polr2e	8.81E-84	0.51513916	0.405	0.101	2.84E-79	17
Alyref5	1.20E-83	0.71238885	0.659	0.22	3.87E-79	17
Gar1	2.10E-83	0.48532393	0.279	0.054	6.77E-79	17
Snu134	3.09E-83	0.68023779	0.744	0.266	9.98E-79	17
Tra2b6	8.06E-83	0.67537948	0.705	0.254	2.60E-78	17
Saraf1	8.49E-83	0.49015763	0.325	0.07	2.74E-78	17
Bcl7c2	8.51E-83	0.46665791	0.371	0.089	2.75E-78	17
Ptma5	2.78E-82	0.72755333	0.998	0.688	8.97E-78	17
Pde4b1	3.32E-82	0.53571567	0.394	0.097	1.07E-77	17
Ptges33	7.81E-82	0.72403908	0.661	0.226	2.52E-77	17
Rps84	1.05E-81	0.80170116	0.998	0.745	3.38E-77	17
Cnbp2	1.45E-81	0.50626004	0.652	0.211	4.69E-77	17
Rpgrip14	3.06E-81	0.5676709	0.437	0.118	9.89E-77	17
Dtnbp11	3.38E-81	0.50060937	0.297	0.063	1.09E-76	17
Ifrd12	5.25E-81	0.66909469	0.526	0.159	1.70E-76	17
Paics1	5.50E-81	0.41398238	0.449	0.119	1.78E-76	17
Tap2	8.61E-81	0.48470737	0.263	0.051	2.78E-76	17
Rplp27	1.61E-80	0.80530836	0.993	0.713	5.20E-76	17
Zfp5931	9.50E-80	0.44797323	0.259	0.05	3.07E-75	17
Polr2f2	3.12E-79	0.50340619	0.535	0.159	1.01E-74	17
Fam133b1	3.16E-79	0.54878289	0.437	0.119	1.02E-74	17
Zfp363	3.31E-79	0.66401135	0.641	0.224	1.07E-74	17
Glul	3.32E-79	0.63938836	0.3	0.064	1.07E-74	17
Suclg11	4.83E-79	0.47651382	0.366	0.09	1.56E-74	17
Pabpc14	2.11E-78	0.83621641	0.968	0.581	6.83E-74	17

Trim281	7.16E-78	0.4459689	0.414	0.108	2.31E-73	17
Nenf	1.37E-77	0.35872025	0.304	0.065	4.43E-73	17
Timm8a11	2.27E-77	0.43301322	0.272	0.055	7.32E-73	17
Atp5d6	6.01E-77	0.56333982	0.737	0.267	1.94E-72	17
Bclaf1	9.31E-77	0.52087942	0.494	0.146	3.01E-72	17
Rpl65	1.06E-76	0.75109363	1	0.756	3.41E-72	17
Commd71	1.09E-76	0.51124601	0.311	0.07	3.51E-72	17
Sox45	1.23E-76	0.39669658	0.533	0.154	3.97E-72	17
Cux11	1.27E-76	0.48175557	0.478	0.138	4.11E-72	17
Drap12	1.36E-76	0.47809754	0.593	0.19	4.40E-72	17
Gspt11	1.38E-76	0.3920853	0.394	0.101	4.44E-72	17
Cks24	2.00E-76	0.98068925	0.664	0.253	6.45E-72	17
Cct31	2.00E-76	0.29291219	0.474	0.131	6.45E-72	17
Nhp22	2.96E-76	0.43463779	0.476	0.134	9.56E-72	17
Abcf12	3.24E-76	0.4776232	0.517	0.155	1.05E-71	17
Gtf2i	3.26E-76	0.34739558	0.284	0.059	1.05E-71	17
Bin11	7.99E-76	0.46304672	0.27	0.055	2.58E-71	17
1110004F10I	1.02E-75	0.55786851	0.563	0.18	3.30E-71	17
Arhgef2	1.26E-75	0.47221006	0.332	0.078	4.08E-71	17
Mybbp1a1	1.46E-75	0.37599486	0.414	0.108	4.72E-71	17
Tob2	2.17E-75	0.51147561	0.268	0.055	6.99E-71	17
Naa384	2.44E-75	0.4759342	0.517	0.157	7.88E-71	17
Cuta4	2.87E-75	0.55020918	0.533	0.169	9.25E-71	17
Serbp15	9.14E-75	0.83292334	0.945	0.482	2.95E-70	17
Utp111	1.75E-74	0.47368085	0.382	0.099	5.66E-70	17
Ndufb55	2.46E-74	0.52217503	0.654	0.224	7.93E-70	17
C1d1	3.02E-74	0.46268279	0.368	0.095	9.75E-70	17
Idh21	3.10E-74	0.38722239	0.332	0.078	1.00E-69	17
Nfkbid	4.24E-74	0.42221121	0.362	0.09	1.37E-69	17
Phf14	5.56E-74	0.41987159	0.311	0.071	1.80E-69	17
Tmem176a	6.78E-74	0.26683969	0.323	0.073	2.19E-69	17
Mrto41	7.40E-74	0.37325153	0.311	0.071	2.39E-69	17
Ndufaf21	1.50E-73	0.3824779	0.307	0.069	4.83E-69	17
Emc62	1.60E-73	0.40071124	0.449	0.127	5.18E-69	17
Phgdh1	1.78E-73	0.34768549	0.387	0.098	5.76E-69	17
Jund5	2.01E-73	0.5896996	0.941	0.509	6.48E-69	17
Dkc1	2.11E-73	0.37993211	0.27	0.056	6.83E-69	17
Rpl419	2.20E-73	0.69789551	1	0.901	7.11E-69	17
Metap24	2.97E-73	0.50900525	0.675	0.238	9.59E-69	17
Mif4	5.08E-73	0.663441	0.835	0.335	1.64E-68	17
Smim42	5.69E-73	0.55666483	0.517	0.166	1.84E-68	17
Mcm63	1.17E-72	0.46432112	0.355	0.089	3.79E-68	17
Camta11	1.61E-72	0.40901055	0.281	0.061	5.20E-68	17

Sf3b64	2.65E-72	0.60070435	0.698	0.259	8.56E-68	17
Ndufc24	1.81E-71	0.56287803	0.634	0.223	5.83E-67	17
Hp1bp3	2.39E-71	0.42717942	0.474	0.14	7.72E-67	17
Adk1	3.99E-71	0.40703706	0.281	0.062	1.29E-66	17
B930036N10	5.10E-71	0.58324106	0.304	0.072	1.65E-66	17
Zfand51	7.00E-71	0.58825931	0.563	0.189	2.26E-66	17
Tomm75	1.37E-70	0.7987785	0.918	0.478	4.41E-66	17
Mtdh1	1.44E-70	0.44899535	0.529	0.168	4.66E-66	17
Snrpb6	2.18E-70	0.58267897	0.723	0.273	7.04E-66	17
Polr2h1	3.23E-70	0.38893555	0.275	0.06	1.04E-65	17
Fam117a1	3.78E-70	0.42448705	0.27	0.058	1.22E-65	17
Dcun1d1	1.09E-69	0.41355254	0.272	0.059	3.53E-65	17
Ddx39b3	1.27E-69	0.43012618	0.545	0.176	4.09E-65	17
Ccnl14	1.33E-69	0.55202567	0.659	0.244	4.30E-65	17
U2af13	2.90E-69	0.53568657	0.616	0.218	9.36E-65	17
Mrps144	4.14E-69	0.47214399	0.563	0.187	1.34E-64	17
Tmem1471	5.32E-69	0.41251001	0.311	0.075	1.72E-64	17
Ythdf2	5.80E-69	0.44693214	0.295	0.069	1.87E-64	17
Eny24	6.73E-69	0.43730494	0.558	0.182	2.17E-64	17
Dtymk4	9.07E-69	0.50778617	0.476	0.146	2.93E-64	17
Lsm44	1.28E-68	0.48293992	0.634	0.221	4.14E-64	17
Gtf3a1	1.30E-68	0.34306684	0.3	0.069	4.19E-64	17
Tomm204	1.81E-68	0.46861298	0.698	0.253	5.84E-64	17
Bola34	2.11E-68	0.4573142	0.416	0.118	6.82E-64	17
Mrpl321	3.06E-68	0.41672495	0.382	0.105	9.88E-64	17
Wdr61	5.64E-68	0.40561859	0.316	0.077	1.82E-63	17
Ndufs74	1.52E-67	0.50417297	0.538	0.18	4.92E-63	17
Sod22	1.90E-67	0.4559979	0.407	0.117	6.15E-63	17
Swi55	2.07E-67	0.47475191	0.668	0.242	6.70E-63	17
Selenow6	2.30E-67	0.38658176	0.723	0.259	7.43E-63	17
Cox144	2.59E-67	0.48184152	0.519	0.171	8.37E-63	17
Bag11	3.46E-67	0.44892963	0.497	0.158	1.12E-62	17
Ppp1r10	4.08E-67	0.36775637	0.334	0.085	1.32E-62	17
Snrpa12	4.24E-67	0.41755489	0.348	0.091	1.37E-62	17
Pabpn11	4.48E-67	0.32268951	0.451	0.134	1.45E-62	17
Rps275	5.21E-67	0.53888413	0.998	0.911	1.68E-62	17
Gatad1	5.57E-67	0.48921068	0.416	0.122	1.80E-62	17
AC149090.1	6.02E-67	0.35865556	0.327	0.081	1.94E-62	17
Hypk1	6.96E-67	0.4758806	0.346	0.092	2.25E-62	17
Pole42	7.57E-67	0.40698851	0.366	0.099	2.44E-62	17
Pnrc15	7.87E-67	0.57256448	0.757	0.311	2.54E-62	17
Ube2e31	9.05E-67	0.37425171	0.32	0.079	2.92E-62	17
Gt(ROSA)26	1.16E-66	0.41314599	0.279	0.064	3.75E-62	17

H2afv4	2.01E-66	0.26659258	0.748	0.279	6.49E-62	17
Pim15	2.01E-66	0.65879594	0.78	0.334	6.49E-62	17
Mrpl361	2.10E-66	0.38666626	0.382	0.106	6.77E-62	17
Prdx66	2.92E-66	0.68685861	0.705	0.279	9.44E-62	17
Ndufb44	1.05E-65	0.50815406	0.641	0.235	3.40E-61	17
Tomm401	1.13E-65	0.3526022	0.256	0.056	3.64E-61	17
Rbmxl12	1.26E-65	0.4189882	0.291	0.07	4.06E-61	17
Luc7l	1.78E-65	0.26876728	0.272	0.061	5.76E-61	17
Mrps334	2.38E-65	0.49658365	0.556	0.191	7.67E-61	17
Lgals92	3.49E-65	0.37022871	0.451	0.136	1.13E-60	17
Sumo25	4.56E-65	0.61155854	0.817	0.344	1.47E-60	17
Ddx18	6.21E-65	0.30432215	0.348	0.09	2.00E-60	17
Impdh21	6.30E-65	0.33688417	0.41	0.117	2.03E-60	17
Thoc1	7.32E-65	0.33631827	0.284	0.066	2.36E-60	17
Ewsr13	8.37E-65	0.3331217	0.497	0.158	2.70E-60	17
Wdr43	9.53E-65	0.35616079	0.38	0.104	3.08E-60	17
Gclm2	9.65E-65	0.44357798	0.375	0.104	3.12E-60	17
Plgrkt	1.87E-64	0.38475825	0.314	0.079	6.03E-60	17
Nfkb1	3.23E-64	0.46213888	0.343	0.092	1.04E-59	17
Wdr83os3	3.30E-64	0.46672673	0.503	0.166	1.07E-59	17
Larp71	3.63E-64	0.38689601	0.382	0.108	1.17E-59	17
Cdc371	4.18E-64	0.45128245	0.453	0.141	1.35E-59	17
Slc25a55	7.26E-64	0.50415328	0.693	0.264	2.34E-59	17
Rheb2	8.72E-64	0.48292452	0.421	0.129	2.81E-59	17
G3bp11	1.03E-63	0.38046856	0.462	0.143	3.34E-59	17
Chchd25	1.10E-63	0.74538471	0.954	0.577	3.56E-59	17
Tmem2565	1.34E-63	0.55829366	0.698	0.27	4.32E-59	17
Atp5mpl6	1.73E-63	0.607064	0.844	0.369	5.58E-59	17
Map2k3	1.81E-63	0.48315917	0.348	0.094	5.84E-59	17
Ubb8	2.50E-63	0.7203004	0.984	0.758	8.06E-59	17
Sec61b5	2.71E-63	0.69198556	0.902	0.442	8.75E-59	17
Ifitm24	3.18E-63	0.54113282	0.908	0.451	1.03E-58	17
Nsa26	3.37E-63	0.6643814	0.938	0.477	1.09E-58	17
Emg13	4.33E-63	0.37399447	0.471	0.149	1.40E-58	17
Rpl36al8	4.61E-63	0.71899013	0.961	0.507	1.49E-58	17
Ndufa123	8.36E-63	0.40085473	0.476	0.151	2.70E-58	17
Eif4a13	9.32E-63	0.57537113	0.817	0.352	3.01E-58	17
Npm31	1.35E-62	0.29693913	0.364	0.099	4.35E-58	17
Imp3	2.02E-62	0.34443455	0.357	0.098	6.52E-58	17
Supt164	2.09E-62	0.48132805	0.497	0.164	6.75E-58	17
Higd1a3	2.75E-62	0.39963815	0.533	0.181	8.88E-58	17
Srsf62	4.28E-62	0.39750804	0.485	0.159	1.38E-57	17
Eif3k5	4.39E-62	0.54375047	0.741	0.305	1.42E-57	17

Aimp13	4.87E-62	0.38893179	0.581	0.203	1.57E-57	17
Caprin11	6.10E-62	0.4112238	0.494	0.162	1.97E-57	17
Sik11	6.29E-62	0.47365364	0.414	0.125	2.03E-57	17
Fkbp34	9.06E-62	0.35681024	0.503	0.163	2.93E-57	17
Ranbp13	9.38E-62	0.49108447	0.648	0.236	3.03E-57	17
Mndal4	1.36E-61	0.3013129	0.4	0.115	4.41E-57	17
Ptprs	1.78E-61	0.26521341	0.297	0.072	5.75E-57	17
Paip23	1.79E-61	0.48006038	0.659	0.255	5.79E-57	17
Snrpd35	2.73E-61	0.53854415	0.677	0.262	8.82E-57	17
Mrpl304	3.46E-61	0.44427285	0.515	0.175	1.12E-56	17
Tmem1602	3.97E-61	0.42048821	0.442	0.14	1.28E-56	17
Npm13	4.31E-61	0.5409835	0.947	0.465	1.39E-56	17
Srpk1	5.92E-61	0.38169253	0.302	0.077	1.91E-56	17
Srsf25	8.69E-61	0.45764666	0.668	0.255	2.80E-56	17
Rps144	9.75E-61	0.62518619	0.998	0.714	3.15E-56	17
Brd11	1.03E-60	0.36508869	0.366	0.104	3.33E-56	17
Prpf4b4	1.08E-60	0.47352259	0.638	0.242	3.48E-56	17
Gtpbp4	1.66E-60	0.42029693	0.316	0.083	5.35E-56	17
21-Mar	1.99E-60	0.34886287	0.3	0.076	6.42E-56	17
Mplkip1	2.55E-60	0.34250055	0.3	0.076	8.24E-56	17
Rnf73	3.35E-60	0.38284632	0.467	0.152	1.08E-55	17
Rbm26	4.17E-60	0.34112789	0.288	0.071	1.35E-55	17
Rabgap1l1	4.91E-60	0.43200667	0.325	0.087	1.59E-55	17
Rbbp72	4.95E-60	0.36104947	0.437	0.135	1.60E-55	17
Zcchc7	4.97E-60	0.29898074	0.357	0.1	1.60E-55	17
Tmbim42	6.86E-60	0.4603965	0.421	0.134	2.21E-55	17
Rpl7a7	7.82E-60	0.68083497	0.97	0.555	2.52E-55	17
Psme17	9.93E-60	0.45721001	0.741	0.303	3.21E-55	17
Mrpl343	1.05E-59	0.33864189	0.497	0.165	3.38E-55	17
Ivns1abp	1.14E-59	0.32491686	0.311	0.081	3.67E-55	17
Lsm33	2.64E-59	0.52543478	0.458	0.152	8.51E-55	17
Dnajc22	2.79E-59	0.28804931	0.378	0.109	9.01E-55	17
Ddx241	3.09E-59	0.31322799	0.432	0.135	9.97E-55	17
Rbbp6	3.10E-59	0.3921217	0.368	0.106	1.00E-54	17
Cdc262	4.62E-59	0.35368018	0.352	0.1	1.49E-54	17
Eif3h7	5.60E-59	0.3596121	0.703	0.271	1.81E-54	17
Runx11	6.35E-59	0.35348573	0.396	0.118	2.05E-54	17
Ndufa84	6.90E-59	0.43914088	0.501	0.174	2.23E-54	17
Cisd23	8.48E-59	0.38966784	0.43	0.137	2.74E-54	17
Cycs5	8.85E-59	0.52978449	0.622	0.237	2.86E-54	17
Gadd45b	1.11E-58	0.44217333	0.327	0.09	3.59E-54	17
Grcc104	1.26E-58	0.47571801	0.67	0.265	4.05E-54	17
Emp35	2.47E-58	0.5699321	0.748	0.318	7.99E-54	17

Osgep1	3.01E-58	0.34195914	0.256	0.061	9.72E-54	17
Ppm1g2	3.25E-58	0.42272428	0.332	0.092	1.05E-53	17
Chchd7	3.44E-58	0.36032163	0.259	0.062	1.11E-53	17
Magohb2	3.57E-58	0.40812746	0.318	0.087	1.15E-53	17
St132	4.69E-58	0.31242833	0.65	0.239	1.51E-53	17
Mrps244	5.11E-58	0.39317964	0.494	0.168	1.65E-53	17
Churc11	5.28E-58	0.34593784	0.412	0.128	1.70E-53	17
Eif1b2	6.79E-58	0.44339507	0.4	0.125	2.19E-53	17
Acat11	1.03E-57	0.34936351	0.293	0.076	3.33E-53	17
Dut5	1.34E-57	0.46455318	0.554	0.195	4.34E-53	17
Cetn32	1.41E-57	0.39827544	0.476	0.161	4.54E-53	17
Eif2s11	1.42E-57	0.31768973	0.362	0.104	4.57E-53	17
Atp5c15	1.66E-57	0.46713948	0.686	0.273	5.37E-53	17
Idh3g1	2.03E-57	0.34917049	0.325	0.09	6.57E-53	17
Irf1	2.04E-57	0.38618341	0.286	0.072	6.58E-53	17
Ddrgk11	2.38E-57	0.2984585	0.288	0.074	7.70E-53	17
Mrps164	3.10E-57	0.35902003	0.471	0.158	1.00E-52	17
Ebna1bp21	4.87E-57	0.36777765	0.343	0.098	1.57E-52	17
Hdac2	5.39E-57	0.33462128	0.259	0.062	1.74E-52	17
Isca12	6.43E-57	0.40569142	0.302	0.08	2.07E-52	17
Maz1	9.12E-57	0.27996263	0.384	0.116	2.94E-52	17
Rtf12	1.12E-56	0.33184716	0.476	0.158	3.60E-52	17
Junb5	1.20E-56	0.33724726	0.879	0.429	3.88E-52	17
Smim3	2.14E-56	0.35803191	0.254	0.06	6.92E-52	17
Dnajc193	2.38E-56	0.42480948	0.419	0.135	7.68E-52	17
Hnrnpa04	3.53E-56	0.4189601	0.714	0.286	1.14E-51	17
Rtraf4	5.24E-56	0.31492566	0.586	0.212	1.69E-51	17
Acin12	5.42E-56	0.29940485	0.476	0.16	1.75E-51	17
Btf37	5.85E-56	0.65742456	0.934	0.544	1.89E-51	17
Eprs	6.22E-56	0.26811043	0.334	0.093	2.01E-51	17
Hnrnpd3	7.89E-56	0.42075031	0.533	0.193	2.55E-51	17
Ddx50	1.10E-55	0.30768844	0.297	0.079	3.54E-51	17
Acadl	1.49E-55	0.38016573	0.339	0.098	4.80E-51	17
Nop531	1.55E-55	0.37850934	0.403	0.126	4.99E-51	17
Eef1e1	2.02E-55	0.29990898	0.252	0.06	6.53E-51	17
Atp5g34	2.84E-55	0.39995471	0.68	0.266	9.16E-51	17
Hint15	3.02E-55	0.63342964	0.867	0.402	9.74E-51	17
0610010K14I	3.37E-55	0.40362109	0.348	0.103	1.09E-50	17
Rpa33	5.41E-55	0.40437204	0.398	0.126	1.75E-50	17
Mrpl481	5.51E-55	0.39513057	0.288	0.077	1.78E-50	17
Ybx33	6.14E-55	0.26518177	0.531	0.183	1.98E-50	17
Rpl45	1.23E-54	0.54295721	0.883	0.411	3.98E-50	17
Nap1l4	1.31E-54	0.36192035	0.307	0.085	4.22E-50	17

Timm232	1.63E-54	0.40444017	0.41	0.131	5.27E-50	17
Cct41	2.02E-54	0.2969823	0.407	0.128	6.51E-50	17
Hspd13	2.31E-54	0.25587436	0.6	0.217	7.45E-50	17
Wsb1	2.77E-54	0.33917973	0.364	0.11	8.93E-50	17
Hdgf3	3.94E-54	0.29279259	0.634	0.239	1.27E-49	17
Znrd13	4.34E-54	0.33289489	0.373	0.114	1.40E-49	17
F2r	4.50E-54	0.27211032	0.263	0.063	1.45E-49	17
Snw1	4.79E-54	0.31130553	0.323	0.091	1.55E-49	17
Pnn2	4.83E-54	0.30278085	0.348	0.102	1.56E-49	17
Ciao2a3	5.54E-54	0.32919216	0.432	0.142	1.79E-49	17
Mcts12	6.67E-54	0.39722219	0.35	0.106	2.15E-49	17
Gm162861	9.40E-54	0.40447276	0.309	0.087	3.04E-49	17
Psmd7	1.06E-53	0.376726	0.389	0.122	3.42E-49	17
Snrnp701	1.27E-53	0.37484713	0.506	0.179	4.11E-49	17
Ndufv24	1.41E-53	0.39412447	0.471	0.164	4.56E-49	17
Ndufaf82	2.27E-53	0.29296866	0.373	0.115	7.32E-49	17
Foxn32	2.63E-53	0.27227298	0.33	0.093	8.49E-49	17
Glo11	3.09E-53	0.35682551	0.293	0.079	9.97E-49	17
Micos107	3.30E-53	0.4684936	0.657	0.266	1.07E-48	17
Gm2000	8.12E-53	0.32092507	0.277	0.072	2.62E-48	17
Ndufa101	1.11E-52	0.33253729	0.334	0.098	3.58E-48	17
Anp32b4	1.16E-52	0.44032427	0.705	0.287	3.73E-48	17
Mrps122	1.19E-52	0.29407207	0.33	0.096	3.85E-48	17
Hnrnpm3	1.37E-52	0.50188991	0.689	0.292	4.43E-48	17
Polr2m1	1.64E-52	0.2652612	0.318	0.09	5.30E-48	17
Eif4a32	3.50E-52	0.30871866	0.35	0.106	1.13E-47	17
Mdh13	3.98E-52	0.29984942	0.412	0.135	1.28E-47	17
Dnajb61	4.68E-52	0.47789479	0.593	0.233	1.51E-47	17
Rbbp44	5.05E-52	0.32169043	0.481	0.169	1.63E-47	17
Srrm12	5.34E-52	0.31007009	0.499	0.179	1.72E-47	17
Plekhj1	5.67E-52	0.39451446	0.323	0.095	1.83E-47	17
Krtcap26	6.15E-52	0.37622902	0.645	0.25	1.99E-47	17
2310009A05	6.18E-52	0.42590419	0.368	0.118	2.00E-47	17
Mettl23	7.57E-52	0.34474664	0.314	0.09	2.44E-47	17
Mcm32	8.14E-52	0.38929754	0.265	0.07	2.63E-47	17
Cox8a6	1.24E-51	0.62170263	0.97	0.682	4.01E-47	17
Phb22	1.61E-51	0.343832	0.387	0.123	5.21E-47	17
Psma54	1.82E-51	0.3324923	0.494	0.177	5.87E-47	17
Ak22	1.98E-51	0.42225176	0.394	0.132	6.41E-47	17
Syncrip1	2.15E-51	0.25412707	0.444	0.148	6.95E-47	17
Rpl126	2.99E-51	0.48464719	0.982	0.534	9.66E-47	17
H2-T232	4.02E-51	0.32834753	0.481	0.17	1.30E-46	17
Hnrnpab3	4.58E-51	0.34297213	0.666	0.263	1.48E-46	17

Gng103	4.77E-51	0.37634836	0.391	0.128	1.54E-46	17
Dennd4a2	5.03E-51	0.41719061	0.449	0.158	1.62E-46	17
Siva15	6.69E-51	0.45156319	0.449	0.157	2.16E-46	17
Hnrnpl3	7.83E-51	0.35209056	0.572	0.222	2.53E-46	17
Rbis3	8.19E-51	0.29042131	0.476	0.167	2.64E-46	17
Sfpq3	1.07E-50	0.30564184	0.59	0.227	3.44E-46	17
Mrpl574	1.11E-50	0.39339996	0.497	0.183	3.58E-46	17
Osbpl8	1.53E-50	0.35414887	0.254	0.066	4.95E-46	17
Dnmt12	2.14E-50	0.44095154	0.343	0.107	6.91E-46	17
Ost46	3.30E-50	0.52986525	0.751	0.344	1.07E-45	17
Rrp11	4.59E-50	0.26900238	0.311	0.09	1.48E-45	17
Srsf112	5.79E-50	0.27818204	0.467	0.165	1.87E-45	17
Pop51	6.22E-50	0.30108518	0.256	0.067	2.01E-45	17
Rps174	6.83E-50	0.5714433	0.961	0.559	2.20E-45	17
Ssr21	6.93E-50	0.35088108	0.41	0.135	2.24E-45	17
Wac	7.42E-50	0.29778028	0.371	0.118	2.40E-45	17
U2af21	7.45E-50	0.28895565	0.412	0.138	2.41E-45	17
Cnpy21	1.02E-49	0.29417432	0.307	0.088	3.29E-45	17
Cacybp1	1.19E-49	0.27951316	0.403	0.132	3.84E-45	17
Gpx44	1.26E-49	0.51893173	0.819	0.393	4.06E-45	17
Psma43	1.28E-49	0.25344678	0.485	0.172	4.12E-45	17
Ndufa55	1.33E-49	0.33759253	0.54	0.202	4.30E-45	17
Klhl9	1.47E-49	0.37634099	0.268	0.072	4.76E-45	17
Sec11a3	1.56E-49	0.32028012	0.396	0.131	5.04E-45	17
Ptbp11	1.58E-49	0.30914536	0.371	0.118	5.11E-45	17
Psmb63	2.24E-49	0.32815051	0.584	0.228	7.25E-45	17
Uqcc22	2.90E-49	0.2720545	0.577	0.215	9.38E-45	17
Lsm64	3.02E-49	0.30165599	0.577	0.22	9.76E-45	17
Jarid21	3.47E-49	0.33623485	0.263	0.071	1.12E-44	17
Btk	3.83E-49	0.29429129	0.256	0.068	1.24E-44	17
Txn21	3.92E-49	0.41419766	0.355	0.113	1.27E-44	17
Itga44	4.89E-49	0.38201823	0.323	0.097	1.58E-44	17
Mrpl422	4.90E-49	0.30853452	0.334	0.102	1.58E-44	17
mt-Nd4l7	5.37E-49	0.4108627	0.883	0.444	1.73E-44	17
Taf105	6.43E-49	0.43500502	0.572	0.227	2.08E-44	17
Odc11	7.66E-49	0.38598143	0.382	0.125	2.47E-44	17
Per1	8.07E-49	0.31391282	0.268	0.072	2.61E-44	17
Ptpre2	1.01E-48	0.42483269	0.35	0.113	3.27E-44	17
Pcbp14	1.07E-48	0.30033953	0.616	0.245	3.46E-44	17
Tex10	1.44E-48	0.32695068	0.252	0.066	4.64E-44	17
Psmb95	1.72E-48	0.39060079	0.444	0.159	5.56E-44	17
Snrpe7	1.91E-48	0.48745748	0.883	0.423	6.18E-44	17
Timm134	2.70E-48	0.46007777	0.574	0.232	8.73E-44	17

Vamp84	2.70E-48	0.5276981	0.634	0.275	8.73E-44	17
Tcea12	2.94E-48	0.31126598	0.478	0.174	9.48E-44	17
Mcm73	3.45E-48	0.3215243	0.403	0.134	1.11E-43	17
Ssb3	3.49E-48	0.32831385	0.561	0.217	1.13E-43	17
Dpm11	5.49E-48	0.2831128	0.366	0.119	1.77E-43	17
Thrap31	1.07E-47	0.27750345	0.398	0.134	3.46E-43	17
Sub17	1.25E-47	0.58041238	0.876	0.475	4.03E-43	17
Sdf2	1.33E-47	0.29356083	0.265	0.073	4.30E-43	17
Wapl1	1.48E-47	0.32938994	0.357	0.114	4.78E-43	17
Tomm52	1.52E-47	0.28737842	0.407	0.137	4.90E-43	17
Nudt31	1.85E-47	0.41042178	0.256	0.069	5.99E-43	17
Sarnp4	2.34E-47	0.37563326	0.597	0.244	7.57E-43	17
Fli11	3.37E-47	0.47102282	0.352	0.117	1.09E-42	17
Nme25	3.81E-47	0.45584888	0.89	0.405	1.23E-42	17
Fau6	3.90E-47	0.40162763	1	0.942	1.26E-42	17
Vdac33	4.53E-47	0.28072065	0.451	0.161	1.46E-42	17
Psma36	4.96E-47	0.35973849	0.613	0.251	1.60E-42	17
Hsd17b10	5.10E-47	0.347952	0.373	0.125	1.65E-42	17
Mrpl151	5.23E-47	0.25957918	0.311	0.094	1.69E-42	17
Sf3b54	6.27E-47	0.3738496	0.535	0.208	2.02E-42	17
Irf2bp21	7.30E-47	0.2821679	0.481	0.18	2.36E-42	17
Timm17b2	7.50E-47	0.32573537	0.343	0.11	2.42E-42	17
0610012G03	8.53E-47	0.31535951	0.323	0.1	2.75E-42	17
Dctn32	9.12E-47	0.30806553	0.469	0.173	2.95E-42	17
Celf24	1.30E-46	0.36541957	0.478	0.181	4.19E-42	17
Zfp911	1.62E-46	0.25120122	0.471	0.171	5.22E-42	17
Slc25a32	1.75E-46	0.47177615	0.803	0.386	5.66E-42	17
Vezf1	1.97E-46	0.25056675	0.272	0.076	6.35E-42	17
Tacc13	2.05E-46	0.3222376	0.426	0.153	6.62E-42	17
Mrpl171	2.52E-46	0.31359624	0.309	0.094	8.14E-42	17
Rpl85	3.00E-46	0.51692348	0.993	0.731	9.67E-42	17
Ifi2034	4.92E-46	0.25247091	0.38	0.125	1.59E-41	17
Ddt2	5.03E-46	0.25811862	0.268	0.075	1.62E-41	17
Calm34	5.60E-46	0.36358112	0.606	0.252	1.81E-41	17
Chchd14	5.64E-46	0.36611581	0.483	0.183	1.82E-41	17
Hmgb15	5.80E-46	0.3381767	0.947	0.533	1.87E-41	17
Eif1a	5.96E-46	0.25328575	0.332	0.103	1.92E-41	17
Kdm1a	8.59E-46	0.25280064	0.297	0.088	2.77E-41	17
Khdrbs11	8.69E-46	0.31540463	0.412	0.143	2.81E-41	17
Sumo14	9.01E-46	0.38914687	0.54	0.219	2.91E-41	17
Clint11	9.85E-46	0.25879137	0.467	0.174	3.18E-41	17
Polr2j4	1.03E-45	0.31836372	0.471	0.177	3.33E-41	17
Gars	1.09E-45	0.26416576	0.263	0.073	3.50E-41	17

Snrnp402	1.18E-45	0.33454926	0.259	0.072	3.80E-41	17
Brd71	1.54E-45	0.25621227	0.384	0.131	4.96E-41	17
Cox5b5	1.96E-45	0.55120209	0.876	0.469	6.33E-41	17
H2-K16	2.27E-45	0.42130795	0.881	0.454	7.34E-41	17
Zc3h154	3.43E-45	0.27406126	0.535	0.208	1.11E-40	17
Uqcr104	4.27E-45	0.49512165	0.794	0.386	1.38E-40	17
Rdx1	6.02E-45	0.25382533	0.471	0.173	1.94E-40	17
Hprt3	6.42E-45	0.29922954	0.359	0.12	2.07E-40	17
Rpn13	6.69E-45	0.38671784	0.458	0.17	2.16E-40	17
Apopt11	2.05E-44	0.32584243	0.254	0.071	6.62E-40	17
Rpl3110	2.35E-44	0.49887572	0.954	0.539	7.57E-40	17
Ccnt1	2.92E-44	0.34884136	0.307	0.095	9.43E-40	17
Cox162	3.27E-44	0.34201693	0.348	0.116	1.06E-39	17
Anp32a4	5.12E-44	0.40550555	0.641	0.278	1.65E-39	17
Prrc2c3	5.17E-44	0.27152152	0.586	0.239	1.67E-39	17
Akr1b32	5.55E-44	0.28200677	0.295	0.09	1.79E-39	17
Prkar1a1	5.76E-44	0.31113255	0.551	0.224	1.86E-39	17
Ift201	8.60E-44	0.29622904	0.32	0.103	2.78E-39	17
Dctn41	8.65E-44	0.30613092	0.435	0.161	2.79E-39	17
Uqcrh6	1.13E-43	0.46926377	0.929	0.509	3.65E-39	17
Fam104a1	1.76E-43	0.32246994	0.432	0.161	5.68E-39	17
Fcf1	2.11E-43	0.28384495	0.293	0.089	6.80E-39	17
Rbm221	2.62E-43	0.28963937	0.341	0.112	8.46E-39	17
Ufc12	3.51E-43	0.27748468	0.348	0.116	1.13E-38	17
Pdcd54	4.57E-43	0.25429891	0.549	0.219	1.47E-38	17
Mrpl241	5.87E-43	0.30935213	0.334	0.11	1.89E-38	17
Grb21	7.79E-43	0.31237365	0.442	0.168	2.51E-38	17
Pfdn23	8.67E-43	0.25864014	0.432	0.16	2.80E-38	17
Tomm66	8.78E-43	0.45323456	0.831	0.415	2.84E-38	17
Gtf3c6	1.36E-42	0.29826376	0.27	0.08	4.39E-38	17
Ubxn13	1.66E-42	0.26250009	0.57	0.232	5.36E-38	17
Dnajb1	1.94E-42	0.44538826	0.286	0.089	6.25E-38	17
Rpp212	2.02E-42	0.34113669	0.394	0.143	6.51E-38	17
Ahsa11	2.41E-42	0.32335758	0.295	0.092	7.79E-38	17
Eef1a15	3.08E-42	0.45426777	0.998	0.723	9.94E-38	17
Sap185	3.41E-42	0.32784571	0.549	0.229	1.10E-37	17
Elf21	3.64E-42	0.2845875	0.295	0.092	1.17E-37	17
Atf42	3.76E-42	0.3437559	0.551	0.23	1.22E-37	17
Agpat51	4.35E-42	0.28604727	0.256	0.074	1.40E-37	17
Hmgcr	6.85E-42	0.27804012	0.304	0.097	2.21E-37	17
Srsf34	7.83E-42	0.42768335	0.792	0.391	2.53E-37	17
Bloc1s13	8.81E-42	0.29264703	0.494	0.197	2.85E-37	17
Ifngr13	1.17E-41	0.37866147	0.341	0.115	3.77E-37	17

Psenen3	1.52E-41	0.25672991	0.593	0.252	4.91E-37	17
Abracl5	1.88E-41	0.41847237	0.62	0.276	6.07E-37	17
Zcrb12	3.58E-41	0.26257071	0.471	0.183	1.16E-36	17
Ran4	7.82E-41	0.34089843	0.78	0.351	2.53E-36	17
Ndufb106	1.11E-40	0.2996667	0.577	0.24	3.60E-36	17
Hsp90aa14	1.14E-40	0.25229172	0.789	0.365	3.70E-36	17
Polr2i4	1.26E-40	0.32016184	0.384	0.14	4.05E-36	17
Srrm23	2.08E-40	0.33693242	0.689	0.315	6.73E-36	17
Coq10b	3.09E-40	0.36678264	0.297	0.096	9.99E-36	17
Lsm54	3.67E-40	0.25241207	0.551	0.224	1.19E-35	17
Rplp15	3.80E-40	0.43311944	0.995	0.706	1.23E-35	17
Uqcrc21	4.08E-40	0.28590252	0.286	0.09	1.32E-35	17
Tubb4b3	4.13E-40	0.33322993	0.46	0.181	1.33E-35	17
Hnrnpf4	5.68E-40	0.33166446	0.696	0.316	1.83E-35	17
Eif1ax3	5.80E-40	0.25015545	0.49	0.193	1.87E-35	17
Hnrnpu3	5.98E-40	0.2662734	0.684	0.301	1.93E-35	17
Rps253	6.17E-40	0.43243831	0.975	0.625	1.99E-35	17
Atp5e8	8.62E-40	0.48789064	0.977	0.695	2.78E-35	17
Alkbh5	1.39E-39	0.25625636	0.309	0.102	4.50E-35	17
Uqcc31	1.87E-39	0.28297333	0.284	0.09	6.04E-35	17
Cggbp12	2.13E-39	0.26935336	0.476	0.192	6.87E-35	17
Atxn7l3b	2.52E-39	0.26473534	0.357	0.127	8.14E-35	17
Eif4e	6.29E-39	0.25393396	0.272	0.085	2.03E-34	17
Rbx15	6.34E-39	0.38747446	0.757	0.37	2.05E-34	17
Nup98	6.80E-39	0.25772255	0.268	0.083	2.19E-34	17
Usp19	1.03E-38	0.266451	0.254	0.077	3.33E-34	17
Tipin3	1.58E-38	0.31706181	0.281	0.09	5.10E-34	17
Wbp111	1.64E-38	0.25993836	0.357	0.127	5.28E-34	17
Unc1192	1.85E-38	0.42955995	0.293	0.098	5.96E-34	17
Cenpw3	3.43E-38	0.30739282	0.325	0.112	1.11E-33	17
Atp6v1f7	5.94E-38	0.26217303	0.693	0.32	1.92E-33	17
Pcbd23	7.75E-38	0.28189097	0.316	0.109	2.50E-33	17
Rps124	1.33E-37	0.31389461	0.995	0.686	4.29E-33	17
Morf4l14	1.39E-37	0.31874859	0.76	0.364	4.50E-33	17
Gtf2a22	1.67E-37	0.26639824	0.38	0.141	5.39E-33	17
Cpne22	2.92E-37	0.35133815	0.261	0.083	9.44E-33	17
Rsu1	3.68E-37	0.3950825	0.254	0.08	1.19E-32	17
Gtf2b1	4.01E-37	0.33249827	0.332	0.12	1.29E-32	17
Smarca54	4.11E-37	0.26568372	0.577	0.251	1.33E-32	17
Cox7b6	5.32E-37	0.35260709	0.721	0.34	1.72E-32	17
Fam173a1	5.70E-37	0.32218277	0.288	0.097	1.84E-32	17
Psmb54	6.54E-37	0.27765649	0.526	0.225	2.11E-32	17
Sumo31	7.42E-37	0.25070893	0.284	0.094	2.40E-32	17

Atp5h6	7.79E-37	0.4354861	0.883	0.471	2.52E-32	17
Fos4	7.84E-37	0.53506027	0.696	0.357	2.53E-32	17
Ech11	1.33E-36	0.27839189	0.309	0.107	4.28E-32	17
Nedd85	2.11E-36	0.29350321	0.737	0.351	6.82E-32	17
Coa33	2.18E-36	0.2946978	0.339	0.123	7.05E-32	17
Ndufb35	2.25E-36	0.25655268	0.568	0.245	7.27E-32	17
Tmem2586	4.23E-36	0.34770364	0.771	0.382	1.37E-31	17
Grpel13	4.85E-36	0.25921299	0.346	0.127	1.57E-31	17
Psip11	5.13E-36	0.27172545	0.325	0.114	1.65E-31	17
Rp92	6.00E-36	0.26664291	0.416	0.165	1.94E-31	17
Hnrnpa2b14	1.24E-35	0.30200531	0.822	0.409	3.99E-31	17
Magoh6	1.49E-35	0.33246677	0.577	0.261	4.80E-31	17
Hnrnpk4	1.96E-35	0.28991368	0.78	0.381	6.34E-31	17
Phlda12	2.40E-35	0.4176569	0.318	0.114	7.76E-31	17
Srp95	3.34E-35	0.25542848	0.584	0.259	1.08E-30	17
Atp5j5	6.28E-35	0.3689017	0.803	0.411	2.03E-30	17
Rack15	7.39E-35	0.31468013	0.952	0.53	2.39E-30	17
Ddx55	8.60E-35	0.40389947	0.913	0.544	2.78E-30	17
Zfp622	1.06E-34	0.28918245	0.268	0.088	3.41E-30	17
Ndufv35	1.75E-34	0.30442584	0.554	0.25	5.64E-30	17
Cox5a5	2.95E-34	0.33575153	0.773	0.386	9.53E-30	17
Glrx22	3.19E-34	0.257193	0.275	0.093	1.03E-29	17
Dnaja13	1.17E-33	0.39502323	0.693	0.347	3.78E-29	17
Tle54	1.96E-33	0.29125231	0.54	0.244	6.32E-29	17
Lig13	2.19E-33	0.26054445	0.286	0.099	7.07E-29	17
Diaph12	3.64E-33	0.2589407	0.378	0.148	1.17E-28	17
Nfu12	1.73E-32	0.26182171	0.304	0.112	5.57E-28	17
Nap1l14	1.89E-32	0.25821693	0.65	0.305	6.10E-28	17
Gng121	2.09E-32	0.27816556	0.3	0.109	6.74E-28	17
Rab5if5	4.27E-32	0.31130891	0.57	0.266	1.38E-27	17
Arpc1a	1.01E-31	0.3976328	0.286	0.103	3.26E-27	17
Ethe14	1.10E-31	0.41278819	0.343	0.138	3.56E-27	17
Srsf55	2.19E-31	0.27504571	0.739	0.379	7.08E-27	17
Tma75	2.90E-31	0.25778093	0.771	0.384	9.38E-27	17
Ndufb95	1.49E-30	0.31003564	0.703	0.358	4.82E-26	17
Rer13	3.75E-30	0.26671964	0.355	0.144	1.21E-25	17
Cox7c7	2.13E-29	0.37784026	0.975	0.668	6.87E-25	17
Atp5f15	2.16E-29	0.29854861	0.643	0.318	6.96E-25	17
Pfdn56	7.80E-28	0.29228366	0.819	0.456	2.52E-23	17
H2afj5	8.88E-27	0.30431247	0.78	0.429	2.87E-22	17
Tmem2081	1.75E-26	0.25960112	0.286	0.112	5.65E-22	17
Ndufb1-ps6	6.98E-25	0.31553195	0.954	0.634	2.25E-20	17
Oaz18	2.14E-24	0.31370437	0.954	0.637	6.89E-20	17

Cox6b15	7.26E-24	0.30154536	0.858	0.494	2.34E-19	17
H2afz5	1.54E-23	0.30587364	0.902	0.571	4.98E-19	17
Gng54	4.19E-22	0.3317069	0.968	0.65	1.35E-17	17
Atp5l5	7.84E-20	0.27078027	0.931	0.605	2.53E-15	17
D8Ertd738e4	1.15E-18	0.31158607	0.737	0.421	3.72E-14	17
Xist5	5.46E-18	0.70377575	0.275	0.131	1.76E-13	17
Rgcc4	2.48E-15	0.26703784	0.265	0.128	8.02E-11	17
Cox6a2	0	5.09223067	0.913	0.01	0	18
Bst21	0	4.41869627	0.983	0.142	0	18
Siglech	0	4.01071453	0.843	0	0	18
Irf82	0	3.44034894	0.913	0.064	0	18
Ccr9	0	2.73495019	0.513	0.001	0	18
Cd71	0	2.69017663	0.643	0.008	0	18
Rnase61	0	2.59342758	0.639	0.031	0	18
Runx2	0	2.50882344	0.665	0.033	0	18
Klk1	0	2.37268441	0.339	0.003	0	18
Klrd11	0	2.24250697	0.552	0.012	0	18
Lair1	0	2.11546777	0.522	0.022	0	18
Cd300c	0	1.81302558	0.361	0.001	0	18
Sh3bgr	0	1.73513573	0.296	0	0	18
Upb1	0	1.64891531	0.339	0.003	0	18
Ppfia4	0	1.53212392	0.335	0.011	0	18
Gm21762	0	1.44103849	0.257	0	0	18
Pltp	0	1.34110969	0.287	0.008	0	18
Arhgap27os2	0	1.29675317	0.261	0.007	0	18
Flt3	0	1.20827767	0.265	0.007	0	18
Tcf41	1.78E-300	2.64136689	0.835	0.1	5.75E-296	18
Smim5	3.19E-289	1.85122668	0.396	0.022	1.03E-284	18
Ccl41	2.56E-283	2.4059251	0.635	0.058	8.25E-279	18
Cd200	2.04E-274	1.38429524	0.291	0.012	6.59E-270	18
Mpeg12	4.78E-246	2.18993036	0.7	0.081	1.54E-241	18
Fyn	7.83E-240	1.44166436	0.361	0.021	2.53E-235	18
Tex2	1.19E-221	1.41272494	0.335	0.02	3.84E-217	18
Tmem229b	1.38E-220	1.27142854	0.27	0.013	4.44E-216	18
Ly6d2	2.52E-213	2.64063866	0.857	0.139	8.13E-209	18
Pld41	1.52E-184	1.73569027	0.465	0.047	4.90E-180	18
Ly863	3.68E-180	1.75745314	0.604	0.079	1.19E-175	18
St8sia41	4.32E-178	1.83200445	0.565	0.071	1.39E-173	18
Ctsh2	1.07E-175	2.00049269	0.7	0.114	3.44E-171	18
Bcl11a1	9.05E-171	1.74359073	0.413	0.04	2.92E-166	18
Nucb2	1.83E-148	1.74318029	0.513	0.071	5.91E-144	18
Gpr171	3.68E-145	1.32650996	0.339	0.031	1.19E-140	18
Irf11	1.75E-143	1.67630593	0.517	0.071	5.64E-139	18

Mef2c5	1.96E-142	1.8428697	0.757	0.152	6.32E-138	18
Plac84	2.03E-141	2.13878214	0.922	0.274	6.56E-137	18
Mvb12a	9.08E-137	1.70493687	0.526	0.079	2.93E-132	18
Ramp13	2.58E-129	1.82268524	0.6	0.112	8.34E-125	18
Rilpl22	9.34E-126	1.52506918	0.5	0.076	3.02E-121	18
Gng104	3.74E-120	1.83974523	0.622	0.128	1.21E-115	18
Rnd3	2.62E-116	1.09869358	0.261	0.023	8.46E-112	18
Psap2	8.12E-116	1.88369026	0.922	0.304	2.62E-111	18
Grn2	1.06E-115	1.85585786	0.665	0.148	3.42E-111	18
Cyth41	1.71E-113	1.63120813	0.448	0.068	5.51E-109	18
Bmyc	4.26E-113	1.3499715	0.33	0.038	1.38E-108	18
Tyrobp5	1.72E-112	2.06194674	0.983	0.411	5.54E-108	18
Iglc32	1.48E-108	1.44158709	0.409	0.055	4.79E-104	18
Ptms3	2.97E-106	1.83088763	0.7	0.169	9.58E-102	18
Tubgcp5	6.03E-106	1.11415123	0.261	0.025	1.95E-101	18
Ptprs1	1.03E-103	1.34062003	0.457	0.072	3.31E-99	18
Ctsl4	2.61E-102	1.85376482	0.761	0.207	8.42E-98	18
H2-DMa3	1.58E-100	1.54697797	0.443	0.073	5.10E-96	18
H2-Aa2	1.16E-98	1.5479576	0.513	0.093	3.76E-94	18
Ctss3	6.30E-97	0.92972065	0.622	0.127	2.03E-92	18
Ctsb5	9.03E-97	1.5717845	0.791	0.237	2.92E-92	18
Rell1	1.78E-96	1.45909983	0.37	0.054	5.76E-92	18
Cyb561a3	2.56E-88	0.973278	0.252	0.028	8.26E-84	18
Gltp1	2.70E-85	1.26971533	0.43	0.079	8.73E-81	18
H2-Eb12	6.52E-85	1.21583861	0.461	0.084	2.10E-80	18
Fyb1	4.10E-83	1.23150341	0.413	0.074	1.32E-78	18
Selplg4	5.98E-83	1.43660911	0.626	0.162	1.93E-78	18
Irf2bp22	5.42E-82	1.50899399	0.657	0.181	1.75E-77	18
Tspan132	5.75E-81	1.27402067	0.483	0.098	1.86E-76	18
Atp1b13	2.15E-76	1.12528979	0.435	0.082	6.95E-72	18
Unc93b14	7.17E-75	1.25132017	0.574	0.144	2.31E-70	18
Tagln27	2.37E-72	1.57758165	0.796	0.305	7.65E-68	18
Rps119	3.43E-72	1.24015847	1	0.73	1.11E-67	18
Cd742	2.60E-71	1.49029858	0.622	0.173	8.41E-67	18
Ctsz3	4.97E-71	1.22235568	0.622	0.17	1.60E-66	18
Tnfrsf13b	1.78E-70	1.09948397	0.313	0.051	5.74E-66	18
Sub18	4.62E-70	1.50280941	0.913	0.479	1.49E-65	18
Sell4	3.82E-69	1.41758281	0.63	0.189	1.23E-64	18
Cst34	1.13E-68	1.35510624	0.926	0.473	3.66E-64	18
Lsp14	2.24E-68	1.42106339	0.804	0.299	7.24E-64	18
Rpl3111	2.83E-68	1.38027119	0.957	0.543	9.14E-64	18
Pgls4	5.65E-68	1.34200809	0.843	0.343	1.82E-63	18
Bmp2k	1.23E-67	1.14917722	0.352	0.065	3.98E-63	18

Dnajc72	3.25E-67	0.59195905	0.713	0.209	1.05E-62	18
Tbc1d8	4.53E-67	1.08592182	0.257	0.038	1.46E-62	18
Psmb87	1.26E-66	1.42327007	0.661	0.214	4.08E-62	18
Slc44a2	1.39E-66	1.08578342	0.313	0.053	4.50E-62	18
H2-Ab12	4.42E-66	1.34189751	0.478	0.112	1.43E-61	18
Bloc1s22	8.21E-66	1.23052828	0.487	0.12	2.65E-61	18
Cd681	8.66E-64	0.98818555	0.3	0.05	2.80E-59	18
Gadd45b1	1.40E-63	1.17156058	0.417	0.091	4.51E-59	18
Emp36	1.52E-63	1.46182735	0.778	0.321	4.91E-59	18
Tmed32	1.84E-62	1.15986348	0.426	0.1	5.95E-58	18
H2-T233	2.44E-62	1.34247295	0.578	0.172	7.87E-58	18
Npc25	1.57E-61	1.15827136	0.726	0.248	5.08E-57	18
Sec61b6	2.08E-61	1.31078977	0.904	0.446	6.73E-57	18
Sec61g7	5.86E-61	1.18990346	0.983	0.61	1.89E-56	18
Lgals13	5.62E-60	1.05677549	0.974	0.403	1.81E-55	18
Ifnar21	6.54E-60	1.24802175	0.435	0.107	2.11E-55	18
Fth13	1.33E-57	0.93464866	1	0.917	4.31E-53	18
Rpl109	1.39E-57	1.22159389	0.987	0.603	4.49E-53	18
Trappc51	2.51E-57	1.16794202	0.365	0.08	8.10E-53	18
Spib2	9.83E-57	0.77823674	0.357	0.071	3.17E-52	18
Rpl36al9	2.55E-56	1.19808682	0.943	0.511	8.23E-52	18
Ppia7	5.24E-56	1.06399367	1	0.71	1.69E-51	18
Rexo22	8.99E-53	1.2460558	0.422	0.11	2.90E-48	18
Insig1	1.33E-52	1.1895227	0.348	0.077	4.28E-48	18
Snx5	2.03E-52	1.07927824	0.465	0.125	6.57E-48	18
Cd483	2.10E-52	1.12621202	0.335	0.073	6.77E-48	18
Stx71	3.87E-52	0.99495324	0.37	0.086	1.25E-47	18
Trim30a	1.87E-51	1.15375677	0.313	0.065	6.02E-47	18
Dad17	3.49E-51	1.10018026	0.67	0.242	1.13E-46	18
Napsa3	4.70E-51	1.10798999	0.491	0.144	1.52E-46	18
Selenow7	1.00E-50	1.09831803	0.709	0.263	3.23E-46	18
Gm2a2	1.04E-50	1.01270524	0.417	0.106	3.36E-46	18
Xbp12	2.08E-49	1.2290546	0.422	0.116	6.73E-45	18
mt-Nd4l8	1.69E-48	1.01429824	0.891	0.448	5.45E-44	18
Fau7	1.85E-48	0.71110859	1	0.942	5.97E-44	18
Sema4b	5.39E-47	0.83391642	0.27	0.051	1.74E-42	18
Stk17b5	8.56E-47	1.05988897	0.748	0.319	2.76E-42	18
Rps276	9.83E-47	0.74860143	1	0.912	3.17E-42	18
Ly6e7	2.00E-46	0.96867483	0.874	0.404	6.44E-42	18
Psme18	3.82E-46	1.06762479	0.726	0.307	1.23E-41	18
11-Sep	5.43E-46	0.93667622	0.343	0.083	1.75E-41	18
Ptpn186	8.94E-46	1.03416352	0.709	0.293	2.89E-41	18
Mbnl15	9.02E-46	1.25693805	0.722	0.327	2.91E-41	18

H2afy6	2.76E-45	1.41726335	0.613	0.241	8.93E-41	18
Clec2d1	4.90E-45	1.07647598	0.37	0.094	1.58E-40	18
Rpl98	7.78E-45	0.82997396	0.987	0.774	2.51E-40	18
Clec12a4	8.42E-45	1.41718775	0.439	0.138	2.72E-40	18
Ppdpf	1.84E-44	0.92246793	0.409	0.112	5.94E-40	18
Fam174a2	2.67E-44	1.05227824	0.278	0.059	8.63E-40	18
Ifi27l2a5	4.63E-44	1.25806999	0.57	0.199	1.50E-39	18
Pdcd46	2.90E-43	0.90124045	0.648	0.241	9.35E-39	18
Rpl35a9	3.48E-43	0.7917009	1	0.857	1.12E-38	18
Rpl309	5.27E-43	0.84769041	1	0.818	1.70E-38	18
Dpm36	8.81E-43	1.01500844	0.643	0.254	2.84E-38	18
Ssr46	1.11E-42	1.01345193	0.648	0.25	3.58E-38	18
Oaz19	3.01E-42	0.91092833	0.948	0.639	9.73E-38	18
Zfp364	8.11E-42	1.03636225	0.6	0.228	2.62E-37	18
Sik12	2.00E-41	1.00920931	0.426	0.128	6.45E-37	18
Rps289	2.23E-41	0.79218202	1	0.846	7.20E-37	18
Dap1	2.30E-41	0.90827326	0.496	0.161	7.42E-37	18
Pycard3	2.67E-41	1.10875613	0.443	0.141	8.61E-37	18
Rps208	1.42E-40	0.82263677	1	0.744	4.60E-36	18
Reep54	1.49E-40	1.16872068	0.635	0.269	4.80E-36	18
Fosb3	1.53E-40	1.09675068	0.457	0.145	4.94E-36	18
Blnk3	1.62E-40	0.80461851	0.322	0.076	5.24E-36	18
Scand15	1.71E-40	0.98011906	0.748	0.355	5.52E-36	18
Ier5	3.54E-40	0.81547406	0.335	0.084	1.14E-35	18
Rps299	4.62E-40	0.69064593	1	0.954	1.49E-35	18
Tsc22d11	5.21E-40	0.87481771	0.339	0.084	1.68E-35	18
Selenos3	2.69E-39	1.07216033	0.409	0.126	8.69E-35	18
Laptm54	3.25E-39	1.02969262	0.643	0.273	1.05E-34	18
Ly6c25	3.44E-39	1.52898823	0.57	0.227	1.11E-34	18
Abhd17a1	4.59E-39	0.95667062	0.361	0.1	1.48E-34	18
Rpgrip15	4.95E-39	1.02946634	0.4	0.121	1.60E-34	18
Rpl198	6.51E-39	0.81078712	0.996	0.761	2.10E-34	18
mt-Co17	7.09E-39	0.76486056	0.996	0.883	2.29E-34	18
Uba528	8.41E-39	0.7765372	0.991	0.758	2.71E-34	18
Rps15a8	2.76E-38	0.77614989	1	0.786	8.90E-34	18
Serp14	1.98E-37	1.08577552	0.648	0.291	6.38E-33	18
Krtcap27	5.35E-37	0.9762844	0.622	0.254	1.73E-32	18
Map3k12	1.65E-36	0.7408349	0.335	0.09	5.34E-32	18
Fgfr1op2	1.75E-36	0.97354395	0.422	0.137	5.64E-32	18
Rps6ka12	1.81E-36	0.8798384	0.274	0.067	5.83E-32	18
Psme23	1.82E-36	0.92850894	0.5	0.184	5.86E-32	18
Sec11c4	1.88E-36	0.92962004	0.674	0.311	6.08E-32	18
Mndal5	2.65E-36	0.90014326	0.391	0.118	8.57E-32	18

Il7r4	8.85E-36	0.67937193	0.3	0.073	2.86E-31	18
Tomm76	1.34E-35	0.85011712	0.874	0.482	4.32E-31	18
Cd475	1.58E-35	1.03032768	0.757	0.401	5.10E-31	18
Spcs22	1.61E-35	0.95657137	0.522	0.197	5.20E-31	18
Kctd123	5.54E-35	0.94169949	0.504	0.189	1.79E-30	18
Rgs101	8.31E-35	0.81227774	0.252	0.058	2.68E-30	18
Rps249	2.11E-34	0.65068362	1	0.856	6.82E-30	18
Rpl399	2.29E-34	0.71731085	1	0.846	7.39E-30	18
Bcl7c3	3.76E-34	0.92453048	0.322	0.091	1.22E-29	18
Smim142	4.33E-34	0.97324113	0.513	0.199	1.40E-29	18
Mtdh2	5.81E-34	0.88444084	0.478	0.172	1.88E-29	18
Ptpn61	9.57E-34	0.93607634	0.4	0.133	3.09E-29	18
2410006H16	1.11E-33	0.85768012	0.783	0.399	3.60E-29	18
Serinc36	1.91E-33	0.82235861	0.739	0.359	6.15E-29	18
AI6622707	3.69E-33	0.93039825	0.409	0.139	1.19E-28	18
Slc38a1	7.99E-33	0.78762294	0.257	0.063	2.58E-28	18
Rps138	8.47E-33	0.70381761	0.996	0.796	2.73E-28	18
Herpud13	1.27E-32	0.80770212	0.452	0.156	4.11E-28	18
Arl6ip52	1.77E-32	0.87548339	0.339	0.102	5.70E-28	18
Arhgap17	2.82E-32	0.78680875	0.283	0.074	9.09E-28	18
Manf3	6.62E-32	0.82905942	0.53	0.208	2.14E-27	18
Smad72	1.31E-31	0.82210608	0.3	0.082	4.24E-27	18
Ubb9	1.32E-31	0.70264873	0.97	0.76	4.25E-27	18
Nsa27	1.58E-31	0.86456028	0.857	0.481	5.10E-27	18
Grcc105	1.83E-31	0.86628949	0.604	0.27	5.90E-27	18
Rpl118	1.90E-31	0.72055587	0.991	0.741	6.13E-27	18
H2-Q73	2.08E-31	0.84986534	0.33	0.095	6.70E-27	18
Ech12	2.47E-31	1.07075093	0.339	0.109	7.98E-27	18
Fos5	2.83E-31	0.90951218	0.713	0.36	9.13E-27	18
Smdt17	2.97E-31	0.91434929	0.674	0.339	9.60E-27	18
Rnf187	5.71E-31	0.95952896	0.317	0.094	1.84E-26	18
Atp5e9	9.26E-31	0.70413129	0.97	0.697	2.99E-26	18
Cybb5	1.49E-30	0.85339723	0.574	0.236	4.82E-26	18
Cmtm76	2.12E-30	0.76223362	0.57	0.239	6.86E-26	18
Rap1a1	2.23E-30	0.95491078	0.474	0.188	7.21E-26	18
Psmb96	3.57E-30	0.91331515	0.435	0.162	1.15E-25	18
Rps4x9	4.81E-30	0.71037848	0.983	0.695	1.55E-25	18
Jund6	8.91E-30	0.58973393	0.87	0.513	2.88E-25	18
Dusp52	1.27E-29	0.68798095	0.37	0.118	4.11E-25	18
H131	1.42E-29	0.86241066	0.391	0.137	4.58E-25	18
Rps27a8	1.73E-29	0.59930274	1	0.893	5.60E-25	18
Rac28	2.30E-29	0.85930476	0.765	0.409	7.44E-25	18
Med10	2.99E-29	0.80390899	0.317	0.098	9.66E-25	18

Hsp90b15	5.15E-29	0.7618745	0.743	0.378	1.66E-24	18
Klf25	2.29E-28	0.83039299	0.535	0.226	7.40E-24	18
Tacc14	5.66E-28	0.76587736	0.417	0.155	1.83E-23	18
Zfp36l13	5.93E-28	0.83281273	0.383	0.129	1.91E-23	18
Rpl369	1.39E-27	0.67229858	0.996	0.762	4.49E-23	18
Rpl298	1.96E-27	0.71469451	0.922	0.575	6.32E-23	18
Uqcr117	2.51E-27	0.91560114	0.7	0.359	8.10E-23	18
Fcer1g6	2.60E-27	0.54530343	0.861	0.396	8.39E-23	18
Cox8a7	2.61E-27	0.66476617	0.961	0.685	8.43E-23	18
Gng21	2.76E-27	0.78040094	0.33	0.107	8.92E-23	18
Rpl37a9	4.33E-27	0.58040725	1	0.905	1.40E-22	18
Rpl189	4.88E-27	0.66905578	0.974	0.714	1.57E-22	18
Rpl218	8.03E-27	0.66283623	0.991	0.72	2.59E-22	18
Atp5g28	1.25E-26	0.74690407	0.783	0.415	4.04E-22	18
Ahnak4	1.30E-26	0.40923191	0.674	0.277	4.19E-22	18
Srsf56	3.05E-26	0.74229948	0.717	0.383	9.86E-22	18
Cytip6	4.28E-26	0.67135467	0.461	0.181	1.38E-21	18
Syngr21	1.12E-25	0.75683211	0.278	0.086	3.62E-21	18
Serf24	1.14E-25	0.65032867	0.961	0.728	3.68E-21	18
Rabgap1l2	1.22E-25	0.68726343	0.291	0.089	3.93E-21	18
H2afj6	1.39E-25	0.84351259	0.743	0.432	4.49E-21	18
Rtraf5	2.30E-25	0.72817425	0.509	0.216	7.42E-21	18
Rpl18a9	4.03E-25	0.5728085	1	0.807	1.30E-20	18
Nptn	4.75E-25	0.85389141	0.309	0.102	1.53E-20	18
Cope2	6.28E-25	0.84774083	0.426	0.174	2.03E-20	18
Eif3f8	7.02E-25	0.74842943	0.6	0.29	2.27E-20	18
Ptprcap6	8.39E-25	0.42819898	0.47	0.176	2.71E-20	18
Rpl279	1.01E-24	0.58770393	0.996	0.725	3.26E-20	18
Mrpl528	1.16E-24	0.686634	0.643	0.302	3.75E-20	18
Tram11	2.12E-24	0.74827889	0.417	0.168	6.85E-20	18
Rpl378	2.25E-24	0.54045447	1	0.915	7.26E-20	18
Rps99	2.34E-24	0.56334108	0.991	0.876	7.56E-20	18
Btg14	4.56E-24	0.32431531	0.957	0.621	1.47E-19	18
Tmem1603	5.83E-24	0.73366431	0.374	0.143	1.88E-19	18
Pfdn57	6.81E-24	0.74130499	0.774	0.459	2.20E-19	18
Tmem2587	9.13E-24	0.75010904	0.709	0.386	2.95E-19	18
Atp6v0e3	3.09E-23	0.69127403	0.604	0.303	9.97E-19	18
Sms1	3.61E-23	0.76774344	0.352	0.128	1.17E-18	18
H3f3b4	4.36E-23	0.41417831	0.987	0.825	1.41E-18	18
Celf25	6.94E-23	0.64223487	0.435	0.184	2.24E-18	18
Rps59	2.66E-22	0.58115414	0.974	0.683	8.59E-18	18
Tapbp1	2.88E-22	0.70054196	0.339	0.124	9.31E-18	18
Jtb1	3.11E-22	0.6777202	0.257	0.081	1.00E-17	18

Spty2d1	4.11E-22	0.64226366	0.261	0.083	1.33E-17	18
Fkbp23	4.47E-22	0.78228045	0.387	0.159	1.44E-17	18
Gm1007611	4.69E-22	0.52613964	1	0.81	1.51E-17	18
Atp6v1d	5.71E-22	0.74029999	0.283	0.096	1.84E-17	18
Pafah1b33	6.42E-22	0.38963736	0.452	0.178	2.07E-17	18
H2-K17	6.48E-22	0.69541533	0.765	0.459	2.09E-17	18
Itm2b5	7.04E-22	0.71983282	0.83	0.537	2.27E-17	18
Rpl349	1.68E-21	0.53255997	0.996	0.826	5.43E-17	18
Ifi303	2.26E-21	0.48135242	0.365	0.133	7.30E-17	18
Rps189	2.67E-21	0.5500576	0.978	0.65	8.61E-17	18
Crip17	2.89E-21	0.57935834	0.761	0.4	9.34E-17	18
Sh3bgrl39	3.75E-21	0.5879932	0.909	0.57	1.21E-16	18
Ucp27	4.13E-21	0.51680155	0.67	0.35	1.33E-16	18
Jmjd1c	1.17E-20	0.60375787	0.283	0.096	3.79E-16	18
Rps39	1.28E-20	0.55173547	0.974	0.708	4.13E-16	18
Zfp36l24	1.61E-20	0.7004412	0.535	0.261	5.19E-16	18
Atp5l6	5.80E-20	0.60756558	0.883	0.609	1.87E-15	18
Atp6v1f8	8.68E-20	0.5382091	0.609	0.324	2.80E-15	18
Dctpp13	8.77E-20	0.70433498	0.304	0.114	2.83E-15	18
Erp295	1.07E-19	0.51020439	0.478	0.211	3.44E-15	18
Chmp4b2	1.39E-19	0.62239022	0.526	0.261	4.48E-15	18
Tomm205	1.51E-19	0.63070995	0.535	0.259	4.88E-15	18
Elf14	2.35E-19	0.57376473	0.352	0.139	7.58E-15	18
Bloc1s14	2.52E-19	0.68839905	0.43	0.2	8.12E-15	18
Rpl320	3.19E-19	0.53826209	0.913	0.559	1.03E-14	18
Cdkn2d3	3.93E-19	0.85292869	0.361	0.155	1.27E-14	18
Snx18	5.78E-19	0.61689774	0.257	0.088	1.87E-14	18
Atp5c16	8.13E-19	0.70332437	0.535	0.278	2.62E-14	18
Tmem591	1.18E-18	0.73420869	0.361	0.154	3.79E-14	18
mt-Atp66	1.18E-18	0.46107886	0.965	0.746	3.82E-14	18
Psmg43	1.37E-18	0.7388044	0.3	0.116	4.42E-14	18
Gpx15	1.53E-18	0.37101878	0.83	0.517	4.93E-14	18
Timm10b5	1.68E-18	0.79395173	0.509	0.261	5.43E-14	18
Rbbp61	2.06E-18	0.62070768	0.291	0.109	6.66E-14	18
mt-Atp87	3.09E-18	0.61044005	0.539	0.277	9.97E-14	18
Lamtor45	3.79E-18	0.71632142	0.539	0.28	1.22E-13	18
Dbnl	5.31E-18	0.65466825	0.283	0.107	1.71E-13	18
Tmem1341	5.83E-18	0.5926714	0.383	0.168	1.88E-13	18
Rpl248	5.89E-18	0.51561183	1	0.766	1.90E-13	18
Rnasek3	5.90E-18	0.62930991	0.543	0.29	1.90E-13	18
Pkig4	8.94E-18	0.49803885	0.348	0.14	2.89E-13	18
Hnrnpl4	9.06E-18	0.61914378	0.465	0.226	2.93E-13	18
Hnrnpa16	1.41E-17	0.63805766	0.543	0.276	4.54E-13	18

Uqcrb6	1.59E-17	0.59060767	0.6	0.323	5.14E-13	18
Rps269	1.69E-17	0.48390869	1	0.788	5.46E-13	18
Brk12	1.76E-17	0.60945402	0.417	0.195	5.68E-13	18
Pim16	2.37E-17	0.49742987	0.626	0.34	7.64E-13	18
Klf41	2.47E-17	0.6396181	0.265	0.096	7.97E-13	18
Rps220	2.50E-17	0.40608736	0.97	0.631	8.07E-13	18
Gm499802	2.79E-17	0.43741211	0.27	0.096	8.99E-13	18
Rpl389	2.82E-17	0.45917644	1	0.843	9.09E-13	18
Micos108	3.62E-17	0.57116669	0.53	0.271	1.17E-12	18
Cenpx4	3.65E-17	0.61253542	0.322	0.135	1.18E-12	18
Cnp2	5.36E-17	0.25867919	0.257	0.088	1.73E-12	18
Micos134	6.17E-17	0.70172601	0.422	0.204	1.99E-12	18
mt-Nd54	6.40E-17	0.42337813	0.557	0.284	2.06E-12	18
Rpl22l18	1.03E-16	0.4661047	0.848	0.522	3.33E-12	18
Atp6v0c4	1.06E-16	0.57443986	0.67	0.397	3.43E-12	18
Churc12	1.21E-16	0.67698929	0.313	0.132	3.91E-12	18
Gas55	2.26E-16	0.44183166	0.761	0.43	7.31E-12	18
Tsc22d35	2.28E-16	0.56347585	0.391	0.178	7.37E-12	18
Rps1610	2.51E-16	0.45715053	0.991	0.778	8.09E-12	18
Taf106	2.82E-16	0.57688532	0.461	0.231	9.10E-12	18
Rasgrp24	3.35E-16	0.59846771	0.339	0.149	1.08E-11	18
Pomp6	3.47E-16	0.57913508	0.604	0.346	1.12E-11	18
Ndufb107	4.10E-16	0.53566091	0.478	0.244	1.32E-11	18
Psmb102	4.65E-16	0.55817019	0.304	0.124	1.50E-11	18
Zmynd8	4.65E-16	0.40473076	0.257	0.093	1.50E-11	18
Plek3	4.86E-16	0.38040679	0.357	0.155	1.57E-11	18
Slc3a2	4.93E-16	0.60584897	0.287	0.115	1.59E-11	18
Sptssa4	5.39E-16	0.68620888	0.278	0.114	1.74E-11	18
Ubc2	6.08E-16	0.68530685	0.661	0.418	1.96E-11	18
Psmb25	6.40E-16	0.61129335	0.452	0.228	2.07E-11	18
Ndufs54	6.59E-16	0.48978013	0.535	0.286	2.13E-11	18
Cirbp2	6.59E-16	0.63029081	0.261	0.1	2.13E-11	18
Jpt13	7.72E-16	0.58897609	0.465	0.233	2.49E-11	18
Rpl10a9	8.12E-16	0.44184727	0.961	0.606	2.62E-11	18
Ikzf11	8.40E-16	0.5849071	0.33	0.144	2.71E-11	18
R3hdm43	8.49E-16	0.74865518	0.387	0.187	2.74E-11	18
Rps79	9.00E-16	0.4274962	0.97	0.731	2.91E-11	18
Clta7	1.11E-15	0.52755571	0.574	0.321	3.60E-11	18
Atp6v0b5	1.27E-15	0.62855665	0.513	0.284	4.10E-11	18
Rplp07	1.36E-15	0.45179783	0.978	0.739	4.39E-11	18
Erh7	1.41E-15	0.46030876	0.474	0.229	4.55E-11	18
Chic21	1.51E-15	0.71812169	0.313	0.137	4.87E-11	18
Bzw12	1.68E-15	0.57384766	0.443	0.221	5.44E-11	18

Rpl329	2.01E-15	0.44281933	0.996	0.731	6.50E-11	18
Gna13	2.11E-15	0.58986239	0.257	0.099	6.83E-11	18
Bax3	2.35E-15	0.50357114	0.343	0.151	7.58E-11	18
Sumo26	2.38E-15	0.5311234	0.622	0.35	7.68E-11	18
Mrps165	2.90E-15	0.60037903	0.352	0.162	9.37E-11	18
Atp5mpl7	2.97E-15	0.56729688	0.643	0.375	9.60E-11	18
Rpl359	2.97E-15	0.45936691	1	0.775	9.60E-11	18
Abracl6	4.37E-15	0.60316757	0.504	0.28	1.41E-10	18
Tle55	4.43E-15	0.56442717	0.465	0.247	1.43E-10	18
Mef2a1	5.72E-15	0.55633593	0.257	0.099	1.85E-10	18
Rpl137	7.19E-15	0.42977657	1	0.793	2.32E-10	18
Coro1a7	7.77E-15	0.49779328	0.722	0.437	2.51E-10	18
Tpd523	8.54E-15	0.39094424	0.396	0.181	2.76E-10	18
Ppp1r11	1.07E-14	0.5849238	0.335	0.153	3.45E-10	18
Atp1a11	1.20E-14	0.52776094	0.291	0.12	3.88E-10	18
Prkcd1	1.21E-14	0.63168076	0.309	0.136	3.91E-10	18
Pnrc16	1.36E-14	0.39178012	0.574	0.317	4.39E-10	18
2-Jun	1.65E-14	0.67648042	0.378	0.179	5.33E-10	18
Rbx16	1.86E-14	0.5204721	0.635	0.375	6.00E-10	18
Klf132	1.87E-14	0.53281144	0.37	0.176	6.03E-10	18
Sdhb4	2.14E-14	0.63223115	0.365	0.176	6.92E-10	18
Sdc43	2.31E-14	0.4314046	0.352	0.15	7.45E-10	18
Park75	3.26E-14	0.47342258	0.457	0.232	1.05E-09	18
Atp5h7	3.31E-14	0.56706564	0.73	0.476	1.07E-09	18
Rpl27a8	3.51E-14	0.41084431	0.996	0.8	1.13E-09	18
Tmco14	4.50E-14	0.66729491	0.339	0.159	1.45E-09	18
Ost47	4.62E-14	0.54230502	0.591	0.35	1.49E-09	18
Sf3b65	4.69E-14	0.56308886	0.487	0.265	1.52E-09	18
mt-Co35	5.15E-14	0.29403834	0.978	0.798	1.66E-09	18
Xist6	5.50E-14	0.56025636	0.309	0.132	1.78E-09	18
Morf4l15	5.53E-14	0.51814919	0.613	0.369	1.78E-09	18
Naca8	5.65E-14	0.475964	0.852	0.574	1.82E-09	18
Trmt1127	6.33E-14	0.45691386	0.47	0.243	2.04E-09	18
Ptprc6	6.70E-14	0.42582804	0.504	0.273	2.16E-09	18
Tmed103	7.83E-14	0.60072238	0.426	0.224	2.53E-09	18
Ubl56	8.67E-14	0.5630244	0.77	0.521	2.80E-09	18
Hspe14	9.12E-14	0.49167601	0.539	0.28	2.94E-09	18
Tmsb106	9.46E-14	0.26436968	0.987	0.747	3.05E-09	18
Brd21	1.02E-13	0.4510738	0.317	0.14	3.30E-09	18
Actb5	1.21E-13	0.3605265	1	0.949	3.90E-09	18
Fbl2	1.24E-13	0.52498533	0.27	0.112	3.99E-09	18
Nme26	1.52E-13	0.36718165	0.748	0.41	4.90E-09	18
Tmed92	1.73E-13	0.4824832	0.343	0.161	5.60E-09	18

Snrpd25	1.86E-13	0.40646058	0.557	0.301	6.01E-09	18
Nt5c2	2.20E-13	0.55370009	0.265	0.112	7.11E-09	18
Dnajb62	2.26E-13	0.44866136	0.448	0.238	7.29E-09	18
Sap186	2.27E-13	0.54225081	0.435	0.232	7.34E-09	18
Atp5g16	2.28E-13	0.56469826	0.517	0.286	7.35E-09	18
Cox5b6	2.39E-13	0.54036897	0.717	0.474	7.72E-09	18
Ndufa19	3.03E-13	0.52093111	0.587	0.358	9.78E-09	18
Eif3k6	3.88E-13	0.45276118	0.548	0.311	1.25E-08	18
Uqcrq4	4.96E-13	0.50913233	0.657	0.411	1.60E-08	18
Ndufaf83	5.45E-13	0.42801317	0.274	0.118	1.76E-08	18
Ddost2	5.47E-13	0.47749527	0.274	0.118	1.77E-08	18
Polr2l4	6.52E-13	0.55410558	0.291	0.132	2.11E-08	18
Skp1a4	6.54E-13	0.45000971	0.43	0.224	2.11E-08	18
Psenen4	7.17E-13	0.47487288	0.47	0.256	2.32E-08	18
Selenok4	8.02E-13	0.43977258	0.639	0.394	2.59E-08	18
Srp143	9.13E-13	0.37309784	0.509	0.287	2.95E-08	18
Ndufb36	1.07E-12	0.4723407	0.457	0.249	3.44E-08	18
Cox145	1.14E-12	0.63372026	0.348	0.176	3.67E-08	18
Atp5g35	1.16E-12	0.44516831	0.496	0.271	3.74E-08	18
Chrac11	1.25E-12	0.56037363	0.261	0.114	4.05E-08	18
Cd164	1.35E-12	0.53127807	0.287	0.13	4.35E-08	18
Yeats41	1.49E-12	0.39582191	0.3	0.137	4.80E-08	18
Ptpn11	1.49E-12	0.52763859	0.27	0.118	4.82E-08	18
Ppib4	1.80E-12	0.46362346	0.574	0.336	5.81E-08	18
Ufc13	1.81E-12	0.48481553	0.27	0.119	5.84E-08	18
Atp6v1g14	1.83E-12	0.48335791	0.565	0.344	5.90E-08	18
Vamp85	2.32E-12	0.48703114	0.483	0.28	7.50E-08	18
Ostc3	3.42E-12	0.45298865	0.365	0.181	1.10E-07	18
Srsf94	3.63E-12	0.51381378	0.37	0.191	1.17E-07	18
Gnas3	4.13E-12	0.30288982	0.7	0.444	1.33E-07	18
Ccdc122	4.25E-12	0.51440079	0.404	0.218	1.37E-07	18
Emc63	5.01E-12	0.45219026	0.287	0.132	1.62E-07	18
Lyn5	5.23E-12	0.39682396	0.413	0.218	1.69E-07	18
Pdia41	5.44E-12	0.47137864	0.265	0.115	1.76E-07	18
Rpl158	8.72E-12	0.35402647	0.957	0.662	2.82E-07	18
Tmed21	8.76E-12	0.40207077	0.4	0.212	2.83E-07	18
Hmgn15	9.00E-12	0.3962	0.478	0.249	2.91E-07	18
Smc61	9.61E-12	0.39473822	0.304	0.142	3.10E-07	18
Lamtor24	9.92E-12	0.45577218	0.474	0.269	3.20E-07	18
Rps3a18	1.05E-11	0.34346507	0.991	0.775	3.38E-07	18
Ndufa47	1.19E-11	0.37234093	0.757	0.465	3.84E-07	18
Rpl2210	1.22E-11	0.33251752	0.978	0.667	3.93E-07	18
Timm135	1.22E-11	0.44491533	0.439	0.236	3.94E-07	18

Ifi2035	1.25E-11	0.58467731	0.283	0.128	4.04E-07	18
Gstp16	1.63E-11	0.51448845	0.304	0.147	5.26E-07	18
Ndufb25	1.70E-11	0.42056414	0.422	0.226	5.49E-07	18
Srp96	1.94E-11	0.4869625	0.461	0.263	6.27E-07	18
Chchd26	2.07E-11	0.38486014	0.822	0.582	6.67E-07	18
Calm23	2.23E-11	0.3847318	0.748	0.487	7.21E-07	18
Rpl239	2.51E-11	0.33941752	0.987	0.825	8.10E-07	18
Atp5d7	2.79E-11	0.38852035	0.483	0.273	9.02E-07	18
Edf16	2.88E-11	0.50824046	0.522	0.313	9.29E-07	18
Ywhae4	3.28E-11	0.47190657	0.587	0.352	1.06E-06	18
Bri34	4.53E-11	0.4114015	0.465	0.264	1.46E-06	18
Sp1001	4.60E-11	0.65201898	0.252	0.117	1.49E-06	18
Mrpl575	5.03E-11	0.43036021	0.357	0.187	1.62E-06	18
Cox5a6	5.39E-11	0.43264616	0.626	0.391	1.74E-06	18
Rpl4110	5.96E-11	0.31521992	1	0.902	1.92E-06	18
Lsm121	6.28E-11	0.40829311	0.261	0.119	2.03E-06	18
Srsf35	7.07E-11	0.35934771	0.639	0.396	2.28E-06	18
Ndufa134	7.15E-11	0.42417222	0.661	0.429	2.31E-06	18
Cox6b16	8.91E-11	0.39136558	0.752	0.498	2.88E-06	18
1110004F10l	8.94E-11	0.41097838	0.357	0.185	2.89E-06	18
Rpsa7	8.97E-11	0.30619792	0.974	0.703	2.90E-06	18
Eif3h8	1.19E-10	0.28543888	0.491	0.277	3.83E-06	18
Snhg17	1.21E-10	0.35691747	0.4	0.21	3.91E-06	18
Uqcr105	1.22E-10	0.43331522	0.613	0.392	3.95E-06	18
Ndufa73	1.65E-10	0.45670776	0.648	0.431	5.33E-06	18
Abhd2	1.75E-10	0.40936301	0.283	0.136	5.64E-06	18
Uqcrh7	1.88E-10	0.39440442	0.748	0.514	6.07E-06	18
Tmem50a1	1.90E-10	0.40212872	0.357	0.189	6.15E-06	18
Zeb22	1.92E-10	0.39689133	0.296	0.142	6.20E-06	18
Dynll14	2.21E-10	0.36966801	0.704	0.453	7.14E-06	18
Med212	2.58E-10	0.4570402	0.27	0.13	8.32E-06	18
Atxn7l3b1	2.61E-10	0.49589735	0.27	0.13	8.42E-06	18
Skap23	2.80E-10	0.5037586	0.3	0.153	9.05E-06	18
Hmgb16	2.81E-10	0.25108745	0.813	0.538	9.06E-06	18
Rpl267	2.91E-10	0.29292636	0.978	0.703	9.41E-06	18
Gatad11	3.11E-10	0.43678385	0.265	0.126	1.00E-05	18
Psma37	3.21E-10	0.35051329	0.452	0.256	1.04E-05	18
H2-D16	3.65E-10	0.3594253	0.87	0.661	1.18E-05	18
Rps109	3.77E-10	0.29510311	0.991	0.804	1.22E-05	18
Nmt14	4.45E-10	0.42335579	0.378	0.208	1.44E-05	18
Psmb16	4.49E-10	0.30918487	0.513	0.304	1.45E-05	18
Rpl288	4.70E-10	0.31293644	0.978	0.76	1.52E-05	18
Etfb4	6.35E-10	0.40765873	0.317	0.165	2.05E-05	18

Tmem2566	6.44E-10	0.36491183	0.47	0.276	2.08E-05	18
Mpc12	6.48E-10	0.54514171	0.339	0.183	2.09E-05	18
Cd536	6.54E-10	0.35401393	0.443	0.26	2.11E-05	18
Sp33	6.62E-10	0.51653074	0.274	0.136	2.14E-05	18
Tra2b7	7.27E-10	0.35634882	0.448	0.26	2.35E-05	18
Arpc36	7.42E-10	0.34737626	0.77	0.518	2.39E-05	18
Rps27l7	7.47E-10	0.30582176	0.587	0.348	2.41E-05	18
Rpl7a8	7.57E-10	0.32766135	0.843	0.56	2.44E-05	18
Ddx56	8.58E-10	0.39389501	0.77	0.549	2.77E-05	18
Snu135	1.01E-09	0.30502604	0.474	0.273	3.26E-05	18
Mdh14	1.04E-09	0.41270841	0.278	0.139	3.37E-05	18
Rps198	1.10E-09	0.27652492	0.97	0.732	3.55E-05	18
Lsm65	1.13E-09	0.37720108	0.4	0.225	3.65E-05	18
Arhgap301	1.16E-09	0.48987163	0.261	0.128	3.75E-05	18
Cib11	1.23E-09	0.37056177	0.274	0.136	3.97E-05	18
Tmem14c4	1.28E-09	0.30077948	0.413	0.235	4.12E-05	18
Pan32	1.46E-09	0.31016605	0.27	0.131	4.72E-05	18
Gm118086	1.70E-09	0.43595288	0.326	0.173	5.47E-05	18
Ubl31	1.88E-09	0.28751086	0.252	0.118	6.06E-05	18
Rab21	1.90E-09	0.39782557	0.3	0.157	6.12E-05	18
Ssr11	1.95E-09	0.40466692	0.304	0.158	6.29E-05	18
Sf3b55	2.05E-09	0.38908063	0.378	0.213	6.62E-05	18
Cuta5	2.34E-09	0.45436214	0.322	0.174	7.54E-05	18
Dctn33	2.60E-09	0.42085694	0.326	0.177	8.39E-05	18
Cwc153	2.62E-09	0.32855187	0.343	0.184	8.46E-05	18
Dnajc194	2.67E-09	0.63132799	0.27	0.139	8.62E-05	18
Ubxn4	2.99E-09	0.37804193	0.252	0.121	9.65E-05	18
Mtch1	3.23E-09	0.3353047	0.265	0.129	0.00010443	18
Mrpl234	3.48E-09	0.33538309	0.291	0.148	0.00011236	18
Snrpd36	3.58E-09	0.4208663	0.448	0.268	0.00011566	18
Supt165	4.25E-09	0.32551881	0.322	0.169	0.00013732	18
Tmem2341	4.33E-09	0.37638725	0.291	0.15	0.0001398	18
Ndufb45	4.50E-09	0.3631888	0.413	0.241	0.00014515	18
Ndufa35	4.57E-09	0.42836787	0.6	0.41	0.00014752	18
Capzb3	4.57E-09	0.31125762	0.487	0.298	0.00014764	18
Arl6ip16	4.77E-09	0.49498356	0.365	0.211	0.00015396	18
Sarnp5	5.21E-09	0.30449103	0.426	0.249	0.00016814	18
Anp32a5	6.17E-09	0.40762884	0.461	0.283	0.00019915	18
Cbl1	6.42E-09	0.44027523	0.252	0.126	0.00020713	18
Ndufa56	6.74E-09	0.32197144	0.37	0.207	0.00021751	18
Tnfaip8	7.18E-09	0.37147169	0.257	0.129	0.00023193	18
Rgcc5	7.57E-09	0.66288072	0.252	0.13	0.00024448	18
Eif5a5	8.16E-09	0.39781263	0.622	0.409	0.00026329	18

Polr1d7	8.89E-09	0.29329935	0.496	0.301	0.00028706	18
Snrpb7	9.12E-09	0.27457771	0.474	0.279	0.00029431	18
Tbca3	9.80E-09	0.33665694	0.474	0.288	0.00031643	18
Rbm36	9.99E-09	0.33500106	0.652	0.429	0.00032259	18
Arf53	1.11E-08	0.35931955	0.543	0.361	0.00035919	18
Atp5j25	1.20E-08	0.30920742	0.778	0.542	0.00038666	18
Ndufb56	1.20E-08	0.35575295	0.396	0.231	0.00038869	18
Ap2b1	1.23E-08	0.33701559	0.283	0.146	0.00039782	18
Lsm45	1.24E-08	0.31666133	0.396	0.227	0.00039976	18
Grpel14	1.68E-08	0.44085931	0.252	0.129	0.00054087	18
Ndufb115	1.98E-08	0.39656574	0.496	0.325	0.00063977	18
Nedd86	2.28E-08	0.3076753	0.557	0.356	0.00073655	18
Mrps145	2.37E-08	0.35775227	0.339	0.193	0.00076489	18
Dctn42	2.50E-08	0.42061875	0.3	0.165	0.00080804	18
Mrfap14	2.64E-08	0.29233136	0.348	0.193	0.00085285	18
Prpf4b5	2.84E-08	0.2648565	0.426	0.248	0.00091737	18
Fis14	2.97E-08	0.37577811	0.557	0.374	0.00095791	18
Ndufs84	3.16E-08	0.31398677	0.317	0.175	0.00102158	18
Mrps335	3.40E-08	0.40525206	0.343	0.196	0.00109627	18
Ssr22	4.03E-08	0.33645544	0.27	0.139	0.00130213	18
Lsm74	4.33E-08	0.38113025	0.287	0.153	0.00139745	18
Mrps245	4.54E-08	0.41632304	0.309	0.173	0.00146694	18
Notch24	4.54E-08	0.30165733	0.365	0.211	0.00146708	18
Ndufc15	4.96E-08	0.34683632	0.509	0.326	0.00160003	18
Syf23	5.79E-08	0.40104822	0.348	0.202	0.00187051	18
BC031181	5.92E-08	0.32686935	0.296	0.162	0.00191261	18
Tmbim64	6.11E-08	0.32998688	0.47	0.299	0.00197216	18
Akr1a13	6.25E-08	0.34213989	0.322	0.178	0.00201716	18
Ndufb1-ps7	6.47E-08	0.26932167	0.839	0.638	0.00208847	18
Ywhaq3	6.55E-08	0.29107087	0.361	0.206	0.00211318	18
Uqcrfs14	6.78E-08	0.27356471	0.313	0.173	0.00218818	18
Snhg33	6.98E-08	0.3657598	0.261	0.135	0.00225404	18
Abcf13	7.36E-08	0.28378188	0.3	0.16	0.00237476	18
Tspo4	8.17E-08	0.46938596	0.648	0.459	0.00263773	18
Cox7b7	8.44E-08	0.32033781	0.535	0.345	0.00272445	18
Capza23	8.69E-08	0.28679388	0.443	0.278	0.00280542	18
Cox6c6	9.43E-08	0.27109244	0.861	0.639	0.00304434	18
Srsf75	1.13E-07	0.27561841	0.378	0.224	0.00365018	18
Ndufv36	1.23E-07	0.52285515	0.391	0.254	0.00397211	18
Atp5f16	1.28E-07	0.42586216	0.474	0.322	0.00414626	18
Ndufa115	1.41E-07	0.33553288	0.422	0.262	0.00456811	18
Macf1	1.53E-07	0.28683323	0.252	0.131	0.00493841	18
Atp5j6	1.85E-07	0.29121534	0.622	0.416	0.00595881	18

Ndufa26	2.00E-07	0.2544019	0.648	0.437	0.00644244	18
Wasf22	2.00E-07	0.25993651	0.283	0.154	0.00645661	18
Naa385	2.06E-07	0.38662522	0.291	0.162	0.00663695	18
Rpl79	2.61E-07	0.25601005	0.87	0.634	0.00841029	18
Smim115	3.11E-07	0.3882546	0.257	0.141	0.01004741	18
Rnf74	3.12E-07	0.29647913	0.283	0.157	0.01006087	18
Skil2	3.15E-07	0.2606097	0.33	0.189	0.01017698	18
Mrpl305	3.70E-07	0.28432664	0.313	0.179	0.01196009	18
Mtpn	3.75E-07	0.25369534	0.326	0.187	0.01211535	18
Rer14	3.82E-07	0.42706658	0.261	0.146	0.01234266	18
Arpp195	4.31E-07	0.41833501	0.496	0.343	0.0139224	18
Hnrnpul12	5.82E-07	0.33430129	0.296	0.17	0.01880143	18
Tomm67	6.31E-07	0.26244111	0.617	0.42	0.02038039	18
Zcrb13	6.36E-07	0.26564229	0.322	0.187	0.02051869	18
Ncf14	7.26E-07	0.29182219	0.313	0.182	0.02342856	18
Hcls13	7.29E-07	0.38864353	0.261	0.147	0.02353999	18
Gng55	7.31E-07	0.3206549	0.857	0.654	0.02358708	18
Pet1003	8.17E-07	0.32578272	0.526	0.356	0.02638959	18
Tma76	8.74E-07	0.39274529	0.557	0.39	0.02820862	18
Rpp213	9.69E-07	0.30744322	0.261	0.147	0.0312993	18
Cyba8	9.70E-07	0.35094549	0.77	0.531	0.03132028	18
Ciao2a4	1.05E-06	0.31180411	0.261	0.146	0.03394592	18
Sec61a1	1.08E-06	0.28404052	0.296	0.169	0.03483515	18
Trappc2l2	1.11E-06	0.30939747	0.252	0.141	0.03585999	18
Swi56	1.47E-06	0.28971568	0.396	0.248	0.04750867	18
Snhg94	1.92E-06	0.29892907	0.252	0.14	0.06206481	18
Cdc42se12	1.94E-06	0.33983304	0.296	0.174	0.06278322	18
Cycs6	2.19E-06	0.3467873	0.378	0.243	0.07056132	18
Tmem1673	2.40E-06	0.27937461	0.37	0.232	0.07751319	18
Birc62	2.65E-06	0.3044652	0.278	0.162	0.08543232	18
Polr2k3	2.77E-06	0.30247543	0.383	0.246	0.08952527	18
Rtf13	3.36E-06	0.28599725	0.278	0.163	0.1086352	18
Calm35	3.80E-06	0.26514159	0.396	0.257	0.12280297	18
Sin3b1	3.99E-06	0.34408847	0.309	0.189	0.12887491	18
Psmb35	4.27E-06	0.34880063	0.435	0.298	0.13774124	18
Grb22	4.51E-06	0.32510555	0.283	0.171	0.14569195	18
Pak21	5.05E-06	0.26261184	0.365	0.233	0.16310439	18
Pitpna1	6.36E-06	0.28166493	0.287	0.173	0.20533525	18
Scp24	6.49E-06	0.30681069	0.348	0.226	0.2093724	18
Arglu12	6.89E-06	0.27455805	0.326	0.202	0.22257883	18
Smim43	7.66E-06	0.33272938	0.278	0.171	0.24733551	18
Spcs12	9.41E-06	0.25810097	0.326	0.207	0.30382595	18
Psma55	9.44E-06	0.26840967	0.296	0.182	0.30464382	18

Ndufab15	1.04E-05	0.25791178	0.33	0.207	0.33658416	18
Zyx4	1.08E-05	0.25040099	0.313	0.194	0.35003355	18
Sep-72	2.28E-05	0.29372594	0.348	0.223	0.73479195	18
Psmd84	2.68E-05	0.26558778	0.309	0.195	0.86565052	18
Kdm6b4	2.72E-05	0.26198746	0.283	0.174	0.87894394	18
Hnrnpf5	3.04E-05	0.2627015	0.461	0.321	0.98121914	18
Ythdc11	3.35E-05	0.25794165	0.278	0.171	1	18
Arhgdia2	3.95E-05	0.27154362	0.335	0.219	1	18
Tcea13	8.35E-05	0.30067399	0.278	0.179	1	18
Cggbp13	0.0001865	0.29556481	0.291	0.196	1	18
Nr4a15	0.00054725	0.29603697	0.296	0.205	1	18
Ly6d3	3.12E-69	0.96827323	0.724	0.143	1.01E-64	19
Ighm4	1.46E-52	0.98120268	0.843	0.231	4.73E-48	19
Ebf14	1.69E-52	0.71857582	0.881	0.22	5.45E-48	19
Vpreb34	1.50E-49	0.3797008	0.813	0.193	4.84E-45	19
Wfdc214	6.24E-46	1.321494	0.91	0.286	2.01E-41	19
Retnlg3	9.21E-46	1.32164007	0.836	0.27	2.97E-41	19
Lcn23	2.97E-44	1.49742381	0.858	0.299	9.59E-40	19
Mmp93	1.37E-43	1.24660693	0.746	0.22	4.42E-39	19
Cd79a4	1.75E-38	0.56628667	0.739	0.201	5.65E-34	19
Pglyrp14	3.67E-38	1.21135373	0.821	0.277	1.19E-33	19
Cd79b4	5.11E-35	0.74577088	0.619	0.169	1.65E-30	19
Ngp3	3.90E-34	1.42034158	0.754	0.28	1.26E-29	19
Ifitm64	6.32E-32	1.27285588	0.694	0.253	2.04E-27	19
Trem13	1.15E-31	1.1564135	0.567	0.176	3.71E-27	19
Camp2	2.05E-31	1.16179943	0.701	0.251	6.63E-27	19
Dnajc73	2.24E-31	0.7227067	0.642	0.211	7.24E-27	19
Mmp83	3.72E-31	1.26150934	0.612	0.198	1.20E-26	19
S100a84	1.98E-30	1.25331568	0.978	0.502	6.39E-26	19
Ltf2	3.16E-30	1.61799797	0.545	0.173	1.02E-25	19
S100a94	5.13E-30	1.30412788	0.978	0.484	1.66E-25	19
Mxd13	6.14E-30	0.96200884	0.701	0.256	1.98E-25	19
Slpi3	6.68E-30	1.183854	0.731	0.285	2.16E-25	19
G0s24	3.29E-29	1.02620042	0.672	0.237	1.06E-24	19
Hdc3	6.32E-28	0.7828926	0.597	0.199	2.04E-23	19
Il1r23	6.43E-28	0.86675183	0.567	0.183	2.08E-23	19
Ly6g3	5.48E-27	1.0577843	0.463	0.137	1.77E-22	19
Adpgk3	1.04E-26	1.17984627	0.575	0.2	3.35E-22	19
Hp6	4.71E-26	0.87588868	0.769	0.307	1.52E-21	19
Ptprcap7	8.43E-26	0.6628158	0.567	0.176	2.72E-21	19
Bach22	1.30E-25	0.6053509	0.343	0.08	4.20E-21	19
Sorl13	1.30E-25	0.94289976	0.47	0.149	4.21E-21	19
Fcrla3	1.35E-25	0.54193569	0.366	0.088	4.37E-21	19

Spib3	6.70E-25	0.96761344	0.313	0.072	2.16E-20	19
Iglc33	8.03E-25	0.61012943	0.276	0.058	2.59E-20	19
Samsn14	2.89E-24	1.05236927	0.716	0.308	9.32E-20	19
Fpr23	4.68E-24	0.8397544	0.381	0.105	1.51E-19	19
Tyrobp6	7.61E-24	0.72436363	0.925	0.413	2.46E-19	19
Cd1773	8.79E-24	0.9076907	0.5	0.166	2.84E-19	19
Chchd103	4.48E-23	0.66896567	0.493	0.151	1.45E-18	19
Lrg14	3.88E-22	0.94541166	0.5	0.175	1.25E-17	19
Atp1b14	7.93E-22	0.60310829	0.328	0.084	2.56E-17	19
Btg15	8.22E-22	0.67732811	0.97	0.623	2.65E-17	19
Srgn3	3.63E-21	0.75555042	0.94	0.512	1.17E-16	19
Dgat14	3.99E-21	0.8882167	0.515	0.188	1.29E-16	19
Mcemp15	5.23E-21	0.78104424	0.575	0.218	1.69E-16	19
Plaur5	5.25E-21	1.00217784	0.709	0.32	1.69E-16	19
Lilr4b3	7.90E-21	0.83205537	0.545	0.205	2.55E-16	19
Actb6	1.90E-20	0.80559753	0.993	0.949	6.15E-16	19
Fcer1g7	1.06E-19	0.70591456	0.851	0.398	3.41E-15	19
Lcp14	1.15E-19	0.7347363	0.709	0.323	3.71E-15	19
Lyz25	3.20E-19	0.45719395	0.851	0.402	1.03E-14	19
R3hdm44	4.94E-19	0.85699857	0.493	0.187	1.60E-14	19
Plbd14	5.23E-19	0.80845082	0.455	0.165	1.69E-14	19
Ncf24	6.69E-19	0.80623663	0.567	0.237	2.16E-14	19
Chil13	7.95E-19	0.85194356	0.321	0.095	2.57E-14	19
Cxcr22	8.05E-19	0.77810148	0.343	0.102	2.60E-14	19
Cd723	1.25E-18	0.60473216	0.328	0.093	4.03E-14	19
4930597A21I	1.47E-18	0.27791411	0.269	0.066	4.76E-14	19
Cd333	2.72E-18	0.64386623	0.313	0.091	8.77E-14	19
Slfn44	3.57E-18	0.75200098	0.343	0.106	1.15E-13	19
Cxcl22	5.06E-18	0.89706773	0.478	0.18	1.63E-13	19
Akap123	8.84E-18	0.44143436	0.284	0.075	2.85E-13	19
Ndel13	9.34E-18	0.77656996	0.418	0.148	3.01E-13	19
Alox5ap5	1.06E-17	0.70424688	0.701	0.326	3.42E-13	19
Coq72	1.95E-17	0.62827602	0.306	0.09	6.29E-13	19
Thbs15	9.53E-17	0.96689516	0.507	0.217	3.08E-12	19
Fos6	9.86E-17	0.65767405	0.739	0.362	3.18E-12	19
Cd528	1.54E-16	0.65502084	0.918	0.517	4.97E-12	19
Arhgdib5	1.93E-16	0.77141554	0.791	0.437	6.24E-12	19
Btg23	2.20E-16	0.85394664	0.687	0.343	7.12E-12	19
Litaf3	2.45E-16	0.68524458	0.597	0.266	7.90E-12	19
Slc16a33	3.12E-16	0.66100297	0.425	0.159	1.01E-11	19
Sik13	3.90E-16	0.93267479	0.366	0.129	1.26E-11	19
Rhoh2	4.31E-16	0.37184123	0.299	0.088	1.39E-11	19
Anxa14	4.63E-16	0.84985085	0.724	0.384	1.50E-11	19

Cxcr45	4.66E-16	0.67995062	0.537	0.222	1.50E-11	19
Ncf15	5.59E-16	0.82212637	0.448	0.182	1.81E-11	19
Slc7a113	6.38E-16	0.78306689	0.373	0.131	2.06E-11	19
Slc2a33	6.94E-16	0.6130987	0.313	0.1	2.24E-11	19
Nfkbid1	9.47E-16	0.91370735	0.299	0.094	3.06E-11	19
Prdx55	9.85E-16	0.7455297	0.791	0.451	3.18E-11	19
Padi43	1.53E-15	0.79968807	0.269	0.082	4.93E-11	19
Ptprc7	2.33E-15	0.5919023	0.604	0.273	7.53E-11	19
Cebpb3	2.36E-15	0.57397163	0.754	0.392	7.61E-11	19
Msrb14	2.59E-15	0.65434497	0.672	0.322	8.37E-11	19
Klhl242	3.20E-15	0.49828061	0.328	0.107	1.03E-10	19
Cybb6	4.26E-15	0.96740105	0.522	0.237	1.38E-10	19
Jund7	4.32E-15	0.3940183	0.881	0.514	1.40E-10	19
Clec4e3	6.86E-15	0.56860684	0.44	0.171	2.21E-10	19
Cd374	7.29E-15	0.54469518	0.478	0.193	2.35E-10	19
Coro1a8	8.29E-15	0.73278539	0.791	0.438	2.68E-10	19
Mcl13	8.76E-15	0.75027306	0.709	0.376	2.83E-10	19
Bcl7a3	2.17E-14	0.66931662	0.284	0.087	7.01E-10	19
Tmsb4x8	2.52E-14	0.53920268	1	0.905	8.13E-10	19
Cyfip23	2.92E-14	0.67507251	0.388	0.15	9.43E-10	19
Dhrs74	4.35E-14	0.70154416	0.478	0.206	1.40E-09	19
Spi16	4.93E-14	0.66471879	0.5	0.22	1.59E-09	19
Il1b2	5.84E-14	1.00692885	0.366	0.14	1.89E-09	19
C5ar13	1.04E-13	0.49760858	0.351	0.126	3.35E-09	19
2310001H17	1.07E-13	0.68324019	0.358	0.134	3.45E-09	19
Ets23	2.57E-13	0.65768913	0.41	0.169	8.28E-09	19
Pirb5	2.98E-13	0.68177063	0.396	0.162	9.61E-09	19
Itgb2l3	3.01E-13	0.61735262	0.276	0.092	9.71E-09	19
Grina4	3.20E-13	0.74083345	0.619	0.308	1.03E-08	19
Trem34	4.12E-13	0.71000256	0.358	0.14	1.33E-08	19
Chil33	4.28E-13	0.80973278	0.433	0.179	1.38E-08	19
Lst14	6.53E-13	0.48478218	0.56	0.267	2.11E-08	19
Gm499803	7.18E-13	0.42117157	0.291	0.096	2.32E-08	19
Tmcc13	9.56E-13	0.95571081	0.351	0.14	3.09E-08	19
Lmo46	1.53E-12	0.62232188	0.537	0.261	4.94E-08	19
Mrgpra2b3	1.66E-12	0.67865007	0.291	0.104	5.36E-08	19
Rac29	3.01E-12	0.76378839	0.724	0.411	9.71E-08	19
S100a114	3.62E-12	0.52279467	0.918	0.685	1.17E-07	19
Junb6	3.68E-12	0.55838112	0.754	0.435	1.19E-07	19
Zyx5	4.11E-12	0.56318222	0.44	0.193	1.33E-07	19
Gmfg8	5.85E-12	0.48964041	0.739	0.405	1.89E-07	19
Fam107b3	7.81E-12	0.75420021	0.455	0.214	2.52E-07	19
Adam83	9.69E-12	0.57502825	0.396	0.162	3.13E-07	19

Stk17b6	1.06E-11	0.59230435	0.619	0.321	3.42E-07	19
Prr133	1.07E-11	0.47025108	0.47	0.21	3.46E-07	19
Lmnb14	1.32E-11	0.68841325	0.567	0.297	4.26E-07	19
Ostf15	1.64E-11	0.534489	0.634	0.33	5.31E-07	19
Pafah1b34	1.82E-11	0.45683727	0.433	0.179	5.88E-07	19
Ly6c26	1.84E-11	0.38771561	0.5	0.229	5.93E-07	19
Ckap44	2.45E-11	0.55610418	0.44	0.197	7.90E-07	19
Cd537	2.99E-11	0.77509916	0.515	0.26	9.65E-07	19
Ccr12	3.92E-11	0.63822024	0.284	0.106	1.27E-06	19
Egr14	5.90E-11	0.5929396	0.41	0.181	1.91E-06	19
Gsr4	6.15E-11	0.56621509	0.59	0.312	1.99E-06	19
Pnrc17	7.69E-11	0.52717695	0.604	0.318	2.48E-06	19
Pim17	8.04E-11	0.41343947	0.657	0.341	2.60E-06	19
Map3k13	2.26E-10	0.54071206	0.254	0.091	7.31E-06	19
Plek4	2.31E-10	0.83514782	0.351	0.155	7.47E-06	19
Cyba9	2.36E-10	0.54603875	0.813	0.531	7.62E-06	19
Pilra3	2.87E-10	0.37208157	0.321	0.128	9.27E-06	19
Cnp3	2.99E-10	0.32123076	0.254	0.089	9.66E-06	19
Hcls14	3.12E-10	0.55549504	0.343	0.147	1.01E-05	19
Lsp15	3.37E-10	0.52733135	0.575	0.303	1.09E-05	19
AA4671972	3.41E-10	0.62244231	0.313	0.129	1.10E-05	19
Actn14	3.80E-10	0.65619153	0.425	0.203	1.23E-05	19
Cd24a5	3.84E-10	0.28890531	0.739	0.424	1.24E-05	19
Trib13	5.75E-10	0.64280037	0.358	0.159	1.86E-05	19
Ifi27l2a6	5.95E-10	0.71813168	0.433	0.201	1.92E-05	19
Cotl15	6.26E-10	0.50155869	0.455	0.221	2.02E-05	19
Selenok5	6.68E-10	0.50528548	0.672	0.395	2.16E-05	19
Dusp53	7.00E-10	0.32545734	0.306	0.119	2.26E-05	19
Pnkp3	8.51E-10	0.53935503	0.284	0.116	2.75E-05	19
Csf3r2	9.90E-10	0.59903371	0.284	0.114	3.20E-05	19
Vsir5	1.08E-09	0.71087319	0.321	0.141	3.50E-05	19
Ddx61	1.53E-09	0.52404405	0.455	0.227	4.94E-05	19
Hcst5	1.66E-09	0.42583623	0.448	0.219	5.35E-05	19
Glrx4	1.68E-09	0.59840792	0.328	0.145	5.43E-05	19
Syne13	2.28E-09	0.65326955	0.276	0.113	7.35E-05	19
Zfp365	2.32E-09	0.51347454	0.463	0.23	7.48E-05	19
Dok32	2.34E-09	0.4844039	0.366	0.167	7.55E-05	19
Rgs21	2.38E-09	0.76399932	0.373	0.176	7.68E-05	19
H3f3a6	2.72E-09	0.34519728	0.94	0.761	8.79E-05	19
Syk4	2.98E-09	0.61008078	0.396	0.186	9.63E-05	19
Nfkbiz3	3.36E-09	0.56847934	0.313	0.136	0.00010841	19
Tut74	4.10E-09	0.60448683	0.418	0.207	0.00013252	19
AB1246113	4.26E-09	0.42063987	0.276	0.112	0.00013757	19

Cd95	5.61E-09	0.34662126	0.649	0.342	0.00018115	19
Clec4d2	5.88E-09	0.63101002	0.299	0.128	0.00018985	19
Cdk11b3	7.54E-09	0.53843886	0.403	0.196	0.00024333	19
Ikzf12	8.51E-09	0.53592098	0.321	0.145	0.00027468	19
Fth14	9.77E-09	0.45558627	0.985	0.918	0.00031528	19
Plekha22	1.14E-08	0.32069944	0.254	0.098	0.00036943	19
Sell5	1.25E-08	0.45523385	0.396	0.193	0.00040397	19
Herpud14	1.26E-08	0.39302746	0.351	0.158	0.00040592	19
Gpsm34	1.35E-08	0.49750943	0.358	0.169	0.000436	19
Itgb25	1.51E-08	0.48021503	0.373	0.178	0.00048659	19
Adgre52	1.53E-08	0.38622498	0.328	0.147	0.00049472	19
Degs14	1.95E-08	0.47814204	0.358	0.17	0.00063055	19
Lyn6	2.28E-08	0.61886	0.425	0.218	0.00073733	19
Dusp13	2.61E-08	0.48400884	0.44	0.226	0.00084331	19
Atp6v1g15	2.78E-08	0.52822042	0.575	0.345	0.000896	19
Kctd124	2.91E-08	0.65049314	0.381	0.191	0.00093823	19
Osm4	2.96E-08	0.6304348	0.269	0.113	0.00095673	19
Arhgap302	3.10E-08	0.46479459	0.291	0.128	0.00099982	19
Ltb4r13	3.32E-08	0.53741437	0.269	0.116	0.00107287	19
Bcl103	3.35E-08	0.61910357	0.381	0.188	0.00108044	19
Nr4a16	4.62E-08	0.61990996	0.396	0.205	0.00149284	19
Slfn24	6.15E-08	0.51870807	0.343	0.164	0.00198682	19
Iqgap14	6.54E-08	0.37648316	0.657	0.393	0.00211298	19
Laptm55	7.24E-08	0.54035932	0.5	0.276	0.00233707	19
Gnai23	9.05E-08	0.42686047	0.776	0.493	0.00292223	19
Rnf1303	9.50E-08	0.54495471	0.291	0.135	0.00306709	19
Arpc55	9.63E-08	0.43170497	0.597	0.353	0.00310883	19
Myb5	9.89E-08	0.39462574	0.269	0.115	0.00319433	19
Kdm6b5	1.03E-07	0.72377685	0.351	0.174	0.00332023	19
Cap13	1.03E-07	0.40913415	0.41	0.211	0.00333909	19
Clk16	1.18E-07	0.57698923	0.507	0.286	0.00381442	19
Vasp4	1.43E-07	0.50793434	0.388	0.197	0.00462157	19
Sat13	1.59E-07	0.41698057	0.545	0.309	0.00512385	19
Stk381	1.59E-07	0.38728789	0.269	0.117	0.00513239	19
Map1lc3b4	2.03E-07	0.4088432	0.687	0.435	0.00654797	19
Rap1b4	2.18E-07	0.42589477	0.47	0.257	0.00704273	19
Skap24	2.21E-07	0.55532293	0.313	0.154	0.00712707	19
Mgst15	2.22E-07	0.45424886	0.433	0.236	0.00715408	19
Tsc22d36	2.22E-07	0.38926959	0.358	0.179	0.0071541	19
Itgam5	2.24E-07	0.41148087	0.366	0.185	0.00722996	19
Taldo15	2.51E-07	0.36932324	0.634	0.395	0.00808961	19
Mrpl334	2.58E-07	0.3442334	0.739	0.454	0.00832404	19
Samd9l2	3.09E-07	0.46273972	0.291	0.135	0.00997403	19

Tkt5	3.50E-07	0.4235713	0.515	0.3	0.01129302	19
Nfam14	4.12E-07	0.69769138	0.261	0.121	0.01329285	19
Sh3bgrl310	4.25E-07	0.39591931	0.843	0.572	0.01372617	19
Atp6v1e15	4.74E-07	0.3683396	0.59	0.351	0.01530595	19
1810026B05I	5.10E-07	0.42973657	0.261	0.118	0.01645533	19
Pten2	5.57E-07	0.34456747	0.381	0.195	0.01797273	19
Celf26	5.68E-07	0.46360555	0.358	0.185	0.018351	19
Tgoln12	7.06E-07	0.39956477	0.284	0.132	0.02279419	19
Arpc48	9.56E-07	0.47236519	0.463	0.267	0.03085415	19
Abhd21	1.11E-06	0.5128149	0.284	0.136	0.03593733	19
Mgst24	1.12E-06	0.40140189	0.284	0.135	0.03621201	19
Supt4a5	1.22E-06	0.39887897	0.493	0.284	0.03925198	19
Ncf45	1.22E-06	0.49892768	0.328	0.172	0.0392586	19
Fxyd55	1.36E-06	0.36917806	0.597	0.372	0.04399498	19
Cd144	1.61E-06	0.47300581	0.358	0.185	0.05191743	19
Sp1002	1.65E-06	0.38993606	0.254	0.118	0.05336106	19
Marcks6	1.88E-06	0.32726458	0.388	0.2	0.06061881	19
Grk24	1.91E-06	0.47010286	0.463	0.274	0.06152918	19
Actg15	1.98E-06	0.35766377	0.94	0.715	0.06395759	19
Clec4a25	2.28E-06	0.43352122	0.336	0.173	0.07373796	19
Arpc37	2.31E-06	0.42685253	0.739	0.519	0.07461327	19
Ubc3	2.50E-06	0.28663096	0.657	0.419	0.08063577	19
Aldh24	2.70E-06	0.57106211	0.448	0.27	0.08711501	19
Slc38a25	3.41E-06	0.39539026	0.59	0.373	0.10999417	19
Cytip7	4.04E-06	0.4902574	0.343	0.183	0.13029985	19
Actr24	4.11E-06	0.41139993	0.507	0.315	0.13271052	19
Cpne32	4.15E-06	0.43046478	0.306	0.16	0.13403955	19
Pgd4	4.56E-06	0.39192009	0.328	0.175	0.14720714	19
Vps37b3	4.81E-06	0.44314096	0.276	0.137	0.15520539	19
Lyst3	6.30E-06	0.25057481	0.261	0.126	0.20335903	19
Dazap23	7.69E-06	0.48139472	0.381	0.217	0.24837501	19
Ypel51	8.11E-06	0.35171495	0.254	0.122	0.2617117	19
Ptpn62	8.80E-06	0.27534193	0.269	0.135	0.28409424	19
Ppp1r183	1.24E-05	0.28496925	0.321	0.169	0.40155187	19
Samhd13	1.38E-05	0.27823055	0.336	0.179	0.44711862	19
Srsf57	1.45E-05	0.39137443	0.582	0.385	0.46713041	19
Ppp2r5a4	1.54E-05	0.43469718	0.358	0.204	0.49609969	19
Lilrb4a4	1.57E-05	0.32802916	0.328	0.177	0.50691385	19
Picalm4	1.63E-05	0.523062	0.388	0.225	0.52713761	19
Actr35	1.64E-05	0.50004104	0.552	0.347	0.52798399	19
Vps4b	1.65E-05	0.25883328	0.254	0.124	0.53155129	19
Capza13	1.73E-05	0.37406935	0.41	0.239	0.55743831	19
Serpinb1a3	1.76E-05	0.29573732	0.291	0.152	0.56941938	19

Sem16	1.85E-05	0.30428849	0.873	0.67	0.59567711	19
Mpc25	1.98E-05	0.37544384	0.418	0.256	0.63979746	19
Fosl24	2.21E-05	0.28764198	0.291	0.154	0.714384	19
Notch25	2.34E-05	0.52555656	0.366	0.212	0.75666735	19
Fam32a2	3.17E-05	0.43684247	0.306	0.17	1	19
Riok31	3.25E-05	0.30375888	0.284	0.149	1	19
Gda4	3.25E-05	0.34051229	0.373	0.21	1	19
Fosb4	3.86E-05	0.66985429	0.276	0.147	1	19
Tnrc6b	3.93E-05	0.41017308	0.276	0.148	1	19
Sbno12	3.98E-05	0.42849266	0.321	0.179	1	19
Scand16	4.18E-05	0.44004657	0.545	0.357	1	19
Arhgap453	4.18E-05	0.43287701	0.291	0.16	1	19
Klf133	4.86E-05	0.38126641	0.313	0.177	1	19
Fmnl13	5.13E-05	0.27006875	0.269	0.139	1	19
U2af22	6.24E-05	0.30284341	0.269	0.142	1	19
Ccnl15	7.16E-05	0.40650736	0.403	0.25	1	19
Akap137	8.04E-05	0.29509484	0.41	0.248	1	19
Ifitm25	8.19E-05	0.4013384	0.687	0.458	1	19
Ccnd34	8.89E-05	0.39502914	0.336	0.198	1	19
Ddx57	9.08E-05	0.25126924	0.784	0.55	1	19
Wdr261	0.00011613	0.27475848	0.306	0.17	1	19
Pkig5	0.00012535	0.27281319	0.269	0.142	1	19
Tsc22d41	0.00012998	0.26773847	0.493	0.318	1	19
Rhog4	0.00013047	0.40307645	0.299	0.175	1	19
Unc93b15	0.0001909	0.27024632	0.269	0.147	1	19
Jak11	0.00040836	0.32521195	0.269	0.155	1	19
Hipk13	0.00043583	0.33316159	0.306	0.189	1	19
Pdcd62	0.00044391	0.42020265	0.306	0.188	1	19
Ppp3ca	0.00055153	0.36494129	0.276	0.164	1	19
Selplg5	0.00066572	0.26867331	0.284	0.166	1	19
Eif15	0.00067788	0.27952207	0.925	0.839	1	19
Rsrp11	0.00095096	0.25791863	0.321	0.2	1	19
Ppp1r2	0.00140304	0.41023281	0.261	0.158	1	19
Nudt43	0.00374652	0.3179669	0.291	0.187	1	19
Lamtor46	0.00488598	0.26615169	0.403	0.282	1	19
Tnfaip24	0.00594618	0.36926416	0.276	0.187	1	19
Tpd524	0.00946939	0.28675815	0.276	0.183	1	19
Tra2b8	0.00996403	0.30900148	0.366	0.262	1	19
Prss34	0	7.62021043	0.652	0.002	0	20
Mcpt8	0	6.42583346	0.748	0.003	0	20
Ccl31	0	6.05014414	0.878	0.039	0	20
Cpa3	0	4.34153778	0.73	0.005	0	20
Rgs12	0	4.32605806	0.809	0.04	0	20

Il6	0	3.49294473	0.643	0.006	0	20
Ms4a2	0	3.19975112	0.739	0.002	0	20
Gata21	0	3.19079096	0.765	0.008	0	20
Cyp11a1	0	3.17226851	0.643	0	0	20
Cd200r3	0	2.82919475	0.574	0	0	20
Hgf	0	2.54818007	0.496	0.008	0	20
Fcer1a	0	2.49612611	0.513	0.001	0	20
Csrp3	0	2.46991229	0.565	0.003	0	20
Il4	0	2.08897042	0.313	0	0	20
Sytl3	0	1.81098161	0.304	0.005	0	20
Slc18a2	0	1.71860101	0.365	0.004	0	20
Itga2b	0	1.66716162	0.339	0.007	0	20
Rnase12	0	1.2784751	0.304	0.003	0	20
Rapsn	0	1.21893828	0.252	0.003	0	20
Ccl42	9.57E-279	6.31348526	0.835	0.059	3.09E-274	20
Tnfaip32	8.96E-199	3.12186048	0.809	0.077	2.89E-194	20
Ikzf2	2.35E-179	1.2931364	0.304	0.011	7.58E-175	20
Ctsg1	1.27E-170	3.07485678	0.522	0.035	4.09E-166	20
Csf2rb2	1.73E-169	1.78427826	0.261	0.008	5.58E-165	20
Ccl92	3.12E-160	2.92840458	0.609	0.051	1.01E-155	20
Dapp11	4.96E-153	1.98397388	0.565	0.046	1.60E-148	20
Ifitm12	7.25E-149	3.76106482	0.852	0.111	2.34E-144	20
Stx3	4.07E-145	1.00723808	0.252	0.009	1.31E-140	20
Lat2	1.03E-142	2.06190725	0.461	0.032	3.32E-138	20
Padi2	1.29E-140	1.5780917	0.322	0.016	4.16E-136	20
Csf1	2.31E-134	1.9646976	0.374	0.022	7.45E-130	20
Ier34	1.29E-128	4.39328736	0.852	0.146	4.17E-124	20
Tec	5.91E-118	1.85235193	0.452	0.037	1.91E-113	20
Cebpa	3.18E-117	1.59863961	0.33	0.02	1.03E-112	20
Nlrp32	1.57E-111	2.52379343	0.696	0.092	5.07E-107	20
Fosb5	3.46E-94	2.67965918	0.791	0.145	1.12E-89	20
Nfkbiz4	2.41E-90	2.31580621	0.757	0.134	7.78E-86	20
Nfkbia7	4.30E-90	3.11193307	0.904	0.239	1.39E-85	20
Hdc4	2.18E-89	2.88244463	0.887	0.198	7.04E-85	20
Spry21	4.17E-89	2.47709593	0.504	0.06	1.35E-84	20
Csf2rb	4.12E-84	2.15458269	0.487	0.059	1.33E-79	20
Cks25	1.94E-78	3.06735172	0.887	0.257	6.27E-74	20
3-Jun	6.66E-78	3.00859056	0.783	0.178	2.15E-73	20
Mpo1	2.32E-76	1.57604991	0.357	0.034	7.49E-72	20
Cd635	1.46E-74	2.32385559	0.896	0.272	4.70E-70	20
Plek5	7.04E-73	2.39178326	0.739	0.154	2.27E-68	20
Il3ra	1.52E-71	1.24902085	0.27	0.022	4.90E-67	20
Nkg73	9.36E-67	1.39851201	0.4	0.048	3.02E-62	20

Mapkapk31	5.50E-64	1.71520646	0.357	0.042	1.77E-59	20
Srgn4	4.86E-63	2.48115044	0.974	0.512	1.57E-58	20
Cd693	7.59E-59	1.81461062	0.513	0.086	2.45E-54	20
Fxyd56	3.46E-57	1.92411613	0.965	0.37	1.12E-52	20
Jak2	8.32E-57	1.32710348	0.357	0.045	2.69E-52	20
Rab441	1.08E-56	1.46855294	0.4	0.058	3.49E-52	20
Cyp4f183	1.26E-54	1.80559452	0.513	0.092	4.06E-50	20
Alox5	1.51E-53	1.33294056	0.391	0.057	4.86E-49	20
Ddit4	5.32E-49	1.43351065	0.278	0.032	1.72E-44	20
Hcst6	8.91E-49	1.88862416	0.739	0.218	2.88E-44	20
Neat13	1.28E-48	1.98975974	0.748	0.217	4.13E-44	20
Ubb10	2.08E-48	2.3376846	0.991	0.761	6.71E-44	20
Ms4a32	7.34E-46	1.42976178	0.304	0.041	2.37E-41	20
H3f3b5	2.91E-45	2.23496258	0.991	0.826	9.38E-41	20
Alox5ap6	3.78E-45	1.80657724	0.878	0.325	1.22E-40	20
Egr15	2.16E-44	2.34791718	0.661	0.18	6.99E-40	20
Mns1	2.71E-44	0.97952464	0.252	0.029	8.76E-40	20
Tax1bp11	1.53E-43	2.11172102	0.748	0.251	4.94E-39	20
Lilr4b4	1.60E-43	2.10317582	0.678	0.205	5.17E-39	20
Plac85	8.64E-41	2.2086417	0.739	0.278	2.79E-36	20
Prkd3	1.54E-40	1.52542425	0.348	0.058	4.98E-36	20
Nucb12	3.04E-40	1.28308928	0.435	0.087	9.80E-36	20
Jund8	4.08E-40	1.47208381	0.974	0.514	1.32E-35	20
Gcnt1	4.15E-40	1.22997391	0.252	0.032	1.34E-35	20
Osm5	5.53E-40	1.65239442	0.496	0.112	1.79E-35	20
Os9	8.50E-40	1.11272256	0.322	0.05	2.74E-35	20
Il18rap	8.64E-40	1.77172609	0.357	0.064	2.79E-35	20
Tyrobp7	8.22E-39	1.50483115	0.974	0.413	2.65E-34	20
Itgb71	2.00E-38	1.19690501	0.261	0.035	6.46E-34	20
Fcer1g8	7.80E-38	1.52309361	0.939	0.398	2.52E-33	20
Tagln28	7.21E-37	1.59377648	0.8	0.308	2.33E-32	20
Spn2	8.18E-37	1.33799537	0.296	0.047	2.64E-32	20
Myl12b6	3.61E-36	1.50648466	0.852	0.424	1.17E-31	20
Tmsb4x9	1.46E-35	1.34518862	1	0.905	4.72E-31	20
Rgs185	1.81E-35	1.55436168	0.487	0.124	5.85E-31	20
Mpc26	1.97E-35	1.7877398	0.713	0.255	6.36E-31	20
Crip18	5.97E-35	1.2394145	0.904	0.401	1.93E-30	20
Sept1	3.19E-34	1.22726383	0.4	0.084	1.03E-29	20
Dhx40	7.14E-34	1.45545475	0.374	0.076	2.31E-29	20
Arl4a2	1.01E-33	1.07071217	0.322	0.056	3.25E-29	20
Gm265321	1.12E-33	1.29536471	0.33	0.06	3.61E-29	20
Fyb2	2.81E-33	1.30979074	0.374	0.075	9.07E-29	20
Rnf1304	3.74E-33	1.59055333	0.496	0.134	1.21E-28	20

Sub19	7.73E-33	1.76536894	0.904	0.481	2.50E-28	20
Samsn15	1.95E-32	1.24704675	0.809	0.308	6.28E-28	20
Neurl3	5.93E-32	1.49568539	0.296	0.052	1.91E-27	20
Dnaja14	7.48E-32	1.67058975	0.809	0.351	2.41E-27	20
Cd96	5.56E-31	1.85805851	0.791	0.341	1.80E-26	20
Ier26	9.50E-31	1.87274011	0.696	0.279	3.07E-26	20
Gng122	1.30E-30	1.22197627	0.443	0.111	4.21E-26	20
Itm2c	1.68E-30	0.89022546	0.313	0.057	5.42E-26	20
Irak3	2.05E-30	1.02600536	0.252	0.041	6.63E-26	20
Man2b11	3.23E-30	1.11291443	0.426	0.102	1.04E-25	20
Emilin22	4.46E-30	1.23732182	0.357	0.075	1.44E-25	20
Plk32	1.07E-29	1.36013078	0.348	0.073	3.44E-25	20
Itm2b6	1.21E-29	1.3735562	0.913	0.538	3.91E-25	20
Prkacb	1.51E-29	1.036524	0.304	0.057	4.88E-25	20
B930036N10	4.53E-29	1.18572193	0.348	0.075	1.46E-24	20
Rps6ka3	5.68E-29	1.0235562	0.304	0.058	1.83E-24	20
Runx12	1.02E-28	1.29935638	0.461	0.122	3.29E-24	20
Rgs22	1.06E-28	1.61346498	0.548	0.175	3.41E-24	20
Ptprs2	1.79E-28	0.84989002	0.357	0.075	5.79E-24	20
Smim31	2.77E-28	1.0875311	0.313	0.063	8.93E-24	20
Tmbim43	4.58E-28	1.08943816	0.487	0.137	1.48E-23	20
Hk21	7.80E-28	1.09185648	0.409	0.102	2.52E-23	20
Dusp14	1.24E-27	1.35777755	0.661	0.225	3.99E-23	20
Fam129a	1.94E-27	0.75375661	0.348	0.074	6.25E-23	20
Cpne23	2.26E-27	0.97175883	0.365	0.084	7.31E-23	20
Hcar21	1.27E-26	1.08658671	0.322	0.067	4.10E-22	20
Plgrkt1	1.86E-26	0.94517353	0.357	0.082	6.01E-22	20
Ap3s12	2.31E-26	1.20837758	0.504	0.158	7.47E-22	20
Prdx67	5.67E-26	1.62474835	0.678	0.285	1.83E-21	20
Ptms4	6.81E-26	1.25678468	0.548	0.173	2.20E-21	20
Adgrg31	7.91E-26	0.97049004	0.261	0.05	2.56E-21	20
Rpl3810	9.88E-26	0.90318828	1	0.844	3.19E-21	20
Tmed33	1.55E-25	1.02207112	0.391	0.102	5.00E-21	20
Srsf58	1.93E-25	1.5931607	0.809	0.384	6.22E-21	20
Tent5a1	2.70E-25	0.99515091	0.252	0.047	8.71E-21	20
Pim18	3.04E-25	1.71629969	0.757	0.34	9.81E-21	20
Csgalnact21	6.14E-25	0.76386028	0.252	0.047	1.98E-20	20
Klf7	1.30E-24	0.98349838	0.339	0.079	4.19E-20	20
Ets14	1.74E-24	1.03885222	0.504	0.154	5.61E-20	20
Rps1110	3.00E-24	0.99345287	0.983	0.731	9.68E-20	20
Tnfaip81	1.07E-23	1.2584311	0.435	0.128	3.45E-19	20
Cd538	8.14E-23	1.29523827	0.643	0.26	2.63E-18	20
Napsa4	1.10E-22	0.97757753	0.47	0.146	3.55E-18	20

Ptpn187	1.78E-22	0.94648894	0.713	0.295	5.76E-18	20
Klf63	3.67E-22	1.37072986	0.6	0.236	1.18E-17	20
Fam107b4	6.57E-22	1.10558145	0.565	0.214	2.12E-17	20
Esyt11	6.96E-22	0.82101734	0.304	0.071	2.25E-17	20
Junb7	7.51E-22	1.43944706	0.826	0.435	2.42E-17	20
Ssr47	1.38E-21	1.29277313	0.6	0.252	4.45E-17	20
Plp23	2.83E-21	1.1799082	0.478	0.161	9.13E-17	20
Arpc1a1	3.06E-21	1.71325542	0.365	0.105	9.87E-17	20
Selenok6	5.34E-21	1.25137493	0.774	0.394	1.73E-16	20
Kdm6b6	6.80E-21	1.2743266	0.504	0.174	2.20E-16	20
Furin	7.62E-21	1.18385235	0.278	0.064	2.46E-16	20
Cebpb4	7.64E-21	0.96529168	0.826	0.392	2.47E-16	20
Atp8b41	2.45E-20	0.78228738	0.27	0.062	7.90E-16	20
Cxcr46	8.24E-20	1.0879879	0.565	0.222	2.66E-15	20
Taldo16	1.57E-19	1.02265603	0.774	0.394	5.08E-15	20
Ftl14	2.02E-19	1.42783498	0.957	0.809	6.53E-15	20
Pnrc18	3.24E-19	1.21292637	0.696	0.318	1.05E-14	20
Adk2	6.76E-19	0.88528814	0.27	0.065	2.18E-14	20
Sh3bgrl311	8.97E-19	0.95814976	0.896	0.572	2.90E-14	20
Dnajb12	9.15E-19	1.38357141	0.322	0.091	2.95E-14	20
Zc3h12a1	1.42E-18	1.22965615	0.261	0.063	4.58E-14	20
Srpk11	1.62E-18	0.82848973	0.304	0.08	5.23E-14	20
Fau8	2.29E-18	0.67038974	1	0.943	7.40E-14	20
Crlf2	2.66E-18	1.02293578	0.313	0.087	8.58E-14	20
Rab11a	3.70E-18	1.17398912	0.452	0.161	1.20E-13	20
Ccl64	8.26E-18	2.20973607	0.461	0.185	2.67E-13	20
Lcp15	9.32E-18	0.76918258	0.713	0.323	3.01E-13	20
Fcgr33	2.01E-17	0.85259068	0.383	0.123	6.50E-13	20
Zfp366	2.58E-17	1.99333884	0.53	0.23	8.33E-13	20
Nedd43	2.71E-17	0.80235632	0.583	0.224	8.75E-13	20
Ppp1r101	2.78E-17	0.86230266	0.313	0.088	8.96E-13	20
Cd84	3.02E-17	0.90406763	0.278	0.072	9.73E-13	20
Uba529	4.70E-17	0.66156588	0.983	0.759	1.52E-12	20
Rpl37a10	5.71E-17	0.68709745	1	0.905	1.84E-12	20
Dnajb9	5.72E-17	1.41288298	0.287	0.08	1.85E-12	20
Acod12	1.19E-16	1.45801863	0.278	0.075	3.84E-12	20
Trappc6a2	2.75E-16	0.51616308	0.27	0.071	8.87E-12	20
Rabac12	4.43E-16	0.84765569	0.513	0.208	1.43E-11	20
Pdia42	1.45E-15	0.86496555	0.357	0.115	4.69E-11	20
Calm24	2.22E-15	1.12841645	0.809	0.488	7.16E-11	20
Ddx58	2.43E-15	1.21403847	0.861	0.549	7.86E-11	20
Sdf2l12	4.37E-15	0.77788627	0.252	0.069	1.41E-10	20
Rfc23	5.28E-15	0.91253839	0.296	0.09	1.71E-10	20

Sqstm1	9.20E-15	1.43450568	0.357	0.121	2.97E-10	20
Rps237	1.04E-14	0.69007169	0.991	0.764	3.34E-10	20
Tmem1604	1.04E-14	0.54480167	0.409	0.144	3.37E-10	20
Pfn16	1.40E-14	0.75260418	0.939	0.751	4.53E-10	20
H2afj7	1.43E-14	0.90463576	0.748	0.434	4.61E-10	20
Hist1h1c2	1.44E-14	1.20711668	0.409	0.158	4.66E-10	20
Litaf4	1.73E-14	0.88817787	0.591	0.266	5.59E-10	20
Ifitm26	1.74E-14	0.90205972	0.791	0.457	5.61E-10	20
Anxa15	2.08E-14	1.12739781	0.687	0.385	6.71E-10	20
Gmfg9	4.52E-14	0.98175942	0.765	0.405	1.46E-09	20
Rps277	5.67E-14	0.52592459	1	0.912	1.83E-09	20
Snhg95	5.83E-14	1.29554798	0.383	0.14	1.88E-09	20
Ptpre3	8.02E-14	0.87295326	0.33	0.116	2.59E-09	20
Ifrd13	8.36E-14	1.15924434	0.417	0.165	2.70E-09	20
Zfp36l25	9.94E-14	1.07249384	0.548	0.262	3.21E-09	20
Clk17	1.15E-13	1.01168191	0.583	0.286	3.73E-09	20
Atp11b	1.19E-13	0.90131125	0.339	0.117	3.85E-09	20
Gm2a3	1.28E-13	0.66663002	0.33	0.108	4.12E-09	20
Marcksl12	1.38E-13	0.73789095	0.33	0.111	4.45E-09	20
Pfdn58	1.81E-13	0.72305259	0.8	0.461	5.85E-09	20
Cmtm77	3.50E-13	0.87746396	0.522	0.241	1.13E-08	20
Ero1l1	4.18E-13	0.95868939	0.252	0.077	1.35E-08	20
Scp25	4.25E-13	1.05835635	0.496	0.225	1.37E-08	20
Ywhaz2	6.76E-13	0.95970602	0.774	0.488	2.18E-08	20
Emb5	8.78E-13	0.83493485	0.4	0.155	2.83E-08	20
Sept111	1.19E-12	0.54063814	0.252	0.074	3.85E-08	20
Cd484	1.50E-12	0.50426276	0.252	0.075	4.85E-08	20
Clic14	1.55E-12	0.92423867	0.739	0.474	5.00E-08	20
Anp32a6	2.12E-12	0.94444178	0.557	0.283	6.86E-08	20
Dennd4a3	4.68E-12	0.73436618	0.4	0.162	1.51E-07	20
Ppp1r15a3	4.72E-12	0.80605196	0.33	0.117	1.52E-07	20
Actb7	6.09E-12	0.66503606	1	0.949	1.97E-07	20
Spcs23	7.52E-12	0.81879578	0.443	0.199	2.43E-07	20
Glipr13	8.34E-12	0.7886209	0.296	0.103	2.69E-07	20
Rpl1010	9.61E-12	0.72493141	0.843	0.606	3.10E-07	20
Creg15	1.08E-11	0.60103194	0.391	0.156	3.47E-07	20
Tle43	1.13E-11	0.82433798	0.348	0.133	3.66E-07	20
Tspo5	1.20E-11	0.73828396	0.748	0.46	3.88E-07	20
Lilrb4a5	1.21E-11	1.16345543	0.409	0.177	3.91E-07	20
Scaf112	1.27E-11	0.80363375	0.461	0.201	4.10E-07	20
Fli12	1.29E-11	0.82412842	0.322	0.12	4.18E-07	20
Stk17b7	1.53E-11	0.92616516	0.6	0.321	4.92E-07	20
Ubc4	1.93E-11	1.13884134	0.722	0.419	6.24E-07	20

Nabp13	2.20E-11	0.8788171	0.252	0.082	7.12E-07	20
Ppp1r111	3.03E-11	0.78237009	0.374	0.154	9.77E-07	20
Clint12	3.52E-11	0.77507063	0.417	0.178	1.14E-06	20
Syne14	3.89E-11	0.94571248	0.304	0.113	1.26E-06	20
Pabpc15	4.21E-11	0.91646375	0.861	0.586	1.36E-06	20
Tmem2588	4.57E-11	0.75160186	0.652	0.387	1.48E-06	20
Fermt33	5.96E-11	0.75147026	0.348	0.139	1.93E-06	20
Gnai3	6.33E-11	0.79673814	0.357	0.143	2.04E-06	20
Clec12a5	6.76E-11	0.95690542	0.339	0.14	2.18E-06	20
Lmo47	7.21E-11	0.88852865	0.513	0.261	2.33E-06	20
Ppib5	7.90E-11	0.74111052	0.6	0.337	2.55E-06	20
Rps310	8.47E-11	0.55163325	0.939	0.709	2.73E-06	20
Sod23	1.01E-10	0.87901592	0.313	0.121	3.25E-06	20
Cenpx5	1.06E-10	0.5642573	0.348	0.136	3.41E-06	20
Fkbp24	1.21E-10	0.60575236	0.383	0.16	3.90E-06	20
Chd2	1.57E-10	0.50611869	0.252	0.083	5.07E-06	20
Grcc106	1.60E-10	0.67982357	0.539	0.272	5.18E-06	20
Rpl99	1.74E-10	0.52456451	0.93	0.775	5.60E-06	20
Rpl299	1.86E-10	0.56464139	0.852	0.577	5.99E-06	20
Mrps246	2.29E-10	0.67631834	0.4	0.173	7.39E-06	20
Skil3	2.50E-10	0.72684898	0.435	0.189	8.07E-06	20
Selenos4	2.84E-10	0.43674529	0.33	0.127	9.18E-06	20
H2-T234	3.04E-10	0.64820343	0.409	0.175	9.81E-06	20
Dad18	3.08E-10	0.67744001	0.504	0.245	9.95E-06	20
Tgoln13	3.36E-10	0.75378138	0.33	0.131	1.08E-05	20
Ptp4a21	3.53E-10	0.85354863	0.443	0.204	1.14E-05	20
Ergic3	3.84E-10	0.44225062	0.261	0.089	1.24E-05	20
Actg16	4.23E-10	0.9212351	0.93	0.716	1.37E-05	20
Jak12	4.80E-10	0.82246167	0.365	0.154	1.55E-05	20
Crlf3	4.99E-10	0.94385147	0.27	0.1	1.61E-05	20
Rpl119	5.23E-10	0.53719362	0.948	0.742	1.69E-05	20
Serf25	5.30E-10	0.56254565	0.913	0.729	1.71E-05	20
Rpl3910	8.02E-10	0.52714372	0.974	0.847	2.59E-05	20
Leprotl11	8.59E-10	0.87227513	0.304	0.12	2.77E-05	20
Tomm77	8.63E-10	0.63149913	0.765	0.485	2.79E-05	20
Tmco15	8.70E-10	0.43906812	0.383	0.16	2.81E-05	20
Atp6ap21	9.29E-10	0.41602032	0.27	0.096	3.00E-05	20
Rac210	1.07E-09	0.63000665	0.696	0.411	3.47E-05	20
AI6622708	1.26E-09	0.5527445	0.339	0.14	4.07E-05	20
Casp31	1.33E-09	0.43511958	0.304	0.114	4.30E-05	20
Surf4	1.62E-09	0.40839805	0.296	0.112	5.24E-05	20
Sec11a4	1.65E-09	0.33530372	0.339	0.135	5.34E-05	20
Rps2410	1.74E-09	0.44765343	0.983	0.856	5.61E-05	20

Rps139	1.85E-09	0.46126648	0.957	0.797	5.98E-05	20
Hspa54	2.10E-09	0.82748192	0.6	0.358	6.79E-05	20
2310001H17I	2.46E-09	0.86906458	0.322	0.135	7.94E-05	20
Chic22	2.49E-09	0.62075626	0.33	0.138	8.03E-05	20
Fis15	5.02E-09	0.56654111	0.652	0.375	0.00016213	20
Cuedc21	5.46E-09	0.52019336	0.304	0.123	0.00017643	20
Tuba1c1	5.63E-09	0.86369182	0.296	0.121	0.00018189	20
Map2k31	5.77E-09	0.64904459	0.261	0.098	0.00018617	20
Sec11c5	5.96E-09	0.55752732	0.557	0.313	0.00019229	20
Tle56	6.68E-09	0.74982333	0.478	0.248	0.00021575	20
Arpc24	6.81E-09	0.66601216	0.713	0.45	0.00021972	20
Reep55	7.23E-09	0.52842555	0.522	0.272	0.00023355	20
Sec61b7	8.35E-09	0.75492416	0.661	0.45	0.00026951	20
2410006H16I	8.63E-09	0.69973079	0.67	0.401	0.00027875	20
Gng22	8.94E-09	0.72233794	0.278	0.109	0.00028871	20
Gng105	9.09E-09	0.4080806	0.322	0.132	0.00029351	20
Trmt1128	1.36E-08	0.4283634	0.496	0.244	0.00044047	20
Ddost3	1.41E-08	0.45534823	0.296	0.119	0.00045662	20
Pdcd47	1.45E-08	0.50675684	0.496	0.244	0.00046803	20
Igf1r	1.50E-08	0.81668534	0.278	0.113	0.00048574	20
Vamp86	1.52E-08	0.60745118	0.513	0.281	0.00049155	20
Cuta6	1.55E-08	0.63323261	0.374	0.174	0.00049922	20
Tmed22	1.60E-08	0.67203628	0.435	0.213	0.00051675	20
Gpi15	1.69E-08	0.69243686	0.539	0.287	0.00054438	20
Hsp90b16	1.90E-08	0.6956643	0.626	0.381	0.00061465	20
Emp37	1.96E-08	0.57946021	0.574	0.324	0.00063273	20
Rpl3410	2.00E-08	0.45934288	0.957	0.827	0.00064416	20
Arpc38	2.01E-08	0.54753767	0.783	0.519	0.00064875	20
Srsf76	2.05E-08	0.74232794	0.443	0.225	0.00066071	20
Ly6e8	2.22E-08	0.40263473	0.696	0.407	0.00071652	20
Dusp54	2.97E-08	0.97726906	0.287	0.12	0.00095826	20
Atp6v0b6	3.36E-08	0.64930468	0.522	0.285	0.00108391	20
Srsf26	3.58E-08	0.6366262	0.504	0.262	0.00115627	20
Gpx45	3.74E-08	0.68988356	0.661	0.4	0.00120792	20
Eif16	6.25E-08	0.44763668	0.939	0.839	0.00201785	20
Dnajb63	6.28E-08	0.92177121	0.452	0.239	0.00202789	20
Ppp3ca1	6.65E-08	0.48349974	0.357	0.164	0.00214841	20
Tln11	7.72E-08	0.6164495	0.478	0.257	0.00249325	20
Arhgap454	9.08E-08	0.86985511	0.339	0.16	0.00293211	20
Dock8	1.03E-07	0.58852927	0.261	0.107	0.0033236	20
Zyx6	1.11E-07	0.90385477	0.374	0.194	0.00357104	20
1810037I17R	1.16E-07	0.64247317	0.565	0.351	0.00375193	20
Serinc37	1.17E-07	0.33633454	0.643	0.362	0.00376322	20

Cux12	1.34E-07	0.31016115	0.33	0.144	0.00431015	20
Ost48	1.59E-07	0.61391291	0.574	0.351	0.00511888	20
Dpm37	1.62E-07	0.46346104	0.478	0.256	0.00523388	20
Fos7	1.73E-07	0.62053038	0.591	0.363	0.00557682	20
Aldoa4	1.81E-07	0.49472169	0.678	0.422	0.00585331	20
Ninj12	1.86E-07	0.52370166	0.252	0.1	0.00601013	20
Manf4	1.93E-07	0.79395858	0.4	0.21	0.00622303	20
Slc3a21	2.28E-07	0.37537606	0.278	0.116	0.0073615	20
Rps2910	2.39E-07	0.35622235	1	0.955	0.00770366	20
Krtcap28	2.49E-07	0.65239276	0.47	0.256	0.00803744	20
Notch26	2.75E-07	0.48975948	0.417	0.212	0.00887887	20
Gtf2b2	4.09E-07	0.44524999	0.278	0.123	0.01321703	20
Tma77	4.40E-07	0.48937979	0.635	0.39	0.01421243	20
Ift202	4.45E-07	0.41338446	0.252	0.106	0.01438141	20
Arhgef12	4.57E-07	0.7430706	0.313	0.147	0.01476187	20
Rpl3210	5.14E-07	0.4533602	0.965	0.733	0.01659028	20
Ppp1r12a	5.39E-07	0.74171465	0.287	0.133	0.01739343	20
Tmbim65	5.66E-07	0.53293505	0.522	0.299	0.01828459	20
Lamtor25	5.75E-07	0.6386862	0.47	0.27	0.01856176	20
Ddx3x1	6.28E-07	0.64238688	0.426	0.229	0.02027211	20
Churc13	6.45E-07	0.43751948	0.296	0.133	0.02081905	20
Flot11	7.56E-07	0.52535775	0.261	0.113	0.02440219	20
Coro1a9	9.45E-07	0.42150298	0.73	0.439	0.03049903	20
Ctsd2	9.61E-07	0.44002037	0.452	0.237	0.03103071	20
Yy1	1.02E-06	0.48155545	0.261	0.114	0.03280366	20
Ssr23	1.21E-06	0.3206195	0.304	0.14	0.03892688	20
Hsbp12	1.24E-06	0.36219597	0.4	0.203	0.04011959	20
Pcbd24	1.36E-06	0.52881486	0.252	0.112	0.04397918	20
Malat15	1.42E-06	0.25400988	1	0.957	0.04587499	20
Hmgb26	1.50E-06	0.55527055	0.843	0.643	0.04850785	20
Cnot31	1.58E-06	0.41697134	0.278	0.124	0.050969	20
Rap1a2	1.63E-06	0.52091105	0.365	0.19	0.05264169	20
Nsd3	1.64E-06	0.55747788	0.287	0.134	0.05301046	20
Fmc12	1.68E-06	0.31900436	0.261	0.114	0.05416593	20
Atp5h8	1.87E-06	0.52011235	0.687	0.478	0.06021807	20
Sf3b56	1.91E-06	0.31604328	0.417	0.213	0.06159771	20
Ndufb116	1.93E-06	0.51673396	0.53	0.326	0.06245525	20
Cops93	1.99E-06	0.5508509	0.478	0.276	0.0641481	20
Serpinb1a4	2.02E-06	0.62908922	0.313	0.152	0.06520506	20
Ostf16	2.07E-06	0.535777	0.539	0.331	0.06686072	20
Syf24	2.10E-06	0.45062562	0.391	0.203	0.06780233	20
Smim44	2.15E-06	0.46886574	0.339	0.171	0.06937337	20
Laptm56	2.22E-06	0.53662199	0.478	0.276	0.07161376	20

BC0055372	3.66E-06	0.51109994	0.4	0.218	0.11807276	20
Mlec1	3.78E-06	0.35738895	0.261	0.117	0.12216016	20
Grb23	3.79E-06	0.34926062	0.339	0.172	0.12251046	20
Hnrnpf6	3.80E-06	0.38626638	0.539	0.322	0.12280131	20
Ikzf13	3.91E-06	0.55149393	0.296	0.145	0.12637168	20
Metap25	4.20E-06	0.45030195	0.435	0.245	0.13574631	20
Itgb26	4.55E-06	0.57024609	0.339	0.179	0.1469891	20
Etfb5	4.56E-06	0.36175475	0.33	0.165	0.14713828	20
Rpl36al10	4.64E-06	0.44425607	0.748	0.514	0.14983165	20
Calr5	4.85E-06	0.54889026	0.426	0.246	0.15664696	20
Naca9	4.95E-06	0.37341357	0.835	0.575	0.15968059	20
Rpl1810	5.24E-06	0.37841203	0.896	0.716	0.16925047	20
Micos135	5.63E-06	0.49863177	0.383	0.206	0.18180563	20
S100a104	5.74E-06	0.25285676	0.713	0.407	0.18537333	20
Sptssa5	5.80E-06	0.39080756	0.252	0.115	0.18714175	20
Elf15	6.11E-06	0.38487096	0.296	0.14	0.19712176	20
H2afz6	6.15E-06	0.6722576	0.757	0.577	0.19862677	20
Bax4	6.35E-06	0.30305964	0.313	0.152	0.20507353	20
Gls	6.87E-06	0.5957353	0.278	0.135	0.22168389	20
Psenen5	6.97E-06	0.25902041	0.461	0.257	0.22500923	20
Atp6v1f9	7.16E-06	0.42597984	0.53	0.325	0.23120512	20
Aamp2	7.59E-06	0.34173461	0.278	0.13	0.24504928	20
Psmb88	7.93E-06	0.2905981	0.409	0.218	0.25612792	20
Cox8a8	8.04E-06	0.40599689	0.817	0.687	0.25952875	20
Lgals93	8.24E-06	0.27210815	0.296	0.141	0.26591361	20
Myh93	8.65E-06	0.63197764	0.548	0.354	0.27925978	20
Calm36	8.84E-06	0.59232747	0.435	0.258	0.28546384	20
Hist1h2bc2	9.82E-06	1.11859071	0.296	0.152	0.31696126	20
Arf41	9.86E-06	0.42338311	0.374	0.198	0.31842848	20
Prkar1a2	1.00E-05	0.57533498	0.4	0.229	0.3235419	20
Rps27a9	1.03E-05	0.30512156	0.974	0.894	0.33105134	20
Gatad12	1.11E-05	0.34096592	0.27	0.127	0.35704242	20
Arpc56	1.16E-05	0.5363867	0.565	0.354	0.37362706	20
Cdc424	1.23E-05	0.47630236	0.722	0.487	0.39802625	20
Gng56	1.72E-05	0.47213038	0.791	0.655	0.55483358	20
Mien12	1.76E-05	0.52237133	0.261	0.129	0.56693443	20
Atf43	1.83E-05	0.42136415	0.409	0.235	0.59174653	20
Lbr4	1.92E-05	0.43062969	0.435	0.257	0.62144143	20
Limd25	1.98E-05	0.53490379	0.391	0.23	0.63822136	20
Pdia35	2.23E-05	0.33574237	0.443	0.259	0.71949647	20
Psme19	2.25E-05	0.37403958	0.513	0.31	0.72726487	20
Rps710	2.51E-05	0.29762182	0.913	0.732	0.80960689	20
Srrm24	2.77E-05	0.36406839	0.53	0.321	0.89443853	20

Pdcd6ip	2.83E-05	0.3173804	0.261	0.126	0.91324635	20
Smap11	3.07E-05	0.37989044	0.252	0.122	0.99217249	20
Sat14	3.32E-05	0.80309192	0.478	0.31	1	20
Swi57	3.57E-05	0.4052616	0.426	0.248	1	20
Hnrnph14	3.82E-05	0.4385888	0.4	0.226	1	20
Ptbp34	3.85E-05	0.53998334	0.452	0.277	1	20
Ndufb108	4.53E-05	0.38043285	0.417	0.246	1	20
Alyref6	4.68E-05	0.28538676	0.409	0.227	1	20
Akap138	4.73E-05	0.50903189	0.426	0.248	1	20
Ndfip11	4.86E-05	0.29953499	0.339	0.182	1	20
Arl6ip17	4.96E-05	0.34483288	0.374	0.211	1	20
Fosl25	5.10E-05	0.54596165	0.287	0.154	1	20
Ap2s14	5.47E-05	0.40650258	0.348	0.195	1	20
Rsrc22	5.72E-05	0.50521009	0.357	0.203	1	20
Ube2l34	5.83E-05	0.40565904	0.365	0.208	1	20
Ndufa110	5.83E-05	0.47412852	0.522	0.36	1	20
Ubl57	5.95E-05	0.38139699	0.704	0.522	1	20
Ndufa48	6.34E-05	0.40969918	0.643	0.467	1	20
Arhgdib6	6.39E-05	0.25789955	0.73	0.438	1	20
Cisd24	6.61E-05	0.31372146	0.278	0.141	1	20
Ncor1	6.86E-05	0.44714277	0.365	0.21	1	20
Rheb3	6.87E-05	0.44988066	0.261	0.133	1	20
Hist1h1b3	6.95E-05	0.80174757	0.252	0.129	1	20
Mpc13	6.99E-05	0.25104287	0.339	0.184	1	20
Capzb4	0.00010539	0.40112886	0.478	0.299	1	20
Rpl219	0.00011734	0.28558965	0.887	0.722	1	20
Cd476	0.00012305	0.39087099	0.583	0.404	1	20
Atox13	0.00012543	0.27178155	0.591	0.395	1	20
Tmem50a2	0.00013322	0.26756039	0.339	0.19	1	20
Slbp3	0.00013748	0.30333773	0.278	0.146	1	20
Ccnl16	0.00014166	0.52034792	0.409	0.251	1	20
Selenoh3	0.00014542	0.27416752	0.339	0.185	1	20
Gm118087	0.00016005	0.49719028	0.313	0.174	1	20
Actr25	0.00016074	0.32480599	0.504	0.315	1	20
Dok33	0.00018078	0.42723838	0.296	0.167	1	20
Rpl199	0.00019343	0.25916989	0.948	0.762	1	20
Pold43	0.00020343	0.55216638	0.27	0.147	1	20
Rab211	0.0002314	0.35033377	0.287	0.157	1	20
Dusp114	0.0002748	0.54440793	0.33	0.193	1	20
Rps1010	0.00029409	0.25148282	0.948	0.805	1	20
Arpc1b4	0.00034011	0.3285469	0.643	0.441	1	20
Trappc4	0.00036011	0.25975334	0.261	0.141	1	20
Rnasek4	0.00037315	0.3536947	0.443	0.291	1	20

Tomm68	0.00037537	0.30126426	0.609	0.421	1	20
Tubb4b4	0.00037792	0.46054814	0.313	0.185	1	20
Hist1h2ap4	0.0004472	0.50219936	0.391	0.253	1	20
Ndufb26	0.00048826	0.42145649	0.374	0.227	1	20
Gabarapl23	0.0004923	0.28647666	0.417	0.262	1	20
Cetn33	0.00054204	0.27797903	0.296	0.166	1	20
Mbnl16	0.00054484	0.26165616	0.504	0.33	1	20
Phf20l1	0.00063357	0.55601694	0.296	0.177	1	20
Ddx39b4	0.00069376	0.25095334	0.313	0.182	1	20
Ube2d32	0.00069421	0.31016568	0.539	0.364	1	20
Pet1004	0.00071604	0.31965856	0.522	0.357	1	20
Clec4e4	0.00076008	0.34208392	0.296	0.172	1	20
Uqcrh8	0.00078866	0.25095488	0.704	0.516	1	20
Arf14	0.00088618	0.2739546	0.443	0.287	1	20
Ppp2r5a5	0.00089223	0.46016395	0.33	0.205	1	20
Dgat15	0.00093293	0.40570746	0.313	0.189	1	20
Cope3	0.00094527	0.29585129	0.296	0.176	1	20
Lamtor47	0.00094712	0.43141358	0.417	0.282	1	20
Trappc2l3	0.00096042	0.30708388	0.252	0.142	1	20
Rpp214	0.00103311	0.26742741	0.261	0.147	1	20
Pafah1b13	0.00112121	0.25498133	0.409	0.262	1	20
Nfe2l21	0.00129551	0.39107672	0.278	0.166	1	20
Rasgrp25	0.0013822	0.33882762	0.261	0.15	1	20
Smc62	0.00138774	0.41828785	0.252	0.143	1	20
Vdac21	0.00146989	0.29497159	0.357	0.226	1	20
Ube2s3	0.0015332	0.25378483	0.461	0.308	1	20
Cnn22	0.00154713	0.630818	0.261	0.156	1	20
Rer15	0.00155804	0.27584558	0.252	0.147	1	20
Denr1	0.00158228	0.32250215	0.261	0.148	1	20
Gnb23	0.00164763	0.32723768	0.522	0.369	1	20
Cggbp14	0.001807	0.31043678	0.322	0.196	1	20
Kmt2e	0.00193335	0.29782596	0.261	0.152	1	20
Myl63	0.00203939	0.27075035	0.861	0.707	1	20
Mrps146	0.00215167	0.30160587	0.313	0.193	1	20
Ndufa36	0.00220671	0.26054605	0.574	0.411	1	20
Hist1h1e2	0.00234948	0.43954333	0.261	0.157	1	20
Atp6v0d11	0.00239968	0.46337634	0.252	0.151	1	20
Chmp4b3	0.00256182	0.44452444	0.391	0.262	1	20
Ldha2	0.00297027	0.3604407	0.539	0.396	1	20
Ccdc123	0.00327795	0.296684	0.339	0.219	1	20
Brk13	0.00372501	0.28891935	0.313	0.197	1	20
Sell6	0.00415696	0.44911368	0.304	0.193	1	20
Iqgap15	0.00514429	0.33981105	0.522	0.394	1	20

Mxd14	0.00595926	0.45204981	0.365	0.258	1	20
Tmed51	0.00614269	0.25442069	0.252	0.156	1	20
S100a135	0.0066911	0.26686733	0.417	0.292	1	20
Map1lc3b5	0.00966986	0.37567702	0.565	0.435	1	20
Igha	0	8.85787703	0.596	0.01	0	21
Jchain	0	8.15562335	0.719	0.005	0	21
Cela3b	0	5.388031	0.27	0.003	0	21
Rnase1	0	4.67655595	0.27	0.003	0	21
Reg3a	0	4.45012015	0.281	0.003	0	21
Spink1	0	4.37219041	0.27	0.003	0	21
Sycn	0	4.3419298	0.258	0.002	0	21
Reg3g	0	4.0035814	0.27	0.002	0	21
Dmbt1	0	3.6980018	0.281	0.003	0	21
Iglv1	0	3.5788311	0.562	0.007	0	21
Prg2	0	2.40221318	0.404	0.001	0	21
Iglv3	0	1.65479989	0.472	0.004	0	21
Derl3	0	1.54964029	0.393	0.002	0	21
Cacna1s	0	1.15951474	0.315	0	0	21
Ctla4	0	1.14459838	0.281	0.003	0	21
Tnfrsf17	0	0.93188996	0.326	0	0	21
Oosp1	0	0.8437765	0.281	0.003	0	21
Fam214a	5.32E-294	1.41125953	0.506	0.014	1.72E-289	21
Cpa1	2.18E-292	4.98703732	0.292	0.005	7.04E-288	21
Epcam	1.09E-286	1.14335297	0.258	0.003	3.53E-282	21
Lax1	6.06E-268	0.74418433	0.326	0.006	1.96E-263	21
Cpb1	6.68E-267	5.16848577	0.27	0.004	2.16E-262	21
Pnliprp1	2.95E-249	5.52633076	0.281	0.005	9.53E-245	21
Gm5547	1.62E-245	0.76074365	0.27	0.005	5.24E-241	21
Cela2a	6.25E-236	5.36150409	0.27	0.005	2.02E-231	21
Clps	2.23E-234	5.9920509	0.27	0.005	7.20E-230	21
Klk11	5.52E-223	3.73788604	0.27	0.005	1.78E-218	21
2210010C04I	1.69E-214	5.73782891	0.27	0.005	5.47E-210	21
Eaf2	1.54E-209	1.0873574	0.337	0.009	4.96E-205	21
Try5	7.22E-171	6.33624046	0.281	0.008	2.33E-166	21
Iglc21	1.79E-161	3.34867692	0.663	0.046	5.79E-157	21
Reg1	6.21E-155	6.71548829	0.281	0.009	2.00E-150	21
Reg2	3.42E-152	6.25430332	0.281	0.009	1.10E-147	21
Iglc11	1.14E-151	6.23782435	0.584	0.038	3.69E-147	21
Reg3b	5.32E-123	6.63762569	0.281	0.011	1.72E-118	21
E330020D12	5.50E-118	0.91527655	0.371	0.019	1.78E-113	21
Prss2	6.03E-116	6.80060874	0.281	0.012	1.95E-111	21
Iglc34	5.81E-112	2.04102966	0.629	0.057	1.88E-107	21
Txndc5	5.02E-106	2.2406825	0.663	0.07	1.62E-101	21

Edem21	2.51E-98	1.19239719	0.472	0.037	8.11E-94	21
Xbp13	4.73E-89	2.58662779	0.764	0.117	1.53E-84	21
Tram2	3.58E-88	0.70477731	0.326	0.02	1.16E-83	21
Mzb14	4.77E-88	3.51435078	0.697	0.098	1.54E-83	21
Prdx43	1.13E-76	1.49776327	0.539	0.061	3.65E-72	21
Ctrb1	7.53E-69	7.51259954	0.281	0.02	2.43E-64	21
Creld21	1.42E-65	1.23099341	0.427	0.044	4.58E-61	21
Trp53inp1	6.78E-63	1.36719546	0.551	0.073	2.19E-58	21
Tent5c1	3.38E-62	0.88701802	0.472	0.054	1.09E-57	21
Fkbp11	3.98E-59	0.87710622	0.371	0.036	1.29E-54	21
Edem1	6.21E-55	1.48597855	0.528	0.08	2.01E-50	21
Igkc2	6.47E-51	7.55977537	0.708	0.198	2.09E-46	21
Sel1l	1.43E-49	0.89286132	0.36	0.041	4.61E-45	21
Pdia43	3.56E-47	1.83555414	0.607	0.115	1.15E-42	21
Rnase62	1.10E-44	0.44006937	0.326	0.036	3.54E-40	21
Sdf2l13	2.30E-44	1.24187687	0.449	0.068	7.42E-40	21
Ssr48	2.41E-44	2.57664713	0.764	0.252	7.78E-40	21
Slamf72	7.87E-44	0.57796773	0.337	0.039	2.54E-39	21
St6gal1	4.67E-43	0.50037642	0.258	0.024	1.51E-38	21
Rexo23	7.60E-41	1.10207975	0.573	0.111	2.45E-36	21
Fndc3a	2.33E-40	0.38031378	0.371	0.05	7.53E-36	21
Ell2	2.48E-39	0.6003772	0.382	0.053	7.99E-35	21
Spint22	4.92E-38	0.77124483	0.438	0.072	1.59E-33	21
Tmem176a1	6.49E-37	0.91904342	0.449	0.076	2.09E-32	21
Tmem176b2	1.01E-36	0.9838932	0.584	0.119	3.25E-32	21
Selenom2	2.61E-36	0.73293194	0.36	0.052	8.44E-32	21
Sdc1	6.97E-36	0.33392823	0.303	0.038	2.25E-31	21
Fkbp25	2.18E-34	1.28877564	0.629	0.16	7.05E-30	21
Herpud15	3.06E-32	1.42557366	0.618	0.157	9.88E-28	21
Cd743	8.16E-32	0.72938897	0.685	0.176	2.63E-27	21
Glipr14	2.51E-31	1.10403947	0.483	0.102	8.10E-27	21
Selenos5	1.39E-30	0.99622276	0.551	0.127	4.49E-26	21
Dnajc31	2.71E-29	0.7583905	0.461	0.095	8.74E-25	21
Gm302112	2.73E-29	0.78794828	0.371	0.064	8.83E-25	21
Spcs13	4.95E-29	1.39629387	0.663	0.206	1.60E-24	21
Shisa53	1.77E-28	0.69351665	0.517	0.114	5.72E-24	21
Fut81	2.44E-26	0.31095942	0.27	0.04	7.89E-22	21
Sec11c6	5.19E-26	1.64754677	0.73	0.313	1.68E-21	21
Man1a	1.07E-25	0.33492065	0.315	0.053	3.45E-21	21
Krtcap29	1.20E-25	1.40066835	0.719	0.255	3.89E-21	21
Ly6a3	5.24E-24	1.02400045	0.562	0.148	1.69E-19	21
Bet11	5.82E-24	0.61384251	0.315	0.058	1.88E-19	21
Zbtb201	1.33E-23	0.46729435	0.371	0.074	4.29E-19	21

Spcs24	1.90E-23	1.14979753	0.618	0.198	6.13E-19	21
Gstp17	3.61E-23	0.90568966	0.539	0.147	1.17E-18	21
Txndc11	3.69E-23	0.55668181	0.281	0.048	1.19E-18	21
Spcs31	1.83E-22	0.54661145	0.438	0.104	5.90E-18	21
Cd694	2.37E-22	0.6552216	0.393	0.087	7.65E-18	21
Derl1	3.58E-22	0.42062664	0.382	0.083	1.16E-17	21
Bst22	4.11E-22	0.4399475	0.551	0.149	1.33E-17	21
H132	1.97E-21	0.73000704	0.506	0.138	6.35E-17	21
Cnpy22	2.49E-21	0.65981091	0.393	0.091	8.03E-17	21
Rabac13	3.83E-21	0.87409361	0.64	0.208	1.24E-16	21
Ube2j1	3.83E-20	0.48872297	0.393	0.094	1.24E-15	21
Fosb6	8.26E-20	0.81687174	0.506	0.147	2.67E-15	21
Manf5	1.44E-19	1.37023674	0.607	0.209	4.64E-15	21
Mef2c6	4.78E-19	0.61032056	0.528	0.157	1.54E-14	21
Lman11	3.50E-18	0.52738925	0.371	0.09	1.13E-13	21
Sec61b8	1.42E-17	1.23578818	0.798	0.449	4.58E-13	21
Cited23	1.86E-17	0.87773379	0.416	0.118	6.00E-13	21
Ift203	5.01E-17	0.6371963	0.393	0.106	1.62E-12	21
Nucb13	1.16E-16	0.43045017	0.348	0.088	3.74E-12	21
Nupr1	2.33E-16	3.48250842	0.258	0.061	7.54E-12	21
Rpl36al11	3.08E-16	1.05529653	0.82	0.514	9.96E-12	21
Erp441	4.49E-16	0.2990047	0.27	0.058	1.45E-11	21
Slc3a22	7.87E-16	0.4931197	0.404	0.115	2.54E-11	21
Dad19	1.21E-15	0.64552708	0.652	0.244	3.90E-11	21
Dnajb111	3.61E-15	0.64119985	0.303	0.076	1.17E-10	21
Serp15	4.16E-15	0.85849199	0.685	0.293	1.34E-10	21
Dnajb91	4.68E-15	0.48274551	0.315	0.08	1.51E-10	21
Tmed104	2.38E-14	0.66645663	0.584	0.225	7.68E-10	21
Tram12	2.41E-14	0.52948889	0.506	0.169	7.77E-10	21
Pdia63	2.66E-14	0.51197839	0.483	0.157	8.60E-10	21
Mrpl576	2.86E-14	0.53279631	0.528	0.187	9.25E-10	21
Gm499804	4.82E-14	0.49317892	0.348	0.097	1.56E-09	21
Hsp90b17	9.79E-14	1.88244343	0.708	0.38	3.16E-09	21
Odc12	1.44E-13	0.65747434	0.404	0.128	4.66E-09	21
Cdc42se2	2.23E-13	0.31752739	0.258	0.062	7.20E-09	21
Ddrgk12	3.76E-13	0.36479775	0.292	0.077	1.21E-08	21
Selenow8	1.41E-12	0.60102337	0.64	0.266	4.54E-08	21
Phgdh2	1.61E-12	0.4491498	0.348	0.103	5.20E-08	21
22-Mar	1.98E-12	0.39061696	0.292	0.079	6.40E-08	21
Ppib6	3.52E-12	0.98814327	0.697	0.337	1.14E-07	21
Srpr	3.92E-12	0.3204286	0.326	0.095	1.27E-07	21
Ddost4	8.98E-12	0.39527673	0.371	0.119	2.90E-07	21
Mt14	1.01E-11	1.46559839	0.551	0.243	3.28E-07	21

Srp97	1.72E-11	0.85248294	0.596	0.264	5.55E-07	21
Tceal93	5.31E-11	0.40229361	0.427	0.15	1.71E-06	21
Cope4	5.54E-11	0.5361716	0.461	0.175	1.79E-06	21
Dpm38	5.79E-11	0.46129878	0.607	0.256	1.87E-06	21
Ubxn41	1.21E-10	0.4943281	0.36	0.122	3.89E-06	21
Mydgf1	1.54E-10	0.38823203	0.315	0.098	4.98E-06	21
Pycard4	2.13E-10	0.96382345	0.393	0.143	6.87E-06	21
Kdelr1	6.52E-10	0.39068229	0.281	0.087	2.11E-05	21
Selenok7	7.30E-10	0.86904938	0.685	0.395	2.36E-05	21
Tmem1472	7.45E-10	0.55136351	0.258	0.078	2.40E-05	21
Tmem1605	8.94E-10	0.28856186	0.393	0.144	2.89E-05	21
Slpi4	9.52E-10	1.96221671	0.517	0.287	3.07E-05	21
Tsc22d37	1.24E-09	0.28235848	0.461	0.179	4.01E-05	21
Magt1	1.71E-09	0.33135428	0.281	0.088	5.51E-05	21
Tmed23	3.33E-09	0.28979595	0.517	0.213	0.00010737	21
Mtdh3	4.74E-09	0.30699499	0.438	0.174	0.00015307	21
Tmem1674	1.63E-08	0.36581488	0.528	0.232	0.00052767	21
Nme27	2.02E-08	0.49193155	0.798	0.412	0.00065353	21
Rap1a3	3.35E-08	0.3103936	0.449	0.19	0.00108158	21
Ndufa116	4.12E-08	0.85053655	0.64	0.36	0.00132981	21
Sars	4.37E-08	0.4026069	0.337	0.127	0.00140984	21
Rpn14	5.30E-08	0.58229002	0.416	0.175	0.001712	21
Ost49	1.46E-07	0.44210182	0.64	0.351	0.00470618	21
Mt23	2.43E-07	0.38293272	0.326	0.128	0.00785059	21
Ptp4a22	2.75E-07	0.30680611	0.461	0.205	0.00886387	21
Tmbim44	3.45E-07	0.31895533	0.337	0.138	0.01115424	21
Ndufb63	9.04E-07	0.33648472	0.348	0.146	0.02918427	21
Kdelr22	9.12E-07	0.45485568	0.326	0.137	0.02943354	21
Tmed34	1.04E-06	0.33551255	0.27	0.103	0.03366237	21
4-Jun	1.84E-06	0.51536281	0.382	0.18	0.05947256	21
Rps27l8	2.97E-06	0.47768849	0.652	0.349	0.09584824	21
Dap2	2.09E-05	0.32321309	0.348	0.164	0.6741565	21
Uqcrq5	3.33E-05	0.3659161	0.708	0.412	1	21
Gm1007612	3.43E-05	0.36421657	0.933	0.811	1	21
Psmb103	4.92E-05	0.27483235	0.281	0.125	1	21
Tmem2589	6.99E-05	0.35071132	0.64	0.388	1	21
Fos8	0.00016703	0.32399958	0.573	0.363	1	21
Sec61g8	0.00062052	0.37631994	0.787	0.613	1	21
Sub110	0.00238747	0.41241589	0.708	0.482	1	21
Ndufa49	0.00360917	0.38812923	0.685	0.467	1	21
Rpl54	0.00428354	0.27767552	0.73	0.462	1	21
Hspa55	0.00507238	0.33236313	0.562	0.359	1	21
Cxcl12	0	7.67138695	0.986	0.004	0	22

Ibsp	0	6.05114632	0.859	0.001	0	22
Igfbp5	0	5.97777794	0.845	0.005	0	22
Cxcl14	0	5.01275914	0.887	0.01	0	22
Serpine21	0	4.88455979	0.873	0.022	0	22
Mgp1	0	4.55729936	0.775	0.018	0	22
Lepr	0	4.31143576	0.873	0.002	0	22
Lpl	0	4.3061809	0.845	0.013	0	22
Gpx31	0	4.24376756	0.789	0.019	0	22
Gas6	0	4.20546313	0.901	0.007	0	22
Adipoq	0	4.12577212	0.746	0	0	22
Serping11	0	4.02910107	0.845	0.017	0	22
Gdpd2	0	3.82610901	0.732	0	0	22
Vcam1	0	3.65771944	0.732	0.021	0	22
Tnc1	0	3.61515527	0.803	0.017	0	22
Fbln5	0	3.56449891	0.62	0.001	0	22
Plpp3	0	3.48366696	0.634	0.016	0	22
Bgn1	0	3.22112298	0.845	0.031	0	22
Fstl11	0	3.14967628	0.789	0.025	0	22
Ccl19	0	3.03922811	0.521	0	0	22
Pappa	0	3.0277843	0.592	0.002	0	22
Cyp1b1	0	2.99429696	0.606	0.003	0	22
Cdh111	0	2.98675687	0.577	0.008	0	22
Chrdl1	0	2.97305523	0.535	0	0	22
Ebf3	0	2.94906479	0.577	0	0	22
Rarres2	0	2.85188597	0.507	0.005	0	22
Pdgfrb	0	2.805745	0.563	0.007	0	22
C4b	0	2.79506615	0.493	0.007	0	22
Ogn	0	2.7931417	0.493	0.006	0	22
Ptprd	0	2.73028562	0.479	0.001	0	22
Esm1	0	2.70377614	0.493	0.001	0	22
Ptx3	0	2.70134666	0.394	0.001	0	22
Sfrp4	0	2.65200104	0.38	0.002	0	22
Epas1	0	2.59243209	0.577	0.002	0	22
Agt	0	2.54038786	0.451	0.001	0	22
Abi3bp	0	2.45882427	0.479	0.007	0	22
Islr	0	2.43354539	0.437	0.005	0	22
Tgfbr3	0	2.35702558	0.465	0.006	0	22
Serpina12	0	2.26428941	0.352	0	0	22
1500009L16F	0	2.25161201	0.38	0.004	0	22
Mdk	0	2.23385774	0.366	0.005	0	22
Fxyd1	0	2.18435681	0.408	0.005	0	22
Slc26a7	0	2.15466946	0.31	0	0	22
Ackr4	0	2.15008149	0.324	0	0	22

Fmo2	0	2.1435272	0.338	0.001	0	22
Foxc1	0	2.0945217	0.338	0.002	0	22
Kng1	0	2.06220393	0.31	0	0	22
Dpep1	0	2.03559571	0.338	0.001	0	22
Kng2	0	1.98830811	0.352	0.001	0	22
Cldn10	0	1.98195965	0.366	0.005	0	22
Mme	0	1.97935509	0.324	0.001	0	22
Fgfr2	0	1.92007516	0.296	0.001	0	22
Snai2	0	1.80929862	0.282	0.002	0	22
Dlc1	0	1.79851362	0.296	0.002	0	22
Bmp6	0	1.79101261	0.31	0.001	0	22
C1ra	0	1.77113561	0.352	0.004	0	22
Vit	0	1.75591808	0.282	0.002	0	22
Il34	0	1.69188108	0.254	0.002	0	22
Vegfc	0	1.69007292	0.254	0.002	0	22
Ar	0	1.66980462	0.254	0.002	0	22
Fgf7	0	1.63484673	0.254	0.001	0	22
Ccn5	0	1.57165692	0.254	0.002	0	22
S1pr3	0	1.53685357	0.268	0.002	0	22
Kitl	1.85E-298	3.7105215	0.761	0.027	5.96E-294	22
Antxr1	1.42E-287	1.76676611	0.268	0.003	4.58E-283	22
Plxdc2	9.23E-284	2.15211212	0.394	0.007	2.98E-279	22
Serpina3n	7.29E-283	1.64207502	0.254	0.003	2.35E-278	22
Cfh	3.46E-277	3.00915495	0.62	0.019	1.12E-272	22
Pdgfra	6.24E-275	1.90914919	0.352	0.006	2.01E-270	22
Serpina3g	3.33E-272	2.08672499	0.408	0.008	1.07E-267	22
Cxcl1	3.13E-264	3.14909202	0.296	0.004	1.01E-259	22
Mmp13	1.31E-252	2.6032681	0.296	0.004	4.23E-248	22
Adamts2	5.77E-243	2.09063369	0.352	0.007	1.86E-238	22
Igsf3	1.98E-236	2.03062347	0.352	0.007	6.39E-232	22
Colec12	1.36E-234	1.82908075	0.282	0.004	4.40E-230	22
Spry1	3.21E-234	1.7104373	0.296	0.005	1.04E-229	22
Cd3021	4.18E-227	3.0270188	0.704	0.031	1.35E-222	22
Lifr	6.75E-226	2.18662968	0.38	0.009	2.18E-221	22
Gja1	1.14E-223	2.92981314	0.62	0.024	3.67E-219	22
Col1a21	1.47E-196	1.56799315	0.732	0.036	4.74E-192	22
C1s1	1.15E-194	2.01525347	0.366	0.009	3.70E-190	22
Adamts1	1.05E-183	2.9313394	0.352	0.009	3.40E-179	22
Cped1	1.70E-181	2.0178874	0.31	0.007	5.49E-177	22
Ccn1	9.40E-166	3.31698747	0.465	0.018	3.03E-161	22
Csf11	1.25E-158	2.25269466	0.507	0.022	4.03E-154	22
Prelp	4.08E-158	1.80016892	0.268	0.006	1.32E-153	22
St5	8.35E-156	1.73684417	0.268	0.006	2.69E-151	22

Mylk	3.09E-155	1.54006187	0.268	0.006	9.99E-151	22
Fst	7.04E-153	1.94310182	0.338	0.01	2.27E-148	22
Loxl11	4.81E-147	1.62626102	0.338	0.01	1.55E-142	22
Gm4951	1.42E-142	1.57712332	0.268	0.007	4.60E-138	22
Bicc1	6.71E-136	1.52135333	0.31	0.01	2.17E-131	22
Tmem176a2	1.20E-132	3.40972791	0.817	0.075	3.87E-128	22
Igfbp71	2.67E-132	3.4083772	0.817	0.073	8.61E-128	22
Steap4	1.76E-131	1.47728836	0.254	0.007	5.67E-127	22
Cp1	1.96E-131	2.25037973	0.563	0.033	6.33E-127	22
Grem1	2.20E-130	1.80341108	0.296	0.009	7.10E-126	22
Cpq	4.00E-126	2.2427418	0.437	0.021	1.29E-121	22
Gm12216	4.92E-126	1.83936616	0.254	0.007	1.59E-121	22
Apoe2	1.09E-123	2.79286121	0.986	0.114	3.53E-119	22
Zfhx4	8.83E-123	1.82889726	0.254	0.007	2.85E-118	22
Arrdc4	7.01E-114	2.44456414	0.38	0.018	2.26E-109	22
Tmem176b3	4.39E-113	3.801901	0.915	0.118	1.42E-108	22
Lox1	6.29E-110	1.7177109	0.324	0.013	2.03E-105	22
Fcgrt	1.41E-97	1.59885938	0.31	0.014	4.54E-93	22
Il1r1	1.67E-96	1.48382481	0.268	0.01	5.40E-92	22
Nupr11	1.92E-92	2.90977188	0.634	0.06	6.19E-88	22
Lhfp	5.62E-90	1.82546572	0.366	0.02	1.81E-85	22
Sparc1	1.41E-83	1.77827892	0.789	0.098	4.55E-79	22
Id32	7.37E-83	3.43177285	0.718	0.09	2.38E-78	22
Errfi1	4.05E-81	2.9310008	0.521	0.046	1.31E-76	22
Dcn1	1.57E-79	1.36505255	0.352	0.021	5.08E-75	22
Pam	2.12E-79	2.71590677	0.606	0.065	6.84E-75	22
Selenop1	2.82E-79	2.33677548	0.606	0.063	9.09E-75	22
Serpinh11	1.01E-78	2.06145627	0.577	0.056	3.26E-74	22
Col8a1	7.50E-78	1.72677496	0.268	0.013	2.42E-73	22
Ghr	2.25E-76	1.6937042	0.324	0.019	7.26E-72	22
Rcn31	9.42E-76	1.5737479	0.31	0.017	3.04E-71	22
Fat1	3.57E-74	1.64566017	0.268	0.013	1.15E-69	22
Maf	1.56E-73	1.88040217	0.408	0.03	5.03E-69	22
Ccn2	1.96E-73	2.32743532	0.296	0.016	6.34E-69	22
Gng11	2.51E-72	1.70490055	0.296	0.017	8.10E-68	22
Trf1	4.15E-70	3.10861324	0.676	0.093	1.34E-65	22
Fbn11	6.87E-70	1.83892063	0.324	0.02	2.22E-65	22
Angpt11	1.31E-69	1.77895716	0.254	0.013	4.23E-65	22
Amotl2	2.82E-68	1.49815949	0.254	0.013	9.11E-64	22
Igfbp43	1.27E-67	3.49183645	0.859	0.174	4.09E-63	22
Socs3	4.72E-63	2.9758363	0.437	0.042	1.52E-58	22
Slfn5	7.20E-62	1.65614133	0.31	0.021	2.32E-57	22
Olfml3	3.99E-60	1.47468682	0.254	0.015	1.29E-55	22

Cd1d1	7.74E-60	1.40865461	0.268	0.016	2.50E-55	22
Thra	1.08E-58	1.54907063	0.31	0.022	3.49E-54	22
Emp2	3.64E-58	1.73410278	0.324	0.025	1.18E-53	22
Col4a21	6.99E-58	1.64456723	0.394	0.035	2.26E-53	22
Apbb2	5.46E-57	1.66251694	0.296	0.021	1.76E-52	22
Ecm1	1.74E-55	1.63241159	0.423	0.042	5.63E-51	22
Gpm6b	3.60E-54	1.66575292	0.352	0.03	1.16E-49	22
Rnd31	5.69E-54	1.92788291	0.31	0.024	1.84E-49	22
Tns3	1.94E-53	1.68607042	0.338	0.029	6.27E-49	22
Camk2n1	2.27E-51	1.59873037	0.31	0.025	7.34E-47	22
Nfia	4.81E-51	1.88057875	0.408	0.043	1.55E-46	22
Tpm13	1.29E-50	2.47649539	0.831	0.185	4.17E-46	22
Fermt2	3.26E-50	1.16979371	0.268	0.019	1.05E-45	22
Cdh2	8.78E-50	1.74946278	0.324	0.028	2.83E-45	22
Lgals3bp	1.22E-49	1.60606109	0.31	0.026	3.95E-45	22
Slc5a3	1.63E-49	1.45869046	0.282	0.022	5.26E-45	22
Iigp1	3.39E-49	2.02291567	0.38	0.038	1.10E-44	22
Selenom3	5.64E-48	1.7472349	0.437	0.052	1.82E-43	22
Insig2	2.15E-46	1.56127506	0.282	0.023	6.93E-42	22
Zcchc14	3.34E-46	1.2261581	0.296	0.025	1.08E-41	22
Fkbp7	1.53E-44	1.38513053	0.254	0.019	4.95E-40	22
Ifi211	1.51E-43	1.4336639	0.282	0.025	4.87E-39	22
Lrp12	3.84E-42	1.94310555	0.451	0.062	1.24E-37	22
Nenf1	8.92E-42	1.99509351	0.465	0.068	2.88E-37	22
Gpx8	3.07E-41	1.55875373	0.352	0.039	9.91E-37	22
Aplp2	8.74E-41	1.76750329	0.465	0.068	2.82E-36	22
Col6a11	2.68E-40	1.74280957	0.394	0.049	8.65E-36	22
Runx13	3.91E-40	2.33869377	0.606	0.122	1.26E-35	22
Sdc11	2.13E-39	1.67966309	0.338	0.038	6.88E-35	22
Rbpms	6.22E-39	1.80294624	0.324	0.036	2.01E-34	22
Zfp36l14	1.82E-38	2.34937782	0.62	0.13	5.88E-34	22
Cst35	4.60E-38	2.81173891	0.944	0.476	1.49E-33	22
Egr16	5.06E-38	3.48356809	0.69	0.18	1.63E-33	22
Nrp1	2.22E-37	1.4211234	0.324	0.036	7.17E-33	22
Hp7	1.17E-36	3.1439902	0.845	0.308	3.78E-32	22
Slc29a1	1.30E-36	1.64859046	0.408	0.058	4.18E-32	22
Fosb7	3.46E-35	3.65378968	0.606	0.147	1.12E-30	22
Lbp1	5.51E-35	1.67124754	0.324	0.04	1.78E-30	22
Hes12	9.21E-35	2.23124457	0.465	0.078	2.97E-30	22
Klf9	9.51E-33	1.73391631	0.366	0.052	3.07E-28	22
Col4a11	1.34E-32	1.38202998	0.437	0.069	4.32E-28	22
Clec2d2	5.70E-32	1.88824666	0.493	0.096	1.84E-27	22
H2-Q10	1.67E-31	1.23368297	0.268	0.029	5.39E-27	22

Ifitm36	1.58E-30	1.82711382	0.944	0.467	5.09E-26	22
Clu2	2.00E-30	1.23244271	0.648	0.146	6.45E-26	22
Ifnar22	2.33E-30	1.93878276	0.507	0.109	7.52E-26	22
Crtap	5.16E-30	1.23019775	0.254	0.028	1.67E-25	22
Col6a21	7.67E-30	1.50595786	0.268	0.031	2.48E-25	22
Laptm4a2	2.36E-29	1.82816774	0.606	0.153	7.61E-25	22
H2-K18	2.24E-28	2.01617762	0.887	0.46	7.23E-24	22
Marcks7	3.78E-28	1.87369817	0.69	0.199	1.22E-23	22
Mt15	1.28E-26	3.43187573	0.704	0.243	4.12E-22	22
Runx21	1.57E-26	1.46161294	0.282	0.038	5.06E-22	22
Vkorc13	9.45E-26	1.50513946	0.338	0.056	3.05E-21	22
App2	5.58E-25	1.68632208	0.592	0.165	1.80E-20	22
Ak3	1.32E-24	1.14657787	0.268	0.037	4.27E-20	22
Oat	7.67E-24	1.74320358	0.31	0.052	2.48E-19	22
Mt24	7.85E-24	2.67972473	0.507	0.127	2.54E-19	22
Gadd45g2	9.35E-24	2.6497978	0.408	0.088	3.02E-19	22
Gadd45b2	2.30E-23	2.15685458	0.423	0.093	7.41E-19	22
Ccl21	3.31E-23	3.22024535	0.268	0.039	1.07E-18	22
Vegfa	3.55E-23	1.69013416	0.324	0.057	1.15E-18	22
Tcf7l2	4.30E-23	1.22576844	0.296	0.046	1.39E-18	22
Lamb1	7.34E-23	1.30660554	0.254	0.035	2.37E-18	22
Malat16	7.88E-23	2.1272287	0.986	0.958	2.54E-18	22
Zbtb202	8.20E-23	1.33021969	0.38	0.074	2.65E-18	22
Lrpap11	2.29E-22	1.19374267	0.282	0.045	7.39E-18	22
Il1rn1	6.72E-22	1.95580054	0.324	0.06	2.17E-17	22
Irf12	8.72E-22	2.01418775	0.366	0.075	2.82E-17	22
Ctsl5	1.37E-21	1.71424158	0.648	0.211	4.43E-17	22
Fndc3b	1.48E-21	1.51977349	0.324	0.059	4.78E-17	22
Tmem592	1.52E-21	1.71588865	0.535	0.155	4.90E-17	22
Pcolce2	1.64E-21	1.75994881	0.38	0.078	5.29E-17	22
Nptn1	3.24E-21	1.34447821	0.437	0.103	1.05E-16	22
Glul1	4.70E-21	2.0029728	0.338	0.068	1.52E-16	22
Slc39a1	5.30E-20	1.46153748	0.366	0.079	1.71E-15	22
H2-D17	6.56E-20	1.58857842	0.887	0.662	2.12E-15	22
Pik3r1	6.84E-20	1.66046083	0.324	0.064	2.21E-15	22
Sgk1	1.23E-19	2.04552501	0.31	0.06	3.98E-15	22
Nedd44	1.45E-19	1.40372029	0.662	0.225	4.67E-15	22
Lamp14	1.82E-19	1.66857442	0.634	0.237	5.88E-15	22
5-Jun	2.45E-19	3.10147102	0.535	0.18	7.92E-15	22
Nfib	2.54E-19	1.17702686	0.296	0.053	8.21E-15	22
Cd812	3.41E-19	1.5115826	0.549	0.172	1.10E-14	22
Tuba1a	5.31E-19	1.34630377	0.338	0.071	1.71E-14	22
St3gal6	6.87E-19	1.14698292	0.282	0.05	2.22E-14	22

Mxd4	4.70E-18	1.37722928	0.282	0.053	1.52E-13	22
Ebf15	6.74E-18	0.34371141	0.746	0.222	2.18E-13	22
Hspa56	1.00E-17	1.49349094	0.761	0.358	3.23E-13	22
H2-Q74	3.99E-17	1.17375754	0.394	0.097	1.29E-12	22
mt-Nd4l9	1.57E-16	1.46016026	0.831	0.451	5.07E-12	22
Tent5a2	1.73E-16	1.15232111	0.254	0.047	5.58E-12	22
Itm2b7	2.15E-16	1.30526597	0.859	0.538	6.95E-12	22
Mfge8	4.32E-16	1.05945993	0.254	0.047	1.40E-11	22
Zeb23	5.71E-16	1.46332503	0.465	0.143	1.84E-11	22
Timp11	7.31E-16	1.47017635	0.268	0.053	2.36E-11	22
Itgb14	1.11E-15	1.31252839	0.606	0.228	3.59E-11	22
Gns	1.28E-15	1.16392015	0.254	0.049	4.14E-11	22
Spp14	2.47E-15	1.00282289	0.704	0.269	7.99E-11	22
Nudt44	3.32E-15	1.3900291	0.521	0.187	1.07E-10	22
Dnajc32	1.74E-14	1.37040544	0.352	0.095	5.60E-10	22
Saraf2	2.11E-14	1.08192662	0.31	0.074	6.82E-10	22
Tm9sf2	3.48E-14	1.30123414	0.338	0.089	1.12E-09	22
Itm2c1	3.52E-14	1.0491995	0.268	0.058	1.14E-09	22
Cd636	1.06E-13	1.4694363	0.606	0.274	3.41E-09	22
Lrrc58	1.22E-13	1.23038621	0.324	0.085	3.95E-09	22
Fos9	1.25E-13	3.03476409	0.634	0.363	4.05E-09	22
Gstp18	2.55E-13	1.33652596	0.437	0.148	8.23E-09	22
Tapbp2	6.13E-13	1.3129641	0.394	0.125	1.98E-08	22
Rbpj	1.19E-12	1.13406092	0.408	0.129	3.85E-08	22
Atraid1	1.30E-12	1.2124127	0.254	0.059	4.19E-08	22
Cyb5a3	6.89E-12	1.14802688	0.451	0.166	2.22E-07	22
Sept112	1.43E-11	1.069794	0.282	0.074	4.60E-07	22
Cald12	1.61E-11	1.03345262	0.352	0.104	5.18E-07	22
Rabac14	1.65E-11	1.15044645	0.507	0.209	5.34E-07	22
Nid11	1.93E-11	0.84330045	0.268	0.065	6.25E-07	22
Selenos6	1.99E-11	1.19354961	0.38	0.127	6.41E-07	22
Ppib7	4.01E-11	1.26555557	0.634	0.338	1.30E-06	22
Ctsb6	4.65E-11	1.00340139	0.563	0.241	1.50E-06	22
Cebpd4	6.34E-11	2.39587256	0.366	0.135	2.05E-06	22
mt-Cytb6	9.23E-11	0.95455767	0.915	0.666	2.98E-06	22
Zfp367	9.35E-11	2.00173398	0.507	0.231	3.02E-06	22
Map1lc3a	1.12E-10	1.18937441	0.296	0.087	3.63E-06	22
Nfe2l1	1.15E-10	1.20672551	0.296	0.086	3.72E-06	22
Wsb11	1.19E-10	1.47380068	0.338	0.114	3.83E-06	22
Pdia36	1.21E-10	1.07951079	0.563	0.259	3.89E-06	22
Slc6a6	2.67E-10	1.38969683	0.366	0.129	8.63E-06	22
Rgcc6	6.16E-10	1.68273385	0.352	0.13	1.99E-05	22
Selenof3	1.53E-09	1.05029254	0.535	0.251	4.95E-05	22

Map4k4	1.84E-09	1.03859143	0.31	0.101	5.94E-05	22
Ier35	2.01E-09	1.5552348	0.38	0.149	6.48E-05	22
Ftl15	2.26E-09	1.01734644	0.901	0.809	7.31E-05	22
B2m5	2.34E-09	0.76451334	0.901	0.712	7.56E-05	22
Kctd125	2.59E-09	1.12163022	0.451	0.191	8.36E-05	22
Ppp1r15a4	2.99E-09	1.04182472	0.338	0.117	9.66E-05	22
Arl1	4.36E-09	1.07040164	0.254	0.074	0.00014082	22
Atp1a12	5.33E-09	1.10873022	0.338	0.121	0.00017195	22
Rhob	5.81E-09	1.16859841	0.324	0.114	0.00018759	22
Hsp90b18	7.63E-09	1.03278118	0.662	0.381	0.0002464	22
Rbms12	8.03E-09	0.9726296	0.479	0.211	0.00025926	22
Hspb11	9.67E-09	0.79483848	0.282	0.085	0.00031235	22
Smad73	1.81E-08	0.91439866	0.268	0.084	0.00058377	22
Sh3bgrl4	2.65E-08	1.0859433	0.423	0.184	0.000856	22
Dnajb92	2.69E-08	1.15048842	0.254	0.08	0.0008686	22
Emp12	2.88E-08	0.80651189	0.324	0.107	0.0009311	22
Alkbh51	4.08E-08	1.05933147	0.296	0.105	0.00131754	22
Soat11	7.74E-08	1.06771964	0.296	0.107	0.0024991	22
Kdelr23	9.39E-08	1.21158287	0.338	0.137	0.00303253	22
Brd22	1.16E-07	0.92377948	0.352	0.141	0.00373009	22
Cdkn1a2	1.35E-07	1.28471393	0.268	0.095	0.00435416	22
Ier51	1.35E-07	1.62962036	0.254	0.086	0.00435874	22
Pdia44	1.35E-07	1.01847406	0.31	0.116	0.00436971	22
Xbp14	1.51E-07	1.18532258	0.31	0.119	0.00488872	22
Tnfrsf1a3	2.24E-07	1.0534389	0.296	0.112	0.00722592	22
Zfand52	2.71E-07	1.1556003	0.408	0.196	0.00873612	22
Eid1	3.45E-07	0.84148444	0.296	0.109	0.01112545	22
Ostc4	3.63E-07	1.12171114	0.394	0.182	0.01172845	22
Samhd14	4.03E-07	1.02323655	0.394	0.179	0.01300331	22
Notch27	5.65E-07	1.21948901	0.423	0.212	0.01823402	22
Ergic31	5.70E-07	0.94177353	0.254	0.089	0.01838896	22
Emc72	6.67E-07	1.04892905	0.282	0.107	0.02152626	22
Lmo48	7.40E-07	0.79728568	0.493	0.262	0.02389233	22
Cstb2	8.20E-07	0.81565128	0.451	0.221	0.02646079	22
Manf6	8.45E-07	0.91526152	0.437	0.21	0.02729136	22
Tacc15	1.05E-06	1.21894744	0.352	0.157	0.03383654	22
Npc26	1.09E-06	0.85454329	0.493	0.252	0.03521065	22
P4hb4	1.45E-06	0.82298039	0.394	0.183	0.0467867	22
Ifngr14	1.71E-06	0.87410465	0.296	0.119	0.05533035	22
Son4	1.92E-06	0.85379174	0.592	0.388	0.06182707	22
Bst23	1.93E-06	0.80378303	0.352	0.15	0.06216671	22
Gsk3b	2.10E-06	0.94076116	0.352	0.159	0.06765642	22
Pdia64	2.52E-06	0.86147112	0.352	0.158	0.0813558	22

Spcs14	2.64E-06	0.89278528	0.408	0.207	0.08514047	22
Srpr1	3.50E-06	0.88256612	0.254	0.095	0.11295914	22
Ptms5	3.97E-06	0.86605209	0.38	0.174	0.12831153	22
Pdcd48	4.50E-06	0.65674401	0.479	0.245	0.14516147	22
Hk22	4.72E-06	1.84181257	0.254	0.103	0.15231281	22
Ddx59	5.69E-06	0.8355775	0.69	0.551	0.18382429	22
Tmco16	7.22E-06	0.97957608	0.338	0.16	0.23296953	22
Cd97	9.44E-06	0.76716326	0.563	0.343	0.30486688	22
Calu2	1.10E-05	0.84770826	0.31	0.134	0.35524774	22
Sec623	1.12E-05	0.83490184	0.507	0.299	0.36137019	22
Ubc5	1.18E-05	1.24501833	0.577	0.42	0.37948032	22
Rpn21	1.18E-05	0.91550084	0.296	0.13	0.38178116	22
Atxn7l3b2	1.38E-05	0.76577011	0.296	0.13	0.44398524	22
C34	1.61E-05	1.18561448	0.31	0.148	0.51848228	22
Nfkbia8	1.76E-05	1.07256141	0.437	0.242	0.56910973	22
Ifi271	2.28E-05	0.87428163	0.31	0.143	0.73665312	22
Eif1b3	2.29E-05	1.01494782	0.282	0.13	0.7404315	22
Cited24	2.32E-05	1.21758122	0.268	0.119	0.74850586	22
Calr6	2.37E-05	0.56555273	0.465	0.246	0.76547946	22
Nfkbiz5	2.65E-05	0.94549401	0.296	0.136	0.8566158	22
Ahnak5	2.91E-05	0.43886978	0.549	0.28	0.93943751	22
Slc38a26	3.01E-05	1.06611866	0.563	0.374	0.97138289	22
Reep3	3.68E-05	0.73573563	0.254	0.107	1	22
Junb8	3.73E-05	1.00816746	0.606	0.437	1	22
Klhl243	4.32E-05	0.81926828	0.254	0.107	1	22
Cops94	6.65E-05	1.06767382	0.437	0.277	1	22
Selenok8	8.03E-05	0.7170686	0.577	0.396	1	22
Rbm394	8.25E-05	0.69498815	0.606	0.409	1	22
Wls1	9.52E-05	0.69026042	0.254	0.11	1	22
Ndufb27	9.89E-05	0.82273863	0.394	0.227	1	22
Lamp25	0.00015113	0.91257897	0.38	0.223	1	22
Lmna3	0.00015135	1.04061031	0.338	0.172	1	22
Ubb11	0.00017753	0.73346845	0.873	0.762	1	22
Selenow9	0.00018088	0.92179751	0.437	0.267	1	22
Csnk1a15	0.00018954	0.67750394	0.423	0.245	1	22
Sod24	0.00019664	0.93586065	0.254	0.122	1	22
Ifrd14	0.00021164	1.47401098	0.31	0.165	1	22
Morf4l16	0.00025037	0.95067285	0.535	0.371	1	22
Pten3	0.00027623	0.73164726	0.352	0.196	1	22
Dad110	0.00029513	0.54357176	0.423	0.245	1	22
mt-Atp88	0.00029605	0.77967863	0.465	0.279	1	22
Dpm39	0.00033272	0.87364595	0.423	0.257	1	22
Hdlbp2	0.00033856	0.99692064	0.282	0.146	1	22

Ifitm27	0.00034622	0.32937921	0.662	0.458	1	22
Scp26	0.00037836	0.66375304	0.38	0.226	1	22
Romo14	0.00040269	0.63126879	0.521	0.352	1	22
Tceal94	0.00041753	0.64683714	0.296	0.151	1	22
Irf2bp23	0.00046472	0.62600795	0.338	0.185	1	22
Fus5	0.00047833	0.69322835	0.423	0.257	1	22
Txnip	0.00050904	0.64590869	0.254	0.122	1	22
Ssr49	0.00063773	0.49369092	0.423	0.253	1	22
Timp24	0.00070691	0.52979797	0.324	0.174	1	22
Cebpb5	0.00073149	0.27707494	0.577	0.394	1	22
Mgst16	0.0012398	0.69548216	0.38	0.237	1	22
mt-Nd46	0.00127913	0.47337549	0.662	0.492	1	22
Sod11	0.00137328	0.68838538	0.282	0.153	1	22
Tsc22d38	0.00143857	0.70009755	0.31	0.179	1	22
Eif17	0.00167645	0.2794198	0.873	0.839	1	22
Pafah1b14	0.00193739	0.83519356	0.394	0.262	1	22
Prdx18	0.00198378	0.54538398	0.592	0.397	1	22
Klf64	0.00206305	1.1390574	0.366	0.238	1	22
Zfp36l26	0.00208858	0.66134856	0.408	0.263	1	22
Slbp4	0.00219815	0.69703032	0.268	0.146	1	22
Rpn15	0.0022369	0.85448651	0.296	0.175	1	22
Krtcap210	0.00228774	0.67207355	0.394	0.257	1	22
Gpx46	0.0023887	0.59856482	0.535	0.401	1	22
Rock21	0.00246174	0.53793717	0.282	0.156	1	22
Grn3	0.00252577	0.79904163	0.268	0.152	1	22
Rex1bd3	0.00257183	0.64951878	0.254	0.139	1	22
Nr4a17	0.00278793	0.57003519	0.338	0.205	1	22
Snhg96	0.0029114	0.84816304	0.254	0.141	1	22
Bsg4	0.00309431	0.47936461	0.394	0.256	1	22
Ddx3x2	0.00342023	0.4365072	0.366	0.229	1	22
Ddx17	0.00362831	0.70804898	0.268	0.153	1	22
Hsbp13	0.00414798	0.62066973	0.324	0.203	1	22
Srrm25	0.00457919	0.4540341	0.465	0.322	1	22
Dleu23	0.00491316	0.47730043	0.254	0.14	1	22
Hspa84	0.00523168	0.4100941	0.676	0.552	1	22
Rer16	0.00551788	0.64701515	0.254	0.147	1	22
Kmt2e1	0.00566689	0.50100189	0.268	0.152	1	22
Picalm5	0.00599814	0.50922939	0.352	0.226	1	22
Rab14	0.0062139	0.74932787	0.296	0.184	1	22
Ost410	0.00665083	0.49855446	0.479	0.351	1	22
Ccnl17	0.00691931	0.76738254	0.366	0.251	1	22
Pnrc19	0.00704268	0.69963026	0.437	0.319	1	22
Tmem50a3	0.00725158	0.39773641	0.31	0.19	1	22

Ndufb109	0.00740369	0.56924331	0.366	0.246	1	22
Ssr32	0.00792775	0.43684883	0.31	0.19	1	22
Sept7	0.00835588	0.5908838	0.338	0.224	1	22
H2-T235	0.00845163	0.59190829	0.282	0.176	1	22
Dbi6	0.00977634	0.48819265	0.437	0.312	1	22
Fabp4	0	5.53326901	0.704	0.004	0	23
Plvap	0	4.05072075	0.685	0.002	0	23
Cdh5	0	3.91900734	0.722	0.001	0	23
Flt1	0	3.47384078	0.574	0.004	0	23
Eng	0	3.39601874	0.574	0.01	0	23
Adgrf5	0	3.10626139	0.5	0.001	0	23
Kdr	0	3.0752689	0.5	0.001	0	23
Esam	0	3.03202833	0.444	0.001	0	23
Pcdh17	0	3.00800399	0.481	0.001	0	23
Aplnr	0	2.75828737	0.407	0.001	0	23
Gpihbp1	0	2.69354307	0.352	0	0	23
Ramp2	0	2.57489032	0.352	0.002	0	23
Ptprb	0	2.53491557	0.352	0.001	0	23
Emcn	0	2.49975288	0.37	0.001	0	23
Ecscr	0	2.2558807	0.352	0.004	0	23
Mmrn2	0	2.17292921	0.315	0.001	0	23
Sox18	0	1.98684633	0.259	0	0	23
Fzd4	5.30E-269	1.72748658	0.259	0.002	1.71E-264	23
Fam167b	3.87E-267	2.20348804	0.278	0.003	1.25E-262	23
Gng111	5.96E-234	3.71379025	0.593	0.016	1.92E-229	23
Sparcl1	8.83E-219	2.96945201	0.389	0.007	2.85E-214	23
Rasip1	1.21E-199	2.42562071	0.278	0.004	3.92E-195	23
Col4a22	3.07E-187	4.1157886	0.759	0.035	9.92E-183	23
Mcam	3.09E-183	2.6421706	0.333	0.006	9.97E-179	23
Arhgap29	7.33E-175	2.03526883	0.259	0.004	2.37E-170	23
Egfl7	3.02E-147	2.69640611	0.463	0.016	9.75E-143	23
Cd2001	2.60E-145	2.99122569	0.426	0.013	8.38E-141	23
Igfbp72	8.83E-130	4.07624527	0.907	0.073	2.85E-125	23
Ctla2a2	7.04E-126	3.51762945	0.648	0.036	2.27E-121	23
Adamts11	5.51E-112	2.53044632	0.315	0.009	1.78E-107	23
Cxcl121	4.62E-109	2.04352368	0.259	0.006	1.49E-104	23
Cd341	1.97E-108	2.8780619	0.426	0.018	6.37E-104	23
Sparc2	3.86E-104	3.22557374	0.944	0.098	1.25E-99	23
Col4a12	1.01E-103	4.34765965	0.778	0.069	3.26E-99	23
Myct11	5.20E-96	2.02987689	0.278	0.009	1.68E-91	23
Myo1b	9.67E-87	2.12315137	0.315	0.012	3.12E-82	23
Adgrl41	1.09E-84	2.14624675	0.296	0.011	3.53E-80	23
Col15a11	2.33E-84	2.0952809	0.333	0.014	7.51E-80	23

Pecam1	3.34E-84	2.91446165	0.463	0.028	1.08E-79	23
Hspg2	1.76E-78	2.73413373	0.481	0.032	5.70E-74	23
Plxnd1	2.42E-73	2.74886124	0.389	0.023	7.80E-69	23
Stab1	1.03E-70	2.04832448	0.259	0.01	3.34E-66	23
Cd931	4.61E-70	3.22748126	0.63	0.064	1.49E-65	23
Crim1	6.28E-63	2.23073209	0.352	0.021	2.03E-58	23
S1pr11	1.16E-54	2.32437915	0.481	0.044	3.74E-50	23
Rbp11	1.79E-53	2.57748767	0.426	0.036	5.78E-49	23
Serpinh12	2.08E-53	2.55143506	0.537	0.056	6.72E-49	23
Tcf42	4.01E-52	2.93530494	0.704	0.106	1.30E-47	23
Ece1	3.45E-47	2.20462587	0.315	0.022	1.12E-42	23
Igfbp3	1.87E-46	3.50944892	0.463	0.05	6.05E-42	23
Gja11	8.03E-42	2.00939262	0.315	0.025	2.59E-37	23
Timp31	2.72E-39	2.51388592	0.296	0.024	8.78E-35	23
Fermt21	2.01E-37	1.87658158	0.259	0.019	6.49E-33	23
Cav1	3.20E-34	2.28486149	0.315	0.03	1.03E-29	23
Icam2	5.32E-34	2.02702671	0.278	0.024	1.72E-29	23
Kitl1	1.43E-31	2.1374213	0.296	0.029	4.61E-27	23
Ppic1	1.65E-27	2.16850388	0.278	0.03	5.32E-23	23
Fkbp1a3	1.27E-22	2.29775373	0.667	0.206	4.10E-18	23
Insr	1.36E-21	1.72469453	0.296	0.041	4.40E-17	23
Xist7	3.37E-21	2.2278246	0.537	0.133	1.09E-16	23
Cavin3	2.22E-20	1.80038744	0.278	0.039	7.17E-16	23
Lgmn1	3.04E-20	1.72973525	0.333	0.053	9.81E-16	23
Pls3	4.02E-20	1.70530748	0.315	0.048	1.30E-15	23
Iigp11	7.14E-20	1.74690842	0.278	0.039	2.30E-15	23
Itgb15	7.27E-20	2.11680891	0.667	0.228	2.35E-15	23
Trp53i11	6.56E-19	1.89456553	0.259	0.036	2.12E-14	23
Mfge81	3.38E-18	1.70894185	0.296	0.047	1.09E-13	23
Gm424184	6.49E-17	1.60221488	1	0.941	2.10E-12	23
Pdlim1	3.72E-16	2.06753027	0.278	0.048	1.20E-11	23
Aplp21	4.00E-16	2.03053885	0.333	0.068	1.29E-11	23
4931406P16I	4.15E-16	1.90967846	0.278	0.048	1.34E-11	23
Nfib1	6.98E-16	1.62277924	0.296	0.053	2.25E-11	23
Ehd4	6.05E-15	1.93820532	0.296	0.058	1.95E-10	23
Elk3	3.22E-14	1.55154618	0.296	0.059	1.04E-09	23
Clic4	3.27E-14	1.71622445	0.37	0.089	1.06E-09	23
Col18a11	4.79E-14	1.86460925	0.315	0.067	1.55E-09	23
Tpm44	4.85E-14	1.99437627	0.63	0.257	1.57E-09	23
Lrrc581	1.18E-13	1.65520273	0.352	0.085	3.82E-09	23
Ets15	3.80E-13	1.71418283	0.481	0.155	1.23E-08	23
Cavin21	1.06E-12	1.5194782	0.296	0.063	3.41E-08	23
Ly6a4	1.91E-12	1.66718046	0.481	0.149	6.15E-08	23

Zbtb203	3.04E-12	1.91357607	0.315	0.074	9.82E-08	23
Tm4sf12	4.16E-12	1.49303026	0.537	0.182	1.34E-07	23
Rhoc	9.85E-12	1.92626736	0.278	0.062	3.18E-07	23
Rrbp14	2.48E-11	1.5728453	0.611	0.276	8.02E-07	23
Map4k41	3.01E-11	1.69552841	0.352	0.101	9.73E-07	23
Nid12	4.01E-11	1.90443065	0.278	0.065	1.30E-06	23
Itga62	7.33E-11	1.74161723	0.333	0.091	2.37E-06	23
Itm2c2	9.26E-11	1.62220054	0.259	0.058	2.99E-06	23
Rps6ka31	1.27E-10	1.35622152	0.259	0.059	4.09E-06	23
mt-Cytb7	2.47E-10	1.14723207	0.907	0.666	7.97E-06	23
mt-Atp67	9.68E-10	1.10556597	0.944	0.748	3.13E-05	23
Selenop2	9.85E-10	2.45078742	0.259	0.064	3.18E-05	23
Calm14	1.13E-09	1.25323233	0.796	0.649	3.66E-05	23
Sptbn13	5.42E-09	1.84874527	0.407	0.161	0.00017512	23
AW1120102	5.71E-09	1.10701342	0.296	0.083	0.00018419	23
Ctnna11	2.80E-08	1.28916527	0.333	0.109	0.00090492	23
Cavin11	3.14E-08	1.55832884	0.278	0.081	0.00101469	23
B2m6	4.82E-08	0.9644394	0.852	0.712	0.00155704	23
Hif1a3	5.10E-08	1.39724079	0.426	0.175	0.00164793	23
Cd813	8.63E-08	1.87824057	0.407	0.173	0.00278466	23
Marcks8	2.04E-07	1.41769456	0.444	0.2	0.00657726	23
Atox14	3.12E-07	1.31038974	0.63	0.396	0.01008637	23
Actn41	4.04E-07	1.87530712	0.37	0.158	0.01304651	23
Csrp12	8.89E-07	1.73557265	0.259	0.085	0.02871215	23
mt-Nd47	1.06E-06	0.99084119	0.722	0.492	0.03419927	23
Fabp54	1.50E-06	1.83206919	0.296	0.112	0.04841141	23
Marcksl13	1.96E-06	1.36827483	0.296	0.112	0.06315165	23
App3	2.25E-06	1.38392998	0.37	0.166	0.07262381	23
Lars25	2.65E-06	1.5252728	0.481	0.24	0.0853951	23
Ly6e9	4.41E-06	1.09044563	0.648	0.408	0.14224053	23
mt-Co36	4.91E-06	0.82354952	0.907	0.799	0.15835842	23
Macf11	5.01E-06	1.6111701	0.315	0.131	0.161852	23
Rhob1	5.60E-06	1.32163176	0.296	0.114	0.18091376	23
Ptms6	6.98E-06	1.24832095	0.389	0.174	0.22527069	23
Pdia37	8.45E-06	1.10778905	0.481	0.259	0.27278357	23
mt-Nd14	9.47E-06	0.7867629	0.704	0.477	0.30579733	23
Gnb11	1.09E-05	1.43152544	0.444	0.247	0.3505846	23
Msn2	1.70E-05	1.69017308	0.5	0.317	0.54956583	23
Tmem176b4	1.75E-05	1.44439943	0.296	0.12	0.56433049	23
Atrx	1.87E-05	1.28638543	0.37	0.18	0.6021808	23
Igfbp44	2.03E-05	1.10461962	0.389	0.176	0.65512899	23
Ctsb7	2.91E-05	1.10504245	0.444	0.242	0.93942794	23
Hspa57	3.22E-05	1.08086651	0.556	0.359	1	23

Zmiz1	3.47E-05	1.21137395	0.259	0.1	1	23
Rock22	3.66E-05	1.46338847	0.333	0.156	1	23
Zfp36l15	3.78E-05	0.90611573	0.315	0.131	1	23
Morf4l2	4.99E-05	1.22061101	0.296	0.13	1	23
Cfl15	8.57E-05	0.90056123	0.722	0.622	1	23
Ctsl6	9.88E-05	2.21535517	0.389	0.212	1	23
Ccnd12	0.00010468	0.97332609	0.315	0.136	1	23
Jak13	0.00011065	1.47576084	0.315	0.155	1	23
Eef1a16	0.00014897	0.47130243	0.907	0.728	1	23
Prkar1a3	0.00018208	1.36839426	0.389	0.23	1	23
Ifitm37	0.0002301	0.9535201	0.611	0.468	1	23
Prrc2c4	0.00025739	1.27980981	0.407	0.245	1	23
Myl64	0.00035201	0.88538878	0.741	0.708	1	23
Eif4g1	0.00039017	1.18695577	0.278	0.134	1	23
Hspa85	0.00040029	1.06243499	0.648	0.553	1	23
Rps154	0.00041113	0.56898351	0.722	0.537	1	23
Lrg15	0.00057259	1.99671738	0.315	0.176	1	23
Vim5	0.00057983	0.99182845	0.63	0.493	1	23
Calu3	0.00066587	0.96983528	0.278	0.134	1	23
mt-Nd26	0.00068963	0.8978023	0.556	0.384	1	23
Hsp90ab13	0.00077179	0.32963169	0.815	0.584	1	23
Ctnnb1	0.00088767	1.10382012	0.296	0.151	1	23
Rps254	0.00123099	0.55316325	0.796	0.631	1	23
Gnas4	0.00125762	0.90531639	0.574	0.446	1	23
Canx4	0.00131074	1.02734492	0.389	0.241	1	23
Degs15	0.00141102	1.35618071	0.296	0.171	1	23
Actg17	0.00141439	0.57214278	0.759	0.717	1	23
H2-K19	0.00147564	0.80523405	0.574	0.461	1	23
Myh94	0.00151922	1.15687656	0.463	0.355	1	23
AY0361184	0.00155365	1.19276246	0.426	0.293	1	23
Rdx2	0.00160125	1.2757138	0.315	0.179	1	23
mt-Co18	0.00163499	0.44616669	0.944	0.884	1	23
Rack16	0.00169994	0.75969126	0.667	0.537	1	23
Tpm14	0.00185876	0.56930872	0.352	0.187	1	23
Hsp90b19	0.00213882	0.75922362	0.519	0.381	1	23
Cltc1	0.00222199	1.10625938	0.37	0.238	1	23
Atp1b32	0.00226834	1.37149459	0.259	0.14	1	23
Rpl55	0.00231893	0.7028534	0.611	0.463	1	23
Skp1a5	0.00239188	1.1888343	0.352	0.225	1	23
Plec2	0.00245733	0.8576609	0.333	0.188	1	23
Ptges34	0.00249466	0.92328132	0.37	0.234	1	23
Serpinb6a1	0.00265226	0.67515428	0.278	0.139	1	23
Sh3glb11	0.00301273	0.8893808	0.444	0.314	1	23

Ybx14	0.00305855	0.73207341	0.574	0.452	1	23
Mef2c7	0.00369114	0.68686983	0.296	0.158	1	23
Wnk14	0.00394582	0.93041227	0.333	0.203	1	23
Pdia65	0.0045216	1.00338763	0.278	0.158	1	23
Galnt11	0.00505182	0.96599621	0.259	0.142	1	23
Tm9sf33	0.00517871	1.17778346	0.333	0.216	1	23
Rpl3112	0.0052197	0.46881514	0.685	0.546	1	23
U2af23	0.00550763	0.86862324	0.259	0.143	1	23
Rac13	0.00555956	0.96846924	0.407	0.296	1	23
Nedd45	0.00566834	0.73386421	0.37	0.226	1	23
mt-Co25	0.00627277	0.56873013	0.889	0.786	1	23
Pcbp15	0.00671443	0.99153972	0.37	0.252	1	23
Slc25a33	0.00684606	0.7685286	0.481	0.393	1	23
Fus6	0.00723401	1.02637976	0.37	0.258	1	23
Gnai24	0.00747602	1.0300363	0.537	0.495	1	23
Calr7	0.00812303	0.8383804	0.37	0.246	1	23
Cox7c8	0.00846496	0.3893795	0.778	0.674	1	23
Zfand53	0.00954013	1.30256631	0.296	0.196	1	23
Tra2a1	0.00994705	0.95090133	0.315	0.206	1	23
