# Supplementary material for: Loss of Notch signaling in skeletal stem cells enhances bone formation with aging
Source: Bone Res. 2023 Sep 27;11:50. doi: 10.1038/s41413-023-00283-8 (PMC10522593; doi:10.1038/s41413-023-00283-8)
Supplement: Supplementary file 3 — Supplementary Table S2 [file 41413_2023_283_MOESM3_ESM.pdf]

Cluster 1 Figure 2B Differential Gene Expression *Ncstn* ckO vs control

	p_val	avg_log2FC	pct.1	pct.2	p_val_adj
Mmp2	2.25E-05	2.51516729	0.375	0	0.72548448
Lepr	4.29E-05	-1.4012554	0.583	0.939	1
H2-Q7	0.00012308	-1.473122	0.125	0.636	1
Sept4	0.00019393	2.00370601	0.375	0.03	1
Cebpb	0.00030111	-1.5467501	0.208	0.667	1
Rpl18a	0.0004444	-1.2816406	0.5	0.848	1
Kif5b	0.00067442	1.51722768	0.333	0.03	1
Sub1	0.00074185	-1.3897624	0.125	0.576	1
Txndc15	0.00074703	1.33198858	0.25	0	1
Map1b	0.00074703	1.63994326	0.25	0	1
Trappc8	0.00074703	0.98423879	0.25	0	1
Pds5b	0.00074703	1.15616983	0.25	0	1
Ints6	0.00088705	-1.3612633	0	0.273	1
Glrx2	0.00088705	-1.5979079	0	0.273	1
Limch1	0.00100162	2.13967595	0.5	0.152	1
Ifitm3	0.00117224	-0.6273099	0.833	1	1
Ubb	0.00117326	-1.1276514	0.625	0.939	1
Cd1d1	0.00119931	-1.3866261	0.042	0.424	1
Cox7a2	0.00125251	-1.2816364	0.208	0.606	1
Ryk	0.00141021	-1.5811971	0.083	0.364	1
Ptx3	0.00146297	-1.8948008	0.167	0.515	1
Ncam1	0.00151277	1.56123318	0.375	0.061	1
Epas1	0.00172521	-1.2938658	0.292	0.697	1
Ccl19	0.00176693	-1.7739747	0.167	0.515	1
Fosb	0.00177184	-2.2022043	0.208	0.606	1
Deptor	0.00185905	-1.1356413	0	0.242	1
Mob4	0.00185905	-1.284721	0	0.242	1
Snx4	0.00185905	-1.4403636	0	0.242	1
Alyref	0.00185905	-1.4548181	0	0.242	1
Col5a2	0.00187309	1.29754882	0.583	0.182	1
Lrp1	0.00195892	1.26297771	0.833	0.515	1
Btg2	0.00212783	-1.5749532	0.083	0.394	1
Nfkbiz	0.00222268	-1.8973666	0.083	0.364	1
B4galt1	0.00222811	1.31225417	0.333	0.03	1
Fign	0.00226592	1.36085174	0.208	0	1
Smoc2	0.00226592	3.10326418	0.208	0	1
Dctn1	0.00226592	1.16911571	0.208	0	1
Tex264	0.00226592	1.34677327	0.208	0	1
Hbb-bt	0.00226592	1.60337031	0.208	0	1
1-Mar	0.00226592	1.46337282	0.208	0	1
Satb2	0.00226592	1.12781677	0.208	0	1

S100a4	0.00226592	2.14866268	0.208	0	1
Chst2	0.00226592	1.57125371	0.208	0	1
Gm12216	0.00246184	-1.5602294	0.042	0.303	1
Syncrip	0.00254545	1.54191915	0.375	0.061	1
Serpina3g	0.00260658	-1.1715878	0.167	0.576	1
Irs2	0.00279614	-1.3770629	0.083	0.424	1
Egr1	0.0028343	-2.0715251	0.292	0.636	1
Pnrc1	0.00292851	-1.67051	0.125	0.424	1
Clmp	0.00293998	1.78175473	0.458	0.152	1
Itga11	0.00319804	1.3291693	0.375	0.061	1
Osmr	0.00323355	-1.3197493	0.083	0.364	1
Snrpb	0.00338034	-1.2658111	0.125	0.455	1
Angpt4	0.00340205	1.53333901	0.292	0.03	1
Fbxo11	0.00346873	-1.3513351	0.042	0.273	1
Psma2	0.0038052	-1.1917205	0.125	0.485	1
Igfbp3	0.00384191	-1.8231717	0	0.212	1
Cfap20	0.00384191	-1.0064778	0	0.212	1
Gpsm3	0.00384191	-1.0193139	0	0.212	1
Arhgap42	0.00384191	-1.5340244	0	0.212	1
Lrrc8a	0.00384191	-1.1246926	0	0.212	1
Mrc2	0.00384191	-1.2167816	0	0.212	1
Appbp2	0.00384191	-1.2979814	0	0.212	1
Ncstn	0.00384191	-1.2350726	0	0.212	1
Copb1	0.00384191	-1.2583649	0	0.212	1
Tcp11l2	0.00384191	-1.2635298	0	0.212	1
Rictor	0.00384191	-1.4286918	0	0.212	1
Milr1	0.00384191	-1.6195921	0	0.212	1
Eif3f	0.00384826	1.66968519	0.458	0.152	1
Tmem259	0.00398154	1.6826273	0.333	0.03	1
Clu	0.0044002	-0.949164	0.375	0.758	1
Comp	0.00445752	2.29260623	0.333	0.091	1
Sfrp1	0.00457761	2.4256491	0.417	0.182	1
Dhrs7	0.00462605	-1.4468067	0.042	0.273	1
Tmsb10	0.00477657	-0.800769	0.792	0.97	1
Phrf1	0.00506377	1.43347399	0.292	0.061	1
Cldn10	0.00511965	-1.5705307	0.167	0.455	1
Fos	0.00623904	-2.105508	0.292	0.576	1
Junb	0.00651187	-1.4367548	0.292	0.636	1
Tent5a	0.00657908	-1.0965819	0.042	0.364	1
Spry1	0.00659306	-1.1363702	0.167	0.515	1
Top1	0.00668039	-1.4842764	0.125	0.455	1
Med15	0.00674515	0.72161651	0.167	0	1
Thoc6	0.00674515	0.75179443	0.167	0	1

Flot2	0.00674515	0.89356438	0.167	0	1
Cic	0.00674515	0.7462127	0.167	0	1
Zfp318	0.00674515	1.35927707	0.167	0	1
Srebf1	0.00674515	0.79653088	0.167	0	1
Luc7l3	0.00674515	0.97268617	0.167	0	1
Zfp467	0.00674515	0.97031766	0.167	0	1
Babam2	0.00674515	0.97410471	0.167	0	1
Hras	0.00674515	0.83316989	0.167	0	1
4833420G17	0.00674515	1.02004132	0.167	0	1
Mrpl44	0.00674515	0.84220236	0.167	0	1
Tmem9	0.00674515	0.81610075	0.167	0	1
Prxl2c	0.00674515	1.11355382	0.167	0	1
Socs5	0.00674515	1.06408628	0.167	0	1
Fip1l1	0.00674515	0.81021347	0.167	0	1
Fat4	0.00674515	1.08463419	0.167	0	1
Tlcd1	0.00674515	1.06574306	0.167	0	1
Rab43	0.00674515	1.26878597	0.167	0	1
Pdcl3	0.00674515	1.29576504	0.167	0	1
Bglap2	0.00674515	1.58814464	0.167	0	1
BC005624	0.00674515	1.50231417	0.167	0	1
Amotl1	0.00674515	1.13377772	0.167	0	1
Sulf2	0.00674515	0.54404286	0.167	0	1
Zfp36	0.00676366	-2.1014523	0.167	0.485	1
Josd2	0.00688563	1.11955061	0.25	0.03	1
Gxylt2	0.00690016	1.3554014	0.292	0.061	1
Iigp1	0.00694987	-1.6263037	0.208	0.545	1
Hsd17b11	0.00709682	1.11666943	0.208	0.03	1
Msl1	0.00712663	1.22491458	0.292	0.03	1
Col4a1	0.0071332	1.0953353	0.708	0.364	1
Fus	0.00724135	-1.1712362	0.25	0.576	1
Glul	0.00756332	-1.4500466	0.125	0.455	1
Rps27	0.00757256	-0.4217446	0.667	0.97	1
Mir100hg	0.00758836	1.27451356	0.333	0.061	1
Man1b1	0.00760389	1.24136676	0.292	0.03	1
Ifrd1	0.00763183	-1.9554879	0.125	0.424	1
Rnf19b	0.00774626	-1.6219571	0.083	0.333	1
Ppp4r2	0.00783562	-1.1607838	0.042	0.242	1
Pappa	0.00784796	-1.0243824	0.292	0.667	1
Lgalsl	0.00785025	-0.9209823	0	0.182	1
Tgif1	0.00785025	-0.9231542	0	0.182	1
Gpbp1l1	0.00785025	-0.9589163	0	0.182	1
Rpgrip1	0.00785025	-0.9711715	0	0.182	1
Pycard	0.00785025	-0.9799797	0	0.182	1

Ywhag	0.00785025	-1.0654309	0	0.182	1
Pcmtd1	0.00785025	-1.4992047	0	0.182	1
Maob	0.00785025	-1.0673095	0	0.182	1
Ifi203	0.00807271	-1.2947938	0.083	0.394	1
Commd1	0.00807798	-1.3126236	0.042	0.242	1
Npm1	0.00818674	-1.2391393	0.167	0.455	1
Mgp	0.00828614	-1.4440126	0.625	0.818	1
Nr1d2	0.00853948	-1.2281049	0.042	0.242	1
Mdk	0.00860621	-0.9627231	0.25	0.606	1
Nasp	0.00868802	-1.1486908	0.042	0.242	1
Tmem109	0.00870859	-1.1191463	0.042	0.242	1
Ywhaq	0.00894591	-1.0049441	0.125	0.455	1
Rad23a	0.00976976	1.07218782	0.208	0.03	1
Lpar1	0.0098588	1.32037302	0.333	0.061	1
Grcc10	0.00994277	-1.4537875	0.167	0.394	1
Ddr2	0.01014891	1.51314746	0.333	0.091	1
Tut4	0.01015264	1.60187844	0.333	0.091	1
Anxa1	0.01037422	-1.4123747	0.292	0.606	1
Chd3	0.01045383	1.12645091	0.25	0.061	1
Eif1	0.01072797	-0.555003	0.583	0.879	1
Acin1	0.01080471	1.3947579	0.333	0.091	1
Cxcl10	0.01090278	-2.3319685	0.083	0.333	1
Adamts1	0.01095147	-1.3712705	0.167	0.515	1
Ddx3x	0.01117599	-1.1166451	0.125	0.424	1
Uqcc2	0.01173244	1.29095104	0.375	0.121	1
mt-Atp8	0.0122376	1.18879137	0.75	0.545	1
Capzb	0.01235668	1.47043211	0.333	0.091	1
Degs1	0.01238854	-1.1686591	0.125	0.394	1
Il34	0.01275449	-1.547471	0.167	0.424	1
Angptl4	0.01291777	1.14377514	0.375	0.061	1
Tfg	0.01319779	1.06265963	0.208	0.03	1
Rab10	0.01325569	-1.0700286	0.042	0.273	1
mt-Nd5	0.01350219	0.76909672	0.708	0.394	1
Ccl2	0.01354735	-3.1058538	0.083	0.364	1
Wsb1	0.01368654	-1.1332012	0.167	0.455	1
Ifi211	0.01375388	-0.8553429	0.083	0.364	1
Mfge8	0.014098	-1.2400852	0.125	0.364	1
Zfp950	0.01431731	1.3132624	0.167	0.03	1
Sod1	0.01487409	-0.9943298	0.083	0.333	1
Ptgfrn	0.01488878	1.21235147	0.25	0.03	1
Cr1l	0.01496304	1.09205847	0.208	0.03	1
Ermp1	0.01498257	-1.1462032	0.042	0.273	1
Rpl21	0.01534926	-0.9565117	0.333	0.636	1

Hk2	0.01543334	-2.2412948	0.208	0.394	1
Hbp1	0.0156256	1.11259259	0.167	0.03	1
Wif1	0.01569898	1.20435948	0.5	0.212	1
Cxcl12	0.01586059	-0.3880294	0.792	0.97	1
Atf2	0.01586988	1.04692067	0.167	0.03	1
Exosc10	0.0159131	-0.9755692	0	0.152	1
Nabp1	0.0159131	-0.9062414	0	0.152	1
Srsf9	0.0159131	-1.0752153	0	0.152	1
Csnk2a2	0.0159131	-1.0169003	0	0.152	1
Epb41l4b	0.0159131	-1.0534064	0	0.152	1
Htra2	0.0159131	-1.0721928	0	0.152	1
Itga5	0.0159131	-0.9338775	0	0.152	1
Ier5l	0.0159131	-0.9664921	0	0.152	1
Sidt2	0.0159131	-1.006701	0	0.152	1
Map2k2	0.0159131	-0.921859	0	0.152	1
Chsy1	0.0159131	-0.9605653	0	0.152	1
Grb2	0.0159131	-0.647611	0	0.152	1
Czib	0.0159131	-1.3024963	0	0.152	1
Phax	0.0159131	-1.0798385	0	0.152	1
Rnf24	0.0159131	-1.0732145	0	0.152	1
Stam2	0.0159131	-0.6524506	0	0.152	1
Adamtsl3	0.0159131	-0.7695091	0	0.152	1
Rai1	0.0159131	-0.6561673	0	0.152	1
Sfswap	0.0159131	-0.9737774	0	0.152	1
Oasl2	0.0159131	-0.9951598	0	0.152	1
Zfp703	0.0159131	-0.6841618	0	0.152	1
Mettl7a1	0.0159131	-1.0234107	0	0.152	1
Mark1	0.0159131	-0.7199754	0	0.152	1
Gprin3	0.0159131	-0.8532176	0	0.152	1
Bcl3	0.0159131	-0.7427853	0	0.152	1
Ahi1	0.0159131	-0.871696	0	0.152	1
Gatad2a	0.0159131	-0.833712	0	0.152	1
Necap2	0.0159131	-0.886751	0	0.152	1
Rab11a	0.0159131	-0.7899086	0	0.152	1
Tspyl1	0.0159131	-0.8407612	0	0.152	1
Gm4070	0.0159131	-0.9096699	0	0.152	1
Upf3a	0.0159131	-1.1414145	0	0.152	1
Tmem19	0.0159131	-0.8044698	0	0.152	1
Zmat5	0.0159131	-0.9270771	0	0.152	1
Nipsnap2	0.0159131	-0.9652642	0	0.152	1
Kcnip3	0.0159131	-1.2430188	0	0.152	1
Hic1	0.0159131	-0.8399592	0	0.152	1
Vezf1	0.0159131	-0.9720942	0	0.152	1

Cers2	0.0161408	1.63948678	0.25	0.03	1
Vamp5	0.01622984	1.37705453	0.25	0.061	1
Vamp3	0.01629302	1.06832531	0.167	0.03	1
Rps29	0.01649237	-0.3131454	0.792	1	1
Dnajb9	0.01653045	-1.4721084	0.125	0.394	1
Islr	0.01664189	1.05718131	0.667	0.424	1
Gdpd2	0.01667774	-0.5598675	0.542	0.848	1
Polr2a	0.01697977	1.11046066	0.25	0.03	1
Fkbp9	0.01717035	1.29265598	0.458	0.212	1
Steap4	0.01717215	-1.1586612	0.083	0.333	1
Mtmr3	0.01790615	0.86401951	0.167	0.03	1
Ubxn4	0.01801492	1.04732636	0.25	0.03	1
Lrba	0.01804342	0.82636295	0.167	0.03	1
Srsf7	0.0180997	-1.2679907	0.125	0.364	1
Pgrmc1	0.01815697	-1.2276896	0.125	0.394	1
Nfkbia	0.0184864	-1.3454611	0.292	0.576	1
mt-Nd4	0.01859164	0.90254055	0.917	0.788	1
Palld	0.01917275	1.36233506	0.417	0.152	1
Dars	0.01940201	1.00820515	0.208	0.03	1
Sumo2	0.01950729	-1.0699499	0.083	0.303	1
Golga1	0.01989681	0.79789085	0.125	0	1
Med8	0.01989681	1.06108409	0.125	0	1
Otud1	0.01989681	0.82356104	0.125	0	1
Ppfibp2	0.01989681	0.82356104	0.125	0	1
Zfp771	0.01989681	0.66102039	0.125	0	1
Traf3	0.01989681	0.66102039	0.125	0	1
Ddx46	0.01989681	0.66220191	0.125	0	1
Hipk2	0.01989681	0.83449763	0.125	0	1
Iars	0.01989681	0.66897174	0.125	0	1
Akirin1	0.01989681	0.60936706	0.125	0	1
Tha1	0.01989681	0.80067751	0.125	0	1
Orai2	0.01989681	0.79153823	0.125	0	1
Ubfd1	0.01989681	0.61893634	0.125	0	1
Gsdme	0.01989681	0.82102896	0.125	0	1
Lama5	0.01989681	0.873841	0.125	0	1
Cpsf3	0.01989681	0.81313273	0.125	0	1
Airn	0.01989681	0.68961707	0.125	0	1
Lrch4	0.01989681	0.77325511	0.125	0	1
Tmem107	0.01989681	1.01182535	0.125	0	1
Plpbp	0.01989681	0.59064706	0.125	0	1
Helz	0.01989681	0.84331649	0.125	0	1
Rars	0.01989681	1.31789993	0.125	0	1
Nup54	0.01989681	0.85735302	0.125	0	1

Zbtb33	0.01989681	0.73906617	0.125	0	1
Clcn5	0.01989681	0.73942743	0.125	0	1
Bloc1s5	0.01989681	0.90642366	0.125	0	1
Adamts15	0.01989681	1.37496434	0.125	0	1
Atp6v1d	0.01989681	0.81115095	0.125	0	1
Ift46	0.01989681	0.97077422	0.125	0	1
Myoc	0.01989681	1.65365166	0.125	0	1
Ints7	0.01989681	0.78550369	0.125	0	1
Mrpl9	0.01989681	0.94764145	0.125	0	1
Ccdc6	0.01989681	0.83515382	0.125	0	1
Siae	0.01989681	0.78918526	0.125	0	1
Hars	0.01989681	0.69060699	0.125	0	1
Hps4	0.01989681	0.80606879	0.125	0	1
Adamtsl5	0.01989681	0.97357923	0.125	0	1
Dvl3	0.01989681	0.96131699	0.125	0	1
Tfdp2	0.01989681	0.99910164	0.125	0	1
Gopc	0.01989681	0.98028751	0.125	0	1
5730409E04l	0.01989681	0.87545669	0.125	0	1
St3gal3	0.01989681	1.11049831	0.125	0	1
Pik3c3	0.01989681	0.9282163	0.125	0	1
Slc22a3	0.01989681	0.68665004	0.125	0	1
Dolk	0.01989681	1.1204044	0.125	0	1
Plaat3	0.01989681	1.02821421	0.125	0	1
Afg3l2	0.01989681	1.01976907	0.125	0	1
Igfbp6	0.01989681	1.6034974	0.125	0	1
Ap1g1	0.01989681	1.02439314	0.125	0	1
Fam210a	0.01989681	1.01447592	0.125	0	1
Cmtm3	0.01989681	0.83897621	0.125	0	1
Apobec3	0.01989681	1.0656033	0.125	0	1
Tbc1d8b	0.01989681	0.85982792	0.125	0	1
Ppa2	0.01989681	0.86180761	0.125	0	1
Tnxb	0.01989681	2.0279108	0.125	0	1
Ell	0.01989681	0.33203303	0.125	0	1
Ccdc3	0.01989681	1.13760108	0.125	0	1
Aggf1	0.01989681	1.24311659	0.125	0	1
Dbn1	0.01989681	0.73764758	0.125	0	1
Postn	0.01989681	1.59923369	0.125	0	1
Lnpk	0.01989681	0.98176692	0.125	0	1
Apip	0.01989681	1.4032772	0.125	0	1
Stk24	0.01989681	0.78479265	0.125	0	1
Rgmb	0.01989681	0.48619584	0.125	0	1
Dpt	0.01989681	2.4875975	0.125	0	1
Otub1	0.01989681	0.39756962	0.125	0	1

Agap1	0.01989681	1.15978248	0.125	0	1
Mark4	0.01989681	1.17156621	0.125	0	1
Gstz1	0.01989681	1.56659265	0.125	0	1
Mif4gd	0.01989681	1.10241157	0.125	0	1
Stat2	0.01989681	1.22520383	0.125	0	1
Prkab1	0.01989681	0.54298722	0.125	0	1
Pgap2	0.01989681	0.44890834	0.125	0	1
Snx13	0.01989681	0.44890834	0.125	0	1
Vps54	0.01989681	0.57689223	0.125	0	1
Gli3	0.01989681	0.47900496	0.125	0	1
Sumf1	0.01989681	1.25051585	0.125	0	1
Pop5	0.01989681	0.60189803	0.125	0	1
Smarcad1	0.01989681	0.61996552	0.125	0	1
Aspn	0.01989681	1.11042813	0.125	0	1
Tbc1d17	0.01989681	0.50451415	0.125	0	1
Ggps1	0.01989681	0.62786	0.125	0	1
Egln1	0.01989681	0.61797533	0.125	0	1
Cdc14b	0.01989681	0.67760262	0.125	0	1
Numb	0.01989681	0.6849048	0.125	0	1
Pxn	0.01989681	0.56020967	0.125	0	1
Mturn	0.01989681	0.65567415	0.125	0	1
Larp7	0.01989681	0.56722769	0.125	0	1
Stard13	0.01989681	0.66041209	0.125	0	1
Zfp866	0.01989681	0.66041209	0.125	0	1
Ssh1	0.01989681	0.58496542	0.125	0	1
Edc4	0.01989681	0.58028119	0.125	0	1
Nek4	0.01989681	0.74347832	0.125	0	1
Atf1	0.01989681	0.74462781	0.125	0	1
Klhdc3	0.01989681	0.61951655	0.125	0	1
Lpcat1	0.01989681	0.90872288	0.125	0	1
Ppp4r3a	0.01989681	1.02450363	0.125	0	1
Tep1	0.01989681	0.70647375	0.125	0	1
Prg4	0.01989681	1.18946507	0.125	0	1
Entpd7	0.01989681	0.78191639	0.125	0	1
Nr1d1	0.02012935	-1.0780082	0.042	0.212	1
Trp53i13	0.02027156	-0.8221738	0.042	0.303	1
Plscr4	0.02045769	1.28646147	0.292	0.091	1
Sphk1	0.02073298	-0.9112824	0.042	0.212	1
Snrpf	0.02097823	-1.1488832	0.167	0.455	1
Reps1	0.02125126	1.02610137	0.167	0.03	1
Macf1	0.02130801	0.67687577	0.5	0.152	1
Zc3h14	0.02131002	0.91247365	0.167	0.03	1
Plxna2	0.02146891	1.21364934	0.458	0.212	1

Plpp3	0.02164206	-0.9676322	0.458	0.697	1
Atp2b1	0.0216787	-1.1789333	0.083	0.333	1
Sec13	0.02170174	-0.8382798	0.042	0.212	1
H3f3b	0.02193003	-1.1263785	0.583	0.788	1
Ube2f	0.02223988	0.72447539	0.167	0.03	1
Use1	0.02229326	-0.8713757	0.042	0.212	1
Rps15a	0.02230269	-0.7394649	0.583	0.818	1
Srsf3	0.02240893	-0.9662348	0.167	0.455	1
Selenok	0.02243689	-0.7323563	0.375	0.667	1
Rhoj	0.02256042	1.12138261	0.208	0.03	1
Pcbd2	0.02263061	-1.0555568	0.042	0.212	1
Dpysl3	0.02312906	1.27181105	0.5	0.273	1
Slc36a2	0.02365532	1.1074409	0.25	0.03	1
Scamp3	0.02366847	0.7380467	0.208	0.03	1
Nradd	0.02406265	0.63048341	0.167	0.03	1
Cebpz	0.02412851	0.99188541	0.25	0.061	1
Ctdnep1	0.02413601	0.94586488	0.292	0.061	1
Cox7b	0.02423543	-0.9874026	0.167	0.424	1
Plec	0.02432414	1.22400028	0.292	0.121	1
Hagh	0.02436799	1.67329033	0.292	0.091	1
Rps16	0.02465307	-0.9820904	0.542	0.758	1
Mdh2	0.02484387	0.98905791	0.208	0.03	1
Pnkd	0.0251603	-0.8556594	0.042	0.273	1
Ctsd	0.02537735	0.86552438	0.583	0.303	1
1810026B05I	0.02598819	-1.3530549	0.083	0.273	1
Smarca5	0.02612304	-1.1764116	0.083	0.273	1
Sdc1	0.02616453	-1.2408481	0.125	0.394	1
Mtx1	0.02621366	-1.1518554	0.042	0.182	1
Pcgf5	0.02622812	-0.9664312	0.042	0.242	1
Dcun1d5	0.02625082	-0.9653292	0.042	0.273	1
Camk2g	0.02631876	0.90028518	0.167	0.03	1
Srpx	0.02633631	-0.9592543	0.083	0.333	1
Polr1d	0.0264706	-0.8848398	0.083	0.333	1
Rps18	0.02656493	-0.4187376	0.333	0.667	1
Sec11c	0.02662917	-1.0126905	0.042	0.242	1
Lamtor5	0.02676103	1.18126976	0.292	0.091	1
Prmt1	0.02739635	-1.2350289	0.042	0.182	1
Supt16	0.02754665	-1.1222192	0.042	0.212	1
Psmc6	0.02758603	-1.254304	0.042	0.182	1
Mmp14	0.02795802	1.61266408	0.333	0.152	1
Irf2bp2	0.0279845	-1.1066829	0.25	0.485	1
Hes1	0.02799461	-1.3174106	0.25	0.515	1
Col1a2	0.02804334	0.48629164	0.917	0.788	1

Sec11a	0.02833645	1.05310679	0.25	0.061	1
Qpct	0.0283505	0.9338872	0.25	0.03	1
Tardbp	0.02863689	0.9985885	0.292	0.061	1
Hnrnpr	0.02867382	-0.8384207	0.083	0.303	1
Plppr3	0.02881579	1.23345507	0.208	0.061	1
Saraf	0.02882438	-1.0961859	0.167	0.394	1
Gas1	0.02906916	1.26947379	0.25	0.061	1
Taldo1	0.02925744	-0.9546792	0.042	0.182	1
Vapb	0.0296192	-0.9489137	0.042	0.182	1
Emilin1	0.02991955	1.51594313	0.375	0.152	1
Slc38a2	0.03039528	-1.1595353	0.292	0.576	1
Tnfrsf1a	0.03041433	-1.1402039	0.167	0.394	1
Emp2	0.03133842	-1.0976842	0.125	0.333	1
Cdh2	0.03187697	1.06797775	0.542	0.303	1
Washc4	0.03187709	1.31863933	0.208	0.03	1
Atl3	0.03203507	1.14519289	0.208	0.061	1
Mtfr1l	0.0321485	-0.7260449	0	0.121	1
Rsf1	0.0321485	-0.7324032	0	0.121	1
Leng1	0.0321485	-0.7521194	0	0.121	1
Plagl1	0.0321485	-1.3544459	0	0.121	1
Slc35b2	0.0321485	-0.8897893	0	0.121	1
Myo1c	0.0321485	-0.8870447	0	0.121	1
Kcmf1	0.0321485	-0.8612903	0	0.121	1
Grpel1	0.0321485	-0.8431269	0	0.121	1
Lpcat4	0.0321485	-0.9629962	0	0.121	1
Grk2	0.0321485	-0.4740983	0	0.121	1
Srcap	0.0321485	-1.0277298	0	0.121	1
Crkl	0.0321485	-0.9110953	0	0.121	1
Rac3	0.0321485	-0.7357457	0	0.121	1
Kdm6a	0.0321485	-0.7523286	0	0.121	1
Gm17501	0.0321485	-0.8953352	0	0.121	1
Fxr2	0.0321485	-0.8888389	0	0.121	1
Ube2o	0.0321485	-0.8125104	0	0.121	1
P3h2	0.0321485	-0.9122077	0	0.121	1
Tor3a	0.0321485	-1.069005	0	0.121	1
AI506816	0.0321485	-0.4703992	0	0.121	1
Gatd1	0.0321485	-0.4912074	0	0.121	1
Eaf1	0.0321485	-0.748554	0	0.121	1
Slco1a4	0.0321485	-0.8437749	0	0.121	1
Ly96	0.0321485	-1.0166511	0	0.121	1
Zfp945	0.0321485	-0.7406788	0	0.121	1
Ccdc9	0.0321485	-1.0013227	0	0.121	1
Tlnrd1	0.0321485	-1.2015795	0	0.121	1

Slc25a51	0.0321485	-0.5733187	0	0.121	1
Oard1	0.0321485	-0.7700181	0	0.121	1
Twf2	0.0321485	-0.5337202	0	0.121	1
Inpp5k	0.0321485	-0.6169443	0	0.121	1
Mrpl18	0.0321485	-0.5480483	0	0.121	1
Tdrd3	0.0321485	-0.7982261	0	0.121	1
Adam15	0.0321485	-0.7882218	0	0.121	1
Rrp15	0.0321485	-0.9635192	0	0.121	1
Pcdhb4	0.0321485	-0.8182914	0	0.121	1
Tcf3	0.0321485	-0.6083484	0	0.121	1
Iffo1	0.0321485	-0.6729495	0	0.121	1
Ncoa4	0.0321485	-0.5685689	0	0.121	1
Snx15	0.0321485	-0.5761929	0	0.121	1
Ndufa8	0.0321485	-0.8447697	0	0.121	1
Mbd1	0.0321485	-0.574048	0	0.121	1
Nfya	0.0321485	-0.6689642	0	0.121	1
Afg3l1	0.0321485	-0.628376	0	0.121	1
Scfd1	0.0321485	-0.6288626	0	0.121	1
Dhx30	0.0321485	-0.5933295	0	0.121	1
Uck2	0.0321485	-0.5933181	0	0.121	1
Zfp131	0.0321485	-0.6027035	0	0.121	1
Plac8	0.0321485	-1.2473425	0	0.121	1
Dtx4	0.0321485	-0.6927788	0	0.121	1
Npc1	0.0321485	-0.6132136	0	0.121	1
Ghdc	0.0321485	-0.7054815	0	0.121	1
C1qtnf12	0.0321485	-0.629917	0	0.121	1
Arfgap1	0.0321485	-0.7334226	0	0.121	1
Atp11a	0.0321485	-0.7327953	0	0.121	1
Wwox	0.0321485	-0.7281074	0	0.121	1
Atp9b	0.0321485	-0.6988132	0	0.121	1
Fmn1	0.0321485	-0.6944769	0	0.121	1
Ptpn2	0.0321485	-0.6641092	0	0.121	1
Itprid2	0.0321485	-0.6688349	0	0.121	1
Mapk1ip1l	0.0321485	-0.6712533	0	0.121	1
Cherp	0.0321485	-0.6725859	0	0.121	1
Prps1	0.0321485	-0.7166165	0	0.121	1
Scn1b	0.0321485	-0.775754	0	0.121	1
Plcl1	0.0321485	-0.6922445	0	0.121	1
Ahsa1	0.0321485	-0.7498845	0	0.121	1
Gpn3	0.0321485	-1.0043155	0	0.121	1
Bvht	0.0321485	-0.7992767	0	0.121	1
Mrps17	0.0321485	-0.4969952	0	0.121	1
Rnaset2b	0.0321485	-0.7737972	0	0.121	1

Rftn2	0.0321485	-0.7387361	0	0.121	1
Rnft1	0.0321485	-0.8519673	0	0.121	1
Ankrd27	0.0321485	-0.7733977	0	0.121	1
Tmem245	0.0321485	-0.8445213	0	0.121	1
2310009B15I	0.0321485	-0.8462351	0	0.121	1
Ints6l	0.0321485	-0.8000799	0	0.121	1
Llgl1	0.0321485	-0.8723884	0	0.121	1
E230032D23I	0.0321485	-0.7855679	0	0.121	1
Snx7	0.0321485	-0.7861967	0	0.121	1
Spry4	0.0321485	-0.8827657	0	0.121	1
Ip6k1	0.0321485	-0.9327495	0	0.121	1
Rnf187	0.0321485	-0.8344953	0	0.121	1
P2ry14	0.0321485	-0.925408	0	0.121	1
Mrps15	0.0321485	-0.8232295	0	0.121	1
Sucla2	0.0321485	-1.0210778	0	0.121	1
Golim4	0.0321485	-0.9086383	0	0.121	1
Ier5	0.03236717	-1.9213106	0.125	0.303	1
Timm10b	0.03278004	-1.1084818	0.083	0.273	1
Serf2	0.03298886	-0.8575747	0.542	0.758	1
Lrrfip2	0.03312157	0.91042844	0.208	0.061	1
Arl1	0.0332151	-1.1217719	0.125	0.364	1
Mdm4	0.03344162	1.0495832	0.25	0.061	1
Cdc42ep1	0.03439503	1.10712276	0.25	0.061	1
Rarres2	0.03445207	-0.8108765	0.417	0.667	1
Cxcl14	0.03452543	-0.5738691	0.708	0.939	1
Tmem176a	0.03464396	-0.4125461	0.625	0.879	1
Birc2	0.03506957	-1.0552183	0.042	0.182	1
Adipor1	0.03517639	1.1524058	0.167	0.03	1
Ak3	0.03520339	-1.00985	0.167	0.394	1
Aebp1	0.03556429	1.31806702	0.375	0.152	1
Cpne3	0.03562796	-1.0825444	0.125	0.333	1
Rpl15	0.03582249	-0.3733902	0.458	0.788	1
Sparc	0.03613479	0.45298474	0.958	0.848	1
Kdm6b	0.03636786	-1.3240287	0.208	0.394	1
Pitpna	0.03644901	0.84913348	0.292	0.061	1
Hint2	0.03663709	-0.9710807	0.042	0.242	1
Olfml2b	0.03709823	1.29205283	0.375	0.182	1
Gm11808	0.03770667	-1.0083564	0.042	0.182	1
mt-Co2	0.037839	0.94213445	0.958	0.909	1
Arl6ip1	0.03793816	-1.0513024	0.125	0.333	1
Mrps33	0.03796712	-0.903185	0.083	0.303	1
Tapbp	0.03822479	-0.6393973	0.167	0.455	1
Ubald2	0.03850308	-1.0583477	0.083	0.212	1

Myh9	0.03854654	-0.7045049	0.333	0.606	1
Abhd12	0.03858458	0.99003671	0.208	0.03	1
Tvp23b	0.03874195	1.05637981	0.167	0.03	1
Plscr3	0.03876054	1.44783287	0.25	0.121	1
Jund	0.03895305	-1.03452	0.333	0.606	1
Dhx36	0.03902508	0.96413221	0.208	0.061	1
Pten	0.03912667	-0.7568979	0.167	0.424	1
Bmp4	0.03930233	1.38100721	0.375	0.182	1
Ms4a4d	0.03936209	-1.2166386	0.167	0.333	1
Rpl3	0.03937976	-0.5801138	0.375	0.667	1
Nfia	0.03944633	-0.7775784	0.25	0.545	1
Ak2	0.03959965	-0.8887386	0.042	0.182	1
Raly	0.03966701	-0.8809424	0.083	0.303	1
Pcna	0.03999709	-0.9047383	0.083	0.273	1
Ednra	0.04004922	-0.9471118	0.042	0.242	1
Tm4sf1	0.04017701	1.16043334	0.292	0.121	1
Fgfr1op2	0.04033441	-0.7450663	0.042	0.182	1
Slc50a1	0.04074368	-0.863497	0.125	0.333	1
Cops2	0.04082441	0.85801733	0.208	0.061	1
Dnajc7	0.04100602	1.04717197	0.167	0.03	1
Socs1	0.04114286	-1.3843076	0.083	0.242	1
Eif4enif1	0.04135112	0.67852392	0.167	0.03	1
mt-Co1	0.04161846	0.87392642	0.958	0.939	1
Immp1l	0.0416369	0.81347557	0.208	0.061	1
Jun	0.04200774	-1.7302324	0.292	0.515	1
Plekhh2	0.04232861	-1.0457298	0.042	0.212	1
Mrpl33	0.04234187	0.8513201	0.458	0.212	1
Scpep1	0.04243747	0.90242687	0.208	0.03	1
Hspa8	0.04286861	-0.8984911	0.542	0.758	1
Sec24b	0.04314977	1.02633933	0.125	0.03	1
Rps3a1	0.043234	-0.5017131	0.542	0.788	1
Arl5a	0.04333889	-1.04408	0.083	0.212	1
Aplp2	0.04346156	-0.6678258	0.375	0.667	1
Odr4	0.04380225	1.14419962	0.125	0.03	1
Pfdn2	0.04403547	-1.0145673	0.125	0.303	1
Cxcl9	0.04425559	-1.7842351	0.083	0.303	1
Atxn1	0.0443214	1.02975693	0.292	0.091	1
Chd6	0.04444285	1.06609563	0.333	0.121	1
Smad7	0.04446625	-1.0400997	0.125	0.333	1
Psma5	0.0451199	-0.8485313	0.083	0.242	1
AC149090.1	0.04530421	1.24701243	0.333	0.152	1
Cetn3	0.04536426	-0.8515188	0.042	0.242	1
mt-Nd3	0.04567443	1.16164453	0.458	0.242	1

Cyfip1	0.04569629	0.74969941	0.25	0.061	1
Elp5	0.04572411	0.89649426	0.125	0.03	1
Sar1a	0.04577964	0.94379331	0.375	0.152	1
Dcaf13	0.04643862	0.76309179	0.167	0.03	1
Bclaf1	0.04699054	1.08125305	0.292	0.091	1
Col6a3	0.04701675	1.91500566	0.292	0.121	1
Tmed1	0.04738334	0.93429725	0.167	0.03	1
Ulk2	0.04746619	0.81908136	0.125	0.03	1
Eif4a2	0.04787092	0.93144458	0.5	0.273	1
Pdcd4	0.04789119	-0.9548147	0.25	0.455	1
Klf6	0.04828043	-1.4658525	0.208	0.394	1
Cxcl1	0.04863866	-1.2114772	0.125	0.364	1
Eif4ebp2	0.04905129	0.86968892	0.167	0.03	1
Zhx1	0.04914228	0.95267492	0.167	0.03	1
Chmp3	0.04969578	-1.0057555	0.042	0.152	1
Cep83	0.04974139	0.65664602	0.125	0.03	1
Tecr	0.04989692	-0.8454726	0.042	0.212	1
Psmc5	0.04995466	0.87720364	0.167	0.03	1
Adamts5	0.05005037	1.02926009	0.458	0.273	1
Egfr	0.05005488	1.64767413	0.208	0.091	1
Entpd2	0.05017455	1.73225806	0.167	0.03	1
Kdm5a	0.05032888	1.1556066	0.167	0.061	1
Ier3	0.05048581	-1.0093056	0.125	0.394	1
Echs1	0.05051476	0.96813949	0.208	0.061	1
mt-Nd4l	0.05064987	0.64606149	0.958	0.879	1
Pum1	0.05092485	-0.8012007	0.083	0.303	1
S100a11	0.0510157	-0.4060262	0.667	0.879	1
Sgk1	0.05116896	-1.5440945	0.167	0.364	1
Copg2	0.05129615	0.63294036	0.125	0.03	1
Rpl9	0.05138681	-0.4854697	0.5	0.788	1
Vps13c	0.05148298	0.91987611	0.167	0.061	1
Cdkn1a	0.05167037	-0.9211268	0.167	0.364	1
Eya4	0.05169707	-1.0199633	0.042	0.212	1
Cfap36	0.05172754	0.67807373	0.208	0.03	1
Vegfa	0.05184459	-0.9176897	0.125	0.364	1
Pfn1	0.05190382	-0.6600007	0.375	0.606	1
Col1a1	0.05210611	1.18471835	0.5	0.273	1
Usp14	0.05270032	0.95190504	0.167	0.061	1
Ctsz	0.05309996	1.64622107	0.417	0.242	1
Tacc2	0.05347314	0.51251009	0.167	0.03	1
Afap1	0.05371232	0.65947656	0.167	0.03	1
Eif1b	0.05403718	-1.1483087	0.167	0.333	1
Ewsr1	0.05419375	0.87812472	0.292	0.091	1

Foxp1	0.05443226	0.95221633	0.5	0.273	1
Tmed9	0.05458565	-1.1032948	0.125	0.303	1
Angptl1	0.05475678	-1.0996592	0.042	0.182	1
10-Sep	0.05486114	-0.807331	0.083	0.242	1
Nrf1	0.05521495	1.24473593	0.167	0.03	1
Sptbn1	0.05573103	1.01925661	0.417	0.212	1
Midn	0.0557648	-0.8949592	0.083	0.242	1
Rpl6	0.05580344	-0.4753722	0.542	0.788	1
Gtf2i	0.05618609	1.20227231	0.208	0.061	1
Ube2e1	0.05625726	0.69228092	0.208	0.061	1
Plgrkt	0.05640482	-0.8828343	0.042	0.212	1
Ilf2	0.05658748	-0.9375048	0.042	0.152	1
Zfp326	0.05695211	0.74153327	0.167	0.03	1
Kalrn	0.05706787	-0.8230624	0.042	0.242	1
Bgn	0.05707722	0.61219243	0.958	0.879	1
Bach1	0.0570976	0.85915075	0.292	0.091	1
Ran	0.05717934	-0.848904	0.125	0.273	1
Zcchc7	0.05763154	1.13326481	0.375	0.182	1
Ppp1cc	0.05783658	0.86757961	0.292	0.091	1
Gng5	0.05818403	-0.5440953	0.542	0.758	1
Arf2	0.0584863	-0.933996	0.042	0.152	1
Vim	0.05859858	-1.0703773	0.458	0.636	1
Ywhae	0.05879078	-0.7433832	0.208	0.455	1
Dnajc13	0.05898811	-0.9373971	0.042	0.152	1
Ppp4c	0.05923362	-0.8039968	0.042	0.152	1
Dad1	0.05927133	-0.6486995	0.333	0.576	1
Rab28	0.05969126	0.73093422	0.125	0.03	1
Tmem115	0.05972167	1.11555916	0.167	0.03	1
Gadd45a	0.05978527	-0.8984137	0.042	0.152	1
Dlat	0.05980913	1.03025254	0.125	0.03	1
Emd	0.05986149	-0.7620157	0.042	0.152	1
Ube2j2	0.06001688	-0.7660358	0.042	0.152	1
Ilk	0.06003919	0.90481959	0.292	0.091	1
Sik1	0.06010748	-0.9573222	0.042	0.152	1
Psmc1	0.06020862	1.07110247	0.292	0.121	1
Nudt4	0.06045067	-0.5880532	0.375	0.636	1
Hvcn1	0.06054559	-0.9312584	0.125	0.333	1
Pdgfra	0.06084681	0.78910144	0.667	0.455	1
Fmr1	0.06085201	0.92608834	0.167	0.03	1
Spcs3	0.06120121	-0.8310354	0.042	0.152	1
Eef1b2	0.06121837	-0.6841102	0.292	0.515	1
Zfp52	0.06214188	0.91395685	0.125	0.03	1
Npdc1	0.06218934	1.06801315	0.375	0.182	1

Dpm2	0.06227889	-0.806501	0.042	0.212	1
Sacm1l	0.06242352	-0.7752232	0.042	0.152	1
Ilf3	0.06246242	-0.8184362	0.042	0.182	1
Brd2	0.06253218	-0.8846229	0.208	0.424	1
Sp7	0.0625914	0.80260465	0.167	0.03	1
Soat1	0.06297522	-1.0566841	0.167	0.364	1
Fbf1	0.06334104	0.51353202	0.125	0.03	1
Smndc1	0.06362961	-0.8176288	0.042	0.212	1
Dcbld2	0.06379197	-1.0416063	0.042	0.182	1
Manea	0.06431123	-0.6987998	0.042	0.152	1
Tbc1d20	0.06444249	0.94131096	0.208	0.061	1
Mt1	0.06446639	-1.1914581	0.583	0.758	1
Limd1	0.06448875	1.16447585	0.167	0.03	1
Trappc2l	0.0648015	-0.7510397	0.042	0.212	1
Rbp1	0.06485955	1.16073672	0.25	0.091	1
Rpia	0.0648596	0.58760675	0.125	0.03	1
Serpina3f	0.06486958	-1.2960493	0.125	0.273	1
Rsbn1l	0.06486992	-0.8443324	0.042	0.212	1
Ndufb11	0.06491308	-0.7779679	0.208	0.424	1
Fau	0.0650548	-0.4276078	0.667	0.879	1
Psmc4	0.06524339	-0.6853306	0.042	0.152	1
Efemp1	0.0653206	-1.1329278	0.167	0.303	1
Pja1	0.06540394	0.85020366	0.167	0.03	1
Fam129b	0.0657241	-0.8856922	0.042	0.182	1
Atp6ap2	0.06633746	1.14398472	0.208	0.091	1
Spop	0.06654655	-0.9281525	0.083	0.212	1
Acat1	0.06664338	-0.9428547	0.083	0.212	1
Sptan1	0.06686176	-0.6442315	0.042	0.152	1
Nsun2	0.06693979	0.5031389	0.125	0.03	1
Tmem165	0.06696314	0.95885208	0.292	0.121	1
Tmem59	0.06731791	-0.7637816	0.417	0.636	1
Fam43a	0.06733713	-0.7870775	0.042	0.152	1
Krr1	0.06736409	0.42754666	0.125	0.03	1
Prkca	0.06750575	-0.7569408	0.042	0.152	1
Agtrap	0.06795306	0.47776605	0.125	0.03	1
Pds5a	0.06804443	-0.6677039	0.042	0.212	1
Zmpste24	0.06811073	-0.9314813	0.042	0.182	1
Slc25a36	0.06812538	0.82289391	0.208	0.061	1
Ap1b1	0.06814609	0.72324838	0.167	0.03	1
Fndc1	0.06859476	0.75927072	0.208	0.03	1
Ssna1	0.06955876	-0.7598031	0.042	0.152	1
Safb2	0.06975831	0.81497331	0.167	0.03	1
Hipk1	0.06994724	0.67422042	0.333	0.091	1

Txn2	0.07001375	0.77979255	0.208	0.03	1
Rpl34	0.07022556	-0.654315	0.583	0.818	1
Sin3b	0.07033517	-0.8060145	0.042	0.152	1
Kmt2c	0.07089125	-0.6820404	0.042	0.212	1
Hspb1	0.07100929	-0.8374017	0.083	0.303	1
Ccnl1	0.07119801	-0.7831793	0.208	0.455	1
Cyp1b1	0.07156118	-0.5197512	0.583	0.788	1
Fam160b1	0.07166188	0.93581822	0.167	0.03	1
Supt5	0.07198289	-1.2755635	0.083	0.212	1
Hnrnpll	0.07200739	-0.7969889	0.042	0.152	1
Tmed10	0.07212909	-0.7766968	0.167	0.394	1
Foxo3	0.07260139	-0.7645583	0.042	0.152	1
Limd2	0.07276856	0.68337354	0.167	0.03	1
Zzz3	0.07291342	0.78302381	0.25	0.061	1
Pcdh19	0.07303191	0.92301824	0.167	0.03	1
R3hdm1	0.07319503	0.70659878	0.25	0.061	1
Fmo2	0.07325331	-0.4648788	0.125	0.394	1
Atp5g2	0.07364193	-0.7387692	0.333	0.545	1
Ankfy1	0.07419213	0.89584859	0.167	0.03	1
Gpx3	0.07516485	1.04815497	0.833	0.818	1
Hp	0.07533052	-0.495283	0.75	0.909	1
Rexo2	0.07538726	-0.8921931	0.083	0.212	1
Ostc	0.0755609	-0.5141904	0.208	0.485	1
Prkci	0.07592267	0.55999415	0.208	0.03	1
Pa2g4	0.07605613	0.89595489	0.208	0.061	1
Plekha1	0.07630109	1.43196076	0.167	0.061	1
Mir99ahg	0.07684178	1.47761283	0.167	0.091	1
Cbr3	0.07685557	0.86326516	0.333	0.121	1
Fndc3a	0.07685979	1.07446447	0.292	0.121	1
Mbtps1	0.07689512	0.90103998	0.167	0.03	1
Nupr1	0.07699795	-0.9765693	0.5	0.697	1
Mphosph8	0.07711645	0.99681157	0.208	0.061	1
Gpm6b	0.07719714	-0.8185973	0.208	0.394	1
Psmb5	0.07742779	1.02603506	0.292	0.121	1
Atf7ip	0.07768498	-1.0257165	0.083	0.212	1
Nsfl1c	0.0780148	0.83260616	0.167	0.03	1
Tmem263	0.07814466	1.06306672	0.208	0.061	1
Ilvbl	0.07827518	0.7829339	0.167	0.03	1
Serpina3c	0.07840624	-0.8676733	0.042	0.152	1
S1pr1	0.07854182	1.07270314	0.417	0.242	1
Hdac2	0.07864225	0.87163346	0.167	0.03	1
Hmgb1	0.07916155	-0.9916357	0.417	0.576	1
Bglap	0.07919552	1.56680161	0.208	0.03	1

Mcl1	0.07921997	-1.1428638	0.167	0.303	1
Thoc7	0.07955324	-0.9254663	0.125	0.273	1
Xdh	0.07997516	-1.3458984	0.125	0.303	1
Cox7c	0.08001227	-0.4727731	0.5	0.727	1
Phlda1	0.08039826	-1.2015767	0.083	0.273	1
Supt20	0.08051473	-0.8071559	0.083	0.242	1
Gnptg	0.08052772	0.92781024	0.167	0.061	1
Eif4g2	0.08054637	0.79062057	0.583	0.364	1
Trib1	0.08055568	-1.1241171	0.125	0.333	1
Eif5b	0.08059153	1.00153096	0.208	0.091	1
Wnk1	0.08075008	0.62124946	0.375	0.121	1
Sde2	0.08105551	1.22173392	0.167	0.03	1
0610010K14I	0.08137947	-0.6169444	0.042	0.152	1
Podnl1	0.0814414	-0.770373	0.042	0.182	1
Arpc3	0.08146904	-0.8814542	0.125	0.303	1
Zc3hav1	0.08157988	-0.4077488	0.125	0.364	1
Eea1	0.08162446	0.74228009	0.208	0.061	1
Zfp36l1	0.08176212	-0.5358959	0.542	0.727	1
Serpine1	0.08182183	-1.9567783	0.042	0.152	1
Hint1	0.08194036	-0.5193115	0.417	0.667	1
S100a9	0.08218953	-1.5357257	0.375	0.636	1
Gsn	0.08226764	4.51335277	0.25	0.182	1
Hotairm1	0.08277496	-0.9327911	0.125	0.273	1
Smpd1	0.0828368	0.85688534	0.208	0.061	1
Gm10076	0.0829546	-0.67031	0.625	0.788	1
Sdcbp	0.08309037	-0.692284	0.083	0.273	1
Gabpb2	0.08310174	0.65042826	0.167	0.03	1
Tln2	0.08340108	0.72925244	0.417	0.212	1
Osbpl1a	0.08358037	0.70497855	0.167	0.03	1
Ssb	0.08393559	-0.6945131	0.125	0.364	1
H2afz	0.08407804	-1.0641826	0.333	0.485	1
Smim4	0.08407815	-0.8193475	0.083	0.212	1
Tcf12	0.08439412	0.61960593	0.292	0.091	1
Erbin	0.0845662	0.71365823	0.208	0.061	1
Rai14	0.0850409	-0.8645669	0.125	0.303	1
Ptma	0.08515276	-0.6391675	0.75	0.879	1
Srek1	0.08574975	0.94100031	0.25	0.091	1
Ptger4	0.08591899	-0.673924	0.083	0.242	1
Lamp2	0.08683562	-0.7186129	0.25	0.424	1
Rrad	0.08712963	-0.6511513	0.042	0.212	1
Aurkaip1	0.08733898	-0.9197626	0.083	0.212	1
Gpc6	0.08762149	-0.7170756	0.083	0.273	1
Fkbp7	0.08766529	-0.8754964	0.208	0.364	1

Atp6v1e1	0.08768129	-0.7812495	0.167	0.364	1
Usp48	0.08783902	-0.7240339	0.042	0.152	1
Lypla2	0.08792742	0.87486354	0.167	0.03	1
Cops9	0.08875643	-0.8263849	0.25	0.455	1
Atp5j	0.08891189	-0.5904182	0.292	0.515	1
Eif4g3	0.08905636	-0.6482892	0.042	0.212	1
Cirbp	0.08926123	0.96621816	0.333	0.152	1
Crip1	0.08936335	1.23410852	0.667	0.485	1
P2rx2	0.0896278	0.70151962	0.167	0.03	1
Tmem140	0.09011044	0.71258756	0.25	0.061	1
Ggt5	0.09119434	0.81910146	0.333	0.152	1
Cnih4	0.0913922	0.82193448	0.208	0.061	1
Sgta	0.09163041	1.09655864	0.25	0.091	1
Prrx2	0.09168875	1.02378074	0.125	0.03	1
Fndc3b	0.09202492	0.73568123	0.5	0.303	1
Pdgfrb	0.09215372	-0.4276957	0.5	0.667	1
Zfp521	0.09252825	0.77950537	0.125	0.03	1
Igfbp5	0.09259497	-0.5588251	0.75	0.909	1
Shc1	0.09262988	-0.5882901	0.042	0.152	1
D5Ertd579e	0.09267738	0.82127013	0.125	0.03	1
Slc26a7	0.0927269	-1.0442903	0.25	0.424	1
Ganab	0.09296517	1.14418162	0.208	0.091	1
Surf4	0.0934246	-0.6204044	0.167	0.333	1
Gas5	0.09349814	-0.7262784	0.292	0.515	1
Cstb	0.09377745	-0.4946592	0.333	0.545	1
Ppfibp1	0.09408682	0.74769261	0.25	0.091	1
Vcan	0.09414554	0.79763659	0.125	0.03	1
Zcchc14	0.09435653	-0.6410445	0.208	0.424	1
Snhg18	0.09476949	0.88485445	0.458	0.273	1
Enpp2	0.09486927	0.92179881	0.25	0.091	1
Ccdc47	0.09579297	-0.8474248	0.042	0.121	1
Atp6v0a1	0.09604396	0.65465464	0.25	0.061	1
Cbr1	0.09656392	0.61746981	0.167	0.03	1
Ssbp2	0.096635	-0.6330921	0.042	0.152	1
Sp3	0.09666565	-0.7576719	0.083	0.242	1
Ank2	0.0969185	0.84773573	0.125	0.03	1
Mtss2	0.09713016	0.65977911	0.167	0.03	1
Dnajb1	0.09727472	-1.2976003	0.042	0.152	1
Znrf1	0.0976748	-0.9154277	0.083	0.242	1
Gadd45b	0.09819823	-1.2670533	0.292	0.455	1
Tra2a	0.09820663	-0.7895807	0.125	0.303	1
Ubtd2	0.09858699	-0.8140677	0.042	0.121	1
Trim30a	0.098671	0.95921993	0.167	0.03	1

Sgsh	0.09891243	0.82573099	0.125	0.03	1
Ndufa4	0.09928591	-0.7910114	0.5	0.636	1
Bola1	0.0996537	0.75205293	0.167	0.03	1
Usp16	0.10001689	-0.9840267	0.042	0.152	1
Nr4a1	0.10010248	-0.6979013	0.167	0.394	1
Btbd7	0.10027744	0.93180168	0.417	0.273	1
Ndufb8	0.10028187	-0.5880539	0.125	0.303	1
Mcrip1	0.10039453	-0.9053321	0.042	0.121	1
Dnaja1	0.10063949	-0.7431863	0.292	0.485	1
Irf1	0.10073381	-0.7893455	0.25	0.455	1
Atp6v1c1	0.10086611	0.77255873	0.125	0.03	1
Cyp4v3	0.10088285	0.6370138	0.125	0.03	1
Rnpc3	0.10098181	0.99981156	0.125	0.03	1
Rbm39	0.10115688	-0.5931464	0.458	0.667	1
Ppia	0.10122965	-0.511928	0.708	0.848	1
Ccny	0.1013452	0.68431307	0.208	0.061	1
Gng10	0.10172228	-0.610518	0.042	0.182	1
Nenf	0.10217503	-0.8987995	0.417	0.576	1
Dguok	0.10260496	-0.811889	0.042	0.182	1
Plod3	0.10265283	-0.7580882	0.042	0.182	1
Acyp2	0.10270827	-0.7066396	0.083	0.212	1
Magt1	0.10332265	0.79627387	0.25	0.091	1
Uqcrq	0.10363316	-0.8356987	0.25	0.424	1
Ylpm1	0.10373336	-0.7319024	0.042	0.121	1
Pdgfd	0.10403078	0.88203092	0.125	0.03	1
Zmiz1	0.10435361	-0.663664	0.125	0.273	1
U2af2	0.10446369	-0.6184676	0.042	0.182	1
Snai2	0.10448425	-0.9485762	0.208	0.364	1
Rhoc	0.10459246	0.8843117	0.333	0.152	1
Ost4	0.10487197	-0.4878049	0.25	0.455	1
Gng2	0.10496025	1.04282875	0.125	0.03	1
Eif2b5	0.10499641	0.93266075	0.125	0.03	1
Psmd1	0.10500258	0.44503358	0.167	0.03	1
Dpysl2	0.1050094	-1.0317599	0.083	0.212	1
Errfi1	0.10507655	-1.0329021	0.542	0.697	1
Bcap31	0.10541316	-0.8632585	0.167	0.303	1
Hdac7	0.10564748	0.66528109	0.167	0.03	1
Pex3	0.10592945	-0.8651195	0.083	0.212	1
Akt1	0.10614127	0.54929197	0.125	0.03	1
Map1lc3b	0.10624914	-0.7756531	0.417	0.545	1
Emb	0.1063974	0.842601	0.292	0.152	1
Cop1	0.1066807	0.64114496	0.208	0.061	1
Snu13	0.10672792	-1.094425	0.167	0.364	1

Bmp1	0.10705673	0.70084301	0.375	0.182	1
Mpv17	0.10710666	0.65426835	0.167	0.061	1
Col8a1	0.10781887	1.54193214	0.417	0.333	1
Cct4	0.10813139	0.71738714	0.25	0.091	1
Trp53	0.10822767	0.63057521	0.167	0.03	1
Rpl23a	0.10840964	0.99119455	0.5	0.333	1
Arrdc3	0.10856489	-0.9686901	0.042	0.182	1
Zfp706	0.10859198	-0.8073161	0.125	0.273	1
Paics	0.10879991	-0.4598255	0.042	0.152	1
Abl1	0.10880487	0.78060113	0.125	0.03	1
S100a6	0.10885333	1.52890761	0.625	0.576	1
mt-Nd2	0.10926975	1.09568886	0.75	0.636	1
Ngp	0.10931057	0.87074377	0.25	0.121	1
Chchd2	0.10984066	-0.6529689	0.583	0.758	1
Vdac1	0.11009011	0.72281964	0.167	0.061	1
Pik3ca	0.11023323	0.51198926	0.25	0.091	1
Slc40a1	0.11036959	0.7925667	0.167	0.061	1
Nosip	0.11047693	0.71958725	0.125	0.03	1
Derl2	0.11053843	-0.7322798	0.042	0.182	1
Gng11	0.11087828	-0.7113159	0.167	0.364	1
Epb41l2	0.11106527	-0.9918566	0.167	0.273	1
Tma7	0.1113512	-0.3233109	0.25	0.515	1
Akap11	0.11145673	-0.7839751	0.042	0.121	1
Fgf7	0.11167931	-0.9675302	0.167	0.333	1
Ddx1	0.1119269	-0.6733054	0.042	0.121	1
Cited2	0.11228813	-1.0437476	0.083	0.212	1
Sema5a	0.11235997	0.74511366	0.333	0.152	1
Skap2	0.11249796	1.03845106	0.125	0.061	1
Bcl2l11	0.11280638	-0.7417356	0.042	0.121	1
Mrps28	0.11347854	-0.7589766	0.083	0.212	1
Smim10l1	0.11357529	-0.7179892	0.042	0.121	1
Phb	0.11381022	-0.6219585	0.042	0.121	1
Pltp	0.11388055	-0.7140773	0.125	0.303	1
Emc7	0.11411656	-0.8272356	0.167	0.333	1
Peli1	0.11448563	-0.7349403	0.083	0.273	1
Slc25a3	0.11465635	0.80502165	0.458	0.303	1
Zmat2	0.11466801	-0.6129749	0.042	0.121	1
Kit	0.11474906	-1.128816	0.042	0.152	1
Dpm1	0.11495229	-0.7725639	0.125	0.273	1
Lasp1	0.11503621	0.73245204	0.25	0.121	1
Cacybp	0.11515229	0.54443594	0.167	0.03	1
Actr3	0.11589534	-0.8447012	0.083	0.152	1
Zbtb7a	0.11590885	-0.6445736	0.083	0.242	1

Brd7	0.11600482	0.77340532	0.208	0.061	1
Tiparp	0.11600677	-0.839233	0.083	0.242	1
Tcn2	0.11602028	-0.7864482	0.083	0.212	1
Rabgap1l	0.11626569	0.53799152	0.125	0.03	1
Rab1b	0.11631064	-0.6232823	0.042	0.182	1
Dennd1b	0.11667237	0.72231563	0.167	0.061	1
Tpm4	0.11681317	1.00372247	0.333	0.212	1
Golt1b	0.11744213	0.69986288	0.208	0.091	1
Id3	0.11762536	-1.2302926	0.708	0.758	1
4930402H24	0.11804768	0.68164422	0.125	0.03	1
Wipf1	0.11824522	0.75501518	0.208	0.061	1
Baz1a	0.11835902	-0.6546392	0.042	0.152	1
Cdk2ap2	0.11854326	-0.6914085	0.083	0.212	1
Nampt	0.11902154	0.76317206	0.25	0.091	1
Gpbp1	0.11917269	-0.7539296	0.083	0.242	1
Txn1	0.11944296	-1.106234	0.417	0.636	1
Thap2	0.12040296	-0.700408	0.042	0.182	1
Kat6b	0.12055718	1.12903583	0.125	0.061	1
Gm42418	0.12062277	1.33837891	1	0.97	1
Fdxr	0.12064165	0.48430829	0.167	0.03	1
Serpinf1	0.12064581	1.03751693	0.333	0.212	1
Ikbip	0.12068563	0.93325313	0.333	0.182	1
Ccn1	0.12109664	-1.0632041	0.375	0.545	1
Ism1	0.12154992	-0.4706437	0.125	0.333	1
Lum	0.12177751	1.14876988	0.25	0.091	1
Timm10	0.12177904	0.94175867	0.125	0.03	1
Erp29	0.12196889	-0.8108391	0.125	0.273	1
Tmem234	0.12198329	-0.7666793	0.083	0.242	1
Nisch	0.12314023	0.89345529	0.375	0.212	1
Cdc73	0.12320414	0.79336842	0.292	0.121	1
Huwe1	0.12326326	0.94926998	0.417	0.273	1
Apod	0.12335689	1.1068884	0.333	0.152	1
Trf	0.12356735	-0.4390363	0.542	0.788	1
Tmem63a	0.12368968	-0.5287803	0.042	0.121	1
Cd82	0.12391191	0.46745277	0.167	0.03	1
Commd7	0.12392024	0.91265446	0.167	0.061	1
Lima1	0.12399453	0.4317888	0.25	0.061	1
Esd	0.124011	-0.7502592	0.125	0.273	1
Dtymk	0.12404912	-0.5642667	0.042	0.121	1
Nipbl	0.12428723	0.71117834	0.25	0.091	1
Stag1	0.12460963	-0.7978583	0.083	0.182	1
Xrn1	0.12467311	-0.9158634	0.083	0.182	1
Wdr26	0.12475354	-0.6458021	0.042	0.121	1

Smtn	0.12487524	1.20837364	0.208	0.091	1
Dock7	0.12507607	0.71821143	0.125	0.03	1
Cux1	0.12529702	0.85677869	0.167	0.061	1
Lyst	0.12538159	0.69271519	0.125	0.03	1
Strn	0.12559425	-0.7736556	0.042	0.152	1
Slc25a11	0.12608147	0.8353357	0.125	0.03	1
Cct2	0.12612989	0.54332001	0.125	0.03	1
Qk	0.12643899	1.27941013	0.458	0.303	1
Glrx5	0.12661357	-0.6096026	0.042	0.152	1
Rpl17	0.1266953	0.81662175	0.625	0.455	1
Bcl9l	0.12677448	0.87331678	0.25	0.152	1
Rheb	0.12686521	-0.5311296	0.042	0.182	1
Adar	0.12692522	0.53048353	0.208	0.061	1
Hist1h1c	0.12699639	0.51008686	0.208	0.061	1
Anapc1	0.12717269	0.83010881	0.125	0.03	1
Arhgap6	0.12734298	0.94712162	0.25	0.121	1
Cfl1	0.12747305	-0.7442074	0.333	0.485	1
Ccar1	0.12785596	0.72424361	0.292	0.121	1
Lsm5	0.12812763	-0.7836915	0.167	0.303	1
Sox4	0.12821305	-1.033416	0.167	0.303	1
Yaf2	0.12867175	0.70002339	0.125	0.03	1
Strn3	0.12890542	-0.8070305	0.083	0.182	1
Hprt	0.12905281	-0.5532961	0.042	0.121	1
Zfp664	0.1293453	0.58503817	0.208	0.061	1
Pcolce	0.12992524	-0.6730414	0.208	0.424	1
Plekhh3	0.13001998	0.91252891	0.167	0.061	1
Npm3	0.13022437	-0.5637524	0.042	0.121	1
Gnai3	0.13024226	-0.7862775	0.042	0.152	1
Arap1	0.13028201	0.98957501	0.167	0.061	1
Gabarapl2	0.13041695	-0.6851254	0.125	0.273	1
Ltbp2	0.13056939	1.36635989	0.375	0.273	1
Fuom	0.13103126	0.98694015	0.125	0.03	1
Stt3a	0.13103962	0.79533063	0.167	0.061	1
Polr2h	0.13111658	-0.5885256	0.042	0.121	1
Ube2q2	0.13131582	-0.9528945	0.083	0.182	1
Mtch2	0.13149032	0.70214298	0.125	0.03	1
Egr2	0.13155574	-0.63267	0.042	0.121	1
Psmd13	0.13206676	0.69553124	0.167	0.061	1
Bri3	0.13228262	-0.8114798	0.125	0.242	1
Acot8	0.13247116	0.86739109	0.125	0.03	1
Myd88	0.13256672	-0.4587922	0.042	0.182	1
Chl1	0.13269111	0.64232129	0.167	0.061	1
M6pr	0.13283136	0.66149057	0.25	0.121	1

Cdc16	0.13313831	-0.5755155	0.042	0.121	1
Rbbp4	0.1336938	-0.6108239	0.125	0.273	1
Calm1	0.13382761	-0.3948891	0.417	0.606	1
Erf	0.13395908	-0.5894299	0.042	0.121	1
Csrnp1	0.13410813	-0.7605256	0.042	0.152	1
Thoc2	0.13417786	0.45944839	0.167	0.03	1
Naaa	0.13462966	0.56967796	0.167	0.03	1
Atp5g3	0.13504199	0.74396348	0.333	0.182	1
Fam120b	0.1353234	0.61601057	0.167	0.061	1
Cflar	0.13539781	0.74475795	0.25	0.121	1
Ssrp1	0.1357774	0.88462755	0.125	0.03	1
Dnajc5	0.13596391	-0.8707781	0.083	0.182	1
E330013P04I	0.13651469	0.6636687	0.125	0.03	1
Ebf1	0.13671337	-0.265705	0.583	0.788	1
Smarcc2	0.1368659	0.73063032	0.25	0.091	1
mt-Nd1	0.13709034	0.74671956	0.75	0.667	1
Arpc1a	0.13715463	1.11551181	0.167	0.091	1
Cplane1	0.13721242	-0.459744	0.042	0.212	1
Tpra1	0.13733591	0.95279159	0.125	0.03	1
Psme4	0.13736963	-0.8417865	0.083	0.212	1
Ppp2r2d	0.13762963	-0.4203996	0.042	0.121	1
Emc3	0.13800164	0.85316684	0.208	0.091	1
Rab11b	0.13831983	0.95160824	0.25	0.091	1
Xrcc4	0.13833794	0.62898188	0.125	0.03	1
Minpp1	0.13857437	0.60126811	0.125	0.03	1
Trappc13	0.13887409	-0.8739712	0.042	0.152	1
Olfml3	0.13892365	0.6797164	0.542	0.364	1
Slit2	0.13894714	0.69139925	0.208	0.061	1
Chka	0.13940002	-0.7567041	0.042	0.152	1
Tmem176b	0.13947942	-0.3542576	0.833	0.939	1
Mbtd1	0.13966534	0.579571	0.208	0.061	1
Gtf2a2	0.13966814	-0.4462965	0.042	0.121	1
Pigyl	0.13976837	-0.6202339	0.042	0.121	1
Cox6c	0.13985087	-0.550375	0.625	0.758	1
Lsm6	0.13998864	-0.733515	0.042	0.182	1
Dync1li1	0.14003514	0.79108743	0.208	0.061	1
Bet1l	0.14006258	0.5555657	0.125	0.03	1
St13	0.14019065	0.71840196	0.417	0.242	1
Nsd3	0.14062232	-0.9170623	0.208	0.303	1
Resf1	0.14071354	-0.7499535	0.083	0.212	1
Mxra8	0.14091155	0.90499724	0.375	0.212	1
Trp53bp2	0.14100502	-1.1297971	0.042	0.121	1
Nrp2	0.14197899	0.91591548	0.333	0.182	1

N4bp2	0.14197997	-0.9729464	0.042	0.121	1
Ibsp	0.14215153	-0.7383764	0.625	0.788	1
Ivns1abp	0.14223767	-0.8672616	0.083	0.152	1
CAAA011183	0.14287999	-0.8467484	0.042	0.121	1
Prdx6	0.14290051	-0.5013376	0.042	0.121	1
Bcl2	0.14293742	0.86272012	0.167	0.061	1
St3gal1	0.14296528	-0.7404943	0.083	0.212	1
Aak1	0.14300006	1.04008132	0.125	0.03	1
Cope	0.14302122	-0.5796302	0.125	0.273	1
Csnk1a1	0.14308971	-0.6182658	0.375	0.545	1
Cdipt	0.14313134	-0.7283399	0.042	0.152	1
Nemf	0.14333908	-0.6732578	0.042	0.121	1
Mrpl57	0.14366898	0.76549525	0.25	0.121	1
Eml4	0.14377664	0.59126669	0.208	0.061	1
Hnrnpl	0.1439098	-0.5777259	0.167	0.333	1
Plod2	0.14443556	1.02299187	0.167	0.061	1
Ncoa7	0.1445973	0.90669327	0.208	0.091	1
Sult1a1	0.14472243	0.50924731	0.125	0.03	1
Arhgef11	0.14472586	-0.5276726	0.042	0.182	1
Mapkapk2	0.14497166	-0.5370105	0.042	0.152	1
Canx	0.14510071	0.79988621	0.458	0.303	1
Bmp5	0.14516601	0.58094319	0.167	0.061	1
Sh3d19	0.14553799	-0.5271737	0.042	0.121	1
Pim1	0.1455799	-0.8559935	0.208	0.394	1
Cript	0.14562666	-0.6796247	0.042	0.182	1
Zfp260	0.14563416	-0.9106543	0.083	0.182	1
Tspan6	0.14593844	-0.5504174	0.042	0.182	1
Pfkp	0.14602666	0.52776618	0.125	0.03	1
Papola	0.14612503	0.96298391	0.25	0.121	1
Usp34	0.14629521	-0.6500496	0.083	0.212	1
Cry2	0.14632298	-0.6394111	0.042	0.152	1
Cavin3	0.14675338	1.01012321	0.25	0.121	1
Shisa4	0.14677717	0.83727258	0.125	0.03	1
Akap1	0.14682645	0.87927844	0.125	0.03	1
Mphosph10	0.14683591	0.67362674	0.167	0.061	1
Fam3c	0.14687001	0.80943435	0.125	0.061	1
Tmem184b	0.14712194	0.86874656	0.125	0.03	1
Cct8	0.14750615	0.7957118	0.125	0.03	1
Eif4ebp1	0.14752802	-0.6085865	0.083	0.212	1
Ids	0.1476622	-0.7189088	0.042	0.182	1
Ppt2	0.14778861	0.47338831	0.125	0.03	1
Cebpd	0.14821839	-1.343625	0.333	0.485	1
Impad1	0.14851025	-0.9365923	0.167	0.303	1

Btg1	0.14858665	-0.4099479	0.375	0.576	1
Hmgcll1	0.14946024	0.53142094	0.167	0.061	1
Zc3h7a	0.1494701	-0.6483164	0.042	0.152	1
Nptn	0.14975479	-0.3231171	0.375	0.576	1
Smyd2	0.14995563	0.58428178	0.167	0.03	1
Jdp2	0.14996003	-0.6106598	0.125	0.242	1
Rab3gap1	0.14997116	-0.5749446	0.042	0.121	1
Timm29	0.14999969	0.66061467	0.125	0.03	1
Ifnar2	0.15007112	-0.5764643	0.333	0.515	1
Tns1	0.15034283	0.806259	0.125	0.03	1
Klf7	0.15065753	0.60868677	0.125	0.061	1
Rnf19a	0.15075038	-0.6717856	0.083	0.152	1
Calu	0.15086355	-1.0241205	0.208	0.333	1
Ndufb6	0.15091339	0.84205923	0.25	0.121	1
Atp5f1	0.15105275	-0.4468371	0.25	0.455	1
Eny2	0.1510865	0.9815583	0.333	0.212	1
Pnn	0.15120677	0.98499566	0.125	0.061	1
Fbn1	0.15182784	1.12093075	0.5	0.364	1
Acsl5	0.15197258	0.40973876	0.167	0.03	1
Ccl9	0.15217895	0.70189703	0.125	0.03	1
Ppp2r5a	0.15250686	-0.8897694	0.125	0.212	1
7-Mar	0.15268339	-0.5200995	0.042	0.182	1
Samhd1	0.1529399	0.45382449	0.542	0.333	1
Vps36	0.15302196	0.37457007	0.208	0.061	1
Etv6	0.1530554	0.44364534	0.167	0.061	1
Sos1	0.15317803	-0.6330352	0.042	0.121	1
Pacs2	0.1531921	0.77656334	0.125	0.061	1
Ptpn1	0.15348519	-0.8756775	0.083	0.182	1
Hsdl2	0.153609	-0.542844	0.042	0.121	1
Vezt	0.1541552	-0.7341978	0.125	0.273	1
Sdk2	0.154218	0.6823188	0.208	0.091	1
Clk4	0.15484791	-0.8542853	0.125	0.242	1
Cul3	0.15525109	-0.9009489	0.125	0.242	1
Kxd1	0.15538573	0.42614922	0.125	0.03	1
Fem1c	0.15546592	1.00455329	0.125	0.061	1
Rab18	0.15644832	-0.4575383	0.125	0.303	1
Mme	0.1566941	0.79254986	0.5	0.364	1
Epb41l4aos	0.15673004	0.41565218	0.125	0.03	1
Hist2h2aa1	0.15693673	0.4624335	0.125	0.03	1
Pdhb	0.15707028	-0.5999804	0.042	0.152	1
Brd4	0.15709528	-0.6346801	0.042	0.152	1
Cct5	0.1578413	0.78678122	0.208	0.091	1
Ctdsp2	0.15794016	0.69218565	0.375	0.212	1

Rnf10	0.15815758	0.95322886	0.292	0.152	1
Rbm27	0.15829195	-0.7152028	0.083	0.152	1
Snapin	0.15832761	0.74728032	0.125	0.03	1
Uso1	0.15858651	0.78542895	0.125	0.03	1
Cops5	0.15868797	0.73895012	0.167	0.061	1
Sod2	0.15880877	-0.7323708	0.25	0.424	1
Il17d	0.15892312	0.60116256	0.167	0.061	1
Fbxo22	0.15893819	0.40372015	0.125	0.03	1
Birc6	0.15900475	-0.8080349	0.25	0.364	1
Hipk3	0.15945548	0.84550269	0.167	0.061	1
Prpf18	0.15946174	0.40513241	0.125	0.03	1
Hmgb2	0.15955669	-0.6630918	0.125	0.303	1
Vgll4	0.15983541	0.48477965	0.292	0.091	1
Pon2	0.16006745	-0.9317944	0.167	0.273	1
Dynlrb1	0.16023162	-0.8302807	0.125	0.242	1
B3gat3	0.16064511	0.87953176	0.125	0.03	1
Itpripl2	0.16064757	0.61456885	0.25	0.121	1
Tlr4	0.16067548	0.6702807	0.125	0.03	1
Grem1	0.16068799	-0.6817885	0.208	0.394	1
Tap2	0.16078542	0.7697471	0.25	0.121	1
Nfu1	0.16085636	-0.5478356	0.042	0.121	1
9530082P21I	0.16092135	0.60092035	0.125	0.03	1
Faf2	0.16153471	1.04295724	0.208	0.121	1
Ldah	0.16186811	0.6595661	0.125	0.03	1
Mark3	0.16199927	-0.9319147	0.042	0.121	1
Tmem39a	0.16230318	0.49761985	0.125	0.03	1
Rnf4	0.16324127	0.42086582	0.125	0.03	1
H2-T23	0.16340156	-0.6911535	0.25	0.394	1
Polr3a	0.16356044	0.61676308	0.125	0.03	1
Mafk	0.16423871	-0.762386	0.042	0.121	1
Sf3b2	0.16511858	-0.7672781	0.125	0.242	1
Skil	0.16515439	-0.4554169	0.125	0.273	1
Dmxl1	0.16533219	-0.6607632	0.042	0.152	1
Ndufs3	0.16539575	-0.8956832	0.083	0.182	1
Zeb2	0.1654902	0.71679761	0.583	0.485	1
Ist1	0.16564165	0.78116325	0.167	0.061	1
Mmd	0.16594978	1.02257222	0.167	0.061	1
Rnf41	0.166002	0.7792915	0.125	0.03	1
Myl12b	0.16616551	-0.559321	0.25	0.424	1
Zfp26	0.16629402	0.74293402	0.125	0.03	1
Lrpap1	0.16643094	-0.501062	0.208	0.364	1
Max	0.16662538	-0.5618679	0.042	0.152	1
Sema7a	0.16690705	0.82829216	0.25	0.121	1

Cmya5	0.16778216	0.77779584	0.125	0.03	1
Nfkbib	0.16778947	0.3639797	0.125	0.03	1
Map3k2	0.16786618	0.53725314	0.25	0.091	1
2610002M06	0.16788863	0.72526618	0.125	0.061	1
Rpl26	0.16791598	-0.2648568	0.5	0.727	1
Suclg1	0.16808742	0.86580886	0.125	0.061	1
Ssr4	0.16835618	-0.43099	0.292	0.485	1
Rps6	0.1685862	-0.4483563	0.292	0.485	1
Arl2bp	0.16951897	0.79428584	0.167	0.061	1
Hspg2	0.16977571	1.08838779	0.292	0.152	1
Rbms1	0.16988748	-0.2668125	0.417	0.667	1
Nt5c	0.16994902	0.92110893	0.167	0.061	1
Hbs1l	0.17036058	-0.6382916	0.042	0.121	1
Atp6v0e	0.1705307	-0.2907719	0.208	0.424	1
Serpina3n	0.17070827	-0.7232076	0.167	0.303	1
Gabarap	0.17119034	-0.5585253	0.292	0.455	1
Uty	0.17144582	0.53243206	0.167	0.061	1
Hdlbp	0.17193775	0.55963488	0.458	0.242	1
Enah	0.17222399	0.7025268	0.292	0.152	1
P3h3	0.17229779	-0.7412358	0.083	0.212	1
Atp6v0b	0.17230694	1.00717289	0.292	0.212	1
Dicer1	0.17271645	0.49128116	0.125	0.03	1
Nt5c2	0.17310399	0.95223813	0.167	0.061	1
Pdcd10	0.17318216	0.40429567	0.167	0.061	1
Mgst1	0.17407015	-0.8178479	0.25	0.394	1
Mmp13	0.1744931	1.45167597	0.417	0.303	1
Zfp740	0.17565538	0.71201685	0.125	0.061	1
Rpl22l1	0.1758165	-0.4772246	0.458	0.636	1
Rab6a	0.17617203	0.55260429	0.25	0.091	1
Fam49b	0.17627944	-0.3540544	0.042	0.121	1
Szrd1	0.17636275	0.55946761	0.125	0.03	1
Hmgn2	0.17679536	-0.3384288	0.167	0.364	1
Creld2	0.17704117	-0.6098045	0.083	0.182	1
Cpeb2	0.17707004	0.62631588	0.125	0.061	1
Rbm42	0.17732538	0.52032917	0.25	0.091	1
Cyb5r3	0.17744531	0.54766414	0.125	0.03	1
Eif2s3x	0.1774942	0.61724211	0.167	0.061	1
Timp2	0.17757128	0.70316776	0.5	0.364	1
Ahctf1	0.17757659	0.75008048	0.167	0.091	1
Tomm22	0.17758606	0.62114241	0.125	0.03	1
Sertad1	0.17784695	-0.7497192	0.083	0.182	1
Abcc1	0.17796467	1.01157605	0.125	0.03	1
Golga3	0.1780716	0.58255175	0.125	0.03	1

Dynlt1f	0.17838794	-0.4624137	0.042	0.182	1
Ddx54	0.17870532	0.48035043	0.167	0.061	1
Luc7l	0.17885223	-0.8703481	0.083	0.152	1
Hlx	0.17892309	0.8146543	0.125	0.03	1
Lyrm2	0.17896578	0.77260779	0.125	0.03	1
Runx3	0.17918737	-0.4518091	0.042	0.182	1
Spry2	0.17937489	-0.8409801	0.083	0.212	1
Ugcg	0.17941799	-0.7394998	0.125	0.212	1
Slc25a17	0.17952623	0.9053681	0.208	0.121	1
Rps6ka3	0.17981266	0.68683781	0.167	0.061	1
Socs3	0.18039771	-0.9821284	0.333	0.485	1
Eid1	0.1804536	-0.7149556	0.167	0.303	1
Trir	0.18068079	-0.6287112	0.125	0.242	1
Gcn1	0.18079467	0.41944699	0.167	0.061	1
Slc39a1	0.18110592	-0.358785	0.333	0.515	1
Tob2	0.18127066	-0.6456038	0.083	0.212	1
Fosl2	0.18133558	-0.9860953	0.083	0.182	1
Mtch1	0.18147839	0.74486473	0.333	0.182	1
Snrpc	0.18162598	-0.5706434	0.042	0.152	1
Akr1c12	0.18166336	0.67144892	0.125	0.061	1
Rplp1	0.18190266	0.63264761	0.875	0.788	1
Clint1	0.18190515	-0.4717554	0.083	0.212	1
Kank2	0.1819552	0.75135822	0.167	0.091	1
Pdia4	0.18202434	0.88012884	0.417	0.273	1
Mt2	0.18228059	-0.5291957	0.417	0.576	1
Tnks1bp1	0.18253003	-0.4306428	0.042	0.121	1
D8Ertd738e	0.18254288	-0.8056639	0.25	0.394	1
G3bp1	0.1826292	0.69313295	0.208	0.091	1
Tmem159	0.1826837	-0.5863448	0.042	0.152	1
Nr4a2	0.18329693	1.04098137	0.125	0.061	1
Ndufa11	0.18340019	-0.7467632	0.167	0.273	1
Mfap2	0.18389107	0.5053699	0.125	0.03	1
Etfb	0.1841015	-0.4886212	0.167	0.303	1
Fgfrl1	0.18421262	0.82835169	0.208	0.091	1
Chp1	0.18431832	-0.6526321	0.083	0.182	1
Ier2	0.18462347	-1.031316	0.167	0.273	1
Irgm2	0.18484364	0.52914366	0.167	0.061	1
Phpt1	0.18494851	0.4648747	0.125	0.03	1
Eef2	0.18534186	0.82933199	0.292	0.152	1
Hspa4	0.1854321	0.8779028	0.167	0.061	1
Seh1l	0.18559873	0.70295958	0.125	0.061	1
Mpzl1	0.18582081	0.53095014	0.292	0.152	1
Tollip	0.18597264	0.45052137	0.125	0.061	1

Slc35e4	0.18672606	-0.6310259	0.042	0.121	1
Hax1	0.18690946	0.75471406	0.167	0.061	1
Plekhj1	0.18735341	-0.8684001	0.042	0.121	1
Guf1	0.18835601	0.70505946	0.167	0.061	1
Rps10	0.18864248	-0.435694	0.708	0.848	1
Fhl1	0.18868587	0.68615868	0.125	0.03	1
Tm9sf4	0.18884035	-0.5474901	0.042	0.152	1
Ncoa1	0.18898642	-0.4623449	0.042	0.152	1
Ddost	0.18900343	-0.5219004	0.167	0.303	1
Nktr	0.18998302	-0.6030908	0.167	0.303	1
Sfr1	0.18999841	-0.4325053	0.25	0.394	1
Dcun1d1	0.1903406	0.38192829	0.167	0.03	1
Bmpr1a	0.19057379	-0.6234176	0.042	0.121	1
Sec24d	0.19124083	0.81563282	0.167	0.061	1
Anxa2	0.19126001	-0.5827989	0.208	0.394	1
Timp3	0.19146315	0.85703187	0.208	0.091	1
Tmbim1	0.19193617	0.76021684	0.125	0.03	1
Cbx3	0.19194719	-0.6636797	0.25	0.424	1
Frg1	0.19212152	-0.5918136	0.042	0.152	1
Ostf1	0.19212843	-0.5927051	0.083	0.182	1
Nbl1	0.19276552	0.91334414	0.125	0.03	1
Snrnp48	0.19281237	0.9157404	0.167	0.061	1
Zdhhc21	0.1929603	-0.5334462	0.083	0.182	1
Sdf2l1	0.19314028	-0.5204087	0.042	0.121	1
Loxl3	0.19319198	-0.7226371	0.208	0.333	1
mt-Co3	0.19330708	0.82793991	0.917	0.939	1
Mef2c	0.19333452	0.36959998	0.208	0.091	1
Polr2i	0.1935611	0.71095092	0.25	0.121	1
Msl3	0.19403025	-0.768171	0.083	0.182	1
Prkag1	0.19411907	0.54651128	0.125	0.03	1
Negr1	0.19451615	-0.7289165	0.042	0.152	1
Irf3	0.1946728	0.47129705	0.125	0.03	1
Eif4b	0.19472179	0.87020872	0.208	0.091	1
Notch3	0.1950086	-0.5171208	0.042	0.152	1
Smurf1	0.19503612	1.13273557	0.125	0.03	1
Mob1a	0.19514275	-0.6586687	0.042	0.152	1
Prkar1a	0.19541002	-0.5808609	0.333	0.455	1
Nus1	0.19546082	0.59009132	0.208	0.091	1
Prtn3	0.19570996	-1.316145	0.042	0.121	1
Ldha	0.19599918	-0.4249371	0.208	0.394	1
Gpatch8	0.19601119	-0.5212159	0.042	0.152	1
Fbh1	0.19603302	0.37941433	0.125	0.03	1
Adprh	0.19624674	-0.5918717	0.042	0.121	1

Kmt5b	0.19626969	0.78042292	0.167	0.091	1
Sertad2	0.19633794	-0.6957635	0.083	0.182	1
Kirrel	0.19660784	0.39676398	0.292	0.121	1
Rgs2	0.19672792	-0.5421082	0.083	0.212	1
Kctd12	0.19697017	-0.4175275	0.333	0.545	1
Npepps	0.19704703	0.96984784	0.125	0.061	1
Rasl11a	0.19712398	-1.0200653	0.042	0.121	1
Ccdc34	0.19723803	0.71195307	0.167	0.091	1
Nfx1	0.19740702	0.36012513	0.208	0.061	1
Swi5	0.19745147	-0.7648558	0.167	0.303	1
Net1	0.19756997	0.66678574	0.125	0.03	1
Zfhx4	0.19762419	0.38875608	0.542	0.364	1
Myl9	0.1976945	0.59580927	0.25	0.121	1
Rpl41	0.19783439	-0.2640635	0.792	0.909	1
Timmdc1	0.19882301	0.41372192	0.125	0.03	1
Znrd2	0.19889904	1.29895397	0.167	0.091	1
Rxylt1	0.19892731	-0.4759741	0.083	0.212	1
Mettl9	0.19892875	0.92882502	0.208	0.121	1
Matr3	0.19920304	-0.6545289	0.083	0.212	1
Pcnp	0.19926239	-0.6158249	0.083	0.212	1
Cep170	0.1993428	0.59916232	0.167	0.061	1
Luzp1	0.19965423	0.50460442	0.167	0.061	1
Adpgk	0.1996993	0.73366861	0.167	0.091	1
Idh2	0.19974208	-0.5335504	0.042	0.121	1
Arhgef1	0.20008787	-0.7606393	0.042	0.121	1
Ftl1	0.20049668	-0.5231571	0.792	0.879	1
Tmem38b	0.2005389	-0.4615282	0.042	0.152	1
Dusp3	0.20054813	1.0250562	0.167	0.091	1
Ndufs2	0.20066615	-0.4375517	0.083	0.212	1
Gm37494	0.20123413	0.62996064	0.167	0.061	1
Rbm4	0.20141791	0.42273234	0.125	0.03	1
Uvrag	0.20162096	0.42398058	0.125	0.03	1
Foxc1	0.20183113	-0.548893	0.25	0.455	1
Edar	0.20198055	0.55353741	0.125	0.03	1
Cped1	0.20200315	-0.34376	0.208	0.394	1
Bmp6	0.202645	-0.3319882	0.25	0.424	1
H2afj	0.20272676	-0.5163995	0.292	0.455	1
Tab2	0.2030267	-0.7380875	0.042	0.152	1
Sqstm1	0.20303339	-0.7938138	0.125	0.273	1
Klhdc8b	0.20442847	0.40035542	0.125	0.03	1
Ift20	0.20450384	-0.3172617	0.042	0.182	1
Tmem11	0.20493604	-0.7305344	0.042	0.152	1
Slc20a1	0.20499615	0.88851487	0.125	0.061	1

Aldh3a2	0.20527295	0.71836651	0.125	0.03	1
Med13	0.20536918	-0.6914853	0.083	0.212	1
Sdc3	0.20537086	0.6694795	0.167	0.061	1
Marcks	0.20539535	0.61563946	0.833	0.758	1
Snx5	0.20557508	0.34471414	0.167	0.061	1
Wbp4	0.20600468	-0.6178759	0.083	0.182	1
Srek1ip1	0.20628298	-0.7042923	0.042	0.121	1
Pdzrn4	0.20643517	-0.5783221	0.083	0.212	1
Klf3	0.20645162	-0.662435	0.042	0.121	1
Gsk3b	0.20659786	-0.4654876	0.292	0.485	1
Ano8	0.20679857	0.62257848	0.125	0.03	1
Cnot6l	0.20698038	0.98546313	0.125	0.061	1
Slbp	0.20725638	-0.5955345	0.125	0.242	1
Shoc2	0.20739097	0.71303793	0.125	0.03	1
Htatsf1	0.2075061	-0.6124523	0.042	0.121	1
Tmem189	0.2080414	0.44234377	0.125	0.03	1
Fxyd5	0.2080414	0.44234377	0.125	0.03	1
Srp14	0.20810717	0.56180974	0.375	0.212	1
Mark2	0.20873713	-0.7031165	0.042	0.121	1
Ubxn1	0.20902394	-0.5250148	0.125	0.242	1
Man2b1	0.20937758	-0.4702808	0.042	0.152	1
Elof1	0.210018	-0.5281067	0.042	0.152	1
Mrps14	0.21013865	0.42499604	0.167	0.061	1
C1galt1	0.21050094	0.50727217	0.125	0.061	1
Setdb1	0.21160598	0.28375196	0.125	0.03	1
Ythdf1	0.21215281	0.51812105	0.125	0.061	1
Elf1	0.21233501	0.82181612	0.292	0.152	1
Usp4	0.21265878	0.70381106	0.125	0.061	1
Cd248	0.21275258	0.64345202	0.125	0.03	1
Elk4	0.21293319	0.74560807	0.208	0.091	1
Arih2	0.21349229	-0.6181448	0.083	0.152	1
Pakap.1	0.21361512	0.43098435	0.208	0.091	1
Ankrd17	0.21422245	0.63358463	0.25	0.091	1
Spcs2	0.21455386	-0.5772436	0.125	0.242	1
Nfe2l1	0.21479301	-0.8065945	0.208	0.333	1
Rnasek	0.21496308	-0.723715	0.292	0.394	1
Lmo1	0.21505363	0.5410686	0.125	0.03	1
Atp5b	0.21517499	0.75501807	0.292	0.182	1
Rala	0.21537199	-0.5661055	0.042	0.152	1
Lpp	0.21570177	0.98848944	0.292	0.212	1
Ap1s1	0.21601316	0.40844832	0.208	0.061	1
Yipf1	0.21626881	-0.5229049	0.042	0.121	1
Ebf3	0.21669602	-0.251693	0.5	0.697	1

Numbl	0.21732604	0.54920694	0.125	0.03	1
Enpp5	0.21740274	-0.6471554	0.042	0.121	1
Arfrp1	0.21828188	0.95936352	0.125	0.061	1
Igfbp7	0.21830992	-0.2788407	0.708	0.818	1
Phex	0.21843408	0.8222038	0.333	0.212	1
Nedd8	0.21879589	-0.4584602	0.375	0.515	1
Cbll1	0.21903618	0.53302618	0.125	0.061	1
Fto	0.21928319	0.86940292	0.208	0.121	1
Myef2	0.21935691	0.47766325	0.167	0.061	1
Rapgef6	0.21945074	-0.7936817	0.125	0.212	1
Tnfaip3	0.2194669	-0.4825003	0.042	0.121	1
Glg1	0.21960044	0.62796794	0.208	0.091	1
Rnf38	0.22003573	0.38756231	0.125	0.03	1
Lnpep	0.22032041	0.46790895	0.375	0.212	1
Appl2	0.22041456	0.84201754	0.167	0.091	1
Khdrbs1	0.22094911	0.5743004	0.125	0.061	1
Uba52	0.22098776	-0.3451624	0.458	0.606	1
Sec61b	0.2210051	0.78332683	0.458	0.303	1
Mast4	0.22153644	0.59577274	0.25	0.121	1
Ubxn8	0.22158217	-0.2931426	0.042	0.182	1
Man2a1	0.22198884	-0.5345563	0.083	0.212	1
Wdr43	0.22207607	-0.4679259	0.042	0.152	1
Hikeshi	0.22225946	-0.6524432	0.083	0.121	1
Emsy	0.22239352	-0.583351	0.042	0.121	1
Rbfox2	0.2224929	0.57005678	0.208	0.091	1
Timm17a	0.22262946	-0.8433039	0.125	0.212	1
Npc2	0.22274795	-0.4853722	0.417	0.576	1
Sema6d	0.22308135	-0.6482225	0.125	0.242	1
Btbd1	0.22324078	0.41993387	0.208	0.061	1
Fam168a	0.22337592	0.41794954	0.125	0.061	1
Tmed3	0.22387438	0.42793572	0.333	0.152	1
Tm9sf1	0.22399053	0.44287223	0.208	0.091	1
Ddah2	0.2242266	-0.4101903	0.083	0.212	1
Asb3	0.22430928	0.68813758	0.125	0.061	1
Msrb1	0.22447804	-0.466434	0.042	0.152	1
Brk1	0.2245481	-0.6247922	0.083	0.212	1
Vdac2	0.22476651	0.5335719	0.292	0.152	1
Tbc1d23	0.2249753	0.58611158	0.167	0.061	1
Pgls	0.22541537	-0.5737452	0.167	0.303	1
Mycbp2	0.22555682	0.69644507	0.458	0.303	1
Txndc9	0.22589855	-0.5093985	0.083	0.182	1
Depdc5	0.2259388	0.58857621	0.167	0.061	1
Hadh	0.22596832	-0.4428484	0.042	0.121	1

Thra	0.22609025	-0.4903901	0.167	0.303	1
Cwc25	0.22625366	0.52776395	0.125	0.03	1
Rspo3	0.22696498	-0.5173478	0.083	0.212	1
Zcchc24	0.22721125	0.97284513	0.333	0.212	1
Pigu	0.22724846	0.64416219	0.167	0.091	1
Fbxw11	0.22733503	0.47197179	0.167	0.061	1
Hmgn3	0.22740992	0.69045669	0.333	0.182	1
Uqcr10	0.22760086	-0.5818803	0.333	0.455	1
Hebp1	0.22769318	-0.470963	0.042	0.152	1
Fam76a	0.22772529	-0.4207599	0.042	0.152	1
Atp6ap1	0.22782638	-0.6142366	0.042	0.152	1
Bet1	0.22782929	0.35039796	0.125	0.03	1
Plekhm2	0.22790268	-0.6066342	0.042	0.121	1
Klf13	0.22824222	-0.5782525	0.083	0.152	1
Eif4e3	0.22833713	0.35540084	0.125	0.03	1
Serf1	0.22843467	0.95095064	0.292	0.182	1
Rpp30	0.22849352	0.74189685	0.125	0.03	1
Hfe	0.2291623	-0.7620568	0.042	0.121	1
Ccnt2	0.2293641	0.67693659	0.125	0.061	1
Vamp8	0.22969635	-0.6035654	0.083	0.212	1
Usf2	0.22972224	0.96711725	0.167	0.091	1
Vasp	0.22992605	0.70208081	0.167	0.091	1
Ep400	0.22995962	-0.6701847	0.083	0.152	1
Tgds	0.23031914	-0.6070272	0.042	0.121	1
Prkaa2	0.23049897	-0.5996036	0.042	0.121	1
Tspan31	0.23059596	0.77370301	0.125	0.091	1
Arhgef25	0.23098858	0.72023284	0.125	0.061	1
0610012G03	0.23159825	0.61699493	0.208	0.121	1
Umad1	0.23161289	0.52507166	0.208	0.091	1
Gtf3c3	0.23163343	0.93467951	0.125	0.061	1
Psma4	0.23184491	-0.4049654	0.167	0.303	1
Serpina12	0.23187817	-0.4735686	0.25	0.424	1
Ybx3	0.2321541	0.69104086	0.208	0.121	1
Rab31	0.23247255	0.58248932	0.25	0.121	1
Luc7l2	0.23255837	0.44503297	0.5	0.333	1
Atxn7l3b	0.23278638	-0.4889894	0.208	0.333	1
Abhd17a	0.23309126	-0.4287701	0.042	0.152	1
R3hdm2	0.23342778	-0.6600387	0.083	0.212	1
Prrg2	0.23348569	-0.4706473	0.083	0.182	1
Raph1	0.23359296	-0.8022135	0.042	0.121	1
Ptges3	0.23371163	-0.6422198	0.25	0.394	1
Asph	0.23376775	0.53229156	0.333	0.212	1
Sat1	0.23379615	-0.2926454	0.125	0.303	1

Tifa	0.23380391	-0.5790247	0.083	0.182	1
Apc	0.23484532	0.50548113	0.125	0.03	1
Glmp	0.2351391	0.49774186	0.125	0.03	1
Ptch1	0.23519755	0.6454967	0.167	0.091	1
Dmpk	0.23532536	0.40777626	0.125	0.03	1
Trappc6a	0.23661895	-0.6112708	0.042	0.152	1
Myo9a	0.23732816	0.4890912	0.167	0.061	1
1300002E11I	0.23747363	0.67362789	0.125	0.061	1
Wdr5	0.23760914	0.86123696	0.167	0.091	1
Sf3b3	0.23774015	-0.6224814	0.167	0.273	1
Epn1	0.2377785	0.42602086	0.208	0.091	1
Ciao2b	0.23787806	-0.2672677	0.083	0.242	1
Col5a1	0.23843873	0.38497483	0.333	0.182	1
Npr2	0.23856744	0.76529273	0.167	0.091	1
Ier3ip1	0.23933943	0.66783089	0.292	0.182	1
Ggcx	0.23954531	0.76191705	0.125	0.061	1
Dact1	0.23958291	-0.8930883	0.125	0.212	1
Clpp	0.23999774	0.83265915	0.208	0.091	1
Rcn3	0.24000205	-0.7030597	0.333	0.424	1
Sp100	0.24001488	0.60009659	0.125	0.061	1
Ptov1	0.24097976	-0.3533088	0.125	0.303	1
Prkrip1	0.24108956	0.98151704	0.125	0.061	1
Irgm1	0.24111174	0.74081236	0.125	0.061	1
Cst3	0.24147689	-0.3274736	0.833	0.909	1
Gabpa	0.24161164	-0.5186219	0.042	0.121	1
Crot	0.24175749	-0.3662227	0.042	0.152	1
Ecpas	0.24299918	-0.7406278	0.125	0.242	1
Ehd1	0.2437062	-0.4347272	0.042	0.121	1
Timm17b	0.2437221	-0.5798349	0.042	0.121	1
Erh	0.24387786	-0.6092766	0.208	0.333	1
Cwc15	0.2440135	-0.6394155	0.083	0.182	1
Chpt1	0.24405163	-0.5052739	0.042	0.121	1
1810037I17R	0.2442495	0.61864242	0.5	0.394	1
Kdelr2	0.24461387	-0.5827	0.375	0.485	1
Tmem243	0.24493167	-0.7034739	0.042	0.152	1
Snrnp70	0.24592808	-0.4623628	0.25	0.394	1
Hadhb	0.24603175	-0.5016455	0.042	0.121	1
Fech	0.24619233	-0.41465	0.042	0.152	1
Pex2	0.24635816	-0.4952087	0.042	0.121	1
C2	0.24670792	0.72292425	0.375	0.242	1
Phf20l1	0.24768166	0.53852076	0.292	0.182	1
Atox1	0.24771779	-0.3545531	0.333	0.455	1
Nol7	0.24798822	-0.6939329	0.167	0.242	1

Itgav	0.24810957	-0.5555544	0.208	0.364	1
Aftph	0.24816499	-0.5041724	0.042	0.121	1
Rab2b	0.24866896	0.59091868	0.167	0.061	1
Pxdc1	0.24874695	-0.6631866	0.167	0.273	1
Ccnd2	0.24917652	0.65639651	0.167	0.061	1
Dlgap4	0.24953896	-0.5798201	0.042	0.121	1
Coq10b	0.24973235	-0.8536018	0.083	0.121	1
Edf1	0.2498556	-0.4947582	0.333	0.455	1
Arf5	0.2498873	0.33416077	0.375	0.212	1
Gstt1	0.24993174	0.47193287	0.167	0.061	1
Rora	0.25022876	-0.5542181	0.083	0.182	1
5730455P16I	0.25039286	-0.5397178	0.042	0.152	1
Dag1	0.2504927	0.55572945	0.292	0.182	1
Il10rb	0.25049517	-0.4191425	0.083	0.182	1
Retnlg	0.25060811	0.36949729	0.125	0.03	1
Eif3a	0.25074507	0.61426181	0.25	0.152	1
Gm4951	0.25124691	-0.3843414	0.167	0.333	1
Smim27	0.25132396	-0.4400978	0.167	0.303	1
Atpif1	0.25133914	-0.3804731	0.25	0.394	1
Tmem223	0.2513856	0.54222129	0.125	0.061	1
C6	0.25184441	1.04881556	0.167	0.091	1
Cald1	0.2521809	-0.4991609	0.333	0.455	1
Herc2	0.25233837	0.37332072	0.292	0.152	1
Slc12a4	0.25240287	-0.5337444	0.042	0.121	1
Plxnb2	0.25306055	0.7620265	0.125	0.061	1
Psd3	0.25319374	0.44096819	0.167	0.091	1
Anks3	0.25344858	0.34113804	0.125	0.03	1
Mbnl2	0.25390605	0.51483641	0.375	0.242	1
Lsm3	0.25391565	-0.6968683	0.125	0.212	1
Ak1	0.25404127	0.82709442	0.125	0.061	1
Ddhd2	0.25422483	0.37546092	0.125	0.03	1
Rbpj	0.25422672	-0.4678944	0.292	0.424	1
Chst15	0.25427816	-0.3457876	0.125	0.303	1
Ppp1r15a	0.25487023	-0.6323037	0.208	0.303	1
Slc39a13	0.25502004	-0.3807139	0.042	0.152	1
Lsm4	0.25547684	-0.5402919	0.083	0.152	1
Ppp3r1	0.2560789	-0.6001294	0.083	0.121	1
Tkt	0.25633251	-0.4744887	0.083	0.212	1
Camkmt	0.25650596	0.61742416	0.125	0.03	1
Atp2a2	0.25680125	-0.27008	0.208	0.333	1
Ube2i	0.25766329	-0.309641	0.208	0.364	1
Uqcc3	0.25768707	-0.5642809	0.125	0.212	1
Phf20	0.25780433	-0.6328379	0.042	0.121	1

Atrx	0.25821602	0.3110378	0.125	0.061	1
Aco2	0.25827044	0.51995075	0.125	0.061	1
Glud1	0.25838957	0.44064452	0.167	0.061	1
Arl6ip4	0.25850192	-0.516693	0.042	0.121	1
Hdac5	0.25860144	0.32714209	0.125	0.03	1
Atp6v0d1	0.25918533	-0.6355177	0.167	0.242	1
Mrpl30	0.25934009	0.79930143	0.208	0.152	1
Ksr1	0.25943253	-0.3767164	0.042	0.121	1
Maged1	0.2596172	0.71416051	0.375	0.212	1
Cggbp1	0.26009255	-0.2555177	0.125	0.273	1
Cox19	0.26031298	0.58124292	0.125	0.03	1
Mrps18a	0.26043468	-0.4328541	0.042	0.152	1
Arid5a	0.26099236	-0.4264996	0.042	0.152	1
Pla2r1	0.26135081	0.44014092	0.125	0.03	1
Polr2j	0.26164369	-0.2793937	0.083	0.242	1
Vps28	0.26189903	-0.6106365	0.083	0.182	1
Dop1a	0.26225607	0.41963812	0.125	0.03	1
Ackr4	0.26225904	-0.7224057	0.375	0.485	1
Colec12	0.26286326	0.31251077	0.583	0.394	1
Hmox1	0.2632523	-0.6688677	0.125	0.212	1
Abracl	0.26375611	0.63606812	0.125	0.061	1
Ing2	0.26452683	0.3946287	0.167	0.061	1
Ddt	0.26464716	-0.4734417	0.125	0.242	1
Rspo2	0.26468489	0.69745292	0.25	0.152	1
Rps2	0.26483615	-0.4976389	0.458	0.576	1
Ldhb	0.26501011	-0.2956405	0.042	0.152	1
Psma3	0.26503222	-0.32548	0.208	0.364	1
Mtdh	0.26513714	0.52069371	0.375	0.242	1
Rnpep	0.26566959	0.36750913	0.125	0.03	1
Col6a2	0.26632352	-0.4172861	0.125	0.273	1
Grina	0.2666108	0.78359079	0.375	0.242	1
Slc30a9	0.26689356	0.79715707	0.167	0.091	1
Rbm7	0.26704923	0.59168291	0.125	0.061	1
Ppp1ca	0.26755456	-0.5402877	0.167	0.303	1
Pja2	0.26758184	-0.4313259	0.042	0.121	1
Srp19	0.26759711	-0.7103799	0.083	0.152	1
Romo1	0.26850336	-0.4472535	0.417	0.545	1
Adarb1	0.26851407	0.77964175	0.167	0.091	1
Olfm1	0.26901002	0.34112952	0.167	0.061	1
Mcts1	0.26914605	0.34334994	0.167	0.061	1
Cd63	0.26920627	0.29929291	0.875	0.758	1
Apbb2	0.26939387	-0.4492735	0.333	0.424	1
Pank3	0.27071345	-0.5308122	0.042	0.121	1

Pcmt1	0.27097472	-0.5602128	0.083	0.152	1
Ndufb3	0.2711679	-0.4789158	0.167	0.273	1
Ppig	0.27157207	0.47460231	0.292	0.152	1
Ext2	0.27158753	0.81870636	0.167	0.091	1
Psme1	0.27189902	0.61130239	0.333	0.242	1
Rpp21	0.27191075	-0.5192192	0.042	0.121	1
Aph1a	0.27199877	0.84416154	0.125	0.091	1
Adap2	0.27207166	-0.4117508	0.042	0.121	1
Ipo5	0.27219009	0.52104151	0.208	0.091	1
Fut11	0.27225001	-0.413176	0.042	0.121	1
Ppm1a	0.27232292	-0.4505389	0.083	0.121	1
Rgcc	0.27241959	-0.8413855	0.292	0.394	1
Dhrs3	0.2725877	0.85524141	0.292	0.182	1
Spred1	0.27290018	-0.6504488	0.083	0.182	1
Rsl24d1	0.2729938	0.3041822	0.125	0.03	1
Nmt1	0.27305262	0.33028224	0.292	0.152	1
Hadha	0.27309632	-0.4559068	0.042	0.121	1
Papss2	0.27326257	-0.5096044	0.042	0.121	1
Csgalnact2	0.27326305	-0.3491121	0.042	0.152	1
Pik3c2a	0.27333548	0.46843243	0.125	0.03	1
Lsm12	0.27349205	0.26928728	0.125	0.03	1
Pet100	0.27373335	0.62655178	0.25	0.152	1
Spsb1	0.27378638	-0.2797134	0.042	0.152	1
Fth1	0.27380538	-0.3024217	0.917	0.939	1
Cdh11	0.2738549	0.75698879	0.708	0.697	1
Yap1	0.27385622	-0.6117107	0.083	0.152	1
Ddit4l	0.27396684	-0.4584771	0.042	0.121	1
Srsf4	0.27432511	-0.4594647	0.042	0.121	1
Kmt2a	0.2745927	0.50055875	0.25	0.121	1
Ube2d2a	0.2746463	0.52442047	0.292	0.182	1
Mfap3	0.27475392	-0.6261904	0.042	0.121	1
Itgb5	0.27492682	0.45836814	0.167	0.061	1
Snx3	0.2754143	-0.6541635	0.125	0.212	1
Pttg1ip	0.27558568	0.63776754	0.333	0.212	1
Jmjd6	0.27558931	-0.3935074	0.042	0.121	1
Usp15	0.27631232	-0.463637	0.042	0.121	1
Tomm34	0.2764272	0.82053477	0.125	0.061	1
Zhx3	0.27654409	0.77432405	0.125	0.061	1
Wwp1	0.27722739	0.4258178	0.125	0.03	1
Taf10	0.27729307	-0.4272113	0.083	0.182	1
Wdr70	0.27733587	-0.5293372	0.042	0.121	1
Rmnd1	0.27754546	0.93488294	0.208	0.121	1
Thsd4	0.27827027	0.82001647	0.208	0.121	1

Twist1	0.27834737	-0.9291559	0.125	0.182	1
Rpl8	0.27853831	-0.3783684	0.583	0.667	1
Pcgf3	0.27903527	-0.5010282	0.042	0.121	1
Acadsb	0.27914541	-0.5132664	0.042	0.121	1
Esyt1	0.27921982	-0.5442327	0.042	0.121	1
Arhgap5	0.27949272	0.90977489	0.25	0.121	1
Psma6	0.2797382	0.58896072	0.208	0.091	1
Impact	0.27982284	-0.6534109	0.083	0.152	1
Trip6	0.27990243	0.30892074	0.167	0.061	1
Ube3c	0.27993518	-0.5236891	0.042	0.121	1
Ap3s1	0.27997948	-0.5496005	0.083	0.182	1
Slain2	0.28006935	0.83662549	0.125	0.061	1
Tomm7	0.28022764	0.51157879	0.458	0.333	1
Mrpl15	0.28058142	0.43740907	0.125	0.03	1
Nop10	0.28060119	-0.5433991	0.208	0.364	1
Utp4	0.28142617	-0.5680907	0.042	0.121	1
Fgl2	0.28239215	1.07797949	0.167	0.121	1
Manf	0.28255076	-0.3953457	0.333	0.485	1
Nav2	0.28277083	0.3654713	0.125	0.03	1
Usp9x	0.28326197	-0.4008549	0.125	0.242	1
Kif2a	0.28391504	0.69628182	0.125	0.061	1
Ipmk	0.28420259	0.36382562	0.125	0.03	1
Abca1	0.28452925	-0.7716586	0.083	0.152	1
Stx12	0.28481345	0.32788211	0.125	0.03	1
Erp44	0.28500081	-0.3745237	0.083	0.212	1
Cpq	0.28517615	-0.2765162	0.292	0.424	1
Idh1	0.28530158	0.66017629	0.25	0.182	1
Cmtm6	0.28550658	0.35838002	0.208	0.091	1
Cfb	0.28611055	0.71939433	0.375	0.273	1
Laptm4a	0.28618812	-0.5132169	0.625	0.727	1
Trim12c	0.28635741	0.57118275	0.167	0.091	1
Frmd4a	0.28642075	-0.5228941	0.125	0.212	1
Pfdn1	0.28652922	0.74110299	0.167	0.121	1
Cul7	0.28666464	0.35376085	0.125	0.03	1
Setd2	0.28708146	0.74835813	0.167	0.091	1
Selenow	0.28715649	0.51356599	0.583	0.455	1
Ndufb10	0.28722691	-0.564151	0.25	0.364	1
Bloc1s1	0.28735324	-0.4825977	0.042	0.121	1
Wbp11	0.28748529	0.31229817	0.125	0.03	1
Tmem208	0.2883185	0.49812914	0.208	0.121	1
Synj2bp	0.28837027	0.43809758	0.167	0.091	1
Ufc1	0.28853982	-0.326183	0.083	0.212	1
Ctbp1	0.28888511	0.66836358	0.208	0.121	1

Calm3	0.28895068	-0.2921676	0.125	0.273	1
Ube2s	0.28908823	-0.5957587	0.167	0.242	1
Trappc4	0.28921457	-0.3966972	0.042	0.152	1
Brd3	0.2897327	-0.5735992	0.042	0.121	1
Cetn2	0.28981434	0.86038393	0.125	0.061	1
Galnt1	0.29007629	0.38945163	0.208	0.091	1
Mgmt	0.29035447	-0.6408643	0.083	0.152	1
Ndfip1	0.29101594	0.49944251	0.292	0.182	1
Cdk13	0.29148341	-0.4019034	0.042	0.121	1
Wdr1	0.29152676	0.42241662	0.25	0.121	1
Pde2a	0.29178903	-0.5024316	0.083	0.182	1
Psenen	0.29200738	-0.5286352	0.083	0.152	1
Abi2	0.29202033	0.51889401	0.125	0.061	1
Tbc1d15	0.29237169	-0.5300879	0.042	0.121	1
Psma7	0.29254619	-0.2746551	0.167	0.303	1
2310009A05I	0.29292783	0.51323135	0.292	0.182	1
Pin1	0.29347326	-0.4162956	0.083	0.182	1
Smarcd3	0.29382618	-0.4619017	0.042	0.121	1
Rbm25	0.29383604	0.47191124	0.333	0.212	1
Sarnp	0.29385775	0.8297072	0.208	0.121	1
Zcchc9	0.2944321	0.26605785	0.125	0.03	1
H6pd	0.29443682	0.56206608	0.125	0.061	1
Rnf2	0.29505288	-0.5970948	0.042	0.121	1
Tpm2	0.29514448	0.62848651	0.125	0.061	1
Pbrm1	0.29533012	0.59229557	0.208	0.121	1
Rps3	0.29551735	-0.4241549	0.458	0.576	1
Mrpl42	0.29563516	-0.5247539	0.042	0.121	1
Rnaset2a	0.29594223	-0.429802	0.083	0.182	1
Csnk2a1	0.29615521	-0.366631	0.25	0.394	1
Wnt4	0.29669728	0.50567803	0.25	0.152	1
Sh3bgrl3	0.29678192	-0.4948119	0.083	0.182	1
Cp	0.29739909	0.85028575	0.667	0.667	1
Med1	0.29740975	0.58099389	0.167	0.061	1
Sptssa	0.29756275	0.62908206	0.208	0.121	1
Adcy2	0.2977042	-0.4579549	0.125	0.212	1
Smap2	0.29789662	0.40013845	0.167	0.061	1
Atraid	0.29821245	-0.4930329	0.25	0.364	1
Fxyd1	0.29831584	-0.302943	0.25	0.394	1
Psmb1	0.29883826	0.45680289	0.292	0.182	1
Trip10	0.29896042	0.56880298	0.125	0.061	1
Mndal	0.29921696	-0.639877	0.083	0.152	1
Bcas2	0.29922717	-0.4528305	0.083	0.152	1
Sh3pxd2b	0.2995315	0.46771381	0.208	0.091	1

Acox1	0.30032205	-0.3155977	0.042	0.152	1
Fnip1	0.30039455	0.41685858	0.208	0.121	1
Snrpg	0.30055167	-0.373728	0.25	0.364	1
Ep300	0.30061393	0.64899837	0.208	0.091	1
Agpat3	0.30159529	0.5570989	0.167	0.091	1
Smim15	0.30268496	-0.5854519	0.083	0.182	1
Csde1	0.30293898	-0.5924434	0.25	0.333	1
Lyar	0.30359385	0.31918253	0.125	0.061	1
Tmem106b	0.30360153	-0.3644718	0.083	0.212	1
Slc4a2	0.30370395	-0.5923366	0.042	0.121	1
Dcn	0.30408062	1.73986194	0.458	0.424	1
Rps6kb1	0.30438996	0.50871198	0.208	0.121	1
Nhs	0.30460483	-0.342228	0.042	0.152	1
Far1	0.3050662	-0.7107042	0.083	0.121	1
Exoc3l4	0.30514837	-0.4196928	0.042	0.121	1
Ndufv1	0.3053211	-0.4321517	0.083	0.152	1
Dnajb14	0.30537655	0.53673412	0.167	0.061	1
Clec2d	0.30552984	-0.480158	0.458	0.545	1
Dnm1l	0.30593936	-0.5094678	0.125	0.212	1
Sypl	0.30596546	0.40935858	0.208	0.091	1
Ptpn9	0.30621535	0.33532686	0.125	0.061	1
Prdx2	0.30622401	0.68612807	0.333	0.242	1
Ndn	0.30648774	0.4119028	0.125	0.03	1
Arid4b	0.30649886	0.31067376	0.125	0.061	1
Cuta	0.30687998	0.90382959	0.208	0.121	1
Glis2	0.30700872	0.68698307	0.125	0.061	1
Nrbp2	0.30745867	-0.6477788	0.042	0.121	1
Ankrd13c	0.30754422	0.33117301	0.125	0.03	1
Borcs8	0.30803467	-0.473621	0.083	0.182	1
Svep1	0.30811803	0.69075964	0.333	0.273	1
Kif1b	0.308178	0.33518276	0.208	0.091	1
Nup153	0.30848116	-0.5456784	0.083	0.121	1
Sntb2	0.30921335	-0.3886313	0.042	0.152	1
Mrpl14	0.30974544	0.75255457	0.125	0.061	1
Slc12a2	0.3100486	0.30622196	0.167	0.061	1
Vps26a	0.31008151	-0.4638041	0.042	0.121	1
Egln2	0.31035078	0.31855539	0.125	0.03	1
Cbl	0.31035401	-0.4531104	0.042	0.121	1
Ndufc2	0.31055032	-0.6095849	0.208	0.303	1
Cenpb	0.31105923	0.43630124	0.167	0.061	1
Camta1	0.3111739	-0.3440713	0.042	0.121	1
Fam234a	0.31117542	0.49574561	0.125	0.061	1
Pebp1	0.3114103	-0.5185922	0.167	0.273	1

AW112010	0.31164725	-0.4684689	0.208	0.333	1
Ndel1	0.31174961	-0.6329434	0.083	0.121	1
Bmpr2	0.31200188	0.3522686	0.333	0.212	1
Eif5a	0.31282974	-0.379615	0.333	0.455	1
Setd5	0.31362669	-0.4412901	0.042	0.121	1
Mcur1	0.31422146	0.33674394	0.167	0.091	1
Man1a2	0.31441089	0.38343836	0.167	0.061	1
Pmepa1	0.31469384	0.34270184	0.5	0.333	1
Ndufa5	0.31530589	-0.3533449	0.208	0.303	1
Synpo	0.31604206	0.65967895	0.208	0.121	1
Dipk1a	0.31604636	0.59939363	0.125	0.061	1
Gclc	0.31632336	0.31120545	0.167	0.061	1
Tbck	0.31638823	0.34414903	0.125	0.03	1
Reep5	0.31643015	-0.3692711	0.333	0.455	1
Dpp8	0.31714169	-0.5605652	0.083	0.152	1
Gnai1	0.3172552	0.26016714	0.167	0.061	1
Mgat2	0.31738854	-0.3951927	0.042	0.121	1
Col5a3	0.31741545	0.27952518	0.25	0.121	1
Syf2	0.31756302	-0.3876446	0.083	0.212	1
Bicc1	0.3176258	0.62693319	0.458	0.394	1
Mthfsl	0.31795432	-0.3959142	0.042	0.121	1
Scd2	0.31840398	1.63238849	0.167	0.121	1
Plxdc2	0.31878635	0.42149583	0.625	0.545	1
Acbd3	0.31894989	-0.6019042	0.083	0.152	1
Hnrnpul1	0.31895604	-0.440721	0.125	0.182	1
Adgrf5	0.31953955	0.75296228	0.375	0.273	1
Chchd7	0.31966067	0.60799361	0.167	0.091	1
Ssr2	0.3197425	-0.2701759	0.125	0.242	1
Arhgdia	0.3198033	0.54929129	0.292	0.182	1
Flna	0.31986982	0.54860591	0.417	0.333	1
Agt	0.31988956	-0.3027712	0.333	0.455	1
Map4	0.31994117	-0.4433379	0.083	0.152	1
Ogn	0.32019984	0.68762309	0.583	0.515	1
Tuba1b	0.32103089	-0.4348938	0.125	0.242	1
Slc1a5	0.32109616	0.49275091	0.333	0.242	1
Mrap	0.32211883	-0.4285925	0.042	0.121	1
Etnk1	0.3223056	-0.3229054	0.125	0.242	1
Ppp2r3a	0.3225278	0.59533462	0.167	0.091	1
Clock	0.32311501	-0.3432139	0.083	0.182	1
Zfp638	0.32355072	0.56068745	0.167	0.091	1
Marf1	0.32393433	-0.4157703	0.042	0.121	1
Ufl1	0.32469124	0.31456763	0.167	0.061	1
Map7d1	0.32484258	-0.6337896	0.083	0.121	1

Simc1	0.32557973	-0.4145356	0.042	0.121	1
Golgb1	0.32603566	0.72642964	0.208	0.121	1
Mprip	0.32636509	0.48409224	0.333	0.212	1
Dusp11	0.32701524	0.67443515	0.333	0.242	1
Xrn2	0.3277466	0.43121929	0.167	0.091	1
Mien1	0.32822581	0.56916054	0.125	0.061	1
Ppp3ca	0.32850563	0.62238465	0.167	0.121	1
H1f0	0.32864588	0.91153903	0.125	0.091	1
Crtc3	0.32866198	-0.3872476	0.083	0.182	1
Cldn1	0.32952535	0.72193969	0.125	0.061	1
Bfar	0.32969371	0.50724957	0.167	0.091	1
Polk	0.32969634	-0.3845929	0.042	0.121	1
U2surp	0.33018427	0.57580156	0.25	0.182	1
Wls	0.33066656	0.70318904	0.333	0.242	1
Chchd1	0.33068042	-0.5225349	0.208	0.273	1
Gaa	0.33108568	-0.6171606	0.125	0.212	1
Ttc14	0.3316531	-0.5609661	0.042	0.121	1
Uqcrc1	0.33232741	-0.411755	0.125	0.212	1
Serp1	0.33241386	-0.6705623	0.125	0.182	1
Plekhm3	0.33270359	-0.2791684	0.042	0.121	1
Arid2	0.33287177	0.7504607	0.125	0.061	1
Bola2	0.33299609	0.61770522	0.208	0.121	1
Pdap1	0.33314428	-0.3539179	0.125	0.242	1
Amn1	0.33331477	-0.2806174	0.042	0.152	1
Stk11	0.33358426	-0.3898529	0.125	0.212	1
Srp68	0.33360626	0.60819674	0.125	0.061	1
Lats2	0.33407437	-0.4635674	0.083	0.121	1
Uba2	0.33434933	0.53555028	0.125	0.061	1
Rbis	0.33465769	-0.7304546	0.167	0.242	1
Marcksl1	0.33473133	-0.5903536	0.125	0.212	1
Galk1	0.33481446	0.70453142	0.125	0.061	1
Cnn3	0.33689691	0.81626151	0.25	0.152	1
Gm16286	0.33707181	-0.3294985	0.042	0.121	1
2610300M13	0.33712566	0.34009261	0.125	0.061	1
Cnot4	0.33719387	-0.3985663	0.083	0.182	1
Golga2	0.33803797	-0.417184	0.083	0.152	1
Rnf181	0.33811899	0.74409663	0.125	0.061	1
Smc1a	0.33880718	-0.5171652	0.125	0.212	1
Axl	0.3389694	1.26707916	0.167	0.091	1
Kdm5b	0.33915172	-0.3149004	0.125	0.242	1
Edem3	0.33967983	0.30438152	0.125	0.061	1
Ogt	0.34006319	0.48136129	0.333	0.212	1
Rc3h2	0.34023488	0.26679058	0.167	0.061	1

Pan3	0.34026368	-0.6380124	0.083	0.121	1
Cd99l2	0.34028128	0.60956469	0.125	0.061	1
Rsrc1	0.34068713	0.42619847	0.333	0.212	1
Arhgef17	0.34088632	0.31733296	0.167	0.061	1
Emg1	0.34108492	0.64621932	0.167	0.091	1
Ergic2	0.34125829	0.67943205	0.208	0.152	1
Slit3	0.34140903	-0.3704415	0.042	0.121	1
Rab8b	0.34148695	-0.5895717	0.125	0.212	1
Myc	0.34172611	-0.8010051	0.167	0.242	1
Cyba	0.34206047	-0.3291221	0.25	0.364	1
Irf8	0.34243957	-0.7043315	0.083	0.121	1
Sppl3	0.34316611	-0.4818874	0.083	0.152	1
Pofut2	0.34320538	0.42797722	0.25	0.152	1
Prrc2c	0.34323175	-0.3893656	0.25	0.364	1
Hsp90aa1	0.34334842	-0.3923868	0.208	0.333	1
Lmbrd1	0.3434507	0.33107676	0.25	0.121	1
Mat2b	0.34355556	0.47937412	0.208	0.121	1
B230219D22	0.34357506	0.52655122	0.292	0.212	1
Dda1	0.34426144	0.30313757	0.125	0.03	1
Timm9	0.34440425	0.49260065	0.125	0.061	1
Igf2r	0.34457708	0.34697484	0.167	0.061	1
Fnta	0.34475741	0.68266286	0.125	0.061	1
Hmgcl	0.34512911	-0.5911969	0.083	0.121	1
Itm2c	0.34573031	-0.3748981	0.292	0.394	1
Actg1	0.34604647	0.31753905	0.583	0.485	1
Maged2	0.34623112	-0.519964	0.083	0.121	1
Derl1	0.34644077	0.42115615	0.167	0.091	1
Sec22b	0.34691525	0.66922503	0.125	0.061	1
Ndufv3	0.3480767	0.63367865	0.333	0.273	1
Impdh2	0.34823942	0.27722967	0.125	0.061	1
Rgs3	0.34830759	0.45872962	0.208	0.121	1
Zscan26	0.34831496	-0.5047862	0.125	0.212	1
Eif3j1	0.34861025	0.32204459	0.292	0.182	1
Ap3b1	0.34898658	0.31042619	0.208	0.091	1
Eif4h	0.34914298	-0.595142	0.125	0.182	1
Tsc22d2	0.34916268	-0.3851947	0.125	0.182	1
Psmd11	0.3495233	-0.3220766	0.167	0.303	1
Zfand5	0.34954528	-0.5587382	0.375	0.455	1
Ppp4r3b	0.35062874	0.55053761	0.25	0.121	1
Atp2c1	0.35093927	0.52676427	0.167	0.091	1
Fat1	0.35110245	0.48203911	0.417	0.333	1
Thbd	0.35128459	-0.5440789	0.167	0.242	1
Tbrg1	0.35156811	-0.5245991	0.125	0.182	1

Ndufb7	0.3519251	0.30748525	0.292	0.152	1
Cdc5l	0.35221945	-0.5682122	0.083	0.121	1
Nab1	0.3530779	0.62826801	0.125	0.061	1
Rgl2	0.35319136	-0.355465	0.042	0.121	1
Cbx5	0.35353408	0.42910826	0.167	0.091	1
Polr2m	0.35356843	-0.3391848	0.083	0.152	1
Map3k5	0.35361132	-0.4088153	0.083	0.152	1
Thap3	0.35384499	0.63381112	0.125	0.061	1
Zdhhc20	0.35397066	-0.6885511	0.083	0.182	1
Trappc10	0.35428729	0.34231786	0.125	0.061	1
Ppp1r2	0.35445255	-0.4422124	0.208	0.303	1
Vma21	0.35491243	-0.4598889	0.083	0.152	1
Cdc42ep3	0.3552173	-0.4867345	0.083	0.152	1
Ddr1	0.35585581	-0.6699117	0.042	0.121	1
Mpdz	0.3566799	0.45129456	0.208	0.121	1
Abce1	0.3570073	-0.5576547	0.083	0.152	1
G0s2	0.35734389	-0.850799	0.083	0.152	1
Ank3	0.3580716	0.47457921	0.125	0.061	1
Add3	0.35830023	0.38552574	0.125	0.061	1
Serpinb6a	0.35837545	0.70714876	0.25	0.212	1
Ebp	0.3586113	0.50377021	0.125	0.061	1
Brd8	0.359018	0.52704765	0.125	0.091	1
Pgd	0.35914579	0.6126584	0.208	0.152	1
Pgk1	0.35918943	-0.3196516	0.042	0.121	1
Prkcd	0.35932432	0.47990152	0.125	0.061	1
Atp6v1g1	0.35948747	0.45030756	0.292	0.182	1
Psmb6	0.35954723	-0.3849885	0.167	0.242	1
Nrep	0.35983156	0.65741736	0.125	0.061	1
Loxl2	0.35987762	0.40520121	0.167	0.091	1
Pim3	0.35988958	-0.7280313	0.167	0.182	1
Dlst	0.35990546	-0.3677935	0.125	0.212	1
Snhg3	0.36013089	0.66363022	0.167	0.091	1
Ldb1	0.360256	-0.4324797	0.042	0.121	1
Pole4	0.36045572	-0.2513972	0.083	0.182	1
Mitd1	0.36061949	0.47566061	0.125	0.061	1
Dst	0.36081368	0.37365252	0.167	0.091	1
Arf1	0.3610962	-0.4590882	0.292	0.394	1
Adgrl2	0.36121529	0.47547034	0.25	0.182	1
Txlng	0.36145872	0.29936394	0.167	0.061	1
Eif1a	0.36160112	-0.3874175	0.083	0.152	1
Tmed7	0.36186402	-0.7515211	0.125	0.152	1
Snx1	0.36190866	-0.4663188	0.083	0.152	1
Trim2	0.36257254	0.57736616	0.208	0.121	1

Ogfr	0.36264719	-0.4773642	0.083	0.152	1
Larp4b	0.36331087	0.34016118	0.125	0.061	1
Tle2	0.36375424	0.64929642	0.375	0.303	1
Alg5	0.36406692	0.37597119	0.167	0.091	1
Cfap298	0.36411814	-0.283016	0.042	0.121	1
Gnai2	0.36551105	-0.3132445	0.333	0.424	1
Hbb-bs	0.36571627	0.98332783	0.292	0.273	1
Spon1	0.36587118	0.93882388	0.208	0.152	1
Hsp90ab1	0.366077	0.47769064	0.625	0.545	1
Il1r1	0.36612281	-0.4211887	0.292	0.394	1
Rac1	0.3664871	0.50884038	0.333	0.212	1
Nmi	0.36678075	-0.3031889	0.042	0.121	1
Snhg11	0.36746759	0.70776775	0.125	0.091	1
Rpl12	0.36775581	-0.2532541	0.292	0.424	1
Gm2a	0.36775847	0.37013736	0.292	0.182	1
Ubr1	0.36790463	-0.6593408	0.167	0.212	1
Pias1	0.36801642	-0.3452716	0.042	0.121	1
Alkbh1	0.36816266	-0.2904169	0.042	0.121	1
Lpgat1	0.36848745	0.54350687	0.208	0.121	1
Pcbp1	0.36937835	-0.3833086	0.25	0.364	1
Prpf39	0.36974264	-0.4173834	0.083	0.152	1
Cavin1	0.36974612	0.54920523	0.292	0.212	1
Camk2n1	0.37036044	-0.2786447	0.292	0.394	1
Snd1	0.37058074	-0.3336299	0.083	0.152	1
Sdhaf4	0.37096155	-0.3001493	0.042	0.121	1
Zmynd8	0.37160524	0.26840437	0.25	0.121	1
Mier1	0.37224175	-0.6239295	0.167	0.212	1
Cdc42bpa	0.37258333	0.45610242	0.125	0.061	1
Rbms3	0.37273593	0.67830725	0.167	0.091	1
Camp	0.37310016	-0.9300155	0.167	0.242	1
Lamb1	0.37367681	0.49900373	0.417	0.333	1
Sec24a	0.37368317	0.36001165	0.125	0.061	1
Map4k2	0.37370707	-0.2948011	0.042	0.121	1
Ctsf	0.37401956	-0.8744496	0.125	0.152	1
Suclg2	0.37422526	0.52135423	0.125	0.091	1
Wasl	0.37454284	0.44221658	0.208	0.121	1
Plekhg2	0.37464617	0.62680174	0.125	0.061	1
Snx30	0.37495691	0.59592072	0.125	0.061	1
Akap9	0.37522315	0.59466013	0.25	0.182	1
Tcf4	0.37546351	0.80164976	0.333	0.273	1
Pbx1	0.37549191	-0.2890053	0.042	0.121	1
Txnip	0.37574973	0.49215063	0.458	0.364	1
Fscn1	0.37667047	-0.4788416	0.083	0.152	1

Amotl2	0.37685733	0.46797368	0.417	0.333	1
Ppid	0.37689323	0.45666942	0.125	0.061	1
Fut8	0.37712241	0.48075405	0.208	0.121	1
P4hb	0.37734452	-0.3839943	0.417	0.545	1
Ncoa6	0.37776143	0.3090115	0.125	0.061	1
Zmym5	0.37806829	-0.3862038	0.083	0.152	1
mt-Atp6	0.37860616	0.65784502	0.875	0.939	1
Dlg5	0.37889637	0.40265756	0.25	0.121	1
Rab5c	0.37904457	-0.4980703	0.083	0.152	1
Slc25a25	0.37976976	-0.302342	0.083	0.152	1
Zfp592	0.38128906	-0.3209333	0.042	0.121	1
Unc5b	0.38134369	-0.4723954	0.083	0.121	1
Celf1	0.38151612	-0.3740515	0.042	0.121	1
Ptp4a2	0.3815677	0.72751591	0.333	0.242	1
Lmf2	0.38195318	-0.4413036	0.083	0.182	1
Klhl9	0.38199687	0.52311849	0.167	0.121	1
Hnrnpa3	0.3820302	0.66190988	0.375	0.303	1
Atn1	0.38252328	-0.4689384	0.125	0.212	1
Ldlrap1	0.3826995	0.48843741	0.167	0.091	1
Ppp3cb	0.38293845	0.6095382	0.167	0.121	1
Syap1	0.38296466	-0.4965775	0.125	0.182	1
Ubn1	0.38321174	0.66623685	0.167	0.121	1
Ube2k	0.38325905	0.39252621	0.167	0.091	1
Sirt1	0.38373231	-0.4556784	0.042	0.121	1
Cir1	0.38387245	0.35254839	0.167	0.091	1
Bptf	0.38391876	0.39720451	0.125	0.061	1
Mir22hg	0.38392885	-0.5019583	0.083	0.121	1
Scaf8	0.38411466	-0.4390327	0.083	0.152	1
Ssbp3	0.38430514	0.66151161	0.125	0.061	1
Nrip1	0.38437016	0.47725901	0.208	0.121	1
Dhx9	0.38502499	-0.3261968	0.083	0.152	1
Atp5d	0.38523781	0.32594296	0.417	0.303	1
Rab3il1	0.3852776	0.5362932	0.125	0.061	1
Esr1	0.38538543	-0.3103538	0.167	0.273	1
Ahcyl2	0.38543148	-0.3945088	0.083	0.152	1
Adh5	0.38556048	-0.2518724	0.042	0.121	1
Txnrd1	0.38646503	0.36609966	0.167	0.091	1
Atxn10	0.38743616	-0.3527386	0.083	0.152	1
Tln1	0.3881212	0.53432658	0.417	0.303	1
BC028528	0.38821056	-0.4343101	0.083	0.152	1
Fkbp2	0.38830285	-0.6148786	0.208	0.273	1
Clec11a	0.38883469	0.50731165	0.25	0.152	1
Ankrd12	0.38903248	0.55737101	0.292	0.212	1

Lbp	0.38914948	-0.3222378	0.25	0.364	1
Fchsd2	0.38958117	0.47939917	0.125	0.061	1
Tmem30a	0.38996636	-0.3503861	0.167	0.242	1
Sel1l	0.39019434	0.48515758	0.25	0.152	1
Cdk4	0.39024944	-0.3609748	0.125	0.212	1
Add1	0.39056548	0.81355789	0.167	0.152	1
Trrap	0.39064791	-0.2688695	0.042	0.121	1
Pbxip1	0.39072858	-0.4449994	0.125	0.182	1
Ptbp3	0.39102369	0.45541457	0.292	0.212	1
Gab1	0.39160988	0.39073055	0.125	0.061	1
Atxn7l3	0.39240114	-0.26737	0.042	0.121	1
Snhg8	0.39313622	0.51349271	0.25	0.152	1
Eif3c	0.39343082	0.44186201	0.125	0.061	1
Nomo1	0.39367776	0.3869686	0.125	0.061	1
Ireb2	0.39406454	0.45961054	0.125	0.061	1
Aida	0.39413446	-0.5443333	0.125	0.182	1
Banf1	0.39419398	-0.5290628	0.083	0.121	1
Ggh	0.39427565	-0.6388841	0.208	0.273	1
Tnc	0.39445347	0.74631685	0.833	0.879	1
Ctsk	0.39477997	-0.3000276	0.208	0.303	1
Ndufs5	0.3958825	0.4291143	0.375	0.273	1
G3bp2	0.39671096	-0.4275036	0.083	0.152	1
Trim56	0.39691902	-0.3245961	0.042	0.121	1
Kpna4	0.39739881	0.63996159	0.125	0.091	1
Id1	0.39747488	-0.2566287	0.042	0.121	1
Abcf1	0.39772174	-0.4350027	0.083	0.121	1
Ppp1r12a	0.3980634	-0.5162958	0.125	0.182	1
Clk3	0.39810845	0.4976466	0.125	0.061	1
Hgs	0.39820098	0.27177594	0.125	0.061	1
Tm7sf3	0.39824955	-0.3259531	0.083	0.182	1
Stub1	0.39882385	0.4703711	0.25	0.182	1
Plin2	0.39884962	-0.3227167	0.042	0.121	1
Fundc2	0.3991116	0.31661169	0.292	0.182	1
Arl8b	0.39917978	0.43964282	0.167	0.121	1
Fasn	0.39924405	0.38031692	0.125	0.061	1
Dnajc3	0.39961771	-0.3763881	0.375	0.455	1
Hnrnpu	0.39975507	0.53049203	0.417	0.333	1
Lrp10	0.40102555	0.38807715	0.167	0.091	1
Sf3b6	0.40133886	-0.3598539	0.167	0.273	1
Psmb2	0.40260991	-0.4915098	0.25	0.333	1
Nop58	0.40269685	-0.6348696	0.167	0.182	1
5-Mar	0.40297085	-0.5351443	0.083	0.121	1
Rap1b	0.40301189	-0.5001006	0.208	0.273	1

Rtl8a	0.40360423	0.71860685	0.125	0.091	1
Dusp6	0.40416684	-0.3399253	0.125	0.212	1
Crebrf	0.40473464	0.48838797	0.167	0.121	1
Cdk9	0.40498246	0.70128012	0.125	0.121	1
Zbtb20	0.40519177	0.46674118	0.542	0.455	1
Eef1d	0.40521218	0.25676155	0.375	0.242	1
Sgce	0.40539142	-0.4807872	0.083	0.152	1
Paip2	0.40571382	0.5050137	0.333	0.242	1
Bcl7c	0.40573724	0.31806276	0.167	0.091	1
Abi3bp	0.40582229	-0.5201217	0.458	0.545	1
Setd1b	0.40602446	-0.2911018	0.042	0.121	1
Mon2	0.406443	0.31635339	0.125	0.061	1
Rbm5	0.40705593	0.27183423	0.292	0.182	1
Polg	0.40708142	-0.270128	0.042	0.121	1
App	0.40824242	0.3494932	0.708	0.636	1
Smg7	0.40863258	-0.4872079	0.125	0.182	1
Dpy30	0.40975725	0.48213311	0.167	0.121	1
Osbpl9	0.40992522	-0.7330537	0.125	0.152	1
Dtnbp1	0.41024648	-0.4981961	0.125	0.182	1
Fap	0.41105991	0.65939378	0.333	0.273	1
Ctsa	0.4110628	0.36201394	0.125	0.061	1
Nfic	0.41163312	-0.3402409	0.125	0.212	1
Irf2bpl	0.41176973	0.48426526	0.125	0.061	1
Lmna	0.41193347	-0.8475319	0.333	0.424	1
Gtf2a1	0.41201303	0.52080012	0.25	0.182	1
Capza2	0.41362901	-0.4306749	0.25	0.333	1
Xist	0.41378205	-0.597538	0.125	0.182	1
Twf1	0.41486094	0.33029414	0.167	0.091	1
Amfr	0.41494232	0.33801562	0.167	0.091	1
Cdk12	0.415326	0.37206044	0.167	0.091	1
Tm9sf2	0.41585596	-0.3840758	0.333	0.394	1
Ppp6r3	0.41599015	-0.376893	0.083	0.182	1
Kctd10	0.41618516	0.30700332	0.125	0.061	1
Rhoa	0.4162514	0.32760761	0.333	0.485	1
Lrrc58	0.4170812	-0.5655391	0.375	0.424	1
Ndufv2	0.41779115	0.61552222	0.25	0.182	1
Hnrnpa0	0.41782655	0.26990031	0.292	0.182	1
Tshz2	0.41814389	0.40809462	0.167	0.091	1
Sec63	0.41832454	0.59049047	0.25	0.182	1
Nucb1	0.41894131	0.42612264	0.333	0.242	1
Ddx5	0.41900498	-0.3156979	0.792	0.848	1
Cdc37	0.41900686	0.49666347	0.167	0.061	1
Ppil4	0.41940205	0.78776091	0.125	0.091	1

Svbp	0.4197824	-0.2930099	0.083	0.152	1
Thrap3	0.42019236	-0.6167934	0.208	0.242	1
Ece1	0.4206883	0.26863355	0.208	0.121	1
Wtap	0.42077912	-0.4836006	0.125	0.152	1
Arpc1b	0.42170507	-0.4389428	0.208	0.273	1
Rps27l	0.42182521	0.48965565	0.458	0.394	1
Srp72	0.42207061	-0.373972	0.125	0.182	1
Tnfrsf21	0.42223725	-0.3163551	0.167	0.242	1
Sdc2	0.42252862	0.52502762	0.375	0.303	1
Cox7a2l	0.42304235	0.38997369	0.208	0.121	1
Arpp19	0.42364681	-0.4698951	0.208	0.242	1
Lcorl	0.4246027	0.33170431	0.125	0.061	1
Zfp938	0.42538409	-0.4637873	0.125	0.182	1
Shox2	0.42602526	-0.363217	0.125	0.212	1
Dstn	0.42633629	0.33627571	0.5	0.394	1
Ube2r2	0.42703262	0.53729591	0.25	0.182	1
Epm2aip1	0.42713068	0.35892765	0.125	0.061	1
Myl6	0.42757251	0.34595863	0.583	0.515	1
Rab21	0.42773645	-0.4251593	0.167	0.212	1
Pptc7	0.42787137	0.25875566	0.125	0.03	1
Clcn4	0.42794947	0.52549055	0.167	0.091	1
Rasa3	0.42834486	-0.5017762	0.208	0.273	1
Kansl3	0.42847041	-0.3863201	0.083	0.152	1
Emp3	0.42925003	0.6589137	0.5	0.455	1
Mylip	0.43107837	-0.3663268	0.083	0.152	1
Dock6	0.4310999	0.34527486	0.125	0.061	1
Lgals3bp	0.43164974	-0.3753302	0.333	0.394	1
Ghr	0.43170937	0.50685056	0.5	0.455	1
Tnks	0.43202292	-0.3951603	0.083	0.121	1
Asxl2	0.43234351	0.39594024	0.125	0.061	1
Tpgs1	0.43428221	0.38241448	0.125	0.061	1
Adgra2	0.43472465	0.42060074	0.125	0.061	1
Fzd5	0.43473369	0.3703255	0.375	0.273	1
Ccnd3	0.43519799	0.52686285	0.25	0.182	1
Tcf7l2	0.43543092	0.42078888	0.417	0.333	1
Pura	0.43593285	0.38055291	0.208	0.121	1
Actb	0.43597063	-0.4459014	0.958	0.939	1
S100a8	0.4365888	-2.0343588	0.458	0.485	1
Bcar1	0.43659049	0.58553713	0.125	0.091	1
Eapp	0.4370191	-0.344138	0.083	0.152	1
Kpnb1	0.43756195	-0.256305	0.083	0.152	1
Iqgap1	0.43766338	0.4835893	0.333	0.242	1
Cox6b1	0.43796961	0.28811945	0.375	0.273	1

Ifi35	0.4390744	-0.470824	0.083	0.121	1
Sgms2	0.43983984	0.37714653	0.167	0.091	1
Elavl1	0.44009436	0.29962689	0.167	0.091	1
Chmp2a	0.44033994	0.27155734	0.375	0.242	1
Mdp1	0.4422591	0.65460851	0.167	0.121	1
Rnps1	0.44337686	-0.5408996	0.125	0.121	1
Tpbg	0.44340362	-0.2839994	0.083	0.152	1
Itpr2	0.44365018	0.37225477	0.208	0.121	1
Pigk	0.4436697	0.30399518	0.208	0.121	1
Ssu72	0.44457584	0.40253162	0.208	0.152	1
Mrps16	0.4448105	0.3705925	0.125	0.091	1
Tef	0.44505348	0.41433176	0.167	0.091	1
Rbm26	0.44552917	-0.5682217	0.167	0.212	1
Rnf167	0.44580431	0.34960154	0.167	0.091	1
Gpalpp1	0.44643834	0.31687928	0.125	0.061	1
Gbf1	0.44662997	-0.2679634	0.083	0.152	1
Arglu1	0.4476364	0.54104451	0.292	0.212	1
Tbl2	0.44791485	-0.3895433	0.083	0.152	1
Lemd2	0.44860047	0.33928476	0.125	0.061	1
Ndufaf8	0.44900077	-0.3890239	0.083	0.152	1
Anxa6	0.45034759	-0.3833483	0.083	0.152	1
Eif3h	0.45103891	-0.2706687	0.083	0.152	1
Rab34	0.45111914	0.50669379	0.125	0.061	1
Ppp1r14b	0.45135044	-0.3720322	0.125	0.182	1
Cers5	0.45178832	-0.5080208	0.125	0.152	1
Fxr1	0.45304142	0.27867952	0.208	0.121	1
C2cd5	0.45328972	0.46011908	0.125	0.091	1
Tpp2	0.45390146	0.35769992	0.125	0.061	1
Wwtr1	0.45459951	-0.4831287	0.083	0.121	1
Zranb2	0.454739	-0.4571943	0.083	0.121	1
Gna13	0.45524375	0.33504205	0.167	0.061	1
Shisa5	0.45530994	-0.5406516	0.125	0.152	1
H2-Q4	0.45614595	0.54101365	0.25	0.212	1
Lamc1	0.45619068	0.2951986	0.333	0.212	1
Mvp	0.45637258	0.39485613	0.208	0.121	1
Sdf2	0.45702811	0.26493268	0.25	0.152	1
Chrac1	0.45704016	-0.4535894	0.125	0.182	1
Mmut	0.45752726	0.36926593	0.125	0.061	1
Ecrg4	0.45787761	0.81050701	0.25	0.182	1
Rgl1	0.45797152	0.64211274	0.208	0.152	1
Rsl1d1	0.4594277	0.33194246	0.167	0.121	1
Gstm2	0.45994349	-0.2540891	0.125	0.182	1
Srpx2	0.4604755	0.56649686	0.208	0.152	1

Oat	0.46070562	-0.4238754	0.292	0.364	1
Tcea1	0.46110098	0.27617035	0.208	0.121	1
Mxd4	0.46110865	0.28616987	0.375	0.273	1
Smim26	0.46173246	0.29129553	0.125	0.061	1
Srrm2	0.46177369	-0.2818264	0.458	0.545	1
Ccn2	0.46192797	0.93862183	0.5	0.455	1
Cul1	0.4619587	0.67017081	0.292	0.242	1
Napa	0.4621189	0.31001693	0.25	0.152	1
Bsg	0.46263868	-0.2919047	0.292	0.394	1
Stxbp6	0.46362004	0.30101025	0.167	0.091	1
Scara3	0.46422615	-0.4497425	0.292	0.364	1
Igf2	0.4643453	0.35466582	0.125	0.091	1
Vasn	0.46562848	-0.8105915	0.292	0.364	1
Ppp1r10	0.46593163	-0.3087761	0.083	0.152	1
Pold4	0.46633585	-0.2509903	0.042	0.121	1
Rp9	0.4665545	0.44437306	0.25	0.152	1
Ndrg1	0.46662247	0.35868567	0.125	0.091	1
Arpc5	0.46747197	0.31709872	0.167	0.091	1
Fbrsl1	0.46824586	-0.3872516	0.083	0.152	1
Mgat1	0.46907153	0.34014895	0.167	0.121	1
Bmp2k	0.46946715	0.38515821	0.167	0.121	1
Ptk7	0.47008162	0.62662159	0.167	0.121	1
Map2k3	0.47030229	-0.4328184	0.083	0.152	1
Cd151	0.47049512	0.26496709	0.167	0.091	1
Maz	0.47075761	0.35286253	0.167	0.121	1
Mkln1	0.47076602	0.5469882	0.125	0.091	1
Zfp280d	0.4710224	0.26667101	0.125	0.061	1
Gng12	0.47129949	0.43151098	0.125	0.091	1
Arnt	0.4713417	0.66945211	0.167	0.121	1
Tomm20	0.47175539	0.35201115	0.25	0.152	1
Tpp1	0.47181465	0.33902246	0.125	0.091	1
Klf2	0.47256458	0.79893749	0.333	0.273	1
Pmp22	0.47261041	1.40094134	0.167	0.333	1
Tmem128	0.47320186	0.38458784	0.125	0.061	1
Ifi202b	0.47348364	0.5251519	0.167	0.091	1
Jag1	0.47360922	-0.333954	0.167	0.242	1
2610001J05F	0.47414393	-0.3179521	0.083	0.121	1
Mbd2	0.47482791	-0.361888	0.083	0.152	1
Tmem134	0.47504196	-0.2911708	0.083	0.152	1
Rnd3	0.47531163	-0.4673751	0.333	0.394	1
Csad	0.47534765	0.25106782	0.208	0.121	1
Polr2e	0.47536217	0.27829245	0.125	0.061	1
Bud31	0.47540102	0.27113908	0.125	0.061	1

Rpl23	0.47558435	0.66106615	0.708	0.697	1
Polr2g	0.47607992	0.26358243	0.25	0.152	1
Ptprd	0.4762565	0.30977436	0.667	0.636	1
Sec62	0.47730802	-0.4536228	0.417	0.485	1
Fam20a	0.47845766	0.29040714	0.125	0.091	1
Ndrg2	0.47883978	-0.4030812	0.125	0.182	1
Col4a2	0.47899986	0.42049117	0.458	0.394	1
Tsc22d4	0.47903622	0.35979397	0.167	0.121	1
Nfkb2	0.47925977	0.5411098	0.167	0.121	1
Sh3rf1	0.4793562	-0.4029471	0.083	0.121	1
Per3	0.47961509	0.25767598	0.125	0.061	1
Scmh1	0.48101788	0.42695515	0.125	0.091	1
Lifr	0.48126195	1.05081054	0.417	0.485	1
Ginm1	0.4815718	-0.4065704	0.167	0.242	1
Gnb2	0.48192764	-0.3153899	0.333	0.394	1
Lix1l	0.48197871	0.35075854	0.167	0.091	1
Tmem33	0.48394874	0.63454725	0.167	0.121	1
Hnrnpf	0.48555241	-0.4662783	0.25	0.303	1
Dnajb4	0.48575079	-0.3127299	0.167	0.242	1
Slc5a3	0.48583738	0.78788335	0.417	0.394	1
Elovl1	0.48614267	-0.2652416	0.083	0.152	1
Lmo7	0.48621939	0.65272935	0.25	0.182	1
Trim26	0.48626644	0.37393369	0.167	0.091	1
Aldh2	0.48716255	0.33093909	0.333	0.242	1
Col3a1	0.48732702	1.17497643	0.167	0.182	1
Gatad2b	0.48763932	-0.3410863	0.083	0.121	1
Phf23	0.48785812	0.49239588	0.125	0.091	1
Anapc16	0.48826367	0.27509921	0.167	0.091	1
Pgam1	0.48861924	0.56937277	0.125	0.091	1
Tmf1	0.48926937	0.28844547	0.208	0.121	1
Arl6ip5	0.49075567	0.62119445	0.125	0.091	1
Xiap	0.49092088	-0.3844787	0.125	0.152	1
Cdkn1b	0.49183696	0.45453886	0.167	0.121	1
Ralbp1	0.49220971	0.31492744	0.25	0.182	1
Icam1	0.49500066	-0.5670314	0.208	0.273	1
Lars2	0.49536383	-0.3464894	0.625	0.485	1
Tmem230	0.49560708	0.66911513	0.125	0.091	1
Ube2e3	0.49594065	-0.3312539	0.083	0.121	1
Rel	0.49645418	-0.4058732	0.167	0.212	1
Il1rn	0.4981092	-0.5556553	0.292	0.333	1
Arid1a	0.49844642	-0.4666108	0.125	0.121	1
Pdia5	0.4992144	0.72430908	0.125	0.091	1
Nrbf2	0.49943626	0.57488227	0.167	0.121	1

Spats2	0.4994599	-0.3224872	0.083	0.121	1
Igf1	0.5001113	-0.54839	0.25	0.333	1
Smim11	0.50012125	-0.3582788	0.125	0.182	1
Mrps36	0.50043018	0.29837322	0.167	0.091	1
Higd2a	0.50084255	-0.3968368	0.167	0.242	1
H2afy	0.50095232	0.47258986	0.125	0.121	1
Sharpin	0.50157815	-0.3435297	0.083	0.121	1
Plbd2	0.50169581	-0.3170504	0.083	0.121	1
Eif5	0.50179853	-0.494817	0.25	0.333	1
Cdk11b	0.50232678	-0.2635113	0.083	0.121	1
Acadm	0.5030586	0.54099079	0.208	0.182	1
Cd302	0.50344507	0.30596794	0.625	0.758	1
Adipoq	0.50352119	0.2582881	0.667	0.848	1
Ppp2ca	0.50362408	0.53260717	0.25	0.212	1
Rbbp7	0.50372908	0.5502812	0.333	0.303	1
Vmp1	0.50518247	0.33197006	0.292	0.212	1
Vwa5a	0.50541305	0.39144744	0.25	0.152	1
Rps8	0.5057828	0.30884216	0.792	0.758	1
Bod1l	0.50702621	0.2902335	0.167	0.091	1
Pdcd6ip	0.50722232	0.38080858	0.208	0.152	1
Mlxip	0.50876231	0.42618784	0.167	0.121	1
Smap1	0.50898902	0.51247875	0.208	0.182	1
Mfsd1	0.5090874	-0.3508962	0.125	0.182	1
Rpa3	0.50983551	-0.5518487	0.083	0.121	1
Pnpla8	0.51037962	-0.3209672	0.125	0.182	1
Plcg1	0.51145013	0.38043627	0.125	0.091	1
Gsr	0.51226788	-0.4822948	0.375	0.394	1
Zfp618	0.51257013	-0.3218362	0.125	0.212	1
Pth1r	0.51420138	0.6274363	0.167	0.121	1
Nono	0.51431703	-0.2658476	0.167	0.242	1
Higd1a	0.51529102	-0.3036696	0.083	0.121	1
Hnrnph1	0.51530805	-0.30531	0.333	0.394	1
Dctn6	0.5157944	-0.3952935	0.125	0.152	1
Mapre2	0.51654866	-0.31656	0.083	0.152	1
Ltbp1	0.51667479	0.71389639	0.333	0.333	1
Anapc11	0.51687098	-0.4071368	0.167	0.212	1
Cuedc2	0.51730523	0.31869849	0.125	0.091	1
Ckap4	0.51829038	0.62390399	0.25	0.242	1
Rab12	0.51957028	-0.2896756	0.125	0.182	1
Chmp5	0.52008016	0.40226992	0.292	0.242	1
Wbp1l	0.52090598	0.52799543	0.167	0.121	1
Ypel3	0.52127479	-0.4117726	0.125	0.182	1
Eif3i	0.52267493	-0.347161	0.125	0.152	1

Anp32b	0.52306809	-0.2565837	0.125	0.182	1
Agfg1	0.5235023	0.26322519	0.208	0.152	1
Mapk1	0.52362944	0.2766782	0.167	0.091	1
Mrfap1	0.52380935	0.62784418	0.375	0.303	1
Stx8	0.52413365	0.26611165	0.125	0.091	1
Flot1	0.52448847	-0.4422131	0.125	0.182	1
Ndnf	0.52505887	0.30092723	0.167	0.091	1
Ankrd10	0.52514407	0.33729678	0.125	0.091	1
Arpc2	0.52521846	0.53176711	0.292	0.242	1
Cfh	0.52529635	0.43134429	0.708	0.788	1
C1s1	0.52543894	0.63219318	0.375	0.364	1
Ranbp9	0.52567103	0.63363404	0.125	0.091	1
Pdgfa	0.52581888	0.3795299	0.167	0.121	1
Brwd1	0.52755963	-0.5394768	0.083	0.121	1
Rtn4rl1	0.52790532	-0.2513585	0.083	0.121	1
Rbm15	0.52812867	-0.3408924	0.083	0.121	1
Mocs2	0.52837874	0.25142907	0.292	0.212	1
Qars	0.52840853	0.33446547	0.167	0.121	1
Wapl	0.5286246	-0.3275324	0.125	0.152	1
Psen1	0.52877721	0.51692619	0.125	0.091	1
Sgms1	0.52889851	0.39235271	0.125	0.061	1
Hnrnpk	0.52985797	0.40870554	0.458	0.424	1
Vcp	0.5301787	0.32199029	0.375	0.303	1
Kng1	0.53036171	-0.4784334	0.25	0.303	1
Klhl24	0.53102833	-0.3244342	0.208	0.303	1
Fzd7	0.53152828	0.2701269	0.125	0.091	1
Bnip2	0.53233332	-0.4157493	0.125	0.152	1
Tmed2	0.53287134	0.33519439	0.25	0.182	1
Psmb8	0.53326875	-0.3922268	0.208	0.273	1
Eri3	0.53367421	0.36221431	0.167	0.091	1
Lysmd2	0.53440121	0.644034	0.167	0.152	1
Zcrb1	0.53546853	-0.3020244	0.167	0.212	1
Gm43672	0.53572911	0.27781532	0.125	0.061	1
Ctnnb1	0.53574134	0.39675225	0.417	0.333	1
Atp5j2	0.53597069	0.45597104	0.5	0.455	1
Snhg1	0.53649831	-0.5606879	0.208	0.212	1
Ddx18	0.5365883	-0.3790628	0.125	0.152	1
Thap12	0.53665193	0.41735026	0.167	0.121	1
Bmyc	0.53749672	0.30932471	0.208	0.152	1
Yif1b	0.53774587	0.45601265	0.167	0.121	1
AY036118	0.5378004	1.08684901	0.375	0.394	1
Sema4c	0.53780599	0.30793269	0.125	0.091	1
Zfp644	0.5382813	-0.355431	0.125	0.152	1

Rab20	0.53872187	-0.3092265	0.083	0.121	1
Rassf1	0.5390659	-0.4388367	0.083	0.121	1
Mydgf	0.54002488	-0.4554683	0.083	0.121	1
Eif4g1	0.54050424	-0.4401131	0.125	0.182	1
Htra3	0.54062067	0.63948848	0.125	0.091	1
Rnf11	0.5413775	-0.4744333	0.125	0.152	1
Smarca2	0.54303856	-0.5376956	0.125	0.152	1
Gls	0.54434103	-0.4526966	0.167	0.242	1
Eif3g	0.54597546	-0.3286227	0.125	0.182	1
Dtx3	0.54640516	0.59934591	0.125	0.121	1
Cltc	0.54668307	0.37458099	0.292	0.242	1
Lman2	0.54694103	-0.266981	0.083	0.152	1
Yae1d1	0.54767839	-1.5841963	0.083	0.121	1
Mecp2	0.54850164	-0.3329217	0.083	0.152	1
Prrx1	0.54853691	0.739991	0.25	0.212	1
Dyrk1a	0.54970276	0.40686219	0.167	0.121	1
Slco3a1	0.5500054	0.34134086	0.167	0.091	1
Ripk1	0.55046673	-0.4321824	0.125	0.152	1
Dync1h1	0.55049917	0.50764062	0.25	0.212	1
Baz2a	0.55152987	-0.3464767	0.083	0.121	1
Hsd17b12	0.5516292	0.6014569	0.125	0.091	1
Tut7	0.55197279	-0.4101445	0.167	0.212	1
Sfrp2	0.55275485	0.50584797	0.125	0.091	1
Ube2h	0.55290469	-0.5125295	0.208	0.212	1
Tns2	0.55298984	-0.368542	0.125	0.152	1
Git2	0.55316255	0.41073238	0.125	0.091	1
Pabpn1	0.55329603	-0.3674519	0.167	0.212	1
Dbi	0.55386269	0.39736287	0.5	0.424	1
Ro60	0.555287	0.46440994	0.125	0.091	1
Irf2	0.55572736	-0.356638	0.125	0.152	1
Sec61g	0.55675427	0.28657693	0.5	0.667	1
Phactr2	0.55783403	0.53449345	0.125	0.091	1
Calr	0.55818008	0.3758292	0.625	0.606	1
Ctdsp1	0.55835827	0.54622802	0.125	0.091	1
H13	0.55942903	-0.5795844	0.167	0.182	1
Serbp1	0.56029187	0.35326973	0.5	0.455	1
Tent4a	0.56083481	-0.257664	0.083	0.121	1
Rbm17	0.56088746	-0.2854315	0.083	0.121	1
Cyth3	0.56113754	-0.3212403	0.125	0.182	1
S100a1	0.56277008	0.36294682	0.125	0.091	1
Rpl7	0.56303629	0.44864144	0.458	0.424	1
Hacd2	0.56391201	0.30757519	0.125	0.091	1
C4b	0.56559633	0.50890381	0.667	0.636	1

P2rx4	0.56573064	0.65483726	0.167	0.152	1
Btf3	0.56651404	-0.273988	0.458	0.515	1
Dclk1	0.5678115	0.51822212	0.125	0.091	1
Rpl27	0.56858522	0.30731179	0.75	0.697	1
Grb10	0.56870147	-0.3145312	0.083	0.121	1
Eif3e	0.56913455	0.4683762	0.292	0.242	1
Ktn1	0.56920567	0.46716582	0.333	0.303	1
Spg20	0.56936013	0.32771544	0.125	0.061	1
Foxn3	0.57007593	0.33173094	0.25	0.212	1
Fbrs	0.57144685	0.30333213	0.167	0.091	1
Fkbp5	0.57230174	0.4572787	0.125	0.091	1
Ap2a2	0.57286044	-0.7503737	0.083	0.121	1
Nfix	0.5728707	0.45943249	0.333	0.273	1
Rras	0.57371885	0.40549504	0.208	0.182	1
Insig1	0.5742044	0.26066758	0.125	0.091	1
Ncl	0.57424333	-0.2561002	0.375	0.485	1
Fkbp8	0.57490651	0.306935	0.208	0.121	1
Tbl1xr1	0.57508451	0.68289222	0.167	0.152	1
Tspan17	0.57513117	0.33644043	0.167	0.121	1
Thbs1	0.57521104	-0.6166074	0.208	0.242	1
Ppp1r11	0.57915187	-0.2904281	0.125	0.182	1
Mzt2	0.57932965	-0.2598296	0.125	0.152	1
Atf6	0.57944797	0.49363131	0.167	0.152	1
Stx4a	0.57945643	-0.2697398	0.125	0.152	1
Zswim8	0.57964659	-0.4170678	0.083	0.121	1
9530068E07I	0.58062491	0.35862555	0.25	0.212	1
Csnk1g2	0.58075102	0.46637678	0.167	0.152	1
Smc6	0.58093277	-0.2847687	0.083	0.121	1
Churc1	0.58118745	-0.269991	0.125	0.152	1
Oxct1	0.58150364	-0.3246849	0.083	0.121	1
Eprs	0.58231436	0.51833315	0.167	0.152	1
Jak2	0.58249315	-0.311239	0.125	0.121	1
Pxk	0.58326969	0.34899534	0.125	0.091	1
Cdk8	0.58374295	-0.2794129	0.083	0.121	1
Fmo1	0.5844292	0.63506478	0.292	0.273	1
Pdcd5	0.58607449	0.38398809	0.208	0.152	1
Ndufa2	0.58625731	0.51417374	0.5	0.455	1
Gstm5	0.58636717	0.50027231	0.125	0.091	1
Spty2d1	0.58668491	0.34367984	0.208	0.182	1
Cmpk1	0.58855749	-0.2878215	0.083	0.121	1
Ipo7	0.58893811	0.36697454	0.167	0.121	1
Atp5k	0.58904704	0.52518316	0.5	0.455	1
Aldh3a1	0.58953178	-0.3345074	0.083	0.121	1

Dpep1	0.58967174	0.29526041	0.5	0.424	1
Stx7	0.58990608	-0.3754073	0.208	0.212	1
Prrc1	0.59057618	-0.3762032	0.125	0.152	1
Rbpms	0.59078275	-0.3053981	0.25	0.303	1
Tpi1	0.59202571	-0.3951008	0.167	0.182	1
Tnrc6a	0.59266317	0.55870974	0.25	0.242	1
Cdc14a	0.59271039	-0.3253037	0.083	0.152	1
Ubap2l	0.59322093	-0.3750442	0.125	0.182	1
U2af1	0.59351218	0.44376536	0.167	0.121	1
Ube2m	0.59367517	0.53679894	0.167	0.152	1
Kdm2a	0.59415608	-0.2533367	0.083	0.121	1
Snrpd3	0.59426963	-0.646451	0.25	0.273	1
Isg15	0.59454799	0.37602344	0.125	0.091	1
Gm15417	0.59821008	0.52942714	0.125	0.091	1
Cemip2	0.59869932	0.41687735	0.208	0.182	1
Rps20	0.59915862	-0.2904585	0.708	0.727	1
Abcc5	0.60012686	-0.2992552	0.167	0.242	1
Rpl35a	0.60471381	0.2606808	0.833	0.848	1
Ndufs4	0.60553989	0.30411418	0.125	0.242	1
Gsk3a	0.60569082	-0.3652042	0.083	0.121	1
Mafb	0.60640314	0.62037923	0.25	0.242	1
Slc39a14	0.60672219	-0.5373251	0.083	0.121	1
Tnfsf11	0.60732829	-0.6841208	0.167	0.182	1
Clic4	0.60952178	0.46435666	0.25	0.242	1
Ccser2	0.60964221	0.58524414	0.125	0.091	1
Csk	0.61084651	0.26781489	0.167	0.121	1
Anapc5	0.61089377	0.4139521	0.167	0.121	1
Prss23	0.61110847	0.54693903	0.333	0.273	1
Map4k4	0.61193004	0.60400102	0.375	0.394	1
Hypk	0.61200286	-0.3011697	0.125	0.152	1
Mapre1	0.61210509	-0.2531309	0.208	0.273	1
Ssh2	0.61279835	0.41605605	0.208	0.182	1
Bag6	0.61280639	-0.2734947	0.125	0.152	1
Sh3bp5	0.61331466	0.4271682	0.125	0.121	1
Asah1	0.61341585	0.27046964	0.167	0.121	1
Hsf2	0.6136571	0.753815	0.125	0.152	1
Naa60	0.61368262	-0.3409232	0.083	0.121	1
Sned1	0.61507497	-0.3272594	0.167	0.182	1
Eif4a1	0.61603786	-0.4091174	0.208	0.242	1
Cbln1	0.61762321	-0.2594854	0.083	0.121	1
Ifngr2	0.61786953	-0.2791668	0.125	0.182	1
Sppl2a	0.61874573	0.31482023	0.125	0.091	1
Cisd2	0.62024194	0.25771981	0.208	0.152	1

Emc1	0.62074594	-0.2940786	0.083	0.121	1
Tgfbr3	0.62113607	0.33815923	0.458	0.606	1
Slk	0.62266954	0.28603286	0.125	0.091	1
Morf4l2	0.62630747	0.65459712	0.167	0.182	1
Os9	0.62631077	0.29668561	0.125	0.091	1
Gigyf1	0.62764755	0.25765268	0.125	0.091	1
Zfas1	0.62881488	0.37687705	0.125	0.121	1
Piezo1	0.63000013	-0.3342082	0.125	0.152	1
Usp25	0.6308007	0.26397152	0.25	0.212	1
Mrpl48	0.63103705	0.30713646	0.167	0.121	1
Peak1	0.63121681	0.26853473	0.167	0.121	1
Dab2ip	0.63316871	-0.449349	0.167	0.182	1
Atp1a1	0.63365021	0.35675184	0.333	0.303	1
Heph	0.63406887	-0.2880162	0.125	0.152	1
Sars	0.63428805	-0.5084172	0.125	0.121	1
Mef2a	0.63558943	0.43267547	0.333	0.303	1
Zc3h15	0.63575781	-0.3755131	0.125	0.152	1
Pdlim2	0.63593593	0.49187181	0.208	0.182	1
Mc5r	0.63801828	0.39698454	0.125	0.091	1
2900097C17I	0.63826622	-0.3565574	0.208	0.212	1
Fyttd1	0.64117518	-0.2750888	0.167	0.182	1
Tubb5	0.64140613	0.66981117	0.25	0.273	1
Klf10	0.64225072	-0.2537485	0.083	0.121	1
Col27a1	0.64264357	-0.2553894	0.083	0.121	1
Tnrc18	0.64339627	0.53642758	0.167	0.182	1
Gnao1	0.64390138	-0.4896598	0.125	0.121	1
Bola3	0.64411018	-0.4364754	0.167	0.152	1
Ndufb9	0.64412374	-0.3836523	0.333	0.364	1
Sh3glb1	0.64427042	0.3675219	0.292	0.242	1
Rpl36a	0.64906029	-0.3014748	0.542	0.576	1
Ssr1	0.65115002	0.56759515	0.292	0.303	1
Zeb1	0.65120852	0.26017195	0.125	0.091	1
Slc39a7	0.65468951	0.38911862	0.167	0.152	1
Copa	0.65564848	-0.3465294	0.167	0.152	1
Ifnar1	0.65572641	-0.2816508	0.125	0.152	1
Ndufb1-ps	0.65595501	0.31744704	0.375	0.485	1
Stat1	0.65626898	-0.2878911	0.167	0.182	1
Rap1a	0.65833891	0.67151684	0.167	0.182	1
Gm26917	0.65920511	0.3206983	0.167	0.152	1
Odc1	0.66016964	-0.9665291	0.25	0.242	1
Med21	0.66230941	0.3177169	0.125	0.121	1
Lims1	0.66245074	-0.4013244	0.292	0.273	1
Wbp2	0.6628383	-0.2815463	0.125	0.152	1

Sav1	0.66331403	-0.3245206	0.167	0.182	1
Ctdspl	0.6645716	-0.3212773	0.167	0.152	1
Mrps21	0.66472547	0.32377615	0.292	0.242	1
Pdlim7	0.66599128	-0.4111287	0.125	0.121	1
Nufip2	0.66611784	-0.3509746	0.208	0.212	1
Scd1	0.66677518	-0.3380495	0.125	0.152	1
Gpc1	0.66689054	0.34031404	0.25	0.212	1
Scarb2	0.66726799	0.25601657	0.125	0.091	1
S1pr3	0.66736085	0.63944103	0.333	0.303	1
Atxn2l	0.66749731	-0.427479	0.167	0.152	1
Purb	0.66840581	-0.28912	0.208	0.242	1
Ttc12	0.66848186	-0.5088775	0.083	0.121	1
Gpx1	0.66872904	0.32925201	0.292	0.242	1
Srsf6	0.66956927	0.37362531	0.208	0.212	1
Mknk2	0.67354748	0.26099493	0.125	0.091	1
Suco	0.67401886	0.72035204	0.125	0.152	1
Yme1l1	0.67421771	0.41559483	0.208	0.182	1
Trip11	0.67607804	0.31829664	0.208	0.182	1
Nrbp1	0.67753017	0.26934179	0.125	0.091	1
Chd4	0.67855489	0.38547427	0.292	0.273	1
Gadd45g	0.68203434	-0.8042718	0.375	0.364	1
Eva1b	0.68209565	0.54958765	0.292	0.242	1
Bag3	0.68263943	-0.3964634	0.125	0.121	1
Gorasp2	0.68443956	-0.3874652	0.125	0.121	1
Ubr5	0.68510885	-0.2863205	0.25	0.273	1
Psmb3	0.68519043	0.2524908	0.375	0.333	1
Secisbp2l	0.6894055	0.71100258	0.208	0.182	1
Tnrc6c	0.68950952	0.52122626	0.208	0.212	1
Srsf11	0.69251171	0.28931284	0.25	0.212	1
Cd9	0.69308146	0.50372608	0.458	0.606	1
Mif	0.69311226	0.5128182	0.167	0.182	1
Rtraf	0.693369	-0.3578111	0.208	0.212	1
Naxe	0.69354937	0.4423004	0.125	0.121	1
Ubl3	0.69358046	-0.3065469	0.125	0.121	1
Clic1	0.69648712	0.26518282	0.333	0.273	1
Top2b	0.69726495	0.28121653	0.208	0.182	1
Wdr83os	0.69770455	0.28084609	0.25	0.212	1
Srsf2	0.69819018	-0.3024916	0.25	0.273	1
Ift43	0.69822287	-0.390637	0.125	0.121	1
Tsc22d1	0.69902382	-0.3527856	0.208	0.212	1
Abat	0.699879	0.28873171	0.125	0.091	1
Ifi207	0.70727678	0.34711233	0.125	0.121	1
Rps11	0.70911887	0.29047309	0.75	0.727	1

Nr3c1	0.71124122	-0.544907	0.167	0.182	1
1500009L16I	0.71215004	0.60566322	0.417	0.424	1
Sf1	0.71361626	0.42471798	0.208	0.212	1
Numa1	0.7144615	-0.2960927	0.25	0.242	1
Hnrnpa1	0.71576793	0.26396285	0.292	0.273	1
Uqcrh	0.71867568	0.36584737	0.542	0.485	1
Thsd7a	0.72011593	0.40103468	0.208	0.182	1
Smdt1	0.72131169	0.5281349	0.333	0.303	1
Rc3h1	0.72386077	0.38720576	0.167	0.152	1
Cox14	0.72426334	-0.3367088	0.167	0.182	1
Atp5mpl	0.72536091	-0.3057383	0.25	0.242	1
Smim8	0.72692677	0.30345674	0.125	0.121	1
Nfib	0.72852994	0.42859271	0.375	0.364	1
Hsp90b1	0.72872345	0.3548779	0.667	0.848	1
Prr13	0.72884477	-0.3191616	0.125	0.121	1
Cfdp1	0.72963416	0.25457334	0.167	0.152	1
Timp1	0.73005728	-1.2046412	0.292	0.303	1
Slc25a5	0.73388922	0.65602115	0.25	0.242	1
Senp6	0.73470707	-0.3300135	0.125	0.152	1
Agpat5	0.73484027	0.30500993	0.167	0.152	1
Fgfr1	0.73780631	-0.4265824	0.292	0.303	1
Sf3b4	0.73808499	0.34042583	0.125	0.121	1
Htatip2	0.74049746	0.32205171	0.125	0.091	1
Ccdc85b	0.74252315	0.3835476	0.208	0.182	1
S100a16	0.74447951	0.3256863	0.25	0.242	1
Ehd2	0.74477551	0.50618336	0.167	0.121	1
Pros1	0.74624012	-0.3907286	0.167	0.182	1
Ubtf	0.747629	-0.2971353	0.167	0.182	1
Supt4a	0.7480477	0.43399958	0.167	0.182	1
Crk	0.74917357	-0.3467919	0.167	0.152	1
Taok1	0.74974254	-0.2717744	0.125	0.121	1
Srrm1	0.74987116	0.4585731	0.25	0.212	1
Rbm3	0.75253339	-0.2868343	0.25	0.273	1
Yipf4	0.75469484	-0.3123155	0.125	0.121	1
Ndufs7	0.75894854	0.36191632	0.292	0.303	1
Myo1b	0.75920274	0.49701044	0.167	0.182	1
Pfdn5	0.75932849	0.48704731	0.5	0.545	1
mt-Cytb	0.75979158	0.42813288	0.958	1	1
Rps7	0.75990532	-0.3133665	0.542	0.545	1
Cib1	0.76256199	-0.3210797	0.167	0.182	1
Usp8	0.76482563	-0.3418283	0.125	0.091	1
Lgals9	0.76679787	0.39556435	0.125	0.121	1
Efr3a	0.76835294	0.4797209	0.083	0.121	1

Slc3a2	0.76861908	-0.2610417	0.125	0.121	1
Sdf4	0.77055328	0.2792917	0.25	0.242	1
Bcl10	0.77161721	-0.2758373	0.167	0.182	1
Slc44a1	0.77428319	-0.304354	0.125	0.091	1
Fubp1	0.77602332	0.37274294	0.167	0.152	1
Kansl1	0.77621966	0.27120746	0.167	0.182	1
Tbl1x	0.77736247	0.29624504	0.125	0.121	1
Ube2b	0.7787669	0.27137048	0.25	0.242	1
Rap1gds1	0.78004206	-0.3238899	0.125	0.121	1
Mga	0.78199259	0.44216118	0.125	0.121	1
Scarf2	0.78391659	0.27761185	0.125	0.121	1
Btg3	0.78503656	0.38055511	0.167	0.152	1
Sri	0.78658041	0.25132311	0.167	0.152	1
Gfpt1	0.78719195	0.2953298	0.125	0.121	1
Eef1a1	0.78752198	0.27488553	0.667	0.758	1
Ndufb5	0.78984361	-0.2839387	0.208	0.182	1
Tmem160	0.7902058	0.44641023	0.25	0.273	1
Kdm7a	0.79182989	0.25443483	0.25	0.273	1
C3	0.79203325	0.50190891	0.333	0.424	1
Tle5	0.7927806	0.30525793	0.125	0.121	1
Acbd6	0.79616039	-0.3450947	0.125	0.091	1
Arl4a	0.79704444	-0.2754523	0.125	0.121	1
Rsrp1	0.79766824	-0.329519	0.333	0.303	1
Rpl28	0.79944827	0.26749263	0.667	0.727	1
Ankrd11	0.80078954	0.27478231	0.208	0.212	1
Ctsb	0.8019422	0.32102224	0.542	0.606	1
Sfrp4	0.80224225	0.27200536	0.333	0.455	1
Commd3	0.80257753	0.30909328	0.167	0.182	1
Cd81	0.8027715	0.25306237	0.625	0.636	1
Ralb	0.80322833	0.30999101	0.125	0.091	1
Fis1	0.80552832	0.28328394	0.25	0.242	1
Mdh1	0.80952401	-0.301656	0.125	0.121	1
Ski	0.8112978	0.28985276	0.375	0.364	1
Smarce1	0.81422475	-0.2808101	0.125	0.091	1
Itih5	0.81577361	0.3204517	0.208	0.212	1
Parva	0.81820931	0.29022337	0.167	0.182	1
Fmc1	0.82213494	0.33172282	0.125	0.121	1
Scara5	0.82242283	0.26026656	0.125	0.121	1
4931406P16I	0.82339584	0.33465054	0.125	0.121	1
Tsen34	0.82494112	-0.2993624	0.125	0.152	1
Tspo	0.8260109	0.30161181	0.167	0.182	1
Utp11	0.82779473	0.2607152	0.167	0.152	1
Prpf40a	0.83111275	0.75194123	0.167	0.212	1

Nifk	0.83152214	0.47106637	0.083	0.121	1
Nid1	0.83271001	0.5295971	0.333	0.333	1
Sra1	0.83596278	0.27029761	0.125	0.152	1
Txnl1	0.83710712	-0.2686215	0.125	0.152	1
S100a10	0.84017454	-0.9017956	0.333	0.242	1
Tpm1	0.84071219	0.5610384	0.667	0.879	1
Esyt2	0.84359105	-0.2750066	0.167	0.152	1
Bax	0.84485275	0.35136676	0.167	0.182	1
Prelid1	0.84673446	0.47195152	0.083	0.121	1
Nfkb1	0.84915144	-0.5439628	0.208	0.182	1
Hnrnpul2	0.84919234	0.43581783	0.125	0.152	1
Son	0.84920955	0.30732559	0.667	0.727	1
Hist1h1e	0.85382236	0.2615937	0.125	0.152	1
Rps19	0.85542226	0.26913569	0.792	0.818	1
Tmcc1	0.85634477	0.30132352	0.125	0.121	1
Clstn1	0.85849564	0.40371948	0.208	0.273	1
Tmed5	0.8586061	-0.3954217	0.167	0.152	1
Gnas	0.85965808	0.43415344	0.583	0.697	1
Dctn4	0.86035225	0.33447689	0.25	0.242	1
Il6st	0.86108854	0.29845281	0.25	0.242	1
C1ra	0.86341658	0.25657028	0.5	0.515	1
Runx2	0.86458229	0.25774285	0.292	0.364	1
Hivep2	0.86630647	0.39531023	0.208	0.303	1
Ar	0.86740556	0.48949011	0.208	0.273	1
Ly6e	0.87208786	-0.2832488	0.167	0.121	1
Tomm6	0.87464835	0.33486134	0.375	0.455	1
Imp3	0.87481817	0.44229969	0.125	0.152	1
Trafd1	0.87695543	0.63294435	0.125	0.152	1
Vcl	0.88149584	-0.444493	0.167	0.121	1
Cwf19l2	0.88202877	-0.2900501	0.125	0.121	1
Rab5a	0.88444277	0.2632221	0.125	0.121	1
Lrp6	0.88902422	0.54609759	0.167	0.152	1
Fcgrt	0.8926264	0.31240297	0.375	0.424	1
Gja1	0.89455244	0.46209769	0.75	0.848	1
Vegfb	0.89522906	0.34754678	0.125	0.121	1
Sdc4	0.89801964	0.32742916	0.25	0.273	1
Fst	0.90242755	0.48797593	0.333	0.455	1
Ogdh	0.90613879	0.273693	0.125	0.121	1
Tm2d2	0.90886614	-0.2953306	0.125	0.121	1
Pkm	0.90963584	0.41316672	0.125	0.152	1
Dynll1	0.90980552	0.55564345	0.542	0.636	1
Sugt1	0.91499914	0.71576234	0.125	0.152	1
Mcee	0.91784927	0.46612727	0.167	0.212	1

Chd2	0.92089345	0.38555283	0.083	0.152	1
Psmb9	0.92573456	-0.2604978	0.167	0.152	1
Eif1ax	0.92581035	0.27159285	0.167	0.212	1
Sorbs2	0.92770479	0.44822641	0.208	0.242	1
Atp5md	0.9285049	0.31143932	0.458	0.515	1
Tagln2	0.93444017	-0.2627794	0.333	0.273	1
Bst2	0.93523615	0.29798111	0.333	0.364	1
Gapdh	0.9394896	0.28368364	0.25	0.303	1
Cdc42ep5	0.94243453	0.29314727	0.083	0.121	1
Usp7	0.94276255	0.33656064	0.125	0.152	1
Bag1	0.9459872	-0.2509364	0.125	0.121	1
Trim47	0.94695556	0.29826772	0.083	0.121	1
Dek	0.94783069	0.39798707	0.208	0.242	1
Cebpa	0.94892418	0.37431481	0.208	0.273	1
Vapa	0.94910299	-0.2955586	0.208	0.182	1
Crip2	0.94946302	0.32656881	0.167	0.242	1
Dpm3	0.94981566	0.32383067	0.417	0.485	1
Cox16	0.95239768	-0.2751086	0.167	0.152	1
Cox5b	0.9586242	0.2710408	0.333	0.364	1
Gnb1	0.96385247	0.38175403	0.25	0.273	1
Kidins220	0.96691391	-0.262813	0.125	0.091	1
Slc35a2	0.97222066	-0.2818259	0.167	0.121	1
Pole3	0.97551089	-0.2534558	0.125	0.091	1
Id4	0.97830302	0.32279924	0.208	0.273	1
Igfbp4	0.97920883	0.61500893	0.833	0.97	1
Bad	0.98297805	0.40606906	0.083	0.121	1
Spp1	0.98854453	-0.3633292	0.708	0.818	1
N4bp2l2	0.99259866	0.25385522	0.083	0.121	1
Ifitm2	0.99414177	0.48173608	0.583	0.667	1
